# The UK Coronavirus Job Retention Scheme and smoking, alcohol consumption and vaping during the COVID-19 pandemic: evidence from eight longitudinal population surveys

**DOI:** 10.1186/s12916-022-02511-0

**Published:** 2022-09-21

**Authors:** Michael J. Green, Jane Maddock, Giorgio Di Gessa, Bożena Wielgoszewska, Sam Parsons, Gareth J. Griffith, Jazz Croft, Anna J. Stevenson, Charlotte F. Huggins, Charlotte Booth, Jacques Wels, Richard J. Silverwood, Praveetha Patalay, Alun D. Hughes, Nishi Chaturvedi, Laura D. Howe, Emla Fitzsimons, Srinivasa Vittal Katikireddi, George B. Ploubidis

**Affiliations:** 1 MRC/CSO Social & Public Health Sciences Unit, University of Glasgow, Glasgow, UK; 2 MRC Unit for Lifelong Health and Ageing, University College London, London, UK; 3 Institute of Epidemiology and Health Care, University College London, London, UK; 4 Centre for Longitudinal Studies, UCL Social Research Institute, University College London, London, UK; 5 MRC Integrative Epidemiology Unit, University of Bristol, Bristol, UK; 6 Centre for Genomic and Experimental Medicine, University of Edinburgh, Edinburgh, UK

**Keywords:** Employment, Furlough, Cigarettes, E-cigarettes, Drinking

## Abstract

**Background:**

Employment disruptions can impact smoking and alcohol consumption. During the COVID-19 pandemic, many countries implemented furlough schemes to prevent job loss. We examine how furlough was associated with smoking, vaping and alcohol consumption in the UK.

**Methods:**

Data from 27,841 participants in eight UK adult longitudinal surveys were analysed. Participants self-reported employment status and current smoking, current vaping and alcohol consumption (>4 days/week or 5+ drinks per typical occasion) both before and during the early stages of the pandemic (April–July 2020). Risk ratios were estimated within each study using modified Poisson regression, adjusting for a range of potential confounders, including pre-pandemic behaviour. Findings were synthesised using random effects meta-analysis.

**Results:**

Compared to stable employment and after adjustment for pre-pandemic characteristics, furlough was not associated with smoking (ARR = 1.05; 95% CI: 0.95–1.16; *I*^2^: 10%), vaping (ARR = 0.89; 95% CI: 0.74–1.08; *I*^2^: 0%) or drinking (ARR = 1.03; 95% CI: 0.94–1.13; *I*^2^: 48%). There were similar findings for no longer being employed, and stable unemployment, though this varied by sex: stable unemployment was associated with smoking for women (ARR = 1.35; 95% CI: 1.00–1.82; *I*^2^: 47%) but not men (0.84; 95% CI: 0.67–1.05; *I*^2^: 0%). No longer being employed was associated with vaping among women (ARR = 2.74; 95% CI: 1.59–4.72; *I*^2^: 0%) but not men (ARR = 1.25; 95% CI: 0.83–1.87; *I*^2^: 0%).

**Conclusions:**

We found no clear evidence of furlough or unemployment having adverse impacts on smoking, vaping or drinking behaviours during the early stages of the COVID-19 pandemic in the UK. Differences in risk compared to those who remained employed were largely explained by pre-pandemic characteristics.

**Supplementary Information:**

The online version contains supplementary material available at 10.1186/s12916-022-02511-0.

## Background

The COVID-19 pandemic and associated virus-suppression measures have disrupted society worldwide [1, 2]. UK restrictions that required staying at home (except for limited purposes such as shopping or essential work), and closure of certain businesses (e.g. retail and leisure) came into effect from 23 March 2020 [3]. COVID-19 has especially disrupted economic activity, with many stopping work, losing income or reducing hours [4–6]. Several high-income countries have implemented furlough schemes to replace lost income and maintain employment for workers temporarily unable to carry out their job. The UK introduced the Coronavirus Job Retention Scheme (CJRS), providing 80% of pay (capped at £2500 a month) for employees who were unable to work during the pandemic [3]. Understanding the impact of such policies is crucial to inform the COVID-19 response internationally and inform international post-pandemic employment policy.

Smoking cigarettes and drinking alcohol damage health and contribute substantially to health inequalities [7–9]. Both are often social behaviours and disruptions to social and economic activity during the COVID-19 pandemic have the potential for considerable impact on smoking and drinking, though the evidence so far is mixed and suggests differential effects [10–14]. Employment-related disruptions may be important: while reductions in both income and social contact with other employees might be expected to limit smoking and drinking behaviour, these behaviours often increase after the loss of work [15–17]. Mechanisms posited to increase smoking and drinking include stress, financial anxieties, increased leisure time, and removal of work-place barriers. It is unclear whether similar effects would be seen for stopping work as part of a furlough scheme, or during a pandemic. Furlough could still reduce social contact, increase leisure time and remove work-place barriers, but mechanisms related to stress, financial concerns, and income loss may be mitigated because furlough is temporary and a portion of income is maintained. Stress and financial concerns could also be more ubiquitous in the context of a pandemic. Some evidence already indicates that furlough is associated with increased alcohol intake [14, 18].

Health impacts of the CJRS could differ, for example, by age, sex or socioeconomic position, so we need to understand who is most affected and to what extent this could modify existing inequalities [2, 6, 19]. Younger workers, women and low earners tend to work in industries or sectors where people are more likely to lose jobs or be furloughed [20]. Socioeconomic inequalities in smoking are well known, while evidence is more mixed for drinking [21–24]. Both behaviours tend to peak in young adulthood with either cessation or decreases thereafter [25, 26]. Additionally, there is evidence that younger generations drink and smoke less [27, 28] and that sex differences (namely that men tend to smoke and drink more) have narrowed [29, 30]. Vaping (i.e. use of electronic or e-cigarettes), a potentially less harmful alternative to smoking, has also become more common in recent years [31, 32].

The UK National Core Studies Longitudinal Health and Wellbeing initiative combines data from many of the UK’s largest, established population-based longitudinal studies, using coordinated analysis to answer priority pandemic-related questions, with a wealth of pre-pandemic sociodemographic and health data. Conducting harmonised primary analyses within each study and pooling results via meta-analysis, means we can provide more robust and nuanced evidence regarding pandemic impacts on population health and behaviours than is possible with any one study alone, and thereby support mitigation efforts. Here, we examine how changes in employment status during the pandemic, especially participation in the furlough scheme, are associated with smoking, vaping, and drinking behaviours. We further assess whether associations vary by sex, age or education.

## Methods

### Participants

Data were from a convenience sample of eight of the largest and most well-established UK population studies that conducted surveys before and during the COVID-19 pandemic. Details of the design, sample frames, current age range, timing of the most recent pre-pandemic and COVID surveys, response rates and analytical sample size are available in Additional file 1: Additional Tables.

Five of these were age homogenous birth cohorts (all individuals of similar age): the Millennium Cohort Study (MCS; [33, 34]); the children of the Avon Longitudinal Study of Parents and Children (ALSPAC-G1; [35]); Next Steps (NS, formerly known as the Longitudinal Study of Young People in England; [34, 36]); the 1970 British Cohort Study (BCS70; [34, 37]); and the 1958 National Child Development Study (NCDS; [34, 38]). Four age heterogeneous studies (covering a range of age groups) were also included: Understanding Society (USOC; [39]); the English Longitudinal Study of Ageing (ELSA; [40]); Generation Scotland: the Scottish Family Health Study (GS; [41]); and the parents of the ALSPAC-G1 cohort (ALSPAC-G0).

Analytical samples were restricted to participants of working age (16–66 years, based on the current UK state pension age; [42]) with at least one outcome in a COVID-19 survey between April and July 2020 (the period covered by the first UK lockdown restrictions) and valid data on all covariates. Most studies were inverse-probability weighted to be representative of their target populations, accounting for sampling design, and differential non-response [34]. Weights were not available for GS.

### Measures

Full details of questions and coding within each study are available in Additional file 2: Variable Coding.

#### Exposure: employment status change

Employment status change (or stability) was based on employment status both prior to the pandemic and at participants’ first COVID-19 survey (April–July 2020; see Additional file 1: Additional Tables for details): stable employed (reference); furloughed (i.e. from work to furlough); no longer employed (i.e. from work to non-work); became employed (i.e. from non-work to work); stable unemployed (i.e. unemployed but looking for work at both points); and stable non-employed (i.e. not employed at either point, including education, early retirement, caring for the home, sick or disabled). Given our primary interest in comparing the behaviours of those in stable employment (seen a priori as the optimal condition) to those who were furloughed, no longer employed and stable unemployed, we focus on these groups. The other employment categories were included in the modelling, but results are not presented as they were not of substantive interest.

#### Outcomes: smoking, vaping and drinking behaviours

We examined smoking, vaping and alcohol consumption. Smoking and vaping were based on any current use (yes vs. no). Regarding alcohol consumption, measures of frequency (4+ days per week vs. less frequently) and quantity per typical drinking occasion (5+ standard alcoholic drinks per occasion vs. less) could be coded consistently across most studies, and we created a combined binary variable indicating a high frequency, quantity or both (Additional file 3 includes separate analyses of frequency and quantity which were consistent with the main analyses). Primary analyses were based on studies/outcomes with measures both during and before the pandemic, whereas for some studies (ELSA/ALSPAC/GS) only information on change since the start of the pandemic was available for drinking. Thus, based on reported consumption before and during the pandemic, or on reported changes in behaviour, we created additional dichotomous variables indicating (in comparison to no change or change in the opposite direction): increased smoking (including take-up/relapse/or more cigarettes); decreased smoking (including cessation and fewer cigarettes); increased vaping (including take-up/relapse or more frequent use); decreased vaping (including cessation and less frequent use); increased drinking (increased frequency or more drinks per occasion or both); and decreased drinking (decreased frequency or fewer drinks per occasion or both). All information on behaviours during the pandemic was from surveys conducted during the first UK lockdown between April and July 2020 (inclusive).

#### Confounders and moderators

Confounders included sex (female vs. male); ethnicity (non-white ethnic minority vs. white -including white ethnic minorities); age; education (degree vs. no degree); UK nation (i.e. England, Wales, Scotland or Northern Ireland or other); household composition (based on having a spouse/partner and/or children); pre-pandemic psychological distress (indicated by symptoms above thresholds on standard screening scales); pre-pandemic self-assessed health (excellent–good vs. fair–poor); and pre-pandemic measures of smoking, vaping and drinking.

### Analysis

For each study, we first produced descriptive statistics examining the distribution of employment status change, both overall, and by sex, education and age group (age was split into three categories for all age-stratified analyses: 16–29; 30–49; and 50 years or more; age-homogeneous cohorts were included in the relevant age band). In each study, we also examined descriptive proportions for each outcome and for equivalent pre-pandemic measures of behaviour. For the main analyses, outcomes were regressed on employment status change within each study. Regressions were conducted using a modified Poisson model with robust standard errors that return risk ratios (RR), facilitating interpretation [43] and avoiding issues related to non-collapsibility of the odds ratio [44]. After estimating unadjusted associations, confounder adjustment was undertaken in two steps. First, a “basic” adjustment including sociodemographic characteristics: age (only in age-heterogeneous studies), sex, ethnicity (except the BCS and NCDS cohorts which were nearly entirely white), education, UK nation (except ALSPAC and ELSA which only had participants from a single country) and household composition. Second, a “full” adjustment additionally including pre-pandemic measures of psychological distress, self-rated health and each outcome behaviour. Sub-group analyses by sex, education and age group were performed with stratified regressions using the “full” adjustment. Both stages of adjustment are relevant because employment change incorporates pre-pandemic employment status, which may have influenced pre-pandemic characteristics such as mental health, self-rated health and outcome behaviours (see Additional file 1: Additional Tables, Figure S8). For the outcomes directly capturing changes in health behaviour, the “full” adjustment did not include pre-pandemic levels of the behaviour in question (as this was now part of the outcome) and so associations with these outcomes may partially reflect associations with pre-pandemic behaviour.

Within-study analyses were conducted using Stata or R. Overall and stratified results from each study were pooled using a random effects meta-analysis with restricted maximum likelihood (*meta* command in Stata). Tests of group differences were performed using the subgroup option. Some studies could not contribute estimates for every comparison due to differences in the ages sampled, measures used and sparsity of data [45]. For a small number of exposure levels the number of outcome cases was low (≤2) and estimates were deemed as unreliable and excluded. While selective exclusion could potentially lead to bias, the low numbers of events mean that the corresponding within-study estimates were so imprecise that their exclusion is unlikely to be impactful (see Additional file 3 for more details and sensitivity to different low cell count exclusion thresholds). We report risk ratios (RR), adjusted risk ratios (ARR), 95% confidence intervals (95% CI) and heterogeneity using the *I*^2^ statistic: 0% indicates estimates were similar across studies, while values closer to 100% represent greater heterogeneity.

## Results

### Descriptives

**Fig. 1:**
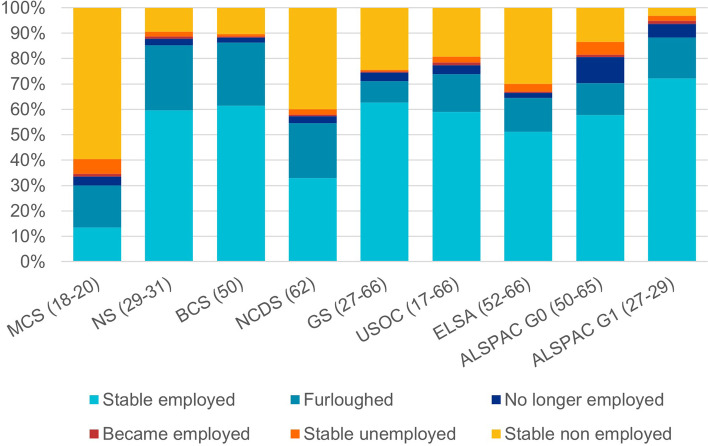
**Patterns of employment status change in the initial stages of the pandemic by study.** Parentheses show the age range of participants in each study sample at the time of data collection. Weighted data (except GS). Analysis for GS, USOC, and ELSA restricted to participants aged 66 and younger. Additional file 2 details the questions asked in each study

Analyses included 27,841 individuals from eight studies (see Additional file 1: Table S3 for sample characteristics). Figure 1 shows patterns of employment status change from pre-pandemic to those recorded in April–July 2020. Around six in 10 participants in NS, BCS, GS, USOC and ALSPAC G0 were employed before and during the initial stages of the pandemic. Prevalence of furlough ranged between 8% (GS) and 26% (NS), while approximately 3% of participants were no longer employed during the pandemic (10% in ALSPAC G0). Stable unemployment ranged in prevalence between 1% (GS) and 6% (MCS). Additional file 1: Table S4 shows how the economic activity was patterned by education, sex and age groups. Furlough was generally more common among younger, female and less educated participants than others, while stable employment was especially common among male, more highly educated and middle-aged participants.

Table 1 shows the prevalence of smoking, vaping and drinking, and changes from pre-pandemic behaviour, in each study. Only USOC and ELSA had comparable data on smoking prevalence both before and during the pandemic, but proportions were similar during the pandemic (in the MCS, NS, BCS and NCDS cohorts, for example, the questions on frequency of pre-pandemic smoking were only asked for those who reported smoking during the pandemic). Vaping was less common than smoking and the pre-pandemic prevalence from USOC (7.5%) was similar to that during the pandemic (6.6%). Proportions of drinking alcohol on more than 4 days a week had risen considerably in many studies (e.g. 28% to 52% in USOC) but not all (9% to 10% in MCS). Nevertheless, many individual participants still reported reducing the frequency of their drinking. All studies showed either decline or stability in proportions reporting five or more drinks per typical drinking occasion (e.g. 31% to 11% in MCS), though a range of 9–31% across all studies reported increased quantities of alcohol per drinking occasion during the pandemic.

**Table 1:** Percent (and *N*) distribution of health behaviours and changes during the pandemic by study

	MCS	NS	BCS	NCDS	GS	USOC	ELSA	ALSPAC G0	ALSPAC G1
*N* participants	2057	1579	3151	4358	2604	8328	2417	2072	1275
Age/age range of participants	18–20	29–31	50	62	27–66	17–66	52–66	50–65	27–29
	**% (*N*)**	**% (*N*)**	**% (*N*)**	**% (*N*)**	**% (*N*)**	**% (*N*)**	**% (*N*)**	**% (*N*)**	**% (*N*)**
**Smoking and vaping**									
Pre-pandemic current smoker	NA	NA	NA	NA	12.2 (317)	15.5 (834)	14.7 (271)	NA	NA
Current smoker (during pandemic)	17.3 (292)	18.0 (189)	16.7 (366)	11.3 (356)	6.2 (162)	14.7 (793)	13.3 (240)	NA	NA
Smoking more -including relapse and initiation	5.4 (78)	8.5 (76)	6.5 (136)	2.9 (85)	2.7 (71)	6.3 (356)	4.4 (77)	NA	NA
Smoking less -including cessation	6.9 (137)	4.0 (46)	2.5 (57)	1.0 (41)	1.0 (24)	6.8 (339)	4.2 (83)	NA	NA
Pre-pandemic current vaping	NA	NA	NA	NA	NA	7.5 (490)	NA	NA	NA
Current vaping (during pandemic)	7.4 (122)	10.2 (106)	9.5 (228)	5.7 (189)	3.3 (87)	6.6 (423)	NA	NA	NA
Vaping more—including relapse and initiation	3.0 (51)	5.0 (52)	2.5 (67)	1.0 (41)	1.0 (26)	2.3 (124)	NA	NA	NA
Vaping less—including cessation	1.4 (27)	1.2 (13)	0.5 (15)	0.4 (16)	< 1.0 (< 10)	3.3 (188)	NA	NA	NA
**Alcohol consumption**									
Drinks 4+ days a week (pre-pandemic)	8.6 (143)	6.2 (72)	15 (496)	24 (1,145)	NA	28.2 (2314)	NA	NA	NA
Drinks 4+ days a week (during the pandemic)	9.6 (151)	13.8 (197)	27.0 (911)	29.7 (1,405)	NA	51.7 (4272)	NA	NA	NA
Drinks more frequently	17.4 (351)	30.5 (492)	25.3 (883)	15.4 (707)	NA	34.6 (3009)	NA	NA	NA
Drinks less frequently	36.2 (786)	14.1 (222)	8.0 (242)	12.4 (519)	NA	24.4 (1904)	NA	NA	NA
Drinks 5+ drinks per occasion (pre-pandemic)	30.8 (687)	12 (180)	12 (329)	9.3 (372)	NA	16.8 (1040)	NA	NA	NA
Drinks 5+ drinks per occasion (during the pandemic)	10.6 (171)	8.6 (102)	11.7 (325)	9.0 (333)	NA	6.9 (485)	NA	NA	NA
More drinks per occasion	17.4 (351)	30.5 (492)	25.3 (883)	15.4 (707)	NA	9.4 (743)	NA	NA	NA
Fewer drinks per occasion	46.5 (782)	20.2 (239)	10.4 (242)	9.8 (297)	NA	36.6 (2695)	NA	NA	NA
Drinks 4+ days/week or 5+ drinks per occasion (pre-pandemic)	35 (748)	17.9 (247)	24.4 (742)	28.9 (1325)	NA	42.8 (3200)	NA	NA	NA
Drinks 4+ days/week or 5+ drinks per occasion (during the pandemic)	18.2 (282)	21.0 (273)	33.4 (1054)	34.8 (1507)	NA	55.8 (4536)	NA	NA	NA
Drinks more/more frequently	23.7 (396)	36.7 (541)	31.7 (1025)	21.1 (889)	26.3 (685)	38.4 (3329)	18.1 (455)	38.1 (789)	13.7 (175)
Drinks less/less frequently	58.4 (921)	26.9 (314)	14.2 (343)	15.3 (497)	15.2 (394)	42.3 (3217)	14.3 (318)	35.1 (730)	38.9 (496)

*MCS* Millennium Cohort Study, *NS* Next Steps, *BCS* 1970 British Cohort Study, *NCDS* National Child Development Study, *GS* Generation Scotland, *USOC* Understanding Society, *ELSA* English Longitudinal Study of Aging, *ALSPAC* Avon Longitudinal Study of Parents and Children (G0 = parents, G1 = children). For more information about the questions asked in each dataset, and how the change variables were coded, please see Additional file 2. Percentages are weighted (except GS). Note: Analysis for GS, USOC, and ELSA restricted to participants aged 66 and younger. *NA* not available

### Pooled analysis

**Fig. 2:**
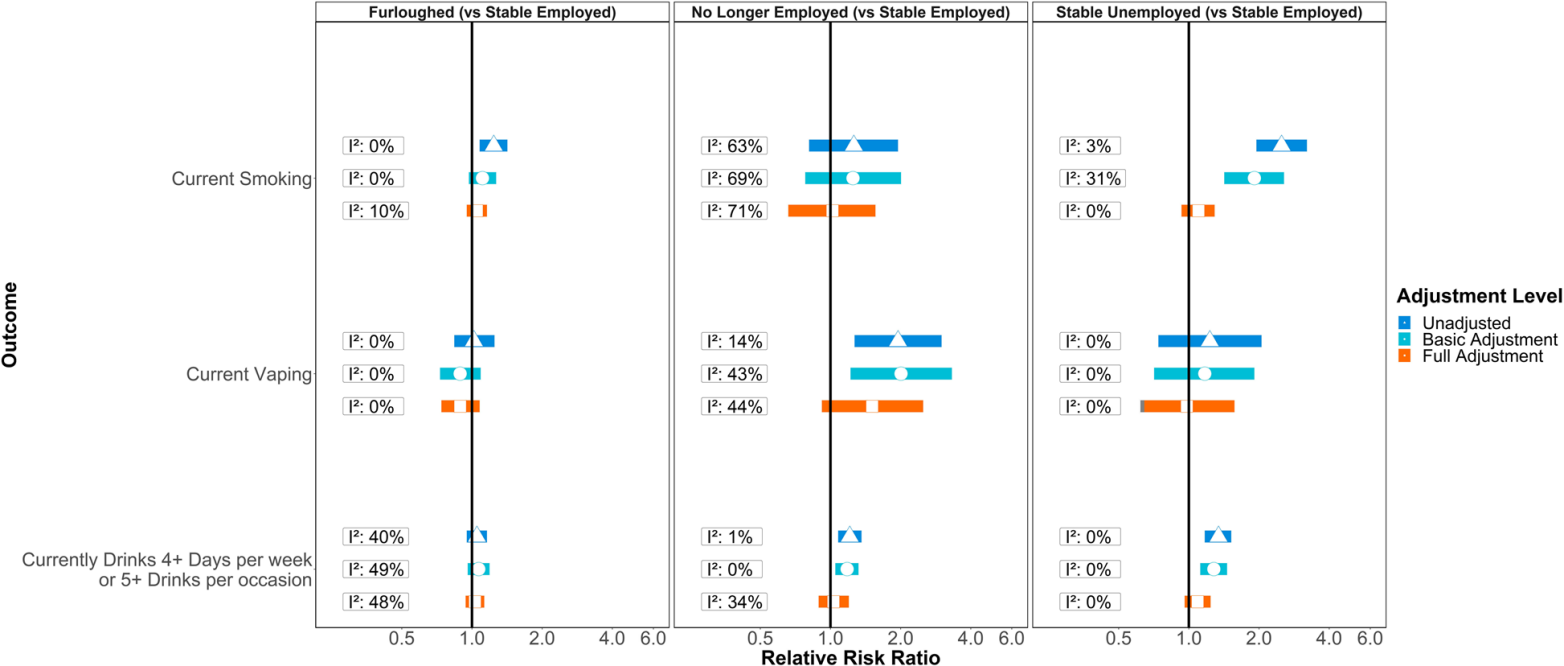
**Associations between changes in employment status during the pandemic and health behaviours in pooled analyses across eight UK longitudinal studies.** ‘Basic’ adjustment includes age, sex, ethnicity, education, UK nation, and household composition. ‘Full’ adjustment additionally includes pre-pandemic measures of mental health, self-rated health, smoking, vaping and drinking

**Fig. 3:**
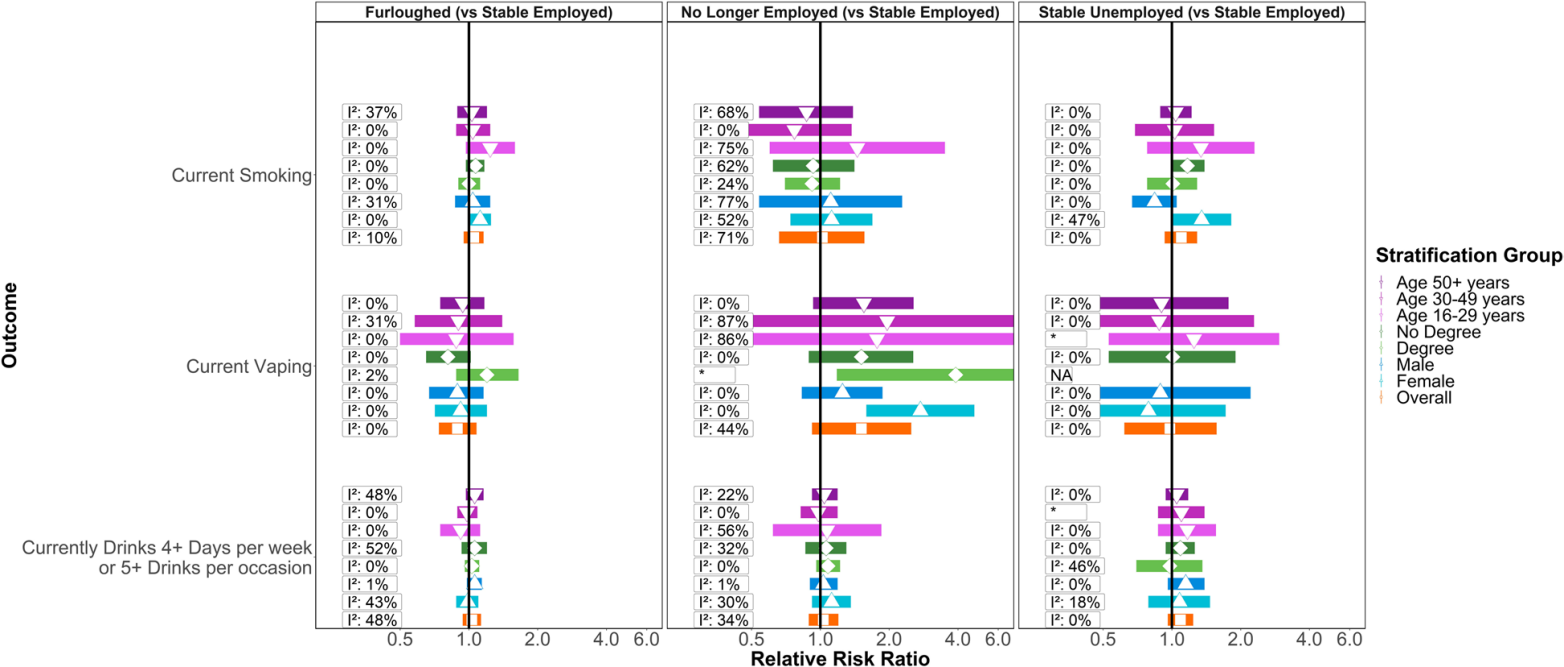
**Associations between changes in employment status during the pandemic and health behaviours, stratified by age, sex and educational attainment.** *No *I*^2^ value as only one study was able to provide an estimate. NA = no studies were able to provide reliable estimates

Figure 2 shows meta-analysis estimates from unadjusted, basic adjusted and fully adjusted models for smoking, vaping and alcohol consumption during the pandemic. Figure 3 shows pooled estimates from fully adjusted models stratified by sex, education and age. Stratified estimates were largely consistent with the main results, though we highlight some differences below. Full details of the meta-analysis including overall and stratified estimates from each study are available in Additional file 3 and Additional file 4.

#### Furlough

Compared to stable employment, unadjusted estimates indicated greater risk of smoking among participants who were furloughed (RR = 1.24; 95% CI: 1.08–1.42; *I*^2^: 0%), but little difference in risk of vaping (RR = 1.02; 95% CI: 0.84–1.25; *I*^2^: 0%) or drinking (RR = 1.05; 95% CI: 0.95–1.16; *I*^2^: 40%). The difference in smoking was attenuated with adjustment for sociodemographic characteristics (ARR = 1.11; 95% CI: 0.97–1.27; *I*^2^: 0%) and further attenuated with adjustment for pre-pandemic mental health, self-rated health and behaviour (ARR = 1.05; 95% CI: 0.95–1.16; *I*^2^: 10%). In the stratified analyses with full adjustment, furlough was still associated with smoking among women (ARR = 1.12; 95% CI: 1.00–1.25; *I*^2^: 0%), but this estimate did not clearly differ (*p*-value: 0.48) from the estimate among men (ARR = 1.04; 95% CI: 0.87–1.24; *I*^2^: 31%). There was also evidence (p-value: 0.05) that furlough was associated with lower risk of vaping for those without degree-level education (ARR = 0.81; 95% CI: 0.65–1.02; *I*^2^: 0%), with the association in the opposite direction for those with a degree (ARR = 1.20; 95% CI: 0.88–1.65; *I*^2^: 2%).

#### No longer employed

Compared to stable employment, those no longer employed during the pandemic had an increased risk of vaping (RR = 1.95; 95% CI: 1.27–3.00; *I*^2^: 14%) and drinking (RR = 1.21; 95% CI: 1.08–1.36; *I*^2^: 15%) and there was some indication of increased risk for smoking (RR = 1.26; 95% CI: 0.81–1.95; *I*^2^: 63%), but confidence intervals were wide and over-lapped the null. Adjusting for sociodemographic characteristics, mildly attenuated the estimate for drinking (ARR = 1.18; 95% CI: 1.05–1.32; *I*^2^: 0%), while those for vaping and smoking were largely unchanged. No clear differences remained in the overall analyses when we additionally adjusted for pre-pandemic health and behaviour. In stratified analyses however, even with full adjustment, the association between no longer being employed and risk of vaping was clearly discernible among women (ARR = 2.74; 95% CI: 1.59–4.72; *I*^2^: 0%) but weaker for men (ARR = 1.25; 95% CI: 0.83–1.87; *I*^2^: 0%; p-value: 0.02).

#### Stable unemployment

Participants in stable unemployment had a higher risk of current smoking (RR = 2.50; 95% CI: 1.95–3.21; *I*^2^: 3%) and drinking (RR = 1.34; 95% CI: 1.17–1.52; *I*^2^: 0%) compared to stable employment, and the higher risk of smoking was of greater magnitude than that associated with being furloughed or no longer employed. No clear differences were present for vaping. The difference in risk for smoking was partially attenuated with adjustment for sociodemographic characteristics (ARR = 1.91; 95% CI: 1.42–2.56; *I*^2^: 31%), but more fully attenuated with adjustment for pre-pandemic health and behaviours (ARR = 1.10; 95% CI: 0.93–1.29; *I*^2^: 0%). Sociodemographic adjustment made little difference to the association with drinking (ARR = 1.28; 95% CI: 1.12–1.46; *I*^2^: 0%), but adjustment for pre-pandemic health and behaviours attenuated the association (ARR = 1.09; 95% CI: 0.96–1.24; *I*^2^: 0%). In stratified analyses, stable unemployment remained associated with risk of smoking for women (ARR = 1.35; 95% CI: 1.00–1.82; *I*^2^: 47%), even after full adjustment, but not for men (0.84; 95% CI: 0.67–1.05; *I*^2^: 0%; *p*-value: 0.01).

#### Changes in behaviour

Pooled estimates for outcomes indicating changes in smoking, vaping or drinking behaviour are presented in Additional file 1: Tables S5-S7. Compared to stable employment, and with adjustment for sociodemographic and other pre-pandemic characteristics, furlough was associated with increased vaping and drinking. Stratified analyses (see Additional file 4) showed that the association between furlough and vaping was particularly evident among those with degree-level education (ARR = 2.93; 95% CI: 1.80–4.75; *I*^2^: 0%), rather than those without (ARR = 1.31; 95% CI: 0.79–2.19; *I*^2^: 51%; p-value: 0.03), while that for drinking was evident among men (ARR = 1.33; 95% CI: 1.08–1.64; *I*^2^: 74%) but not women (ARR = 1.02; 95% CI: 0.93–1.12; *I*^2^: 24%; p-value: 0.02).

## Discussion

Coordinated analyses in eight UK longitudinal population surveys found that, compared to stable employment, furlough was associated with a greater risk of smoking during the early stages of the COVID-19 pandemic (April–July 2020). The magnitude of this greater risk was similar to that for no longer being employed but smaller than for stable unemployment. Furlough was not associated with excess risk of vaping or drinking, compared to stable employment, while no longer being employed was associated with higher risk for both outcomes. All of these differences were largely explained when accounting for sociodemographic characteristics and pre-pandemic health (mental and physical) and behaviour, though this did differ by sex. Among women but not men, furlough and stable unemployment remained associated with risk of smoking (the magnitude of excess risk was greater for stable unemployment than for furlough), and no longer being employed remained associated with vaping, even after adjusting for pre-pandemic characteristics.

Other evidence indicates declines in smoking and vaping during the pandemic [10], with increases in smoking cessation attempts and successful quitting [12]. Meanwhile, the frequency of drinking and binge drinking have increased [10, 13], but younger cohorts have reduced their drinking [46]. There has been little evidence on how employment disruptions have contributed, though some small-scale and self-selected surveys show associations between furlough and increased drinking [14, 18]. New unemployment has previously been associated with increases in smoking and drinking [15–17], so furlough could potentially have adversely impacted on smoking and drinking behaviour, but the preservation of income and recognisably temporary nature of furlough may have softened usual impacts of ceasing work. After adjusting for pre-pandemic characteristics, there was little evidence of adverse impacts. We found only that furlough was associated with a higher risk of smoking among women, and with increased alcohol consumption among men and that only when specifically analysing the self-reported change in drinking.

Where we did find associations, these were weak enough to be plausibly explained by unmeasured confounding. Furthermore, there was no clear evidence that the association between furlough and smoking for women was actually stronger than that among men: the overall analyses did not show a clear relationship. The association between furlough and increased drinking for men was not consistently apparent across all analyses, as would be expected if it were a strong and robust causal relationship: the main analyses did not support this, either before or after adjustment for pre-pandemic characteristics (including pre-pandemic drinking). This discrepancy could be because: associations with change outcomes can include effects of employment on pre-pandemic drinking; or because analyses of change were sensitive to relatively minor changes in behaviour above or below the threshold used to identify heavy drinking in the main analyses (and such minor changes may have less overall relevance for health). Measurement may be especially important, as a previous study only found associations with furlough for heavy episodic drinking [14], rather than for frequency or quantity measures. There was also an indication that furlough was associated with increased vaping, but some or all of this may represent moves towards smoking cessation. Thus, furlough does not appear to have had any clear, adverse impacts on smoking, vaping or drinking behaviour. Loss of work has previously been shown to be detrimental, being associated with increases in smoking and alcohol consumption [16, 17]. The lack of such associations with furlough, at least in the early stages of the pandemic (when furlough was at its peak), suggests the policy, as an alternative to job loss, may have been protective.

Combining analyses from eight UK prospective studies (six of which were nationally representative of their target age range) makes a clear contribution to understanding the potential impact of furlough, but limitations should be taken into account. We were not able to achieve full harmonisation of measures across studies, for example, a number of studies had only asked questions on recent pre-pandemic smoking of those who were smoking during the pandemic, meaning smoking cessation during the pandemic was unobserved in those studies. This means the analyses of change in smoking for these studies focused only on reductions in cigarettes smoked, rather than outright cessation. Main analyses will be less affected, though there may be some potential residual confounding from participants who had smoked but given up before being surveyed during the pandemic. Maximising comparability of measures across studies limited our scope to explore varied definitions with respect to frequency, quantity or other aspects of use, such as binge drinking or concurrence of smoking and vaping behaviour. Our findings represent the early stages of the pandemic (April–July 2020) in the UK. Findings may not necessarily generalise to other countries and relationships could change with: the duration of lockdown or furlough; subsequent changes to either; or even with seasonal changes in weather. Further research could explore heterogeneity over the course of the pandemic (e.g. as seasons or restrictions change), within workers who retained employment or who were furloughed from differing occupational classes or industries, and between different outcome measurement definitions/thresholds.

As with most observational studies, unobserved confounding could have affected our estimates. We did not adjust for occupational class (since it was unobservable for those who had not been employed), and there may have been differences between participants whose jobs were retained, versus those who experienced furlough or job loss. Our fully adjusted models account for differences in key pre-pandemic characteristics among employment groups or industry sectors (self-rated health, mental health, smoking, vaping and alcohol consumption), but associations with furlough could reflect other traits of these employment groups, such as how workers in different industries were responding to the pandemic, rather than being effects of furlough specifically. Nevertheless, since we largely did not find differences in behaviour to be associated with furlough, it seems implausible that confounding from occupational characteristics somehow obscured the presence of a genuine effect. Despite being embedded in long-standing cohorts, pandemic survey responses were selective, and while weighting was employed to correct for this, bias due to selective non-response cannot be excluded [47]. Adjustment for pre-pandemic characteristics was also important, but may have introduced bias in estimates if there were unobserved determinants of both pre-pandemic characteristics and behaviour during the pandemic [48].

Furlough was considerably more common in the early stages of the pandemic than being no longer employed or in stable unemployment. Therefore, while meta-analyses of furlough exhibited low heterogeneity between estimates (<50%), estimates for these latter groups were based on small numbers with more heterogeneity and considerable imprecision, especially in stratified analyses, or for rarer outcomes like vaping or change in smoking. Nevertheless, confidence intervals aside, the estimated magnitude of the associations for no longer being employed and stable unemployment were close to the null after adjusting for pre-pandemic characteristics (the association between no longer being employed and risk of vaping was the clearest exception to this pattern). Moving out of employment within the context of the pandemic may have different impacts compared to more typical labour market circumstances.

The lack of association that we find between furlough and either smoking or alcohol consumption may indicate that stress-related mechanisms are especially important for the increases in these behaviours normally associated with loss of work. A qualitative study on smoking during the pandemic identified stresses associated with confinement, removal of barriers/distractions, curtailment of social routines and feelings of boredom as mechanisms that could contribute to increased smoking [49], and these are similar to the mechanisms posited for increases in smoking and drinking behaviour following loss of work [15, 16]. Compared to the loss of work under normal circumstances, furlough will likely still have removed some work-place barriers and increased leisure time or boredom, but stress-related mechanisms would be mitigated by the maintained income and temporary nature of furlough. Furthermore, during a pandemic, even people in stable employment have had their life and working patterns disrupted, for example, by having to start working from home or make other adaptations, so stress is less-specifically associated with stopping work (by furlough or otherwise). The ubiquity of disruption may therefore help explain the lack of association observed overall, with clear differences only emerging in sub-groups or additional analyses of behaviour change.

With respect to vaping, the clearest association observed was for workers who were no longer employed during the pandemic. We interpret this cautiously, since numbers in this group were small and vaping was rare, but positively since most vapers were or had been smokers, and this group also decreased their cigarette use. While not what our study was designed to address, this is consistent with e-cigarettes being used as an aid to reducing or quitting smoking [50, 51] among a disadvantaged group (socioeconomically disadvantaged groups have historically tended to be less successful with smoking cessation). There was also evidence indicating that furlough was associated with less vaping among those without degree-level education. Again, we should be cautious given the small numbers involved, and it may simply be that the finding is clearest in this group because the vaping prevalence is higher among those with less education (who are more likely to smoke). Decreased vaping associated with furlough is a potential concern but since there was not evidence of raised risk for smoking in this group, it is unlikely to represent a tendency for furloughed participants to choose smoking over vaping. It may be that furloughed respondents with less education find it more difficult to obtain e-cigarette devices and liquids when they are remaining at home more.

## Conclusions

Alongside the beneficial role of furlough in mitigating adverse economic impacts of the pandemic, investigations with data from eight longitudinal UK population surveys found little evidence of any detrimental impacts of furlough on smoking, vaping and drinking behaviours relative to remaining in employment. This supports at least the short-term use of such job-retention policies for economic mitigation during public health crises. Indeed, no longer being employed and stable unemployment were not clearly associated with these behaviours after accounting for pre-pandemic characteristics either. Employment status disruption does not appear to have been a clear driver of smoking, vaping or drinking behaviours during the early stages of the pandemic in the UK.

## Supplementary Information

**Additional file 1: Table S1**. Description of Studies. **Table S2**. Ethics and data access statements for each study. **Table S3**. Sample characteristics by study. **Table S4**. Employment status change by sex, education, and age-group. **Table S5**. Meta-analysed risk ratios and heterogeneity estimates for associations between changes in employment status and drinking behaviour: unadjusted, basic & full adjustment results. **Table S6**. Meta-analysed risk ratios and heterogeneity estimates for associations between changes in employment status and smoking: unadjusted, basic & full adjustment results. **Table S7**. Meta-analysed risk ratios and heterogeneity estimates for associations between changes in employment status and vaping: unadjusted, basic & full adjustment results. **Figure S8**. Causal pathways blocked under differing levels of adjustment.

**Additional file 2.** Variable Coding.

**Additional file 3. **Meta-Analysis. **Table 1**. Main analysis excluding studies with ≤5 cell counts for exposure-outcome. **Table 2**. Main analysis excluding studies with ≤2 cell counts for exposure-outcome. **Table 3**. Main analysis excluding studies with zero cell counts for exposure-outcome. **Table 4**. Analysis of change excluding studies with ≤ 5 cell counts. **Table 5**. Analysis of change excluding studies with ≤ 2 cell counts. **Table 6**. Analysis of change excluding studies with zero cell counts. **Figure set 1**. Currently drinks 4+ days/week or 5+ drinks/occasion. **Figure set 2**. Increased alcohol consumption. **Figure set 3**. Reduced alcohol consumption. **Figure set 4**. Currently drinks 5+ drinks/occasion. **Figure set 5**. Drinks more alcohol units per occasion. **Figure set 6**. Drinks fewer alcohol units per occasion. **Figure set 7**. Currently drinks 4+ days/week. **Figure set 8**. Drinks more frequently. **Figure set 9**. Drinks less frequently. **Figure set 10**. Current smoker. **Figure set 11**. Smoking more. **Figure set 12**. Smoking less. **Figure set 13**. Current vaper. **Figure set 14**. Vaping more. **Figure set 15**. Vaping less.

**Additional file 4. **Stratified Analysis (Forest plots)**. Figure set 1 16 31**. Currently drinks 4+ days/week or 5+ drinks/occasion. **Figure set 2 17 32**. Increased alcohol consumption. **Figure set 3 18 33**. Reduced alcohol consumption. **Figure set 4 19 34**. Drinks 5+ drinks/occasion. **Figure set 5 20 35**. Drinks more alcohol units per occasion. **Figure set 6 21 36**. Drinks fewer alcohol units per occasion. **Figure set 7 22 37**. Currently drinks 4+ days/week. **Figure set 8 23 38**. Drinks more frequently. **Figure set 9 24 39**: Drinks less frequently. **Figure set 10 25 40**. Current smoker. **Figure set 11 26 41**. Smoking more. **Figure set 12 27 42**. Smoking less. **Figure set 13 28 43**. Current vaper. **Figure set 14 29 44**. Vaping more. **Figure set 15 30 45**. Vaping less.

## Data Availability

All datasets included in this analysis have established data sharing processes, and for most included studies the anonymised datasets with corresponding documentation can be downloaded for use by researchers from the UK Data Service. We have detailed the processes for each dataset in Additional file 1: Table S2.

## Abbreviations

ARR: Adjusted risk ratio
ALSPAC-G1: Avon Longitudinal Study of Parents and Children
ALSPAC-G0: Parents of ALSPAC-G1
BCS70: 1970 British Cohort Study
CI: Confidence interval
CJRS: Coronavirus Job Retention Scheme
ELSA: English Longitudinal Study of Ageing
GS: Generation Scotland: the Scottish Family Health Study
MCS: Millennium Cohort Study
NCDS: 1958 National Child Development Study
NS: Next Steps (formerly the Longitudinal Study of Young People in England).
UK: United Kingdom
USOC: Understanding Society
