# Supplementary material for: The UK Coronavirus Job Retention Scheme and smoking, alcohol consumption and vaping during the COVID-19 pandemic: evidence from eight longitudinal population surveys
Source: BMC Med. 2022 Sep 21;20:345. doi: 10.1186/s12916-022-02511-0 (PMC9489267; doi:10.1186/s12916-022-02511-0)
Supplement: Supplementary file 1 — Additional file 1: Table S1. Description of Studies. Table S2. Ethics and data access statements for each study. Table S3. Sample characteristics by study. Table S4. Employment status change by sex, education, and age-group. Table S5. Meta-analysed risk ratios and heterogeneity estimates for associations between changes in employment status and drinking behaviour: unadjusted, basic & full adjustment results. Table S6. Meta-analysed risk ratios and heterogeneity estimates for associations between changes in employment status and smoking: unadjusted, basic & full adjustment results. Table S7. Meta-analysed risk ratios and heterogeneity estimates for associations between changes in employment status and vaping: unadjusted, basic & full adjustment results. Figure S8. Causal pathways blocked under differing levels of adjustment. [file 12916_2022_2511_MOESM1_ESM.pdf]

The UK Coronavirus Job Retention Scheme and smoking, alcohol consumption and vaping during the COVID-19 pandemic: Evidence from eight longitudinal population surveys

List of additional tables:

Table S1: Description of Studies

Table S2: Ethics and data access statements for each study

Table S3: Sample characteristics by study

Table S4: Employment status change by sex, education, and age-group

Table S5: Meta-analysed risk ratios and heterogeneity estimates for associations between changes in employment status and drinking behaviour: unadjusted, basic & full adjustment results

Table S6: Meta-analysed risk ratios and heterogeneity estimates for associations between changes in employment status and smoking: unadjusted, basic & full adjustment results

Table S7: Meta-analysed risk ratios and heterogeneity estimates for associations between changes in employment status and vaping: unadjusted, basic & full adjustment results

Figure S8: Causal pathways blocked under differing levels of adjustment

Table S1: Description of Studies

Study Population	Design, Sample Frame and Weighting	2020 Age range (years)	Most recent pre-pandemic survey	Details of 2020 COVID surveys (response rate)	Analytical N
Age Homogenous Cohorts					
MCS: Millennium Cohort Study	A nationally representative cohort of UK children born between Sept 2000 and Jan 2002 with regular follow-up surveys from birth. Weighted for sampling design, pre-pandemic attrition and non-response to COVID surveys.	18-20	2018-2019	May (26.6%)	2057
ALSPAC (G1): Avon Longitudinal Study of Parents and Children- Generation 1 (original young people)	Cohort of children born in the South-West of England between April 1991 and Dec 1992, with regular follow-up surveys from birth. Weighted for pre-pandemic attrition and non-response to COVID surveys.	27-29	2017-2018	June (17.4%)	1275
NS: Next Steps, formerly known as Longitudinal Study of Young People in England	A nationally representative sample recruited via secondary schools in England at around age 13 with regular follow-up surveys thereafter. Weighted for sampling design, pre-pandemic attrition and non-response to COVID surveys.	29-31	2015	May (20.3%)	1579
BCS70: British Cohort Study 1970	A nationally representative cohort of all children born in Great Britain (i.e. England, Wales & Scotland) in one week in 1970, with regular follow-up surveys from birth. Weighted for pre-pandemic attrition and non-response to COVID surveys.	50	2016	May (40.4%)	3151
NCDS: National Child Development Study	A nationally representative cohort of all children born in Great Britain (i.e. England, Wales & Scotland) in one week in 1958, with regular follow-up surveys from birth. Weighted for pre-pandemic attrition and non-response to COVID surveys.	62	2013	May (57.9%)	4358
Age Heterogeneous Studies					
USOC: Understanding Society: the UK Household Longitudinal Survey	A nationally representative longitudinal household panel study, based on a clustered-stratified probability sample of UK households, with all adults aged 16+ in chosen households surveyed annually. Weighted for sampling design, pre-pandemic attrition, non-response to COVID surveys, and outcome non-response within COVID surveys.	17-66	2018-2019	April (40.3%)	8328
ELSA: English Longitudinal Study of Aging	A nationally representative population study of individuals aged 50+ living in England, with biennial surveys and periodic refreshing of the sample to maintain representativeness. Weighted for sampling design, pre-pandemic attrition, and non-response to COVID surveys.	52-66	2018-2019	Jun-July (75%)	2417
GS: Generation Scotland: the Scottish Family Health Study	A family-structured, population-based Scottish cohort, with participants aged 18-99 recruited between 2006-2011. No weights were available.	27-66	2006-2011	April-Jun (21.6%)	2604
ALSPAC(G0): Avon Longitudinal Study of Parents and Children- Generation 0 (original parents)	Parents of the ALSPAC(G1) cohort described above, treated as a separate age-heterogenous study population.	44-66	2011-2013	June (12.2%)	2072

Table S2: Ethics and data access statements for each study

The most recent sweeps of the **NCDS, BCS70**, **Next Steps** and **MCS** have all been granted ethical approval by the National Health Service (NHS) Research Ethics Committee and all participants have given informed consent. Data for NCDS (SN 6137), BCS70 (SN 8547), Next Steps (SN 5545), MCS (SN 8682) and all four COVID-19 surveys (SN 8658) are available through the UK Data Service. NSHD data are available on request to the NSHD Data Sharing Committee. Interested researchers can apply to access the NSHD data via a standard application procedure. Data requests should be submitted to mrclha.swiftinfo@ucl.ac.uk; further details can be found at http://www.nshd.mrc.ac.uk/data.aspx. doi:10.5522/NSHD/Q101; doi:10.5522/NSHD/Q10.
Ethical approval was obtained from the **ALSPAC** Ethics and Law Committee and the Local Research Ethics Committees. The study website contains details of all the data that is available through a fully searchable data dictionary and variable search tool: http://www.bristol.ac.uk/alspac/researchers/our-data. ALSPAC data is available to researchers through an online proposal system. Information regarding access can be found on the ALSPAC website (http://www.bristol.ac.uk/media-library/sites/alspac/documents/researchers/data-access/ALSPAC_Access_Policy.pdf).
All waves of **TwinsUK** have received ethical approval associated with TwinsUK Biobank (19/NW/0187), TwinsUK (EC04/015) or Healthy Ageing Twin Study (H.A.T.S) (07/H0802/84) studies from NHS Research Ethics Committees at the Department of Twin Research and Genetic Epidemiology, King's College London. The TwinsUK Resource Executive Committee (TREC) oversees management, data sharing and collaborations involving the TwinsUK registry (for further details see https://twinsuk.ac.uk/resources-for-researchers/access-our-data/).
The University of Essex Ethics Committee has approved all data collection for the **Understanding Society** main study and COVID-19 waves. No additional ethical approval was necessary for this secondary data analysis. All data are available through the UK Data Service (SN 6614 and SN 8644).
Waves 1-9 of **ELSA** were approved through the National Research Ethics Service, while the COVID-19 Sub-study was approved by the UCL Research Ethics Committee. All participants provided informed consent. All data are available through the UK Data Service (SN 8688 and 5050).
**Generation Scotland** obtained ethical approval from the East of Scotland Committee on Medical Research Ethics (on behalf of the National Health Service). Reference number 20/ES/0021. Access to data is approved by the Generation Scotland Access Committee. See https://www.ed.ac.uk/generation-scotland/for-researchers/access or email access@generationscotland.org for further details.

Table S3: Sample Characteristics by Study

		MCS	NS	BCS	NCDS	GS	USOC	ELSA	ALSPAC-G0	ALSPAC-G1
	Total N	2057	1579	3151	4358	2604	8,328	2,417	2072	1275
	Age/Age range	18-20	29-31	50	62	27-66	17-66	52-66	50-65	27-29
		%	%	%	%	%	%	%	%	%
Sex	Male	49.6 (46.3-52.9)	44.1 (40.0-48.4)	48.0 (46.3-49.8)	49.4 (47.9-50.9)	33.5 (31.7-35.3)	47.9 (46.5-49.2)	48.0 (45.6-50.5)	22.8 (20.5-25.4)	29.0 (25.6-32.7)
	Female	50.4 (47.1-53.7)	55.9 (51.6-60.0)	52.0 (50.2-53.7)	50.6 (49.1-52.1)	66.5 (64.7-68.3)	52.1 (50.8-53.5)	52.0 (49.5-54.4)	77.2 (74.6-79.5)	71.0 (67.3-74.4)
Ethnicity	White	88.3 (84.9-91.1)	87.6 (85.0-89.8)	NA	NA	99.2 (98.8-99.5)	89.6 (88.2-90.8)	90.2 (88.2-91.8)	98.8 (98.0-99.2)	96.5 (94.3-97.9)
	Non-White Ethnic Minority	11.7 (8.9-15.1)	12.4 (10.2-15.0)	NA	NA	0.8 (0.5-1.2)	10.4 (9.2-11.8)	9.8 (8.2-11.8)	1.2 (0.7-2.0)	3.5 (2.1-5.8)
Education	Degree	39.4 (34.9-44.0)	45.3 (41.3-49.3)	39.7 (38.0-41.4)	35.4 (33.9-36.8)	52.1 (50.2-54.0)	39.6 (38.1-41.0)	25.0 (23.0-27.1)	20.8 (19.0-22.8)	30.6 (27.1-34.3)
	Less than degree	60.6 (56.0-65.1)	54.7 (50.7-58.7)	60.3 (58.6-62.0)	64.6 (63.2-66.1)	47.9 (46.0-49.8)	60.4 (59.0-61.9)	75.0 (73.0-77.0)	79.2 (77.2-81.0)	69.4 (65.7-72.9)
Household composition	Single, no children/ Alone	2.3 (1.2-4.2)	10.9 (8.8-13.4)	14.2 (13.0-15.4)	24.0 (22.7-25.3)	13.0 (11.8-14.4)	39.9 (38.3-41.6)	18.3 (16.3-20.4)	6.8 (5.4-8.6)	8.3 (6.3-10.9)
	Couple, no children/ Living only with partner	2.5 (1.8-3.6)	31.6 (28.0=35.4)	18.2 (16.9-19.6)	46.9 (45.4-48.4)	39.4 (37.6-41.3)	30.9 (29.7-32.2)	38.4 (36.2-40.8)	33.3 (30.8-36.0)	46.7 (42.8-50.7)
	Single, with children	NA	NA	NA	NA	4.8 (4.0-5.6)	4.0 (3.4-4.8)	8.7 (7.3-10.5)	5.3 (4.0-6.8)	1.1 (0.4-2.6)
	Couple, with children	4.0 (2.9-5.5)	34.6 (30.7-38.8)	55.4 (53.7-57.2)	22.5 (21.3-23.8)	35.4 (33.6-37.3)	25.1 (23.7-26.5)	30.8 (28.6-33.2)	20.8 (18.7-23.1)	11.6 (9.2-14.4)
	Others	91.2 (89.0-93.1)	23.0 (19.6-26.7)	12.2 (11.1-13.4)	6.6 (5.9-7.4)	7.4 (6.4-8.4)	NA	3.7 (3.0-4.7)	33.7 (31.1-36.5)	34.3 (30.7-37.2)
UK Nation	England	83.6 (80.8-86.0)	96.7 (94.7-97.9)	86.4 (85.2-87.6)	85.2 (84.1-86.2)	0.5 (0.3-0.9)	86.5 (85.4-87.6)	100.0	NA	NA
	Wales	5.5 (4.3-7.0)	1.3 (0.3-3.6)	5.2 (4.5-6.0)	4.4 (3.8-5.0)	NA	4.0 (3.5-4.5)	NA	NA	NA
	Scotland	8.3 (6.7-10.2)	0.7 (0.2-1.6)	7.4 (6.6-8.4)	8.3 (7.5-9.2)	99.4 (99.1-99.7)	7.3 (6.4-8.3)	NA	NA	NA
	Northern Ireland	2.0 (1.4-2.7)	0.1 (0.0-0.4)	0.2 (0.0-0.4)	0.3 (0.2-0.6)	NA	2.2 (1.9-2.5)	NA	NA	NA
	Other (Channel Islands / Isle of Man	0.7 (0.1-2.4)	1.2 (0.8-1.9)	0.8 (0.5-1.2)	1.7 (1.4-2.2)	0.1 (0.0-0.3)	NA	NA	NA	NA
	Pre-Pandemic Mental Health - High symptoms	18.2 (15.6-21.2)	25.4 (21.9-29.1)	19.0 (17.7-20.5)	14.6 (13.6-15.7)	12.7 (11.5-14.1)	22.3 (21.0-23.6)	13.7 (12.0-15.6)	18.9 (16.7-21.4)	22.9 (19.7-26.5)
	Pre-Pandemic Self-Rated Health Fair-Poor	14.1 (11.7-16.8)	11.9 (9.2-15.1)	14.8 (13.6-16.1)	18.4 (17.3-19.6)	NA	19.7 (18.4-21.1)	22.9 (20.7-25.3)	NA	NA

Percentages are weighted (except GS). Note: Analysis for GS, USOC, and ELSA restricted to participants aged 66 and younger. NA= Not Available

Table S4: Employment status change by sex, education, and age-group

	MCS	NS	BCS	NCDS	GS	USOC	ELSA	ALSPAC-G0	ALSPAC-G1
Total N	2057	1579	3151	4358	2604	8,328	2,417	2072	1275
Age/Age range	18-20	29-31	50	62	27-66	17-66	52-66	50-66	27-29
	%	%	%	%	%	%	%	%	%
**Stable Employed**	13.4 (11.1-16.1)	59.6 (55.3-63.8)	61.5 (59.8-63.2)	32.8 (31.4-34.2)	62.7 (60.8-64.5)	59.0 (57.4-60.5)	51.1 (48.6-53.6)	58.1 (55.3-60.9)	72.3 (68.5-75.7)
Male	13.6 (10.6-17.4)	68.8 (62.2-74.7)	65.3 (62.7-67.9)	35.2 (33.1-37.3)	65.6 (62.4-68.7)	60.4 (58.0-62.8)	55.3 (51.4-59.1)	57.8 (51.5-63.8)	76.2 (69.9-81.6)
Female	13.1 (9.9-17.0)	52.3 (47.2-57.4)	58.0 (55.7-60.2)	30.5 (28.7-32.4)	61.2 (58.9-63.5)	57.6 (55.7-59.5)	47.2 (44.1-50.3)	58.2 (55.0-61.3)	70.6 (66.1-74.8)
Degree	9.4 (6.1-14.2)	69.8 (64.3-74.7)	70.0 (67.7-72.2)	34.2 (32.2-36.3)	67.4 (64.8-69.8)	70.6 (68.8-72.4)	54.9 (50.5-59.3)	54.4 (49.7-59.0)	77.0 (70.7-82.2)
No Degree	16.0 (13.3-19.2)	51.2 (45.3-57.0)	55.9 (53.4-58.3)	32.1 (30.2-34.0)	57.6 (54.8-60.3)	51.3 (49.1-53.5)	49.8 (46.9-52.8)	59.1 (55.7-62.3)	70.0 (65.3-74.3)
Age 16-29	13.4 (11.1-16.1)				73.0 (57.0-84.6)	49.2 (45.2-53.1)			72.3 (68.5-75.7)
Age 30-49		59.6 (55.3-63.8)			78.2 (75.2-80.9)	69.3 (66.9-71.6)			
Age 50-66			61.5 (59.8-63.2)	32.8 (31.4-34.2)	55.4 (53.1-57.7)	54.9 (53.0-56.7)	51.1 (48.6-53.6)	58.1 (55.3-60.9)	
**Furloughed**	16.6 (13.8-19.7)	25.6 (22.2-29.5)	24.8 (23.3-26.4)	21.6 (20.4-22.9)	8.4 (7.4-9.5)	14.9 (13.8-16.1)	13.4 (11.9-15.2)	12.8 (10.9-15.0)	16.1 (13.3-19.3)
Male	12.9 (9.2-17.8)	23.0 (17.7-29.3)	24.8 (22.5-27.2)	25.6 (23.7-27.5)	10.1 (8.3-12.3)	15.8 (14.1-17.7)	12.8 (10.4-15.5)	13.5 (9.4-19.0)	13.3 (9.2-19.1)
Female	19.8 (16.4-23.6)	27.7 (23.1-32.8)	24.9 (23.0-26.9)	13.8 (12.4-19.4)	7.6 (5.4-8.9)	14.1 (12.9-15.5)	14.1 (12.1-16.4)	12.6 (10.5-15.1)	17.1 (13.8-21.2)
Degree	10.7 (8.1-13.9)	19.0 (14.8-24.1)	20.4 (18.5-22.5)	13.8 (12.4-15.4)	5.1 (4.0-6.4)	10.6 (9.4-11.9)	11.5 (9.0-14.6)	7.8 (5.6-10.9)	14.3 (9.9-20.3)
No Degree	20.4 (16.6-24.7)	31.1 (25.9-36.8)	27.7 (25.6-30.0)	25.9 (24.2-27.8)	12.0 (10.3-14.0)	17.8 (16.2-19.5)	14.1 (12.2-16.2)	14.1 (11.8-16.8)	16.9 (13.6-20.9)
Age 16-29	16.6 (13.8-19.7)				2.7 (0.5-13.8)	17.9 (15.2-20.8)			16.1 (13.3-19.3)
Age 30-49		25.6 (22.2-29.5)			10.5 (8.5-12.8)	16.0 (14.2-17.9)			
Age 50-66			24.8 (23.3-26.4)	21.6 (20.4-22.9)	7.6 (6.4-8.9)	11.8 (10.6-13.0)	13.4 (11.9-15.2)	12.8 (10.9-15.0)	

	MCS	NS	BCS	NCDS	GS	USOC	ELSA	ALSPAC-G0	ALSPAC-G1
**No Longer Employed**	3.6 (2.6-5.0)	2.6 (1.7-3.9)	1.9 (1.5-2.5)	2.8 (2.4-3.4)	3.3 (2.7-4.1)	3.5 (2.8-4.2)	2.0 (1.4-2.7)	10.3 (8.8-12.1)	5.4 (3.8-7.5)
Male	4.3 (2.6-6.9)	1.5 (0.8-2.8)	2.0 (1.3-2.9)	3.0 (2.3-3.8)	3.4 (2.4-4.9)	3.2 (2.3-4.4)	2.2 (1.3-3.6)	10.6 (7.6-14.6)	5.3 (2.9-9.3)
Female	2.9 (2.0-4.0)	3.4 (2.0-5.6)	1.9 (1.4-2.6)	2.7 (2.1-3.4)	3.3 (2.5-4.2)	3.7 (2.9-4.8)	1.8 (1.2-2.6)	10.3 (8.6-12.2)	5.4 (3.6-8.1)
Degree	4.5 (3.1-6.6)	3.2 (1.7-5.7)	1.8 (1.3-2.6)	2.8 (2.1-3.6)	3.3 (2.5-4.4)	3.0 (2.4-3.8)	1.5 (0.9-2.6)	12.7 (10.1-16.1)	4.9 (2.9-8.3)
No Degree	3.0 (1.8-5.0)	2.1 (128-3.6)	2.0 (1.4-2.8)	2.8 (2.2-3.6)	3.4 (2.5-4.5)	3.8 (2.8-5.0)	2.1 (1.5-3.1)	9.7 (8.0-11.7)	5.6 (3.6-8.5)
Age 16-29	3.6 (2.6-5.0)					5.6 (3.9-7.9)			5.4 (3.8-7.5)
Age 30-49		2.6 (1.7-3.9)	1.9 (1.5-2.5)	2.8 (2.4-3.4)	2.7 (1.8-4.1)	2.6 (1.7-4.0)			
Age 50-66					3.7 (2.9-4.7)	2.9 (2.4-3.5)	2.0 (1.4-2.7)	10.3 (8.8-12.1)	
**Stable Unemployed**	5.8 (3.7-8.8)	1.8 (1.0-3.1)	0.9 (0.6-1.3)	2.1 (1.8-2.6)	0.5 (0.3-0.8)	2.4 (1.9-3.0)	3.1 (2.2-4.4)	5.1 (4.1-6.5)	2.9 (2.2-3.8)
Male	8.4 (4.6-14.6)	1.4 (0.5-3.2)	1.0 (0.6-1.8)	2.7 (2.0-3.5)	0.8 (0.4-1.6)	2.6 (1.8-3.6)	4.0 (2.5-6.3)	7.3 (4.5-11.6)	2.4 (1.2-4.9)
Female	3.3 (2.1-5.0)	2.2 (1.0-4.4)	0.7 (0.4-1.2)	1.6 (1.1-2.2)	0.3 (0.2-0.8)	2.2 (1.7-3.0)	2.4 (1.5-3.9)	4.5 (3.5-5.8)	1.8 (1.0-3.2)
Degree	2.2 (1.1-4.2)	0.8 (0.3-2.1)	0.7 (0.4-1.2)	1.6 (1.2-2.3)	0.3 (0.1-0.8)	0.7 (0.5-1.2)	2.5 (1.1-5.3)	9.2 (6.9-12.1)	1.1 (0.4-3.1)
No Degree	8.1 (5.0-12.8)	2.6 (1.3-5.0)	1.0 (0.6-1.7)	2.4 (1.9-3.1)	0.7 (0.4-1.4)	3.5 (2.7-4.4)	3.4 (2.3-4.9)	4.1 (2.9-5.6)	2.4 (1.5-3.9)
Age 16-29	5.8 (3.7-8.8)					3.6 (2.4-5.5)			2.0 (1.3-3.1)
Age 30-49		1.8 (1.0-3.1)			0.1 (0.0-0.7)	1.7 (1.2-2.6)			
Age 50-66			0.9 (0.6-1.3)	2.1 (1.8-2.6)	0.7 (0.4-1.2)	2.3 (1.7-3.1)	3.1 (2.2-4.4)	5.1 (4.1-6.5)	

	MCS	NS	BCS	NCDS	GS	USOC	ELSA	ALSPAC-G0	ALSPAC-G1
**Became Employed**	1.1 (0.7-1.8)	0.9 (0.4-1.6)	0.5 (0.3-0.9)	0.6 (0.4-0.9)	0.6 (0.3-0.9)	1.1 (0.8-1.4)	0.4 (0.2-0.7)	0.1 (0.0-0.4)	1.1 (0.6-2.0)
Male	1.3 (0.6-2.8)	1.0 (0.4-2.3)	0.2 (0.03-0.7)	0.8 (0.4-1.3)	0.2 (0.1-0.8)	1.1 (0.7-1.8)	0.2 (0.0-0.6)	NA	1.6 (0.6-4.3)
Female	1.1 (0.6-2.1)	0.7 (0.2-1.8)	0.8 (0.5-1.3)	0.4 (0.2-0.8)	0.8 (0.4-1.3)	1.0 (0.7-1.4)	0.5 (0.2-1.2)	0.1 (0.0-0.5)	0.9 (0.4-1.9)
Degree	1.1 (0.5-1.3)	0.7 (0.3-1.4)	0.6 (0.3-1.1)	0.8 (0.5-1.3)	0.5 (0.3-1.1)	1.3 (0.5-1.2)	0.5 (0.2-1.7)	NA	2.2 (1.0-4.5)
No Degree	1.0 (0.5-2.4)	1.0 (0.3-2.3)	0.5 (0.2-1.0)	0.5 (0.3-0.9)	0.6 (0.3-1.3)	0.9 (0.6-1.4)	0.3 (0.1-0.7)	0.1 (0.0-0.5)	0.6 (0.2-1.8)
Age 16-29	1.1 (0.7-1.8)				2.7 (0.5-13.8)	1.5 (0.9-2.5)			1.1 (0.6-2.0)
Age 30-49		0.9 (0.4-1.6)			2.5 (0.1-0.9)	0.9 (0.6-1.5)			
Age 50-66			0.5 (0.3-0.9)	0.6 (0.4-0.9)	0.7 (0.4-1.2)	0.9 (0.6-1.3)	0.4 (0.2-0.7)	0.1 (0.0-0.4)	
**Stable Non-Employed**	59.6 (54.5-64.4)	9.5 (7.3-12.3)	10.4 (9.3-11.5)	40.0 (38.5-41.4)	24.5 (22.9-26.2)	19.2 (18.0-20.4)	30.0 (27.9-32.2)	13.5 (11.7-15.6)	3.3 (2.0-5.1)
Male	59.4 (52.5-66.0)	4.2 (2.2-7.9)	6.7 (5.4-8.2)	32.8 (30.8-34.9)	19.8 (17.3-22.6)	16.8 (15.0-18.8)	25.6 (22.5-29.1)	10.8 (7.6-15.2)	1.1 (0.5-2.4)
Female	59.9 (55.6-64.0)	13.7 (10.3-18.0)	13.8 (12.3-15.4)	46.9 (44.9-49.0)	26.8 (24.8-29.0)	21.3 (19.8-22.9)	34.0 (31.2-36.9)	14.3 (12.2-16.7)	4.1 (2.5-6.7)
Degree	72.0 (66.6-76.9)	6.5 (4.3-9.7)	6.5 (5.4-7.8)	46.8 (44.6-49.0)	23.4 (21.3-25.8)	13.7 (12.5-15.0)	29.1 (25.4-33.1)	15.7 (12.4-19.7)	0.5 (0.2-1.3)
No Degree	51.5 (45.7-57.2)	12.0 (8.6-16.5)	12.9 (11.4-14.7)	36.2 (34.3-38.2)	25.6 (23.3-28.2)	22.7 (21.0-24.6)	30.3 (27.8-33.0)	12.9 (10.8-15.4)	4.4 (2.7-7.1)
Age 16-29	59.6 (54.5-64.4)				21.6 (11.4-37.2)	22.2 (19.0-25.9)			3.3 (2.0-5.1)
Age 30-49		9.5 (7.3-12.3)			8.2 (6.5-10.3)	9.4 (8.0-11.0)			
Age 50+-66			10.4 (9.3-11.5)	40.0 (38.5-41.4)	32.0 (29.8-34.2)	27.3 (25.6-29.0)	30.0 (27.9-32.2)	13.5 (11.7-15.6)	

Percentages are weighted (except GS). Note: Analysis for GS, USOC, and ELSA restricted to participants aged 66 and younger.

Table S5: Meta-analysed risk ratios and heterogeneity estimates for associations between changes in employment status and drinking behaviour: unadjusted, basic & full adjustment results

Ref Cat: ***Stable Employed***	**Currently drinks 4+days/week or 5+ drinks/occasion**			**Reduced Alcohol Consumption**			**Increased Alcohol Consumption**		
	**Unadjusted**	**Basic**	**Full**	**Unadjusted**	**Basic**	**Full**	**Unadjusted**	**Basic**	**Full**
**Furloughed**	1.05 [0.95-1.16]	1.07 [0.96-1.19]	1.03 [0.94-1.13]	1.00 [0.89-1.12]	0.97 [0.85-1.11]	0.97 [0.85-1.11]	1.09 [0.97-1.22]	1.10 [0.97-1.25]	1.11 [0.98-1.26]
I^2 %	40.1	49.3	47.9	27.8	44.7	43.7	60.4	68.4	71.2
**No longer employed**	1.21 [1.08-1.36]	1.18 [1.05-1.32]	1.03 [0.89-1.20]	1.18 [1.02-1.37]	1.13 [0.97-1.31]	1.11 [0.95-1.28]	0.94 [0.82-1.08]	0.97 [0.86-1.10]	0.99 [0.87-1.11]
I^2 %	1.00	0.0	34.2	0.00	0.0	0.0	10.3	0.0	0.0
**Stable Unemployed**	1.34 [1.17-1.52]	1.28 [1.12-1.46]	1.09 [0.96-1.24]	1.10 [0.82-1.48]	0.97 [0.75-1.25]	0.96 [0.75-1.22]	0.61 [0.43-0.86]	0.70 [0.53-0.94]	0.68 [0.50-0.91]
I^2 %	0.00	0.00	0.00	32.3	17.0	10.8	43.3	27.1	27.8

Notes: 'Basic' adjustment includes age, sex, ethnicity, education, UK nation, and household composition. 'Full' adjustment additionally includes pre-pandemic measures of mental health, self-rated health, smoking, vaping and drinking. Pre-pandemic drinking was not included in models of drinking change (Reduced Alcohol Consumption or Increased Alcohol Consumption).

Table S6: Meta-analysed risk ratios and heterogeneity estimates for associations between changes in employment status and smoking: unadjusted, basic & full adjustment results

Ref Cat: Stable Employed	**Current Smoker**			**Smoking Less**			**Smoking More**		
	**Unadjusted**	**Basic**	**Full**	**Unadjusted**	**Basic**	**Full**	**Unadjusted**	**Basic**	**Full**
**Furloughed**	1.24 [1.08-1.42]	1.11 [0.97-1.27]	1.05 [0.95-1.16]	1.53 [1.00-2.36]	1.44 [0.90-2.31]	1.43 [0.91-2.25]	1.58 [1.12-2.22]	1.38 [0.94-2.03]	1.31 [0.88-1.96]
I^2 %	0.0	0.0	9.9	54.4	62.5	63.1	50.9	62.8	65.3
**No longer employed**	1.26 [0.81-1.95]	1.25 [0.78-2.01]	1.02 [0.66-1.56]	3.60 [2.17-5.95]	3.35 [1.80-6.22]	2.79 [1.44-5.39]	1.52 [0.90-2.56]	1.49 [0.88-2.55]	1.72 [0.67-4.45]
I^2 %	63.1	68.8	70.6	5.3	40.2	50.0	0.0	0.0	47.7
**Stable Unemployed**	2.50 [1.95-3.21]	1.91 [1.42-2.56]	1.10 [0.93-1.29]	2.38 [1.50-3.79]	1.86 [1.16-2.97]	1.74 [1.06-2.86]	2.10 [0.74-5.96]	1.38 [0.60-3.15]	1.20 [0.50-2.91]
I^2 %	3.5	31.1	0.0	0.0	0.0	0.0	61.6	43.8	46.6

Notes: 'Basic' adjustment includes age, sex, ethnicity, education, UK nation, and household composition. 'Full' adjustment additionally includes pre-pandemic measures of mental health, self-rated health, smoking, vaping and drinking. Pre-pandemic smoking was not included in models of smoking change (Smoking Less or Smoking More).

Table S7: Meta-analysed risk ratios and heterogeneity estimates for associations between changes in employment status and vaping: unadjusted, basic & full adjustment results

Ref Cat: Stable Employed	**Current Vaper**			**Vaping Less**			**Vaping More**		
	**Unadjusted**	**Basic**	**Full**	**Unadjusted**	**Basic**	**Full**	**Unadjusted**	**Basic**	**Full**
**Furloughed**	1.02 [0.84-1.25]	0.89 [0.73-1.09]	0.89 [0.74-1.08]	1.51 [0.99-2.30]	1.32 [0.87-2.01]	1.42 [0.94-2.14]	1.70 [1.26-2.31]	1.54 [1.13-2.09]	1.53 [1.14-2.07]
I^2 %	0.0	0.0	0.0	0.0	0.0	0.0	0.0	0.0	0.0
**No longer employed**	1.95 [1.27-3.00]	2.01 [1.22-3.32]	1.51 [0.92-2.50]	1.95 [0.86-4.44]	1.64 [0.70-3.85]	1.14 [0.42-3.09]	4.07 [1.88-8.81]	4.69 [2.34-9.39]	3.21 [1.51-6.80]
I^2 %	13.5	42.6	43.7	0.0	0.0	0.0	0.0	0.0	23.1
**Stable Unemployed**	1.23 [0.74-2.05]	1.17 [0.71-1.91]	0.98 [0.62-1.57]	2.27 [0.79-6.51]	1.76 [0.60-5.22]	1.64 [0.54-5.00]	2.17 [0.77-6.13]	2.14 [0.77-5.92]	1.97 [0.93-4.17]
I^2 %	0.0	0.0	0.0	NA	NA	NA	59.9	59.8	20.2

Notes: 'Basic' adjustment includes age, sex, ethnicity, education, UK nation, and household composition. 'Full' adjustment additionally includes pre-pandemic measures of mental health, self-rated health, smoking, vaping and drinking. Pre-pandemic vaping was not included in models of vaping change (Vaping Less or Vaping More). I2 values are not presented for estimates of the association between stable unemployment and vaping less as only one study (USOC) had sufficient data to provide estimates.

Figure S8: Causal pathways blocked under differing levels of adjustment

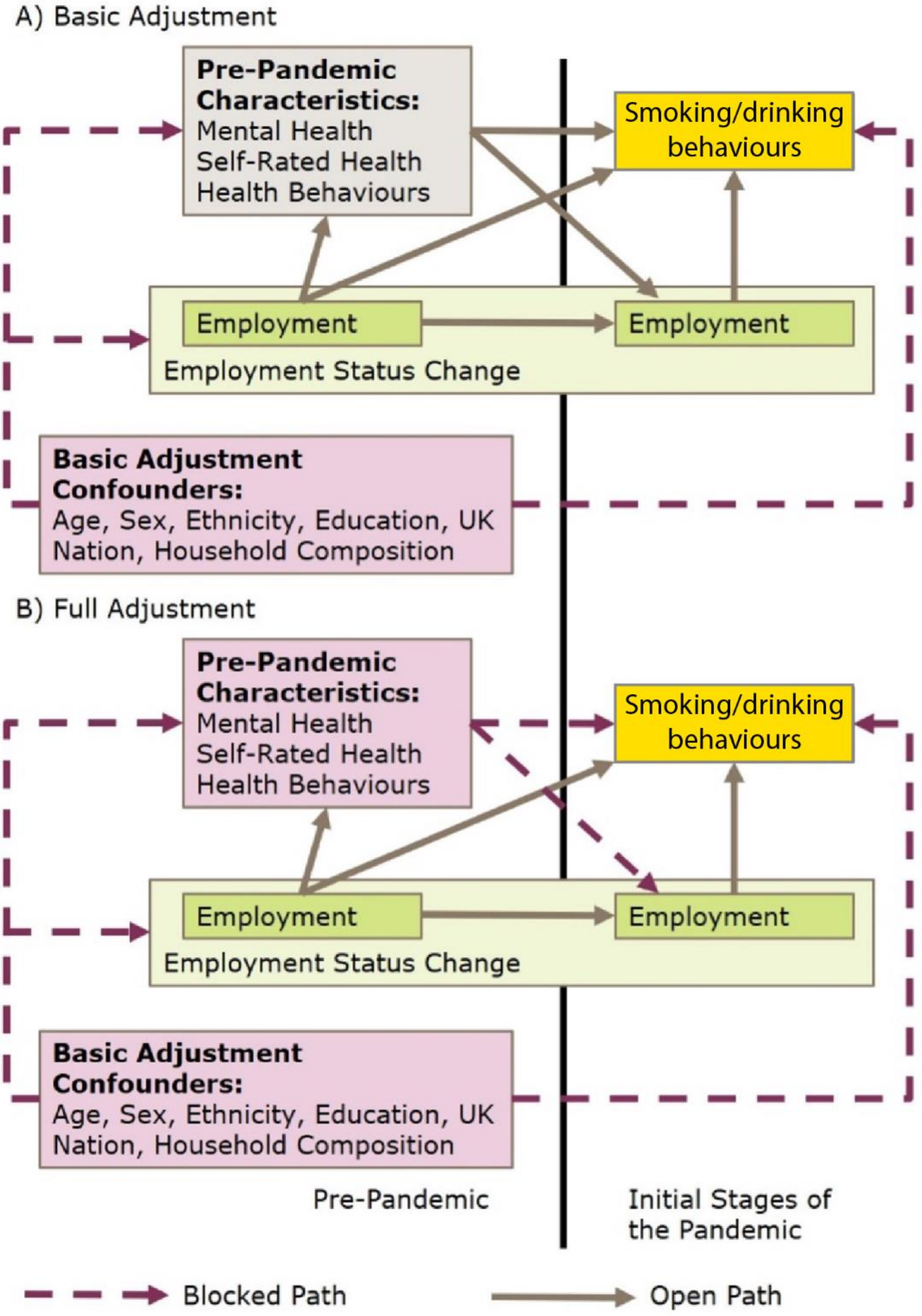

Without control for pre-pandemic characteristics, the basic adjusted risk ratios may represent both newly acquired behaviour and/or continuation of established (pre-pandemic) behaviour. The full adjustment risk ratios block effects via these pre-pandemic characteristics and are interpreted as representing differential change in health behaviour independent of these pre-pandemic characteristics. For the outcomes directly capturing changes in health behaviour, the full adjustment did not include pre-pandemic levels of the behaviour in question (as it was part of the outcome) and even full adjustment risk ratios may partially reflect associations with pre-pandemic behaviour
