# Supplementary material for: The UK Coronavirus Job Retention Scheme and smoking, alcohol consumption and vaping during the COVID-19 pandemic: evidence from eight longitudinal population surveys
Source: BMC Med. 2022 Sep 21;20:345. doi: 10.1186/s12916-022-02511-0 (PMC9489267; doi:10.1186/s12916-022-02511-0)
Supplement: Supplementary file 2 — Additional file 2. Variable Coding. [file 12916_2022_2511_MOESM2_ESM.pdf]

Study	Question (exact wording)	Possible Answers	Recoding
*** CHANGE in EMPLOYMENT STATUS ***			
	**1='Stable employed'; 2='Furloughed'; 3='Became employed'; 4='No longer employed'; 5='Stable unemployed'; 6='Stable in other category'**		
**MCS**<br>**NS**<br>**BCS 70**<br>**NCDS**	Q1: Which of these best describes what you were doing just before the Coronavirus outbreak in March? If you were doing more than one activity, please choose the activity that you spent most time doing.<br>Q2: Which of these would you say best describes your situation now?	Q1. 1=Employed; 2=Self-employed; 3=In unpaid/ voluntary work; 4=Apprenticeship; 5=Unemployed; 6=Permanently sick or disabled; 7=Looking after home or family; 8=In education at school/college/university; 9=Retired; 10=Doing something else.<br>Q2. 1=Employed and currently working (or on annual leave/holiday); 2=Employed but on paid leave (including furlough); 3=Employed and on unpaid leave; 4=Apprenticeship; 5=In unpaid/voluntary work; 6=Self-employed and currently working (or on holiday); 7=Self-employed but not currently working; 8=Unemployed; 9=Permanently sick or disabled; 10=Looking after home or family; 11=In education at school/college/university; 12=Retired; 13=Doing something else.<br>Q3. 1=There was no interruption to learning activities 2. I took a break from learning activities 3. I was studying at home with online resources provided by my learning establishment 4. I was studying at home with no online resources provided by my learning establishment 5. My course finished earlier than planned 6. I dropped out from learning activities	1= if (Q1 = 1) & (Q2 = 1 OR 6)<br>2= if (Q1 = 1) & (Q2 = 2 OR 7)<br>3= if (Q1 != 1 OR 5) & (Q2 = 1 OR 6)<br>4= if (Q1 = 1) & (Q2 != 1 OR 6)<br>5= if (Q1 = 5) & (Q2 = 8)<br>6= if (Q1 = != 1 OR 5) & (Q2 != 1 OR 2 OR 6 OR 7 OR 8)"
**ALSPAC**	Q1. Just before the lockdown on the 23rd March 2020, were you?<br>Q2. Which of these would you say best describes your current situation now?	Q1. 1=In full time paid work (30 or more hours a week); 2=In part-time paid work (less than 30 hours a week); 3=In irregular or occasional work; 4=Doing a modern apprenticeship or other government supported training/work-experience scheme; 5=Unemployed and looking for work; 6=Unable to work through sickness/disability; 7=In full-time education; 8=In part-time education; 9=Doing voluntary work; 10=Self-employed; 11=A full/part time carer; 12=Retired; 13=Other.<br>Q2. 1=Employed and working same number of hours as pre-lockdown; 2=Employed and working reduced number of hours; 3=Employed and working more hours than before; 4=Employed but on paid leave (including furlough); 5=Employed and on unpaid leave; 6=Apprenticeship; 7=In unpaid/voluntary work; 8=Self-employed and currently working; 9=self-employed but not currently working; 10=Unemployed; 11=Permanently Sick/Disabled; 12=looking after home or family;13=In education at school/college/university	1=if(Q1=1/3) & (Q2= 1/3 OR 8)<br>2=if(Q1=1/3) & (Q2=4)<br>3=if(Q1!=1/3) & (Q2 = 1/3 OR 8)<br>4=if(Q1=1/3) & (Q2 = 5/7 OR 9/13)<br>5=if(Q1=5) & (Q2 = 10)<br>6=if(Q1!=1/3) & (Q2 = 5/7 OR 9/13)
**USOC**	Q1: Thinking back to earlier this year, before the outbreak of the coronavirus pandemic. Were you in paid work or self-employment at any time in January or February 2020?<br>Q2: Thinking about your situation now. Even if you did not do any paid work last week, are you currently employed or self-employed?<br>Q3: Have you received a written letter or email from your employer to confirm that you have been furloughed under the Coronavirus Job Retention Scheme?<br>Q4: (asked pre-pandemic) Which of these best describes your current employment situation?	Q1: 1. Yes, employed; 2. Yes, self-employed; 3. Yes, both employed and self-employed; 4. No.<br>Q2: 1. Yes, employed; 2. Yes, self-employed; 3. Yes, both employed and self-employed; 4. No.<br>Q3: 1. Yes; 2. No.<br>Q4: 1. Self employed; 2. Paid employment; 3. Unemployed; 4. Retired; 5. On maternity leave; 6. Family care or home; 7. Full-time student; 8. LT sick or disabled; 9. Govt training scheme; 10. Unpaid, family business; 11. On apprenticeship. 97. Doing something else.	1=if (Q1<=3) & (Q2<=3)<br>2=if (Q1<=3) & Q3=1<br>3=if (Q1=4) & (Q2<=3)<br>4=if (Q1<=3) & (Q2=4)<br>5=if (Q1=4) & (Q2=4) & (Q4=3)<br>6=if (Q1=4) & (Q2=4) & (Q4!=3)
**ELSA**	Q1: Which of these best describes what you were doing just before the coronavirus outbreak?<br>Q2: And which of these would you say best describes your current situation?	Q1. 1=Retired; 2=Employed; 3=Self-employed; 4=Unemployed; 5=Permanently sick or disabled; 6=Looking after home or family<br>Q2. 1=Retired; 2=Employed; 3=Paid/unpaid leave from employment (including furlough); 4=Self-employed and currently working; 5=Self-employed but not currently working; 6=Unemployed; 7=Permanently sick or disabled; 8=Looking after home or family.	1= if (Q1=2 OR 3) & (Q2=2 OR 4)<br>2= if (Q1=1 OR 2 OR 3) & (Q2=3 OR 5)<br>3= if (Q1=1 OR >=4) & (Q2=2 OR =4)<br>4= if (Q1=2 OR 3) & (Q2>=6 OR Q2==1)<br>5= if Q1=4 & Q2=6<br>6= if (Q1=1 & Q2=1) OR (Q1>=4 & (Q2=1 OR 3 OR 5 OR 7 OR 8)) OR (Q1=1 & Q2>=7)
**GS**	Q1.What was your employment status just before the COVID-19 measures were introduced (i.e. January 2020)?<br>Q2. What is your employment status now?<br>Q3. Have any of the following happened to you due to COVID-19 measures?	Q1 and Q2. 1=Self-employed employing others; 2=Self-employed not employing others; 3=Paid employee supervising others; 4=Paid employee not supervising others; 5=In unpaid employment; 6=Homemaker; 7=Looking after children; 8=Looking after other dependents; 9=Retired; 10=Still in school/studying full-time; 11=Unemployed as sick or disabled; 12=Unemployed; 13=Other<br>Q3. 1=Yes; 0=NA	1= if Q1<=4 & Q2<=4<br>2= if Q3=1<br>3= if Q1>=5 & Q2<=4<br>4= if Q1<=4 & Q2 >= 5<br>5= if Q1=12 & Q2=12<br>6= if (Q1>=5 & Q1<=11|Q1==13) & ((Q2>=5 & Q2<=11)|Q2==13)

Study	Question (exact wording)	Possible Answers	Recoding
*** CURRENT SMOKER * -- 1= Currently smokes**			
**MCS**<br>**NS**<br>**BCS 70**<br>**NCDS**	Which of these statements applies to you?	1=I've never smoked cigarettes; 2=I used to smoke cigarettes but don't at all now; 3=I now smoke cigarettes occasionally but not every day; 4=I smoke cigarettes every day	=3 OR 4
**ALSPAC**	Not Available		
**USOC**	Do you smoke cigarettes? Please do not include electronic cigarettes (e-cigarettes).	1=Yes; 0=No	=1
**ELSA**	Do you currently smoke?	1=Yes; 0=No	=1
**GS**	Compared to before COVID-19 measures were introduced (i.e., January 2020) are you:	1=Smoking more now; 2=Smoking about the same now; 3=Smoking less now; 4=I don't smoke: ex-smoker; 5=I don't smoke: never-smoker	=1 OR 2 OR 3

*** SMOKING MORE (including relapse/initiation) * -- 1= Smokes more**		**u**	
**MCS**<br>**NS**<br>**BCS 70**<br>**NCDS**	Q1: In the month before the Coronavirus outbreak, how many cigarettes a day did you usually smoke?<br>Q2: Since the start of the Coronavirus outbreak, how many cigarettes a day have you typically smoked?	Q1 & Q2: 0-99	Q2>Q1
**ALSPAC**	Not Available		
**USOC**	Q1: Do you smoke cigarettes? Please do not include electronic cigarettes (e-cigarettes).<br>Q2: Approximately how many cigarettes a day do you usually smoke, including those you roll yourself?	Q1. 1=Yes; 2=No<br>Q2: 0 or more (0 indicates <1 per day)	Pre-Pandemic Q1=2 AND COVID Q1=1, OR both Q=1 AND Pre-Pandemic Q2<COVID Q2
**ELSA**	Q1. Do you currently smoke?<br>Q2. Since the start of the coronavirus outbreak, please say whether you have been smoking…<br>Q3 [Pre-Pandemic]. Do you smoke cigarettes at all nowadays?	Q1. 1=Yes; 2=No<br>Q2. 1=Less than usual; 2=About the same; 3=More than usual<br>Q3. 1=Yes; 2.No	Q2=3 OR (Q3=2 & Q1=1)
**GS**	Compared to before COVID-19 measures were introduced (i.e., January 2020) are you:	1=Smoking more now; 2=Smoking about the same now; 3=Smoking less now; 4=I don't smoke: ex-smoker; 5=I don't smoke: never-smoker	=1

*** CURRENT VAPER * -- 1= Currently vapes**			
**MCS**<br>**NS**<br>**BCS 70**<br>**NCDS**	Now thinking about electronic cigarettes or vaping devices, which of these statements applies to you?	1=I've never used an electronic cigarette or a vaping device;<br>2=I used to use an electronic cigarette or a vaping device but don't at all now;<br>3=I now use an electronic cigarette or a vaping device occasionally but not every day;<br>4=I use electronic cigarettes or vaping devices every day	=3 OR 4
**ALSPAC**	Not Available		
**USOC**	Do you ever use electronic cigarettes (e-cigarettes)?	1=I have never used e-cigarettes<br>2=I have only tried using e-cigarettes once or twice<br>3=I used e-cigarettes regularly in the past, but I never use them now<br>4=I sometimes use e-cigarettes but less than once a month<br>5=I use e-cigarettes at least once a month, but less than once a week<br>6=I use e-cigarettes at least once a week	=4 or more
**ELSA**	Not Available		
**GS**	Compared to before COVID-19 measures were introduced (i.e., January 2020), are you:	1=Vaping more now;<br>2=Vaping about the same now;<br>3=Vaping less now;<br>4=I don't vape	=1 OR 2 OR 3

Study	Question (exact wording)	Possible Answers	Recoding
*** VAPING MORE * -- 1= Vapes more**			
**MCS**<br>**NS**<br>**BCS 70**<br>**NCDS**	Since the start of the Coronavirus outbreak, has the amount you have been using an electronic cigarette or vaping device changed?	1= More frequent use of e-cig/vaping device<br>2= Less frequent use of e-cig/vaping device<br>3= No change (only asked if now use an electronic cigarette or a vaping device occasionally but not every day or use electronic cigarettes or vaping devices every day)	=1
**ALSPAC**	Not Available		
**USOC**	Do you ever use electronic cigarettes (e-cigarettes)?	1=I have never used e-cigarettes<br>2=I have only tried using e-cigarettes once or twice<br>3=I used e-cigarettes regularly in the past, but I never use them now<br>4=I sometimes use e-cigarettes but less than once a month<br>5=I use e-cigarettes at least once a month, but less than once a week<br>6=I use e-cigarettes at least once a week	(Pre-Pandemic=1-3 AND COVID=4-6)<br>OR (Pre-Pandemic=4-5 AND COVID=6)
**ELSA**	Not Available		
**GS**	Compared to before COVID-19 measures were introduced (i.e., January 2020), are you:	1=Vaping more now<br>2=Vaping about the same now<br>3=Vaping less now<br>4=I don't vape	=1

Study	Question (exact wording)	Possible Answers	Recoding
*** VAPING LESS * -- 1= Vapes less**			
**MCS**<br>**NS**<br>**BCS 70**<br>**NCDS**	Since the start of the Coronavirus outbreak, has the amount you have been using an electronic cigarette or vaping device changed?	1= More frequent use of e-cig/vaping device<br>2= Less frequent use of e-cig/vaping device<br>3= No change (only asked if now use an electronic cigarette or a vaping device occasionally but not every day or use electronic cigarettes or vaping devices every day)	=2
**ALSPAC**	Not Available		
**USOC**	Do you ever use electronic cigarettes (e-cigarettes)?	1=I have never used e-cigarettes<br>2=I have only tried using e-cigarettes once or twice<br>3=I used e-cigarettes regularly in the past, but I never use them now<br>4=I sometimes use e-cigarettes but less than once a month<br>5=I use e-cigarettes at least once a month, but less than once a week<br>6=I use e-cigarettes at least once a week	(Pre-Pandemic=1-3 & COVID=1-3)<br>OR (Pre-Pandemic=6 & COVID=4-5)
**ELSA**	Not Available		
**GS**	Compared to before COVID-19 measures were introduced (i.e., January 2020), are you:	1=Vaping more now<br>2=Vaping about the same now<br>3=Vaping less now<br>4=I don't vape	=1

Study	Question (exact wording)	Possible Answers	Recoding
*** DRINKING FREQUENCY * -- 1= Drinks 4+ days a week**			
**MCS**<br>**NS**<br>**BCS 70**<br>**NCDS**	Since the start of the Coronavirus outbreak, how often have you had a drink containing alcohol?	1=4 or more times a week;<br>2=2/3 times a week;<br>3=2/4 times per month;<br>4=Monthly or less;<br>5=Never	=1
**ALSPAC**	Not available		
**USOC**	Pre-Pandemic: Thinking about the past 12 months, how often do you have a drink containing alcohol?<br>COVID survey: Thinking about the last 4 weeks, how often did you have a drink containing alcohol?	Pre-Pandemic:<br>1=Never; 2=Monthly or less; 3=2-4 times per month; 4=2-3 times per week; 5=4+ times per week<br>COVID survey:<br>1=Never; 2=Once; 3=2-4 times in total; 4=2-3 times per week; 5=4-6 times per week; 6=Daily	In COVID survey =5 or more
**ELSA**	Not Available		
**GS**	Not Available		
*** DRINKING INCREASED FREQUENCY * -- 1= Drinks more frequently**			
**MCS**<br>**NS**<br>**BCS 70**<br>**NCDS**	Q1: In the months before the Coronavirus outbreak, how often have you had a drink containing alcohol?<br>Q2: Since the start of the Coronavirus outbreak, how often have you had a drink containing alcohol?	Q1 & Q2. 1=4 or more times a week;<br>2=2/3 times a week;<br>3=2/4 times per month;<br>4=Monthly or less;<br>5=Never	Q2>Q1
**ALSPAC**	Not Available		
**USOC**	Pre-Pandemic: Thinking about the past 12 months, how often do you have a drink containing alcohol?<br>COVID survey: Thinking about the last 4 weeks, how often did you have a drink containing alcohol?	Pre-Pandemic:<br>1=Never; 2=Monthly or less; 3=2-4 times per month; 4=2-3 times per week; 5=4+ times per week<br>COVID survey:<br>1=Never; 2=Once; 3=2-4 times in total; 4=2-3 times per week; 5=4-6 times per week; 6=Daily	If Pre-Pandemic answer < answer during COVID
**ELSA**	Not Available		
**GS**	Not Available		
*** DRINKING DECREASED FREQUENCY * -- 1= Drinks less frequently**			
**MCS**<br>**NS**<br>**BCS 70**<br>**NCDS**	Q1: In the months before the Coronavirus outbreak, how often have you had a drink containing alcohol?<br>Q2: Since the start of the Coronavirus outbreak, how often have you had a drink containing alcohol?	Q1 & Q2. 1=4 or more times a week;<br>2=2/3 times a week;<br>3=2/4 times per month;<br>4=Monthly or less;<br>5=Never	Q2<Q1
**ALSPAC**	Not Available		
**USOC**	Pre-Pandemic: Thinking about the past 12 months, how often do you have a drink containing alcohol?<br>COVID survey: Thinking about the last 4 weeks, how often did you have a drink containing alcohol?	Pre-Pandemic:<br>1=Never; 2=Monthly or less; 3=2-4 times per month; 4=2-3 times per week; 5=4+ times per week<br>COVID survey:<br>1=Never; 2=Once; 3=2-4 times in total; 4=2-3 times per week; 5=4-6 times per week; 6=Daily	If Pre-Pandemic answer > answer during COVID
**ELSA**	Not Available		
**GS**	Not Available		

Study	Question (exact wording)	Possible Answers	Recoding
*** DRINKING QUANTITY * -- 1= Drinks 5 or more alcohol units per occasion**			
**MCS**<br>**NS**<br>**BCS 70**<br>**NCDS**	Since the start of the Coronavirus outbreak, how many standard alcoholic drinks do you have on a typical day when you were drinking?	1=1/2; 2=3-4; 3=5/6; 4=7-9; 5=10 or more	=3 OR 4 OR 5
**ALSPAC**	Not Available		
**USOC**	How many drinks do you have on a typical day when you are drinking?	1=1-2 drinks; 2=3-4 drinks; 3=5-6 drinks; 4=7-9 drinks; 5=10+ drinks	=3 OR 4 OR 5
**ELSA**	Not Available		
**GS**	Not Available		

Study	Question (exact wording)	Possible Answers	Recoding
*** DRINKING INCREASED QUANTITY * -- 1= Drinks more alcohol units per occasion**			
**MCS**<br>**NS**<br>**BCS 70**<br>**NCDS**	Q1: In the months before the Coronavirus outbreak, how many standard alcoholic drinks do you have on a typical day when you were drinking?<br>Q2: Since the start of the Coronavirus outbreak, how many standard alcoholic drinks do you have on a typical day when you were drinking?	Q1 & Q2.<br>1=1/2;<br>2=3-4;<br>3=5/6;<br>4=7-9;<br>5=10 or more	Q2>Q1
**ALSPAC**	Since lockdown, have any of the following aspects of your life changed?: Amount of alcohol you drink:	1=Decreased a lot<br>2=Decreased a little<br>3=Stayed the same<br>4=Increased a little<br>5=Increased a lot<br>6=NA	4/5 == 1<br>Else ==0<br>[note: this variable is included in the outcome “Drinking more” and “Drinking less” only]
**USOC**	Both Pre-Pandemic and Covid Survey:<br>How many drinks do you have on a typical day when you are drinking?	1=1-2 drinks<br>2=3-4 drinks<br>3=5-6 drinks<br>4=7-9 drinks<br>5=10+ drinks	If Pre-Pandemic answer is < answer during COVID
**ELSA**	Not Available		
**GS**	Not Available		

Study	Question (exact wording)	Possible Answers	Recoding
*** DRINKING DECREASED QUANTITY * -- 1= = Drinks fewer alcohol units per occasion**			
**MCS**<br>**NS**<br>**BCS 70**<br>**NCDS**	Q1: In the months before the Coronavirus outbreak, how many standard alcoholic drinks do you have on a typical day when you were drinking?<br>Q2: Since the start of the Coronavirus outbreak, how many standard alcoholic drinks do you have on a typical day when you were drinking?	Q1 & Q2.<br>1=1/2;<br>2=3-4;<br>3=5/6;<br>4=7-9;<br>5=10 or more	Q2>Q1
**ALSPAC**	Since lockdown, have any of the following aspects of your life changed?: Amount of alcohol you drink:	1=Decreased a lot<br>2=Decreased a little<br>3=Stayed the same<br>4=Increased a little<br>5=Increased a lot<br>6=NA	1/2==1<br>Else==0<br>[note: this variable is included in the outcome “Drinking more” and “Drinking less” only]
**USOC**	Both Pre-Pandemic and Covid Survey:<br>How many drinks do you have on a typical day when you are drinking?	1=1-2 drinks<br>2=3-4 drinks<br>3=5-6 drinks<br>4=7-9 drinks	If Pre-Pandemic answer is < answer during COVID

	5=10+ drinks
**ELSA**	Not Available
**GS**	Not Available

Study	Possible Answers	Recoding
*** Sex * 0=Male; 1=Female**		
**All**	0=Male; 1=Female	
*** Ethnicity * 0=White; 1=Ethnic Minority**		
**MCS**	1=White; 2=Mixed; 3=Indian; 4=Pakistani; 5=Bangladeshi; 6=Other Asian; 7=Black Caribbean; 8=Black African; 9=Other Black; 10=Chinese; 11=Other ethnic group	1=0; 2/11=1
**NS**	1=White; 2=Mixed; 3=Indian; 4=Pakistani; 5=Bangladeshi; 6=Black Caribbean; 7=Black African; 8=Other	1=0; 2/8=1
**BCS70**	Not Available	
**NCDS**	Not Available	
**ALSPAC**	G0 (Parents) 1=White; 2=Black Caribbean; 3=Black African; 4=Other black; 5=Indian; 6=Pakistani; 7=Bangladeshi; 8=Chinese; 9=Other<br>G1 (Children) 1=White; 2=Mixed/Multiple Ethnic group; 3=Asian; 4=Black/African/Caribbean/Black British; 5=Arab or Other	1=0; 2/9=1
**USOC**	1=White British; 2=Irish (White); 3=Gypsy or Irish Traveller (white); 4=Any other white background; 5=White and black Caribbean (mixed); 6=White and black African (mixed); 7=White and Asian (mixed); 8=Any other mixed background; 9=Indian (Asian or Asian British); 10=Pakistani (Asian or Asian British); 11=Bangladeshi (Asian or Asian British); 12=Chinese (Asian or Asian British); 13=Any other Asian background (Asian or Asian British); 14=Caribbean (Black or Black British); 15=African (Black or Black British); 16=Any other Black background (Black or Black British); 17=Arab (other Ethnic group); 97=Any other ethnic group	1-4=0; 5-97=1
**ELSA**	1.White; 2=Mixed ethnic group; 3=Black; 4=Black British; 5=Asian; 6=Asian British	1=0; 2/6=1
**GS**	1=White Scottish; 2=White English; 3=White Welsh; 4=White N. Irish; 5=White Irish; 6=White Gypsy/Irish traveller; 7=White Polish; 8=Any other white; 9=Asian/British Asian - Indian; 10=Asian/British Asian - Pakistani; 11=Asian/British Asian - Bangladeshi; 12=Asian/British Asian - Chinese; 13=Any other Asian background; 14=Black or Black British - African; 15=Black or Black British - Caribbean; 16=Any other Black/African/Caribbean background; 17=Arab or Arab British; 18=Mixed - White and Black Caribbean; 19=Mixed - White and Black African; 20=Mixed - White and Asian; 21=Any other Mixed/Multiple ethnic background; 22=Any other ethnic group	1-8=0; 9-22=1
*** Education * 0=No Degree; 1=Degree**		
**MCS**<br>**NS**<br>**BCS 70**<br>**NCDS**	0=None; 1=Nvq1; 2=Nvq2; 3=Nvq3; 4=Nvq4; 5=Nvq5<br>*parent's education for MCS	1/4=0; 5=1
**ALSPAC**	1=Degree; 2=A levels/AS levels or equivalent; 3=O levels; 4=Vocational; 5=CSE<br>*parent's education for G1 (Children)	2/5=0
**USOC**	1.Higher degree 2. 1st degree or equivalent 3. Diploma in Higher Education 4. Teaching qualification (not PGCE) 5. Nursing or other medical qualification 6. Other higher degree 7. A-Level 8. Welsh baccalaureate 9. International baccalaureate 10. AS Level 11. Scottish Highers 12. Certificate of 6th year studies 13. GCSE/O-Level 14. Certificate of secondary education 15. Standard or lower 16. Other school certificate 96. No qualifications	1/6=1; 7-96=0
**ELSA**	1=Nvq4/nvq5/degree or equivalent; 2=Higher Education below degree; 3=Nvq3/GCE A level equivalent; 4=Nvq2/GCE O level equivalent; 5=Nvq1/CSE other grade equivalent; 6=Foreign/other; 7=No qualification	2/7=0
**GS**	1=No qualifications; 2=Other (please specify); 3=School leavers certificate; 4=CSEs or equivalent; 5=Standard grade, National 4 or 5, O levels, GCSEs or equivalent; 6=Higher grade, A levels, AS levels or equivalent; 7=NVQ or HND or HNC or equivalent; 8=Other professional or technical qualification; 9=Undergraduate degree; 10=Postgraduate degree	1/8=0; 9 OR 10=1
*** Living Arrangements * 1=Alone; 2=With partner/spouse only; 3=With partner/spouse and child(ren); 4=With child(ren), without partner/spouse; 5=Any other living arrangement**<br>**OR * Partnership Status * 1=Married/Partnered; 0=Not married/partnered**		
*** Pre-Pandemic Self-Assessed Health * 1=Good/Very Good/Excellent; 0=Fair/Poor**		
**MCS**<br>**NS**<br>**BCS 70**<br>**NCDS**	In general, in the 3 months before the Coronavirus outbreak would you say your health was … 1=Excellent; 2=Very Good; 3=Good; 4=Fair; 5=Poor	1/3=1; 4/5=0
**ALSPAC**	Derived: Prepandemic Asthma (0: No, 1: Yes) + Prepandemic Obesity (0: No, 1:Yes) + Prepandemic Diabetes (0: No, 1:Yes)	0=1; >1=0
**USOC**	In general, would you say your health is… 1=Excellent; 2=Very Good; 3=Good; 4=Fair; 5=Poor	1/3=1; 4/5=0
**ELSA**	Would you say your health is… 1=Excellent; 2=Very Good; 3=Good; 4=Fair; 5=Poor	1/3=1; 4/5=0
**GS**	Not available	

Study	Possible Answers	Recoding
*** Pre-Pandemic Mental Health * 1=High Psychological Distress; 0=No Psychological Distress**		
**MCS**	Kessler K6 measure of psychological distress [Kessler, R. C., Andrews, G., Colpe, L. J., Hiripi, E., Mroczek, D. K., Normand, S. L., ... & Zaslavsky, A. M. (2002). Short screening scales to monitor population prevalences and trends in non-specific psychological distress. Psychological medicine, 32(6), 959-976.]	=1 if 13+
**NS**	General Health Questionnaire GHQ-12 [Goldberg DP, Gater R, Sartorius N,et al. The validity of two versions of the GHQ in the WHO study of mental illness in general health care. Psychol Med 1997;27:191–7.]	=1 if 4+
**BCS70**	Malaise Inventory. [Rodgers, B., Pickles, A., Power, C., Collishaw, S., & Maughan, B. (1999). Validity of the Malaise Inventory in general population samples. Social psychiatry and psychiatric epidemiology, 34(6), 333-341.]	=1 if 4+
**NCDS**	Malaise Inventory. [Rodgers, B., Pickles, A., Power, C., Collishaw, S., & Maughan, B. (1999). Validity of the Malaise Inventory in general population samples. Social psychiatry and psychiatric epidemiology, 34(6), 333-341.]	=1 if 4+
**ALSPAC G1**	Short Mood and Feelings Questionnaire (SMFQ) [Angold, A., Costello, E.J., Messer, S.C., & Pickles, A. (1995). Development of a short questionnaire for use in epidemiological studies of depression in children and adolescents. International Journal of Methods in Psychiatric Research, 5, 237–249.]	=1 if 12+
**ALSPAC G0**	Edinburgh Postnatal Depression Scale EPDS [Matthey S, Barnett B, Kavanagh DJ, et al. Validation of the Edinburgh Postnatal Depression Scale for men, and comparison of item endorsement with their partners. Journal of Affective Disorders 2001;64(2-3):175-84.]	=1 if 11+
**USOC**	General Health Questionnaire GHQ-12 [Goldberg DP, Gater R, Sartorius N,et al. The validity of two versions of the GHQ in the WHO study of mental illness in general health care. Psychol Med 1997;27:191–7.]	=1 if 4+
**ELSA**	Eight-item version of the original CES-D (felt depressed; felt everything was an effort; restless sleep; not happy; felt lonely; not enjoyed life; felt sad; could not get going) [Radloff LS. The CES-D Scale: A Self-Report Depression Scale for Research in the General Population. Applied Psychological Measurement. 1977;1(3):385-401]	=1 if 4 or more symptoms reported
**GS**	General Health Questionnaire GHQ-28 [Goldberg DP, Gater R, Sartorius N,et al.The validity of two versions of the GHQ in the WHO study of mental illness in general health care. Psychol Med 1997;27:191–7]	=1 if 24 or more
