# Supplementary material for: The UK Coronavirus Job Retention Scheme and smoking, alcohol consumption and vaping during the COVID-19 pandemic: evidence from eight longitudinal population surveys
Source: BMC Med. 2022 Sep 21;20:345. doi: 10.1186/s12916-022-02511-0 (PMC9489267; doi:10.1186/s12916-022-02511-0)
Supplement: Supplementary file 3 — Additional file 3. Meta-Analysis. Table 1. Main analysis excluding studies with ≤5 cell counts for exposure-outcome. Table 2. Main analysis excluding studies with ≤2 cell counts for exposure-outcome. Table 3. Main analysis excluding studies with zero cell counts for exposure-outcome. Table 4. Analysis of change excluding studies with ≤ 5 cell counts. Table 5. Analysis of change excluding studies with ≤ 2 cell counts. Table 6. Analysis of change excluding studies with zero cell counts. Figure set 1. Currently drinks 4+ days/week or 5+ drinks/occasion. Figure set 2. Increased alcohol consumption. Figure set 3. Reduced alcohol consumption. Figure set 4. Currently drinks 5+ drinks/occasion. Figure set 5. Drinks more alcohol units per occasion. Figure set 6. Drinks fewer alcohol units per occasion. Figure set 7. Currently drinks 4+ days/week. Figure set 8. Drinks more frequently. Figure set 9. Drinks less frequently. Figure set 10. Current smoker. Figure set 11. Smoking more. Figure set 12. Smoking less. Figure set 13. Current vaper. Figure set 14. Vaping more. Figure set 15. Vaping less. [file 12916_2022_2511_MOESM3_ESM.pdf]

Additional File 3: Meta-Analysis results

Summary Tables

Table 1. Main analysis excluding studies with ≤5 cell counts for exposure-outcome 2

Table 2. Main analysis excluding studies with ≤2 cell counts for exposure-outcome 3

Table 3. Main analysis excluding studies with zero cell counts for exposure-outcome 4

Table 4. Analysis of change excluding studies with ≤ 5 cell counts 5

Table 5. Analysis of change excluding studies with ≤ 2 cell counts 7

Table 6. Analysis of change excluding studies with zero cell counts 9

Forest Plots (*Unadjusted, Basic, and Fully adjusted*)

Drinking behaviours

Figure set 1. Currently drinks 4+ days/week or 5+ drinks/occasion 11

Figure set 2. Increased alcohol consumption 14

Figure set 3. Reduced alcohol consumption 19

Figure set 4. Currently drinks 5+ drinks/occasion 24

Figure set 5. Drinks more alcohol units per occasion 27

Figure set 6. Drinks fewer alcohol units per occasion 30

Figure set 7. Currently drinks 4+ days/week 33

Figure set 8. Drinks more frequently 37

Figure set 9. Drinks less frequently 40

Smoking and Vaping

Figure set 10. Current smoker 43

Figure set 11. Smoking more 46

Figure set 12. Smoking less 49

Figure set 13. Current vaper 52

Figure set 14. Vaping more 55

Figure set 15. Vaping less 58

Notes: Basic adjustment includes socio-demographic characteristics: age (only in age-heterogeneous studies), sex, ethnicity (except the BCS70 and NCDS cohorts which were nearly are almost entirely white), education, UK nation (except ALSPAC, GS and ELSA which only had participants from a single country), and household composition. Full adjustment additionally includes pre-pandemic measures of psychological distress, self-rated health, and (where appropriate) each outcome behaviours.

Table 1. Main analysis excluding studies with ≤5 cell counts for exposure-outcome

		Currently drinks 4+days/week or 5+ drinks/occasion				Current smoker				Current vaper			
		RR	Lower CI	Upper CI	I2%	RR	Lower CI	Upper CI	I2%	RR	Lower CI	Upper CI	I2%
Furloughed	Overall	1.03	0.94	1.13	47.92	1.05	0.95	1.16	9.86	0.91	0.74	1.1	0
	Female	0.99	0.88	1.10	43.16	52	1	1.25	0	0.92	0.71	1.2	0
	Male	1.06	0.98	1.14	0.63	1.04	0.87	1.24	31.05	0.87	0.66	1.16	0
	Degree	1.03	0.96	1.11	0	1.01	0.9	1.13	0	1.2	0.83	1.72	10.43
	No degree	1.06	0.93	1.20	52.47	1.07	0.97	1.17	0	0.81	0.65	1.02	0
	16-29y	0.92	0.75	1.12	0	1.24	0.97	1.59	0	0.88	0.5	1.57	0
	30-49y	0.98	0.89	1.09	0	1.04	0.88	1.24	0	0.96	0.57	1.62	54.6
	50+y	1.06	0.97	1.16	48.17	1.03	0.89	1.2	37.38	0.94	0.75	1.17	0
No longer employed	Overall	1.03	0.89	1.20	34.15	1.07	0.61	1.86	80.22	1.84	1	3.39	46.98
	Female	1.13	0.94	1.37	33.51	1.3	0.57	2.97	72.06	2.75	1.32	5.73	*
	Male	1.04	0.90	1.20	3.14	1.25	0.23	6.82	85.82	1.19	0.66	2.15	*
	Degree	1.10	0.97	1.25	0	1.08	0.84	1.39	0	no information			
	No degree	1.06	0.86	1.30	32.15	1.26	0.73	2.18	69.39	1.95	0.93	4.09	
	16-29y	1.07	0.62	1.85	56.05	1.45	0.6	3.51	75.09	no information			
	30-49y	0.98	0.82	1.19	0	0.77	0.43	1.37	0	1.96	0.42	9.29	87.47
	50+y	1.04	0.92	1.19	21.97	0.96	0.49	1.87	83.43	1.63	0.92	2.88	0
Stable unemployed	Overall	1.09	0.96	1.24	0	1.1	0.94	1.3	0	0.95	0.54	1.65	0
	Female	1.00	0.83	1.20	3.5	1.65	1.18	2.3	0	0.66	0.25	1.72	*
	Male	1.15	0.96	1.39	0	0.84	0.67	1.05	0	no information			
	Degree	0.98	0.70	1.36	46.26	1.03	0.8	1.34	0				
	No degree	1.09	0.94	1.26	0	1.17	0.99	1.39	0	1.01	0.53	1.9	0
	16-29y	1.17	0.87	1.56	0	1.28	0.7	2.33	*	1.25	0.53	2.95	*
	30-49y	1.10	0.87	1.39	*	1.07	0.7	1.63	*	no information			
	50+y	1.05	0.94	1.18	0	1.04	0.89	1.22	0	0.75	0.29	1.94	*

* indicates only one study included

Table 2. Main analysis excluding studies with ≤2 cell counts for exposure-outcome

		Currently drinks 4+days/week or 5+ drinks/occasion				Current smoker				Current vaper			
		RR	Lower CI	Upper CI	I2%	RR	Lower CI	Upper CI	I2%	RR	Lower CI	Upper CI	I2%
Furloughed	Overall	1.03	0.94	1.13	47.92	1.05	0.95	1.16	9.86	0.89	0.74	1.08	0
	Female	0.99	0.88	1.10	43.16	1.12	1	1.25	0	0.92	0.71	1.2	0
	Male	1.06	0.98	1.14	0.63	1.04	0.87	1.24	31.05	0.89	0.67	1.16	0
	Degree	1.03	0.96	1.11	0	1.03	0.9	1.12	0	1.2	0.88	1.65	1.58
	No degree	1.06	0.93	1.20	52.47	1.06	0.97	1.17	0	0.81	0.65	1.02	0
	16-29y	0.92	0.75	1.12	0	1.24	0.97	1.59	0	0.88	0.5	1.57	0
	30-49y	0.98	0.89	1.09	0	1.04	0.88	1.24	0	0.9	0.58	1.4	31.09
	50+y	1.06	0.97	1.16	48.17	1.03	0.89	1.2	37.38	0.94	0.75	1.17	0
No longer employed	Overall	1.03	0.89	1.20	34.15	1.02	0.66	1.56	70.57	1.51	0.92	2.5	43.69
	Female	1.12	0.92	1.36	29.92	1.12	0.74	1.69	52.12	2.74	1.59	4.72	0
	Male	1.03	0.90	1.19	0.75	1.11	0.54	2.28	76.84	1.25	0.83	1.87	0
	Degree	1.08	0.96	1.22	0	0.92	0.7	1.22	23.79	3.91	1.18	12.9	*
	No degree	1.06	0.86	1.30	32.15	0.93	0.62	1.41	61.88	1.51	0.89	2.55	0
	16-29y	1.07	0.62	1.85	56.05	1.45	0.6	3.51	75.09	1.77	0.36	8.61	86.17
	30-49y	0.98	0.82	1.19	0	0.77	0.43	1.37	0	1.96	0.42	9.29	87.47
	50+y	1.04	0.92	1.19	21.97	0.87	0.54	1.39	67.75	1.55	0.93	2.56	0
Stable unemployed	Overall	1.09	0.96	1.24	0	1.1	0.93	1.29	0	0.98	0.62	1.57	0
	Female	1.08	0.79	1.47	18.4	1.35	1	1.82	47	0.79	0.36	1.72	0
	Male	1.15	0.96	1.39	0	0.84	0.67	1.05	0	0.89	0.36	2.21	0
	Degree	0.98	0.70	1.36	46.26	1.01	0.78	1.29	0	no information			
	No degree	1.09	0.94	1.26	0	1.17	0.99	1.39	0	1.01	0.53	1.9	0
	16-29y	1.17	0.87	1.56	0	1.34	0.78	2.3	0	1.25	0.53	2.95	*
	30-49y	1.10	0.87	1.39	*	1.03	0.69	1.53	0	0.88	0.34	2.29	0
	50+y	1.05	0.94	1.18	0	1.04	0.89	1.22	0	0.9	0.45	1.77	0

Note: I2 estimates from stratified models are different to the overall I2 presented in subgroup figures as separate models were run for each group.
* indicates only one study included

Table 3. Main analysis excluding studies with zero cell counts for exposure-outcome

		Currently drinks 4+days/week or 5+ drinks/occasion				Current smoker				Current vaper			
		RR	Lower CI	Upper CI	I2%	RR	Lower CI	Upper CI	I2%	RR	Lower CI	Upper CI	I2%
Furloughed	Overall	1.03	0.94	1.13	47.92	1.05	0.95	1.16	9.86	0.89	0.74	1.08	0
	Female	0.99	0.88	1.10	43.16	1.12	1	1.25	0	0.92	0.71	1.2	0
	Male	1.06	0.98	1.14	0.63	1.01	0.83	1.22	36.58	0.89	0.67	1.16	0
	Degree	1.03	0.96	1.11	0	1	0.9	1.12	0	1.2	0.88	1.65	1.58
	No degree	1.06	0.93	1.20	52.47	1.07	0.97	1.17	0	0.81	0.65	1.02	0
	16-29y	0.92	0.75	1.12	0	1.24	0.97	1.59	0	0.88	0.5	1.57	0
	30-49y	0.98	0.89	1.09	0	1.04	0.88	1.24	0	0.9	0.58	1.4	31.09
	50+y	1.06	0.97	1.16	48.17	1.03	0.89	1.2	37.38	0.94	0.75	1.17	0
No longer employed	Overall	1.03	0.89	1.20	34.15	1.02	0.66	1.56	70.57	1.51	0.92	2.5	43.69
	Female	1.12	0.92	1.36	29.92	1.04	0.69	1.58	52.05	1.1	0.35	3.45	74.79
	Male	1.03	0.90	1.19	0.75	1.05	0.56	1.98	72.47	1.41	0.98	2.03	0
	Degree	1.08	0.96	1.22	0	0.91	0.69	1.19	20.25	1.76	0.96	3.25	6.66
	No degree	1.05	0.85	1.28	27.01	0.93	0.62	1.41	61.88	1.28	0.81	2.03	10.84
	16-29y	1.07	0.62	1.85	56.05	1.45	0.6	3.51	75.09	1.77	0.36	8.61	86.17
	30-49y	0.98	0.82	1.19	0	0.94	0.57	1.53	0	1.67	0.58	4.85	72.6
	50+y	1.04	0.92	1.19	21.97	0.87	0.54	1.39	67.75	1.4	0.86	2.27	0
Stable unemployed	Overall	1.24	0.95	1.63	41.28	1.1	0.93	1.29	0	1.36	0.73	2.52	52.27
	Female	1.00	0.87	1.15	0	1.35	1	1.82	47	0.91	0.48	1.73	0
	Male	1.32	0.93	1.86	46.4	0.84	0.67	1.04	0	1.72	0.42	7.04	79.83
	Degree	0.95	0.70	1.28	29.76	0.65	0.27	1.56	89.56	0.86	0.52	1.42	0
	No degree	1.32	0.93	1.87	44.03	1.17	0.98	1.38	0	1.49	0.57	3.9	70.57
	16-29y	1.17	0.87	1.56	0	1.34	0.78	2.3	0	1.25	0.53	2.95	*
	30-49y	0.95	0.47	1.94	25.14	1.12	0.79	1.6	0	0.88	0.34	2.29	0
	50+y	1.23	0.89	1.70	63.19	1.04	0.89	1.22	0	1.48	0.56	3.89	70.06

* indicates only one study included

Table 4. Analysis of change excluding studies with ≤ 5 cell counts

		Drinking more alcohol				Drinking less alcohol				Smoking more			
		RR	Lower CI	Upper CI	I2%	RR	Lower CI	Upper CI	I2%	RR	Lower CI	Upper CI	I2%
Furloughed	Overall	1.11	0.98	1.26	71.15	0.97	0.85	1.11	43.66	1.31	0.88	1.96	65.25
	Female	1.02	0.93	1.12	23.96	0.78	0.46	1.31	79.99	1.46	1.14	1.87	0.00
	Male	1.33	1.08	1.64	73.84	0.97	0.86	1.09	0	2.08	0.71	6.14	81.98
	Degree	1.02	0.90	1.17	40.63	1.18	0.95	1.45	43.64	1.44	0.93	2.25	0.00
	No degree	1.18	0.99	1.42	77.36	0.89	0.77	1.03	31.74	1.48	0.91	2.40	72.41
	16-29y	0.98	0.73	1.32	61.21	0.96	0.82	1.13	10.62	1.56	0.82	2.99	31.39
	30-49y	1.05	0.82	1.35	71.96	0.95	0.83	1.10	0	0.71	0.42	1.21	0.00
	50+y	1.19	1.09	1.30	27.54	1.05	0.95	1.17	0	1.46	0.95	2.24	51.93
No longer employed	Overall	0.99	0.88	1.13	0	1.11	0.95	1.28	0	0.84	0.35	2.01	*
	Female	1.00	0.87	1.14	0	1.21	0.73	2.02	74.16	no information			
	Male	1.05	0.77	1.42	27.8	1.03	0.79	1.33	0	no information			
	Degree	1.04	0.89	1.22	0	1.28	0.88	1.84	38.4	no information			
	No degree	0.98	0.83	1.16	0	1.13	0.92	1.39	0	0.84	0.30	2.35	*
	16-29y	0.99	0.74	1.32	0	1.24	1.01	1.53	0	no information			
	30-49y	0.88	0.63	1.21	0	0.86	0.56	1.33	0	no information			
	50+y	1.04	0.92	1.17	0	0.99	0.82	1.20	0	no information			
Stable unemployed	Overall	0.73	0.53	1.00	33.12	0.97	0.75	1.25	15.96	2.49	0.88	7.07	*
	Female	0.64	0.47	0.88	12.45	0.83	0.40	1.72	58.75	no information			
	Male	0.89	0.59	1.35	24.58	1.32	0.82	2.13	55.54	no information			
	Degree	0.68	0.33	1.39	52.65	1.28	0.48	3.45	69.58	no information			
	No degree	0.81	0.57	1.15	26.19	0.88	0.66	1.17	4.99	no information			
	16-29y	0.63	0.41	0.98	0	0.86	0.62	1.19	0	no information			
	30-49y	0.40	0.19	0.81	*	0.74	0.39	1.41	*	2.49	0.88	7.07	*
	50+y	0.89	0.74	1.07	0	1.01	0.69	1.48	31.33	no information			

* indicates only one study included

		Smoking less				Vaping more				Vaping less			
		RR	Lower CI	Upper CI	I2%	RR	Lower CI	Upper CI	I2%	RR	Lower CI	Upper CI	I2%
Furloughed	Overall	1.42	0.86	2.33	68.92	1.53	1.14	2.07	0.00	1.30	0.82	2.06	*
	Female	0.79	0.45	1.39	*	1.64	0.93	2.90	42.27	1.58	0.85	2.95	*
	Male	1.42	0.76	2.66	56.85	1.72	0.88	3.36	38.79	1.03	0.53	2.01	*
	Degree	2.07	0.95	4.53	35.49	2.71	1.59	4.62	0.00		no information		
	No degree	1.31	0.75	2.31	68.46	1.32	0.74	2.37	59.43	1.48	0.88	2.48	
	16-29y	1.10	0.14	8.76	92.84	1.45	0.58	3.66	36.77	2.45	1.04	5.77	*
	30-49y	1.79	0.70	4.58	78.27	1.67	0.48	5.85	75.83	1.05	0.54	2.03	*
	50+y	0.96	0.65	1.41	0.00	1.44	0.91	2.27	0.00	0.73	0.29	1.89	*
No longer employed	Overall	3.23	1.30	8.04	71.26	2.45	1.16	5.15	*	1.00	0.33	3.00	*
	Female	2.72	1.60	4.63	*		no information				no information		
	Male		no information				no information				no information		
	Degree	8.90	1.83	43.25	*		no information				no information		
	No degree	2.13	1.07	4.23	*	2.60	1.15	5.91	*		no information		
	16-29y	3.14	1.18	8.38	74.59		no information				no information		
	30-49y		no information				no information				no information		
	50+y		no information				no information				no information		
Stable unemployed	Overall	1.65	0.94	2.89	0.00	1.44	0.48	4.31	*		no information		
	Female		no information				no information				no information		
	Male	1.59	0.71	3.53	*		no information				no information		
	Degree		no information				no information				no information		
	No degree	1.70	0.93	3.09	0.00		no information				no information		
	16-29y	2.45	0.66	9.08	*		no information				no information		
	30-49y		no information				no information				no information		
	50+y	2.74	1.44	5.20	*		no information				no information		

* indicates only one study included

Table 5. Analysis of change excluding studies with ≤ 2 cell counts

		Drinking more alcohol				Drinking less alcohol				Smoking more			
		RR	Lower CI	Upper CI	I2%	RR	Lower CI	Upper CI	I2%	RR	Lower CI	Upper CI	I2%
Furloughed	Overall	1.11	0.98	1.26	71.15	0.97	0.85	1.11	43.66	1.31	0.88	1.96	65.25
	Female	1.02	0.93	1.12	23.96	0.78	0.46	1.31	79.99	1.46	1.14	1.87	0.00
	Male	1.33	1.08	1.64	73.84	0.97	0.86	1.09	0	1.76	0.81	3.83	69.91
	Degree	1.02	0.90	1.17	40.63	1.14	0.92	1.41	41.94	1.44	0.93	2.25	0.00
	No degree	1.18	0.99	1.42	77.36	0.89	0.77	1.03	31.74	1.48	0.91	2.40	72.41
	16-29y	0.98	0.73	1.32	61.21	0.96	0.82	1.13	10.62	1.56	0.82	2.99	31.39
	30-49y	1.05	0.82	1.35	71.96	0.95	0.83	1.10	0	0.74	0.46	1.21	0.00
	50+y	1.20	1.09	1.32	32.89	1.05	0.95	1.17	0	1.46	0.95	2.24	51.93
No longer employed	Overall	0.99	0.87	1.11	0	1.11	0.95	1.28	0	1.72	0.67	4.45	47.68
	Female	1.00	0.87	1.14	0	1.21	0.73	2.02	74.16	1.77	0.45	6.98	71.72
	Male	1.01	0.81	1.26	0.1	1.03	0.82	1.31	0	0.81	0.22	3.06	*
	Degree	1.03	0.88	1.20	0	1.15	0.94	1.40	0	no information			
	No degree	0.98	0.83	1.16	0	1.13	0.92	1.39	0	2.20	0.28	17.00	79.55
	16-29y	0.99	0.74	1.32	0	1.24	1.01	1.53	0	1.55	0.28	8.69	62.63
	30-49y	0.88	0.63	1.21	0	0.93	0.63	1.37	0	1.56	0.46	5.23	*
	50+y	1.02	0.89	1.16	0	0.99	0.82	1.20	0	2.54	0.66	9.71	*
Stable unemployed	Overall	0.68	0.50	0.91	27.75	0.96	0.75	1.22	10.79	1.20	0.50	2.91	46.60
	Female	0.65	0.48	0.88	11.05	0.86	0.49	1.51	37.57	2.55	0.85	7.71	58.12
	Male	0.91	0.63	1.33	15.09	1.24	0.84	1.81	34.45	no information			
	Degree	0.62	0.36	1.05	34.23	1.80	1.09	2.96	51.36	no information			
	No degree	0.75	0.54	1.05	22.16	0.82	0.63	1.06	0	1.24	0.45	3.41	53.56
	16-29y	0.63	0.41	0.98	0	0.86	0.62	1.19	0	1.19	0.33	4.35	*
	30-49y	0.38	0.21	0.70	0	0.74	0.39	1.41	*	2.49	0.88	7.07	*
	50+y	0.85	0.66	1.09	0	1.01	0.72	1.42	21.01	0.62	0.19	2.07	*

* indicates only one study included

		Smoking less				Vaping more				Vaping less			
		RR	Lower CI	Upper CI	I2%	RR	Lower CI	Upper CI	I2%	RR	Lower CI	Upper CI	I2%
Furloughed	Overall	1.43	0.91	2.25	63.08	1.53	1.14	2.07	0.00	1.42	0.94	2.14	0.00
	Female	1.12	0.70	1.80	26.26	1.54	0.96	2.46	25.25	1.58	0.85	2.95	*
	Male	1.61	0.91	2.85	59.77	1.61	1.04	2.51	0.00	1.09	0.61	1.97	0.00
	Degree	1.61	1.04	2.50	0.00	2.93	1.80	4.75	0.00	0.72	0.26	1.99	*
	No degree	1.31	0.75	2.31	68.46	1.31	0.79	2.19	50.61	1.54	0.95	2.49	0.00
	16-29y	1.10	0.14	8.76	92.84	1.45	0.58	3.66	36.77	2.39	1.08	5.30	0.00
	30-49y	1.93	0.92	4.04	59.34	1.67	0.48	5.85	75.83	1.05	0.54	2.03	*
	50+y	0.96	0.65	1.41	0.00	1.56	1.04	2.34	0.00	1.23	0.58	2.60	15.56
No longer employed	Overall	2.79	1.44	5.39	49.98	3.21	1.51	6.80	23.12	1.14	0.42	3.09	0.00
	Female	2.69	1.68	4.30	0.00	3.46	1.75	6.83	0.00	0.99	0.23	4.27	*
	Male	2.96	0.44	20.16	79.80	1.57	0.43	5.68	*	1.32	0.28	6.22	*
	Degree	3.71	0.92	14.96	58.89	11.29	2.20	58.03	*	2.21	0.47	10.37	*
	No degree	2.57	1.55	4.26	0.00	2.60	1.15	5.91	*	0.90	0.24	3.40	*
	16-29y	3.14	1.18	8.38	74.59	4.98	1.97	12.57	*	2.15	0.19	24.37	*
	30-49y	1.57	0.73	3.35	*	5.51	1.69	17.93	*	2.59	0.58	11.60	*
	50+y	1.21	0.52	2.81	0.00	4.16	1.68	10.30	*	no information			
Stable unemployed	Overall	1.74	1.06	2.86	0.00	1.97	0.93	4.17	20.23	1.64	0.54	5.00	*
	Female	3.10	1.17	8.19	52.49	1.30	0.36	4.77	*	no information			
	Male	1.77	0.87	3.61	0.00	no information				no information			
	Degree	no information				no information				no information			
	No degree	1.84	1.06	3.21	0.00	1.47	0.57	3.82	0.00	1.85	0.58	5.92	*
	16-29y	2.45	0.66	9.08	*	0.76	0.14	4.27	*	no information			
	30-49y	1.63	0.29	9.30	*	1.86	0.53	6.58	0.00	no information			
	50+y	2.69	1.50	4.80	0.00	1.98	0.44	8.89	69.74	1.80	0.51	6.38	*

* indicates only one study included

Table 6. Analysis of change excluding studies with zero cell counts

		Drinking more alcohol				Drinking less alcohol				Smoking more			
		RR	Lower CI	Upper CI	I2%	RR	Lower CI	Upper CI	I2%	RR	Lower CI	Upper CI	I2%
Furloughed	Overall	1.11	0.98	1.26	71.15	0.97	0.85	1.11	43.66	1.31	0.88	1.96	65.25
	Female	1.02	0.93	1.12	23.96	0.78	0.46	1.31	79.99	1.46	1.14	1.87	0.00
	Male	1.33	1.08	1.64	73.84	0.97	0.86	1.09	0	1.16	0.40	3.38	84.27
	Degree	1.02	0.90	1.17	40.63	1.14	0.92	1.41	41.94	1.15	0.74	1.79	6.66
	No degree	1.18	0.99	1.42	77.36	0.89	0.77	1.03	31.74	1.48	0.91	2.40	72.41
	16-29y	0.98	0.73	1.32	61.21	0.96	0.82	1.13	10.62	1.56	0.82	2.99	31.39
	30-49y	1.05	0.82	1.35	71.96	0.95	0.83	1.10	0	0.74	0.46	1.21	0.00
	50+y	1.19	1.09	1.30	27.54	1.05	0.95	1.17	0	1.46	0.95	2.24	51.93
No longer employed	Overall	0.99	0.87	1.11	0	1.08	0.93	1.26	0	1.07	0.65	1.77	4.98
	Female	1.00	0.87	1.14	0	1.21	0.73	2.02	74.16	1.27	0.60	2.68	31.61
	Male	0.99	0.79	1.23	0.7	0.99	0.79	1.25	0	1.36	0.68	2.71	0.00
	Degree	1.02	0.87	1.19	0	1.15	0.94	1.40	0	0.87	0.38	1.99	0.00
	No degree	0.98	0.83	1.16	0	1.08	0.88	1.32	0	1.12	0.45	2.77	59.50
	16-29y	0.99	0.74	1.32	0	1.24	1.01	1.53	0	1.55	0.28	8.69	62.63
	30-49y	0.88	0.63	1.21	0	0.93	0.63	1.37	0	1.05	0.42	2.59	0.00
	50+y	1.04	0.92	1.17	0	0.96	0.80	1.16	0	0.92	0.48	1.77	0.00
Stable unemployed	Overall	0.73	0.55	0.96	24.7	0.93	0.74	1.17	5.25	1.12	0.60	2.08	27.57
	Female	0.70	0.54	0.91	3.7	0.90	0.57	1.42	26.06	2.35	1.17	4.70	20.85
	Male	0.91	0.67	1.24	0.00	1.20	0.85	1.70	24.49	0.88	0.32	2.41	0.00
	Degree	0.67	0.43	1.04	21.73	1.59	0.98	2.58	46.62	2.48	0.73	8.46	0.00
	No degree	0.78	0.59	1.05	11.39	0.81	0.63	1.05	0	1.17	0.60	2.28	31.00
	16-29y	0.63	0.41	0.98	0	0.86	0.62	1.19	0	1.02	0.36	2.93	0.00
	30-49y	0.84	0.18	3.90	92.55	0.73	0.40	1.33	0	2.49	0.88	7.07	*
	50+y	0.88	0.73	1.05	0	0.98	0.70	1.37	16.72	0.83	0.40	1.72	0.00

		Smoking less				Vaping more				Vaping less			
		RR	Lower CI	Upper CI	I2%	RR	Lower CI	Upper CI	I2%	RR	Lower CI	Upper CI	I2%
Furloughed	Overall	1.43	0.91	2.25	63.08	1.53	1.14	2.07	0.00	1.41	0.94	2.11	0.00
	Female	0.88	0.43	1.80	27.97	1.54	0.96	2.46	25.25	1.59	0.87	2.91	0.00
	Male	1.53	0.90	2.61	53.22	1.63	1.06	2.50	0.00	1.13	0.64	1.98	0.00
	Degree	1.83	1.14	2.95	16.40	2.70	1.73	4.22	0.00	2.58	0.40	16.60	72.11
	No degree	1.29	0.77	2.17	62.51	1.31	0.79	2.19	50.61	1.55	0.96	2.48	0.00
	16-29y	1.10	0.14	8.76	92.84	1.45	0.58	3.66	36.77	2.39	1.08	5.30	0.00
	30-49y	1.93	0.92	4.04	59.34	1.44	0.52	3.96	58.64	1.05	0.54	2.03	*
	50+y	0.98	0.68	1.43	0.00	1.56	1.04	2.34	0.00	1.47	0.65	3.32	24.93
No longer employed	Overall	2.51	1.48	4.25	28.06	2.61	1.29	5.27	21.85	1.60	0.66	3.87	1.66
	Female	2.76	1.71	4.47	0.00	2.88	1.52	5.45	0.00	0.99	0.23	4.27	*
	Male	2.12	0.76	5.97	46.32	1.95	0.70	5.42	0.00	1.79	0.51	6.29	0.00
	Degree	3.64	1.50	8.81	29.96	2.51	0.44	14.30	59.42	2.21	0.47	10.37	*
	No degree	2.54	1.57	4.12	0.00	2.33	1.16	4.69	0.00	1.31	0.47	3.71	2.37
	16-29y	3.14	1.18	8.38	74.59	4.98	1.97	12.57	*	1.13	0.24	5.29	0.00
	30-49y	1.63	0.82	3.25	0.00	1.99	0.18	21.77	70.24	3.33	0.95	11.69	0.00
	50+y	1.27	0.58	2.77	0.00	2.31	0.56	9.51	60.40	1.39	0.16	12.08	50.66
Stable unemployed	Overall	1.74	1.06	2.86	0.00	1.97	0.93	4.17	20.23	1.41	0.24	8.26	70.08
	Female	1.76	0.73	4.23	0.00	2.02	0.91	4.47	0.00	2.77	0.51	14.89	*
	Male	1.72	0.87	3.40	0.00	2.06	0.70	6.09	0.00	1.15	0.25	5.38	*
	Degree	1.46	0.37	5.76	36.01	0.82	0.06	10.87	68.81	no information			
	No degree	1.77	1.03	3.04	0.00	2.07	1.00	4.28	0.00	1.86	0.20	17.47	74.48
	16-29y	1.20	0.21	6.77	51.82	0.76	0.14	4.27	*	0.84	0.02	28.67	75.19
	30-49y	1.31	0.43	4.04	0.00	1.86	0.53	6.58	0.00	5.30	1.15	24.41	*
	50+y	2.69	1.50	4.80	0.00	1.98	0.44	8.89	69.74	1.80	0.51	6.38	*

* indicates only one study included

Figure set 1. Currently drinks 4+ days/week or 5+ drinks/occasion

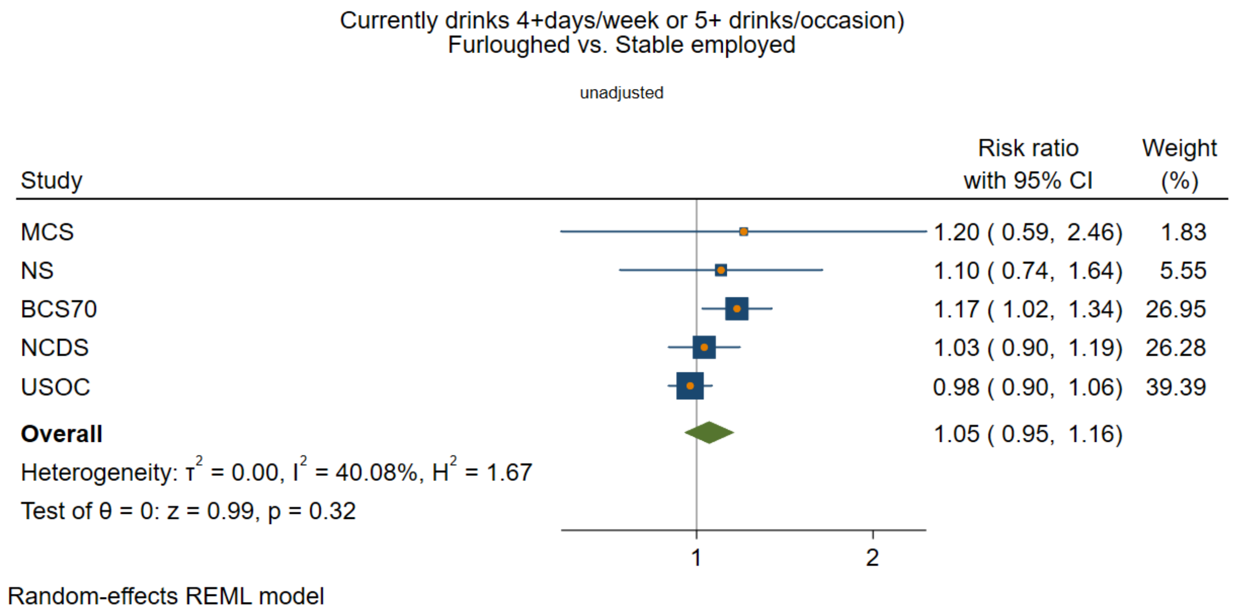

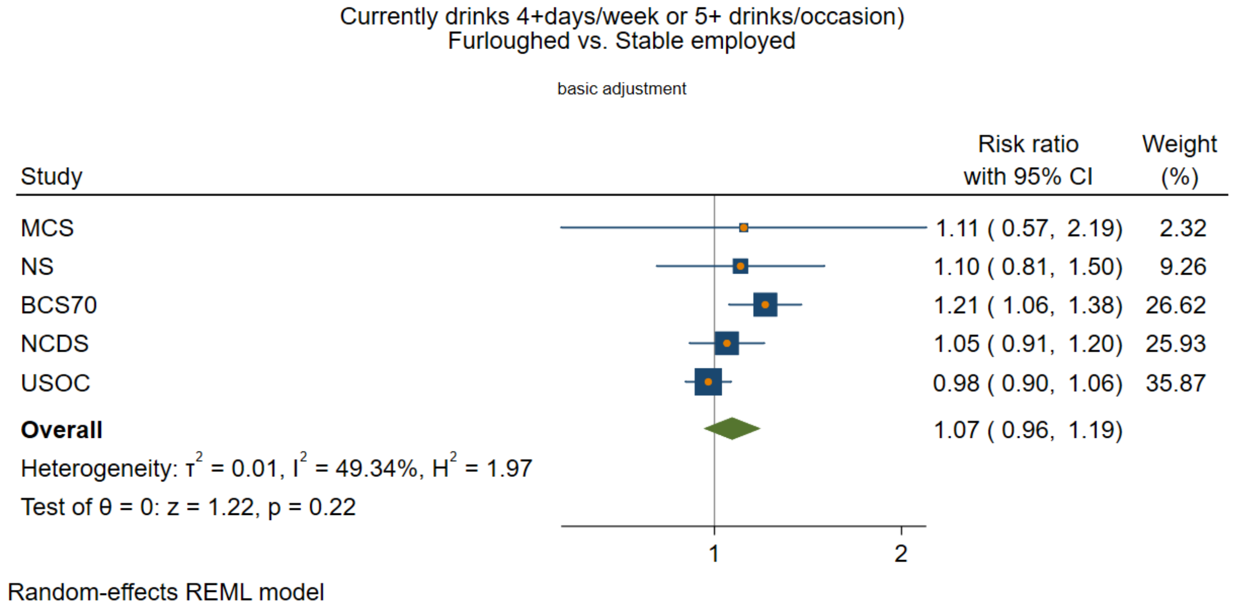

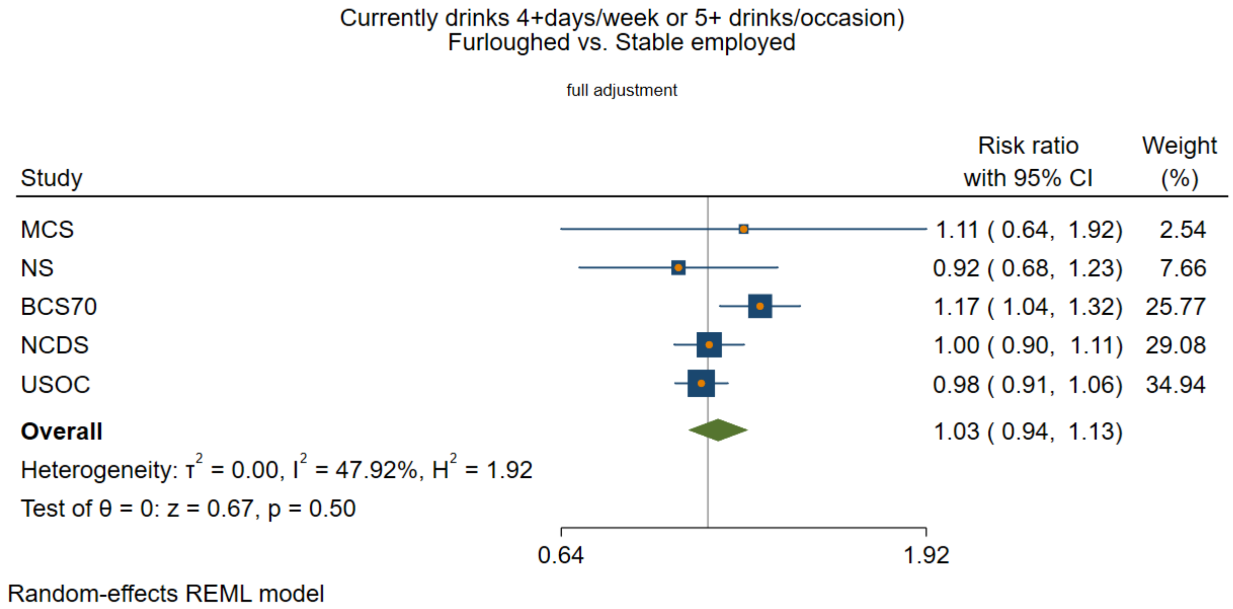

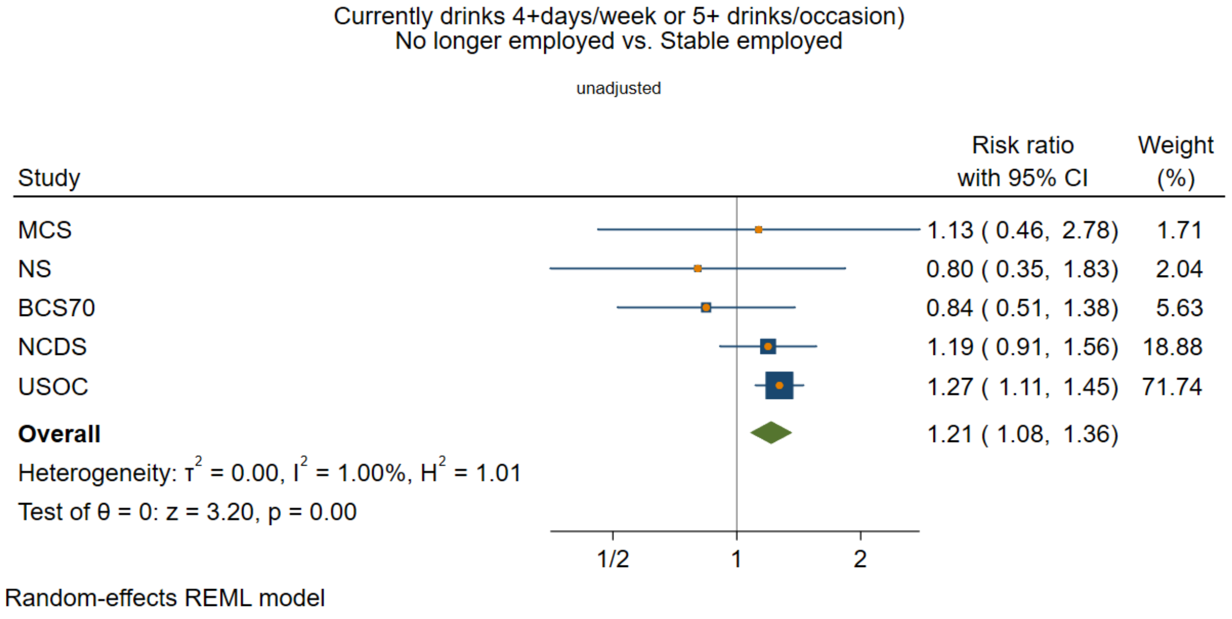
Currently drinks 4+days/week or 5+ drinks/occasion)
No longer employed vs. Stable employed
unadjusted
Study
Risk ratio with 95% CI
Weight (%)
MCS 1.13 (0.46, 2.78) 1.71
NS 0.80 (0.35, 1.83) 2.04
BCS70 0.84 (0.51, 1.38) 5.63
NCDS 1.19 (0.91, 1.56) 18.88
USOC 1.27 (1.11, 1.45) 71.74
Overall 1.21 (1.08, 1.36)
Heterogeneity: τ^2 = 0.00, I^2 = 1.00%, H^2 = 1.01
Test of θ = 0: z = 3.20, p = 0.00
1/2
1
2
Random-effects REML model

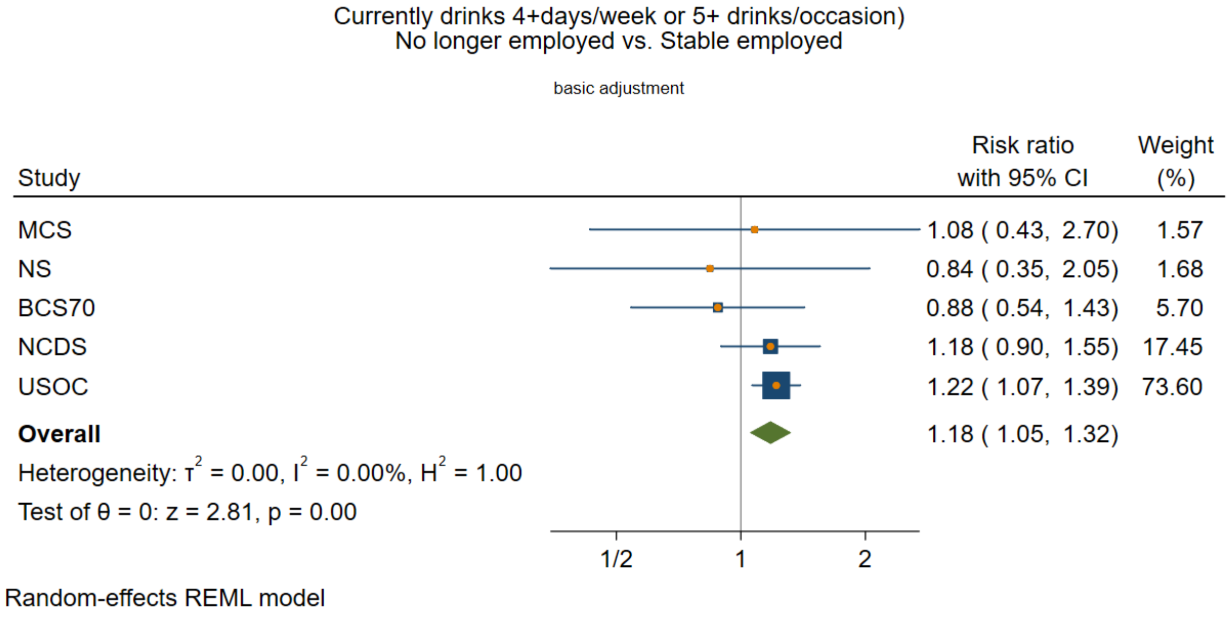
Currently drinks 4+days/week or 5+ drinks/occasion)
No longer employed vs. Stable employed
basic adjustment
Study
Risk ratio with 95% CI
Weight (%)
MCS 1.08 (0.43, 2.70) 1.57
NS 0.84 (0.35, 2.05) 1.68
BCS70 0.88 (0.54, 1.43) 5.70
NCDS 1.18 (0.90, 1.55) 17.45
USOC 1.22 (1.07, 1.39) 73.60
Overall 1.18 (1.05, 1.32)
Heterogeneity: τ^2 = 0.00, I^2 = 0.00%, H^2 = 1.00
Test of θ = 0: z = 2.81, p = 0.00
1/2
1
2
Random-effects REML model

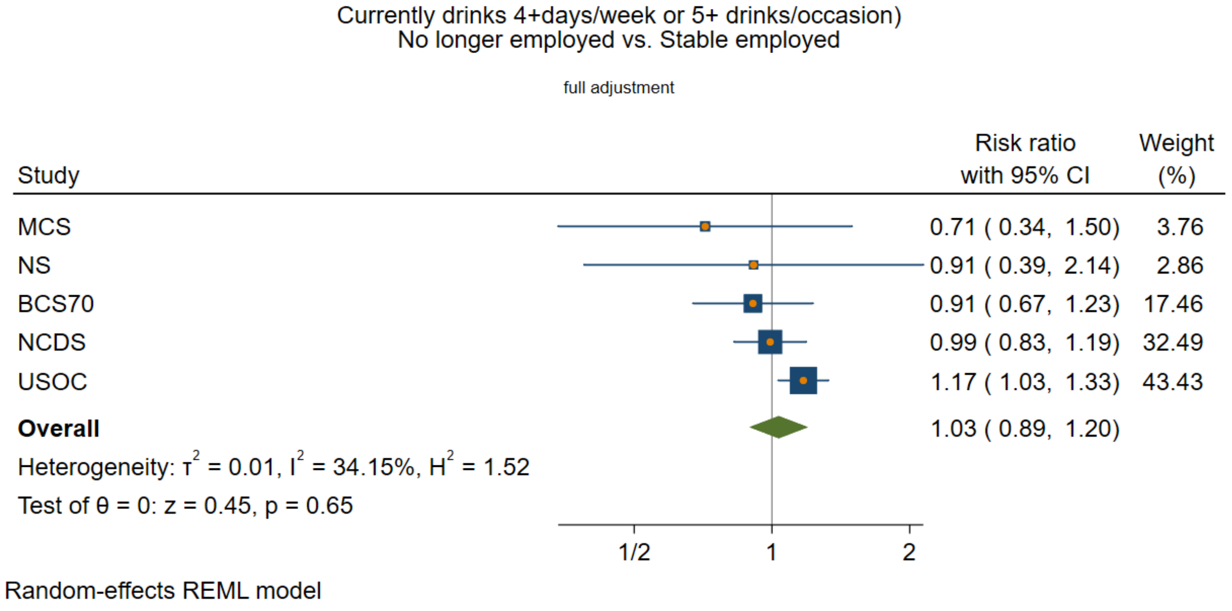
Currently drinks 4+days/week or 5+ drinks/occasion)
No longer employed vs. Stable employed
full adjustment
Study
Risk ratio with 95% CI
Weight (%)
MCS 0.71 (0.34, 1.50) 3.76
NS 0.91 (0.39, 2.14) 2.86
BCS70 0.91 (0.67, 1.23) 17.46
NCDS 0.99 (0.83, 1.19) 32.49
USOC 1.17 (1.03, 1.33) 43.43
Overall 1.03 (0.89, 1.20)
Heterogeneity: τ^2 = 0.01, I^2 = 34.15%, H^2 = 1.52
Test of θ = 0: z = 0.45, p = 0.65
1/2
1
2
Random-effects REML model

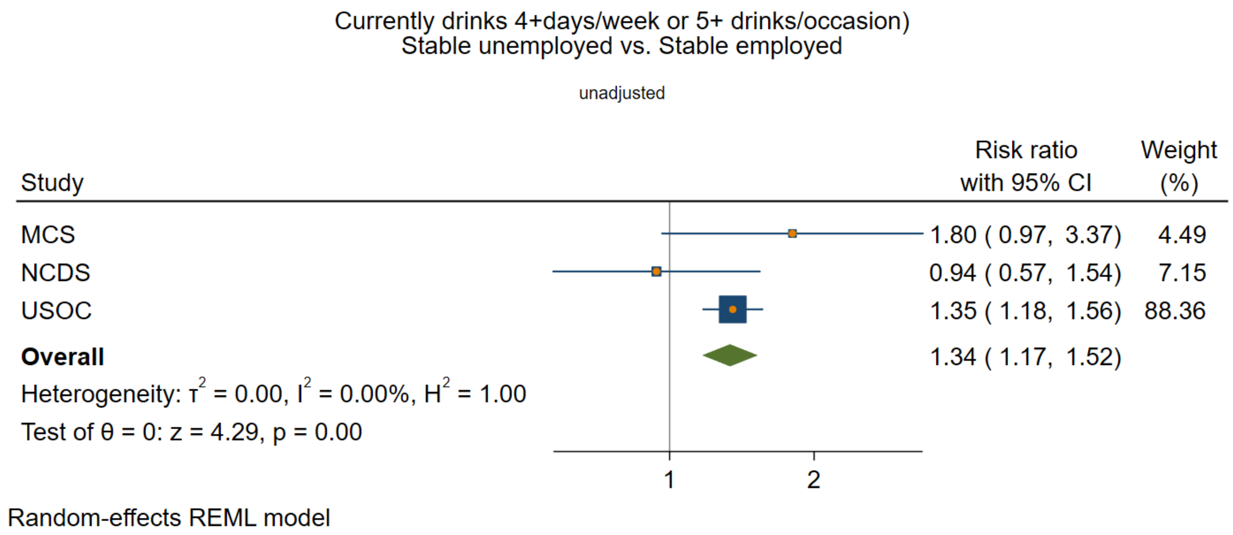
Currently drinks 4+days/week or 5+ drinks/occasion)
Stable unemployed vs. Stable employed
unadjusted
Study
Risk ratio with 95% CI
Weight (%)
MCS 1.80 (0.97, 3.37) 4.49
NCDS 0.94 (0.57, 1.54) 7.15
USOC 1.35 (1.18, 1.56) 88.36
Overall 1.34 (1.17, 1.52)
Heterogeneity: τ² = 0.00, I² = 0.00%, H² = 1.00
Test of θ = 0: z = 4.29, p = 0.00
1
2
Random-effects REML model

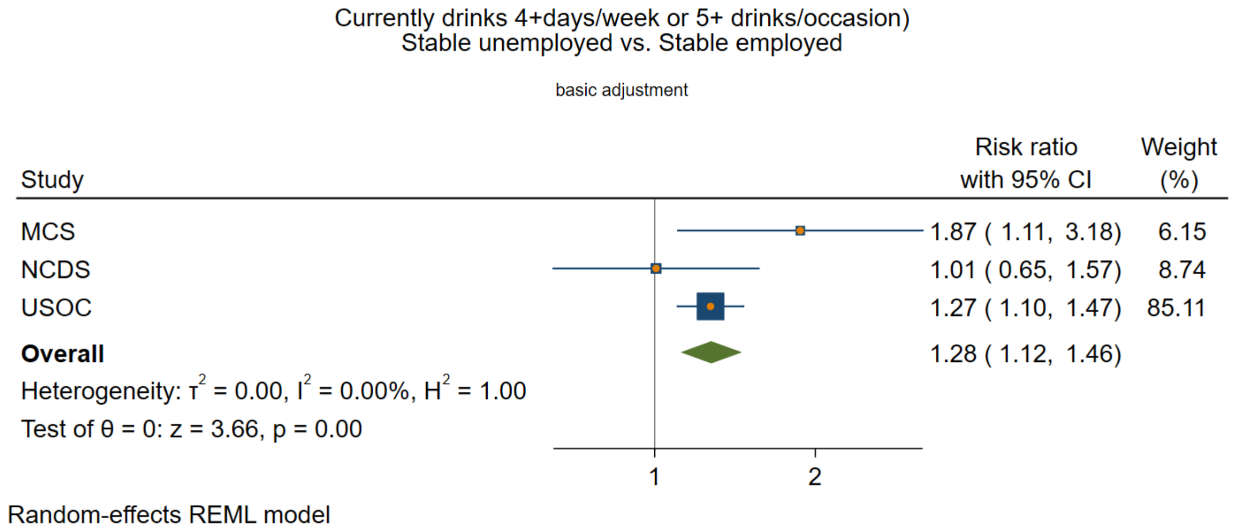
Currently drinks 4+days/week or 5+ drinks/occasion)
Stable unemployed vs. Stable employed
basic adjustment
Study
Risk ratio with 95% CI
Weight (%)
MCS 1.87 (1.11, 3.18) 6.15
NCDS 1.01 (0.65, 1.57) 8.74
USOC 1.27 (1.10, 1.47) 85.11
Overall 1.28 (1.12, 1.46)
Heterogeneity: τ² = 0.00, I² = 0.00%, H² = 1.00
Test of θ = 0: z = 3.66, p = 0.00
1
2
Random-effects REML model

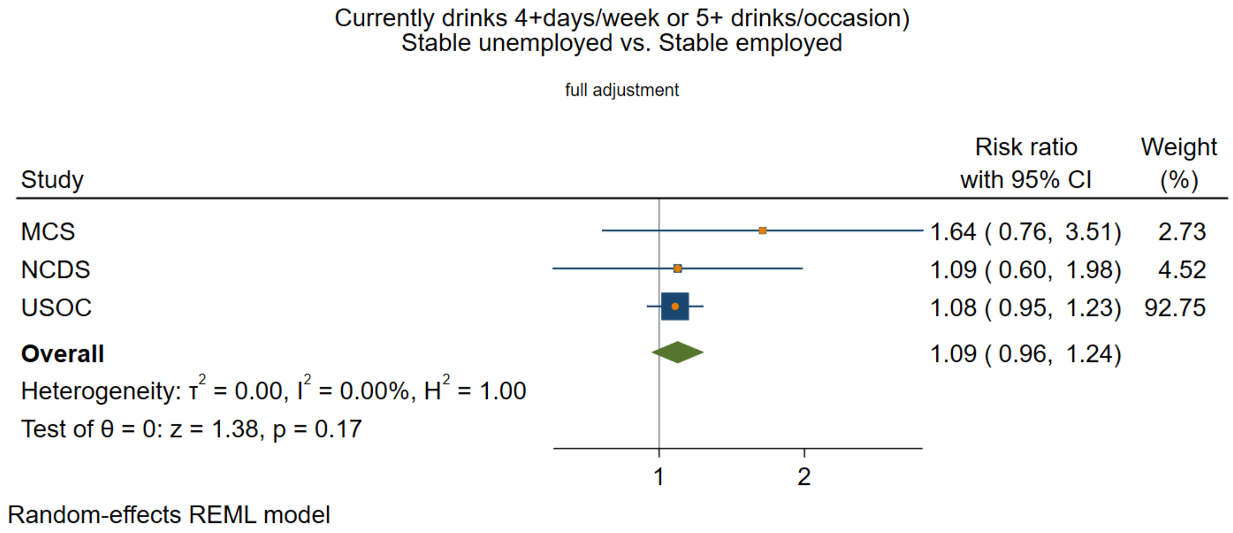
Currently drinks 4+days/week or 5+ drinks/occasion)
Stable unemployed vs. Stable employed
full adjustment
Study
Risk ratio with 95% CI
Weight (%)
MCS 1.64 (0.76, 3.51) 2.73
NCDS 1.09 (0.60, 1.98) 4.52
USOC 1.08 (0.95, 1.23) 92.75
Overall 1.09 (0.96, 1.24)
Heterogeneity: τ² = 0.00, I² = 0.00%, H² = 1.00
Test of θ = 0: z = 1.38, p = 0.17
1
2
Random-effects REML model

Figure set 2. Increased alcohol consumption

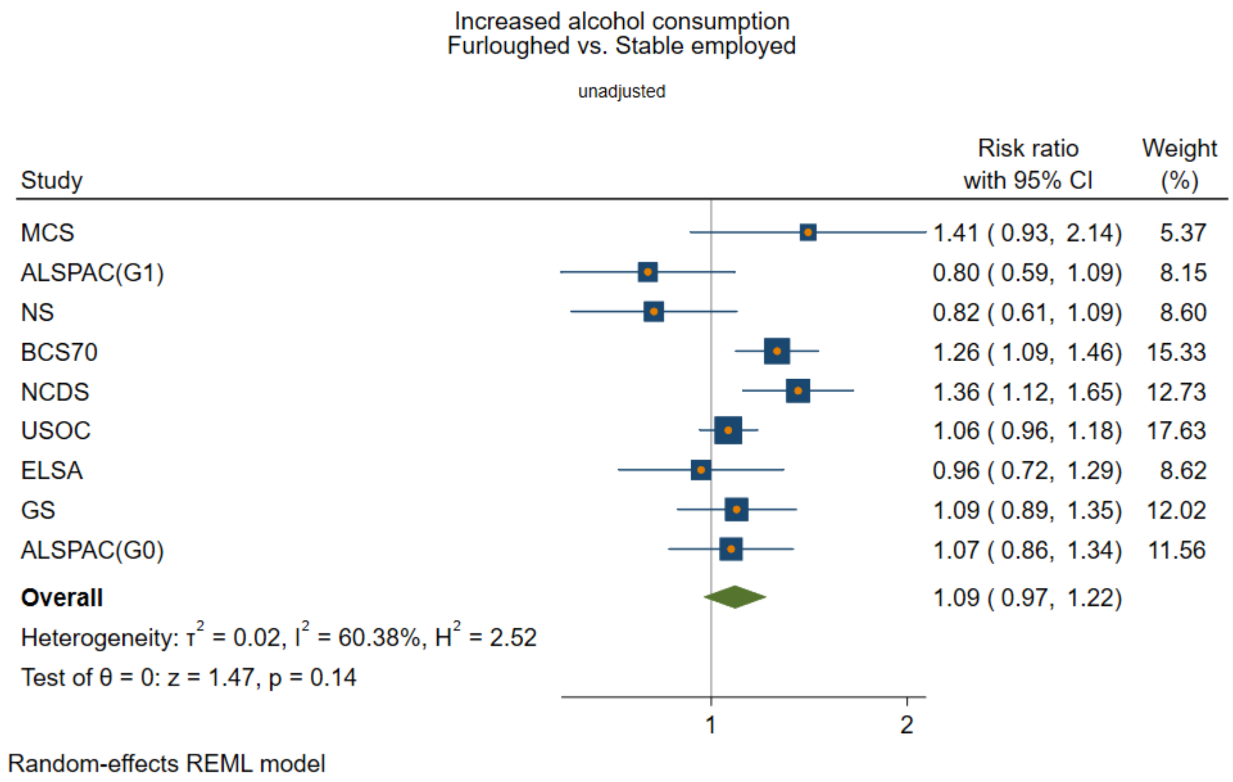

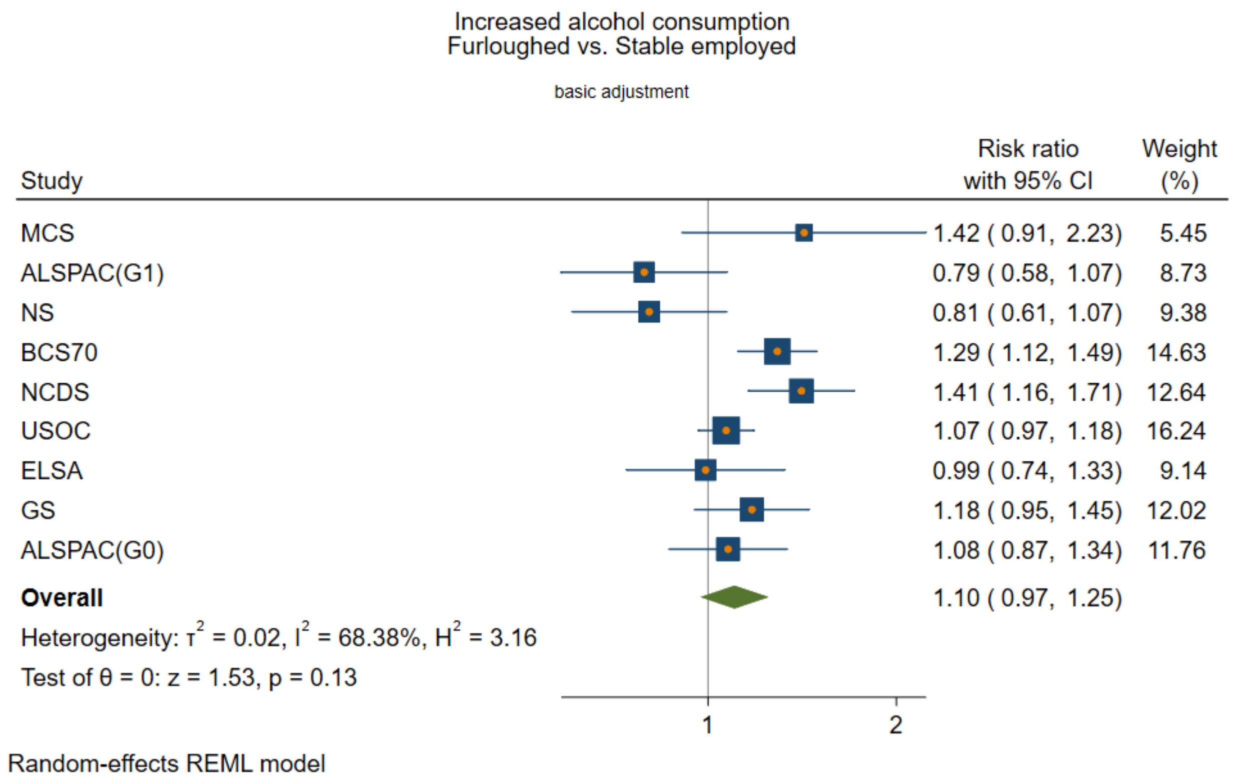

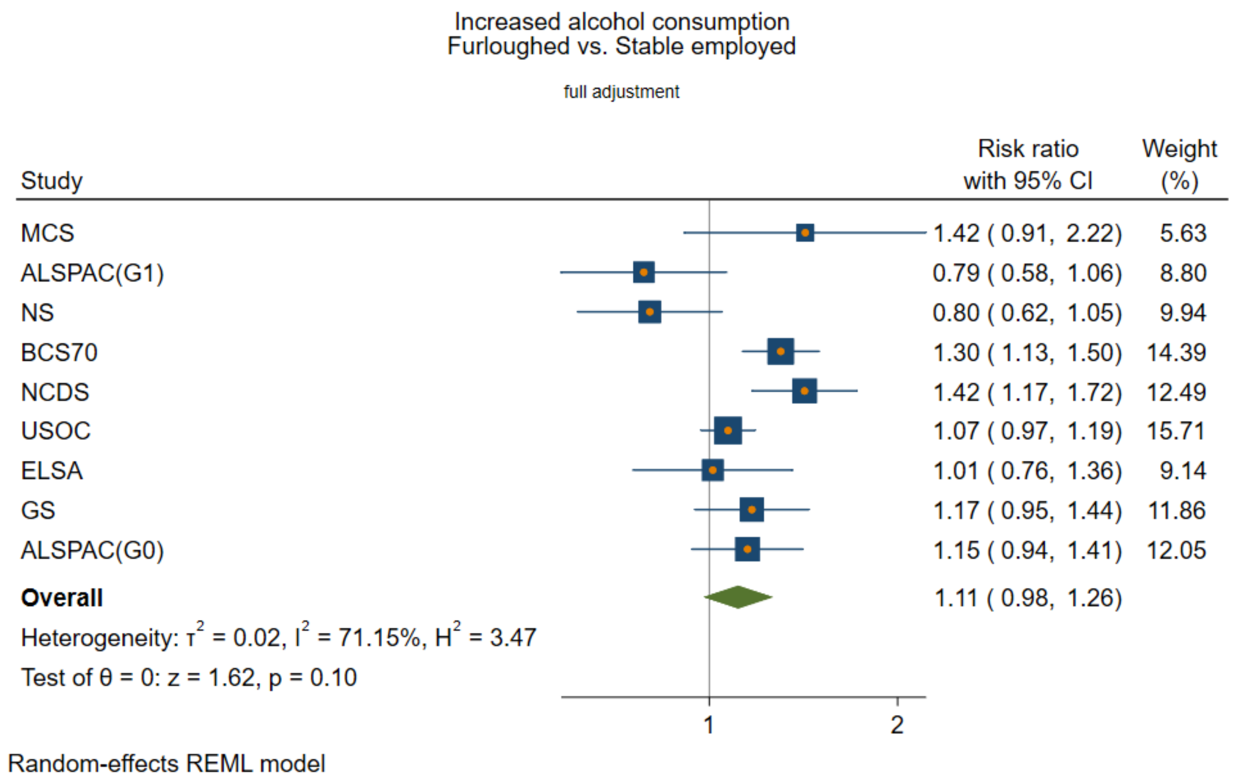
Increased alcohol consumption
Furloughed vs. Stable employed
full adjustment
Study
Risk ratio with 95% CI
Weight (%)
MCS 1.42 (0.91, 2.22) 5.63
ALSPAC(G1) 0.79 (0.58, 1.06) 8.80
NS 0.80 (0.62, 1.05) 9.94
BCS70 1.30 (1.13, 1.50) 14.39
NCDS 1.42 (1.17, 1.72) 12.49
USOC 1.07 (0.97, 1.19) 15.71
ELSA 1.01 (0.76, 1.36) 9.14
GS 1.17 (0.95, 1.44) 11.86
ALSPAC(G0) 1.15 (0.94, 1.41) 12.05
Overall 1.11 (0.98, 1.26)
Heterogeneity: τ² = 0.02, I² = 71.15%, H² = 3.47
Test of θ = 0: z = 1.62, p = 0.10
1
2
Random-effects REML model

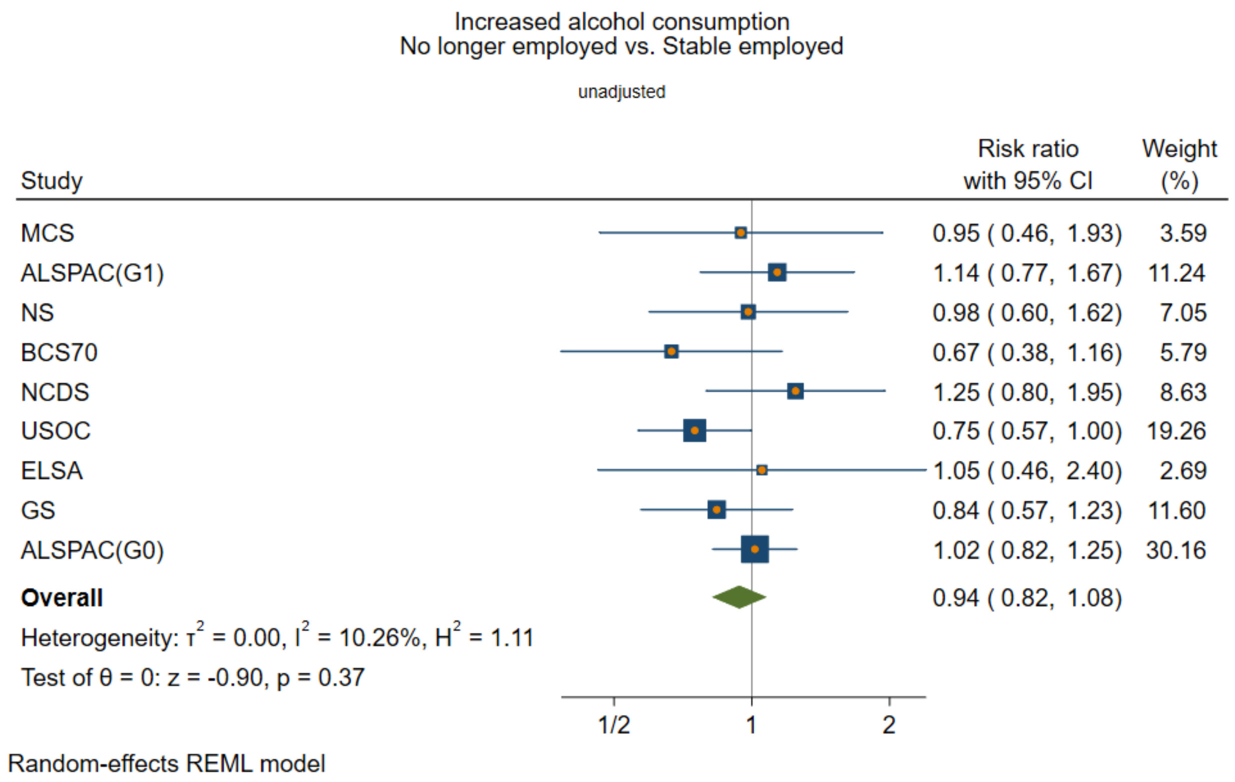
Increased alcohol consumption
No longer employed vs. Stable employed
unadjusted
Study
Risk ratio with 95% CI
Weight (%)
MCS 0.95 (0.46, 1.93) 3.59
ALSPAC(G1) 1.14 (0.77, 1.67) 11.24
NS 0.98 (0.60, 1.62) 7.05
BCS70 0.67 (0.38, 1.16) 5.79
NCDS 1.25 (0.80, 1.95) 8.63
USOC 0.75 (0.57, 1.00) 19.26
ELSA 1.05 (0.46, 2.40) 2.69
GS 0.84 (0.57, 1.23) 11.60
ALSPAC(G0) 1.02 (0.82, 1.25) 30.16
Overall 0.94 (0.82, 1.08)
Heterogeneity: τ² = 0.00, I² = 10.26%, H² = 1.11
Test of θ = 0: z = -0.90, p = 0.37
1/2
1
2
Random-effects REML model

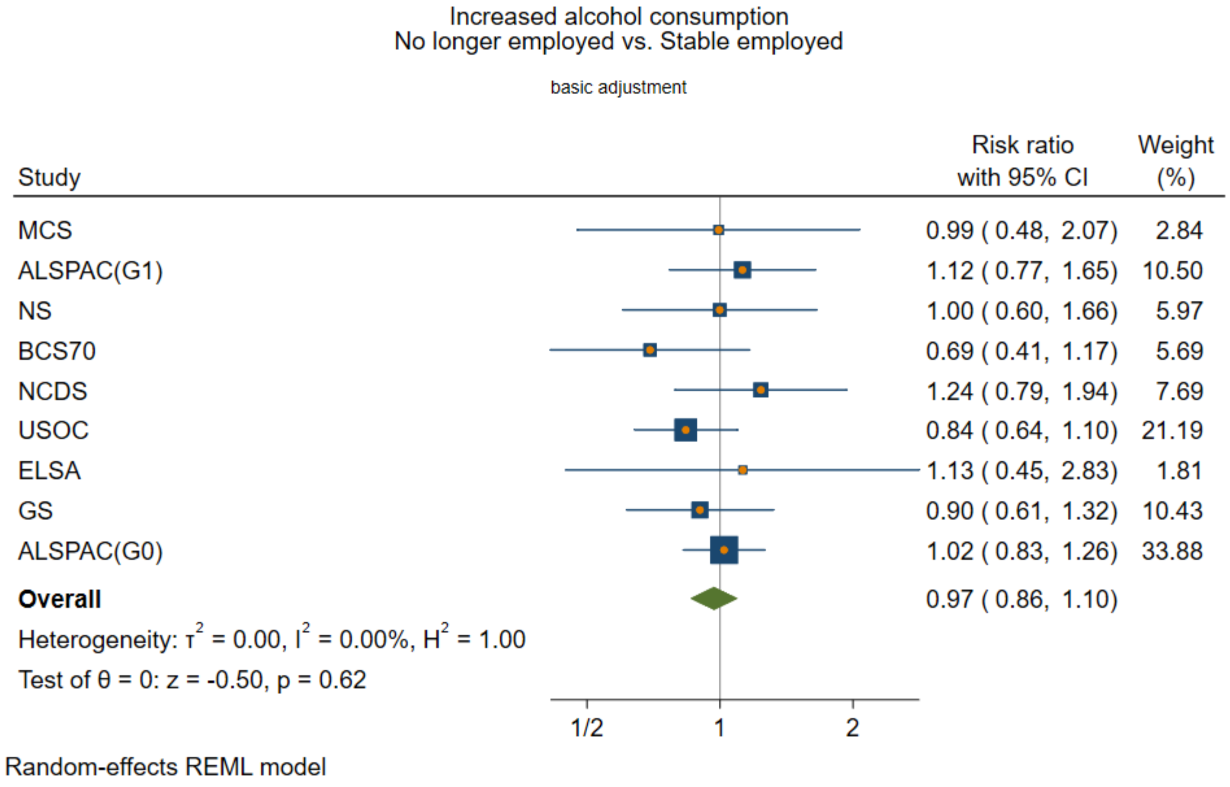
Increased alcohol consumption
No longer employed vs. Stable employed
basic adjustment
Study
Risk ratio with 95% CI
Weight (%)
MCS 0.99 (0.48, 2.07) 2.84
ALSPAC(G1) 1.12 (0.77, 1.65) 10.50
NS 1.00 (0.60, 1.66) 5.97
BCS70 0.69 (0.41, 1.17) 5.69
NCDS 1.24 (0.79, 1.94) 7.69
USOC 0.84 (0.64, 1.10) 21.19
ELSA 1.13 (0.45, 2.83) 1.81
GS 0.90 (0.61, 1.32) 10.43
ALSPAC(G0) 1.02 (0.83, 1.26) 33.88
Overall 0.97 (0.86, 1.10)
Heterogeneity: τ² = 0.00, I² = 0.00%, H² = 1.00
Test of θ = 0: z = -0.50, p = 0.62
1/2
1
2
Random-effects REML model

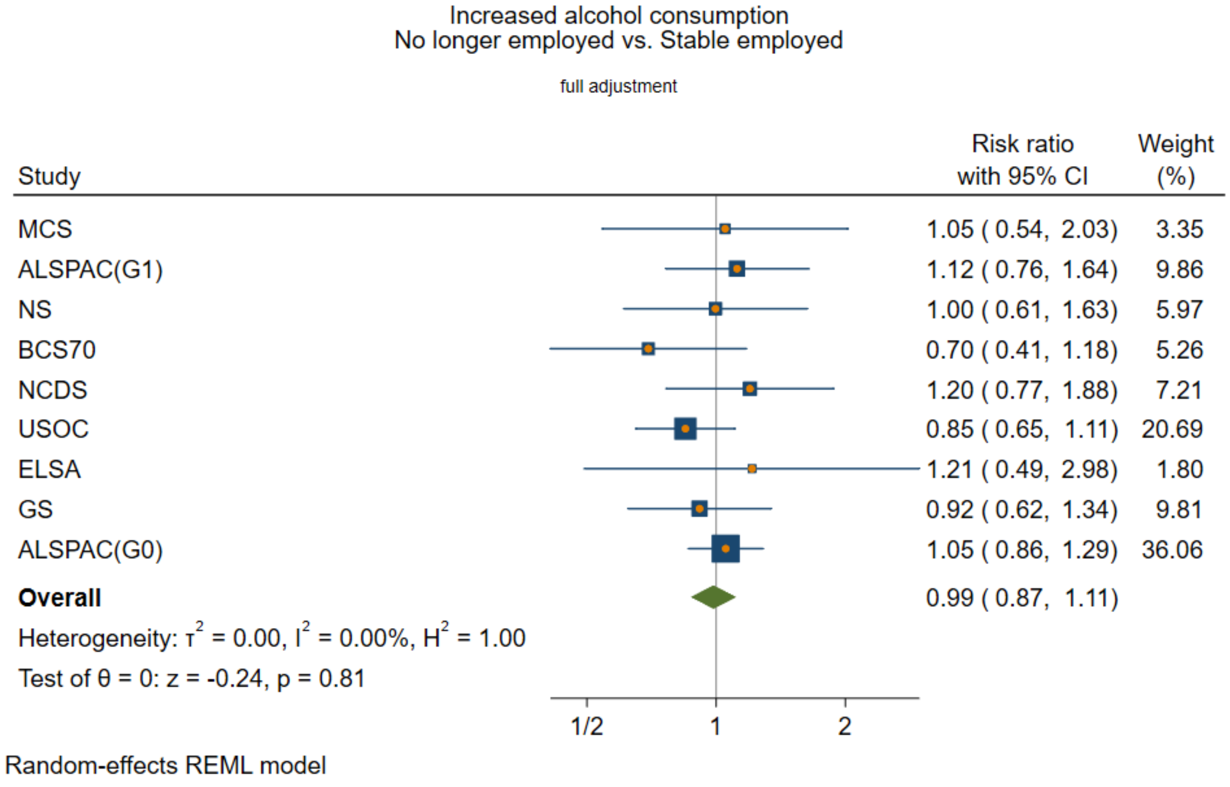
Increased alcohol consumption
No longer employed vs. Stable employed
full adjustment
Study
Risk ratio with 95% CI
Weight (%)
MCS 1.05 (0.54, 2.03) 3.35
ALSPAC(G1) 1.12 (0.76, 1.64) 9.86
NS 1.00 (0.61, 1.63) 5.97
BCS70 0.70 (0.41, 1.18) 5.26
NCDS 1.20 (0.77, 1.88) 7.21
USOC 0.85 (0.65, 1.11) 20.69
ELSA 1.21 (0.49, 2.98) 1.80
GS 0.92 (0.62, 1.34) 9.81
ALSPAC(G0) 1.05 (0.86, 1.29) 36.06
Overall 0.99 (0.87, 1.11)
Heterogeneity: τ² = 0.00, I² = 0.00%, H² = 1.00
Test of θ = 0: z = -0.24, p = 0.81
1/2
1
2
Random-effects REML model

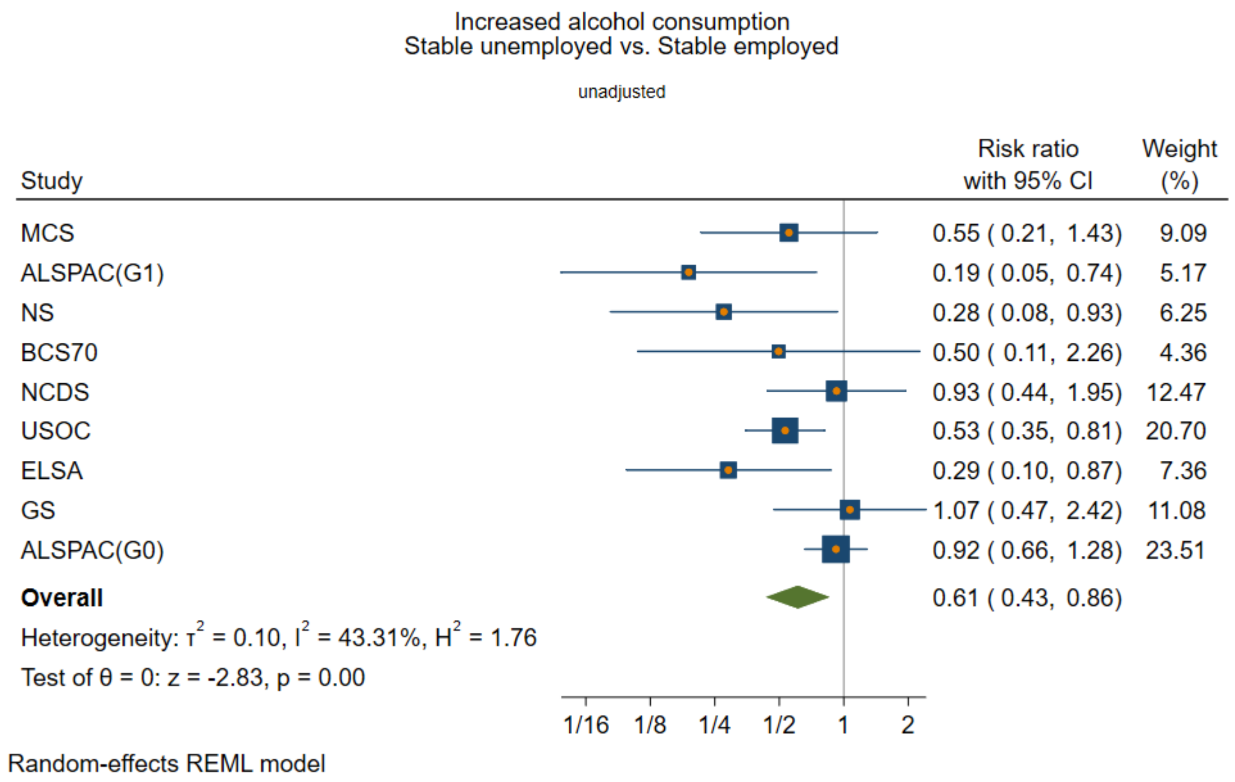

Increased alcohol consumption
Stable unemployed vs. Stable employed
unadjusted
Study
Risk ratio with 95% CI
Weight (%)
MCS 0.55 (0.21, 1.43) 9.09
ALSPAC(G1) 0.19 (0.05, 0.74) 5.17
NS 0.28 (0.08, 0.93) 6.25
BCS70 0.50 (0.11, 2.26) 4.36
NCDS 0.93 (0.44, 1.95) 12.47
USOC 0.53 (0.35, 0.81) 20.70
ELSA 0.29 (0.10, 0.87) 7.36
GS 1.07 (0.47, 2.42) 11.08
ALSPAC(G0) 0.92 (0.66, 1.28) 23.51
Overall 0.61 (0.43, 0.86)
Heterogeneity: τ² = 0.10, I² = 43.31%, H² = 1.76
Test of θ = 0: z = -2.83, p = 0.00
1/16
1/8
1/4
1/2
1
2
Random-effects REML model

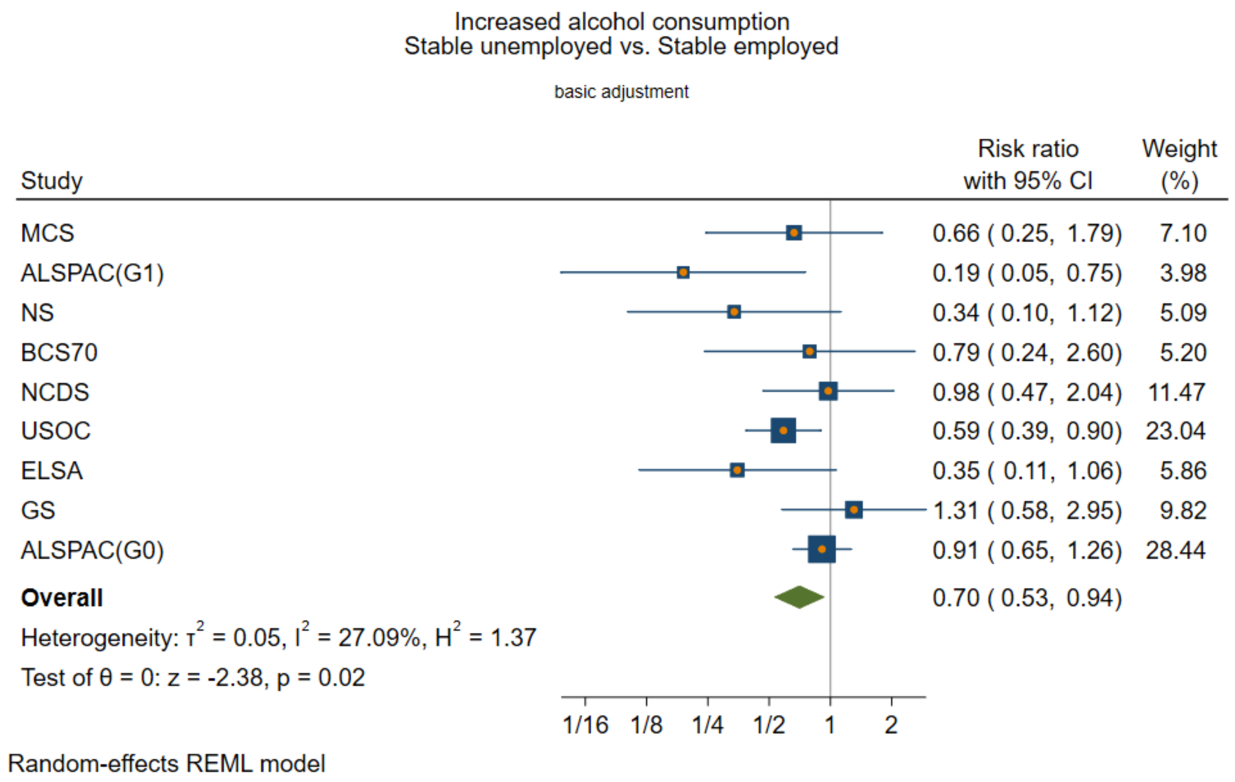

Increased alcohol consumption
Stable unemployed vs. Stable employed
basic adjustment
Study
Risk ratio with 95% CI
Weight (%)
MCS 0.66 (0.25, 1.79) 7.10
ALSPAC(G1) 0.19 (0.05, 0.75) 3.98
NS 0.34 (0.10, 1.12) 5.09
BCS70 0.79 (0.24, 2.60) 5.20
NCDS 0.98 (0.47, 2.04) 11.47
USOC 0.59 (0.39, 0.90) 23.04
ELSA 0.35 (0.11, 1.06) 5.86
GS 1.31 (0.58, 2.95) 9.82
ALSPAC(G0) 0.91 (0.65, 1.26) 28.44
Overall 0.70 (0.53, 0.94)
Heterogeneity: τ² = 0.05, I² = 27.09%, H² = 1.37
Test of θ = 0: z = -2.38, p = 0.02
1/16
1/8
1/4
1/2
1
2
Random-effects REML model

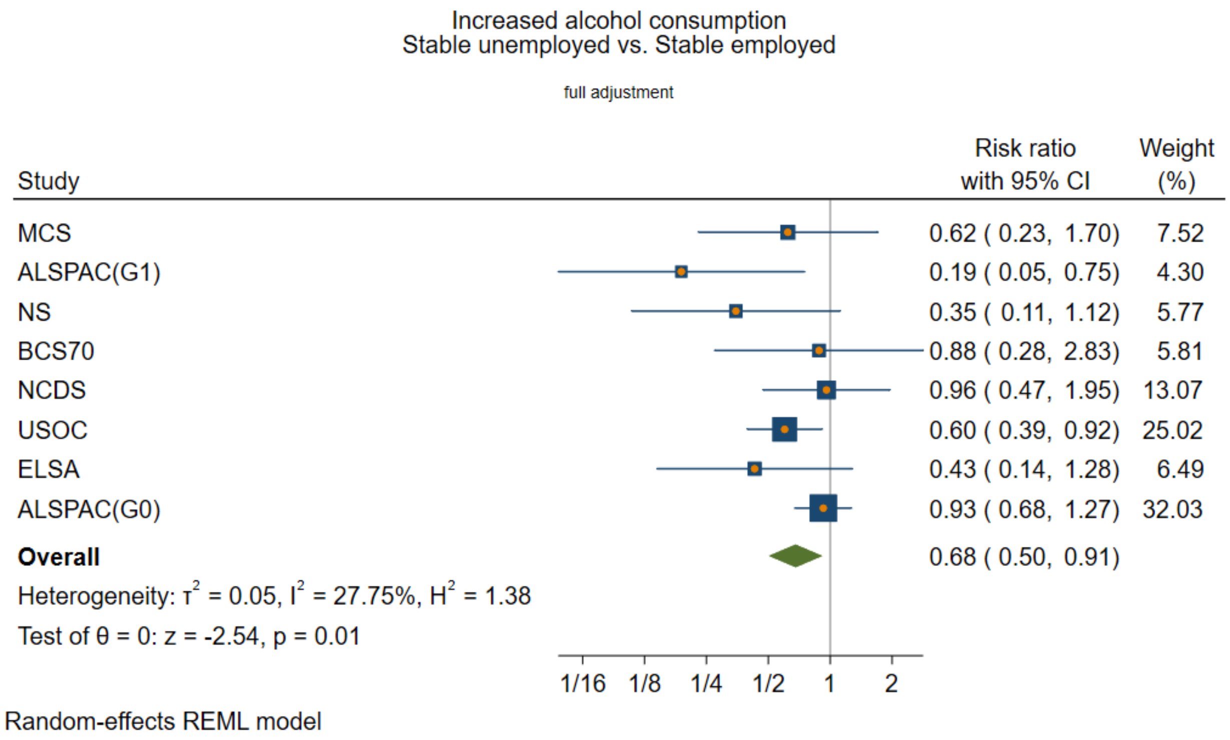
Increased alcohol consumption
Stable unemployed vs. Stable employed
full adjustment
Study
Risk ratio with 95% CI
Weight (%)
MCS 0.62 (0.23, 1.70) 7.52
ALSPAC(G1) 0.19 (0.05, 0.75) 4.30
NS 0.35 (0.11, 1.12) 5.77
BCS70 0.88 (0.28, 2.83) 5.81
NCDS 0.96 (0.47, 1.95) 13.07
USOC 0.60 (0.39, 0.92) 25.02
ELSA 0.43 (0.14, 1.28) 6.49
ALSPAC(G0) 0.93 (0.68, 1.27) 32.03
Overall 0.68 (0.50, 0.91)
Heterogeneity: τ² = 0.05, I² = 27.75%, H² = 1.38
Test of θ = 0: z = -2.54, p = 0.01
1/16 1/8 1/4 1/2 1 2
Random-effects REML model

Figure set 3. Reduced alcohol consumption

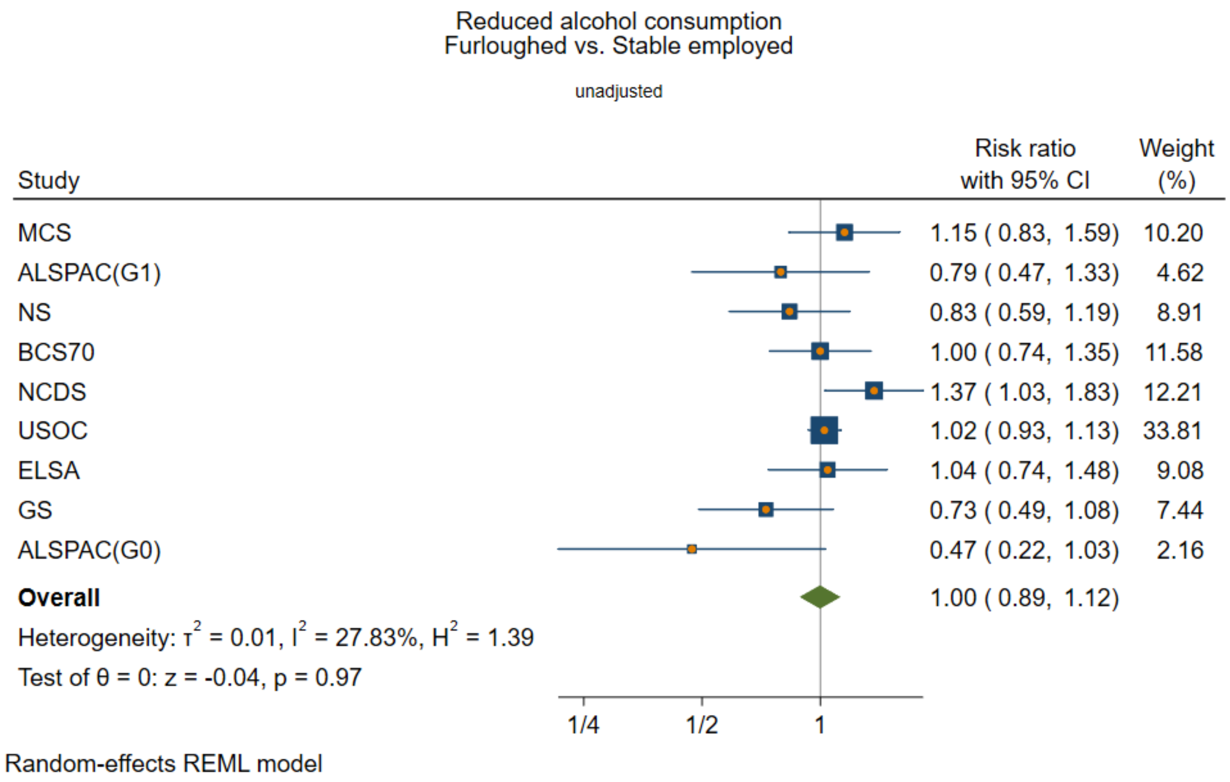

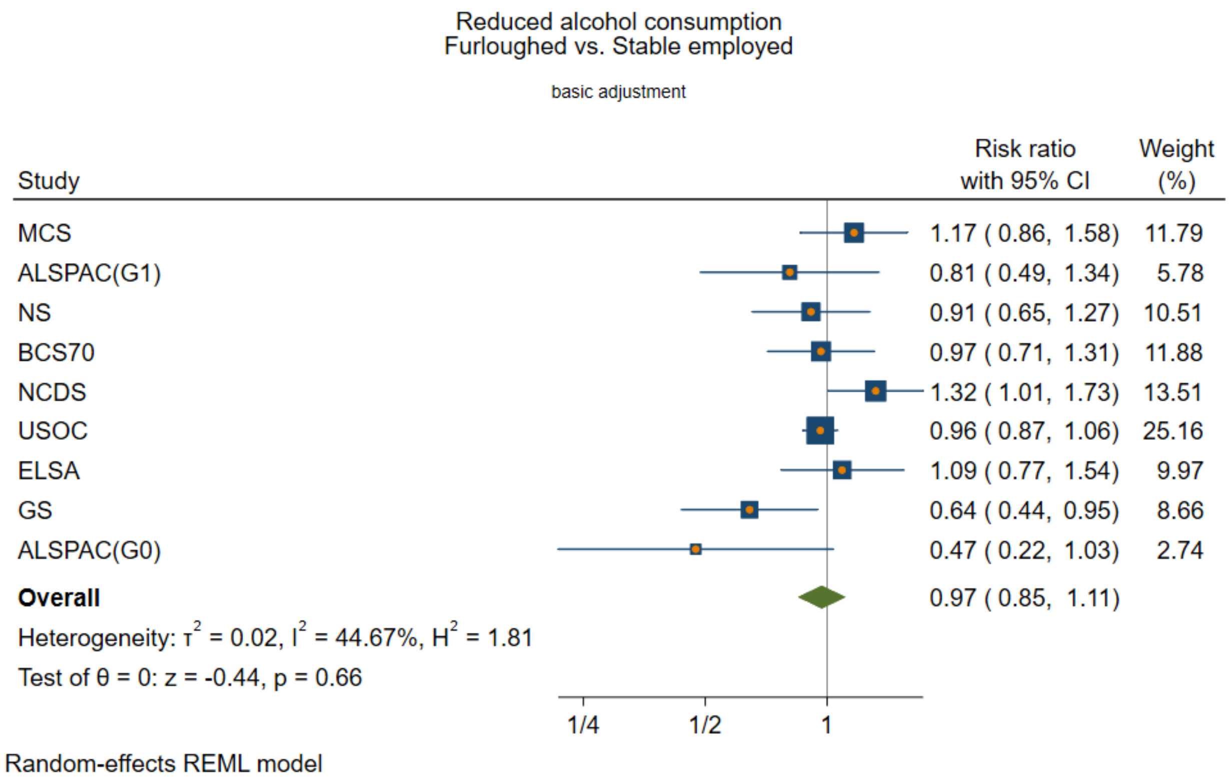

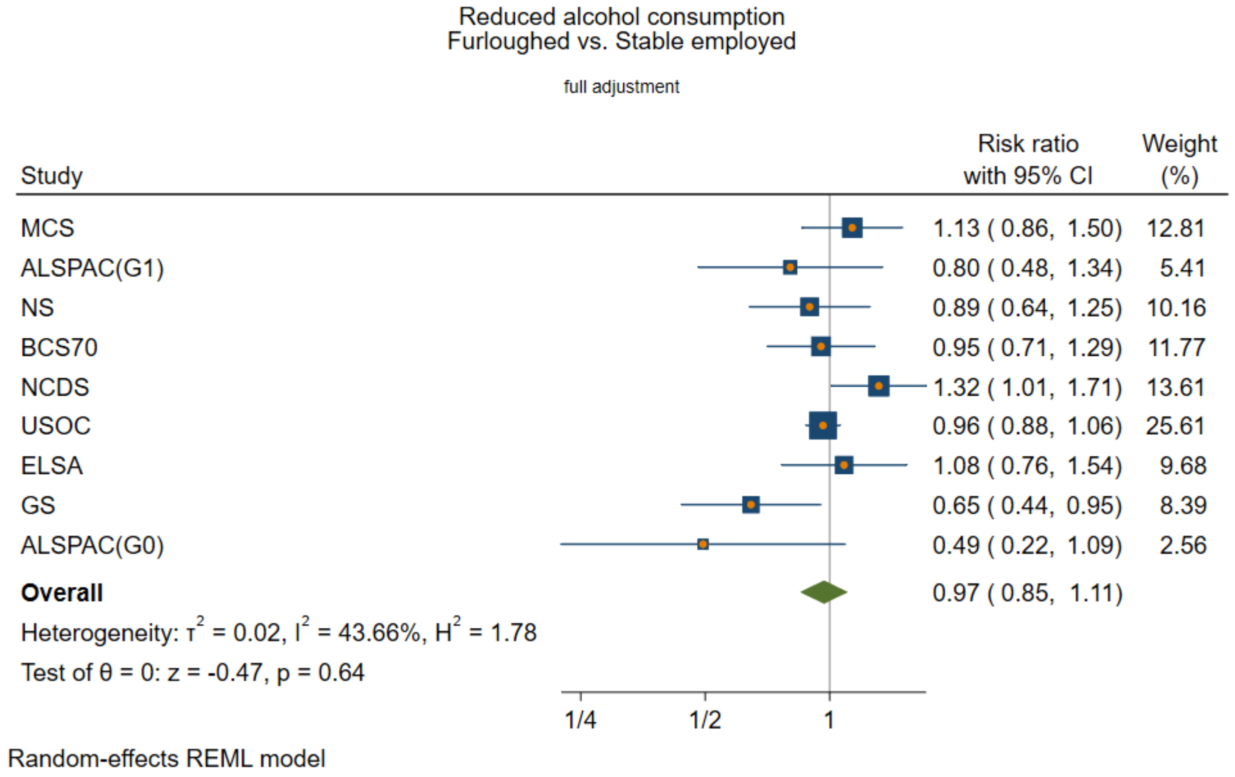
Reduced alcohol consumption
Furloughed vs. Stable employed
full adjustment
Study
Risk ratio with 95% CI
Weight (%)
MCS 1.13 (0.86, 1.50) 12.81
ALSPAC(G1) 0.80 (0.48, 1.34) 5.41
NS 0.89 (0.64, 1.25) 10.16
BCS70 0.95 (0.71, 1.29) 11.77
NCDS 1.32 (1.01, 1.71) 13.61
USOC 0.96 (0.88, 1.06) 25.61
ELSA 1.08 (0.76, 1.54) 9.68
GS 0.65 (0.44, 0.95) 8.39
ALSPAC(G0) 0.49 (0.22, 1.09) 2.56
Overall 0.97 (0.85, 1.11)
Heterogeneity: τ2 = 0.02, I2 = 43.66%, H2 = 1.78
Test of θ = 0: z = -0.47, p = 0.64
1/4
1/2
1
Random-effects REML model

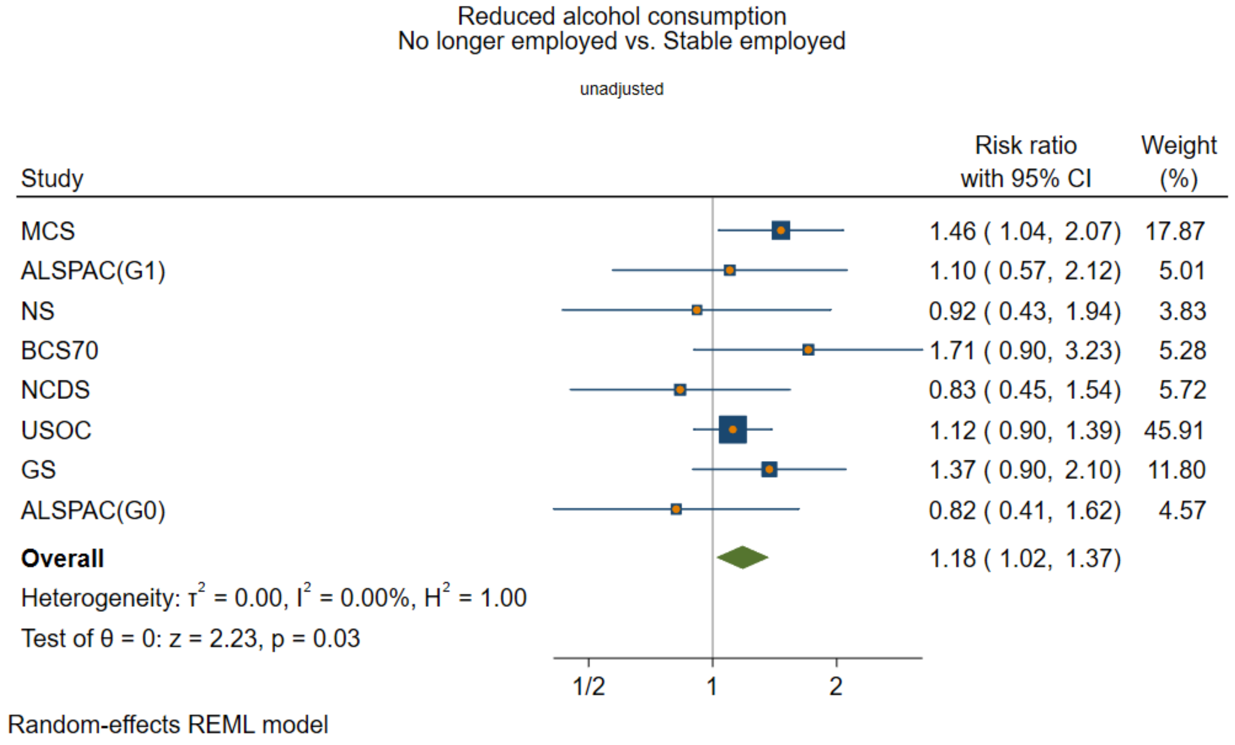
Reduced alcohol consumption
No longer employed vs. Stable employed
unadjusted
Study
Risk ratio with 95% CI
Weight (%)
MCS 1.46 (1.04, 2.07) 17.87
ALSPAC(G1) 1.10 (0.57, 2.12) 5.01
NS 0.92 (0.43, 1.94) 3.83
BCS70 1.71 (0.90, 3.23) 5.28
NCDS 0.83 (0.45, 1.54) 5.72
USOC 1.12 (0.90, 1.39) 45.91
GS 1.37 (0.90, 2.10) 11.80
ALSPAC(G0) 0.82 (0.41, 1.62) 4.57
Overall 1.18 (1.02, 1.37)
Heterogeneity: τ2 = 0.00, I2 = 0.00%, H2 = 1.00
Test of θ = 0: z = 2.23, p = 0.03
1/2
1
2
Random-effects REML model

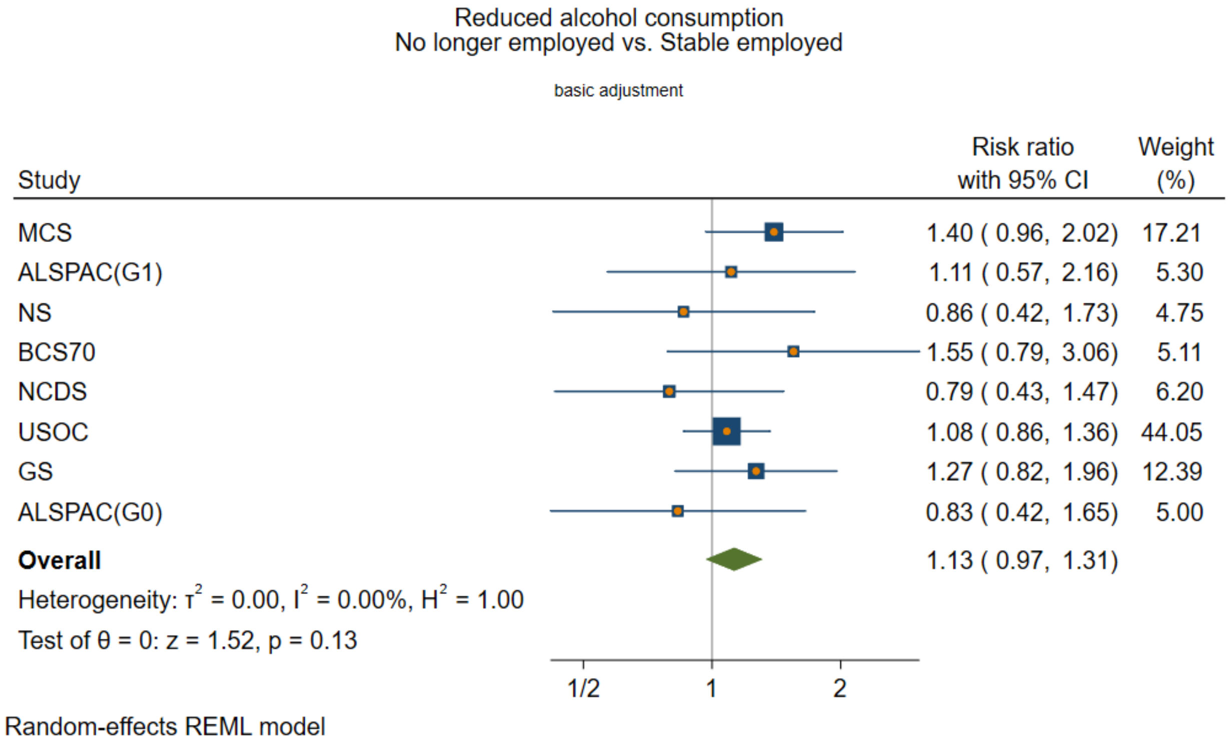
Reduced alcohol consumption
No longer employed vs. Stable employed
basic adjustment
Study
Risk ratio with 95% CI
Weight (%)
MCS 1.40 (0.96, 2.02) 17.21
ALSPAC(G1) 1.11 (0.57, 2.16) 5.30
NS 0.86 (0.42, 1.73) 4.75
BCS70 1.55 (0.79, 3.06) 5.11
NCDS 0.79 (0.43, 1.47) 6.20
USOC 1.08 (0.86, 1.36) 44.05
GS 1.27 (0.82, 1.96) 12.39
ALSPAC(G0) 0.83 (0.42, 1.65) 5.00
Overall 1.13 (0.97, 1.31)
Heterogeneity: τ² = 0.00, I² = 0.00%, H² = 1.00
Test of θ = 0: z = 1.52, p = 0.13
1/2
1
2
Random-effects REML model

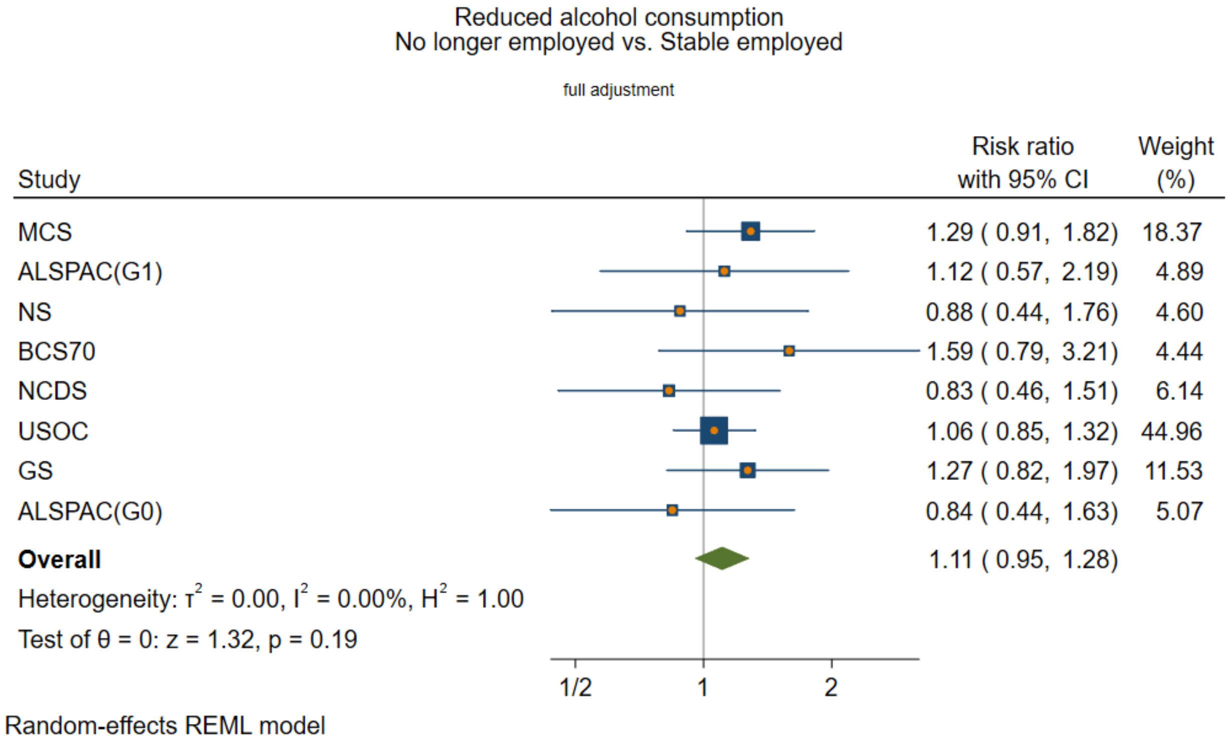
Reduced alcohol consumption
No longer employed vs. Stable employed
full adjustment
Study
Risk ratio with 95% CI
Weight (%)
MCS 1.29 (0.91, 1.82) 18.37
ALSPAC(G1) 1.12 (0.57, 2.19) 4.89
NS 0.88 (0.44, 1.76) 4.60
BCS70 1.59 (0.79, 3.21) 4.44
NCDS 0.83 (0.46, 1.51) 6.14
USOC 1.06 (0.85, 1.32) 44.96
GS 1.27 (0.82, 1.97) 11.53
ALSPAC(G0) 0.84 (0.44, 1.63) 5.07
Overall 1.11 (0.95, 1.28)
Heterogeneity: τ² = 0.00, I² = 0.00%, H² = 1.00
Test of θ = 0: z = 1.32, p = 0.19
1/2
1
2
Random-effects REML model

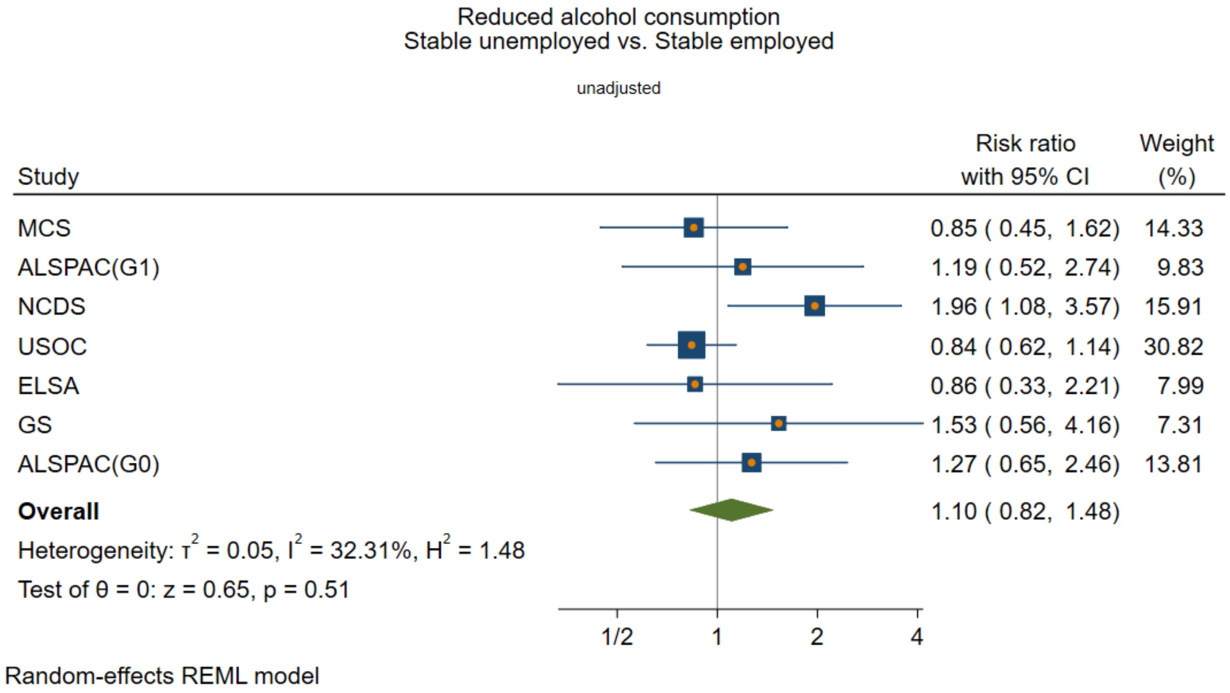
Reduced alcohol consumption
Stable unemployed vs. Stable employed
unadjusted
Study
Risk ratio with 95% CI
Weight (%)
MCS 0.85 (0.45, 1.62) 14.33
ALSPAC(G1) 1.19 (0.52, 2.74) 9.83
NCDS 1.96 (1.08, 3.57) 15.91
USOC 0.84 (0.62, 1.14) 30.82
ELSA 0.86 (0.33, 2.21) 7.99
GS 1.53 (0.56, 4.16) 7.31
ALSPAC(G0) 1.27 (0.65, 2.46) 13.81
Overall 1.10 (0.82, 1.48)
Heterogeneity: τ² = 0.05, I² = 32.31%, H² = 1.48
Test of θ = 0: z = 0.65, p = 0.51
1/2
1
2
4
Random-effects REML model

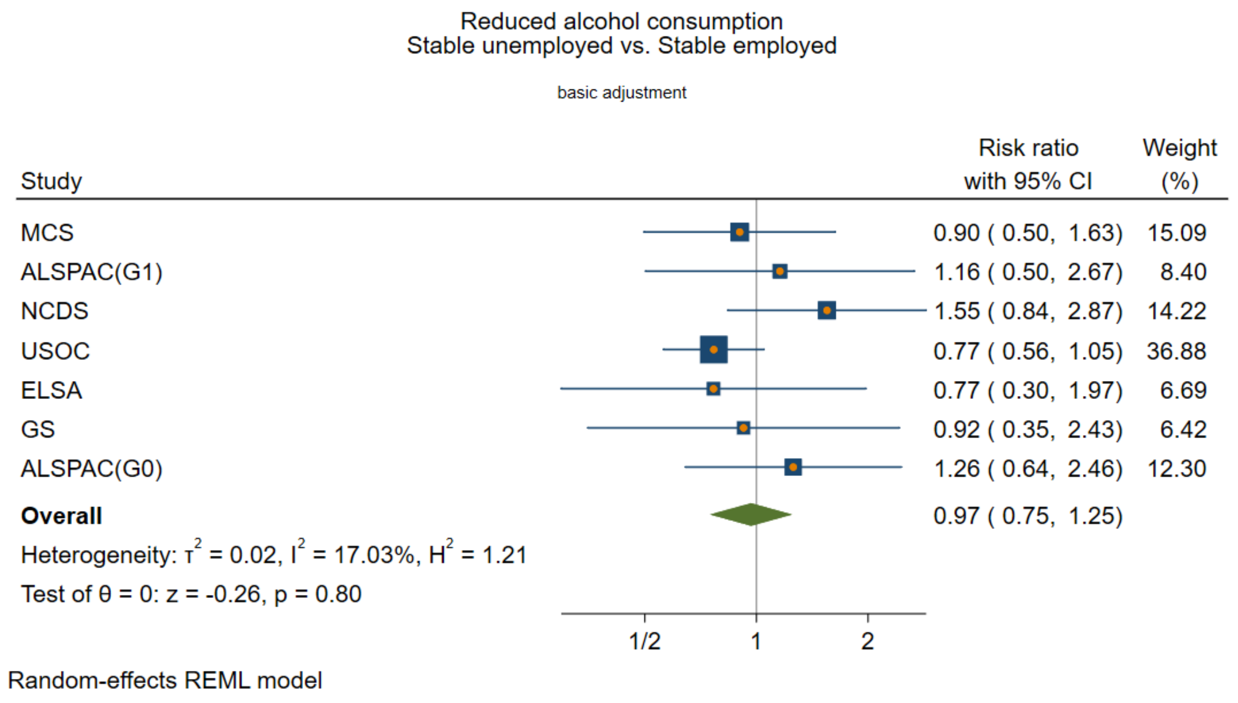
Reduced alcohol consumption
Stable unemployed vs. Stable employed
basic adjustment
Study
Risk ratio with 95% CI
Weight (%)
MCS 0.90 (0.50, 1.63) 15.09
ALSPAC(G1) 1.16 (0.50, 2.67) 8.40
NCDS 1.55 (0.84, 2.87) 14.22
USOC 0.77 (0.56, 1.05) 36.88
ELSA 0.77 (0.30, 1.97) 6.69
GS 0.92 (0.35, 2.43) 6.42
ALSPAC(G0) 1.26 (0.64, 2.46) 12.30
Overall 0.97 (0.75, 1.25)
Heterogeneity: τ² = 0.02, I² = 17.03%, H² = 1.21
Test of θ = 0: z = -0.26, p = 0.80
1/2
1
2
Random-effects REML model

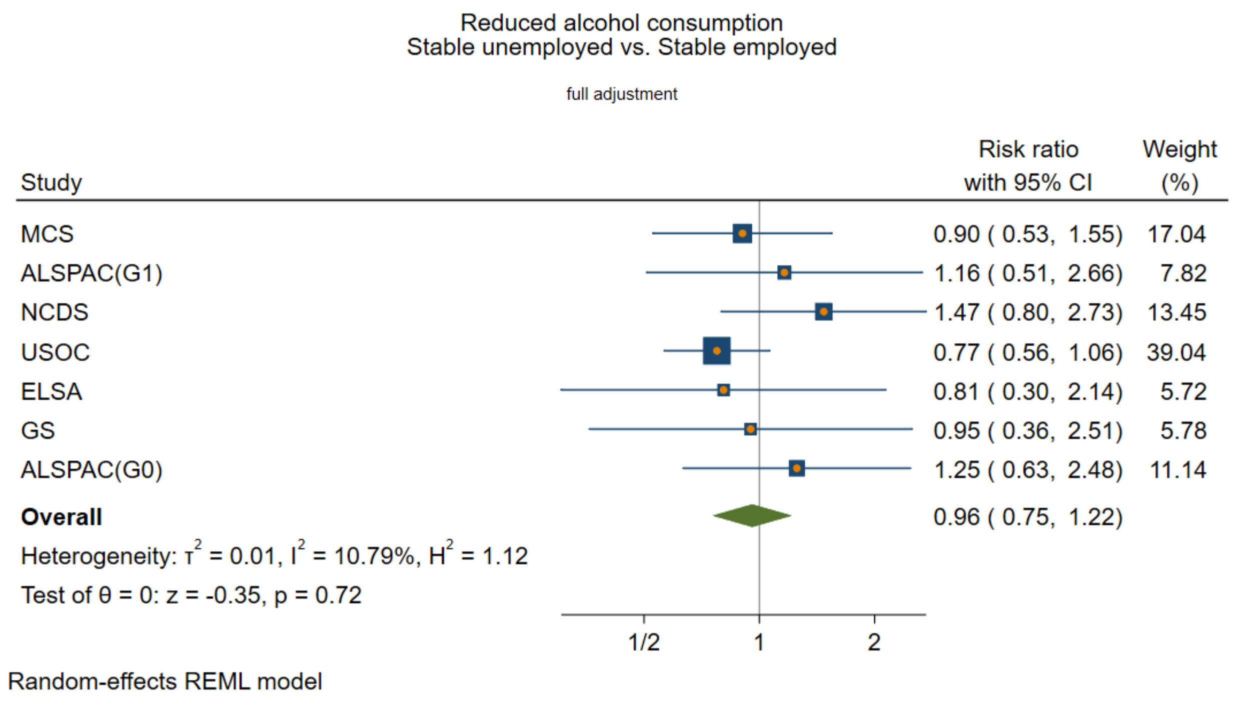
Reduced alcohol consumption
Stable unemployed vs. Stable employed
full adjustment
Study
Risk ratio with 95% CI
Weight (%)
MCS 0.90 (0.53, 1.55) 17.04
ALSPAC(G1) 1.16 (0.51, 2.66) 7.82
NCDS 1.47 (0.80, 2.73) 13.45
USOC 0.77 (0.56, 1.06) 39.04
ELSA 0.81 (0.30, 2.14) 5.72
GS 0.95 (0.36, 2.51) 5.78
ALSPAC(G0) 1.25 (0.63, 2.48) 11.14
Overall 0.96 (0.75, 1.22)
Heterogeneity: τ² = 0.01, I² = 10.79%, H² = 1.12
Test of θ = 0: z = -0.35, p = 0.72
1/2
1
2
Random-effects REML model

Figure set 4. Currently drinks 5+ drinks/occasion

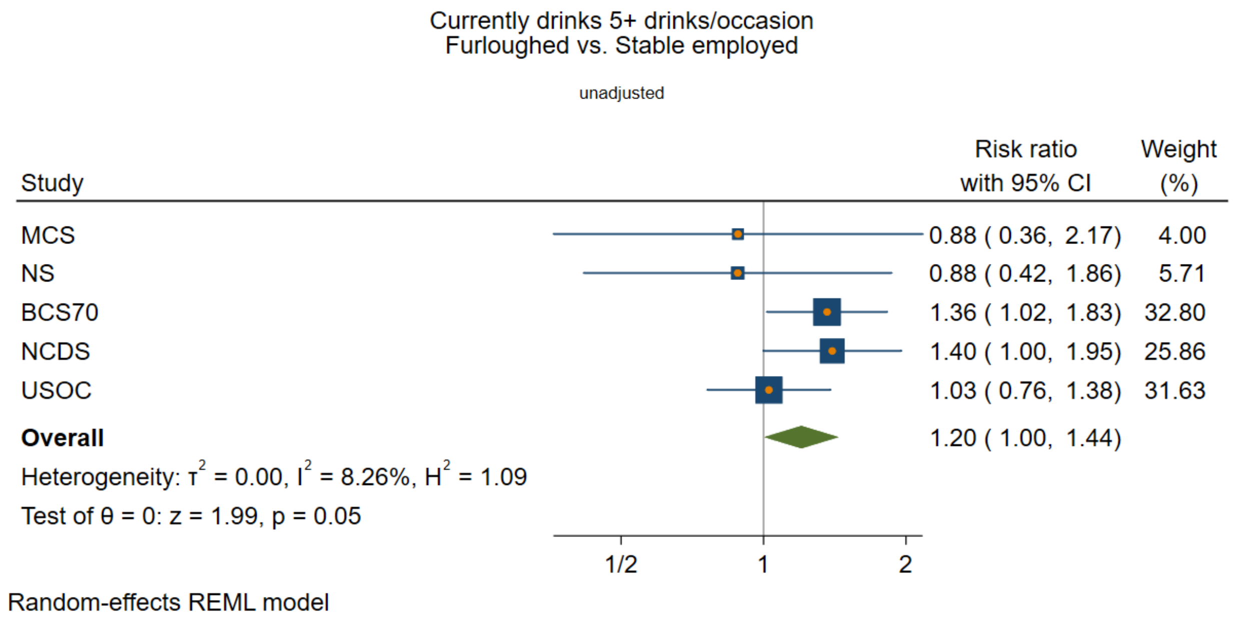

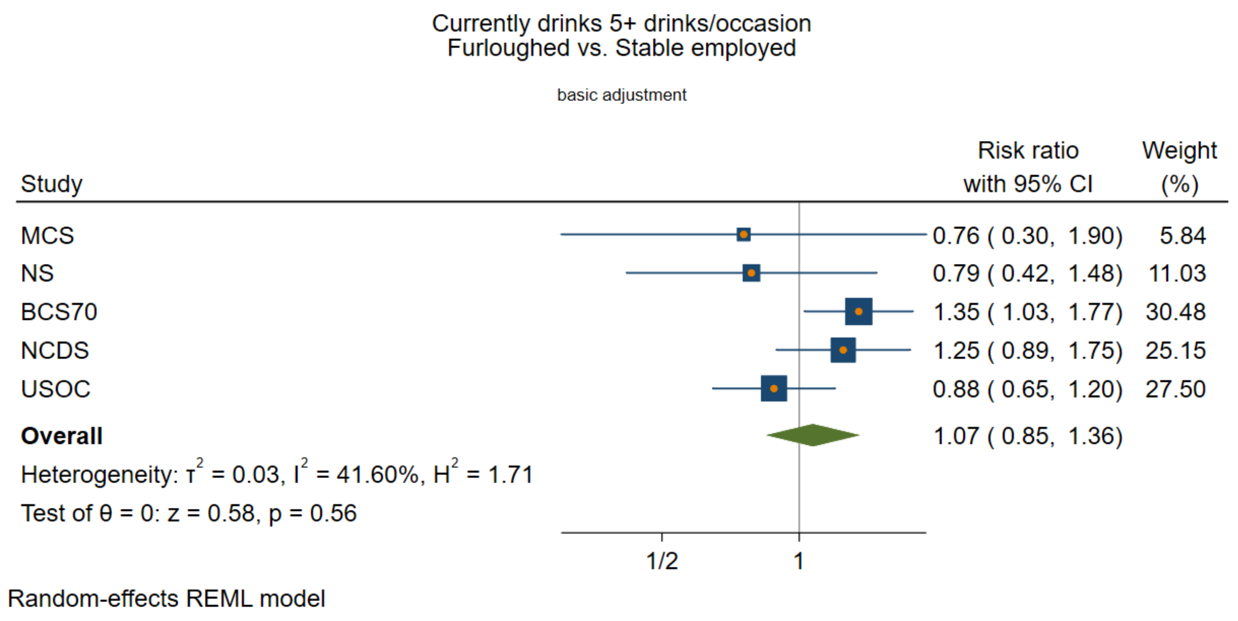

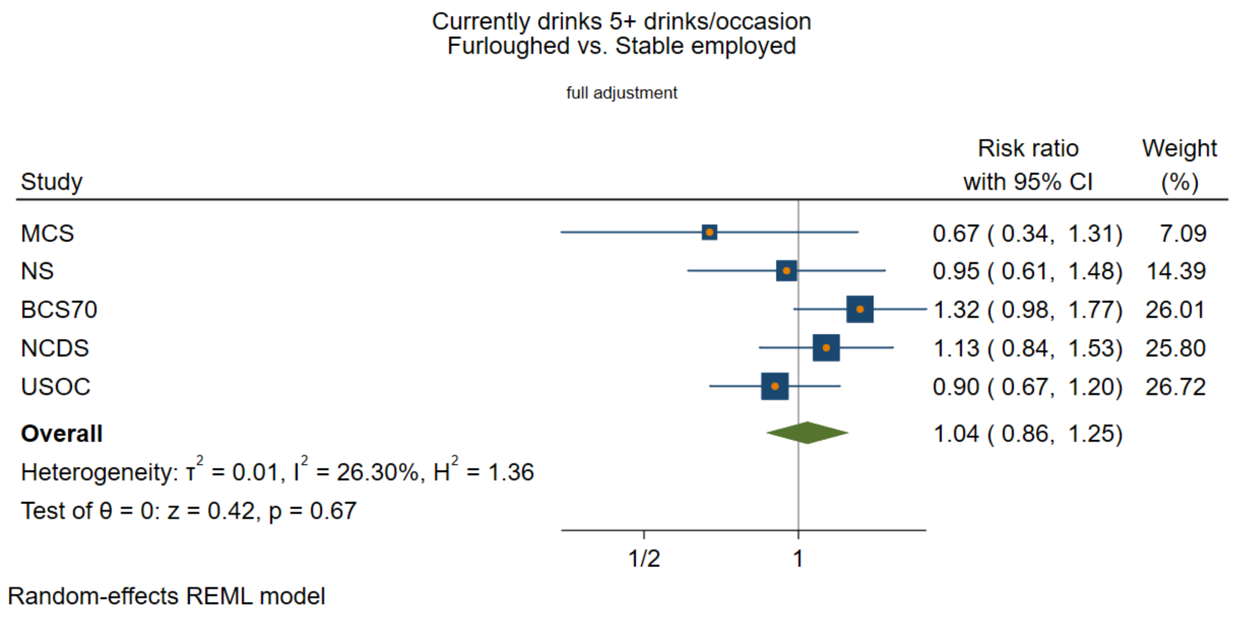

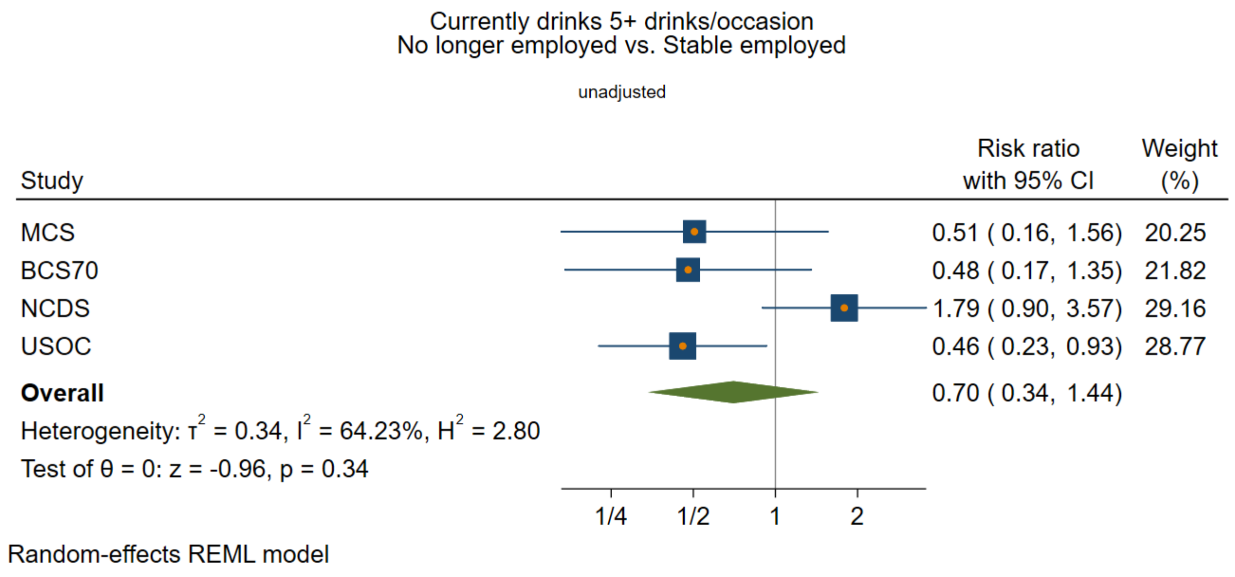
Currently drinks 5+ drinks/occasion
No longer employed vs. Stable employed
unadjusted
Study
Risk ratio with 95% CI
Weight (%)
MCS 0.51 (0.16, 1.56) 20.25
BCS70 0.48 (0.17, 1.35) 21.82
NCDS 1.79 (0.90, 3.57) 29.16
USOC 0.46 (0.23, 0.93) 28.77
Overall 0.70 (0.34, 1.44)
Heterogeneity: τ² = 0.34, I² = 64.23%, H² = 2.80
Test of θ = 0: z = -0.96, p = 0.34
1/4 1/2 1 2
Random-effects REML model

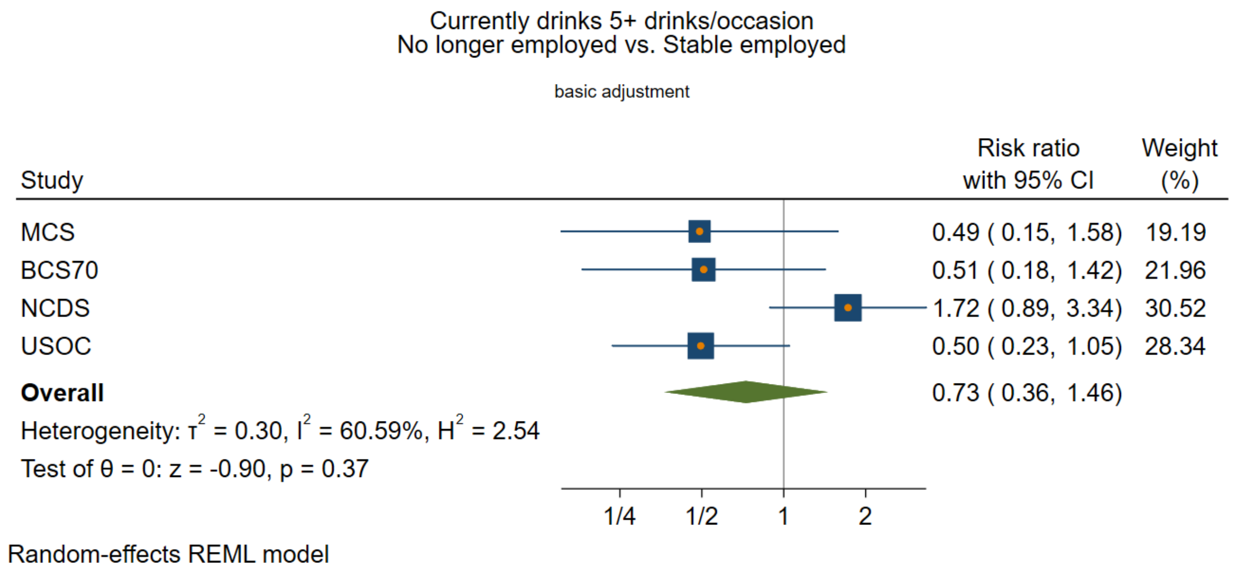
Currently drinks 5+ drinks/occasion
No longer employed vs. Stable employed
basic adjustment
Study
Risk ratio with 95% CI
Weight (%)
MCS 0.49 (0.15, 1.58) 19.19
BCS70 0.51 (0.18, 1.42) 21.96
NCDS 1.72 (0.89, 3.34) 30.52
USOC 0.50 (0.23, 1.05) 28.34
Overall 0.73 (0.36, 1.46)
Heterogeneity: τ² = 0.30, I² = 60.59%, H² = 2.54
Test of θ = 0: z = -0.90, p = 0.37
1/4 1/2 1 2
Random-effects REML model

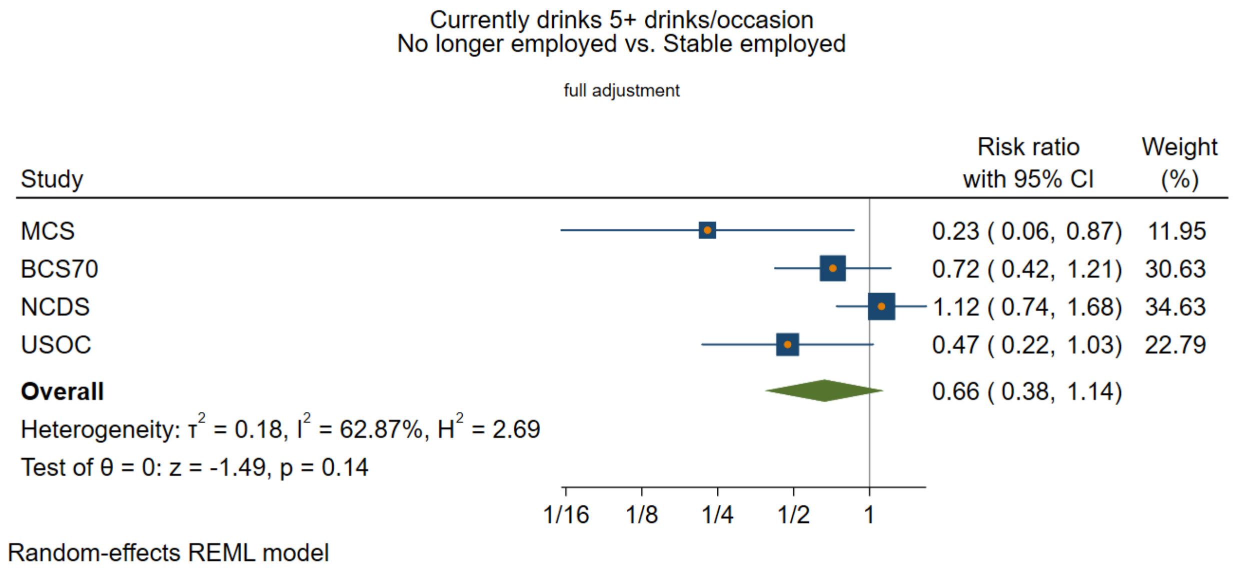
Currently drinks 5+ drinks/occasion
No longer employed vs. Stable employed
full adjustment
Study
Risk ratio with 95% CI
Weight (%)
MCS 0.23 (0.06, 0.87) 11.95
BCS70 0.72 (0.42, 1.21) 30.63
NCDS 1.12 (0.74, 1.68) 34.63
USOC 0.47 (0.22, 1.03) 22.79
Overall 0.66 (0.38, 1.14)
Heterogeneity: τ² = 0.18, I² = 62.87%, H² = 2.69
Test of θ = 0: z = -1.49, p = 0.14
1/16 1/8 1/4 1/2 1
Random-effects REML model

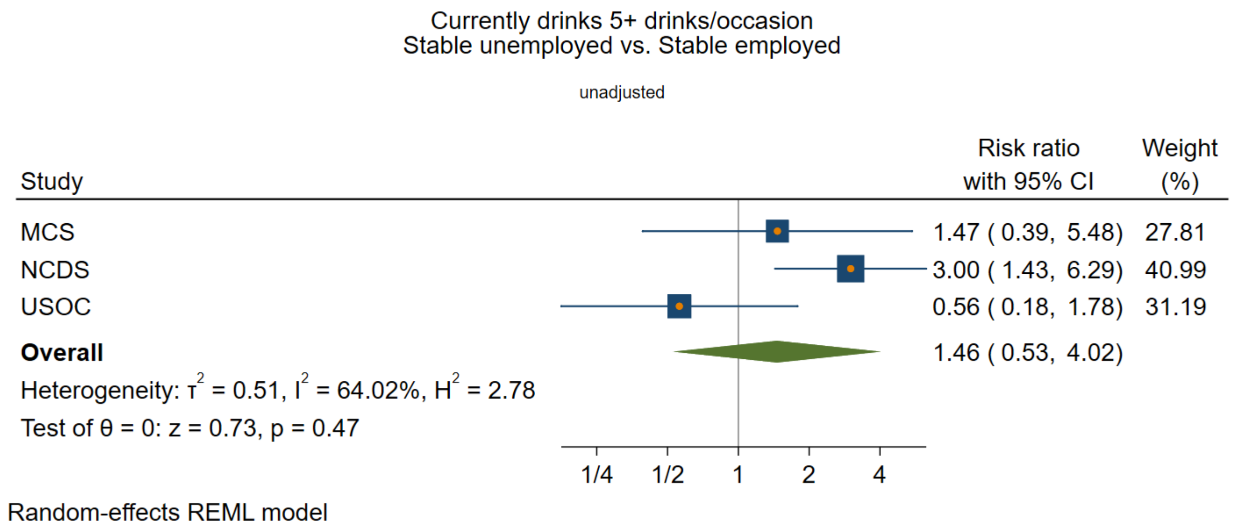
Currently drinks 5+ drinks/occasion
Stable unemployed vs. Stable employed
unadjusted
Study
Risk ratio with 95% CI
Weight (%)
MCS 1.47 (0.39, 5.48) 27.81
NCDS 3.00 (1.43, 6.29) 40.99
USOC 0.56 (0.18, 1.78) 31.19
Overall 1.46 (0.53, 4.02)
Heterogeneity: τ² = 0.51, I² = 64.02%, H² = 2.78
Test of θ = 0: z = 0.73, p = 0.47
1/4 1/2 1 2 4
Random-effects REML model

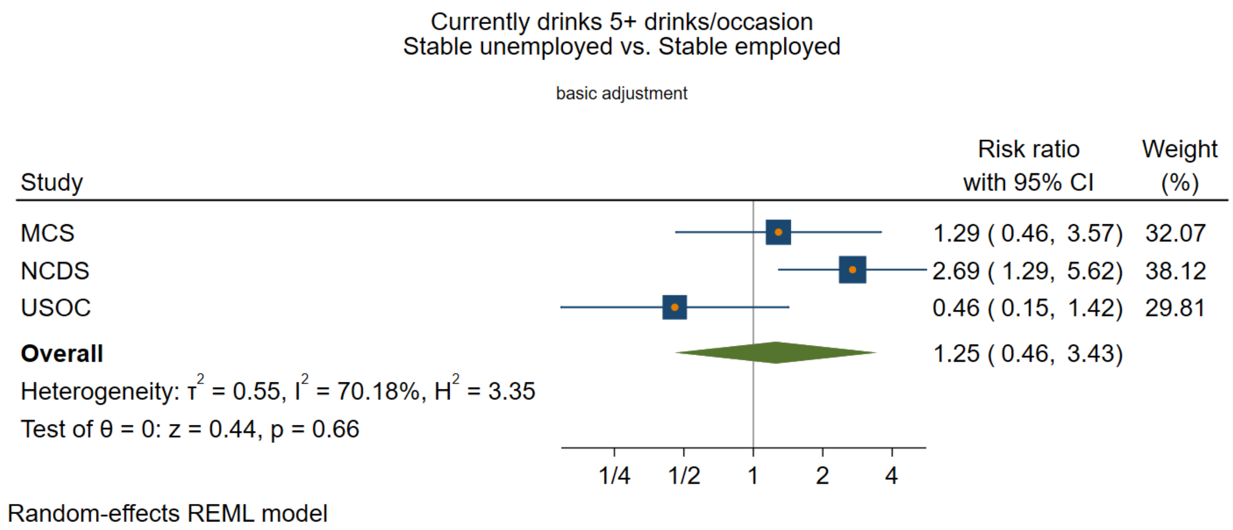
Currently drinks 5+ drinks/occasion
Stable unemployed vs. Stable employed
basic adjustment
Study
Risk ratio with 95% CI
Weight (%)
MCS 1.29 (0.46, 3.57) 32.07
NCDS 2.69 (1.29, 5.62) 38.12
USOC 0.46 (0.15, 1.42) 29.81
Overall 1.25 (0.46, 3.43)
Heterogeneity: τ² = 0.55, I² = 70.18%, H² = 3.35
Test of θ = 0: z = 0.44, p = 0.66
1/4 1/2 1 2 4
Random-effects REML model

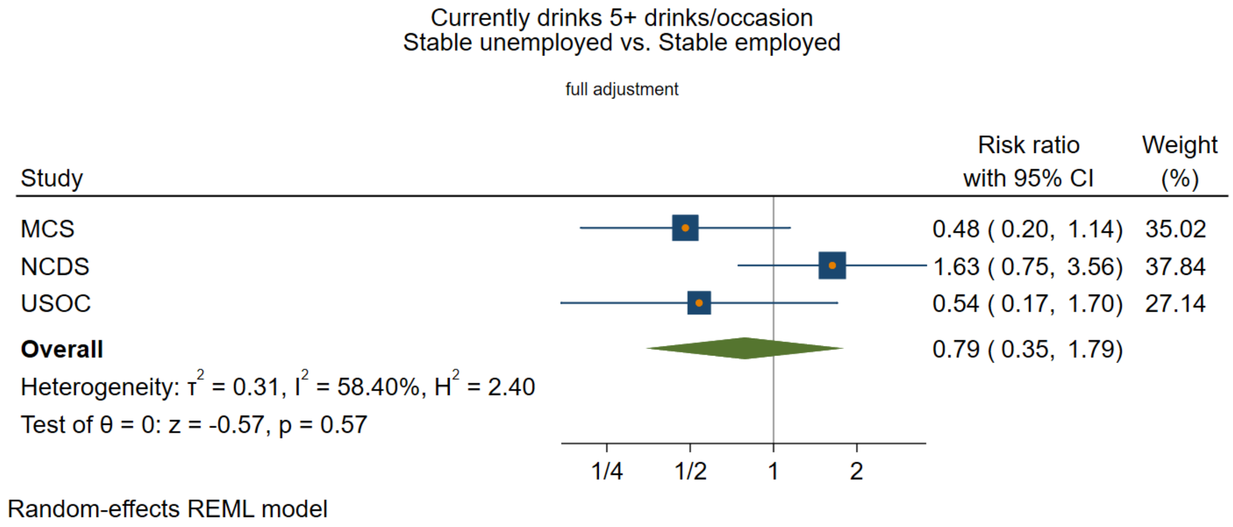
Currently drinks 5+ drinks/occasion
Stable unemployed vs. Stable employed
full adjustment
Study
Risk ratio with 95% CI
Weight (%)
MCS 0.48 (0.20, 1.14) 35.02
NCDS 1.63 (0.75, 3.56) 37.84
USOC 0.54 (0.17, 1.70) 27.14
Overall 0.79 (0.35, 1.79)
Heterogeneity: τ² = 0.31, I² = 58.40%, H² = 2.40
Test of θ = 0: z = -0.57, p = 0.57
1/4 1/2 1 2
Random-effects REML model

Figure set 5. Drinks more alcohol units per occasion

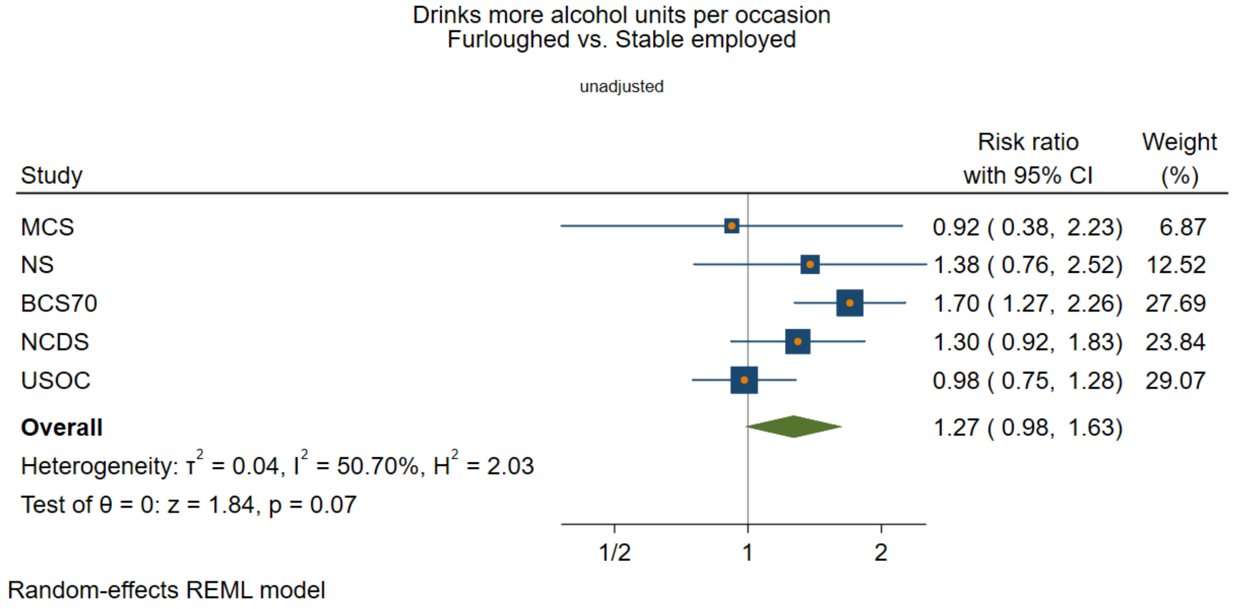

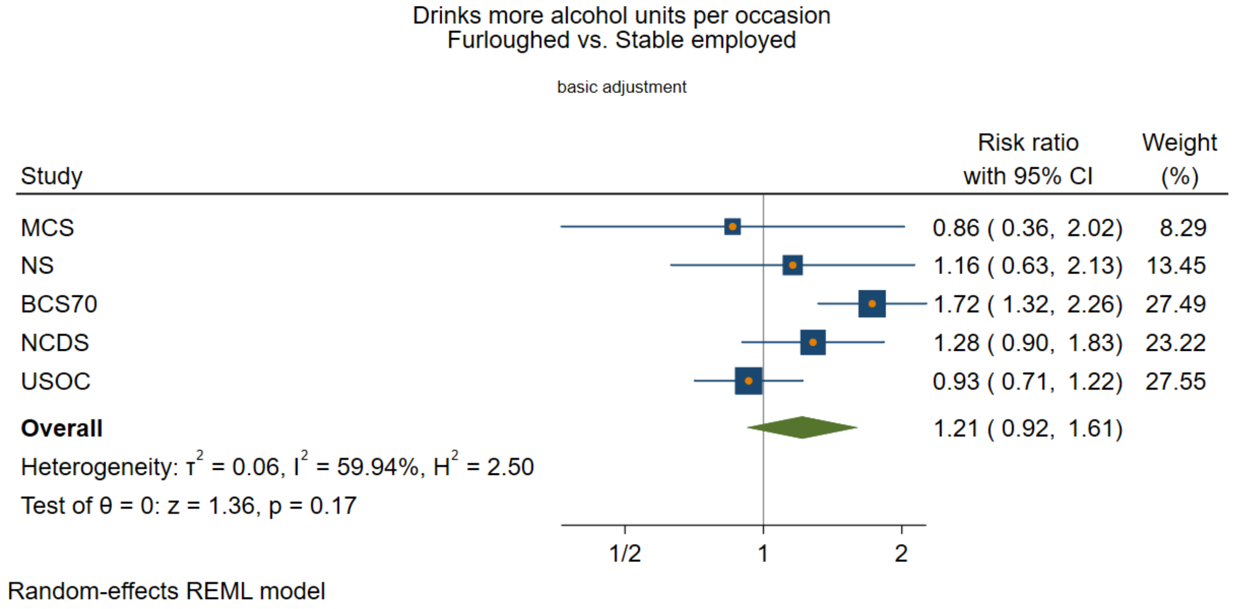

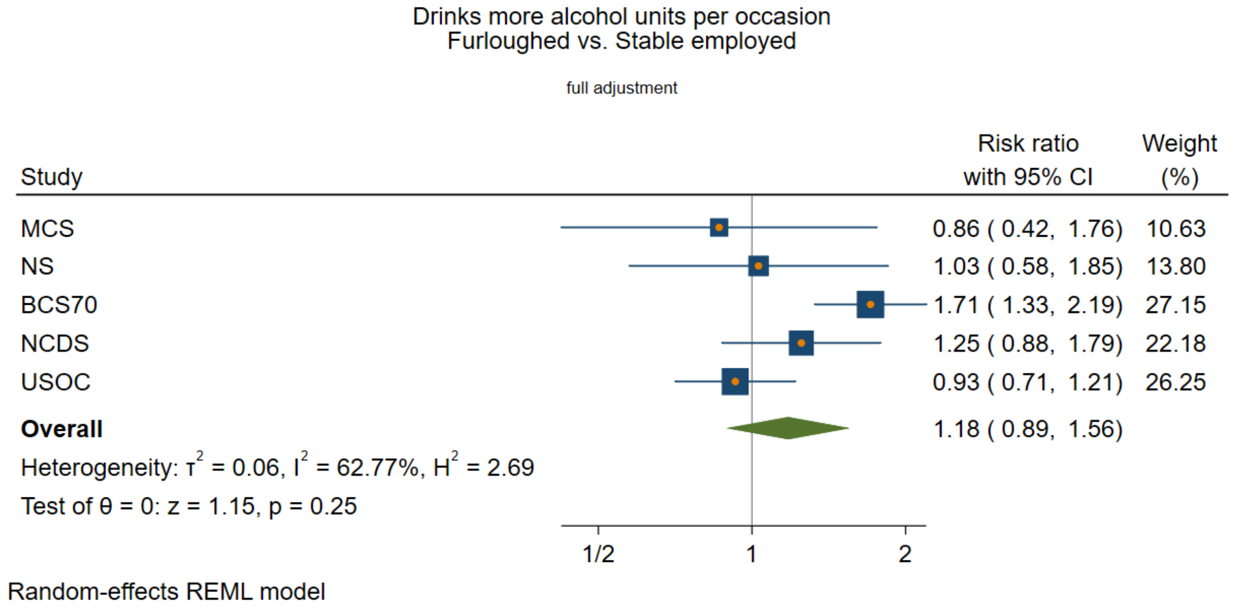

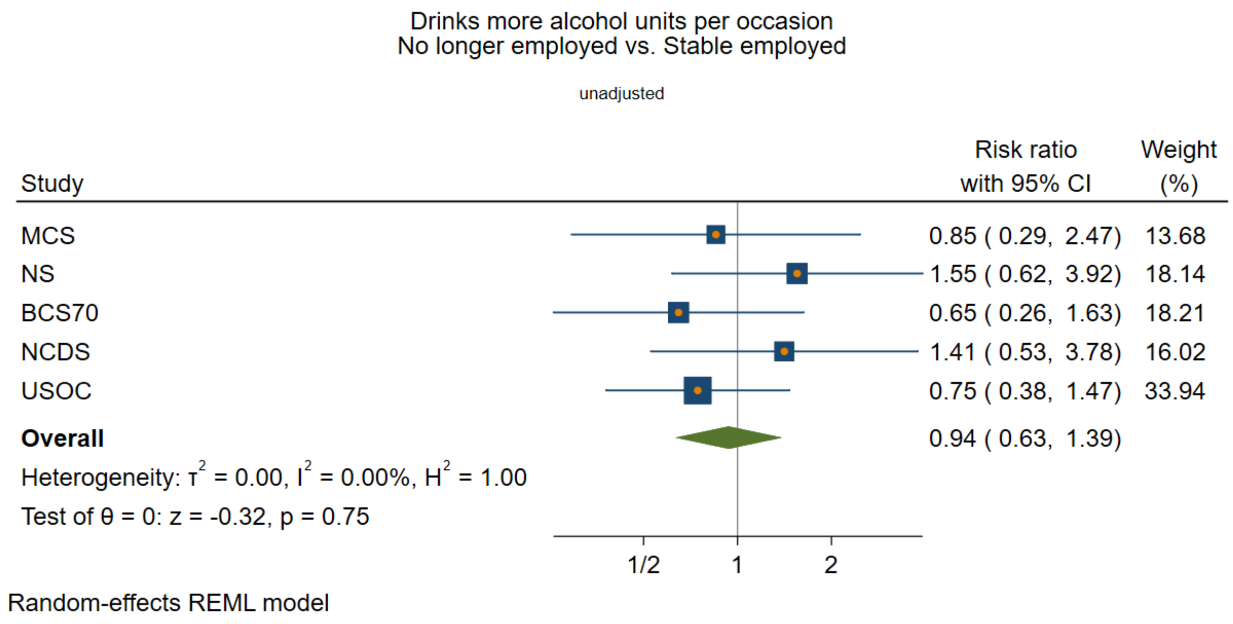
Drinks more alcohol units per occasion
No longer employed vs. Stable employed
unadjusted
Study
Risk ratio with 95% CI
Weight (%)
MCS 0.85 (0.29, 2.47) 13.68
NS 1.55 (0.62, 3.92) 18.14
BCS70 0.65 (0.26, 1.63) 18.21
NCDS 1.41 (0.53, 3.78) 16.02
USOC 0.75 (0.38, 1.47) 33.94
Overall 0.94 (0.63, 1.39)
Heterogeneity: τ² = 0.00, I² = 0.00%, H² = 1.00
Test of θ = 0: z = -0.32, p = 0.75
1/2
1
2
Random-effects REML model

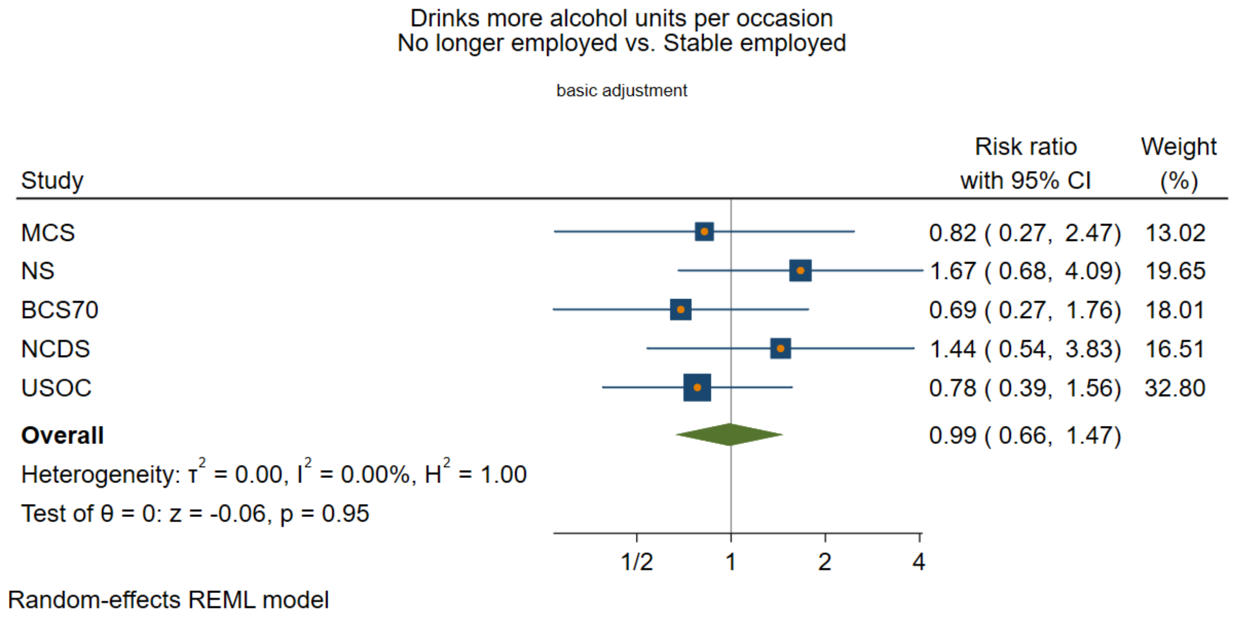
Drinks more alcohol units per occasion
No longer employed vs. Stable employed
basic adjustment
Study
Risk ratio with 95% CI
Weight (%)
MCS 0.82 (0.27, 2.47) 13.02
NS 1.67 (0.68, 4.09) 19.65
BCS70 0.69 (0.27, 1.76) 18.01
NCDS 1.44 (0.54, 3.83) 16.51
USOC 0.78 (0.39, 1.56) 32.80
Overall 0.99 (0.66, 1.47)
Heterogeneity: τ² = 0.00, I² = 0.00%, H² = 1.00
Test of θ = 0: z = -0.06, p = 0.95
1/2
1
2
4
Random-effects REML model

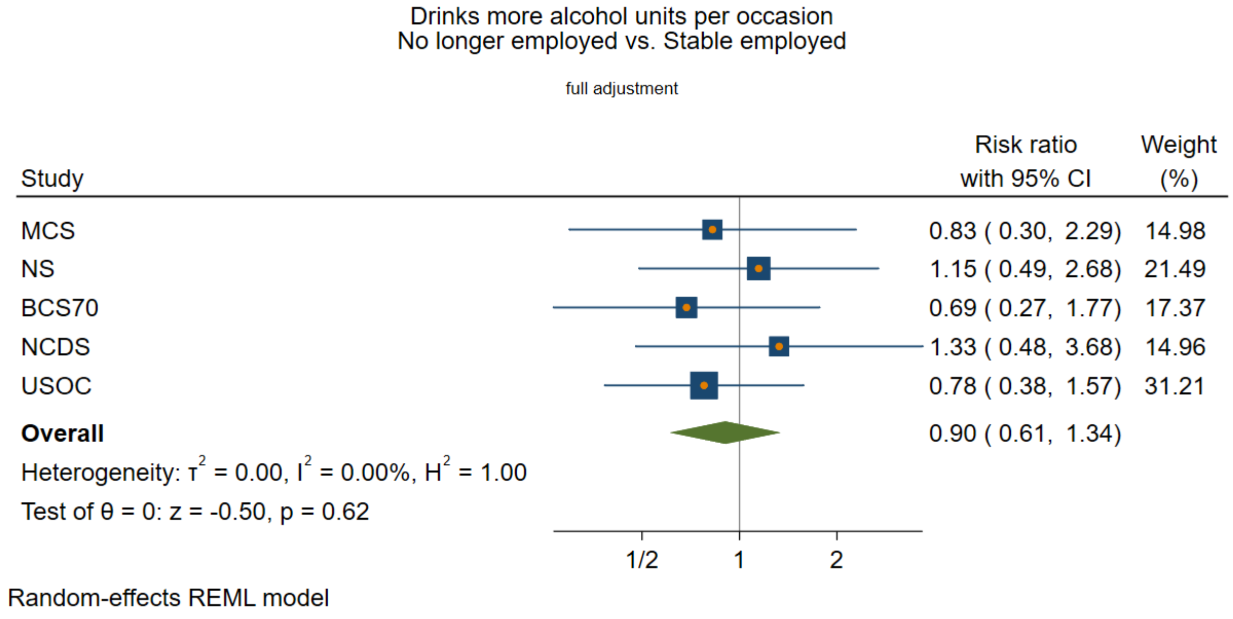
Drinks more alcohol units per occasion
No longer employed vs. Stable employed
full adjustment
Study
Risk ratio with 95% CI
Weight (%)
MCS 0.83 (0.30, 2.29) 14.98
NS 1.15 (0.49, 2.68) 21.49
BCS70 0.69 (0.27, 1.77) 17.37
NCDS 1.33 (0.48, 3.68) 14.96
USOC 0.78 (0.38, 1.57) 31.21
Overall 0.90 (0.61, 1.34)
Heterogeneity: τ² = 0.00, I² = 0.00%, H² = 1.00
Test of θ = 0: z = -0.50, p = 0.62
1/2
1
2
Random-effects REML model

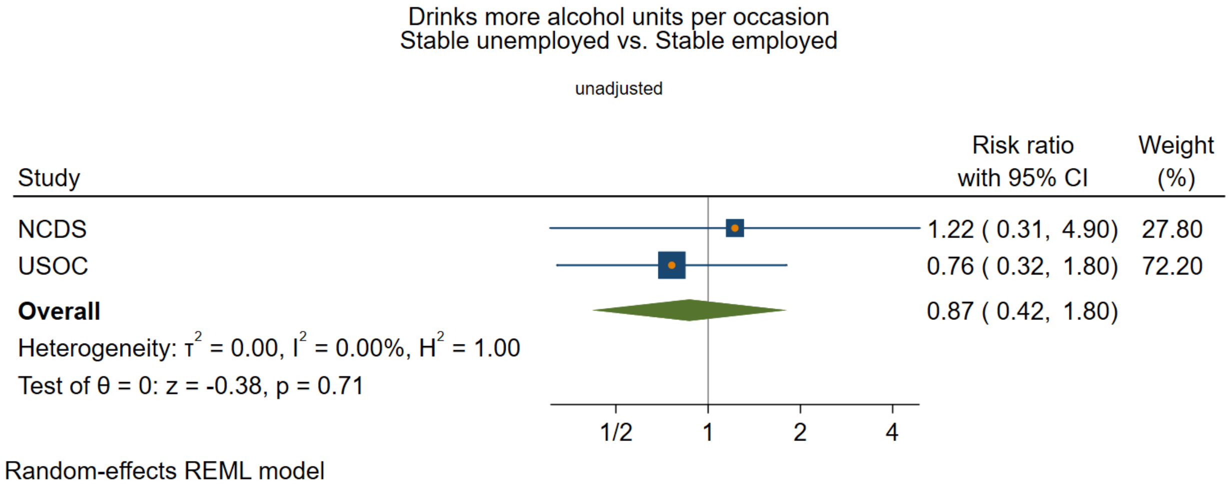
Drinks more alcohol units per occasion
Stable unemployed vs. Stable employed
unadjusted
Study
Risk ratio with 95% CI
Weight (%)
NCDS 1.22 (0.31, 4.90) 27.80
USOC 0.76 (0.32, 1.80) 72.20
Overall 0.87 (0.42, 1.80)
Heterogeneity: τ² = 0.00, I² = 0.00%, H² = 1.00
Test of θ = 0: z = -0.38, p = 0.71
1/2
1
2
4
Random-effects REML model

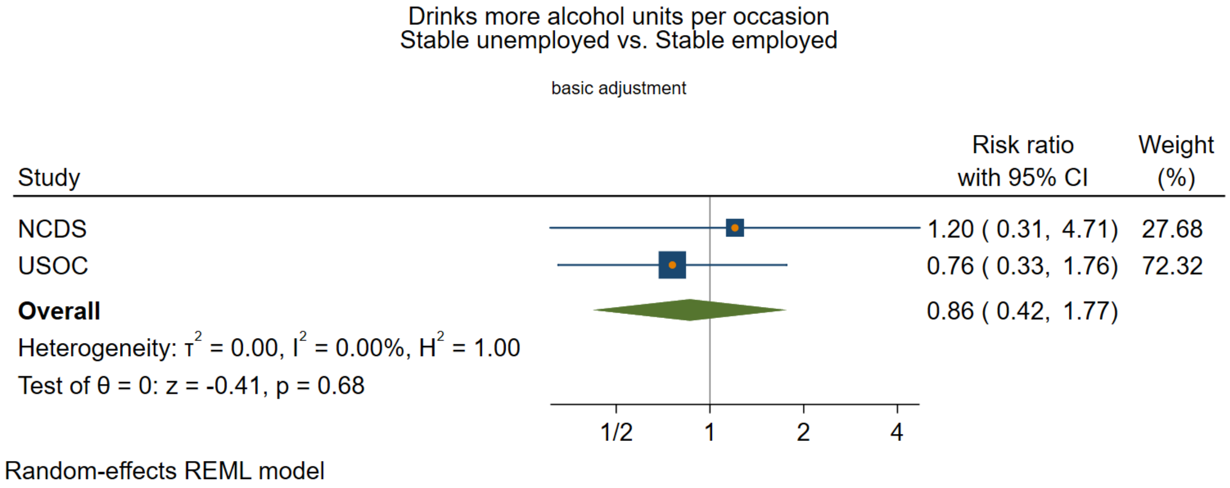
Drinks more alcohol units per occasion
Stable unemployed vs. Stable employed
basic adjustment
Study
Risk ratio with 95% CI
Weight (%)
NCDS 1.20 (0.31, 4.71) 27.68
USOC 0.76 (0.33, 1.76) 72.32
Overall 0.86 (0.42, 1.77)
Heterogeneity: τ² = 0.00, I² = 0.00%, H² = 1.00
Test of θ = 0: z = -0.41, p = 0.68
1/2
1
2
4
Random-effects REML model

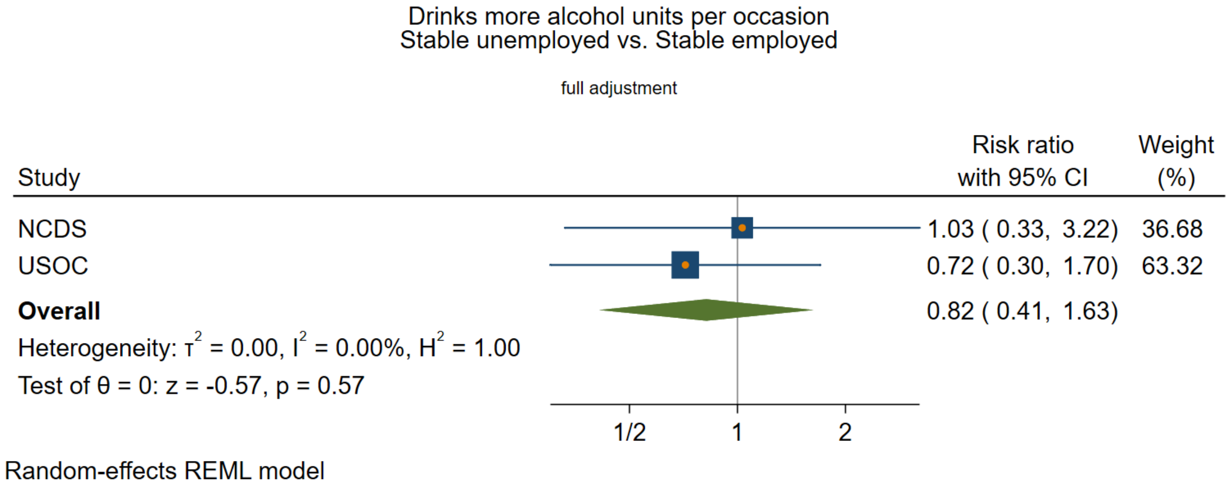
Drinks more alcohol units per occasion
Stable unemployed vs. Stable employed
full adjustment
Study
Risk ratio with 95% CI
Weight (%)
NCDS 1.03 (0.33, 3.22) 36.68
USOC 0.72 (0.30, 1.70) 63.32
Overall 0.82 (0.41, 1.63)
Heterogeneity: τ² = 0.00, I² = 0.00%, H² = 1.00
Test of θ = 0: z = -0.57, p = 0.57
1/2
1
2
Random-effects REML model

Figure set 6. Drinks fewer alcohol units per occasion

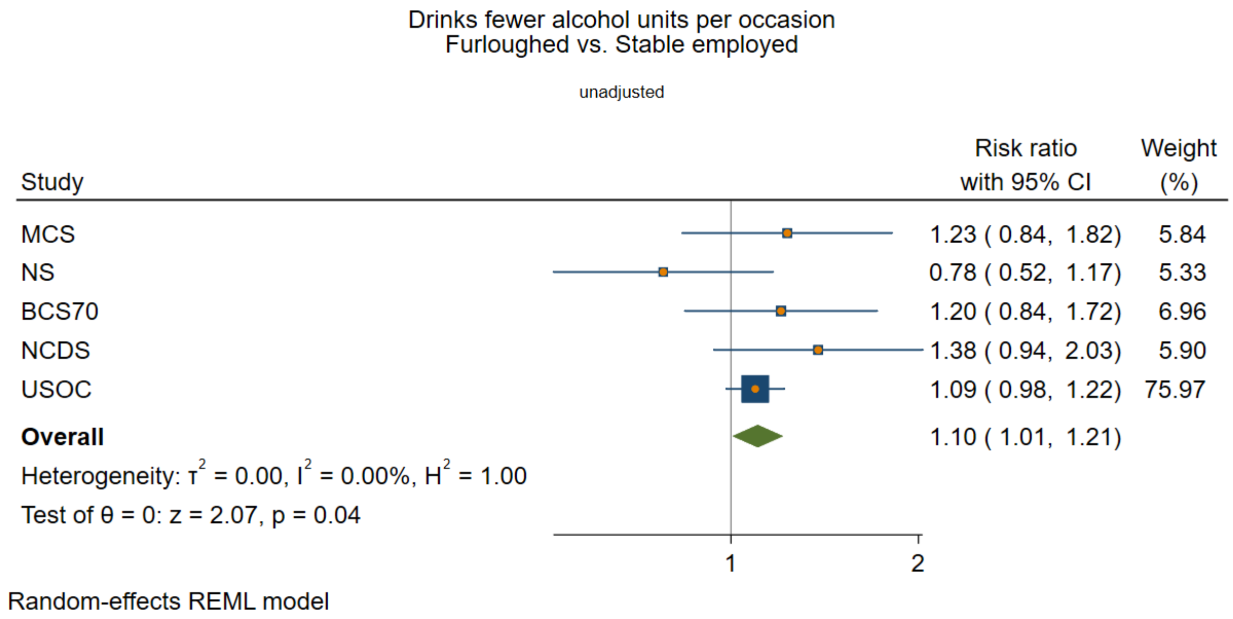

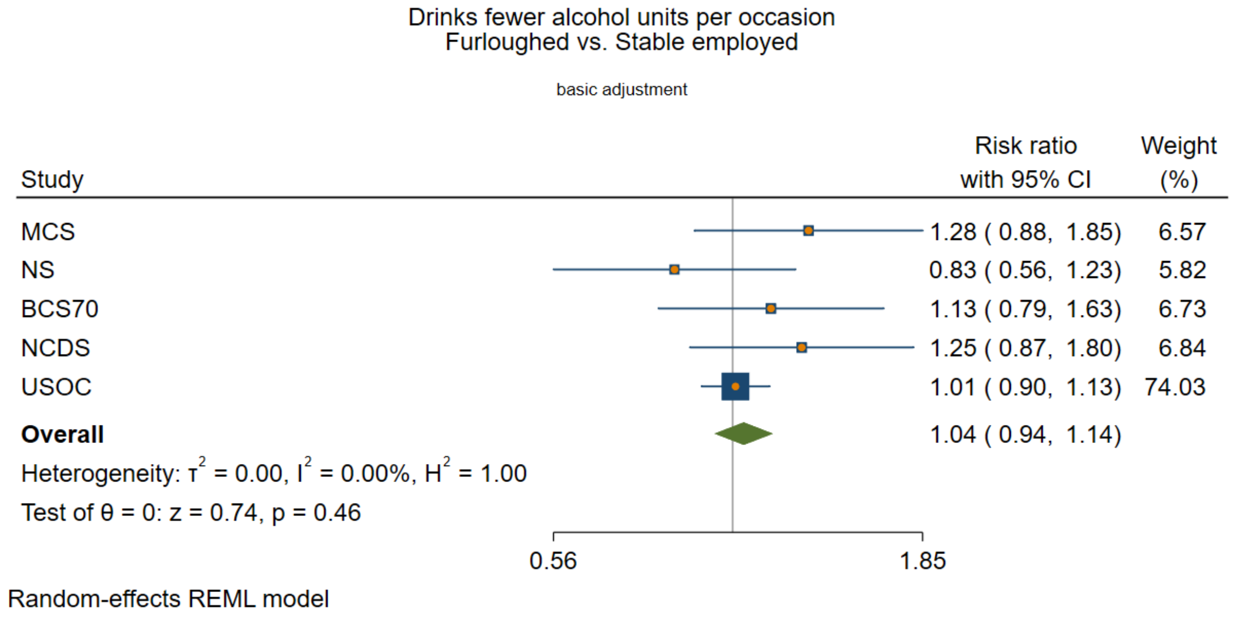

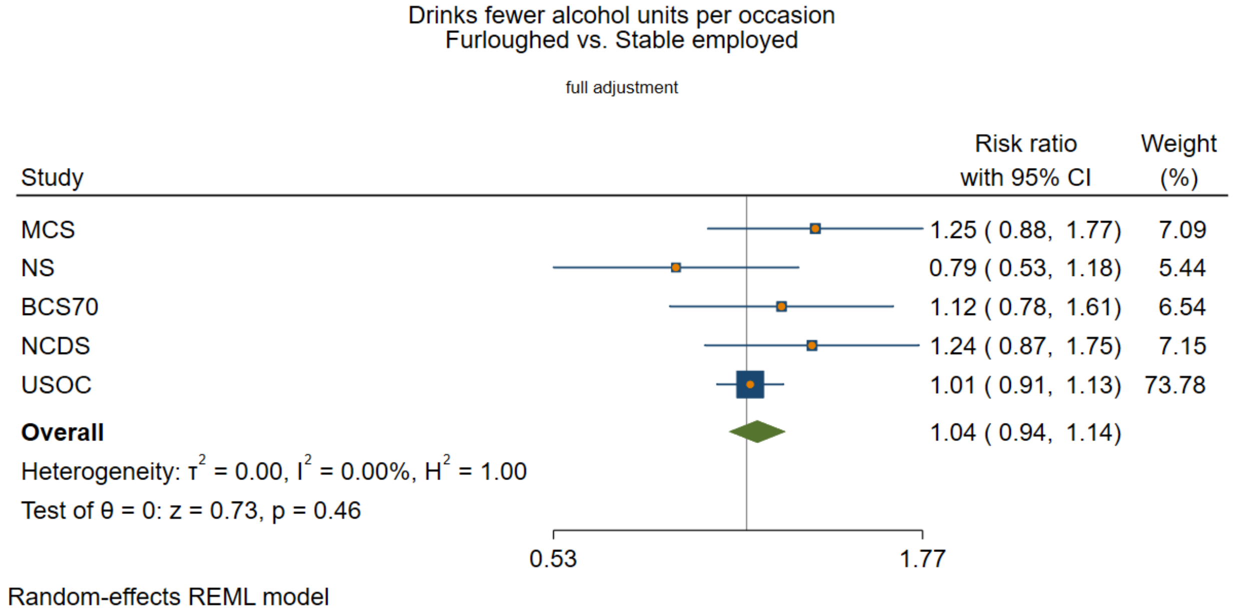

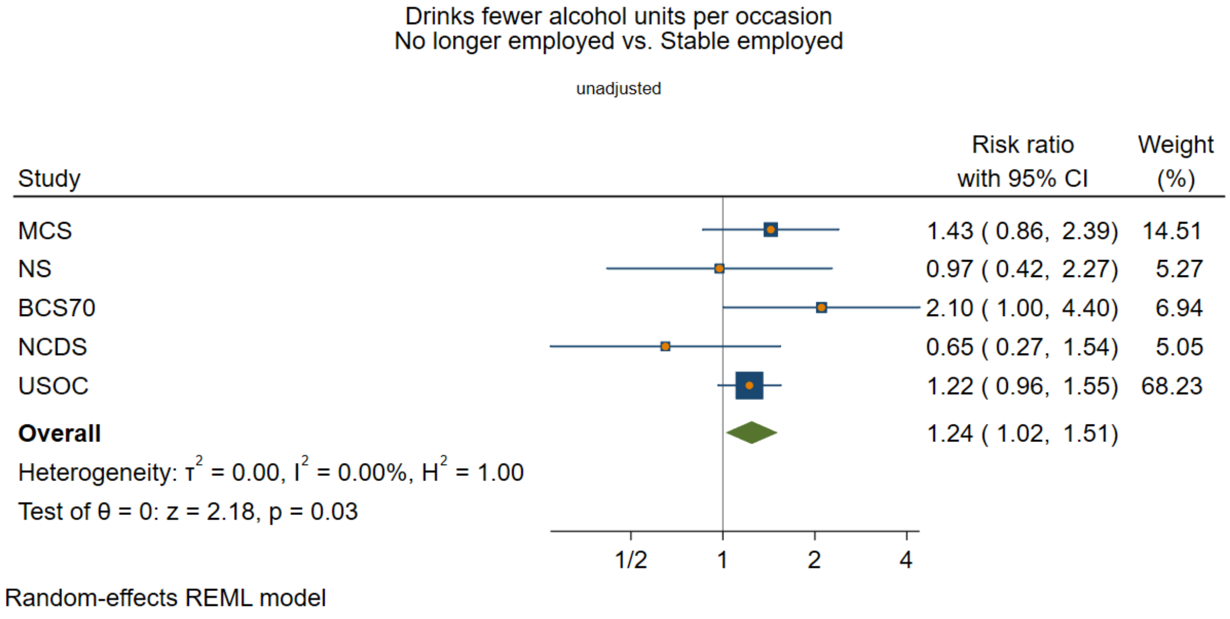
Drinks fewer alcohol units per occasion
No longer employed vs. Stable employed
unadjusted
Study
Risk ratio with 95% CI
Weight (%)
MCS 1.43 (0.86, 2.39) 14.51
NS 0.97 (0.42, 2.27) 5.27
BCS70 2.10 (1.00, 4.40) 6.94
NCDS 0.65 (0.27, 1.54) 5.05
USOC 1.22 (0.96, 1.55) 68.23
Overall 1.24 (1.02, 1.51)
Heterogeneity: τ² = 0.00, I² = 0.00%, H² = 1.00
Test of θ = 0: z = 2.18, p = 0.03
1/2
1
2
4
Random-effects REML model

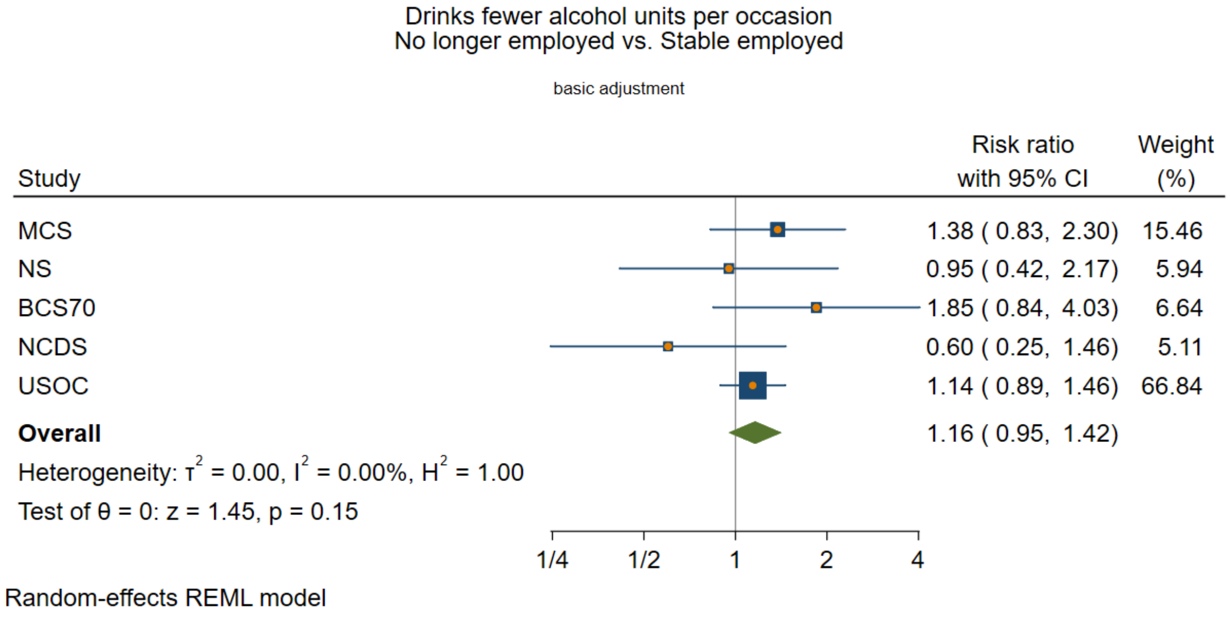
Drinks fewer alcohol units per occasion
No longer employed vs. Stable employed
basic adjustment
Study
Risk ratio with 95% CI
Weight (%)
MCS 1.38 (0.83, 2.30) 15.46
NS 0.95 (0.42, 2.17) 5.94
BCS70 1.85 (0.84, 4.03) 6.64
NCDS 0.60 (0.25, 1.46) 5.11
USOC 1.14 (0.89, 1.46) 66.84
Overall 1.16 (0.95, 1.42)
Heterogeneity: τ² = 0.00, I² = 0.00%, H² = 1.00
Test of θ = 0: z = 1.45, p = 0.15
1/4
1/2
1
2
4
Random-effects REML model

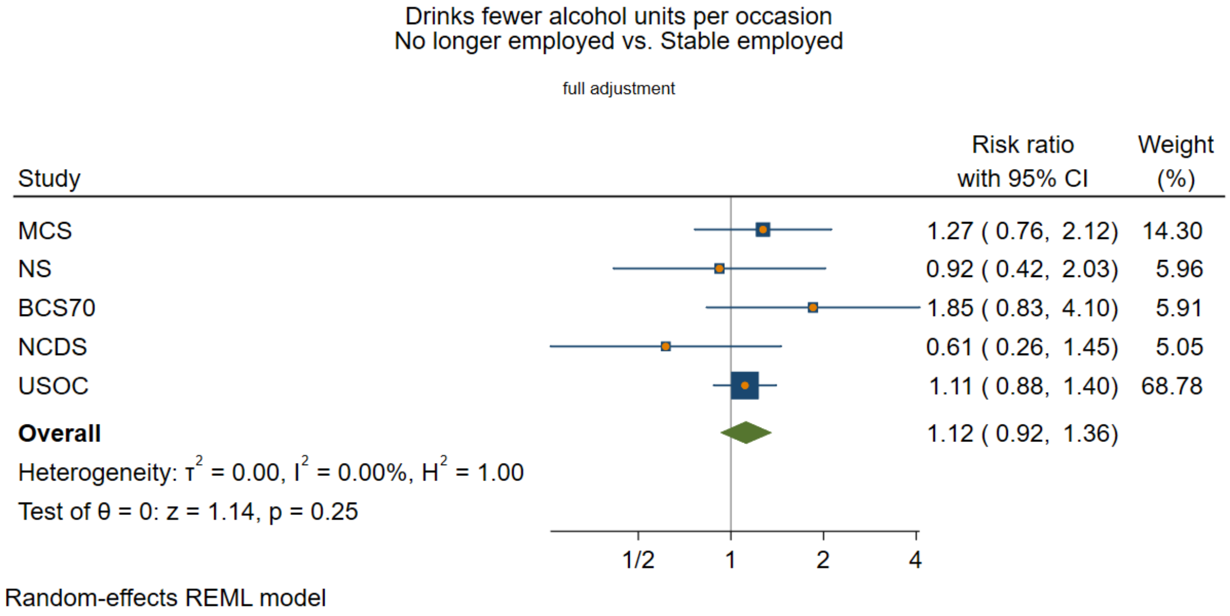
Drinks fewer alcohol units per occasion
No longer employed vs. Stable employed
full adjustment
Study
Risk ratio with 95% CI
Weight (%)
MCS 1.27 (0.76, 2.12) 14.30
NS 0.92 (0.42, 2.03) 5.96
BCS70 1.85 (0.83, 4.10) 5.91
NCDS 0.61 (0.26, 1.45) 5.05
USOC 1.11 (0.88, 1.40) 68.78
Overall 1.12 (0.92, 1.36)
Heterogeneity: τ² = 0.00, I² = 0.00%, H² = 1.00
Test of θ = 0: z = 1.14, p = 0.25
1/2
1
2
4
Random-effects REML model

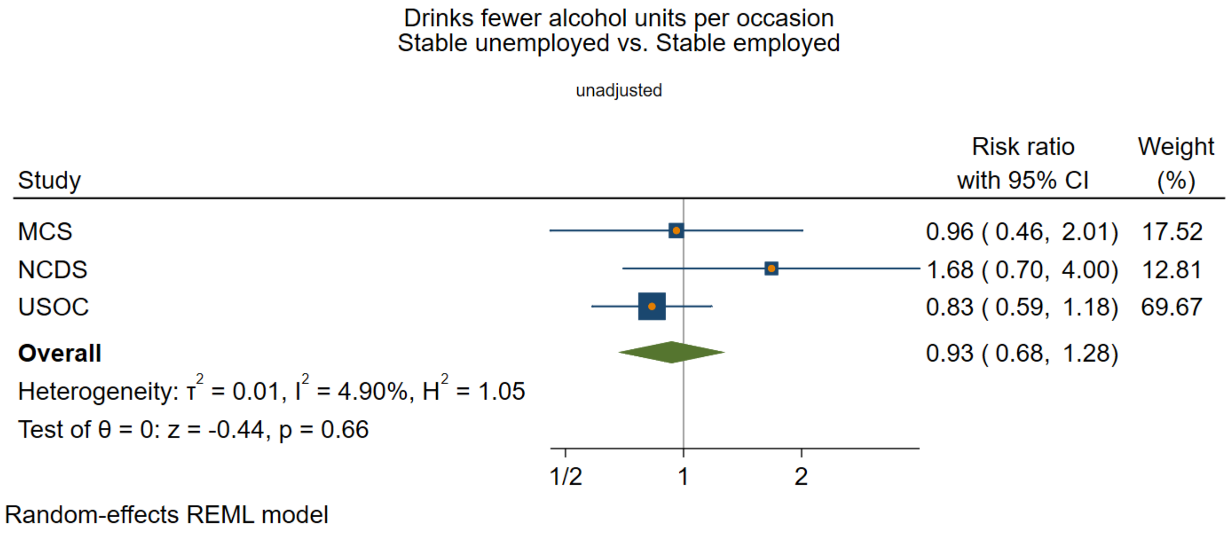

Drinks fewer alcohol units per occasion
Stable unemployed vs. Stable employed
unadjusted
Study
Risk ratio with 95% CI
Weight (%)
MCS 0.96 (0.46, 2.01) 17.52
NCDS 1.68 (0.70, 4.00) 12.81
USOC 0.83 (0.59, 1.18) 69.67
Overall 0.93 (0.68, 1.28)
Heterogeneity: τ² = 0.01, I² = 4.90%, H² = 1.05
Test of θ = 0: z = -0.44, p = 0.66
1/2
1
2
Random-effects REML model

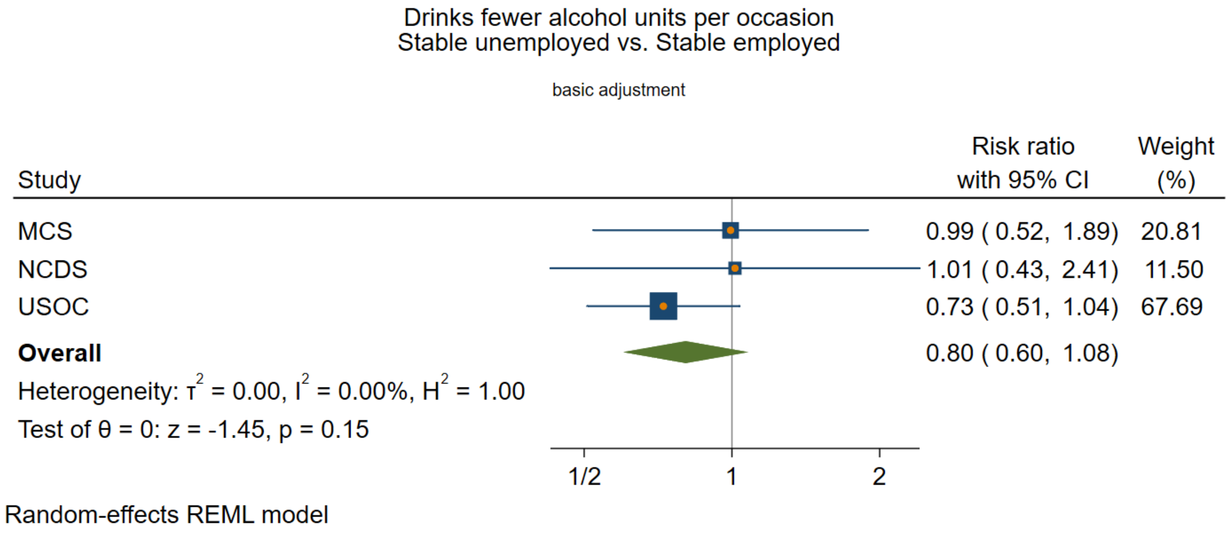

Drinks fewer alcohol units per occasion
Stable unemployed vs. Stable employed
basic adjustment
Study
Risk ratio with 95% CI
Weight (%)
MCS 0.99 (0.52, 1.89) 20.81
NCDS 1.01 (0.43, 2.41) 11.50
USOC 0.73 (0.51, 1.04) 67.69
Overall 0.80 (0.60, 1.08)
Heterogeneity: τ² = 0.00, I² = 0.00%, H² = 1.00
Test of θ = 0: z = -1.45, p = 0.15
1/2
1
2
Random-effects REML model

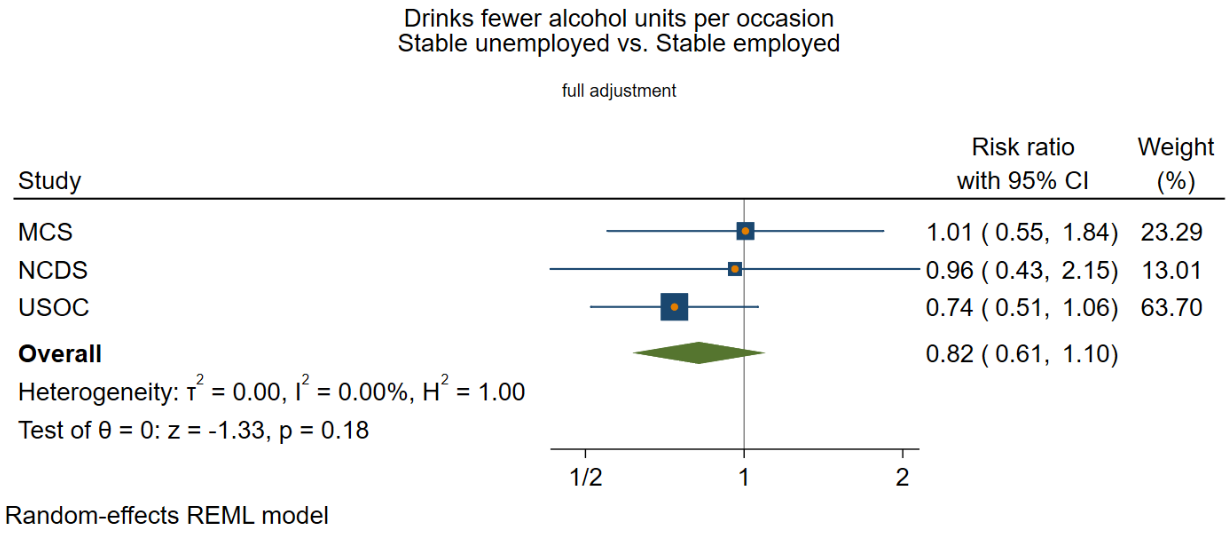

Drinks fewer alcohol units per occasion
Stable unemployed vs. Stable employed
full adjustment
Study
Risk ratio with 95% CI
Weight (%)
MCS 1.01 (0.55, 1.84) 23.29
NCDS 0.96 (0.43, 2.15) 13.01
USOC 0.74 (0.51, 1.06) 63.70
Overall 0.82 (0.61, 1.10)
Heterogeneity: τ² = 0.00, I² = 0.00%, H² = 1.00
Test of θ = 0: z = -1.33, p = 0.18
1/2
1
2
Random-effects REML model

Figure set 7. Currently drinks 4+ days/week

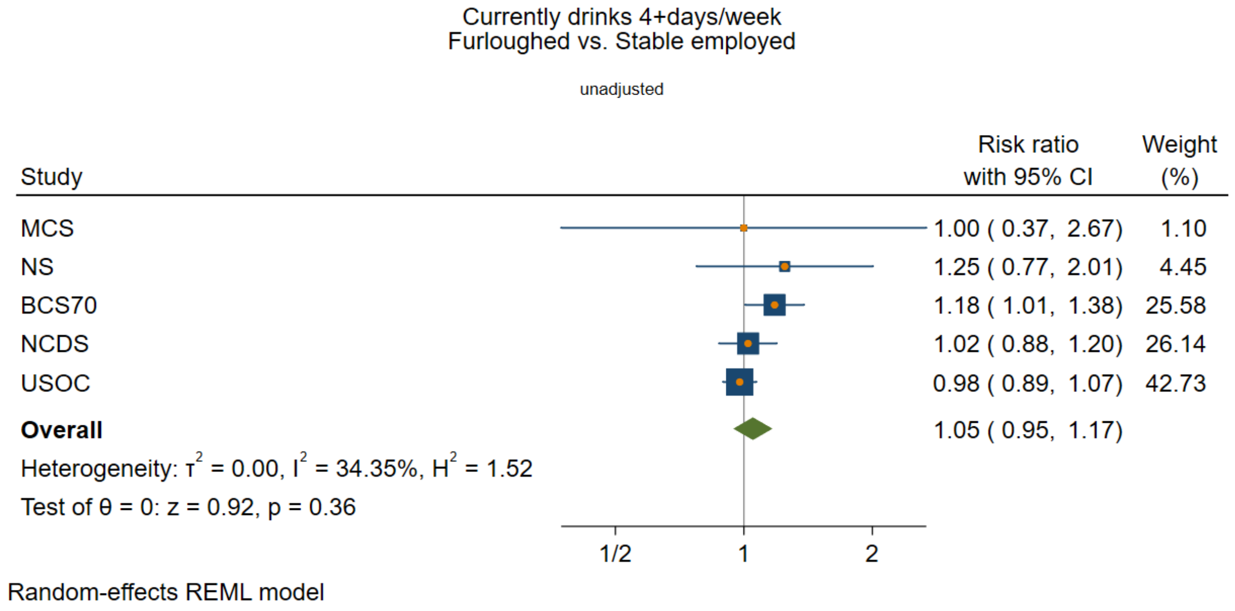

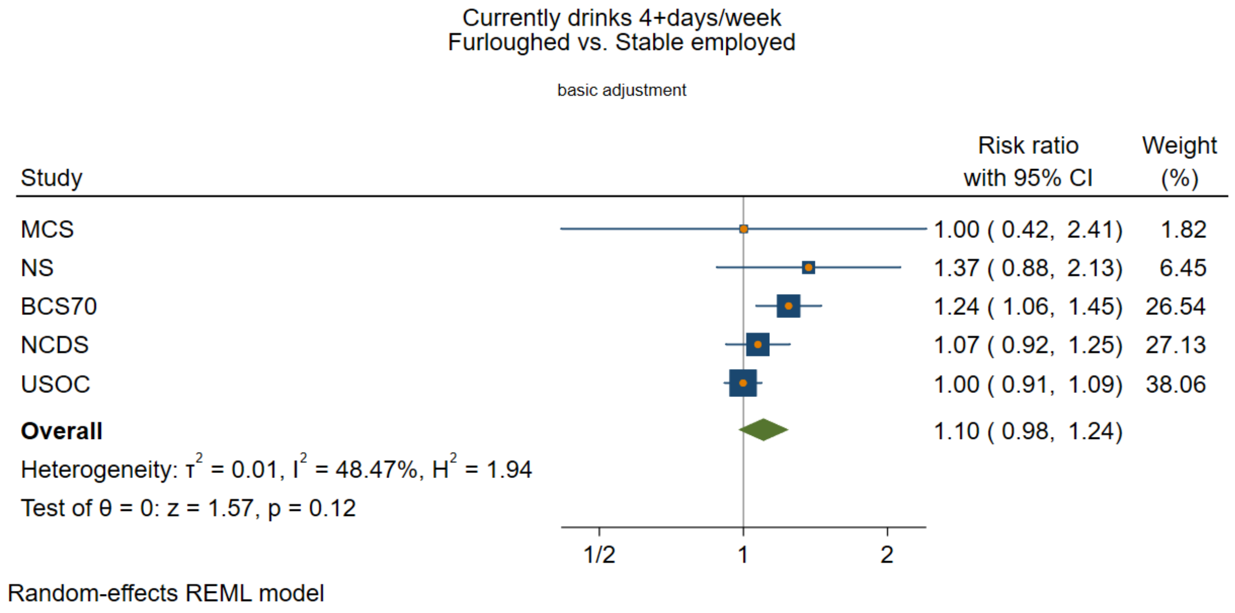

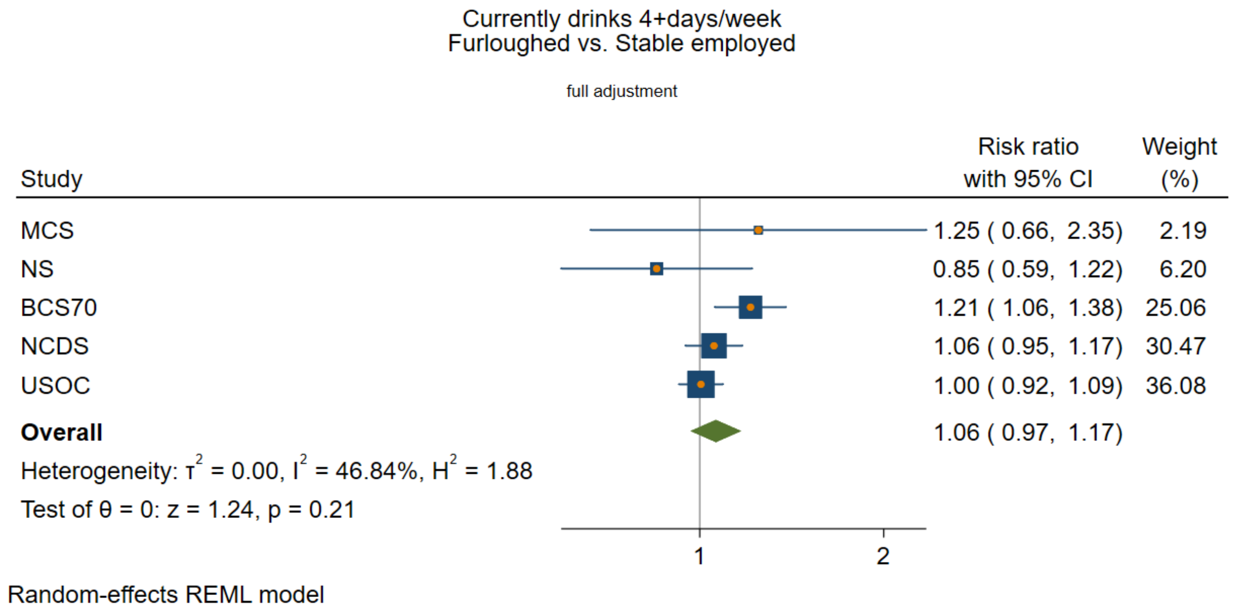

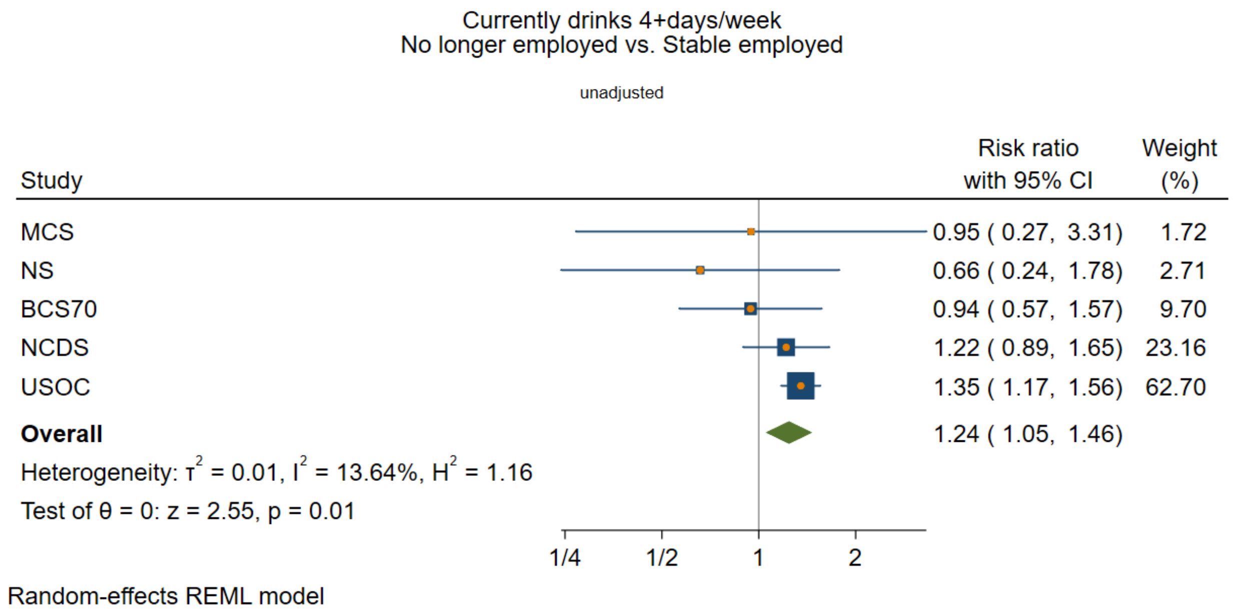
Currently drinks 4+days/week
No longer employed vs. Stable employed
unadjusted
Study
Risk ratio with 95% CI
Weight (%)
MCS 0.95 (0.27, 3.31) 1.72
NS 0.66 (0.24, 1.78) 2.71
BCS70 0.94 (0.57, 1.57) 9.70
NCDS 1.22 (0.89, 1.65) 23.16
USOC 1.35 (1.17, 1.56) 62.70
Overall 1.24 (1.05, 1.46)
Heterogeneity: τ² = 0.01, I² = 13.64%, H² = 1.16
Test of θ = 0: z = 2.55, p = 0.01
1/4
1/2
1
2
Random-effects REML model

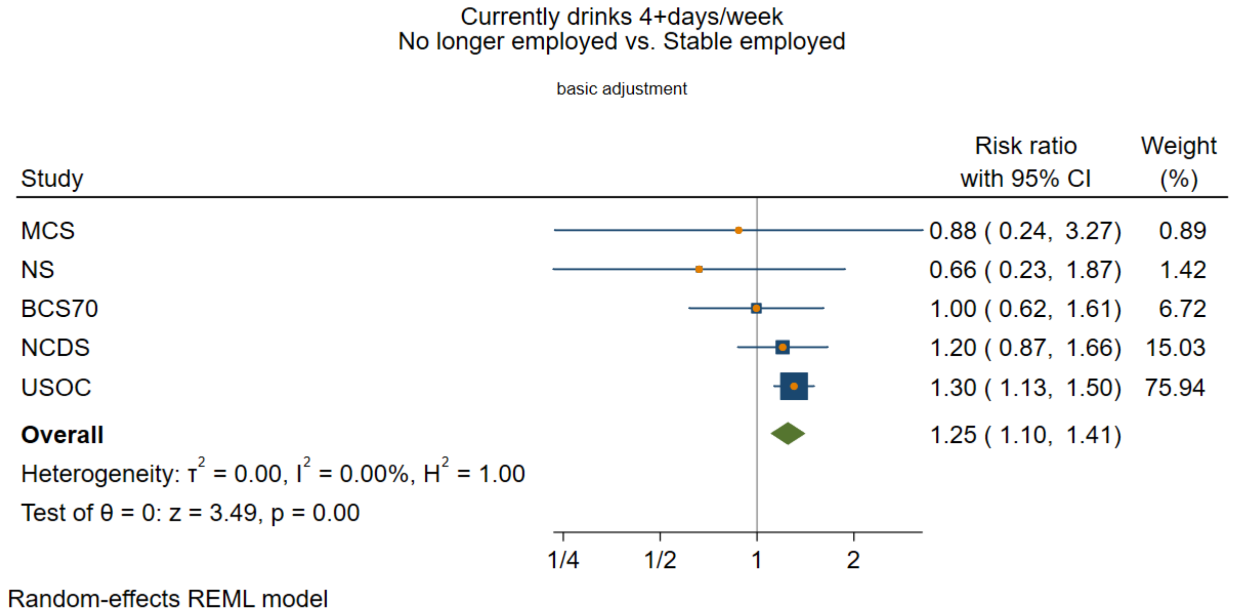
Currently drinks 4+days/week
No longer employed vs. Stable employed
basic adjustment
Study
Risk ratio with 95% CI
Weight (%)
MCS 0.88 (0.24, 3.27) 0.89
NS 0.66 (0.23, 1.87) 1.42
BCS70 1.00 (0.62, 1.61) 6.72
NCDS 1.20 (0.87, 1.66) 15.03
USOC 1.30 (1.13, 1.50) 75.94
Overall 1.25 (1.10, 1.41)
Heterogeneity: τ² = 0.00, I² = 0.00%, H² = 1.00
Test of θ = 0: z = 3.49, p = 0.00
1/4
1/2
1
2
Random-effects REML model

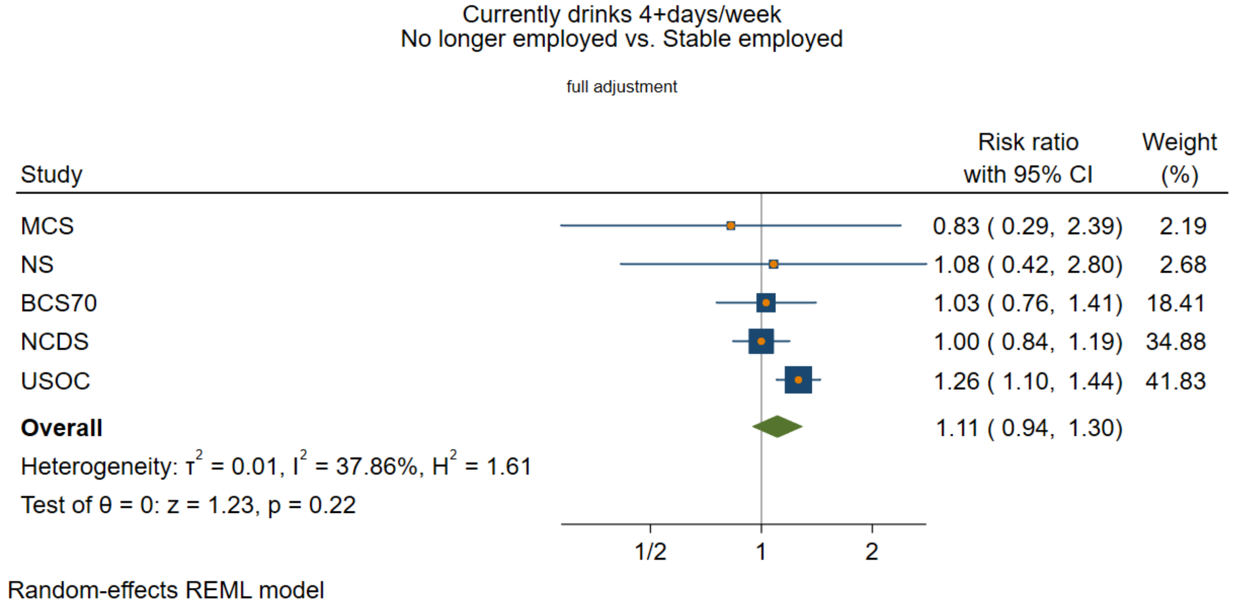
Currently drinks 4+days/week
No longer employed vs. Stable employed
full adjustment
Study
Risk ratio with 95% CI
Weight (%)
MCS 0.83 (0.29, 2.39) 2.19
NS 1.08 (0.42, 2.80) 2.68
BCS70 1.03 (0.76, 1.41) 18.41
NCDS 1.00 (0.84, 1.19) 34.88
USOC 1.26 (1.10, 1.44) 41.83
Overall 1.11 (0.94, 1.30)
Heterogeneity: τ² = 0.01, I² = 37.86%, H² = 1.61
Test of θ = 0: z = 1.23, p = 0.22
1/2
1
2
Random-effects REML model

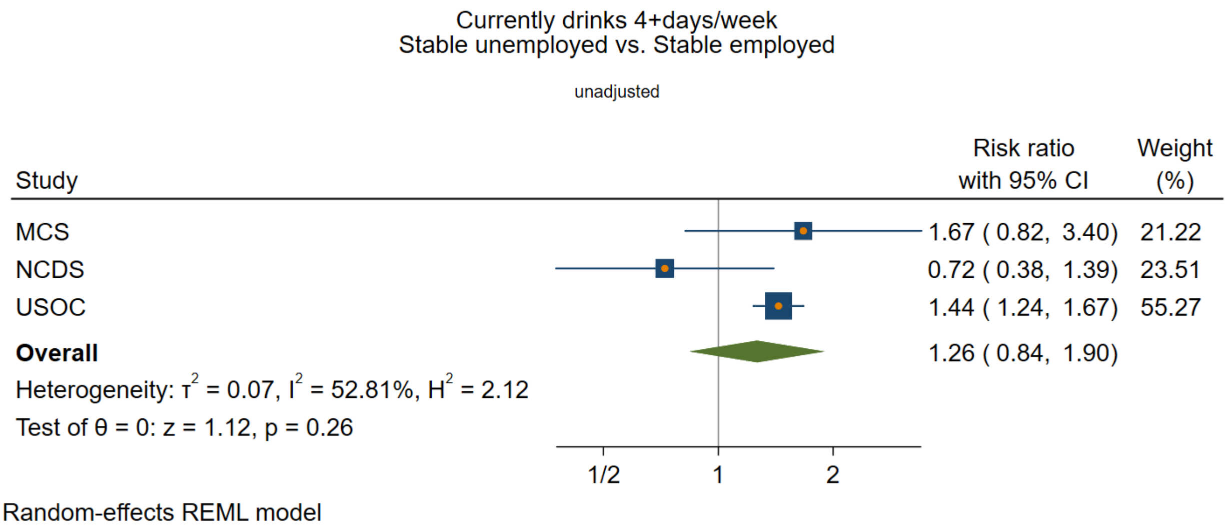
Currently drinks 4+days/week
Stable unemployed vs. Stable employed
unadjusted
Study
Risk ratio with 95% CI
Weight (%)
MCS 1.67 (0.82, 3.40) 21.22
NCDS 0.72 (0.38, 1.39) 23.51
USOC 1.44 (1.24, 1.67) 55.27
Overall 1.26 (0.84, 1.90)
Heterogeneity: τ² = 0.07, I² = 52.81%, H² = 2.12
Test of θ = 0: z = 1.12, p = 0.26
1/2
1
2
Random-effects REML model

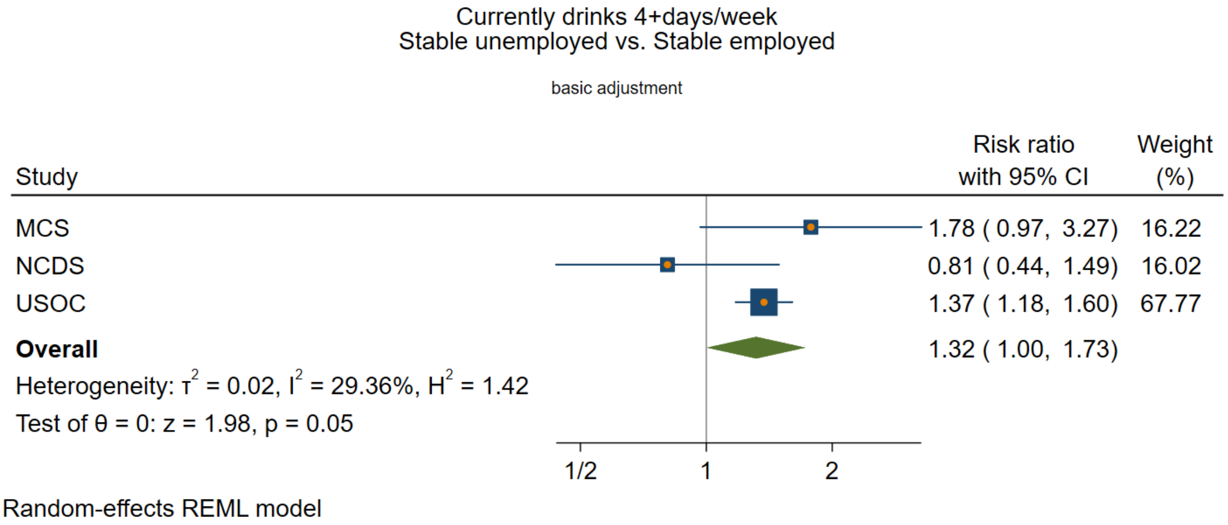
Currently drinks 4+days/week
Stable unemployed vs. Stable employed
basic adjustment
Study
Risk ratio with 95% CI
Weight (%)
MCS 1.78 (0.97, 3.27) 16.22
NCDS 0.81 (0.44, 1.49) 16.02
USOC 1.37 (1.18, 1.60) 67.77
Overall 1.32 (1.00, 1.73)
Heterogeneity: τ² = 0.02, I² = 29.36%, H² = 1.42
Test of θ = 0: z = 1.98, p = 0.05
1/2
1
2
Random-effects REML model

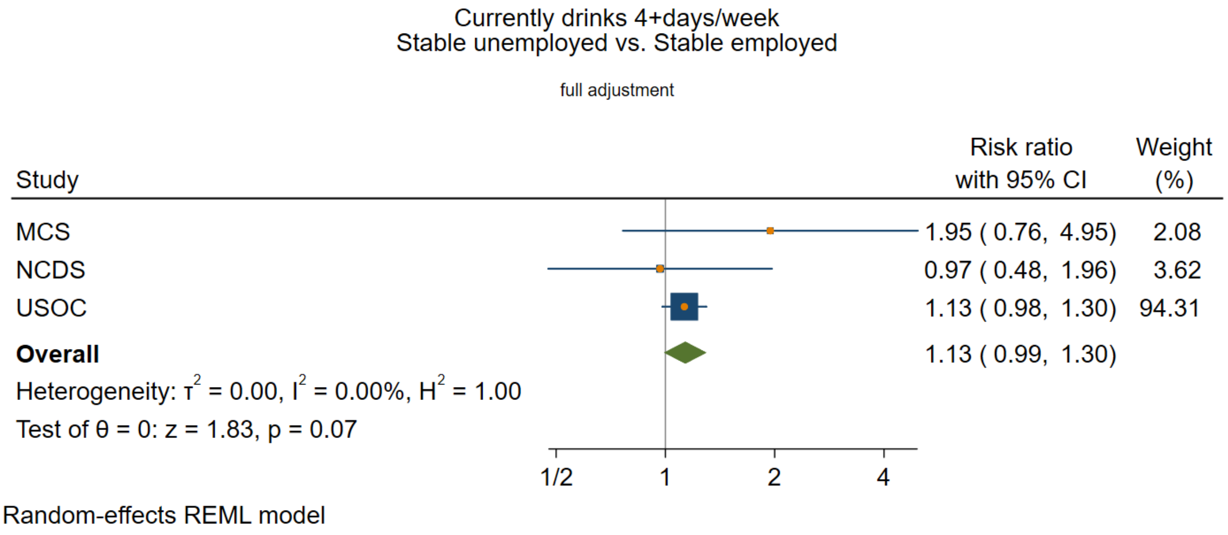
Currently drinks 4+days/week
Stable unemployed vs. Stable employed
full adjustment
Study
Risk ratio with 95% CI
Weight (%)
MCS 1.95 (0.76, 4.95) 2.08
NCDS 0.97 (0.48, 1.96) 3.62
USOC 1.13 (0.98, 1.30) 94.31
Overall 1.13 (0.99, 1.30)
Heterogeneity: τ² = 0.00, I² = 0.00%, H² = 1.00
Test of θ = 0: z = 1.83, p = 0.07
1/2
1
2
4
Random-effects REML model

Figure set 8. Drinks more frequently

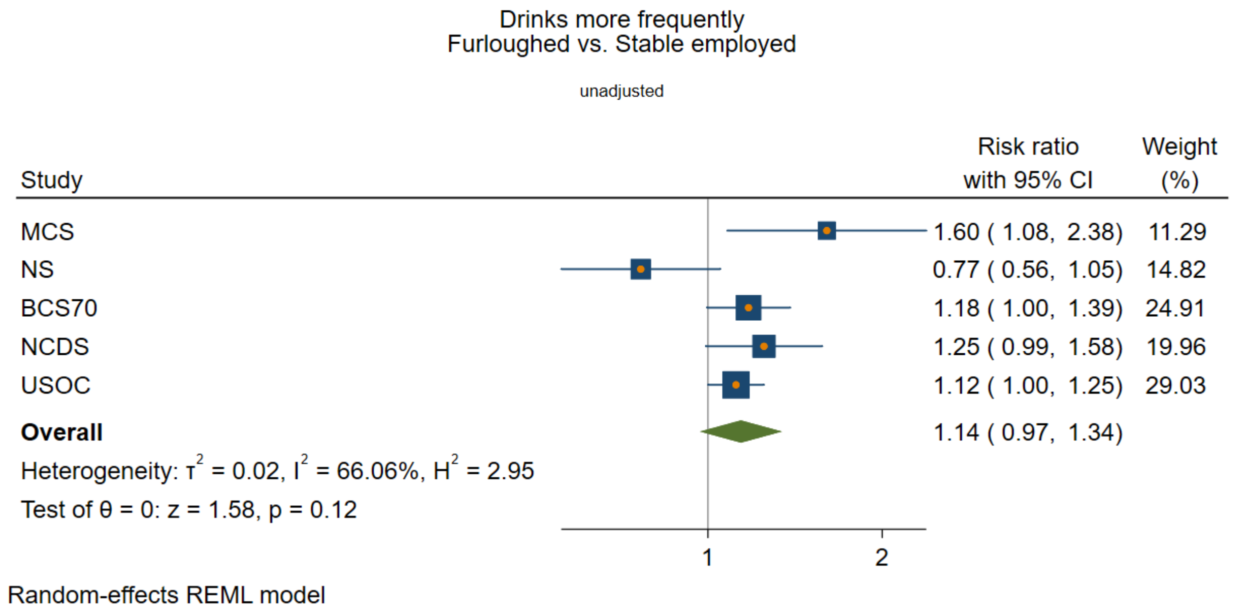

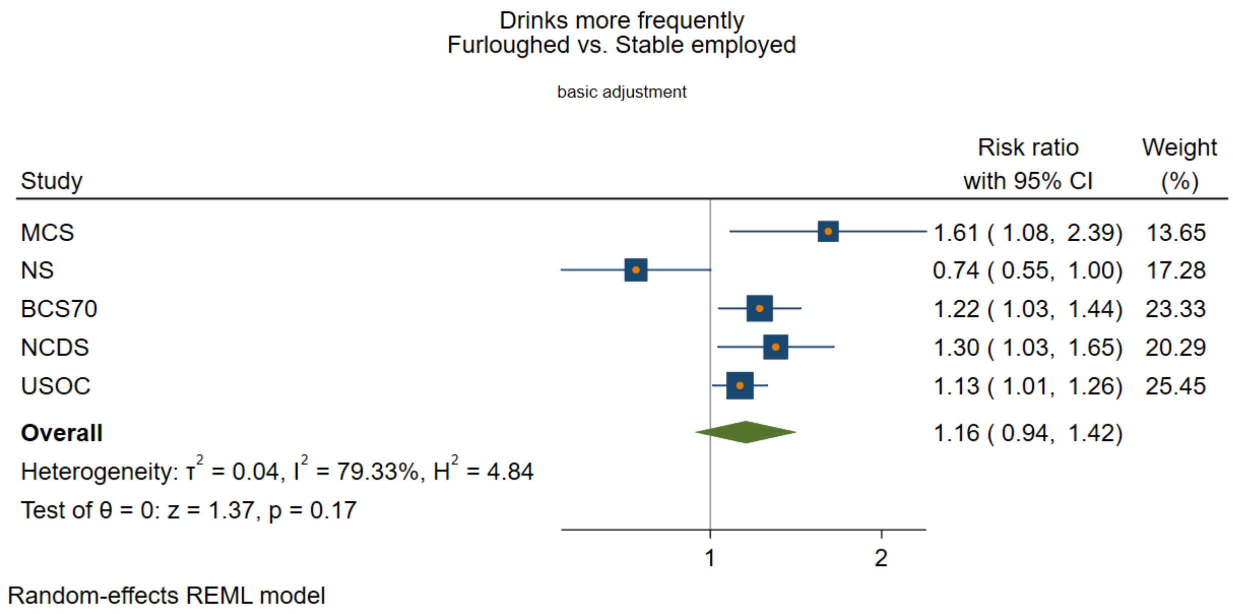

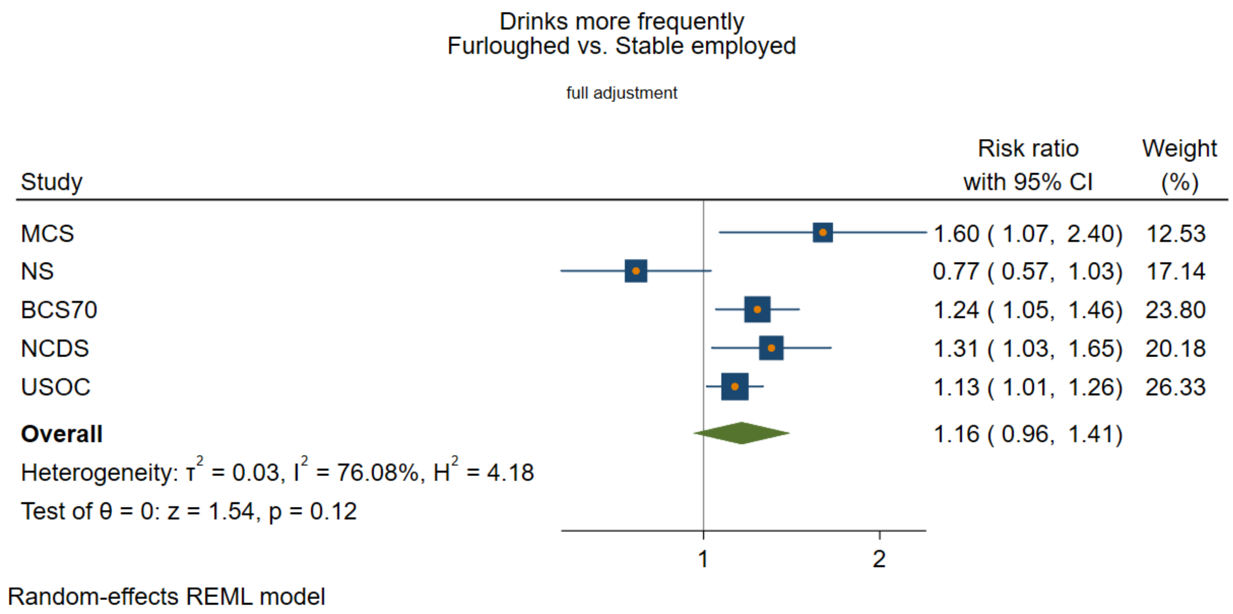

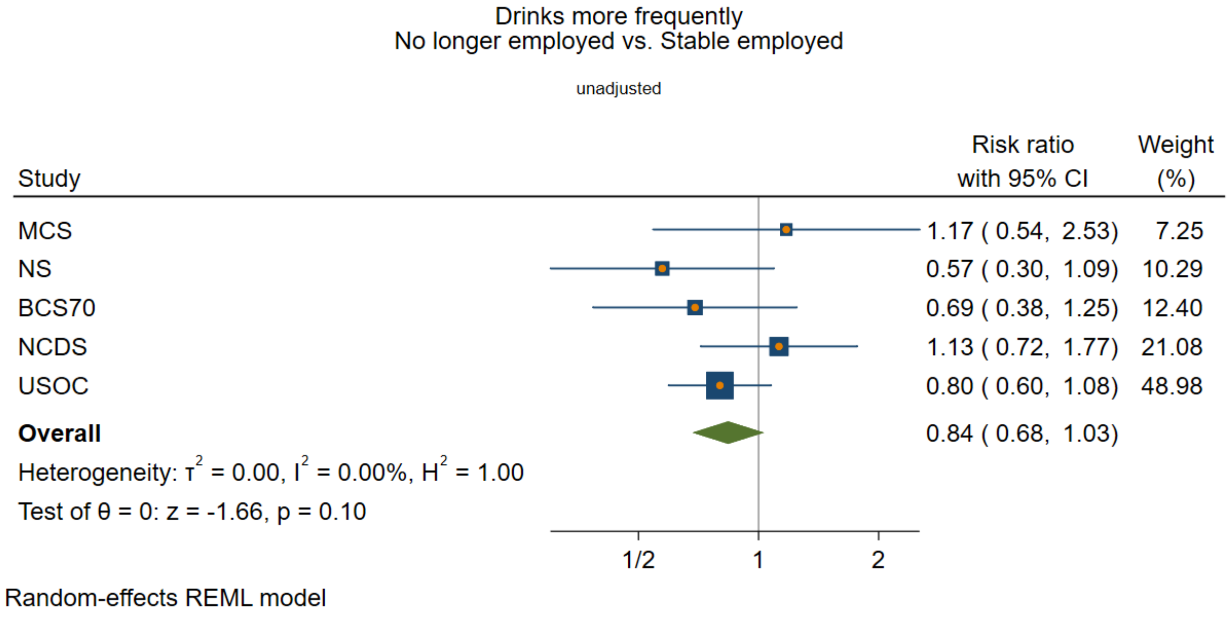
Drinks more frequently
No longer employed vs. Stable employed
unadjusted
Study
Risk ratio with 95% CI
Weight (%)
MCS 1.17 (0.54, 2.53) 7.25
NS 0.57 (0.30, 1.09) 10.29
BCS70 0.69 (0.38, 1.25) 12.40
NCDS 1.13 (0.72, 1.77) 21.08
USOC 0.80 (0.60, 1.08) 48.98
Overall 0.84 (0.68, 1.03)
Heterogeneity: τ² = 0.00, I² = 0.00%, H² = 1.00
Test of θ = 0: z = -1.66, p = 0.10
1/2
1
2
Random-effects REML model

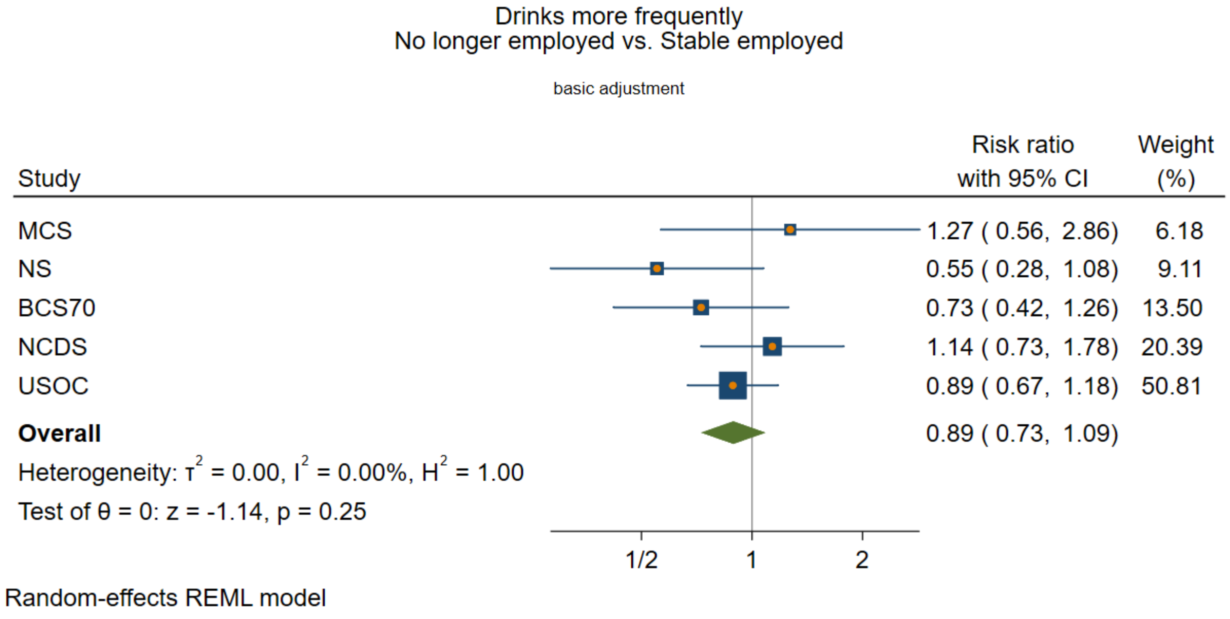
Drinks more frequently
No longer employed vs. Stable employed
basic adjustment
Study
Risk ratio with 95% CI
Weight (%)
MCS 1.27 (0.56, 2.86) 6.18
NS 0.55 (0.28, 1.08) 9.11
BCS70 0.73 (0.42, 1.26) 13.50
NCDS 1.14 (0.73, 1.78) 20.39
USOC 0.89 (0.67, 1.18) 50.81
Overall 0.89 (0.73, 1.09)
Heterogeneity: τ² = 0.00, I² = 0.00%, H² = 1.00
Test of θ = 0: z = -1.14, p = 0.25
1/2
1
2
Random-effects REML model

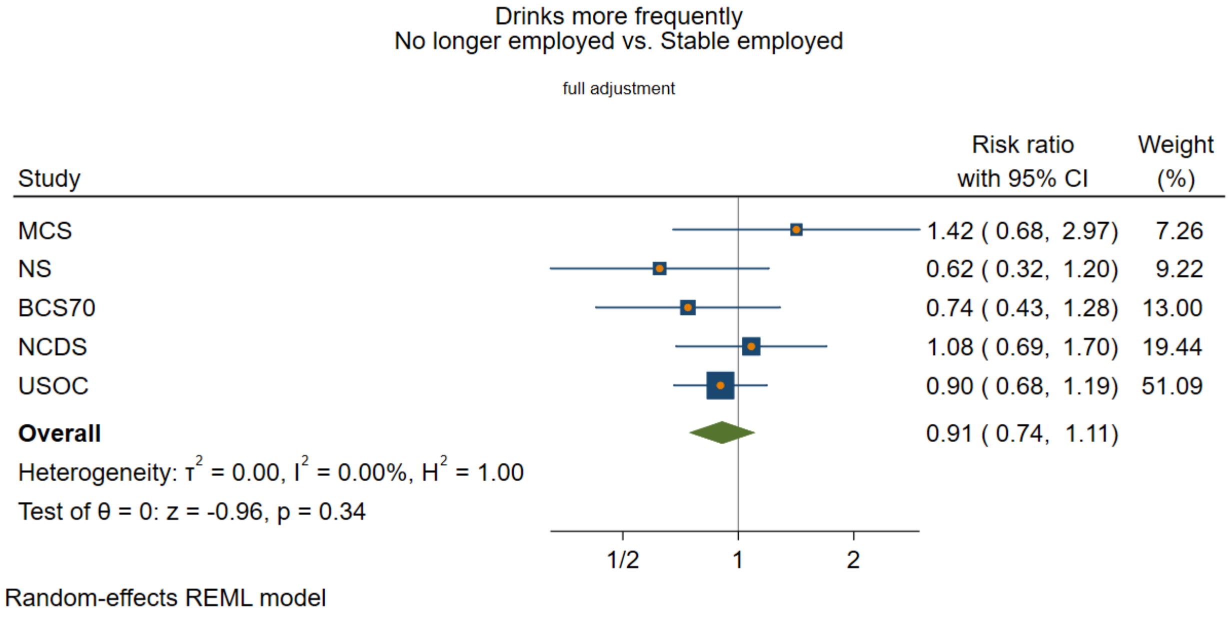
Drinks more frequently
No longer employed vs. Stable employed
full adjustment
Study
Risk ratio with 95% CI
Weight (%)
MCS 1.42 (0.68, 2.97) 7.26
NS 0.62 (0.32, 1.20) 9.22
BCS70 0.74 (0.43, 1.28) 13.00
NCDS 1.08 (0.69, 1.70) 19.44
USOC 0.90 (0.68, 1.19) 51.09
Overall 0.91 (0.74, 1.11)
Heterogeneity: τ² = 0.00, I² = 0.00%, H² = 1.00
Test of θ = 0: z = -0.96, p = 0.34
1/2
1
2
Random-effects REML model

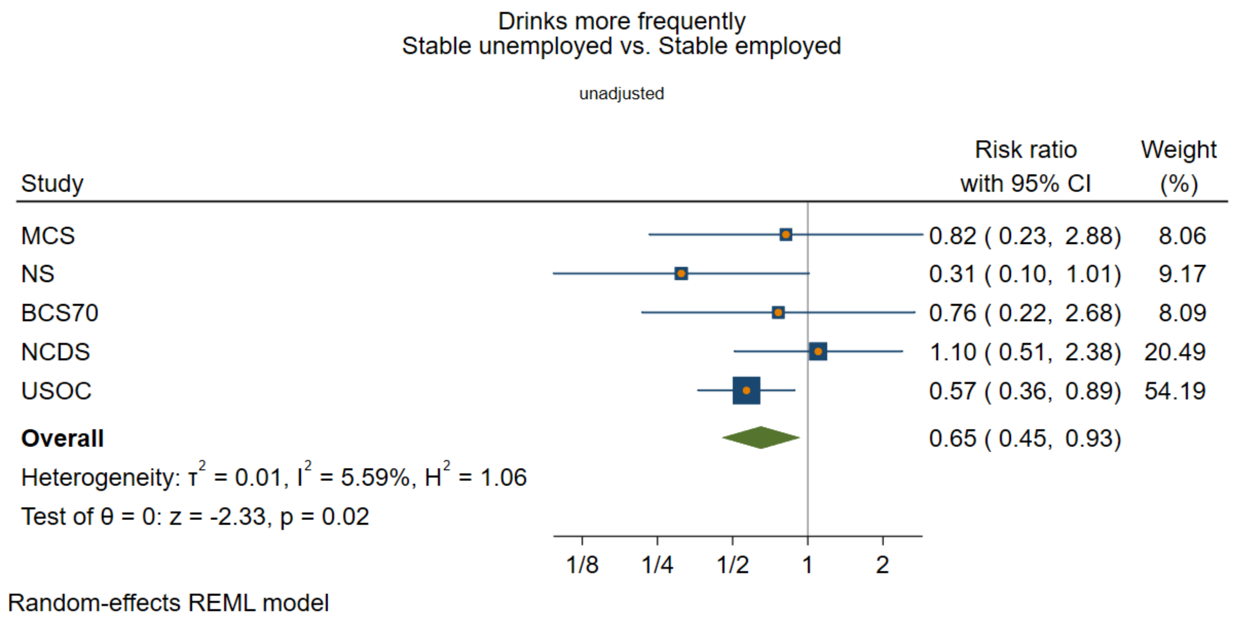
Drinks more frequently
Stable unemployed vs. Stable employed
unadjusted
Study
Risk ratio with 95% CI
Weight (%)
MCS 0.82 (0.23, 2.88) 8.06
NS 0.31 (0.10, 1.01) 9.17
BCS70 0.76 (0.22, 2.68) 8.09
NCDS 1.10 (0.51, 2.38) 20.49
USOC 0.57 (0.36, 0.89) 54.19
Overall 0.65 (0.45, 0.93)
Heterogeneity: τ² = 0.01, I² = 5.59%, H² = 1.06
Test of θ = 0: z = -2.33, p = 0.02
1/8
1/4
1/2
1
2
Random-effects REML model

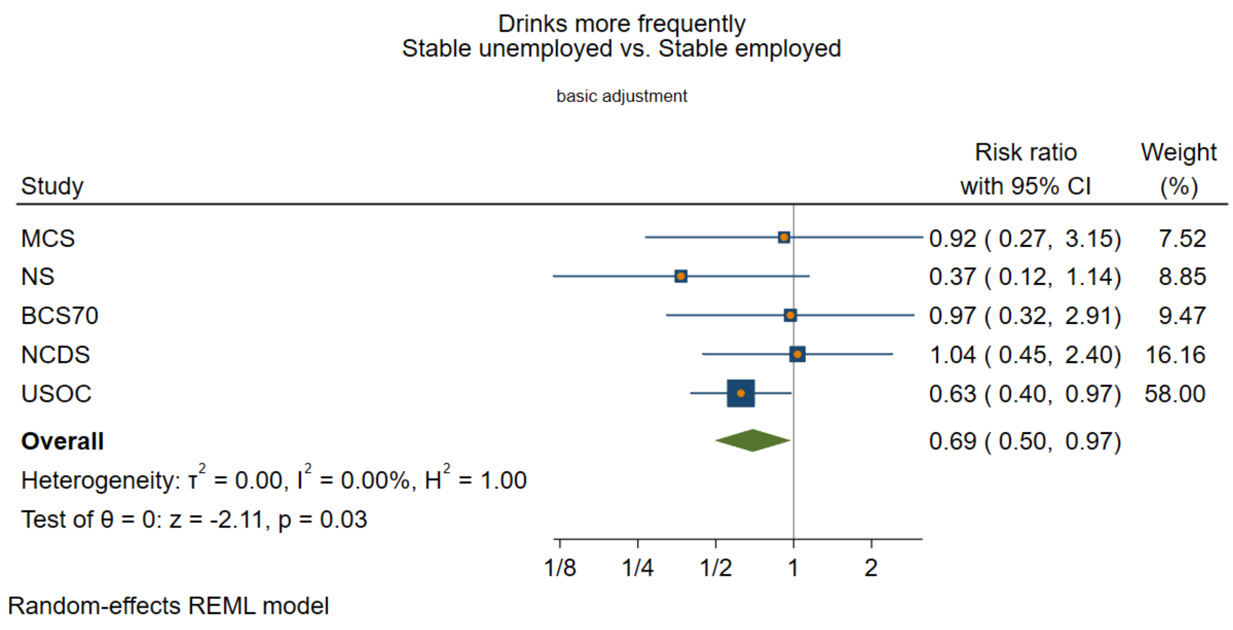
Drinks more frequently
Stable unemployed vs. Stable employed
basic adjustment
Study
Risk ratio with 95% CI
Weight (%)
MCS 0.92 (0.27, 3.15) 7.52
NS 0.37 (0.12, 1.14) 8.85
BCS70 0.97 (0.32, 2.91) 9.47
NCDS 1.04 (0.45, 2.40) 16.16
USOC 0.63 (0.40, 0.97) 58.00
Overall 0.69 (0.50, 0.97)
Heterogeneity: τ² = 0.00, I² = 0.00%, H² = 1.00
Test of θ = 0: z = -2.11, p = 0.03
1/8
1/4
1/2
1
2
Random-effects REML model

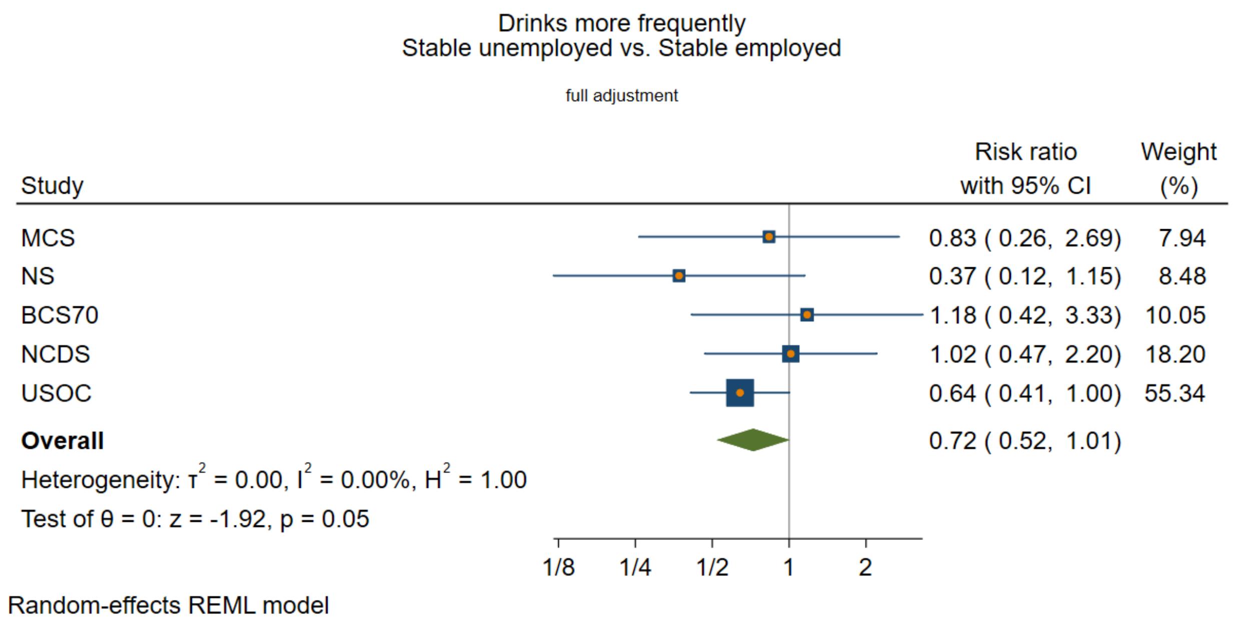
Drinks more frequently
Stable unemployed vs. Stable employed
full adjustment
Study
Risk ratio with 95% CI
Weight (%)
MCS 0.83 (0.26, 2.69) 7.94
NS 0.37 (0.12, 1.15) 8.48
BCS70 1.18 (0.42, 3.33) 10.05
NCDS 1.02 (0.47, 2.20) 18.20
USOC 0.64 (0.41, 1.00) 55.34
Overall 0.72 (0.52, 1.01)
Heterogeneity: τ² = 0.00, I² = 0.00%, H² = 1.00
Test of θ = 0: z = -1.92, p = 0.05
1/8
1/4
1/2
1
2
Random-effects REML model

Figure set 9. Drinks less frequently

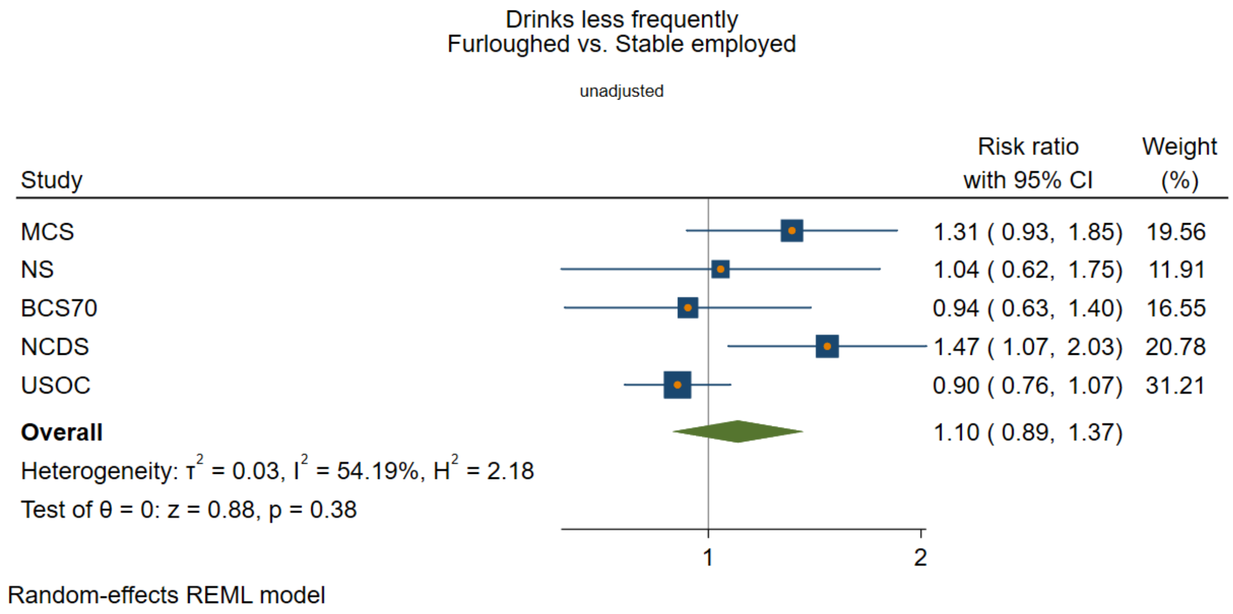

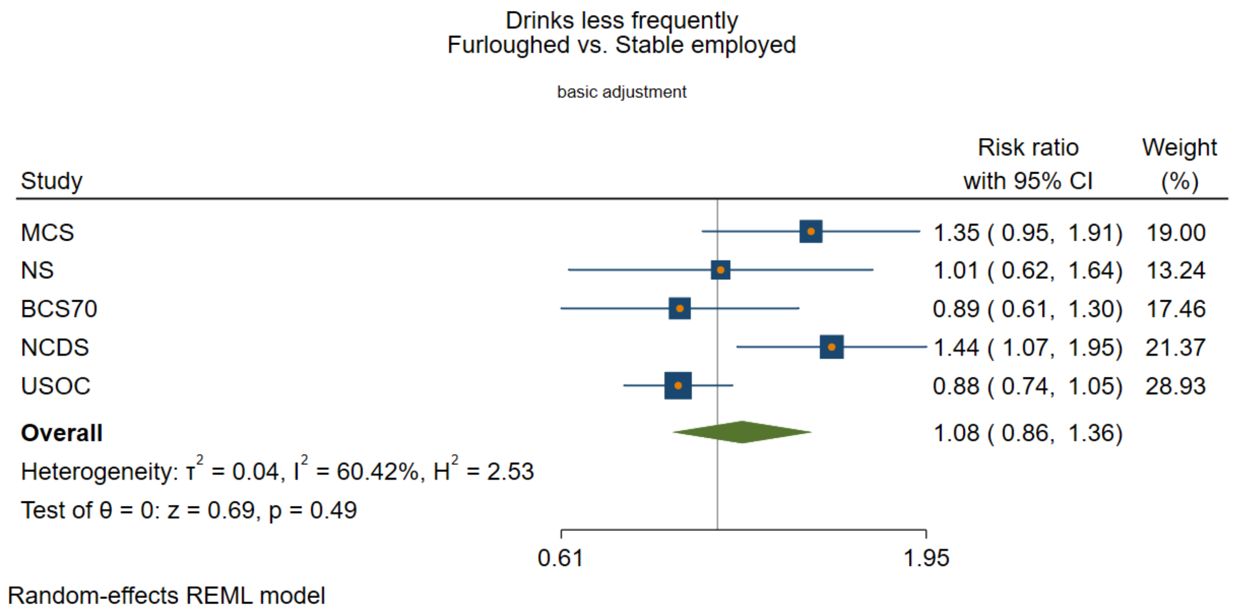

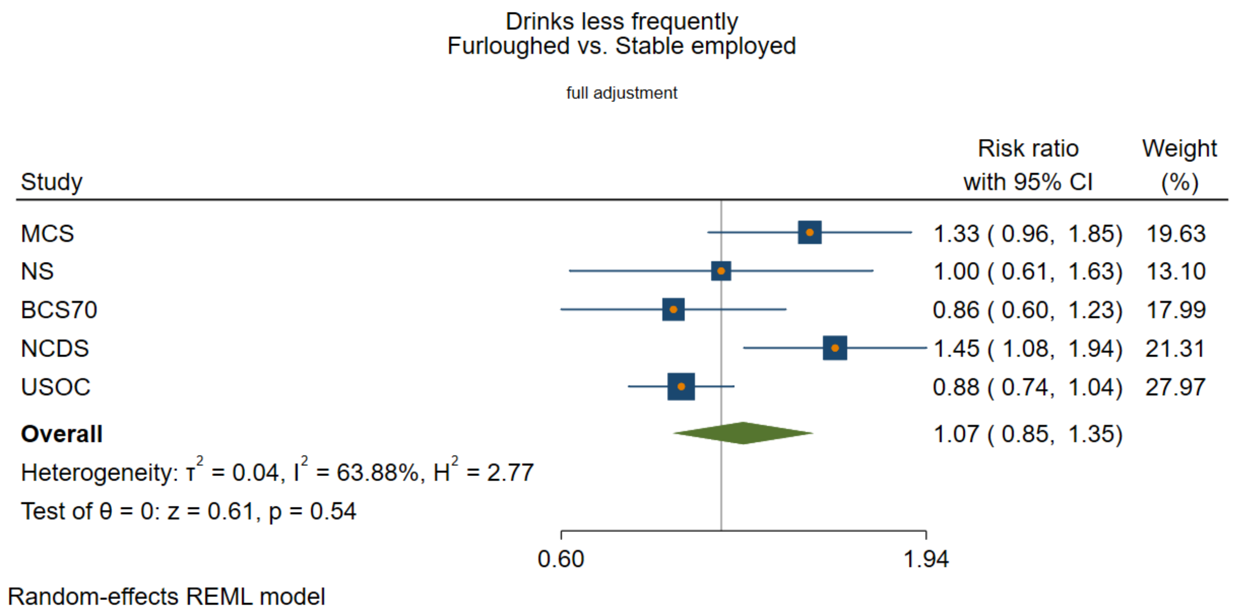

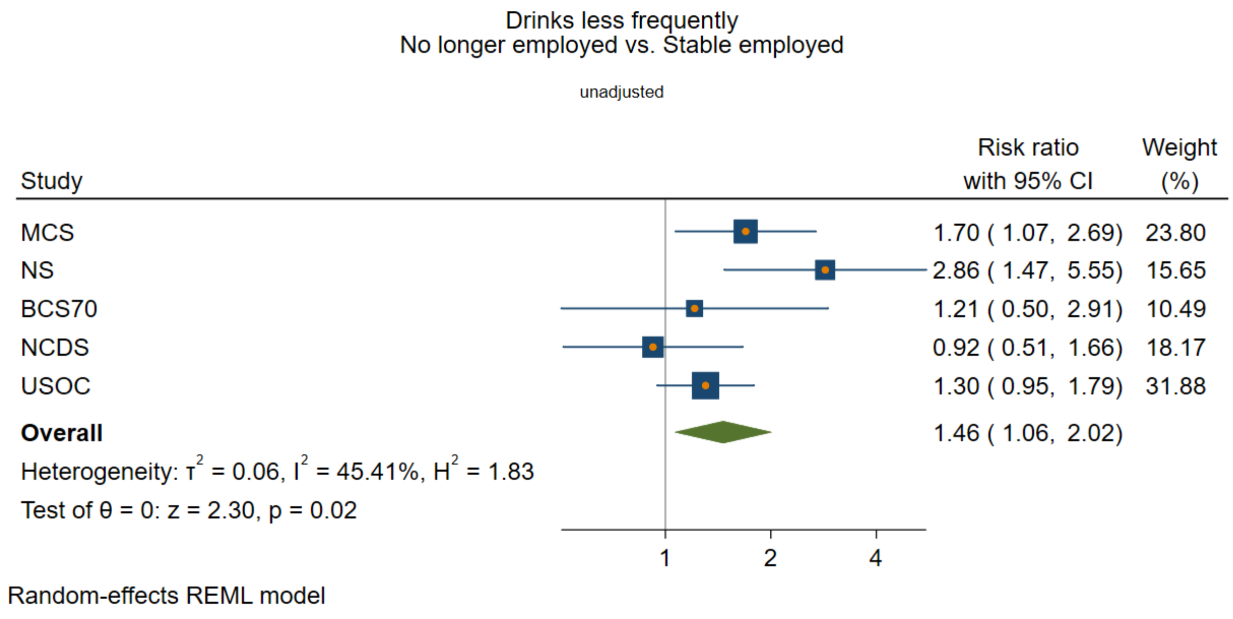
Drinks less frequently
No longer employed vs. Stable employed
unadjusted
Study
Risk ratio with 95% CI
Weight (%)
MCS 1.70 (1.07, 2.69) 23.80
NS 2.86 (1.47, 5.55) 15.65
BCS70 1.21 (0.50, 2.91) 10.49
NCDS 0.92 (0.51, 1.66) 18.17
USOC 1.30 (0.95, 1.79) 31.88
Overall 1.46 (1.06, 2.02)
Heterogeneity: τ² = 0.06, I² = 45.41%, H² = 1.83
Test of θ = 0: z = 2.30, p = 0.02
1
2
4
Random-effects REML model

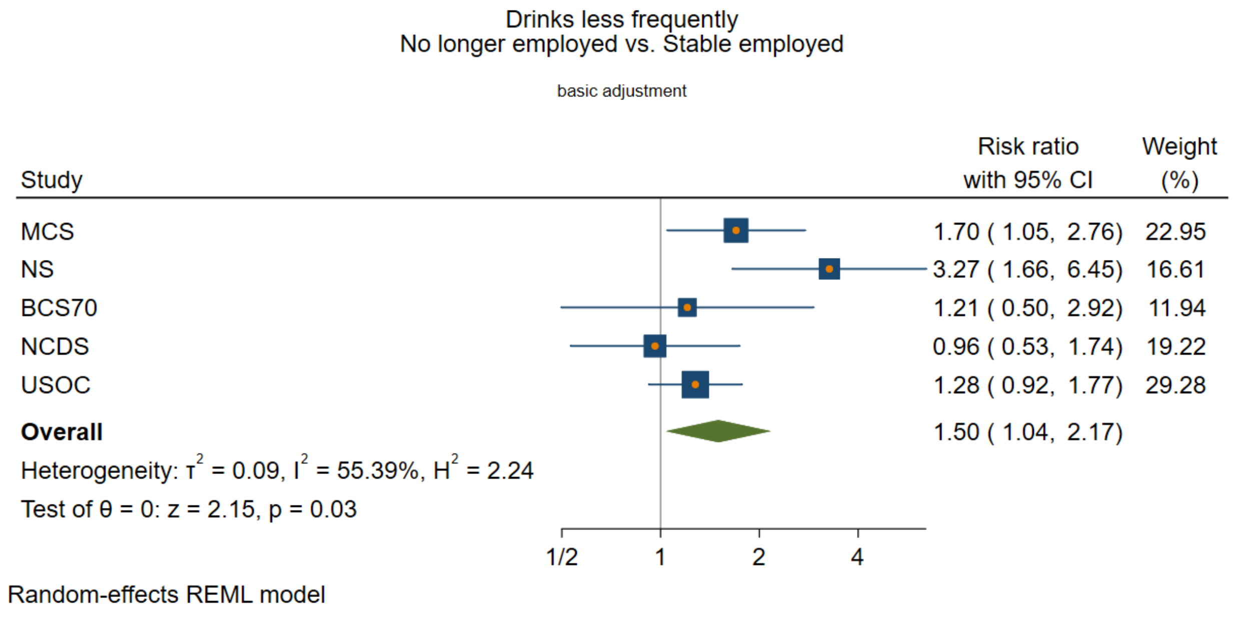
Drinks less frequently
No longer employed vs. Stable employed
basic adjustment
Study
Risk ratio with 95% CI
Weight (%)
MCS 1.70 (1.05, 2.76) 22.95
NS 3.27 (1.66, 6.45) 16.61
BCS70 1.21 (0.50, 2.92) 11.94
NCDS 0.96 (0.53, 1.74) 19.22
USOC 1.28 (0.92, 1.77) 29.28
Overall 1.50 (1.04, 2.17)
Heterogeneity: τ² = 0.09, I² = 55.39%, H² = 2.24
Test of θ = 0: z = 2.15, p = 0.03
1/2
1
2
4
Random-effects REML model

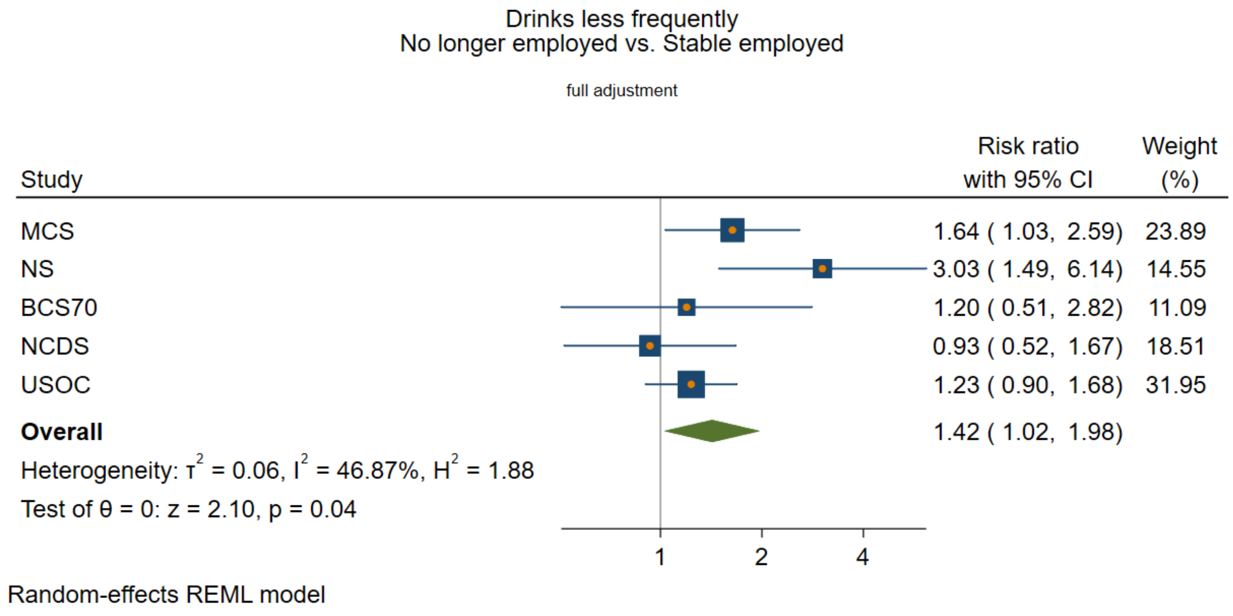
Drinks less frequently
No longer employed vs. Stable employed
full adjustment
Study
Risk ratio with 95% CI
Weight (%)
MCS 1.64 (1.03, 2.59) 23.89
NS 3.03 (1.49, 6.14) 14.55
BCS70 1.20 (0.51, 2.82) 11.09
NCDS 0.93 (0.52, 1.67) 18.51
USOC 1.23 (0.90, 1.68) 31.95
Overall 1.42 (1.02, 1.98)
Heterogeneity: τ² = 0.06, I² = 46.87%, H² = 1.88
Test of θ = 0: z = 2.10, p = 0.04
1
2
4
Random-effects REML model

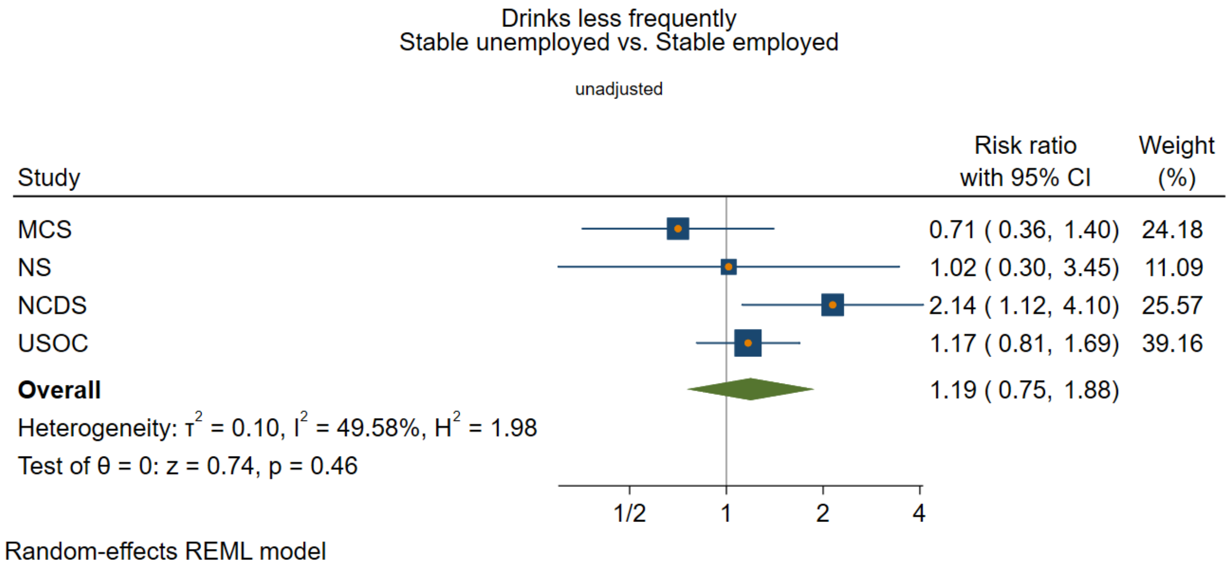
Drinks less frequently
Stable unemployed vs. Stable employed
unadjusted
Study
Risk ratio with 95% CI
Weight (%)
MCS 0.71 (0.36, 1.40) 24.18
NS 1.02 (0.30, 3.45) 11.09
NCDS 2.14 (1.12, 4.10) 25.57
USOC 1.17 (0.81, 1.69) 39.16
Overall 1.19 (0.75, 1.88)
Heterogeneity: τ² = 0.10, I² = 49.58%, H² = 1.98
Test of θ = 0: z = 0.74, p = 0.46
1/2
1
2
4
Random-effects REML model

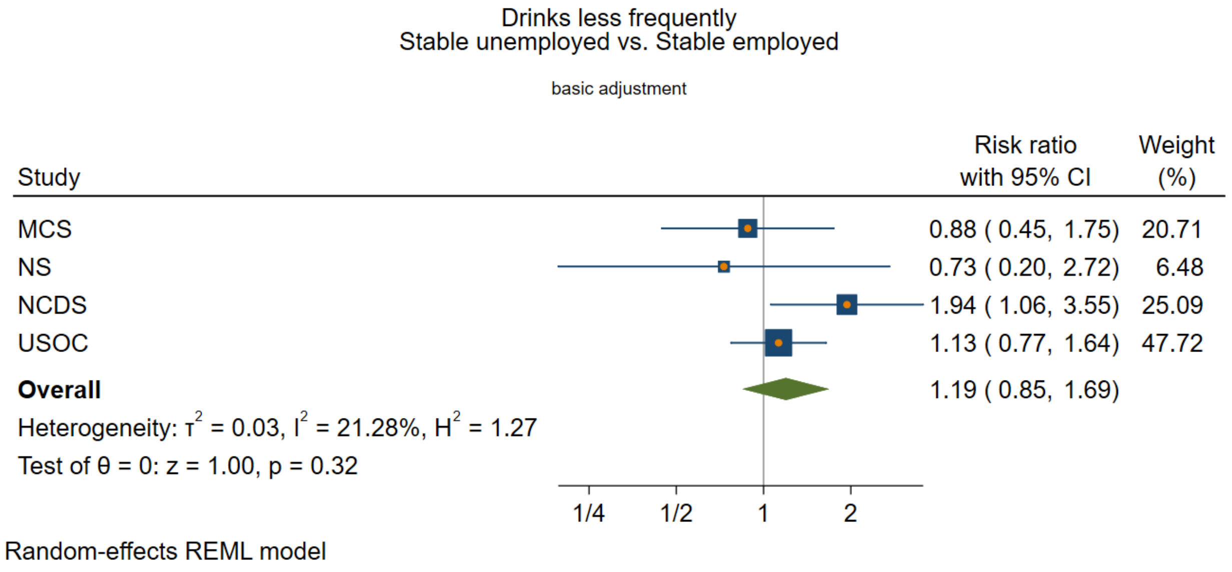
Drinks less frequently
Stable unemployed vs. Stable employed
basic adjustment
Study
Risk ratio with 95% CI
Weight (%)
MCS 0.88 (0.45, 1.75) 20.71
NS 0.73 (0.20, 2.72) 6.48
NCDS 1.94 (1.06, 3.55) 25.09
USOC 1.13 (0.77, 1.64) 47.72
Overall 1.19 (0.85, 1.69)
Heterogeneity: τ² = 0.03, I² = 21.28%, H² = 1.27
Test of θ = 0: z = 1.00, p = 0.32
1/4
1/2
1
2
Random-effects REML model

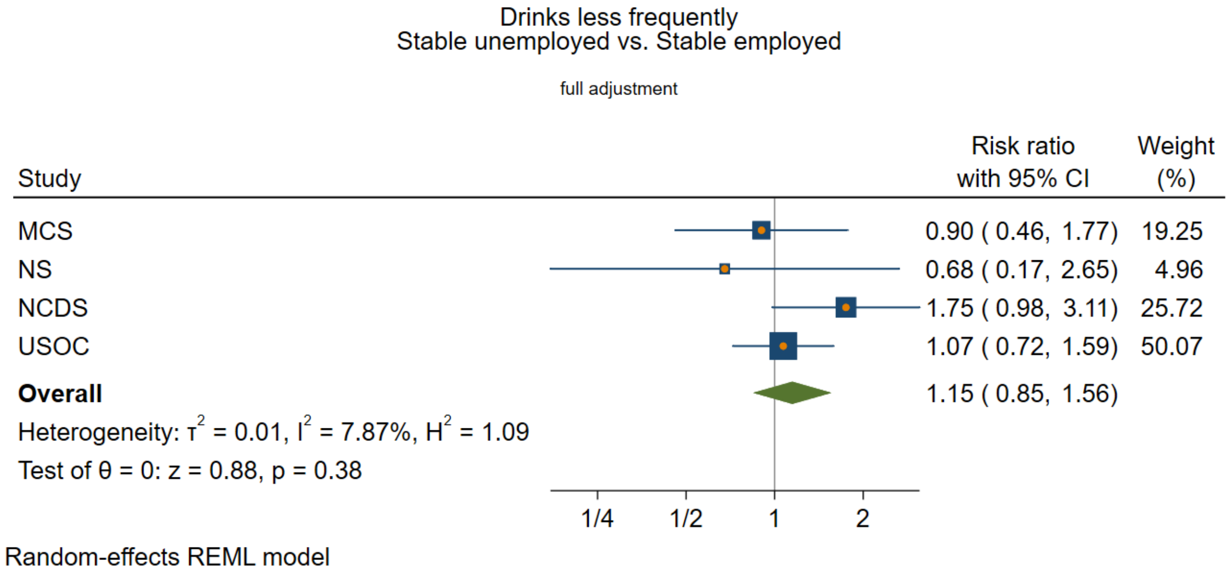
Drinks less frequently
Stable unemployed vs. Stable employed
full adjustment
Study
Risk ratio with 95% CI
Weight (%)
MCS 0.90 (0.46, 1.77) 19.25
NS 0.68 (0.17, 2.65) 4.96
NCDS 1.75 (0.98, 3.11) 25.72
USOC 1.07 (0.72, 1.59) 50.07
Overall 1.15 (0.85, 1.56)
Heterogeneity: τ² = 0.01, I² = 7.87%, H² = 1.09
Test of θ = 0: z = 0.88, p = 0.38
1/4
1/2
1
2
Random-effects REML model

Figure set 10. Current smoker

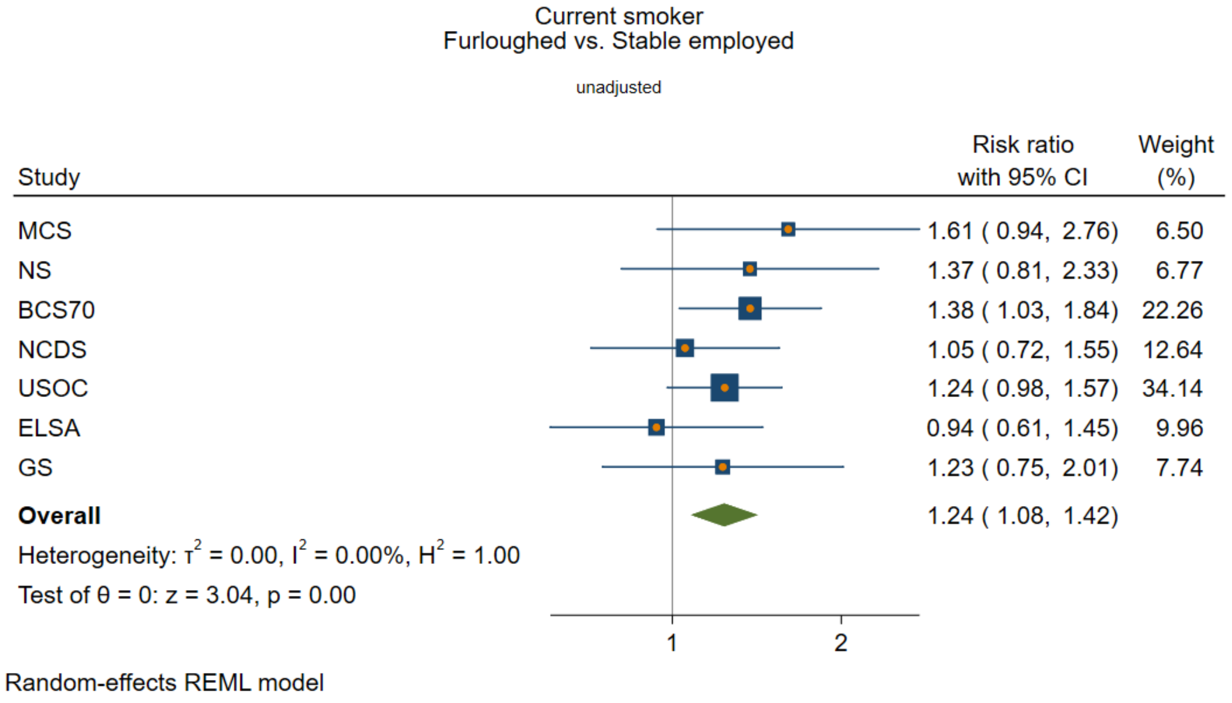

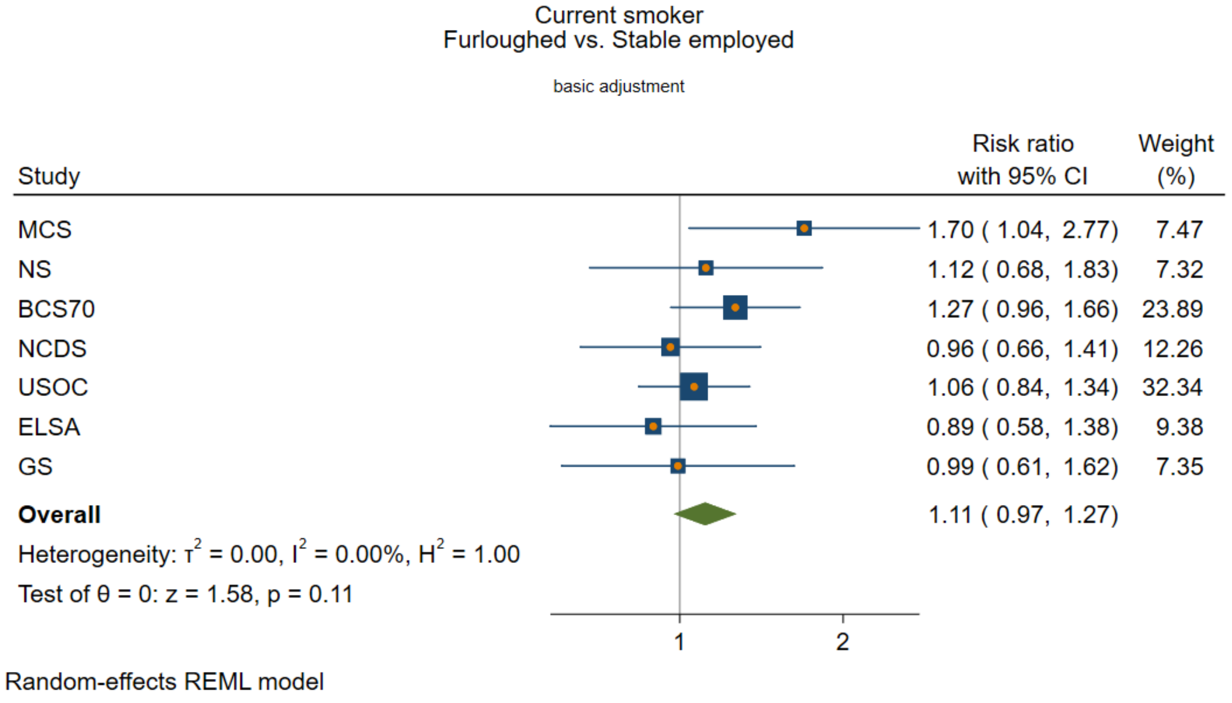

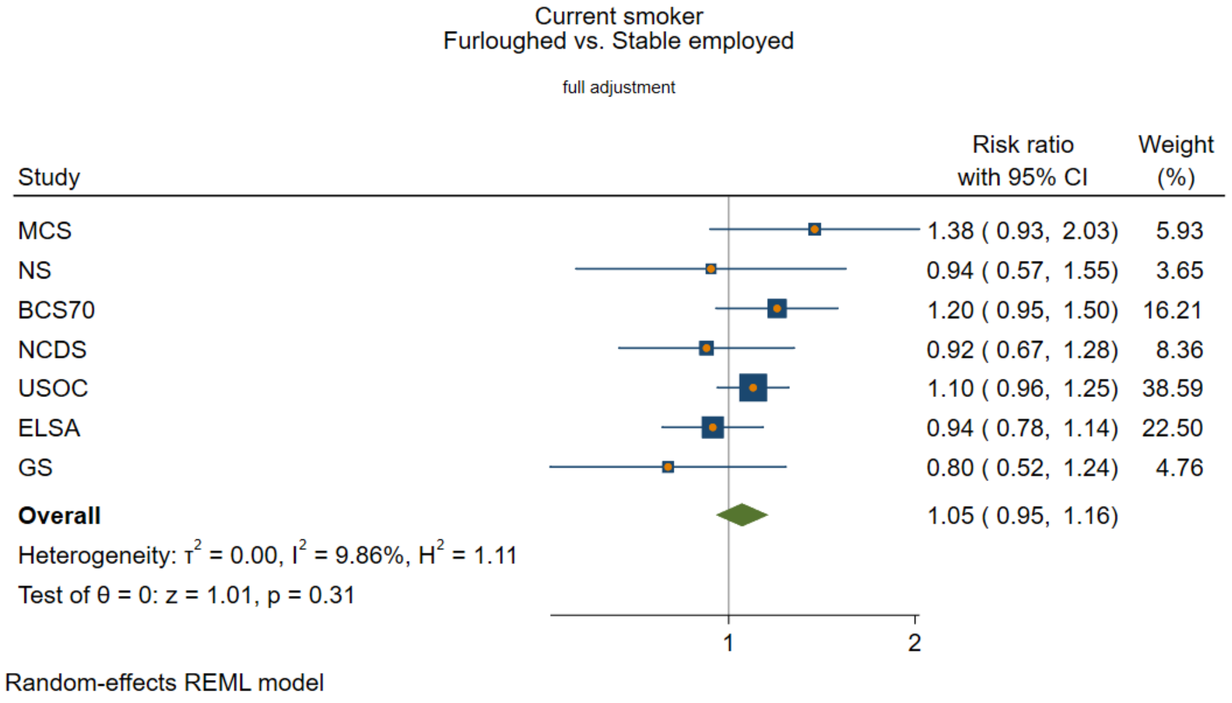

Current smoker
No longer employed vs. Stable employed

unadjusted

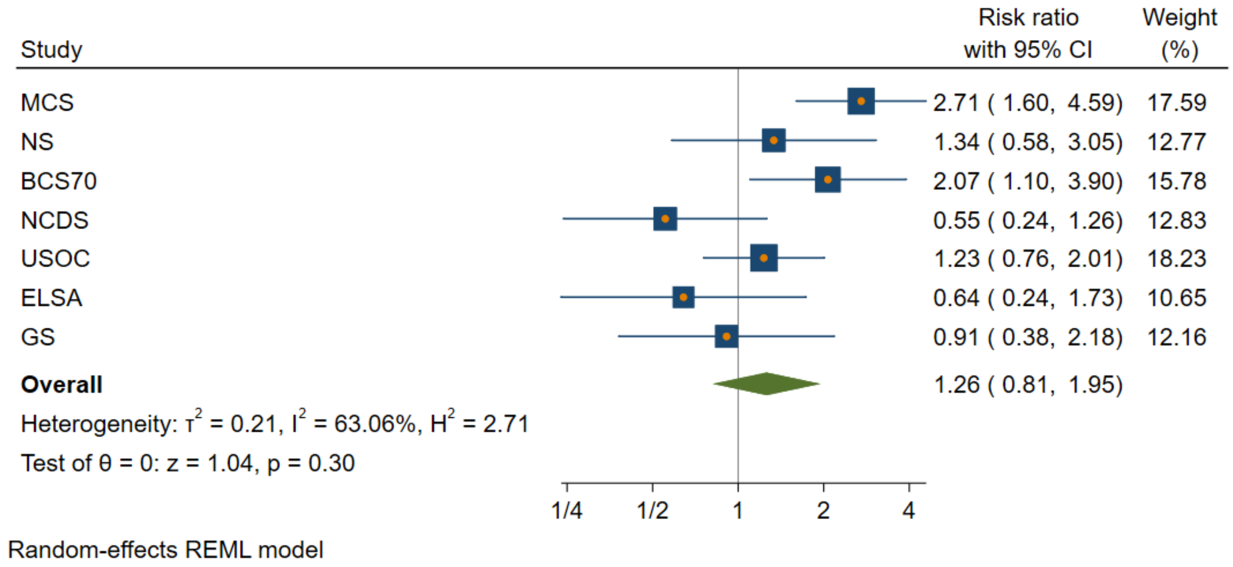

Current smoker
No longer employed vs. Stable employed

basic adjustment

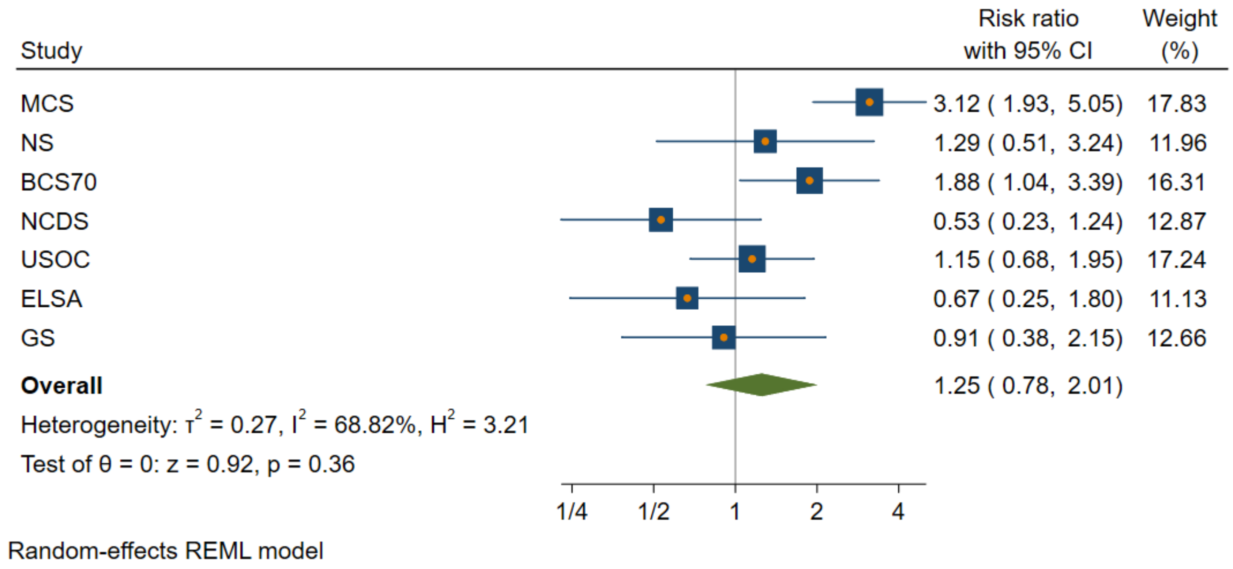

Current smoker
No longer employed vs. Stable employed

full adjustment

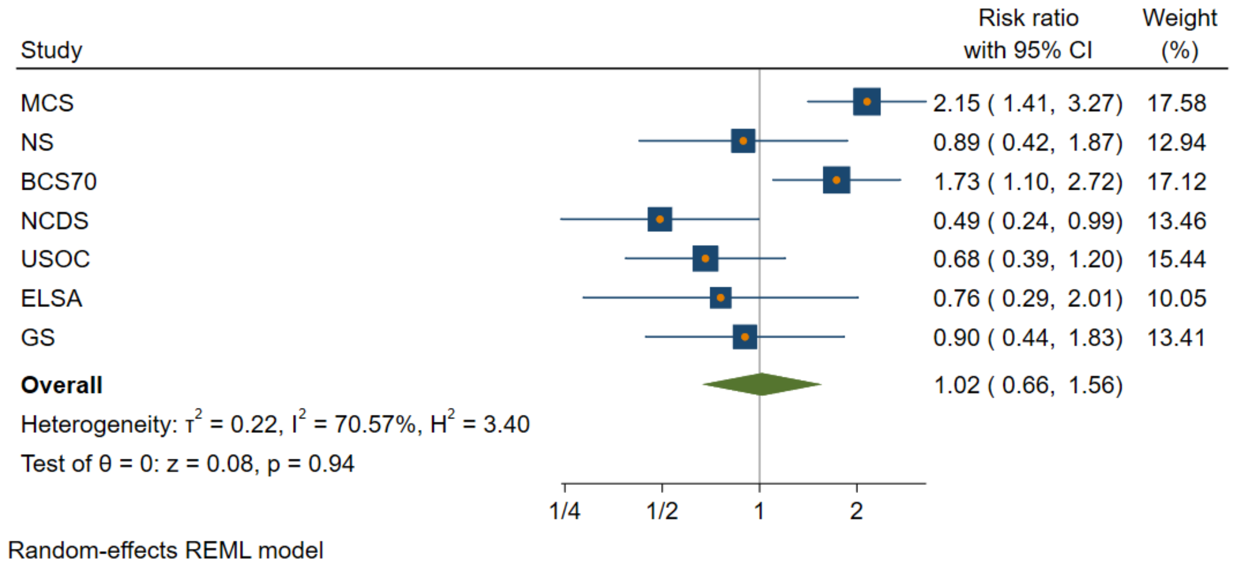

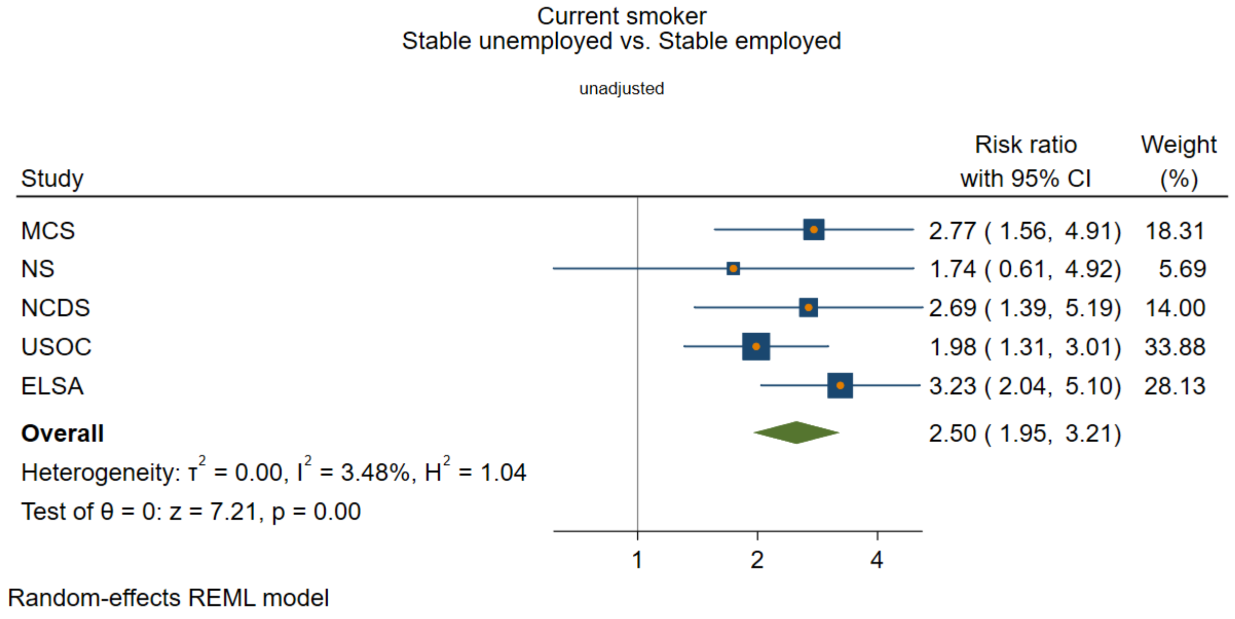
Current smoker
Stable unemployed vs. Stable employed
unadjusted
Study
Risk ratio with 95% CI
Weight (%)
MCS 2.77 (1.56, 4.91) 18.31
NS 1.74 (0.61, 4.92) 5.69
NCDS 2.69 (1.39, 5.19) 14.00
USOC 1.98 (1.31, 3.01) 33.88
ELSA 3.23 (2.04, 5.10) 28.13
Overall 2.50 (1.95, 3.21)
Heterogeneity: τ² = 0.00, I² = 3.48%, H² = 1.04
Test of θ = 0: z = 7.21, p = 0.00
1
2
4
Random-effects REML model

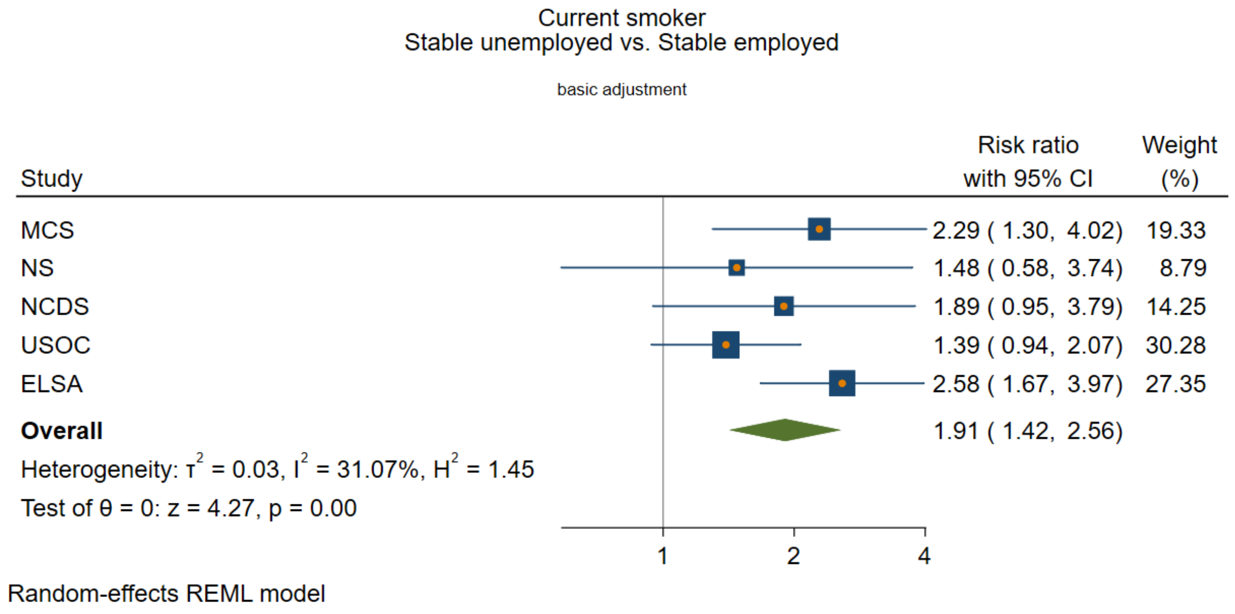
Current smoker
Stable unemployed vs. Stable employed
basic adjustment
Study
Risk ratio with 95% CI
Weight (%)
MCS 2.29 (1.30, 4.02) 19.33
NS 1.48 (0.58, 3.74) 8.79
NCDS 1.89 (0.95, 3.79) 14.25
USOC 1.39 (0.94, 2.07) 30.28
ELSA 2.58 (1.67, 3.97) 27.35
Overall 1.91 (1.42, 2.56)
Heterogeneity: τ² = 0.03, I² = 31.07%, H² = 1.45
Test of θ = 0: z = 4.27, p = 0.00
1
2
4
Random-effects REML model

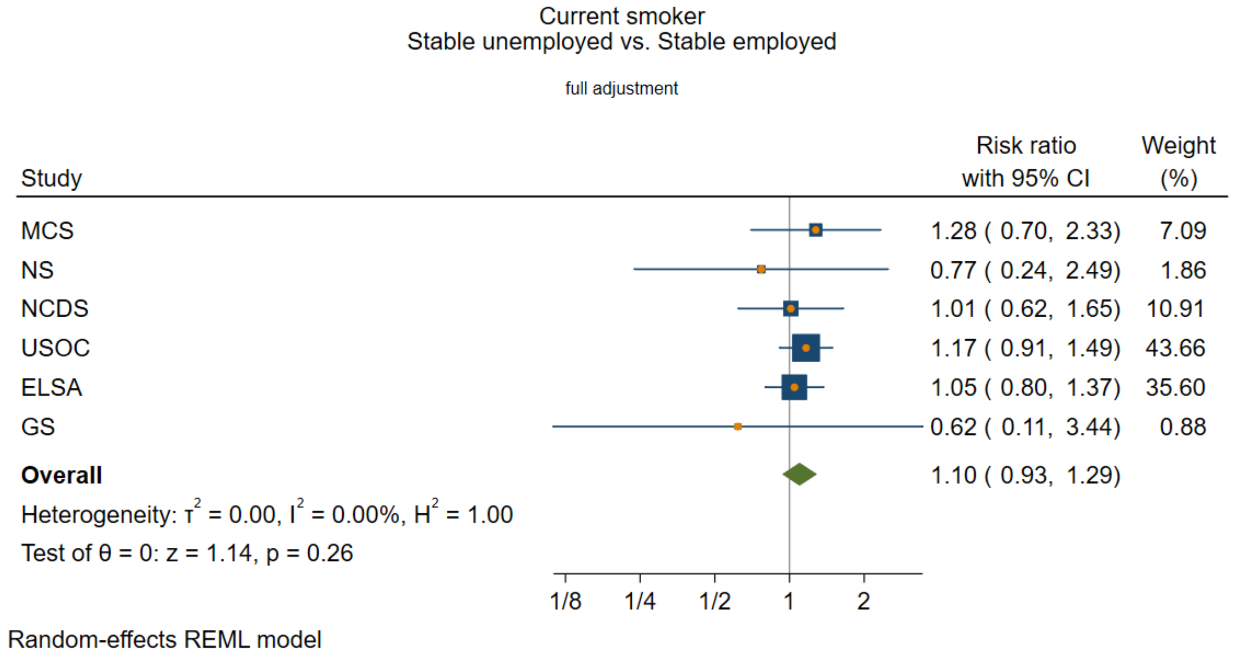
Current smoker
Stable unemployed vs. Stable employed
full adjustment
Study
Risk ratio with 95% CI
Weight (%)
MCS 1.28 (0.70, 2.33) 7.09
NS 0.77 (0.24, 2.49) 1.86
NCDS 1.01 (0.62, 1.65) 10.91
USOC 1.17 (0.91, 1.49) 43.66
ELSA 1.05 (0.80, 1.37) 35.60
GS 0.62 (0.11, 3.44) 0.88
Overall 1.10 (0.93, 1.29)
Heterogeneity: τ² = 0.00, I² = 0.00%, H² = 1.00
Test of θ = 0: z = 1.14, p = 0.26
1/8
1/4
1/2
1
2
Random-effects REML model

Figure set 11. Smoking more

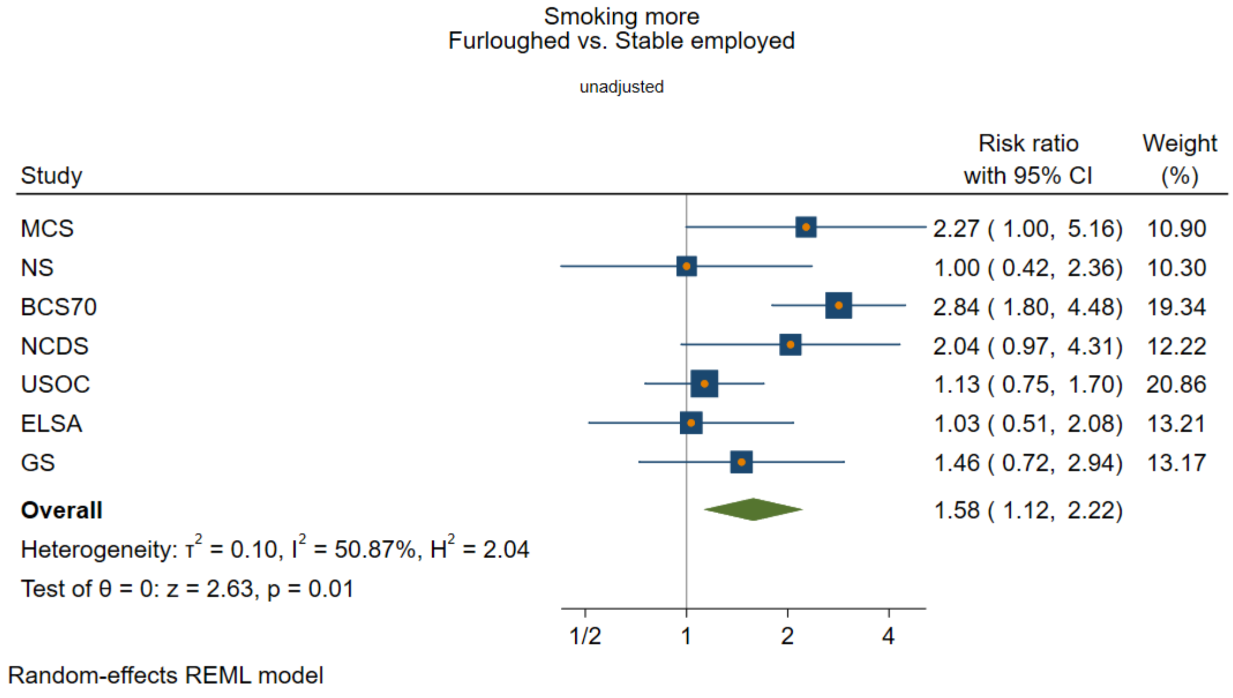

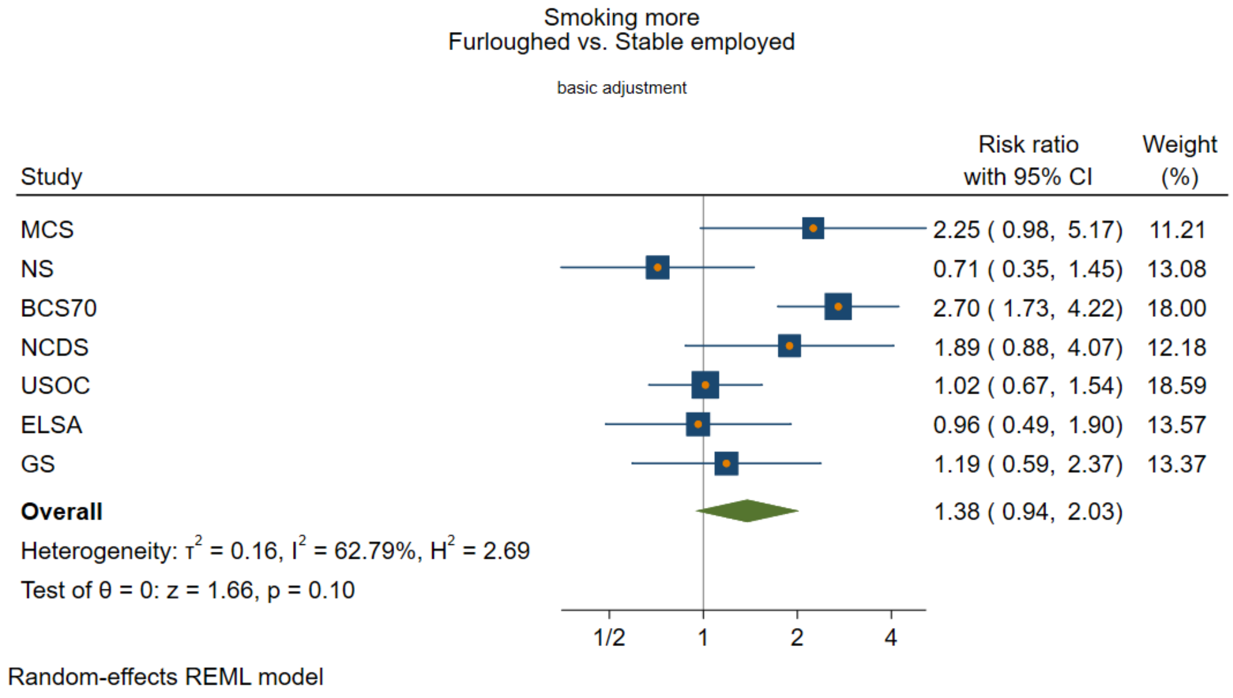

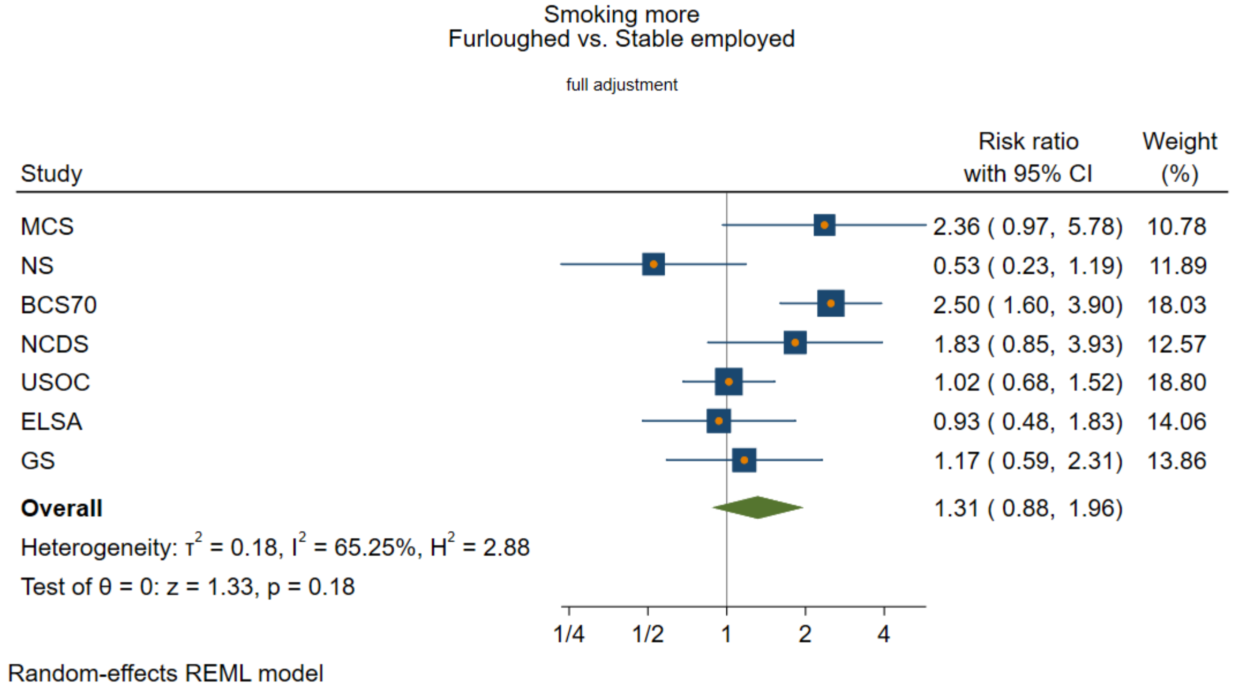

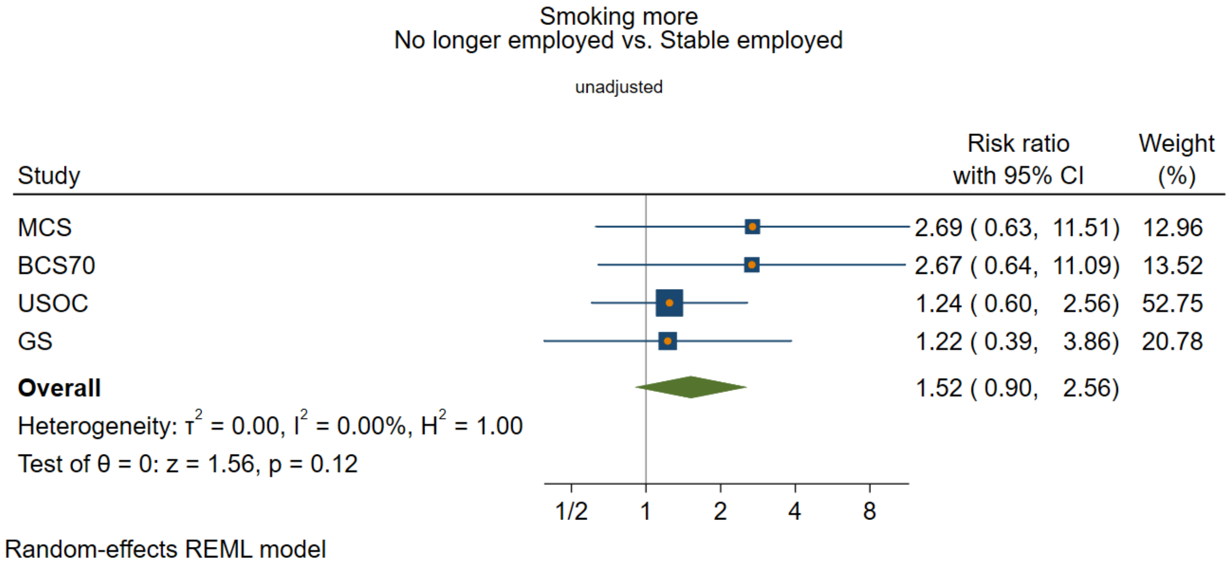
Smoking more
No longer employed vs. Stable employed
unadjusted
Study
Risk ratio with 95% CI
Weight (%)
MCS 2.69 (0.63, 11.51) 12.96
BCS70 2.67 (0.64, 11.09) 13.52
USOC 1.24 (0.60, 2.56) 52.75
GS 1.22 (0.39, 3.86) 20.78
Overall 1.52 (0.90, 2.56)
Heterogeneity: τ² = 0.00, I² = 0.00%, H² = 1.00
Test of θ = 0: z = 1.56, p = 0.12
1/2
1
2
4
8
Random-effects REML model

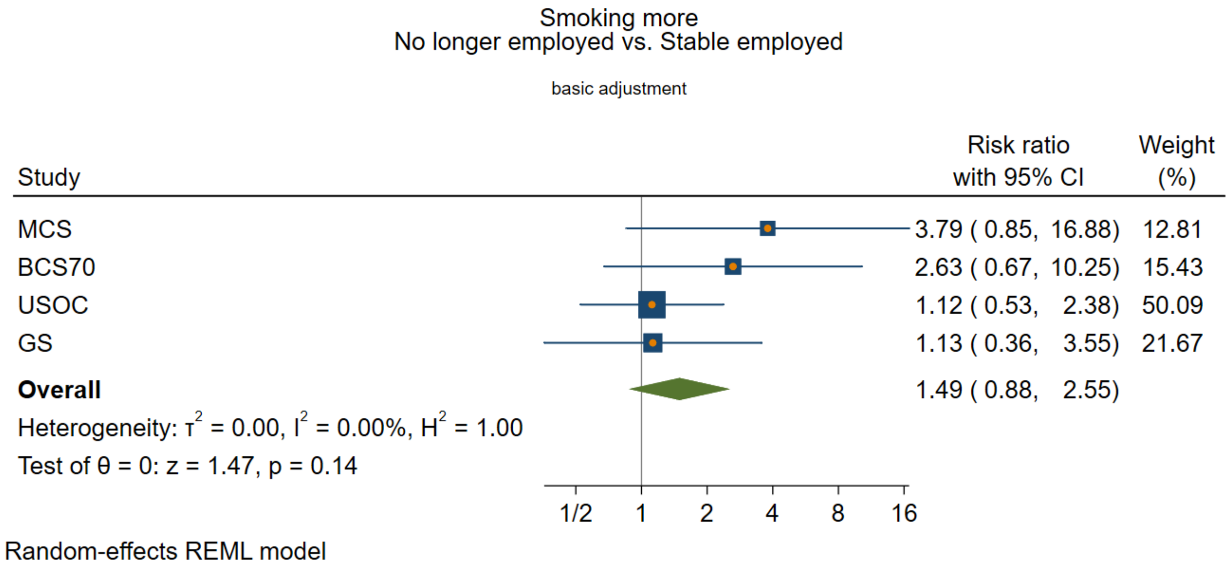
Smoking more
No longer employed vs. Stable employed
basic adjustment
Study
Risk ratio with 95% CI
Weight (%)
MCS 3.79 (0.85, 16.88) 12.81
BCS70 2.63 (0.67, 10.25) 15.43
USOC 1.12 (0.53, 2.38) 50.09
GS 1.13 (0.36, 3.55) 21.67
Overall 1.49 (0.88, 2.55)
Heterogeneity: τ² = 0.00, I² = 0.00%, H² = 1.00
Test of θ = 0: z = 1.47, p = 0.14
1/2
1
2
4
8
16
Random-effects REML model

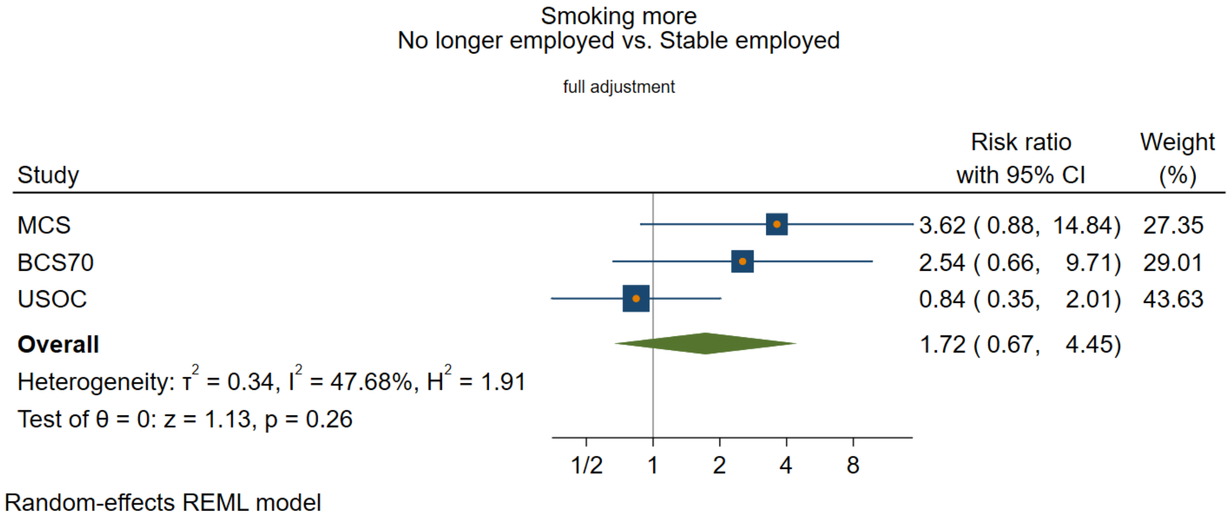
Smoking more
No longer employed vs. Stable employed
full adjustment
Study
Risk ratio with 95% CI
Weight (%)
MCS 3.62 (0.88, 14.84) 27.35
BCS70 2.54 (0.66, 9.71) 29.01
USOC 0.84 (0.35, 2.01) 43.63
Overall 1.72 (0.67, 4.45)
Heterogeneity: τ² = 0.34, I² = 47.68%, H² = 1.91
Test of θ = 0: z = 1.13, p = 0.26
1/2
1
2
4
8
Random-effects REML model

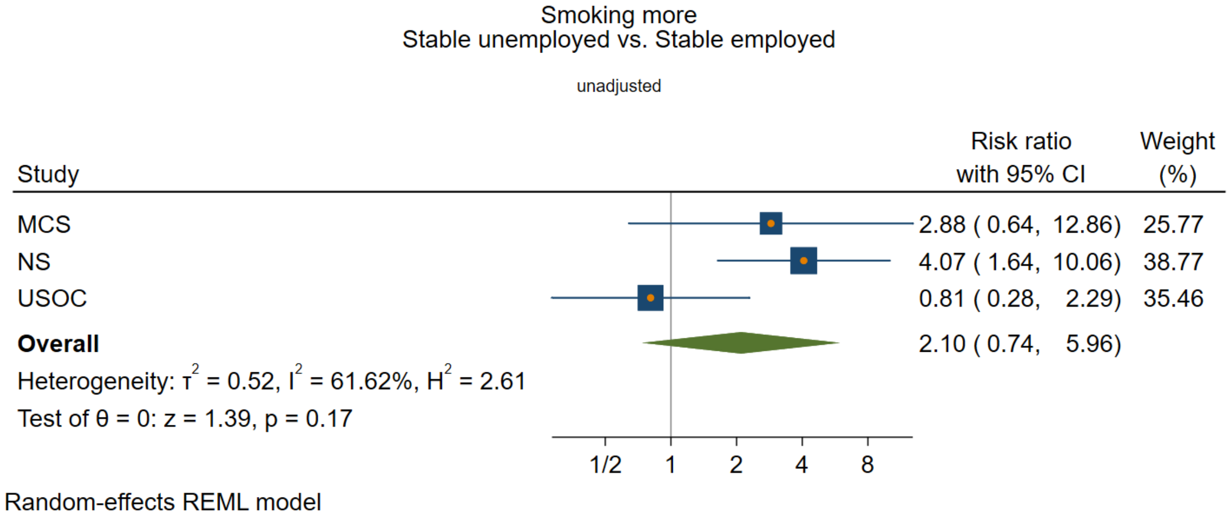
Smoking more
Stable unemployed vs. Stable employed
unadjusted
Study
Risk ratio with 95% CI
Weight (%)
MCS 2.88 (0.64, 12.86) 25.77
NS 4.07 (1.64, 10.06) 38.77
USOC 0.81 (0.28, 2.29) 35.46
Overall 2.10 (0.74, 5.96)
Heterogeneity: τ² = 0.52, I² = 61.62%, H² = 2.61
Test of θ = 0: z = 1.39, p = 0.17
1/2 1 2 4 8
Random-effects REML model

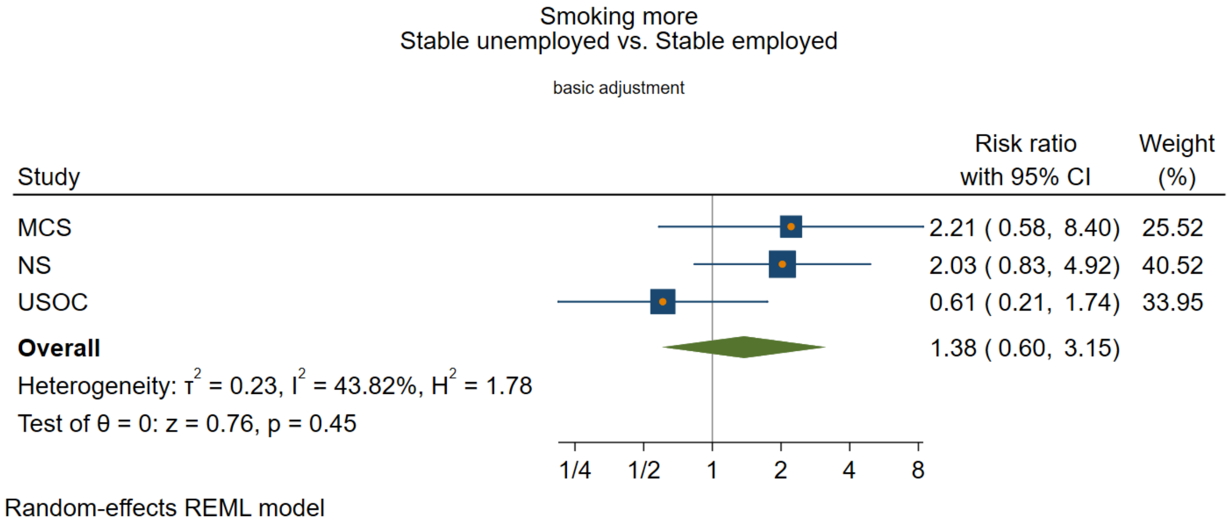
Smoking more
Stable unemployed vs. Stable employed
basic adjustment
Study
Risk ratio with 95% CI
Weight (%)
MCS 2.21 (0.58, 8.40) 25.52
NS 2.03 (0.83, 4.92) 40.52
USOC 0.61 (0.21, 1.74) 33.95
Overall 1.38 (0.60, 3.15)
Heterogeneity: τ² = 0.23, I² = 43.82%, H² = 1.78
Test of θ = 0: z = 0.76, p = 0.45
1/4 1/2 1 2 4 8
Random-effects REML model

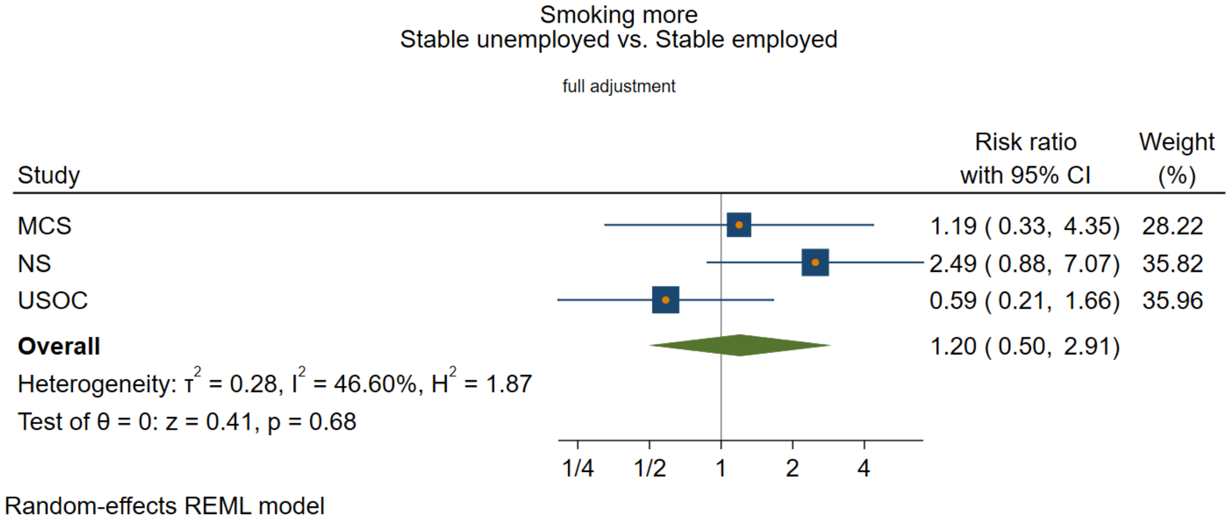
Smoking more
Stable unemployed vs. Stable employed
full adjustment
Study
Risk ratio with 95% CI
Weight (%)
MCS 1.19 (0.33, 4.35) 28.22
NS 2.49 (0.88, 7.07) 35.82
USOC 0.59 (0.21, 1.66) 35.96
Overall 1.20 (0.50, 2.91)
Heterogeneity: τ² = 0.28, I² = 46.60%, H² = 1.87
Test of θ = 0: z = 0.41, p = 0.68
1/4 1/2 1 2 4
Random-effects REML model

Figure set 12. Smoking less

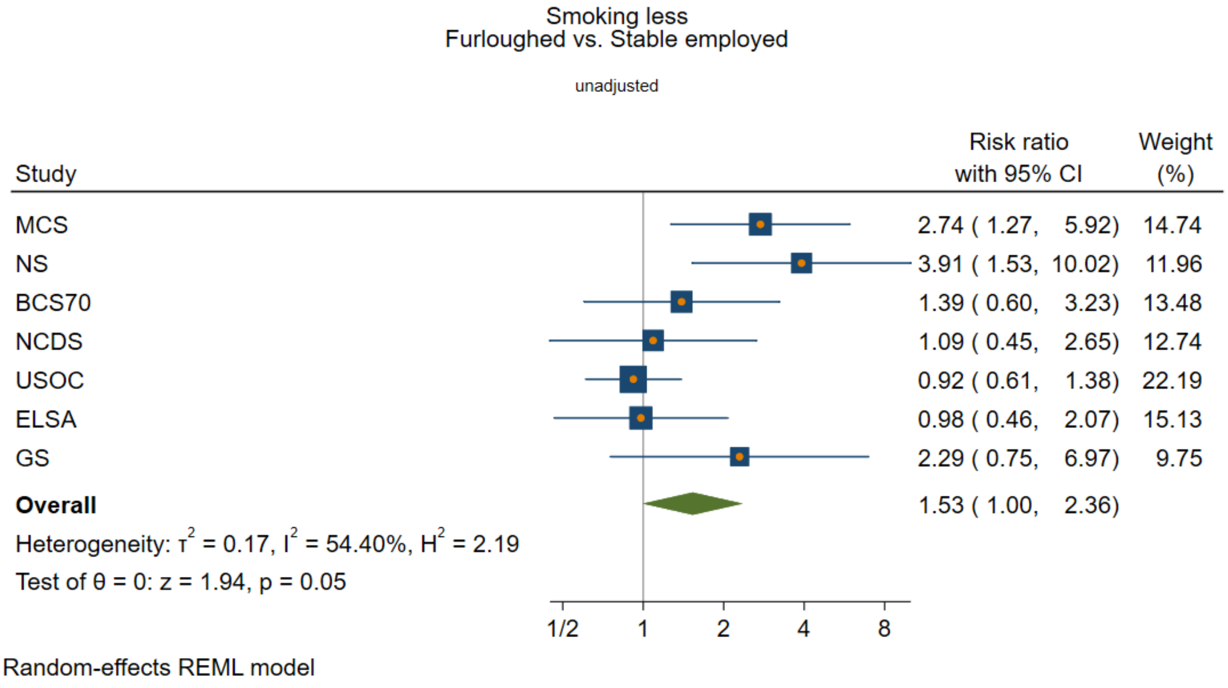

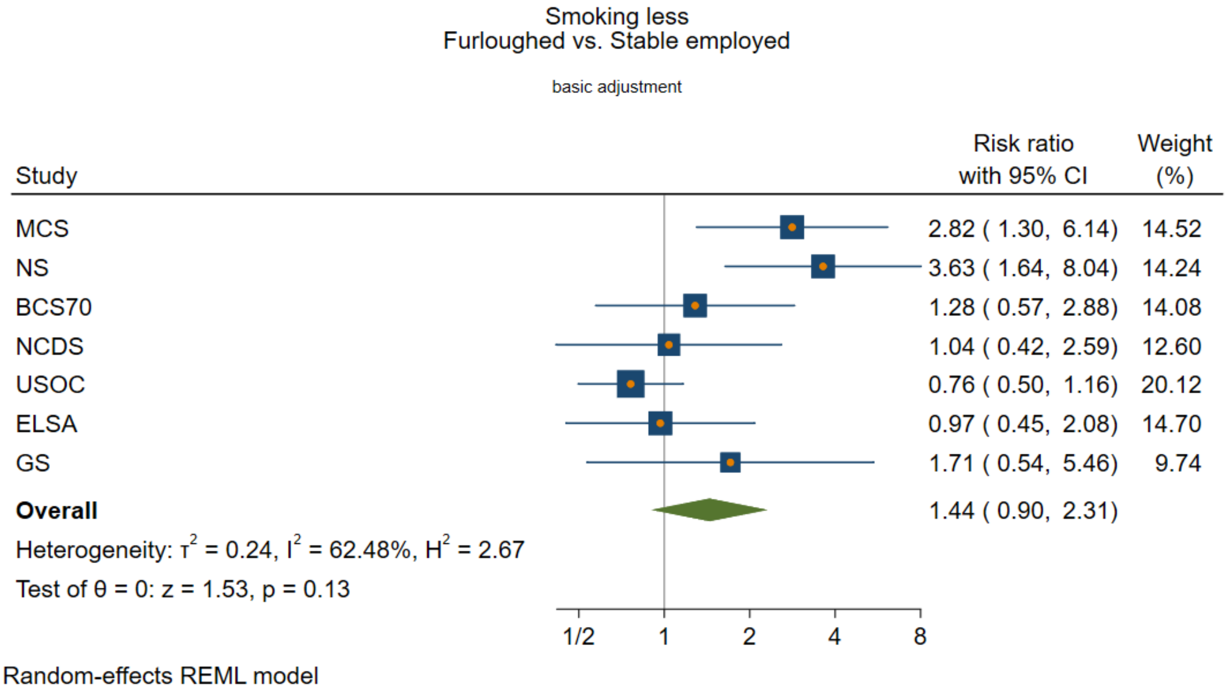

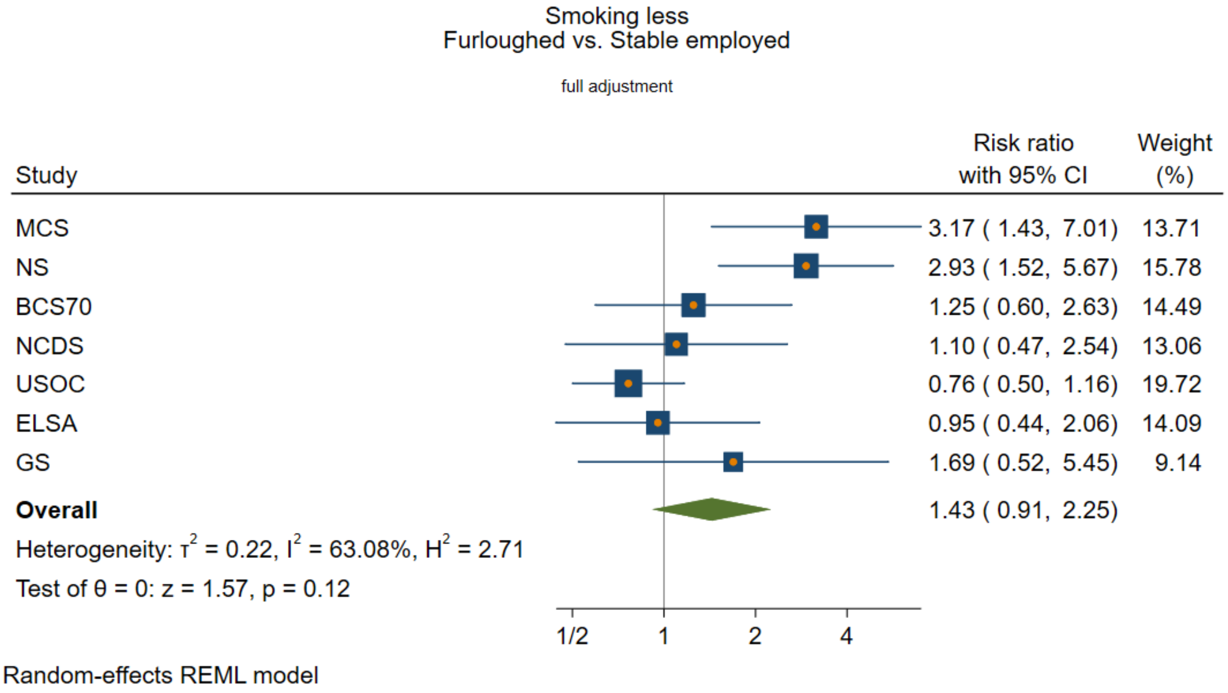

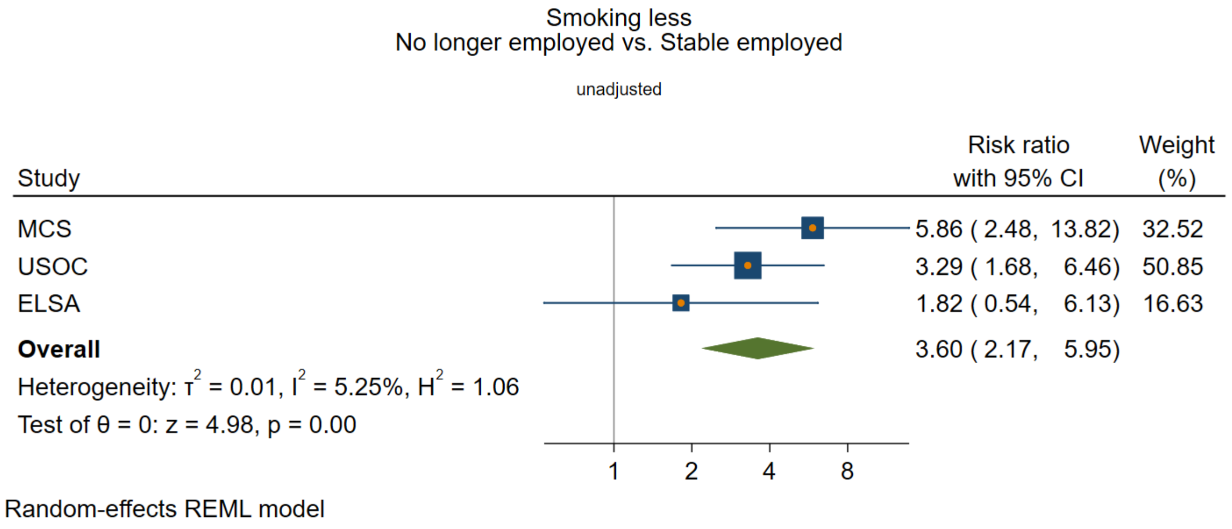

Smoking less
No longer employed vs. Stable employed
unadjusted
Study
Risk ratio with 95% CI
Weight (%)
MCS 5.86 (2.48, 13.82) 32.52
USOC 3.29 (1.68, 6.46) 50.85
ELSA 1.82 (0.54, 6.13) 16.63
Overall 3.60 (2.17, 5.95)
Heterogeneity: τ^2 = 0.01, I^2 = 5.25%, H^2 = 1.06
Test of θ = 0: z = 4.98, p = 0.00
1 2 4 8
Random-effects REML model

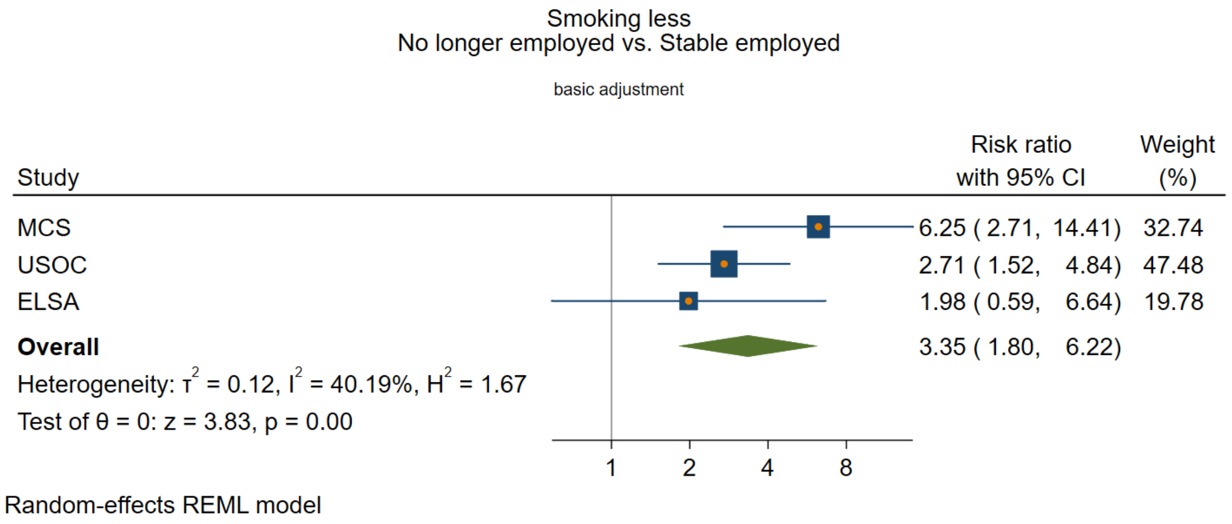

Smoking less
No longer employed vs. Stable employed
basic adjustment
Study
Risk ratio with 95% CI
Weight (%)
MCS 6.25 (2.71, 14.41) 32.74
USOC 2.71 (1.52, 4.84) 47.48
ELSA 1.98 (0.59, 6.64) 19.78
Overall 3.35 (1.80, 6.22)
Heterogeneity: τ^2 = 0.12, I^2 = 40.19%, H^2 = 1.67
Test of θ = 0: z = 3.83, p = 0.00
1 2 4 8
Random-effects REML model

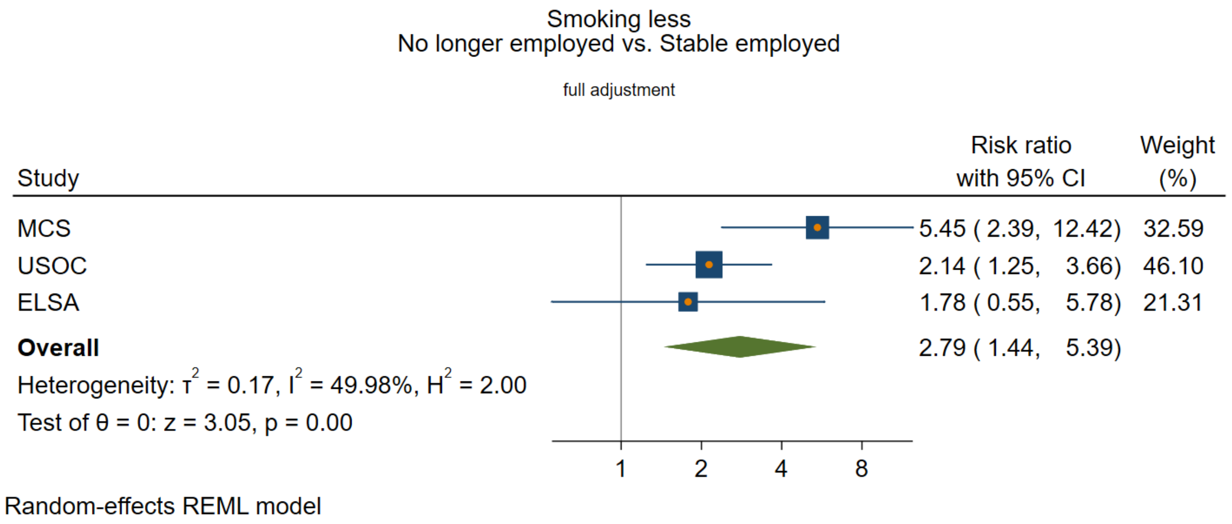

Smoking less
No longer employed vs. Stable employed
full adjustment
Study
Risk ratio with 95% CI
Weight (%)
MCS 5.45 (2.39, 12.42) 32.59
USOC 2.14 (1.25, 3.66) 46.10
ELSA 1.78 (0.55, 5.78) 21.31
Overall 2.79 (1.44, 5.39)
Heterogeneity: τ^2 = 0.17, I^2 = 49.98%, H^2 = 2.00
Test of θ = 0: z = 3.05, p = 0.00
1 2 4 8
Random-effects REML model

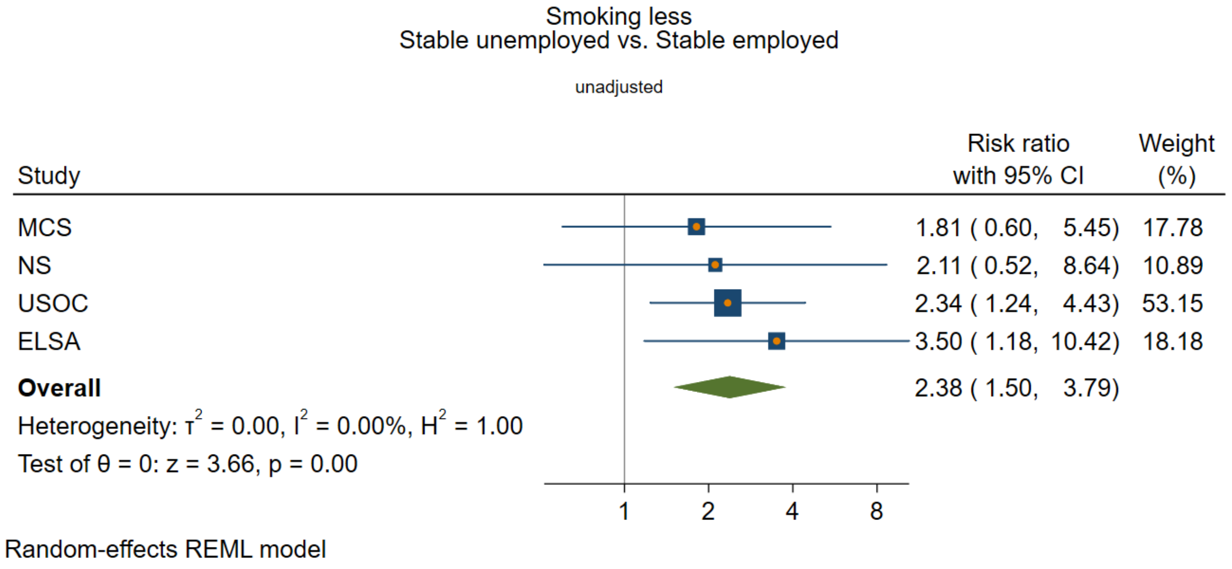
Smoking less
Stable unemployed vs. Stable employed
unadjusted
Study
Risk ratio with 95% CI
Weight (%)
MCS 1.81 (0.60, 5.45) 17.78
NS 2.11 (0.52, 8.64) 10.89
USOC 2.34 (1.24, 4.43) 53.15
ELSA 3.50 (1.18, 10.42) 18.18
Overall 2.38 (1.50, 3.79)
Heterogeneity: τ² = 0.00, I² = 0.00%, H² = 1.00
Test of θ = 0: z = 3.66, p = 0.00
1 2 4 8
Random-effects REML model

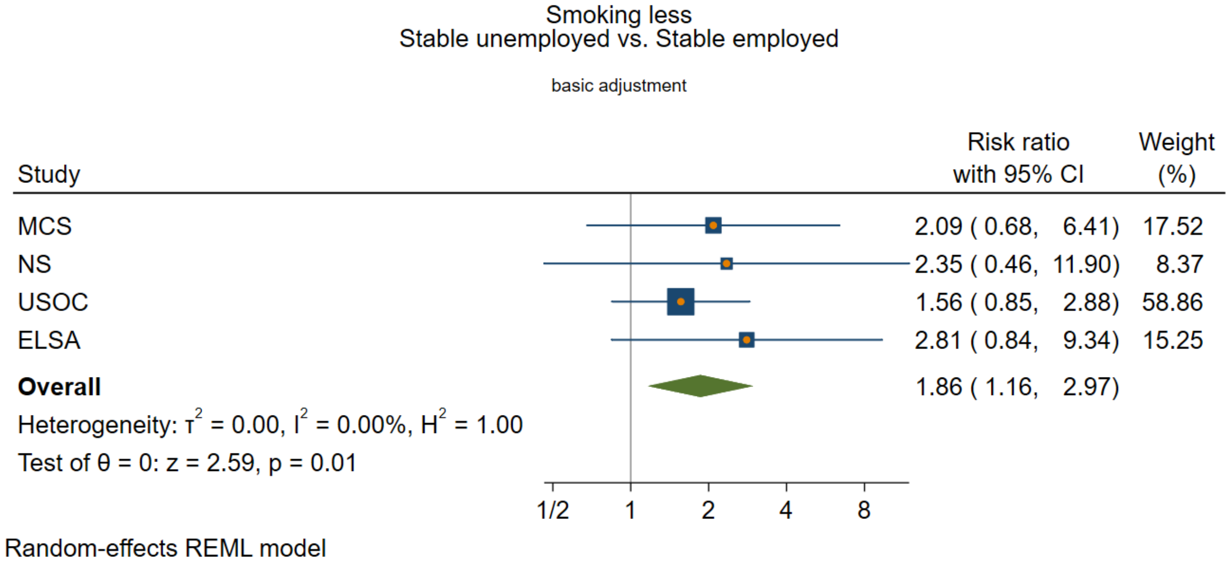
Smoking less
Stable unemployed vs. Stable employed
basic adjustment
Study
Risk ratio with 95% CI
Weight (%)
MCS 2.09 (0.68, 6.41) 17.52
NS 2.35 (0.46, 11.90) 8.37
USOC 1.56 (0.85, 2.88) 58.86
ELSA 2.81 (0.84, 9.34) 15.25
Overall 1.86 (1.16, 2.97)
Heterogeneity: τ² = 0.00, I² = 0.00%, H² = 1.00
Test of θ = 0: z = 2.59, p = 0.01
1/2 1 2 4 8
Random-effects REML model

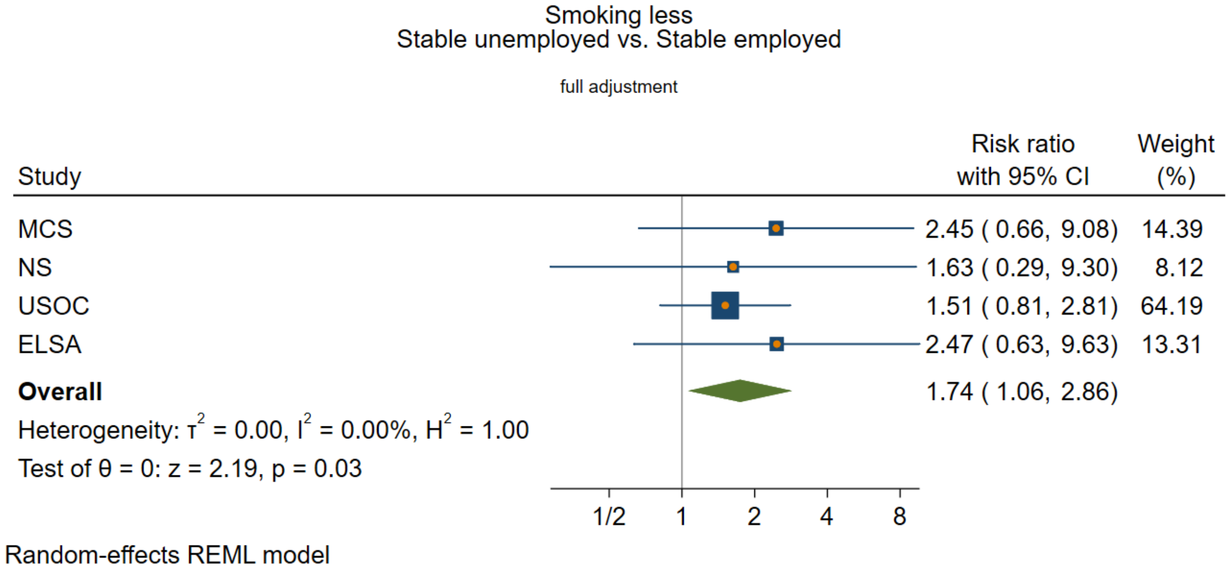
Smoking less
Stable unemployed vs. Stable employed
full adjustment
Study
Risk ratio with 95% CI
Weight (%)
MCS 2.45 (0.66, 9.08) 14.39
NS 1.63 (0.29, 9.30) 8.12
USOC 1.51 (0.81, 2.81) 64.19
ELSA 2.47 (0.63, 9.63) 13.31
Overall 1.74 (1.06, 2.86)
Heterogeneity: τ² = 0.00, I² = 0.00%, H² = 1.00
Test of θ = 0: z = 2.19, p = 0.03
1/2 1 2 4 8
Random-effects REML model

Figure set 13. Current vaper

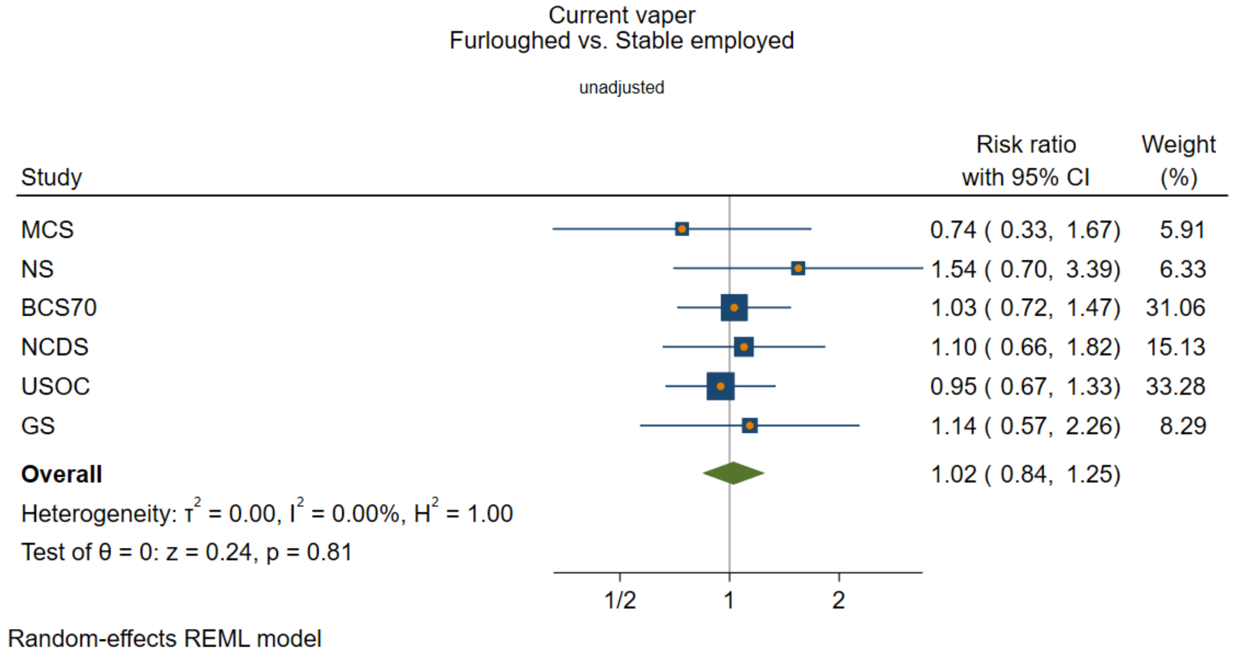

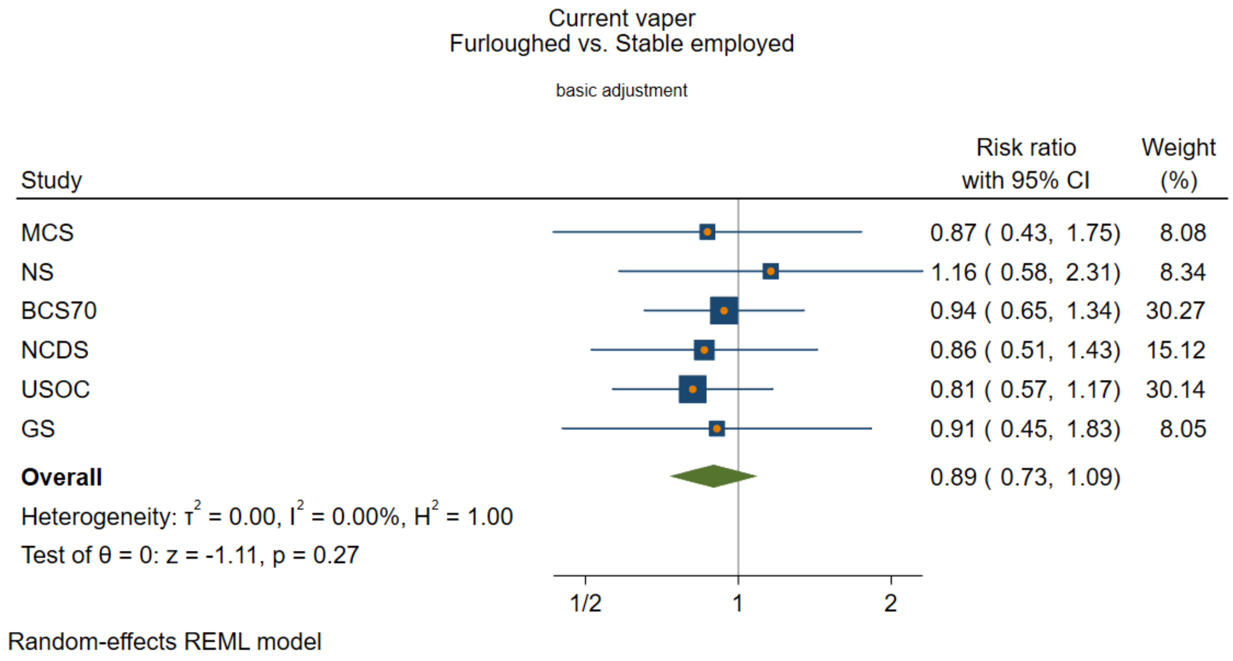

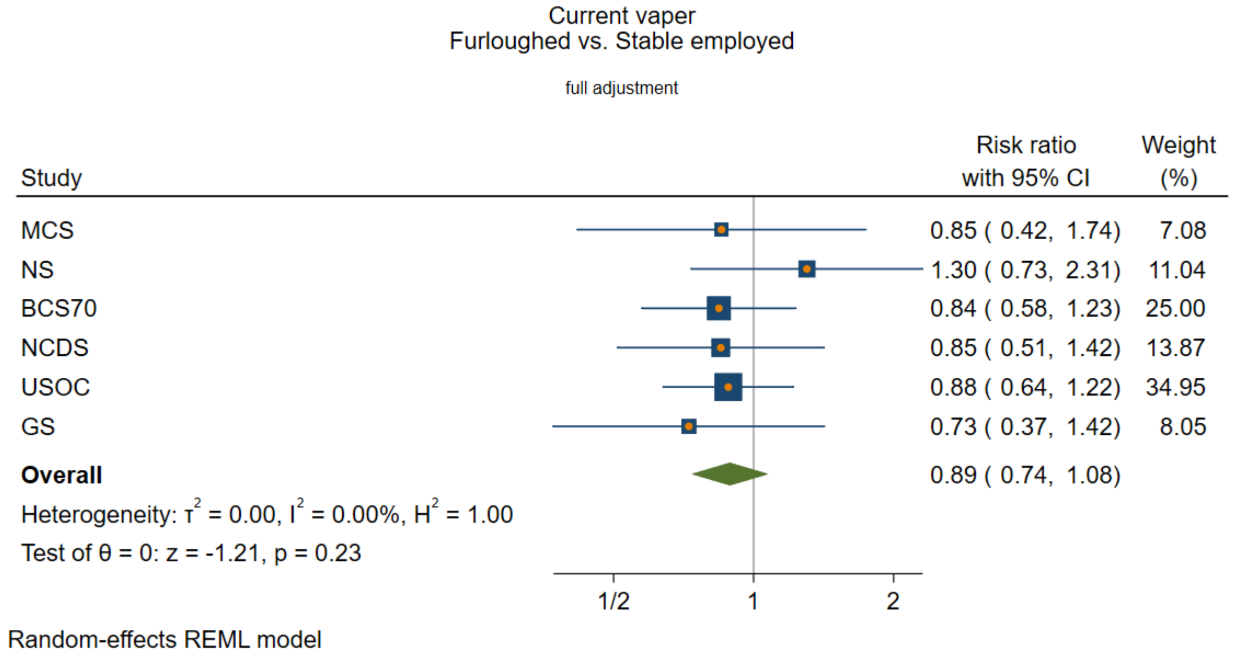

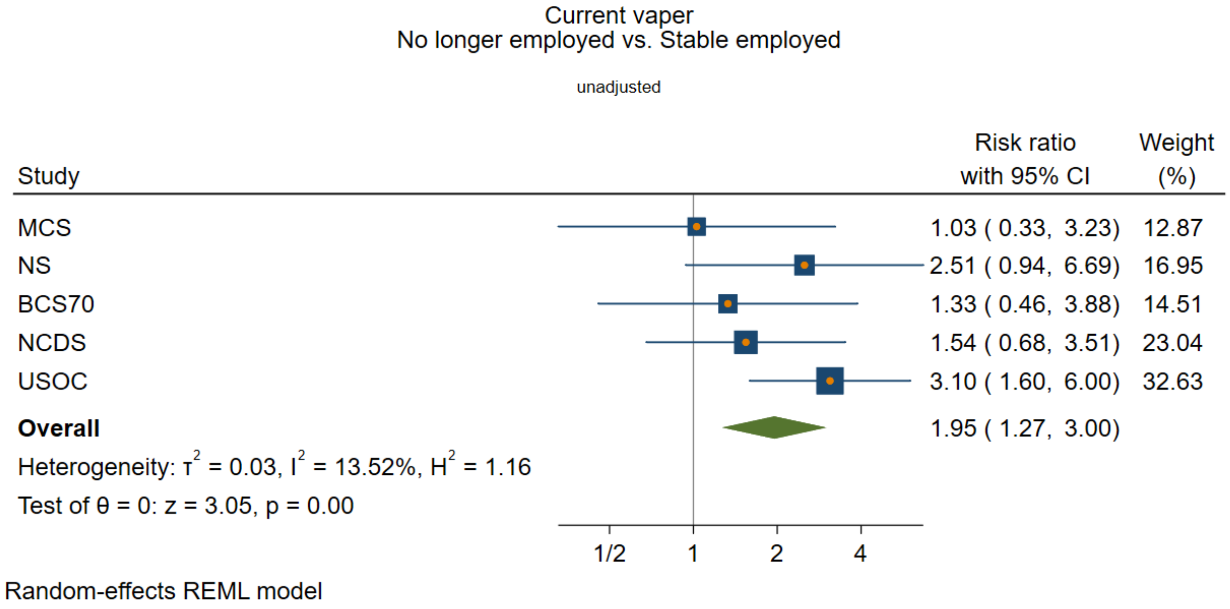
Current vaper
No longer employed vs. Stable employed
unadjusted
Study
Risk ratio with 95% CI
Weight (%)
MCS 1.03 (0.33, 3.23) 12.87
NS 2.51 (0.94, 6.69) 16.95
BCS70 1.33 (0.46, 3.88) 14.51
NCDS 1.54 (0.68, 3.51) 23.04
USOC 3.10 (1.60, 6.00) 32.63
Overall 1.95 (1.27, 3.00)
Heterogeneity: τ^2 = 0.03, I^2 = 13.52%, H^2 = 1.16
Test of θ = 0: z = 3.05, p = 0.00
1/2
1
2
4
Random-effects REML model

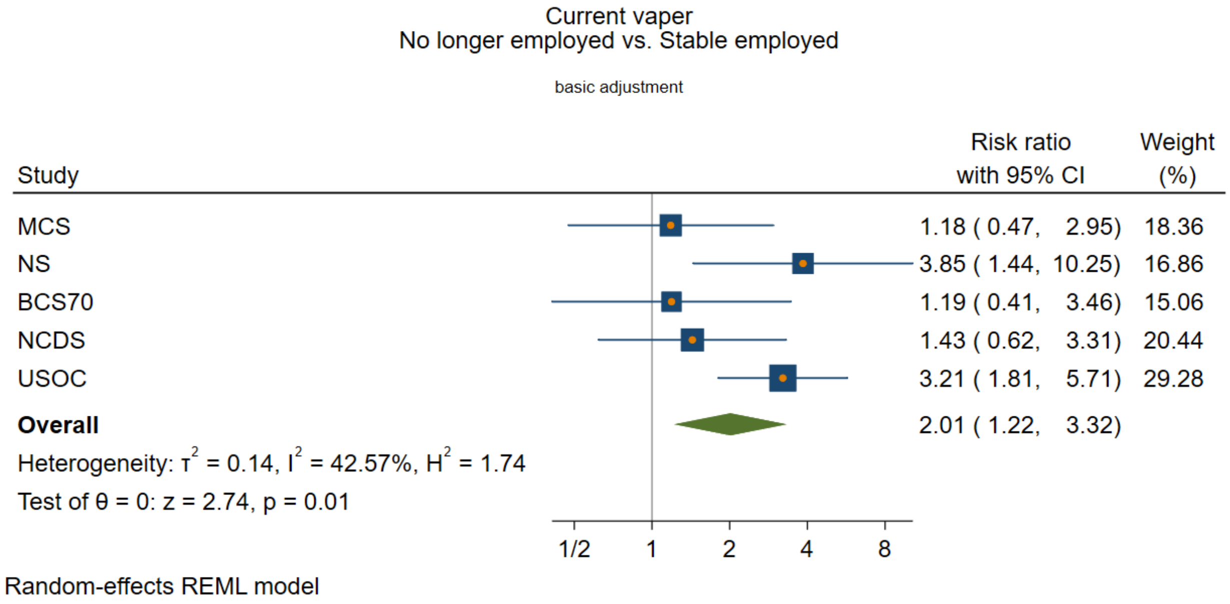
Current vaper
No longer employed vs. Stable employed
basic adjustment
Study
Risk ratio with 95% CI
Weight (%)
MCS 1.18 (0.47, 2.95) 18.36
NS 3.85 (1.44, 10.25) 16.86
BCS70 1.19 (0.41, 3.46) 15.06
NCDS 1.43 (0.62, 3.31) 20.44
USOC 3.21 (1.81, 5.71) 29.28
Overall 2.01 (1.22, 3.32)
Heterogeneity: τ^2 = 0.14, I^2 = 42.57%, H^2 = 1.74
Test of θ = 0: z = 2.74, p = 0.01
1/2
1
2
4
8
Random-effects REML model

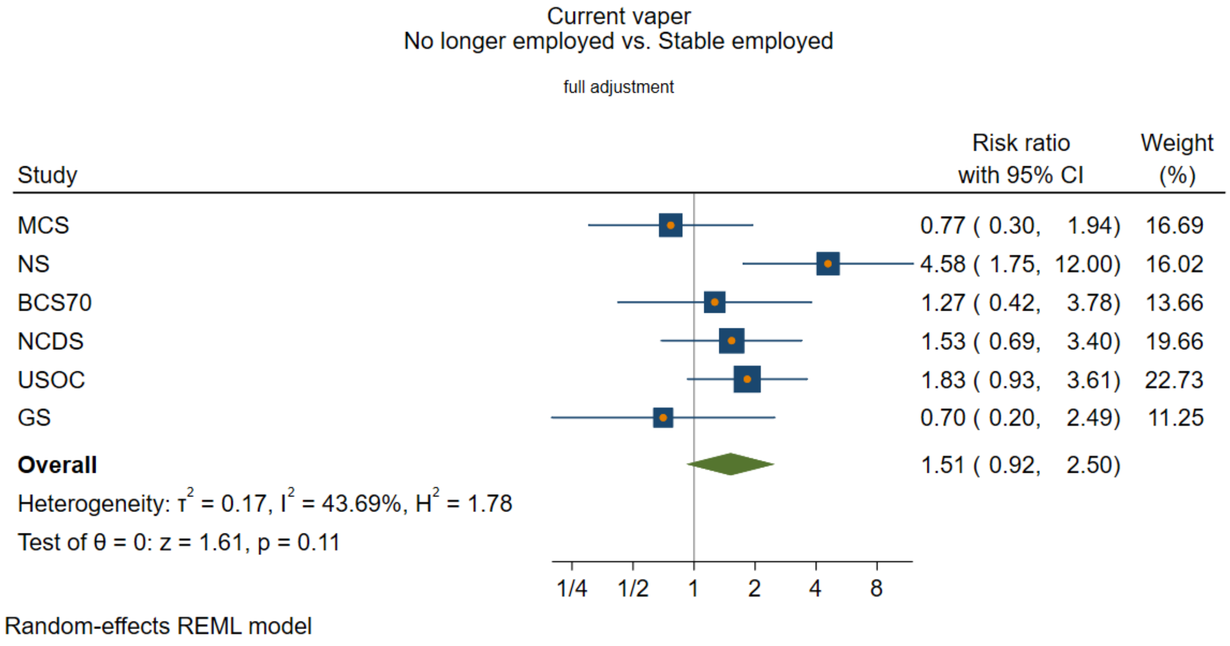
Current vaper
No longer employed vs. Stable employed
full adjustment
Study
Risk ratio with 95% CI
Weight (%)
MCS 0.77 (0.30, 1.94) 16.69
NS 4.58 (1.75, 12.00) 16.02
BCS70 1.27 (0.42, 3.78) 13.66
NCDS 1.53 (0.69, 3.40) 19.66
USOC 1.83 (0.93, 3.61) 22.73
GS 0.70 (0.20, 2.49) 11.25
Overall 1.51 (0.92, 2.50)
Heterogeneity: τ^2 = 0.17, I^2 = 43.69%, H^2 = 1.78
Test of θ = 0: z = 1.61, p = 0.11
1/4
1/2
1
2
4
8
Random-effects REML model

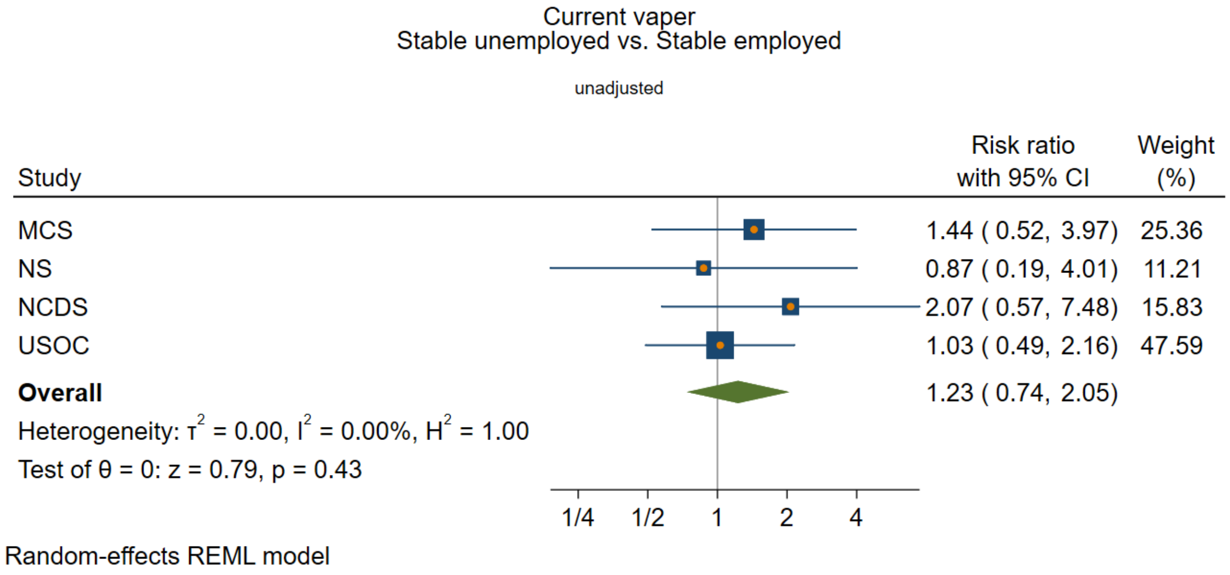
Current vaper
Stable unemployed vs. Stable employed
unadjusted
Study
Risk ratio with 95% CI
Weight (%)
MCS 1.44 (0.52, 3.97) 25.36
NS 0.87 (0.19, 4.01) 11.21
NCDS 2.07 (0.57, 7.48) 15.83
USOC 1.03 (0.49, 2.16) 47.59
Overall 1.23 (0.74, 2.05)
Heterogeneity: τ² = 0.00, I² = 0.00%, H² = 1.00
Test of θ = 0: z = 0.79, p = 0.43
1/4 1/2 1 2 4
Random-effects REML model

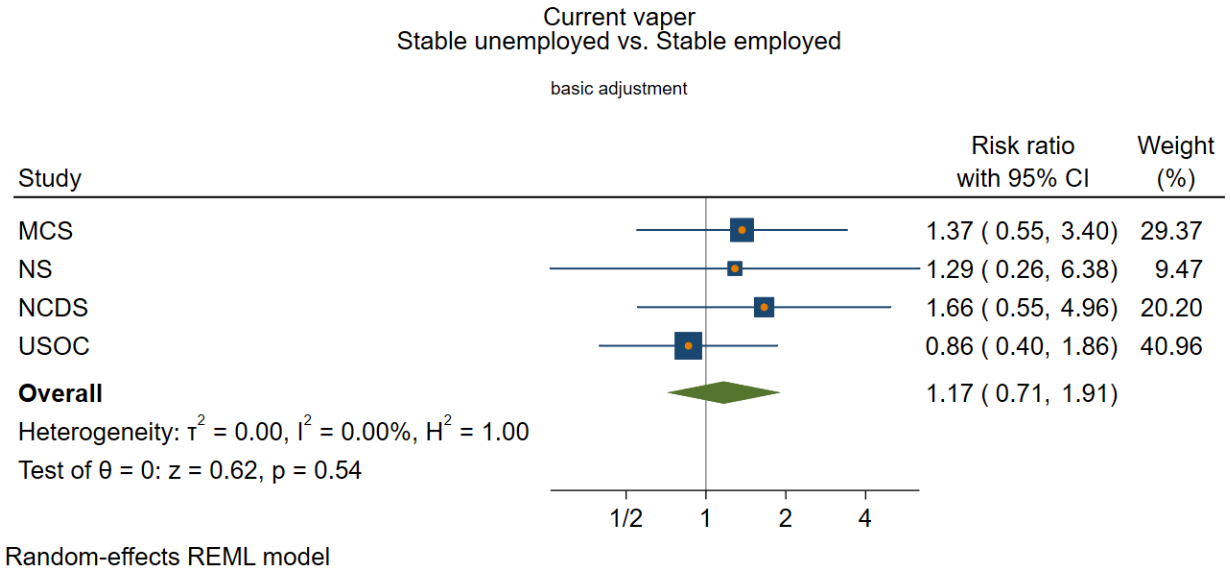
Current vaper
Stable unemployed vs. Stable employed
basic adjustment
Study
Risk ratio with 95% CI
Weight (%)
MCS 1.37 (0.55, 3.40) 29.37
NS 1.29 (0.26, 6.38) 9.47
NCDS 1.66 (0.55, 4.96) 20.20
USOC 0.86 (0.40, 1.86) 40.96
Overall 1.17 (0.71, 1.91)
Heterogeneity: τ² = 0.00, I² = 0.00%, H² = 1.00
Test of θ = 0: z = 0.62, p = 0.54
1/2 1 2 4
Random-effects REML model

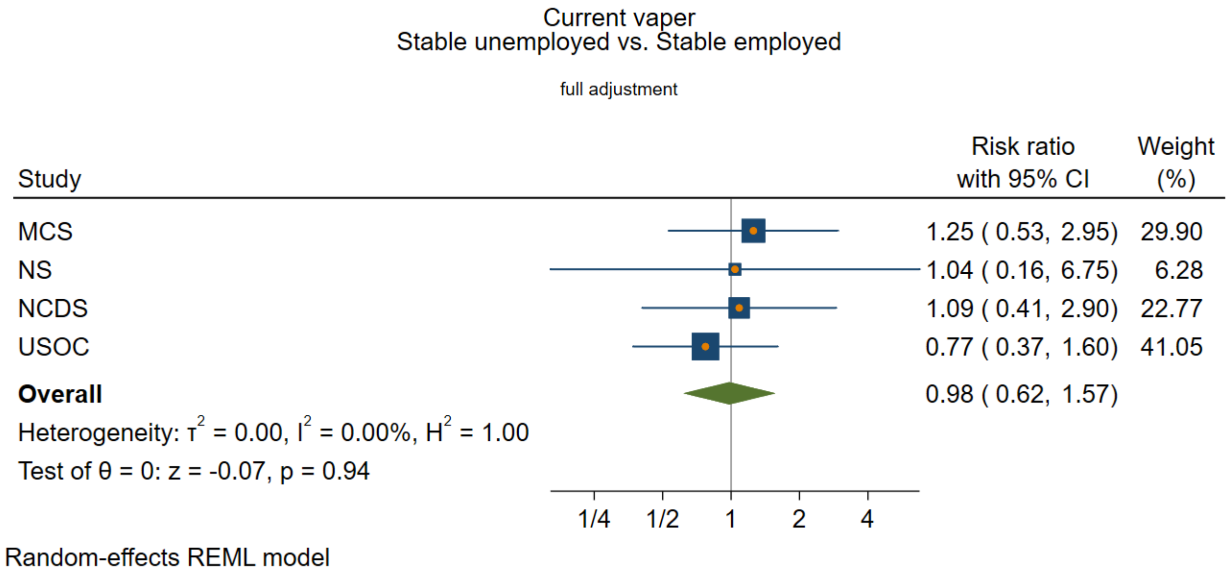
Current vaper
Stable unemployed vs. Stable employed
full adjustment
Study
Risk ratio with 95% CI
Weight (%)
MCS 1.25 (0.53, 2.95) 29.90
NS 1.04 (0.16, 6.75) 6.28
NCDS 1.09 (0.41, 2.90) 22.77
USOC 0.77 (0.37, 1.60) 41.05
Overall 0.98 (0.62, 1.57)
Heterogeneity: τ² = 0.00, I² = 0.00%, H² = 1.00
Test of θ = 0: z = -0.07, p = 0.94
1/4 1/2 1 2 4
Random-effects REML model

Figure set 14. Vaping more

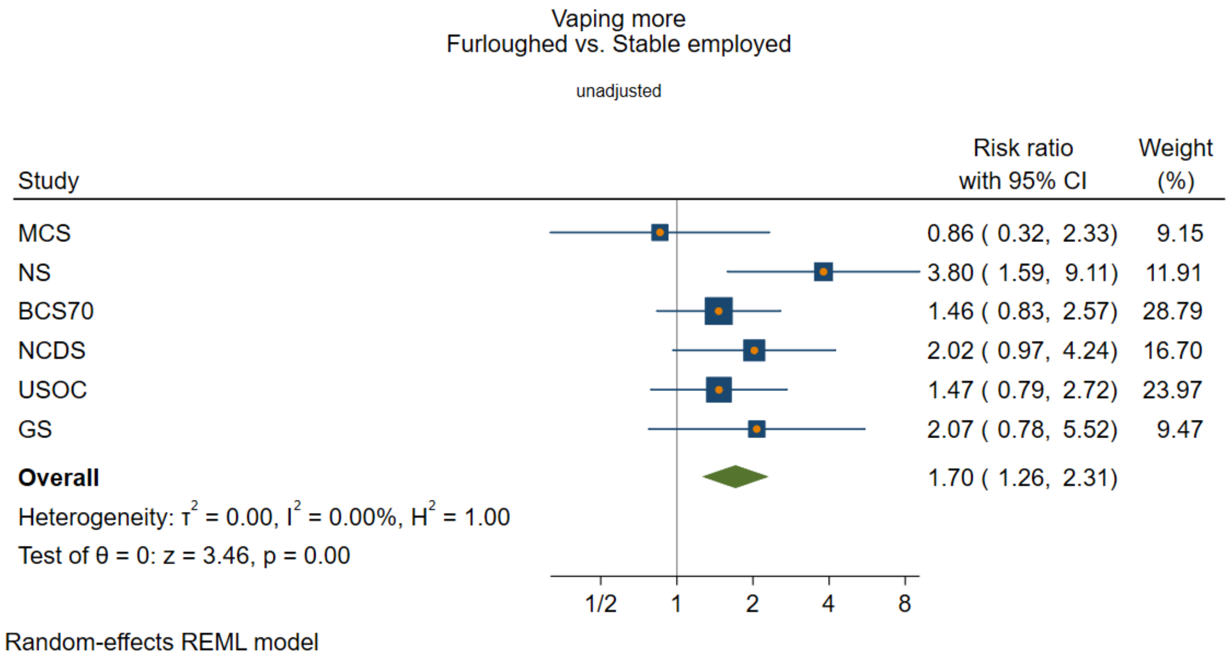

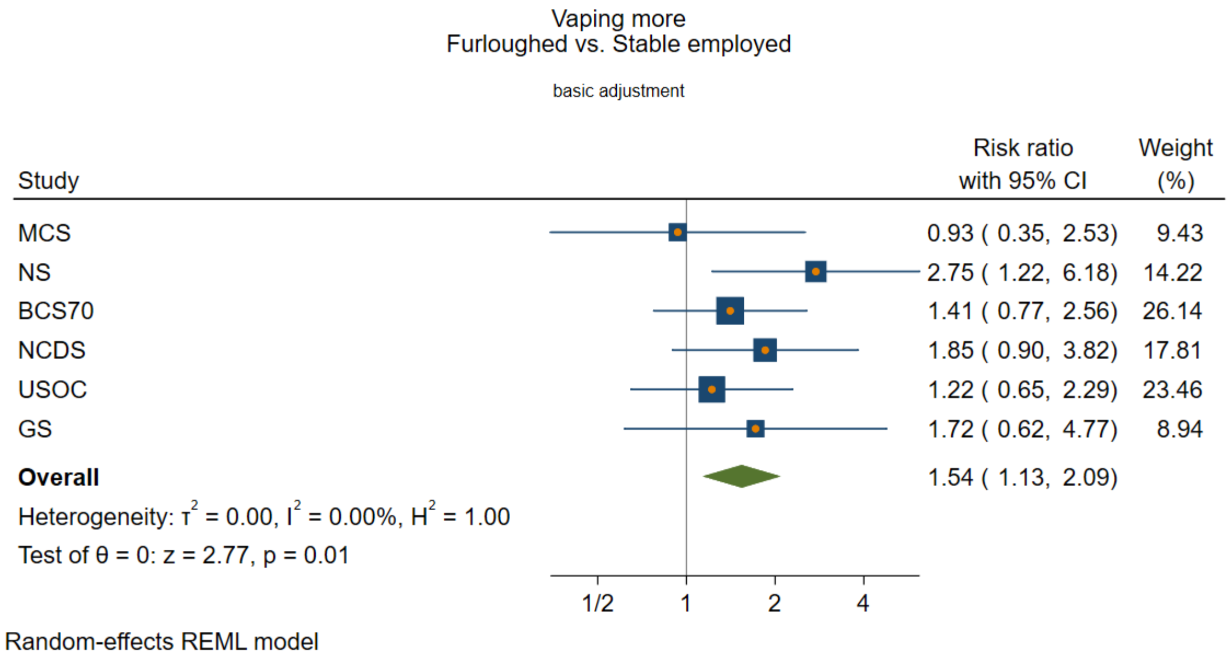

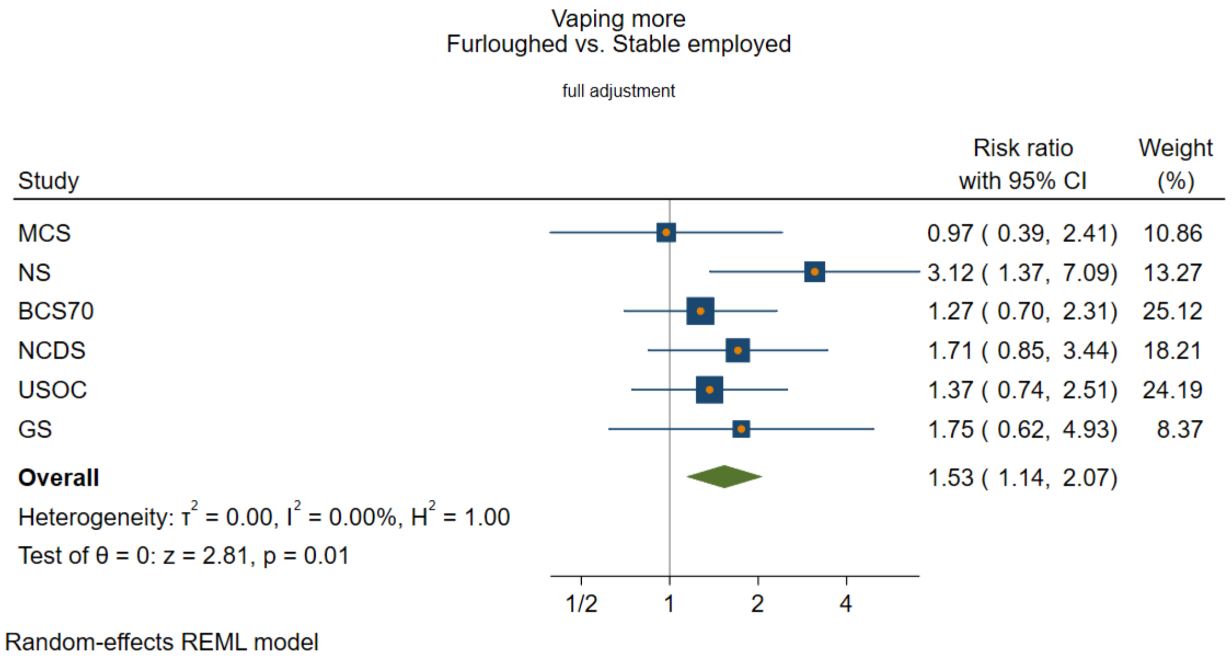

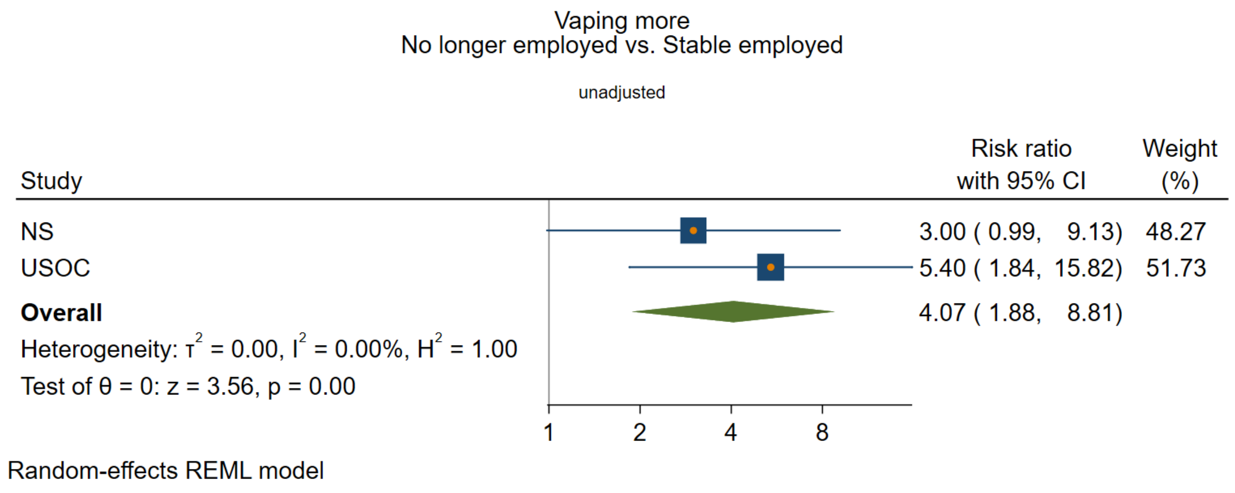
Vaping more
No longer employed vs. Stable employed
unadjusted
Study
Risk ratio with 95% CI
Weight (%)
NS
3.00 (0.99, 9.13)
48.27
USOC
5.40 (1.84, 15.82)
51.73
Overall
4.07 (1.88, 8.81)
Heterogeneity: τ^2 = 0.00, I^2 = 0.00%, H^2 = 1.00
Test of θ = 0: z = 3.56, p = 0.00
1
2
4
8
Random-effects REML model

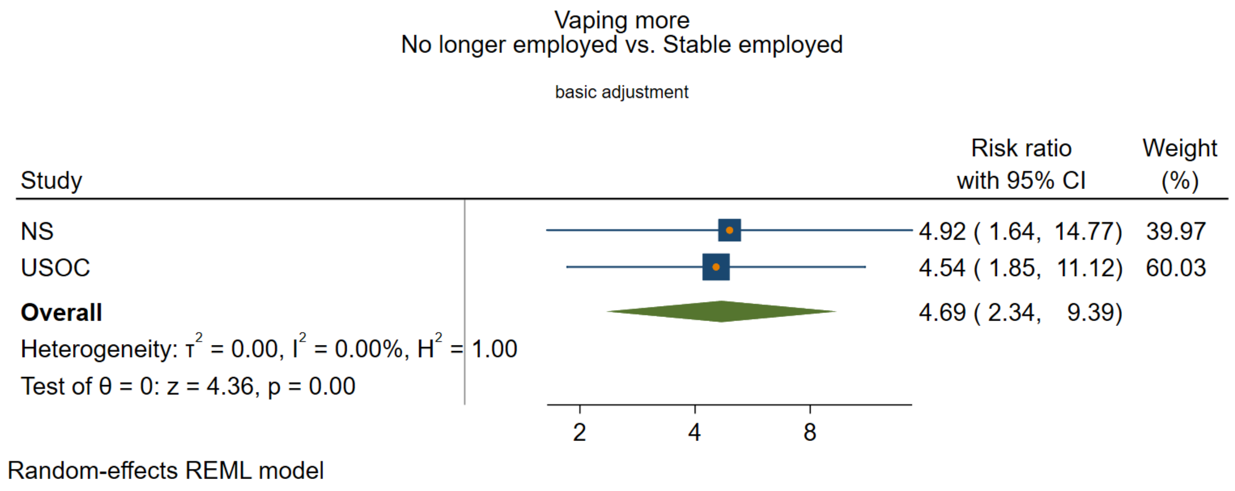
Vaping more
No longer employed vs. Stable employed
basic adjustment
Study
Risk ratio with 95% CI
Weight (%)
NS
4.92 (1.64, 14.77)
39.97
USOC
4.54 (1.85, 11.12)
60.03
Overall
4.69 (2.34, 9.39)
Heterogeneity: τ^2 = 0.00, I^2 = 0.00%, H^2 = 1.00
Test of θ = 0: z = 4.36, p = 0.00
2
4
8
Random-effects REML model

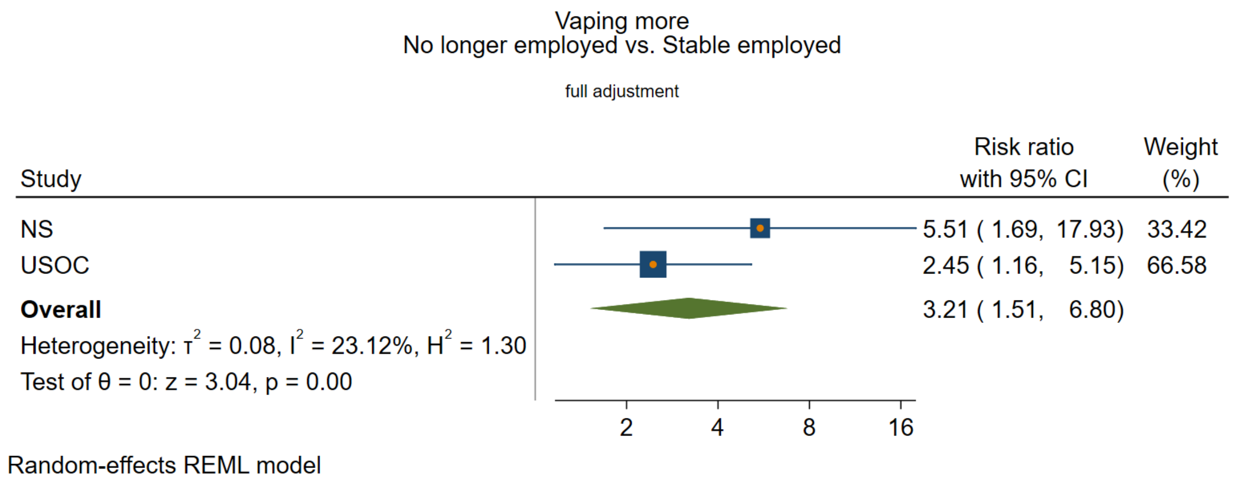
Vaping more
No longer employed vs. Stable employed
full adjustment
Study
Risk ratio with 95% CI
Weight (%)
NS
5.51 (1.69, 17.93)
33.42
USOC
2.45 (1.16, 5.15)
66.58
Overall
3.21 (1.51, 6.80)
Heterogeneity: τ^2 = 0.08, I^2 = 23.12%, H^2 = 1.30
Test of θ = 0: z = 3.04, p = 0.00
2
4
8
16
Random-effects REML model

Vaping more
Stable unemployed vs. Stable employed

unadjusted

Study	Risk ratio with 95% CI	Weight (%)
MCS	0.63 (0.15, 2.60)	23.51
NS	1.81 (0.37, 8.72)	21.34
NCDS	8.32 (2.37, 29.20)	25.99
USOC	2.05 (0.71, 5.93)	29.15
**Overall**	2.17 (0.77, 6.13)	

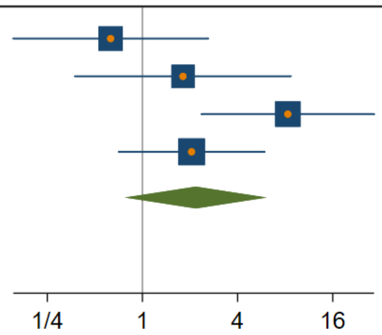

Heterogeneity: $\tau^2 = 0.66$, $I^2 = 59.94\%$, $H^2 = 2.50$

Test of $\theta = 0$: z = 1.47, p = 0.14

Random-effects REML model

Vaping more
Stable unemployed vs. Stable employed

basic adjustment

Study	Risk ratio with 95% CI	Weight (%)
MCS	0.68 (0.14, 3.27)	21.38
NS	1.75 (0.27, 11.51)	17.41
NCDS	6.58 (2.66, 16.26)	32.22
USOC	1.61 (0.54, 4.77)	28.99
**Overall**	2.14 (0.77, 5.92)	

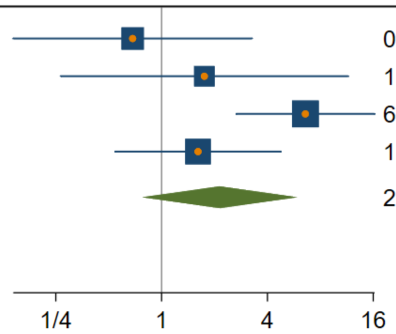

Heterogeneity: $\tau^2 = 0.62$, $I^2 = 59.84\%$, $H^2 = 2.49$

Test of $\theta = 0$: z = 1.47, p = 0.14

Random-effects REML model

Vaping more
Stable unemployed vs. Stable employed

full adjustment

Study	Risk ratio with 95% CI	Weight (%)
MCS	0.76 (0.14, 4.27)	16.50
NS	1.95 (0.29, 13.29)	13.66
NCDS	4.07 (1.43, 11.62)	36.10
USOC	1.44 (0.48, 4.31)	33.74
**Overall**	1.97 (0.93, 4.17)	

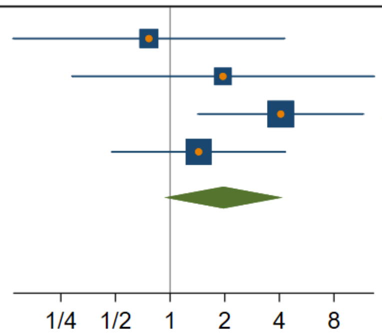

Heterogeneity: $\tau^2 = 0.12$, $I^2 = 20.23\%$, $H^2 = 1.25$

Test of $\theta = 0$: z = 1.76, p = 0.08

Random-effects REML model

Figure set 15. Vaping less

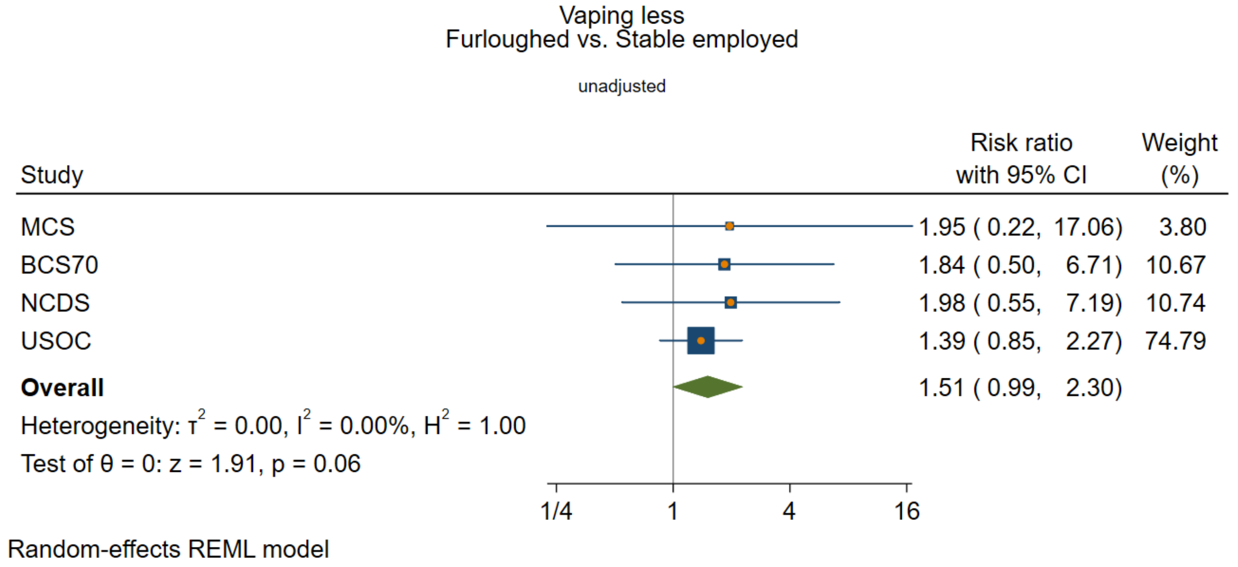

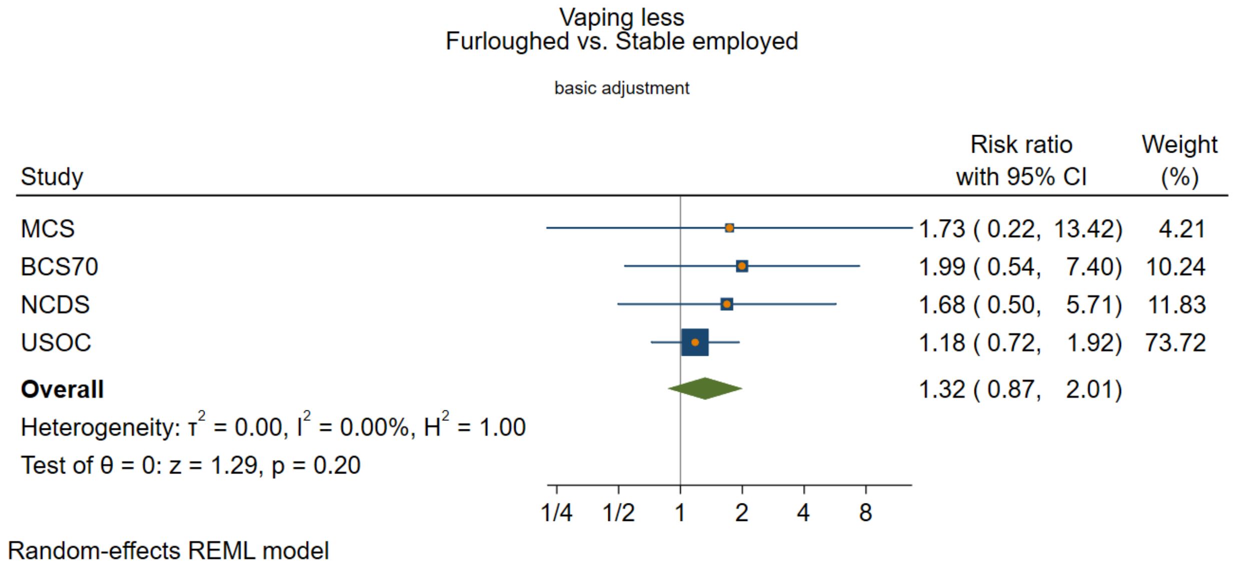

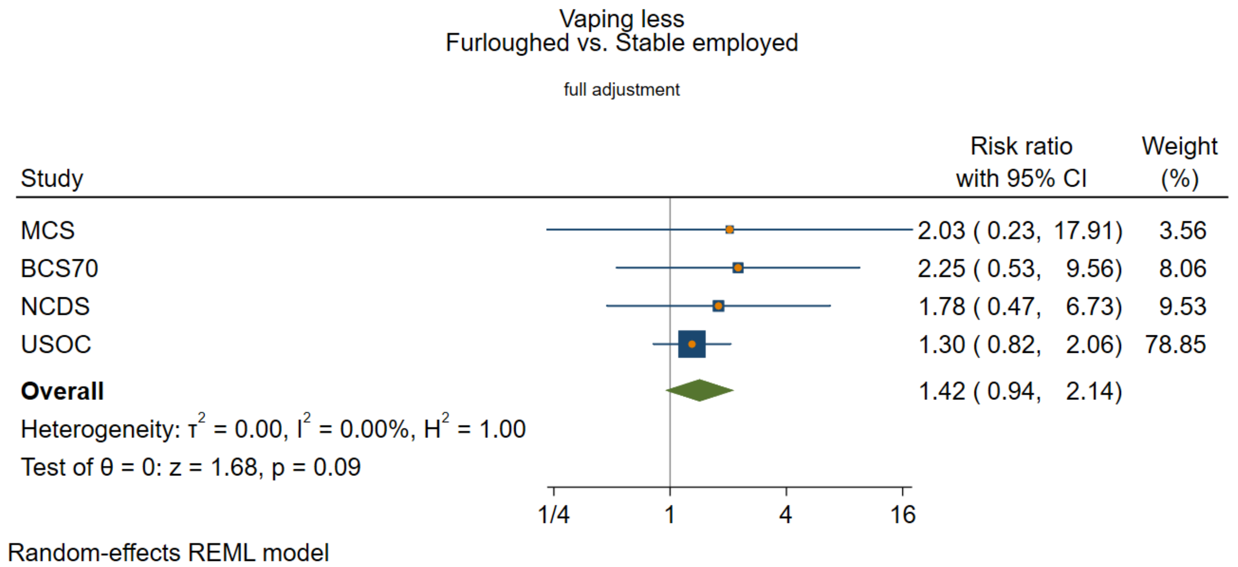

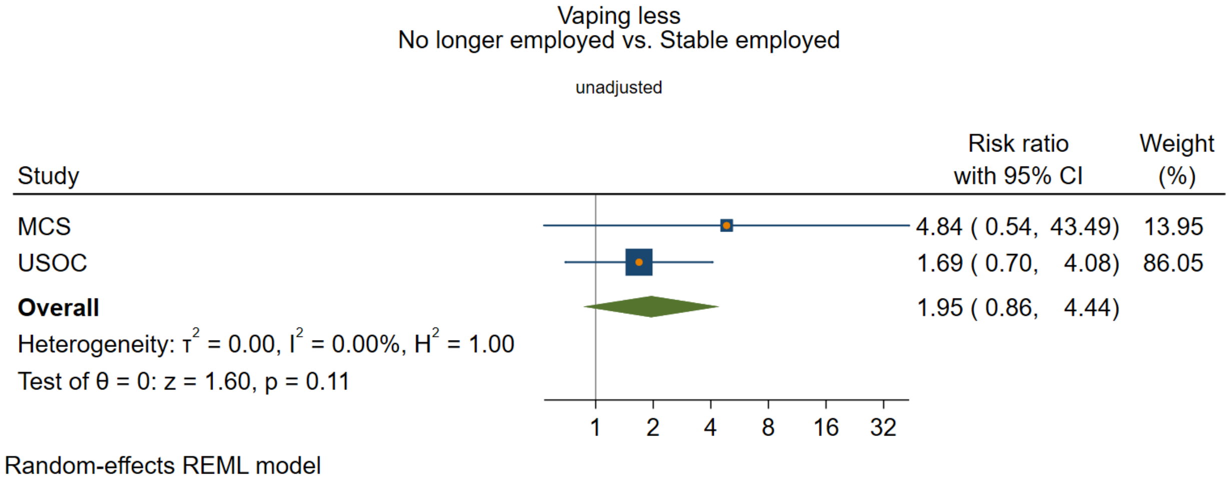
Vaping less
No longer employed vs. Stable employed
unadjusted
Study
Risk ratio with 95% CI
Weight (%)
MCS
4.84 (0.54, 43.49)
13.95
USOC
1.69 (0.70, 4.08)
86.05
Overall
1.95 (0.86, 4.44)
Heterogeneity: τ² = 0.00, I² = 0.00%, H² = 1.00
Test of θ = 0: z = 1.60, p = 0.11
1
2
4
8
16
32
Random-effects REML model

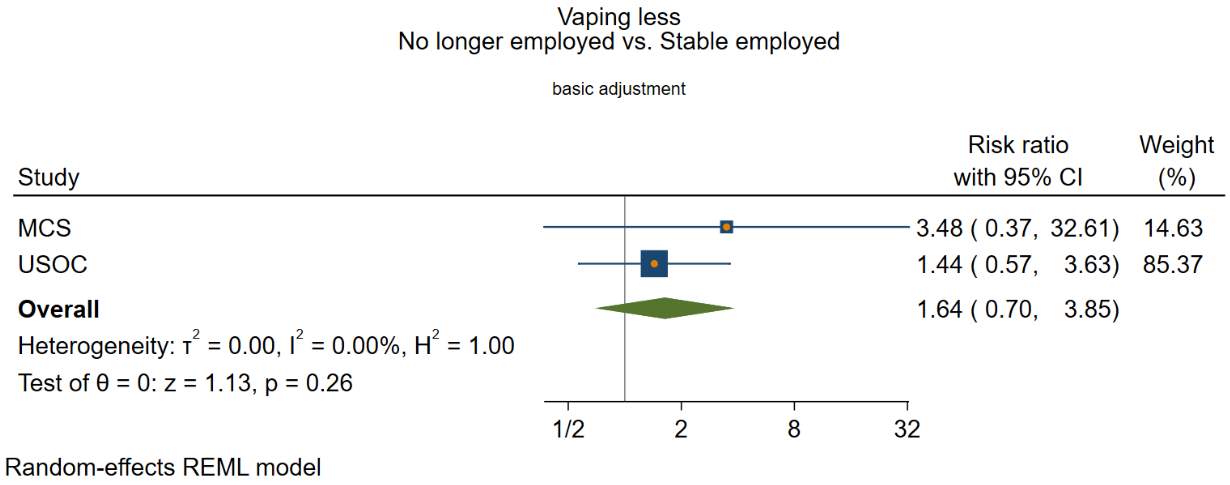
Vaping less
No longer employed vs. Stable employed
basic adjustment
Study
Risk ratio with 95% CI
Weight (%)
MCS
3.48 (0.37, 32.61)
14.63
USOC
1.44 (0.57, 3.63)
85.37
Overall
1.64 (0.70, 3.85)
Heterogeneity: τ² = 0.00, I² = 0.00%, H² = 1.00
Test of θ = 0: z = 1.13, p = 0.26
1/2
2
8
32
Random-effects REML model

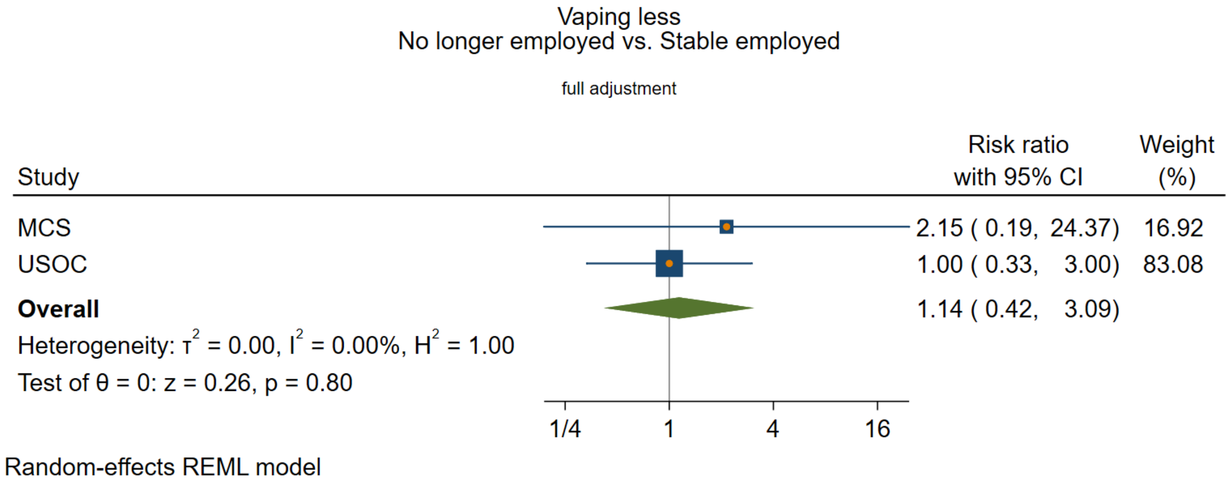
Vaping less
No longer employed vs. Stable employed
full adjustment
Study
Risk ratio with 95% CI
Weight (%)
MCS
2.15 (0.19, 24.37)
16.92
USOC
1.00 (0.33, 3.00)
83.08
Overall
1.14 (0.42, 3.09)
Heterogeneity: τ² = 0.00, I² = 0.00%, H² = 1.00
Test of θ = 0: z = 0.26, p = 0.80
1/4
1
4
16
Random-effects REML model

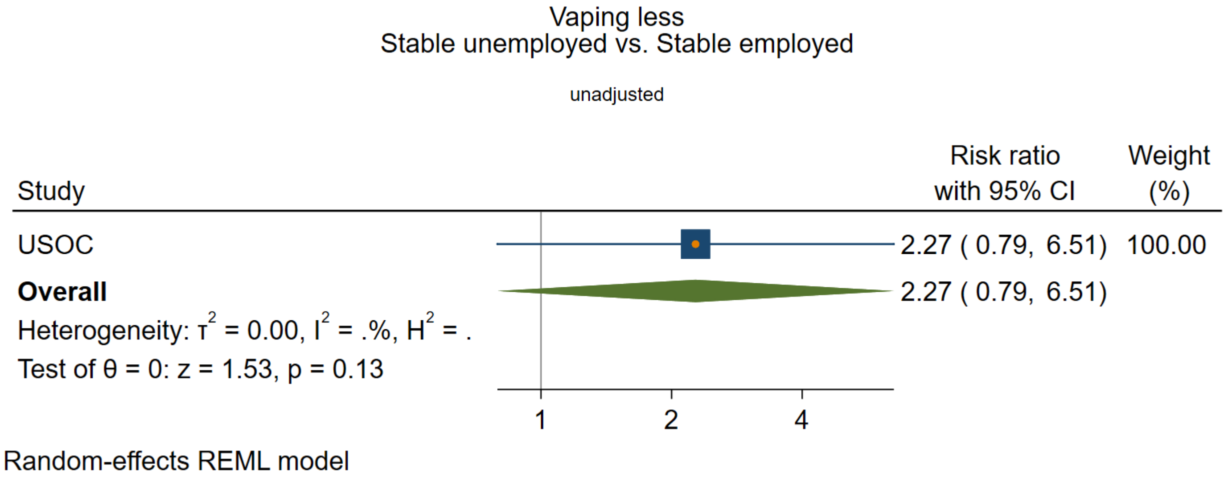
Vaping less
Stable unemployed vs. Stable employed
unadjusted
Study
Risk ratio with 95% CI
Weight (%)
USOC
2.27 (0.79, 6.51)
100.00
Overall
2.27 (0.79, 6.51)
Heterogeneity: τ2 = 0.00, I2 = .%, H2 = .
Test of θ = 0: z = 1.53, p = 0.13
1
2
4
Random-effects REML model

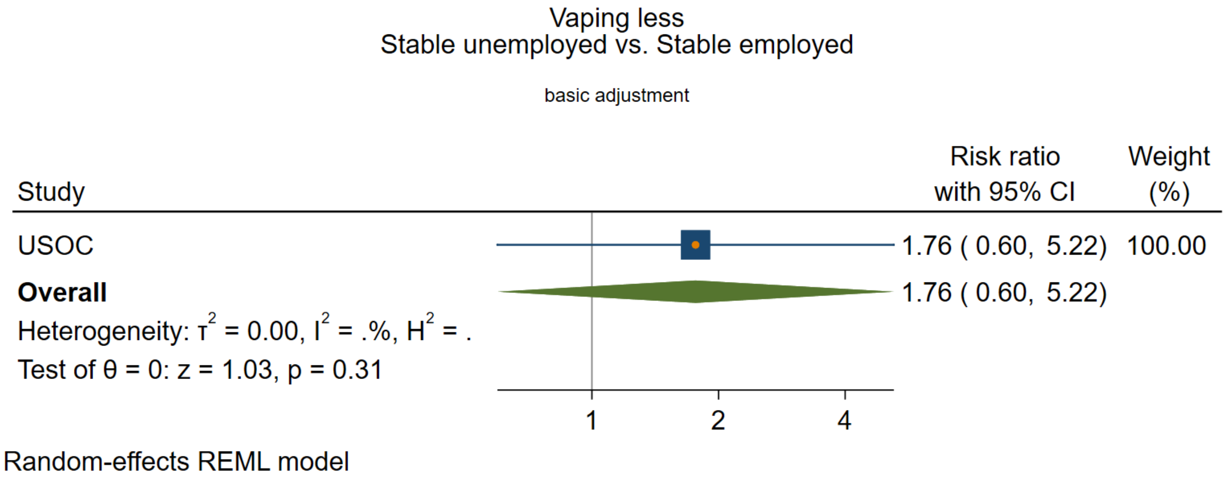
Vaping less
Stable unemployed vs. Stable employed
basic adjustment
Study
Risk ratio with 95% CI
Weight (%)
USOC
1.76 (0.60, 5.22)
100.00
Overall
1.76 (0.60, 5.22)
Heterogeneity: τ2 = 0.00, I2 = .%, H2 = .
Test of θ = 0: z = 1.03, p = 0.31
1
2
4
Random-effects REML model

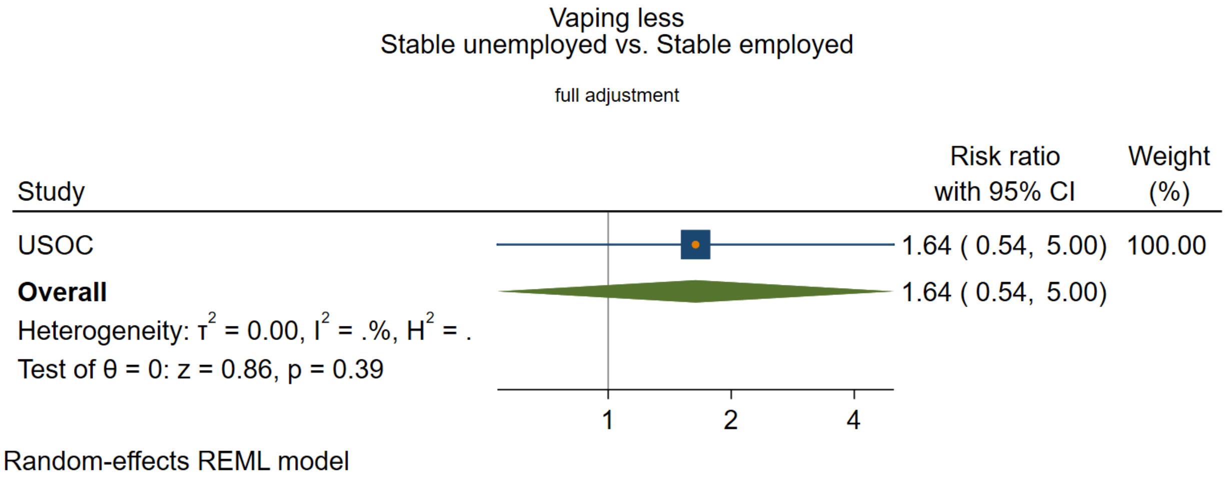
Vaping less
Stable unemployed vs. Stable employed
full adjustment
Study
Risk ratio with 95% CI
Weight (%)
USOC
1.64 (0.54, 5.00)
100.00
Overall
1.64 (0.54, 5.00)
Heterogeneity: τ2 = 0.00, I2 = .%, H2 = .
Test of θ = 0: z = 0.86, p = 0.39
1
2
4
Random-effects REML model
