# Supplementary material for: The UK Coronavirus Job Retention Scheme and smoking, alcohol consumption and vaping during the COVID-19 pandemic: evidence from eight longitudinal population surveys
Source: BMC Med. 2022 Sep 21;20:345. doi: 10.1186/s12916-022-02511-0 (PMC9489267; doi:10.1186/s12916-022-02511-0)
Supplement: Supplementary file 4 — Additional file 4. Stratified Analysis (Forest plots). Figure set 1 16 31. Currently drinks 4+ days/week or 5+ drinks/occasion. Figure set 2 17 32. Increased alcohol consumption. Figure set 3 18 33. Reduced alcohol consumption. Figure set 4 19 34. Drinks 5+ drinks/occasion. Figure set 5 20 35. Drinks more alcohol units per occasion. Figure set 6 21 36. Drinks fewer alcohol units per occasion. Figure set 7 22 37. Currently drinks 4+ days/week. Figure set 8 23 38. Drinks more frequently. Figure set 9 24 39: Drinks less frequently. Figure set 10 25 40. Current smoker. Figure set 11 26 41. Smoking more. Figure set 12 27 42. Smoking less. Figure set 13 28 43. Current vaper. Figure set 14 29 44. Vaping more. Figure set 15 30 45. Vaping less. [file 12916_2022_2511_MOESM4_ESM.pdf]

Additional File 4: Stratified Results

Results stratified by Age

Figure set 1: Currently drinks 4+ days/week or 5+ drinks/occasion 3
Figure set 2: Increased alcohol consumption 5
Figure set 3: Reduced alcohol consumption 7
Figure set 4: Drinks 5+ drinks/occasion 9
Figure set 5: Drinks more alcohol units per occasion 10
Figure set 6: Drinks fewer alcohol units per occasion 12
Figure set 7: Currently drinks 4+ days/week 14
Figure set 8: Drinks more frequently 16
Figure set 9: Drinks less frequently 18
Figure set 10: Current smoker 20
Figure set 11: Smoking more 22
Figure set 12: Smoking less 24
Figure set 13: Current vaper 26
Figure set 14: Vaping more 28
Figure set 15: Vaping less 30

Results stratified by Education

Figure set 16: Currently drinks 4+ days/week or 5+ drinks/occasion 32
Figure set 17: Increased alcohol consumption 35
Figure set 18: Reduced alcohol consumption 37
Figure set 19: Currently drinks 5+ drinks/occasions 39
Figure set 20: Drinks more alcohol units per occasions 41
Figure set 21: Drinks fewer alcohol units per occasions 43
Figure set 22: Currently drinks 4+ days/week 45
Figure set 23: Drinks more frequently 47
Figure set 24: Drinks less frequently 49
Figure set 24: Current smoker 51
Figure set 26: Smoking more 53
Figure set 27: Smoking less 54
Figure set 28: Current vaper 56
Figure set 29: Vaping more 58
Figure set 30: Vaping less 60

Results stratified by Sex

Figure set 31: Currently drinks 4+ days/week or 5+ drinks/occasion 62
Figure set 32: Increased alcohol consumption 65
Figure set 33: Reduced alcohol consumption 67
Figure set 34: Currently drinks 5+ drinks/occasion 69
Figure set 35: Drinks more alcohol units per occasion 71
Figure set 36: Drinks fewer alcohol units per occasion 73
Figure set 37: Currently drinks 4+ days/week 75
Figure set 38: Drinks more frequently 77
Figure set 39: Drinks less frequently 79
Figure set 40: Current smoker 81
Figure set 41: Smoking more 83
Figure set 42: Smoking less 85
Figure set 43: Current vaper 87
Figure set 44: Vaping more 89
Figure set 45: Vaping less 91

Notes: Moderation by age, education, and sex was assessed with stratified regressions using "full" adjustment that includes socio-demographic characteristics as well as pre-pandemic measures of psychological distress, self-rated health, and health behaviours.

Figure set 1: Currently drinks 4+ days/week or 5+ drinks/occasion

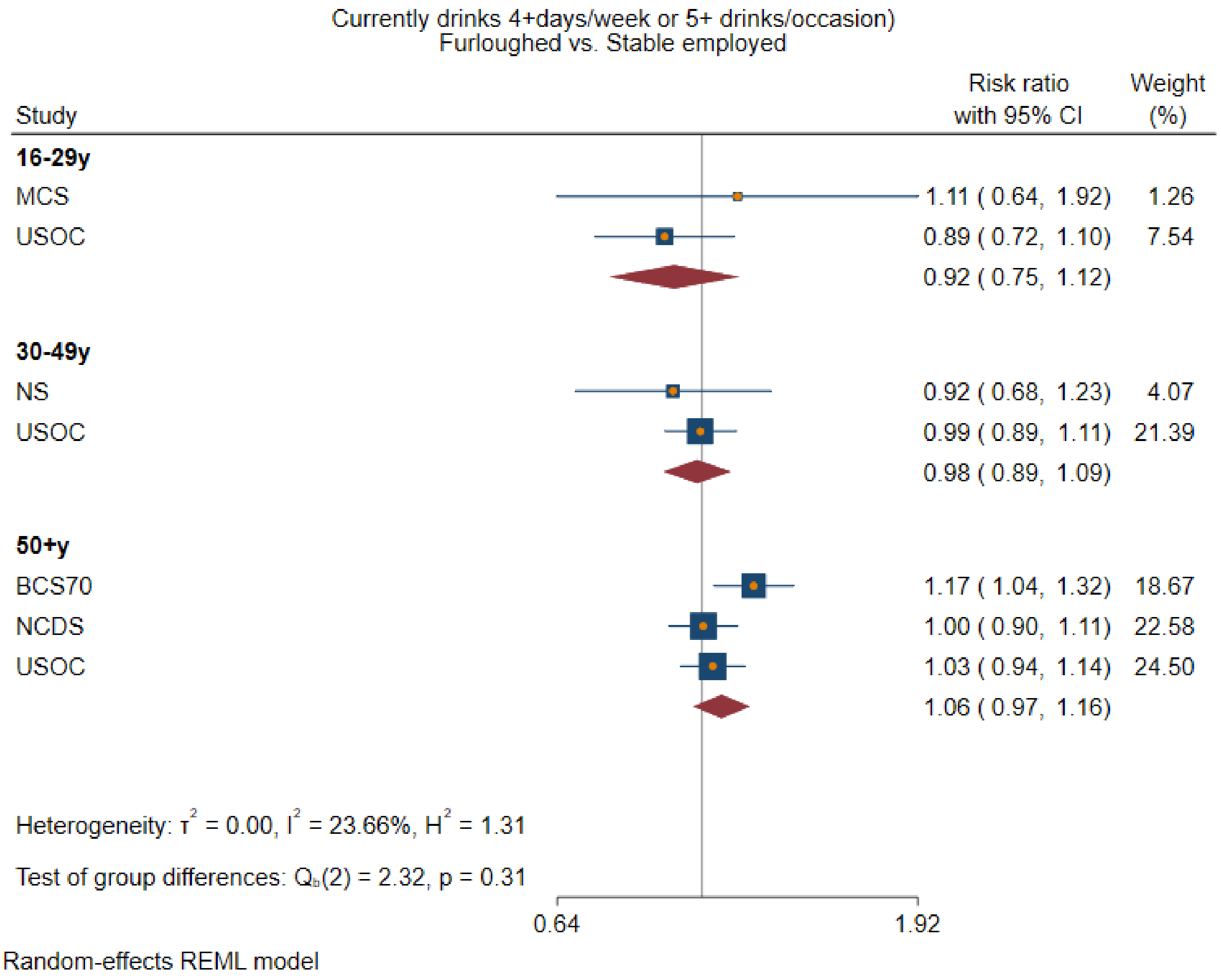

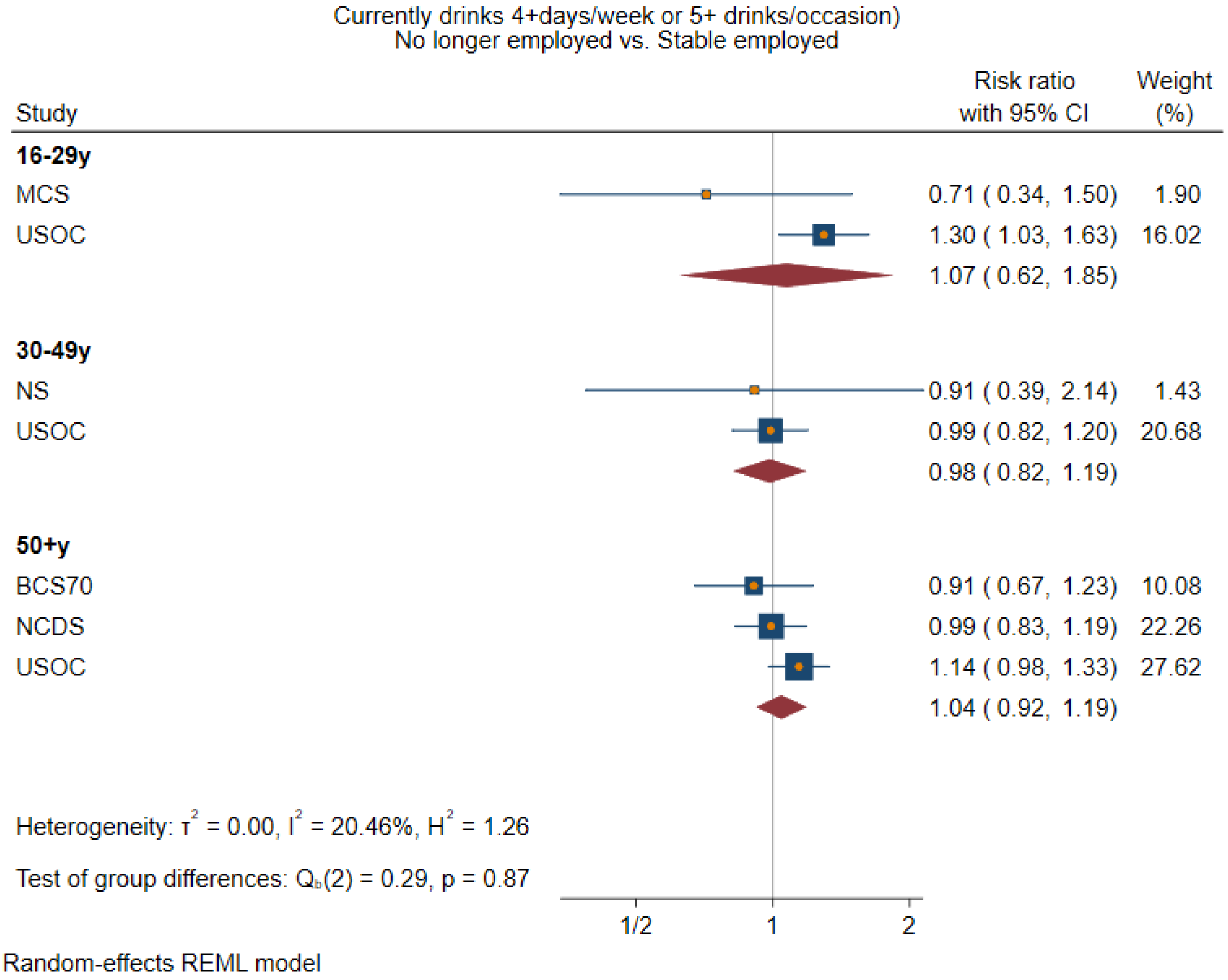

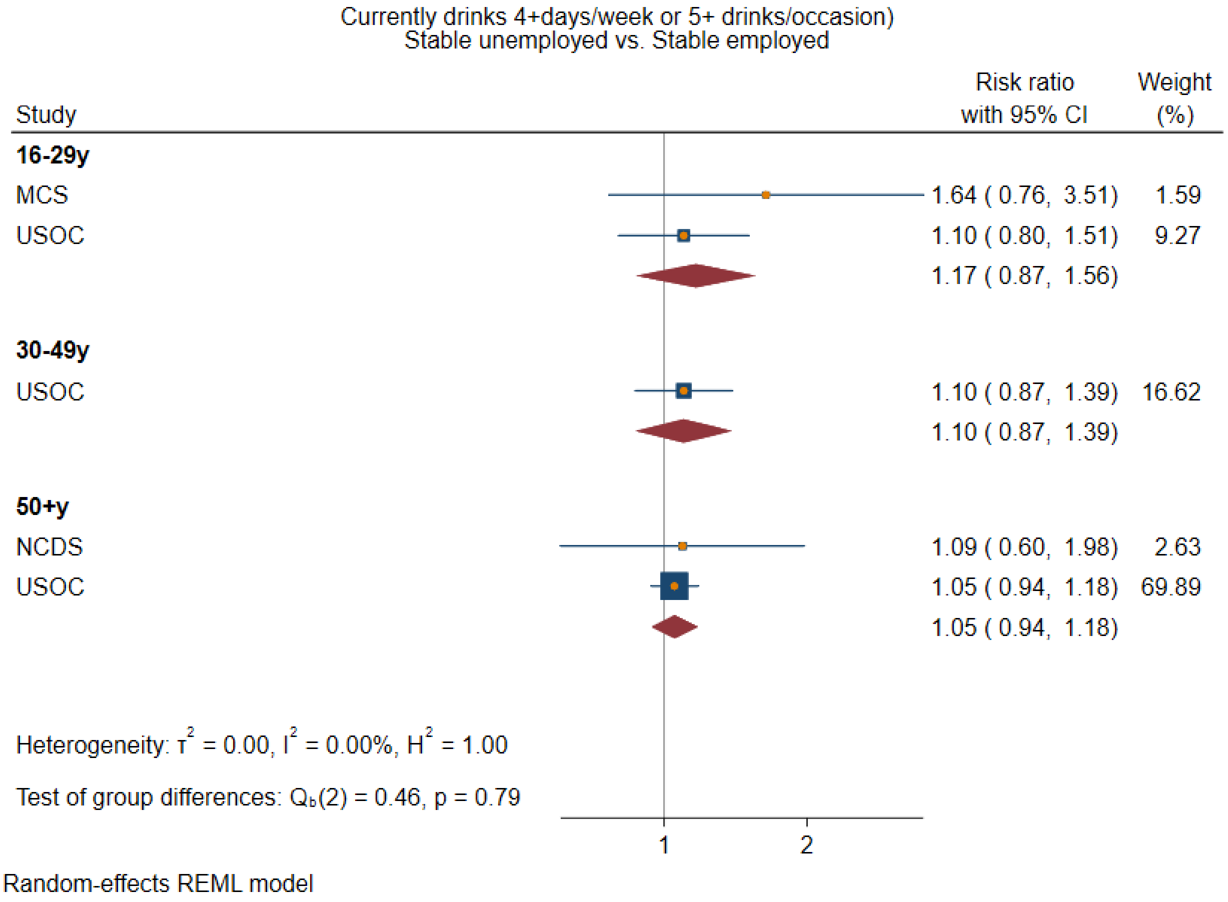
Currently drinks 4+days/week or 5+ drinks/occasion)
Stable unemployed vs. Stable employed
Study
Risk ratio with 95% CI
Weight (%)
16-29y
MCS 1.64 (0.76, 3.51) 1.59
USOC 1.10 (0.80, 1.51) 9.27
1.17 (0.87, 1.56)
30-49y
USOC 1.10 (0.87, 1.39) 16.62
1.10 (0.87, 1.39)
50+y
NCDS 1.09 (0.60, 1.98) 2.63
USOC 1.05 (0.94, 1.18) 69.89
1.05 (0.94, 1.18)
Heterogeneity: τ^2 = 0.00, I^2 = 0.00%, H^2 = 1.00
Test of group differences: $Q_b(2)$ = 0.46, p = 0.79
1
2
Random-effects REML model

Figure set 2: Increased alcohol consumption

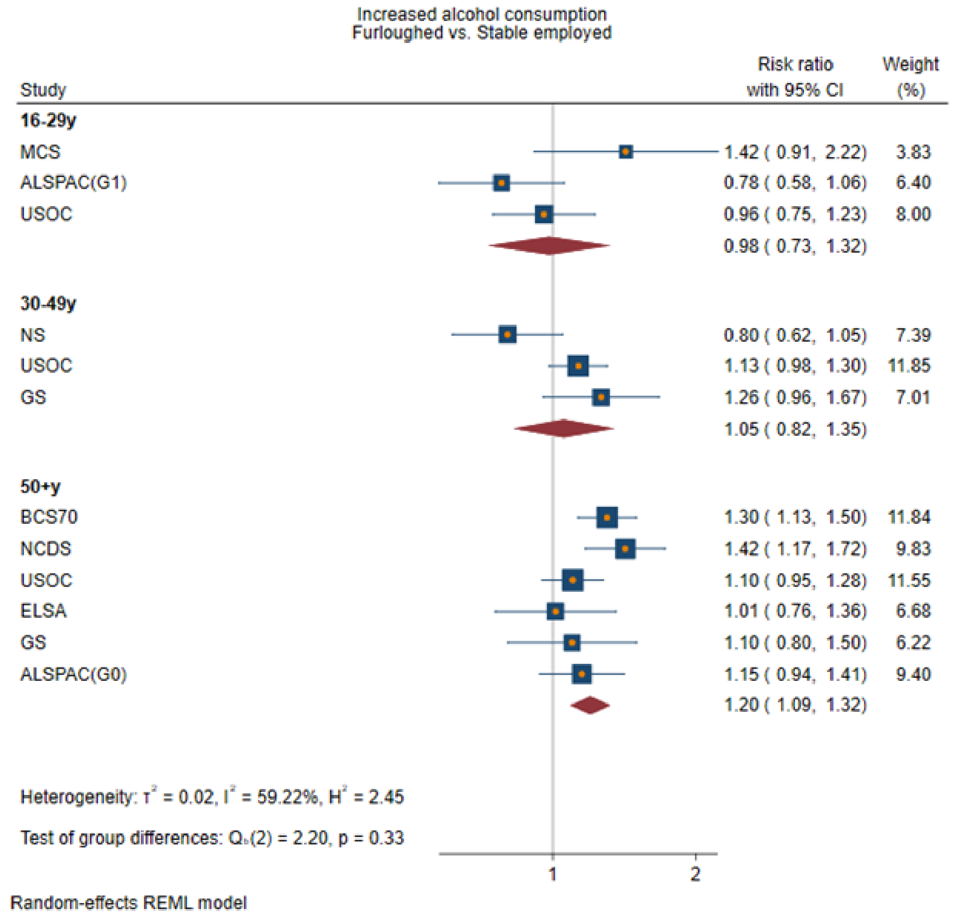

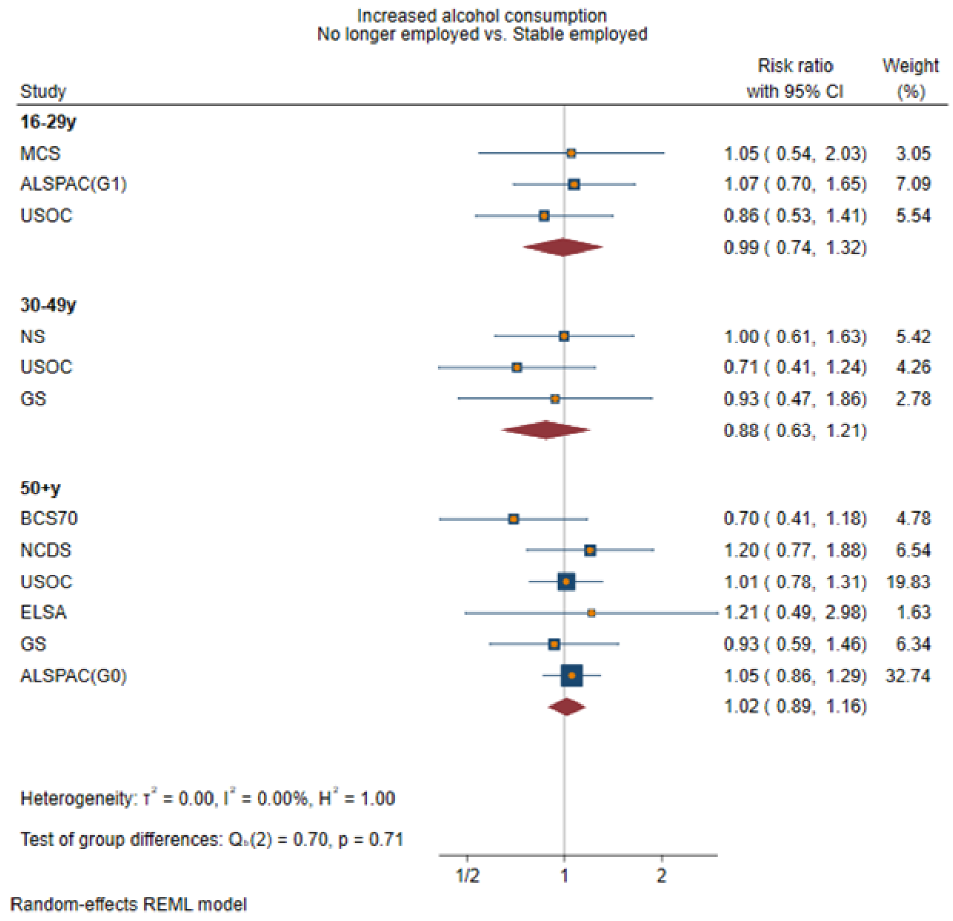

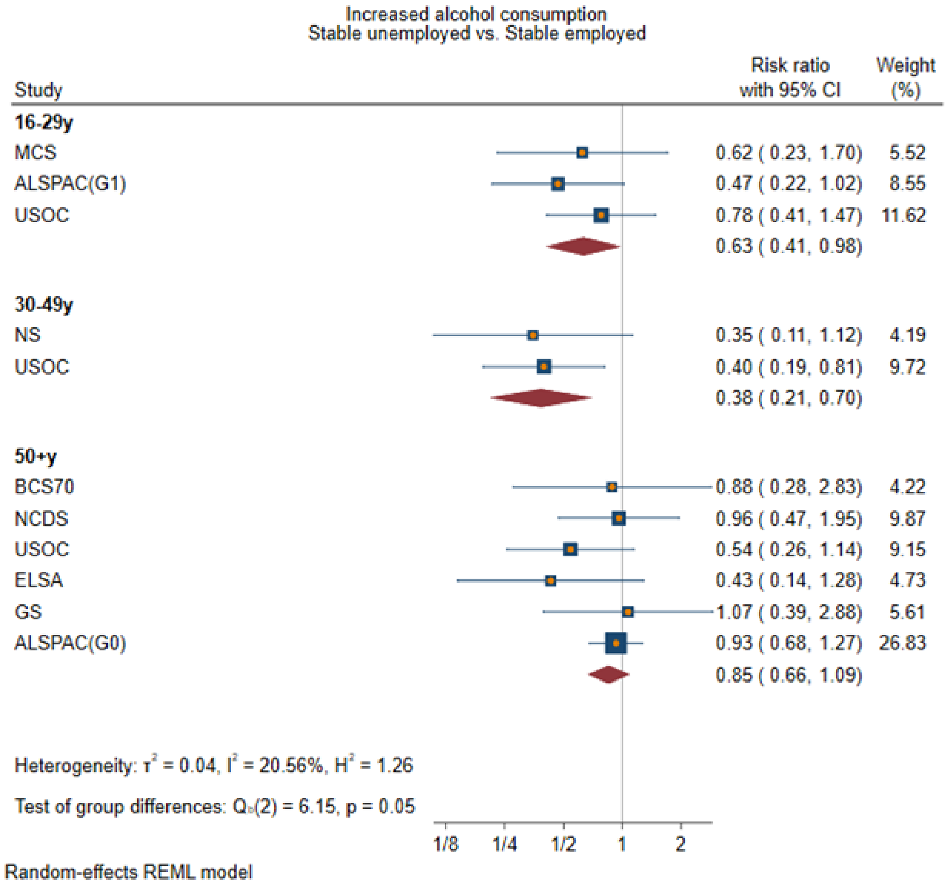
Increased alcohol consumption
Stable unemployed vs. Stable employed
Study
Risk ratio with 95% CI
Weight (%)
16-29y
MCS 0.62 (0.23, 1.70) 5.52
ALSPAC(G1) 0.47 (0.22, 1.02) 8.55
USOC 0.78 (0.41, 1.47) 11.62
0.63 (0.41, 0.98)
30-49y
NS 0.35 (0.11, 1.12) 4.19
USOC 0.40 (0.19, 0.81) 9.72
0.38 (0.21, 0.70)
50+y
BCS70 0.88 (0.28, 2.83) 4.22
NCDS 0.96 (0.47, 1.95) 9.87
USOC 0.54 (0.26, 1.14) 9.15
ELSA 0.43 (0.14, 1.28) 4.73
GS 1.07 (0.39, 2.88) 5.61
ALSPAC(G0) 0.93 (0.68, 1.27) 26.83
0.85 (0.66, 1.09)
Heterogeneity: τ^2 = 0.04, I^2 = 20.56%, H^2 = 1.26
Test of group differences: $Q_b(2)$ = 6.15, p = 0.05
1/8 1/4 1/2 1 2
Random-effects REML model

Figure set 3: Reduced alcohol consumption

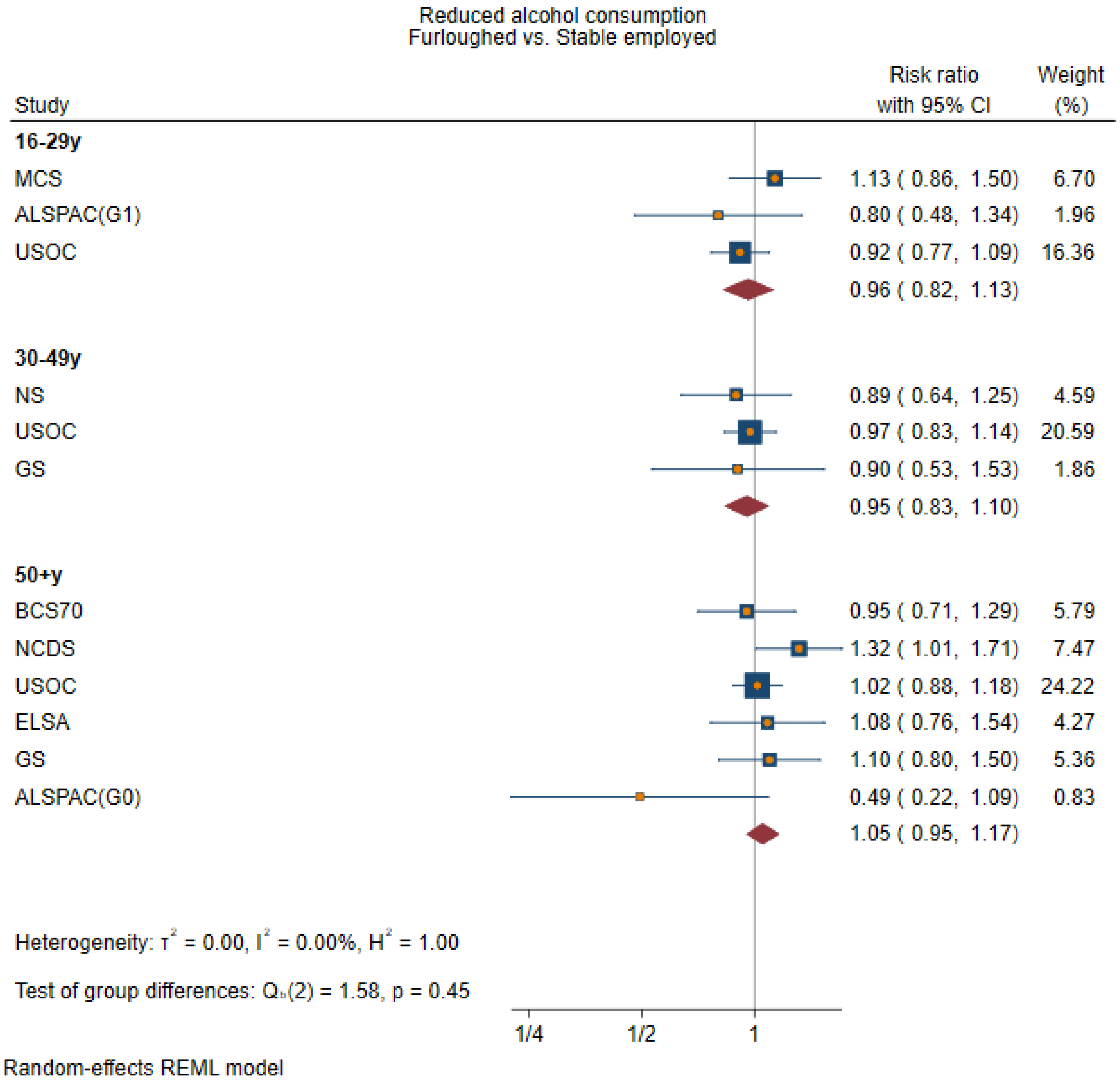

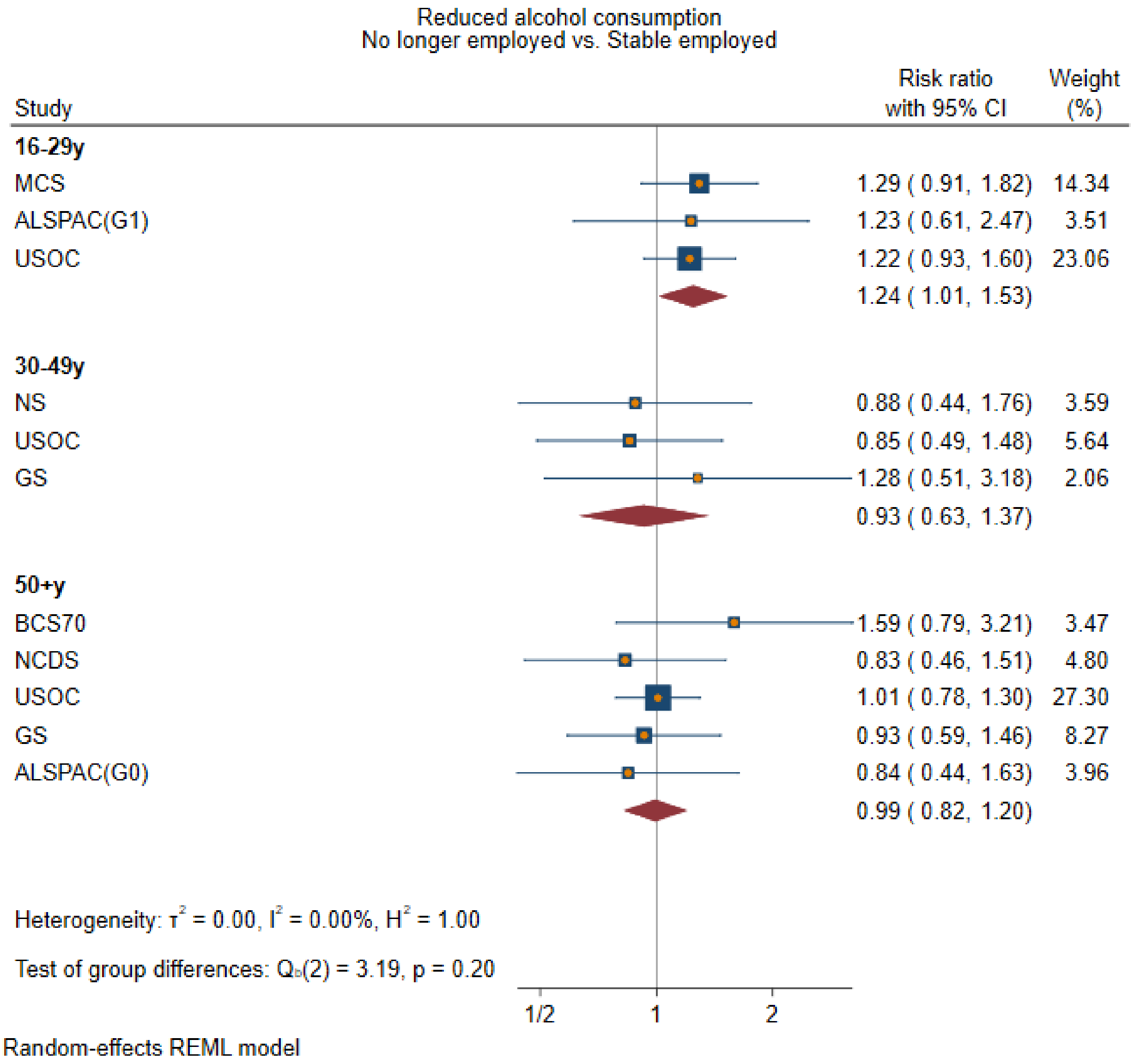
Reduced alcohol consumption
No longer employed vs. Stable employed
Study
Risk ratio with 95% CI
Weight (%)
16-29y
MCS 1.29 (0.91, 1.82) 14.34
ALSPAC(G1) 1.23 (0.61, 2.47) 3.51
USOC 1.22 (0.93, 1.60) 23.06
1.24 (1.01, 1.53)
30-49y
NS 0.88 (0.44, 1.76) 3.59
USOC 0.85 (0.49, 1.48) 5.64
GS 1.28 (0.51, 3.18) 2.06
0.93 (0.63, 1.37)
50+y
BCS70 1.59 (0.79, 3.21) 3.47
NCDS 0.83 (0.46, 1.51) 4.80
USOC 1.01 (0.78, 1.30) 27.30
GS 0.93 (0.59, 1.46) 8.27
ALSPAC(G0) 0.84 (0.44, 1.63) 3.96
0.99 (0.82, 1.20)
Heterogeneity: τ^2 = 0.00, I^2 = 0.00%, H^2 = 1.00
Test of group differences: $Q_b(2)$ = 3.19, p = 0.20
1/2
1
2
Random-effects REML model

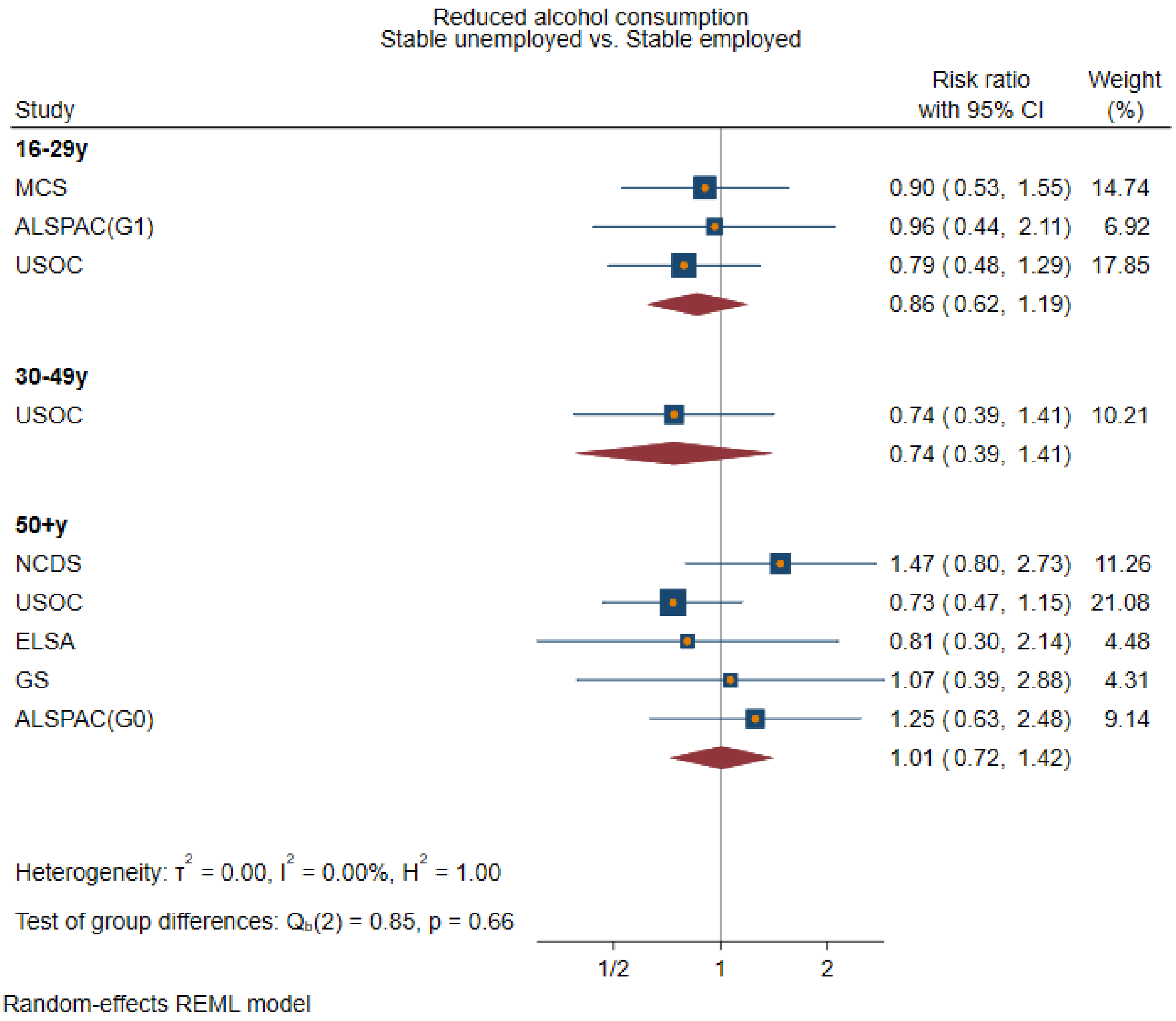
Reduced alcohol consumption
Stable unemployed vs. Stable employed
Study
Risk ratio with 95% CI
Weight (%)
16-29y
MCS 0.90 (0.53, 1.55) 14.74
ALSPAC(G1) 0.96 (0.44, 2.11) 6.92
USOC 0.79 (0.48, 1.29) 17.85
0.86 (0.62, 1.19)
30-49y
USOC 0.74 (0.39, 1.41) 10.21
0.74 (0.39, 1.41)
50+y
NCDS 1.47 (0.80, 2.73) 11.26
USOC 0.73 (0.47, 1.15) 21.08
ELSA 0.81 (0.30, 2.14) 4.48
GS 1.07 (0.39, 2.88) 4.31
ALSPAC(G0) 1.25 (0.63, 2.48) 9.14
1.01 (0.72, 1.42)
Heterogeneity: τ^2 = 0.00, I^2 = 0.00%, H^2 = 1.00
Test of group differences: $Q_b(2)$ = 0.85, p = 0.66
1/2
1
2
Random-effects REML model

Figure set 4: Drinks 5+ drinks/occasion

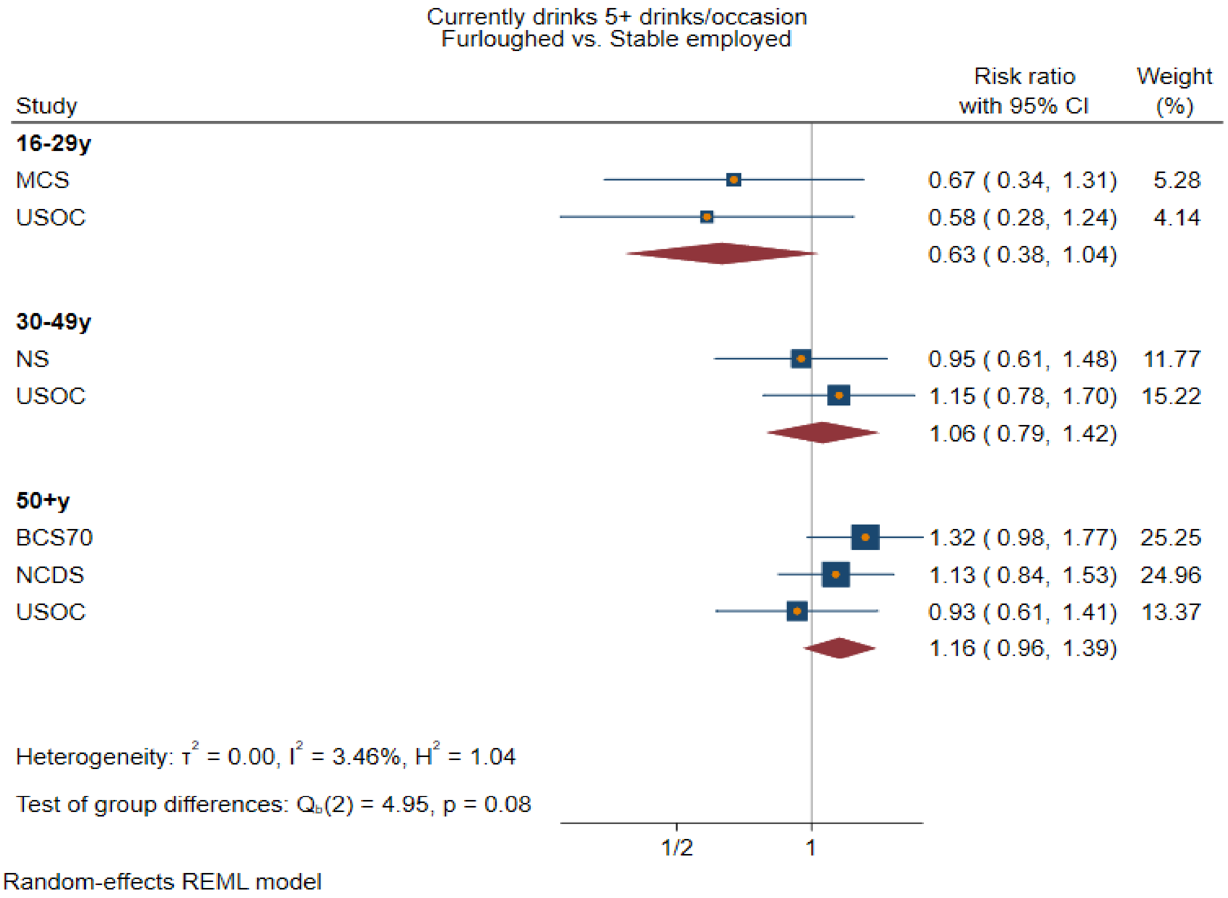

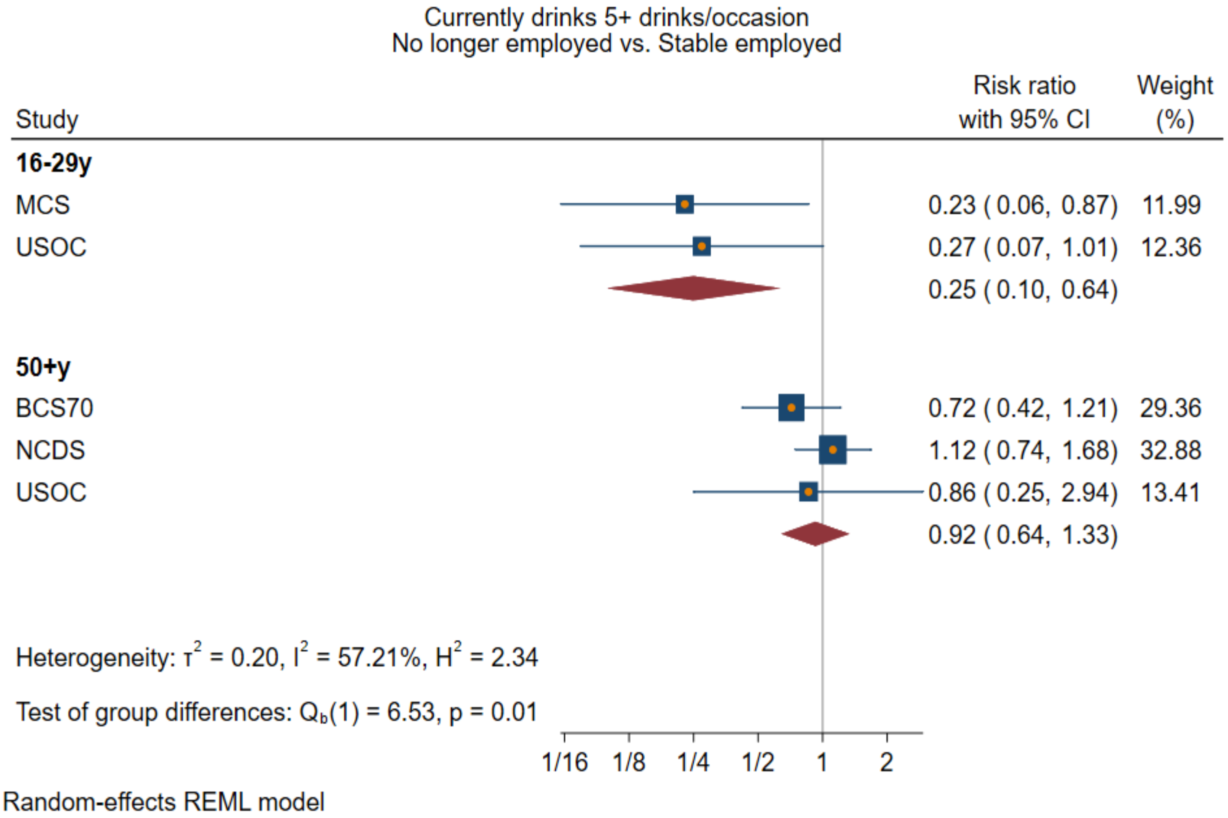

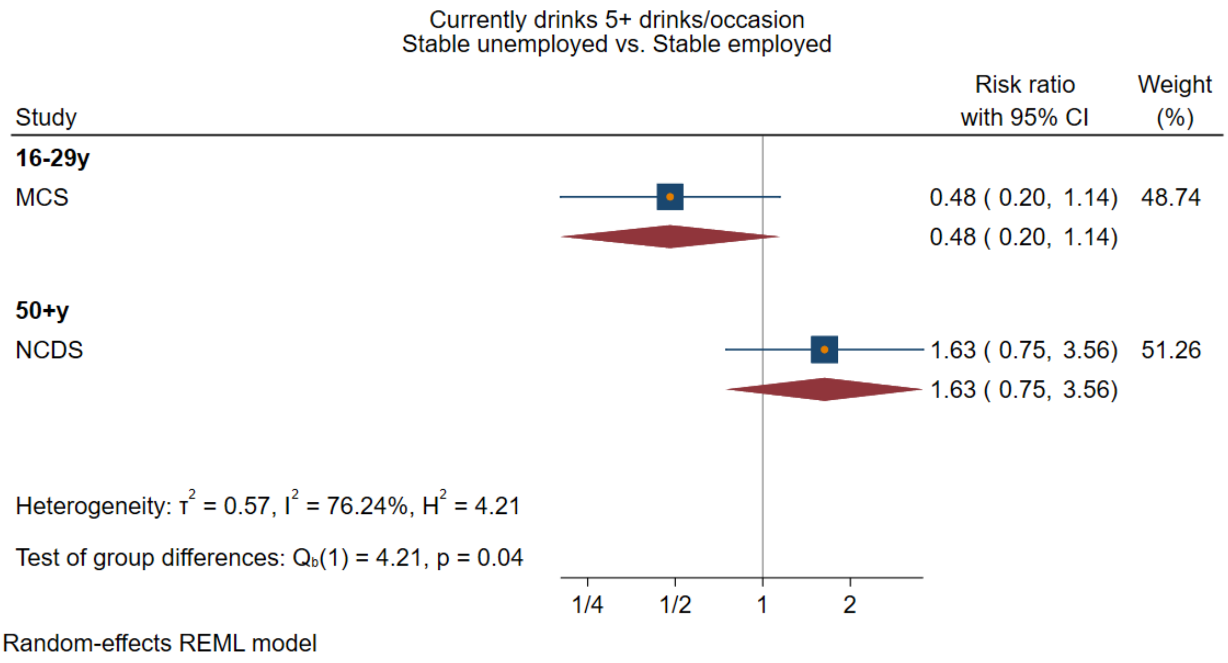

Figure set 5: Drinks more alcohol units per occasion

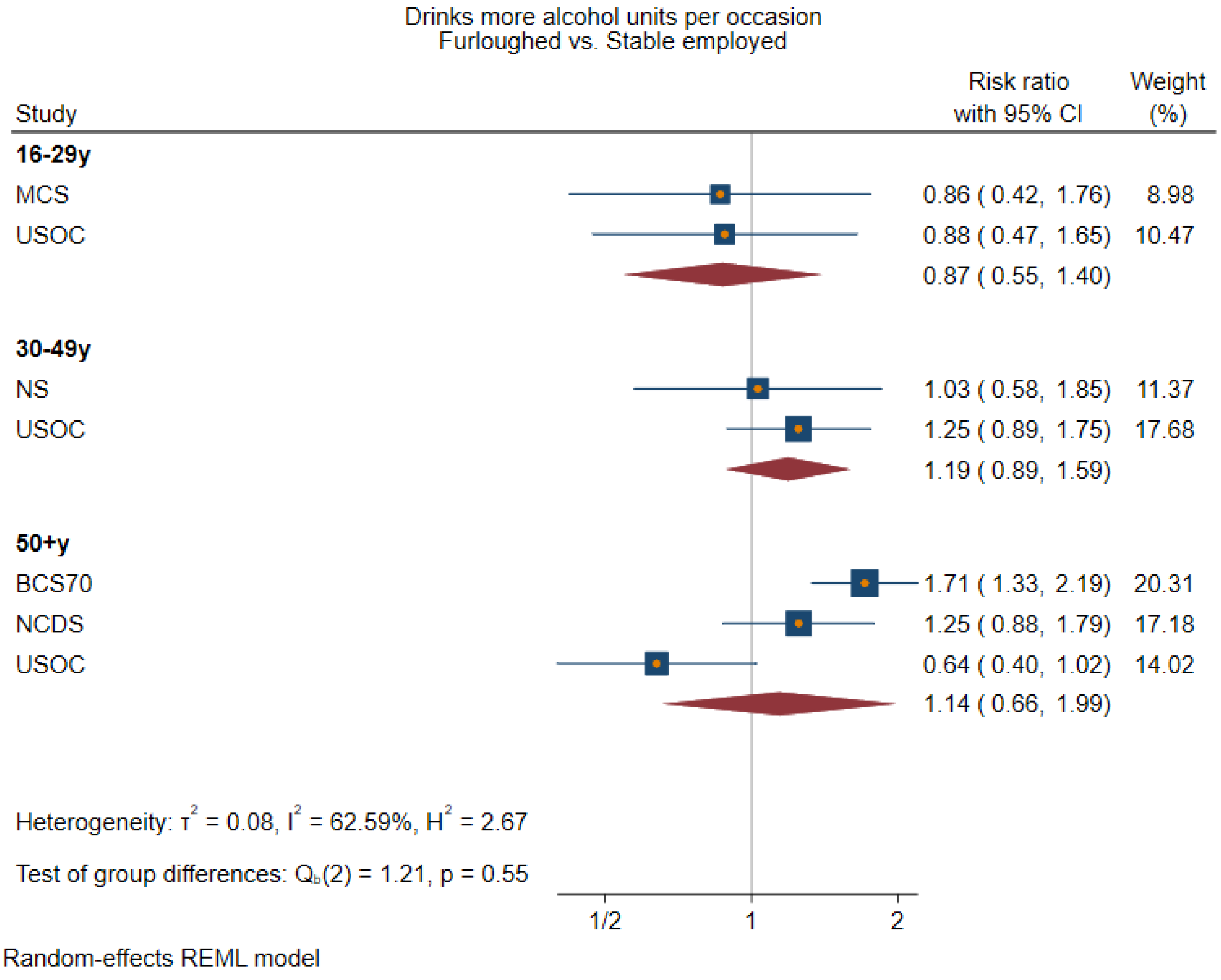

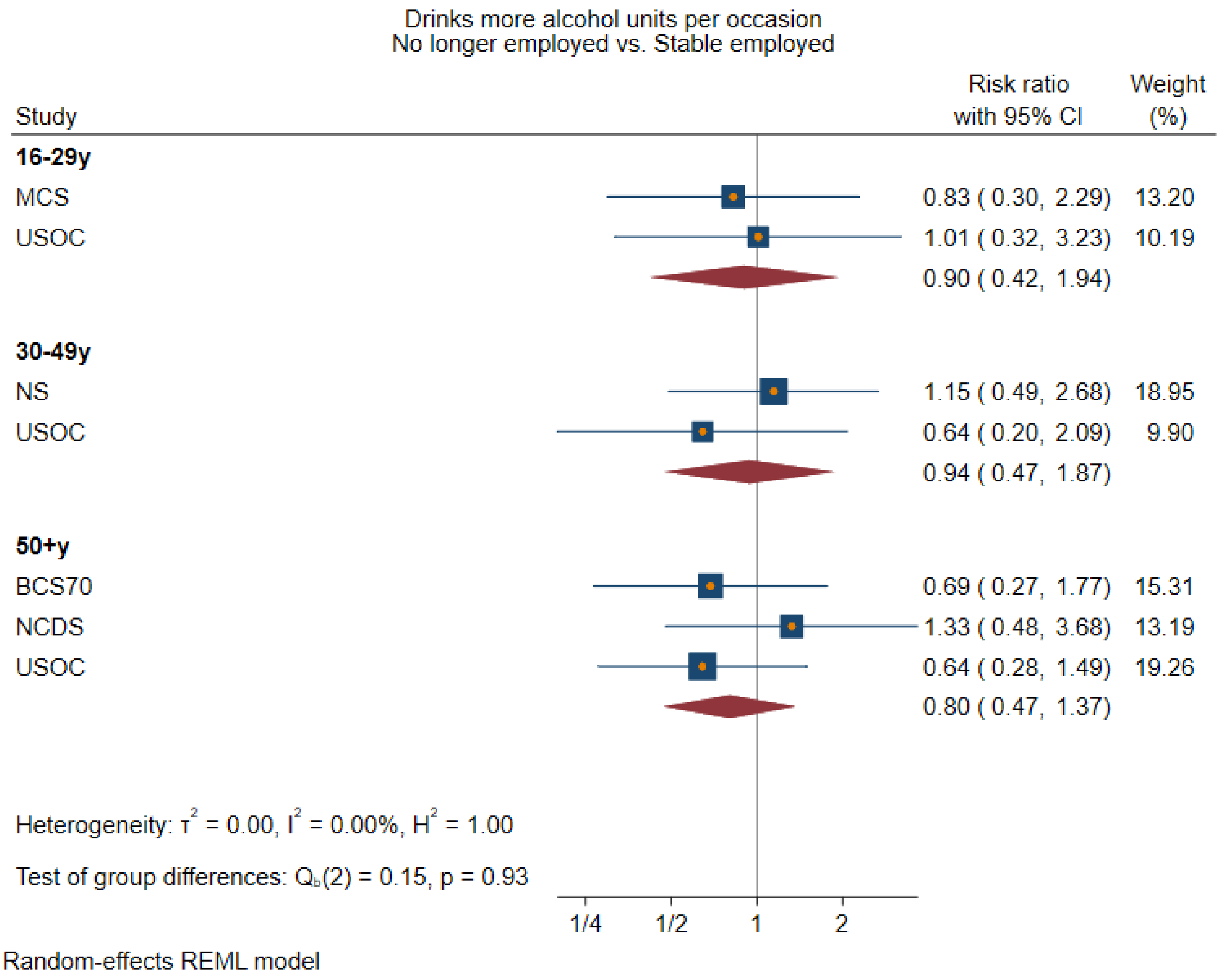
Drinks more alcohol units per occasion
No longer employed vs. Stable employed
Study
Risk ratio with 95% CI
Weight (%)
16-29y
MCS 0.83 (0.30, 2.29) 13.20
USOC 1.01 (0.32, 3.23) 10.19
0.90 (0.42, 1.94)
30-49y
NS 1.15 (0.49, 2.68) 18.95
USOC 0.64 (0.20, 2.09) 9.90
0.94 (0.47, 1.87)
50+y
BCS70 0.69 (0.27, 1.77) 15.31
NCDS 1.33 (0.48, 3.68) 13.19
USOC 0.64 (0.28, 1.49) 19.26
0.80 (0.47, 1.37)
Heterogeneity: τ^2 = 0.00, I^2 = 0.00%, H^2 = 1.00
Test of group differences: $Q_b(2)$ = 0.15, p = 0.93
1/4
1/2
1
2
Random-effects REML model

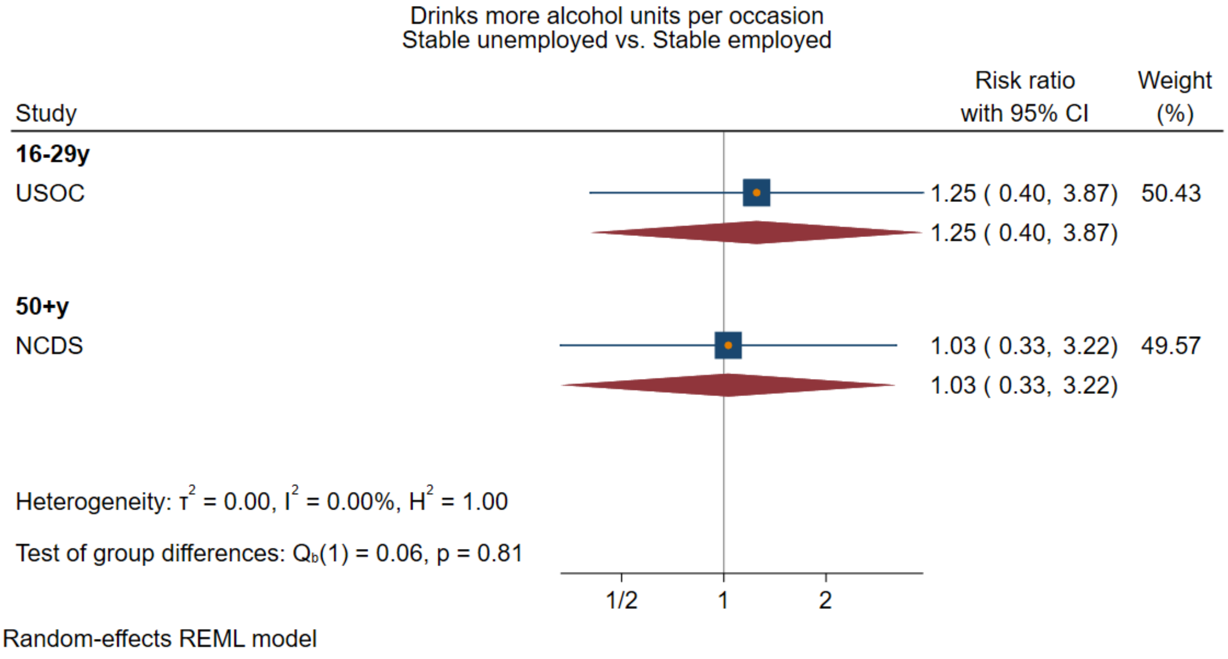
Drinks more alcohol units per occasion
Stable unemployed vs. Stable employed
Study
Risk ratio with 95% CI
Weight (%)
16-29y
USOC 1.25 (0.40, 3.87) 50.43
1.25 (0.40, 3.87)
50+y
NCDS 1.03 (0.33, 3.22) 49.57
1.03 (0.33, 3.22)
Heterogeneity: τ^2 = 0.00, I^2 = 0.00%, H^2 = 1.00
Test of group differences: $Q_b(1)$ = 0.06, p = 0.81
1/2
1
2
Random-effects REML model

Figure set 6: Drinks fewer alcohol units per occasion

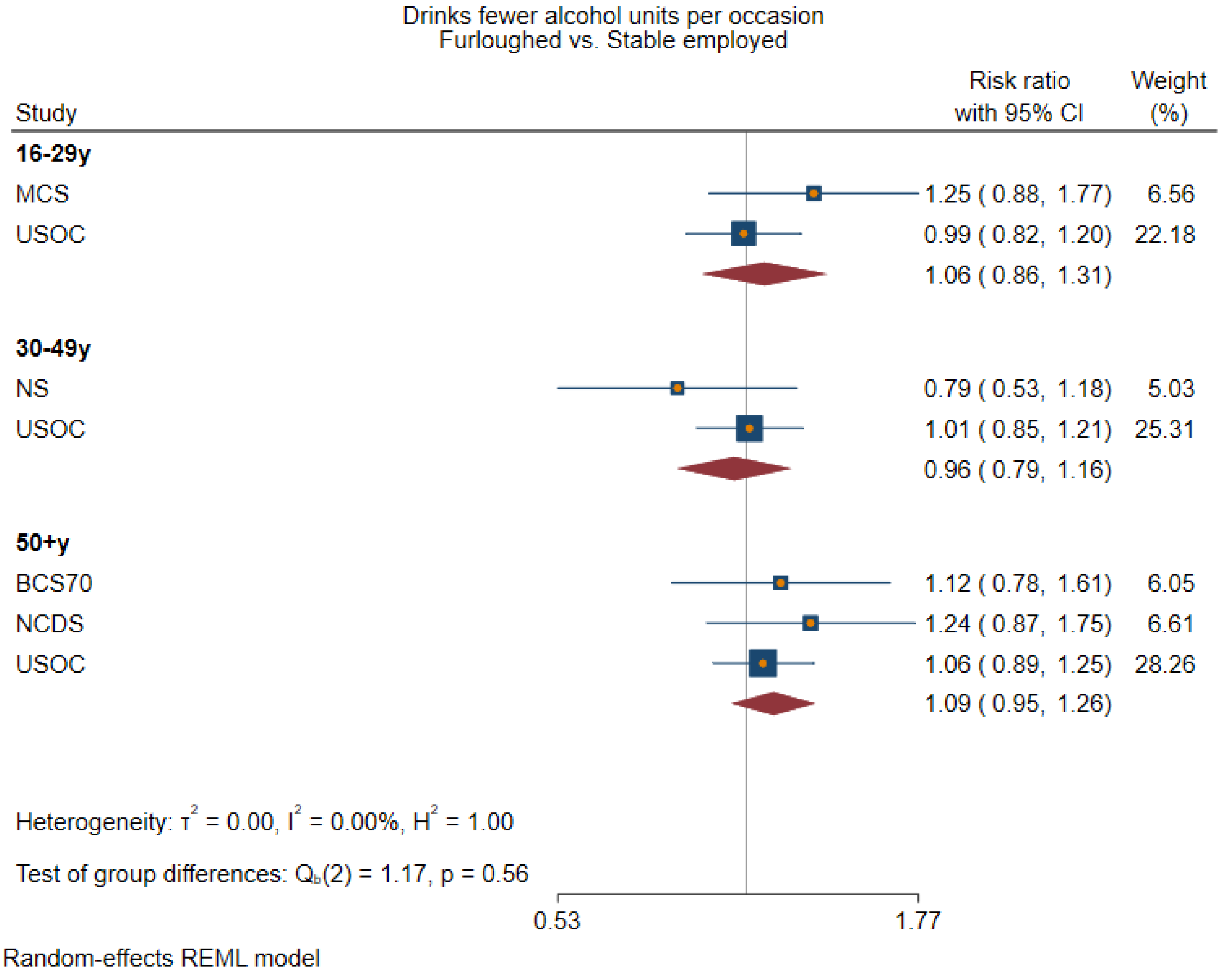

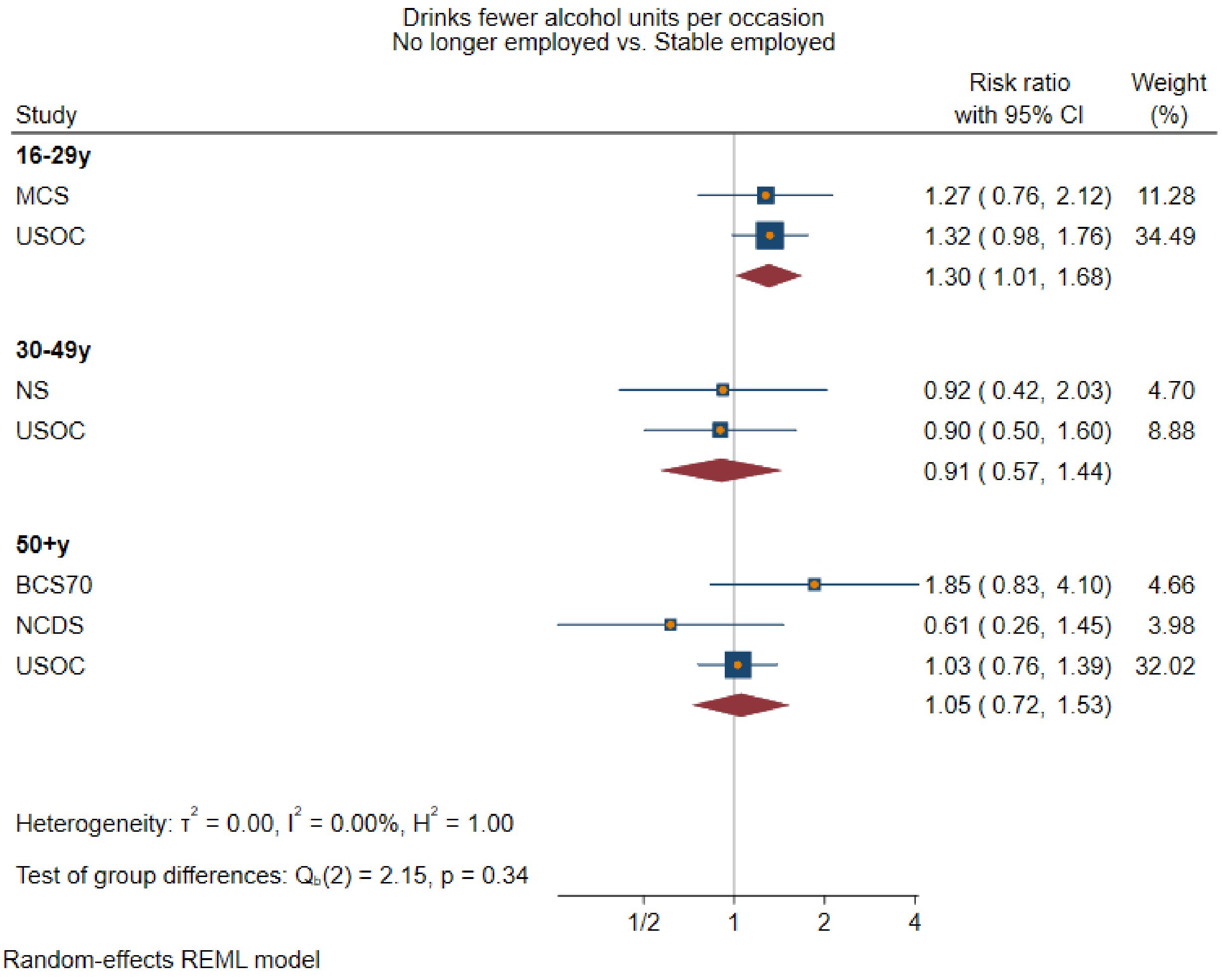

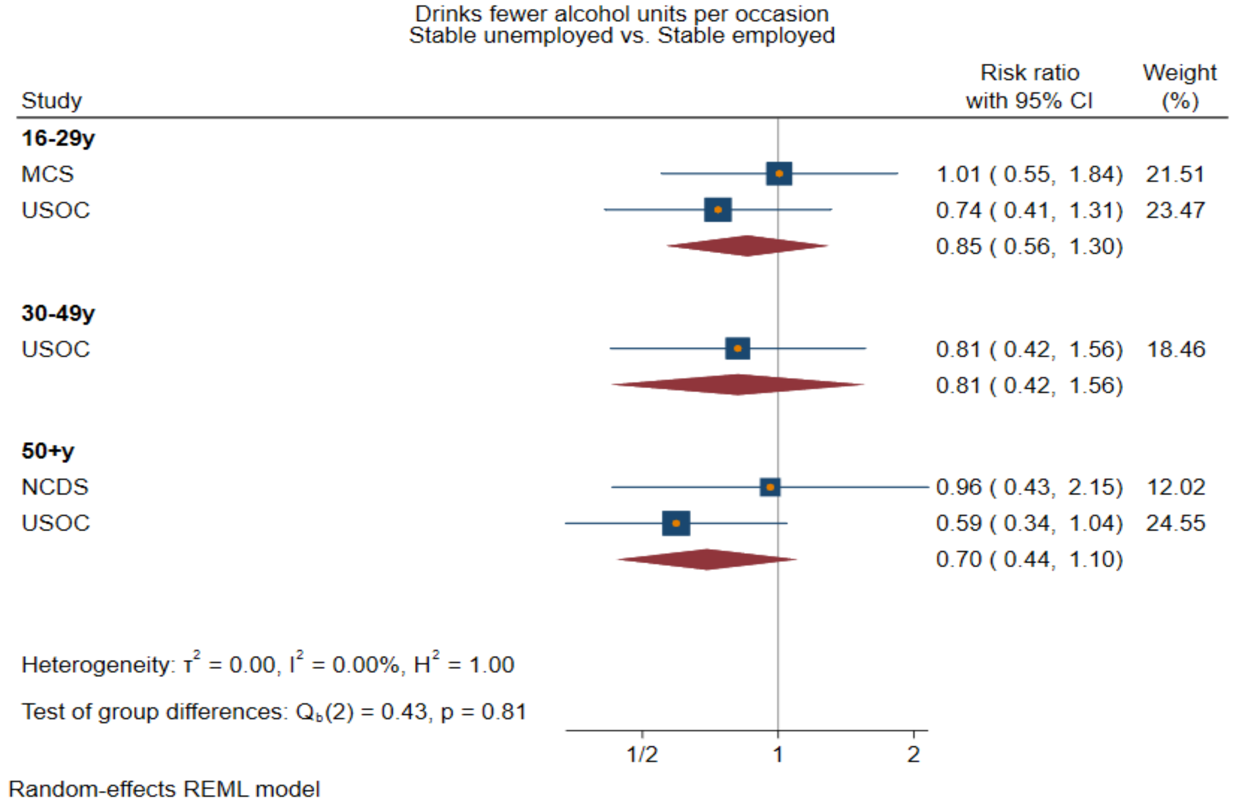

Drinks fewer alcohol units per occasion
Stable unemployed vs. Stable employed
Study
Risk ratio with 95% CI
Weight (%)
16-29y
MCS 1.01 (0.55, 1.84) 21.51
USOC 0.74 (0.41, 1.31) 23.47
0.85 (0.56, 1.30)
30-49y
USOC 0.81 (0.42, 1.56) 18.46
0.81 (0.42, 1.56)
50+y
NCDS 0.96 (0.43, 2.15) 12.02
USOC 0.59 (0.34, 1.04) 24.55
0.70 (0.44, 1.10)
Heterogeneity: τ² = 0.00, I² = 0.00%, H² = 1.00
Test of group differences: Q_b(2) = 0.43, p = 0.81
1/2
1
2
Random-effects REML model

Figure set 7: Currently drinks 4+ days/week

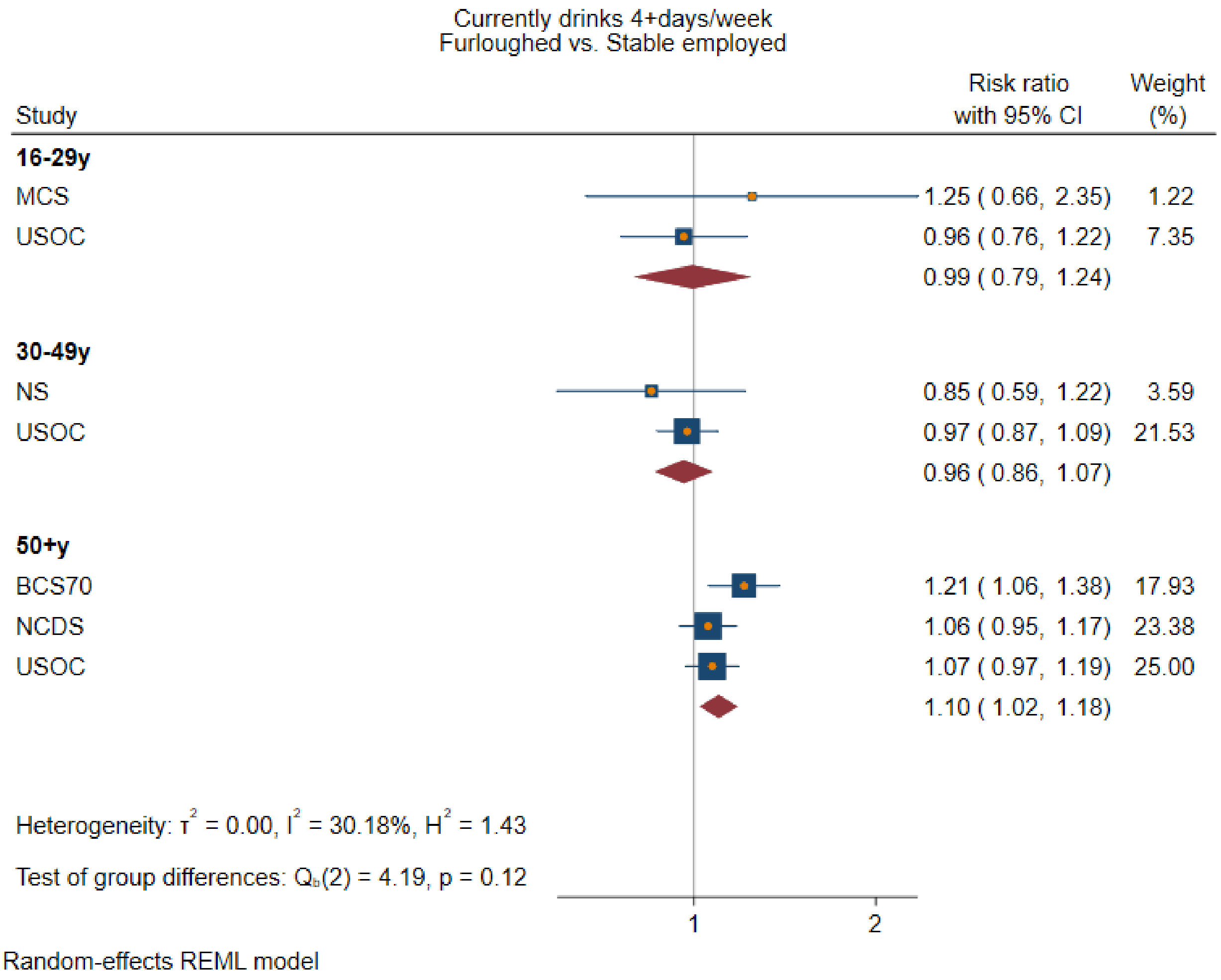

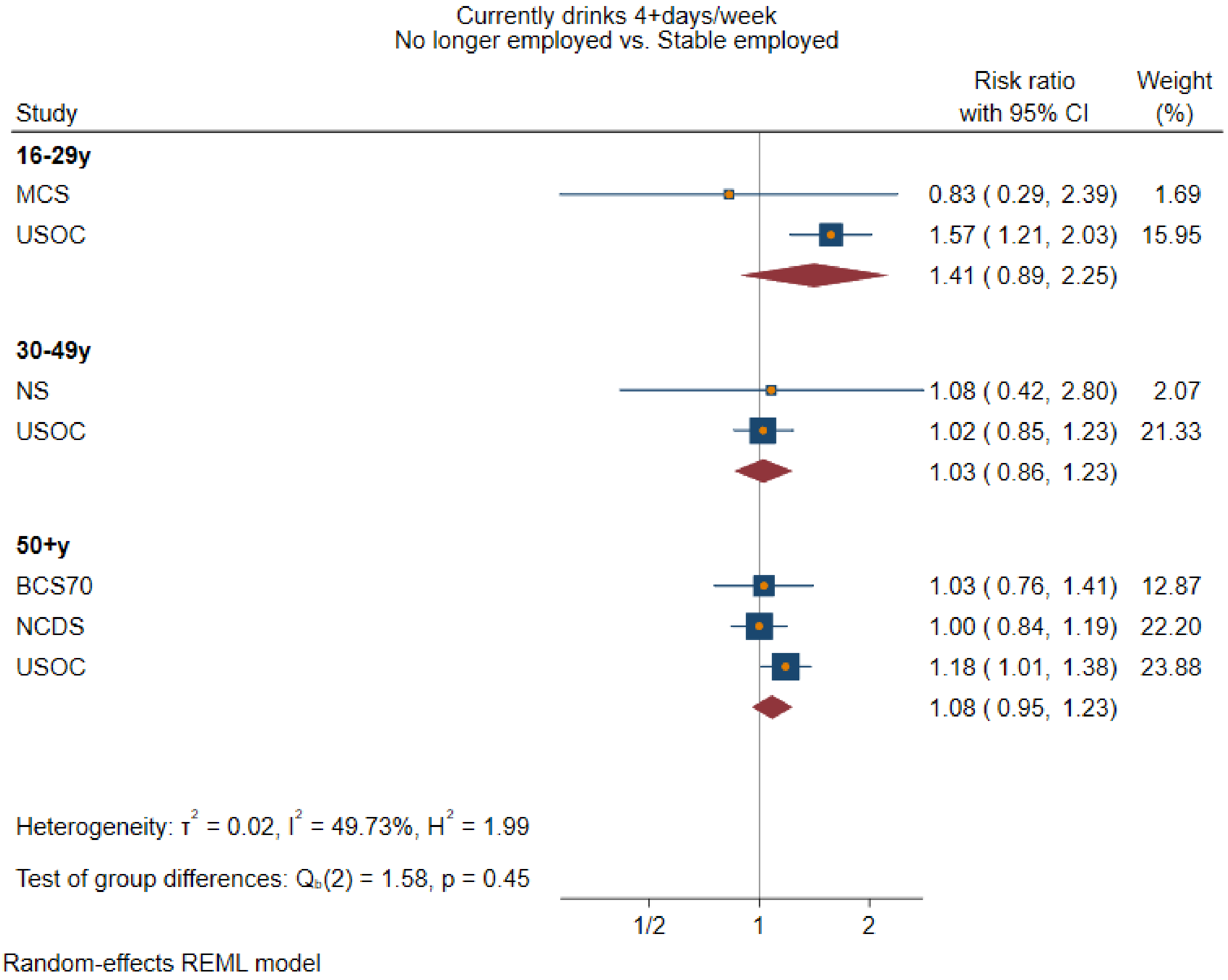

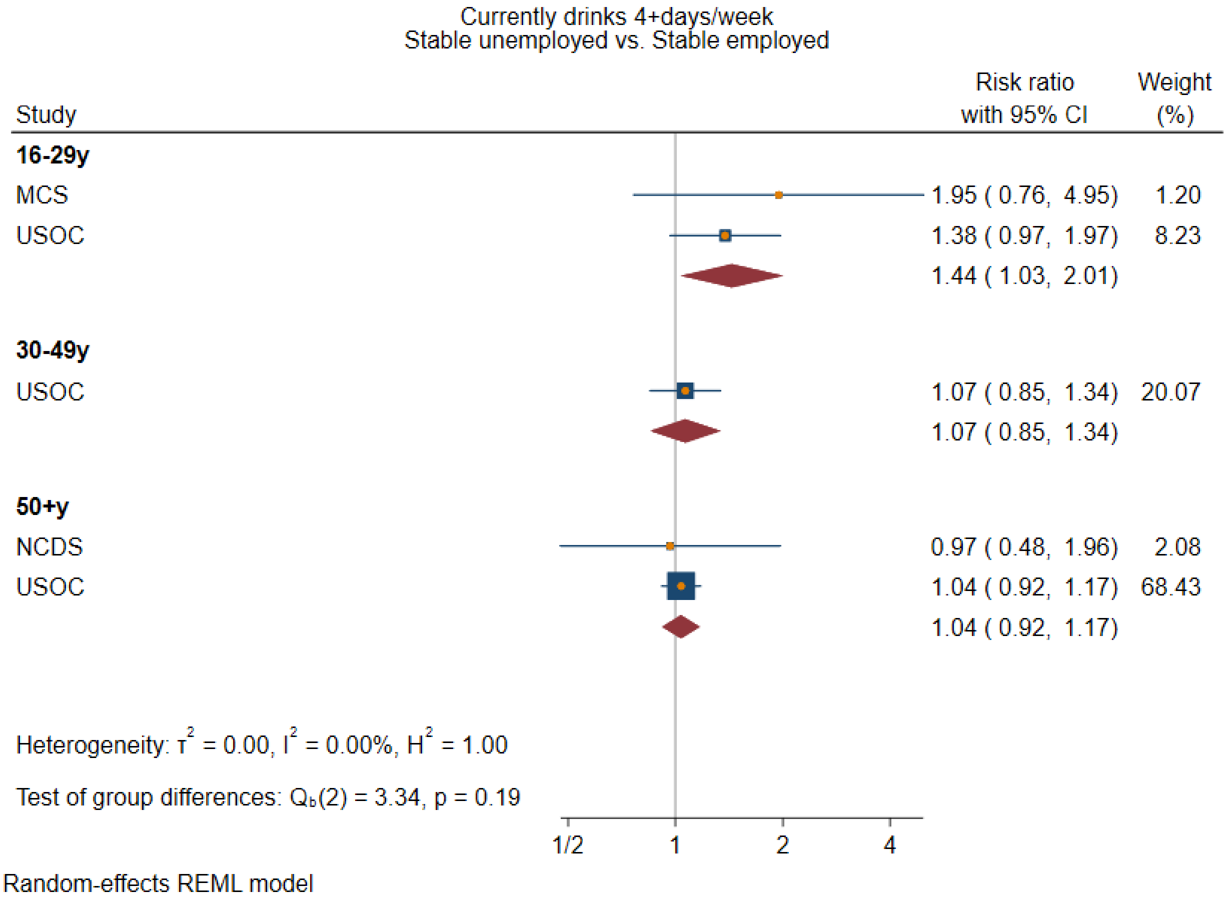
Currently drinks 4+days/week
Stable unemployed vs. Stable employed
Study
Risk ratio with 95% CI
Weight (%)
16-29y
MCS
1.95 (0.76, 4.95)
1.20
USOC
1.38 (0.97, 1.97)
8.23
1.44 (1.03, 2.01)
30-49y
USOC
1.07 (0.85, 1.34)
20.07
1.07 (0.85, 1.34)
50+y
NCDS
0.97 (0.48, 1.96)
2.08
USOC
1.04 (0.92, 1.17)
68.43
1.04 (0.92, 1.17)
Heterogeneity: τ² = 0.00, I² = 0.00%, H² = 1.00
Test of group differences: Q_b(2) = 3.34, p = 0.19
1/2
1
2
4
Random-effects REML model

Figure set 8: Drinks more frequently

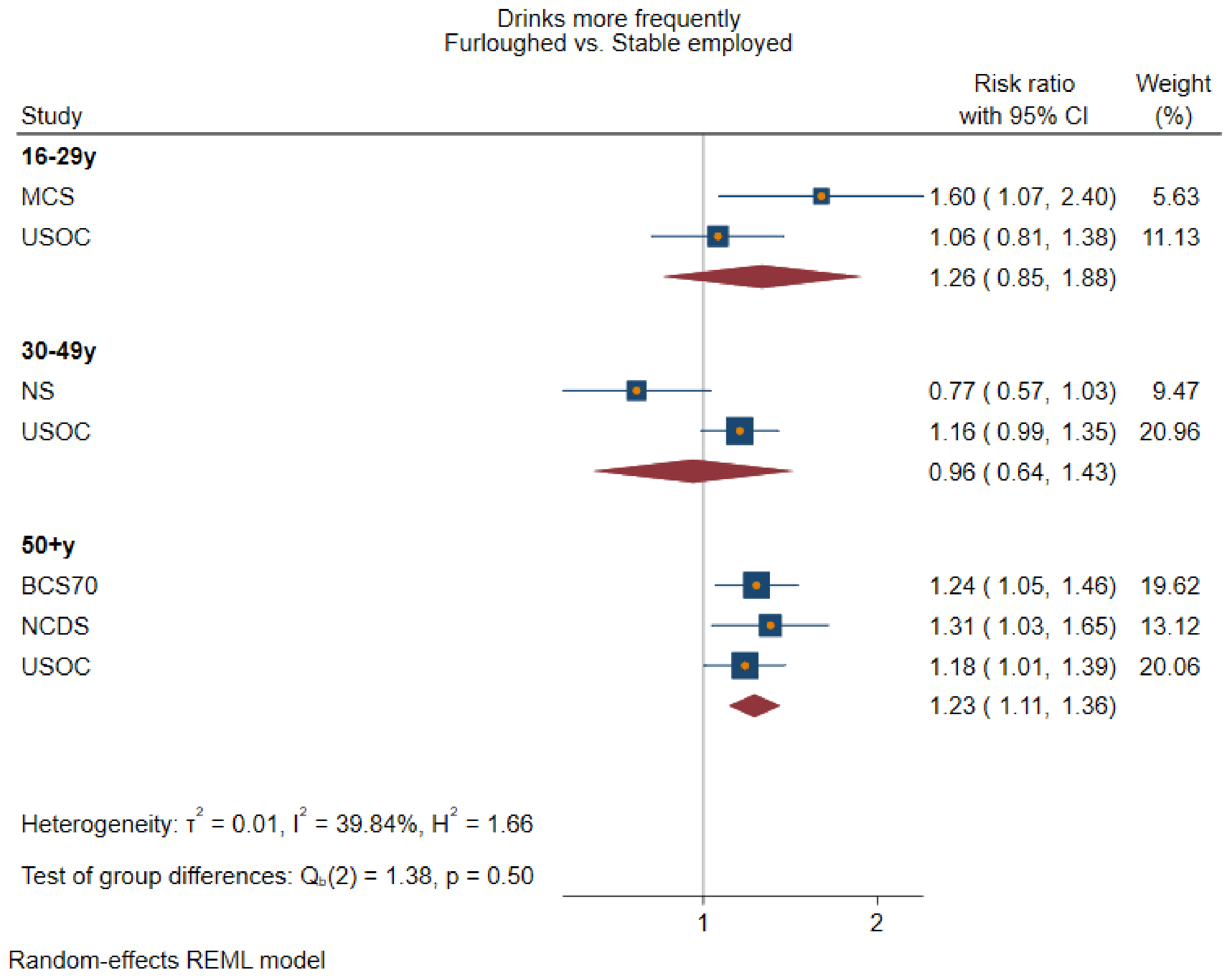

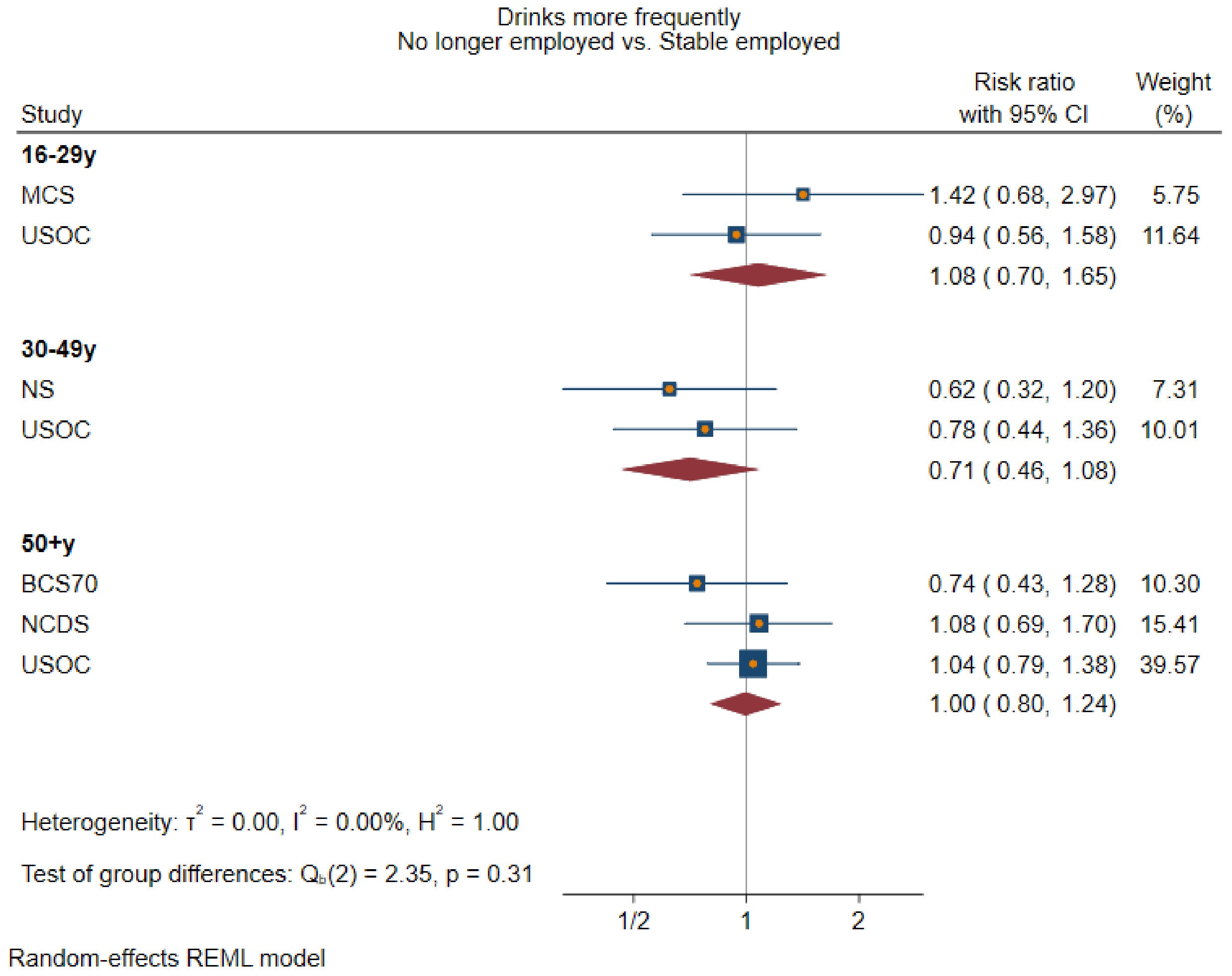

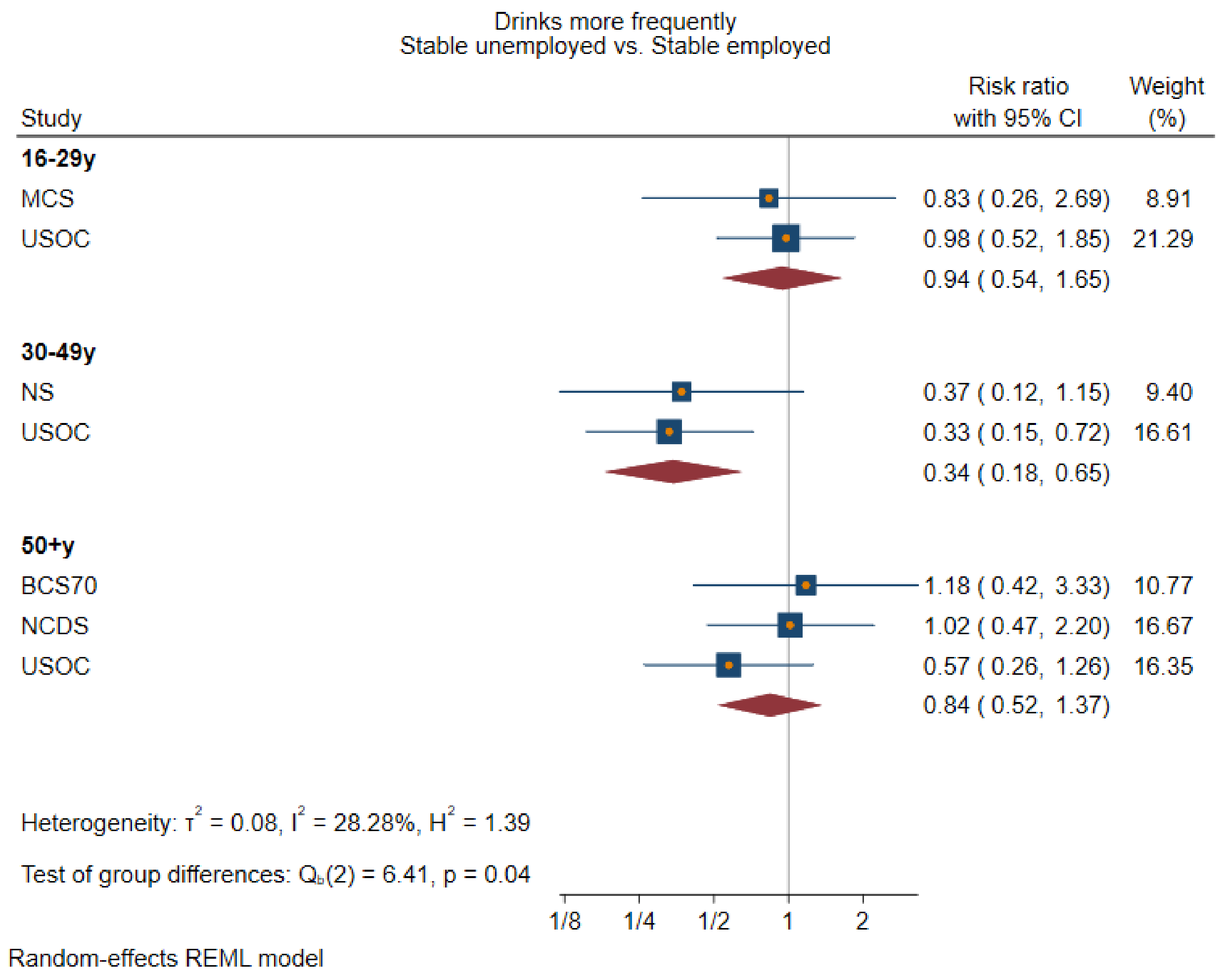
Drinks more frequently
Stable unemployed vs. Stable employed
Study
Risk ratio with 95% CI
Weight (%)
16-29y
MCS
0.83 (0.26, 2.69) 8.91
USOC
0.98 (0.52, 1.85) 21.29
0.94 (0.54, 1.65)
30-49y
NS
0.37 (0.12, 1.15) 9.40
USOC
0.33 (0.15, 0.72) 16.61
0.34 (0.18, 0.65)
50+y
BCS70
1.18 (0.42, 3.33) 10.77
NCDS
1.02 (0.47, 2.20) 16.67
USOC
0.57 (0.26, 1.26) 16.35
0.84 (0.52, 1.37)
Heterogeneity: τ² = 0.08, I² = 28.28%, H² = 1.39
Test of group differences: Qb(2) = 6.41, p = 0.04
1/8
1/4
1/2
1
2
Random-effects REML model

Figure set 9: Drinks less frequently

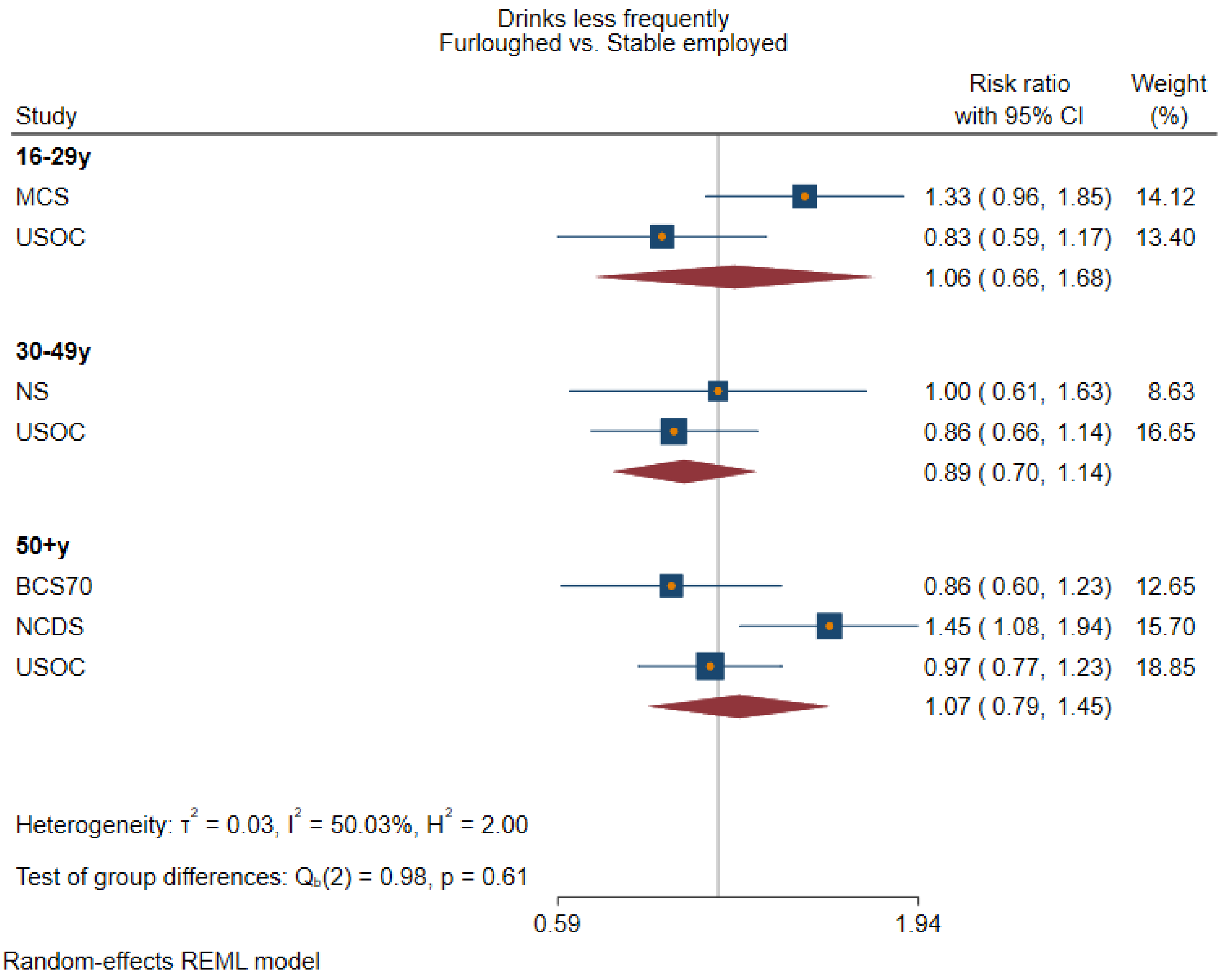

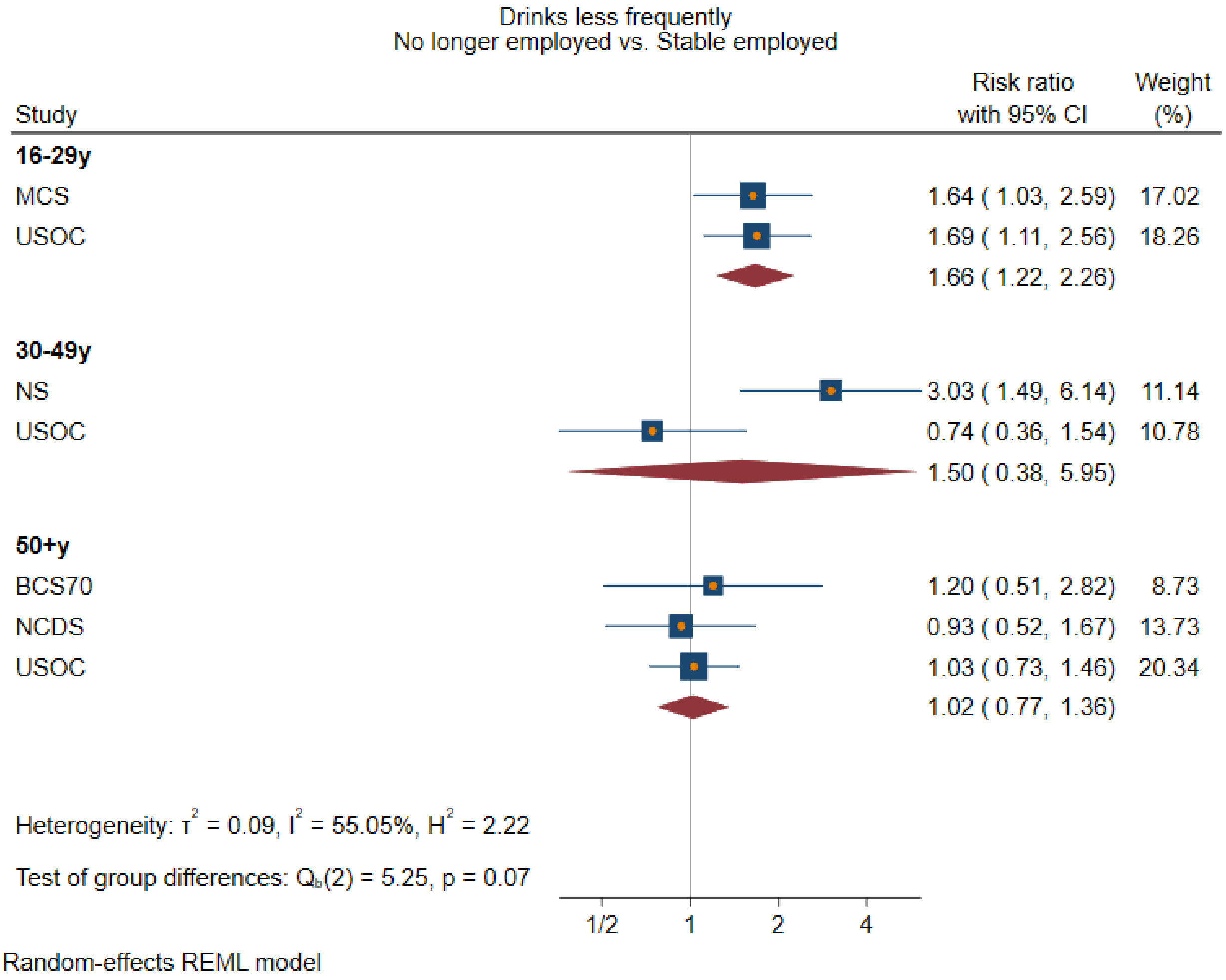

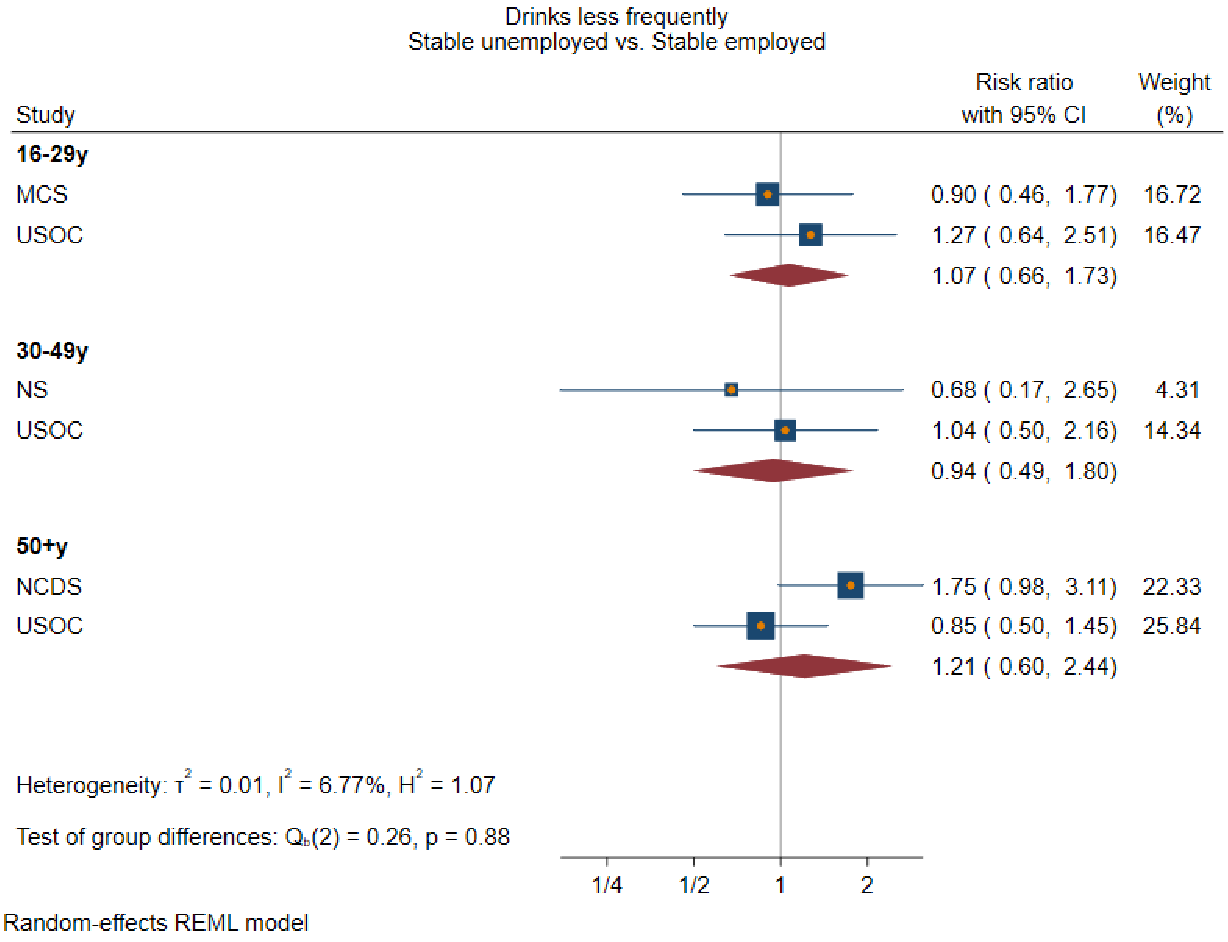
Drinks less frequently
Stable unemployed vs. Stable employed
Study
Risk ratio with 95% CI
Weight (%)
16-29y
MCS 0.90 (0.46, 1.77) 16.72
USOC 1.27 (0.64, 2.51) 16.47
1.07 (0.66, 1.73)
30-49y
NS 0.68 (0.17, 2.65) 4.31
USOC 1.04 (0.50, 2.16) 14.34
0.94 (0.49, 1.80)
50+y
NCDS 1.75 (0.98, 3.11) 22.33
USOC 0.85 (0.50, 1.45) 25.84
1.21 (0.60, 2.44)
Heterogeneity: τ² = 0.01, I² = 6.77%, H² = 1.07
Test of group differences: Q_b(2) = 0.26, p = 0.88
1/4
1/2
1
2
Random-effects REML model

Figure set 10: Current smoker

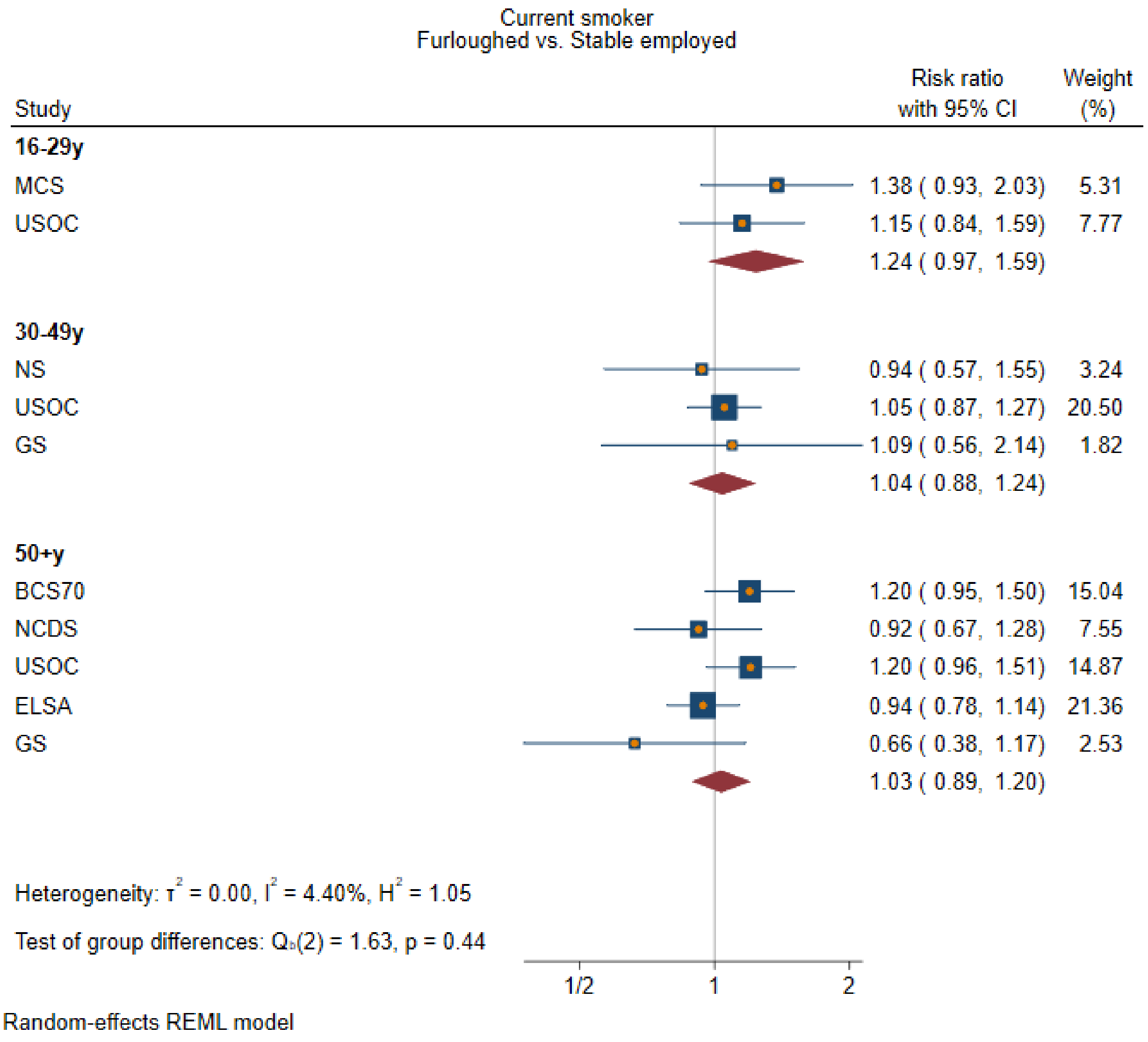

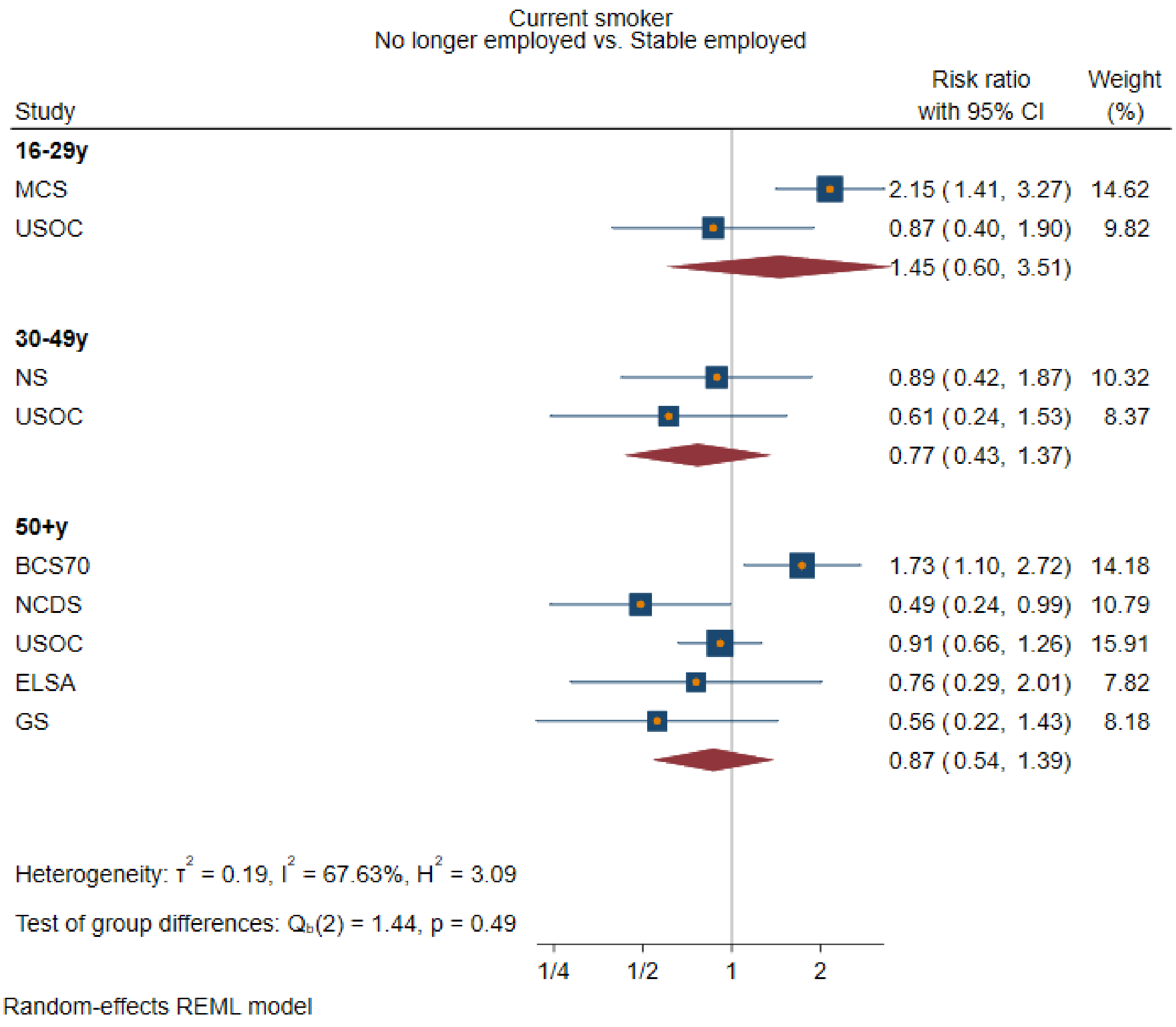

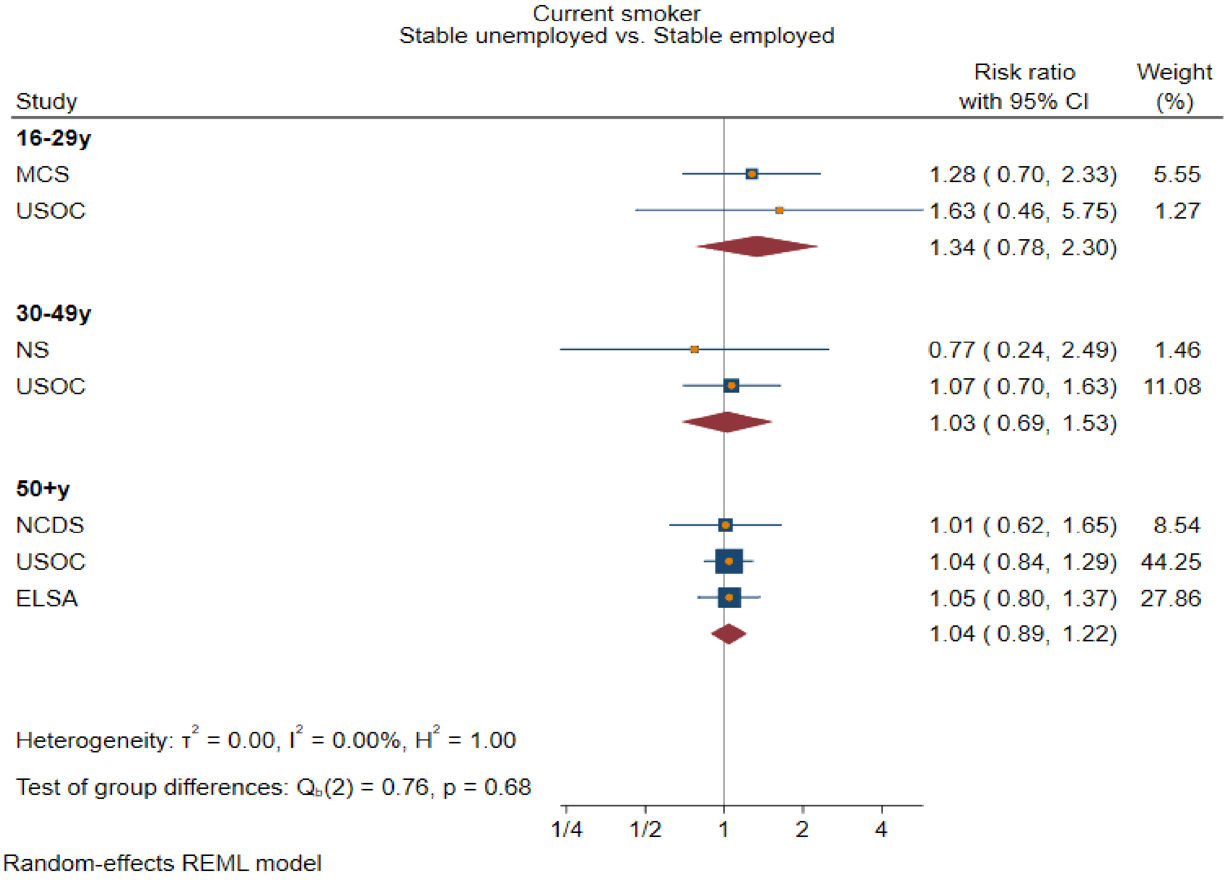
Current smoker
Stable unemployed vs. Stable employed
Study
Risk ratio with 95% CI
Weight (%)
16-29y
MCS 1.28 (0.70, 2.33) 5.55
USOC 1.63 (0.46, 5.75) 1.27
1.34 (0.78, 2.30)
30-49y
NS 0.77 (0.24, 2.49) 1.46
USOC 1.07 (0.70, 1.63) 11.08
1.03 (0.69, 1.53)
50+y
NCDS 1.01 (0.62, 1.65) 8.54
USOC 1.04 (0.84, 1.29) 44.25
ELSA 1.05 (0.80, 1.37) 27.86
1.04 (0.89, 1.22)
Heterogeneity: τ^2 = 0.00, I^2 = 0.00%, H^2 = 1.00
Test of group differences: $Q_b(2)$ = 0.76, p = 0.68
1/4
1/2
1
2
4
Random-effects REML model

Figure set 11: Smoking more

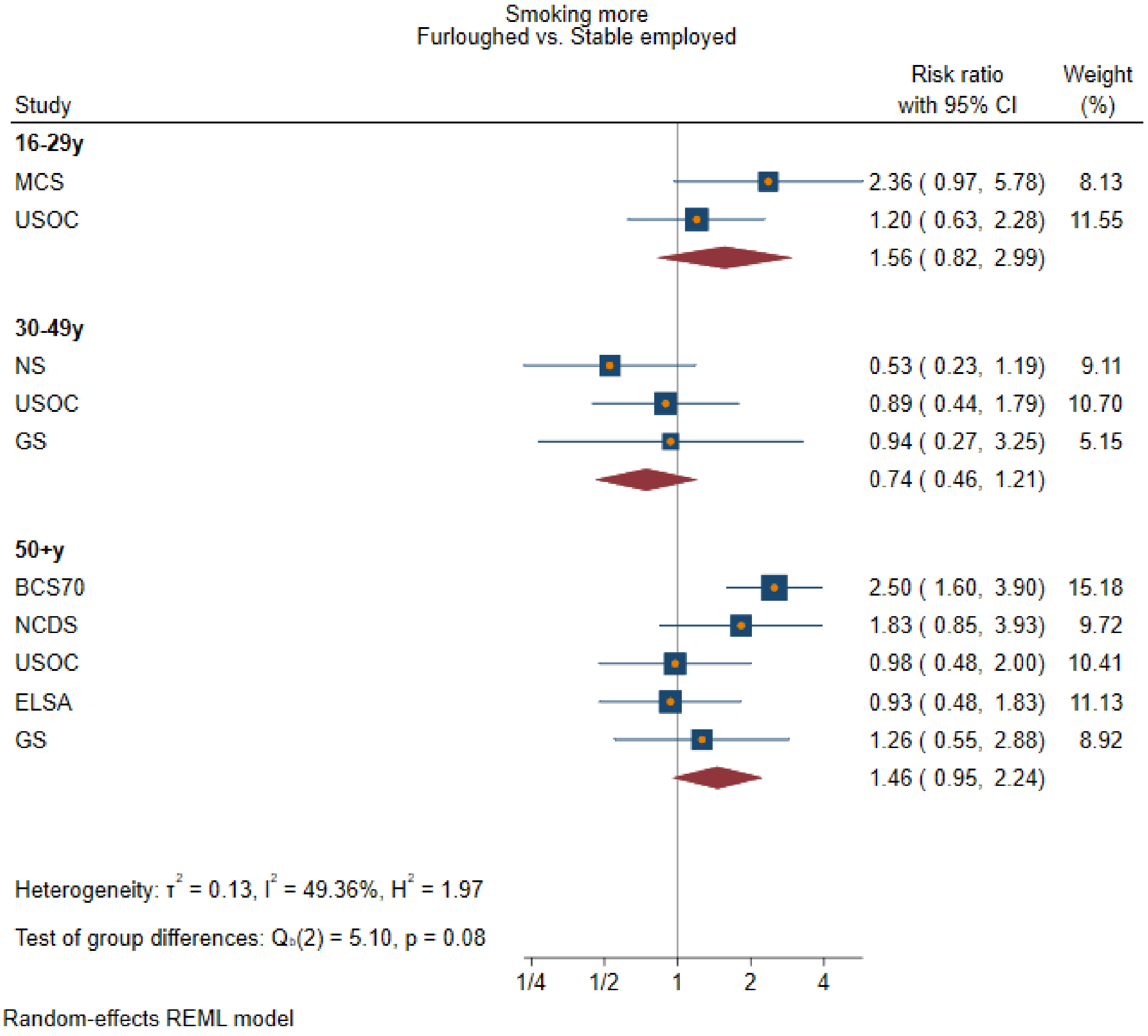

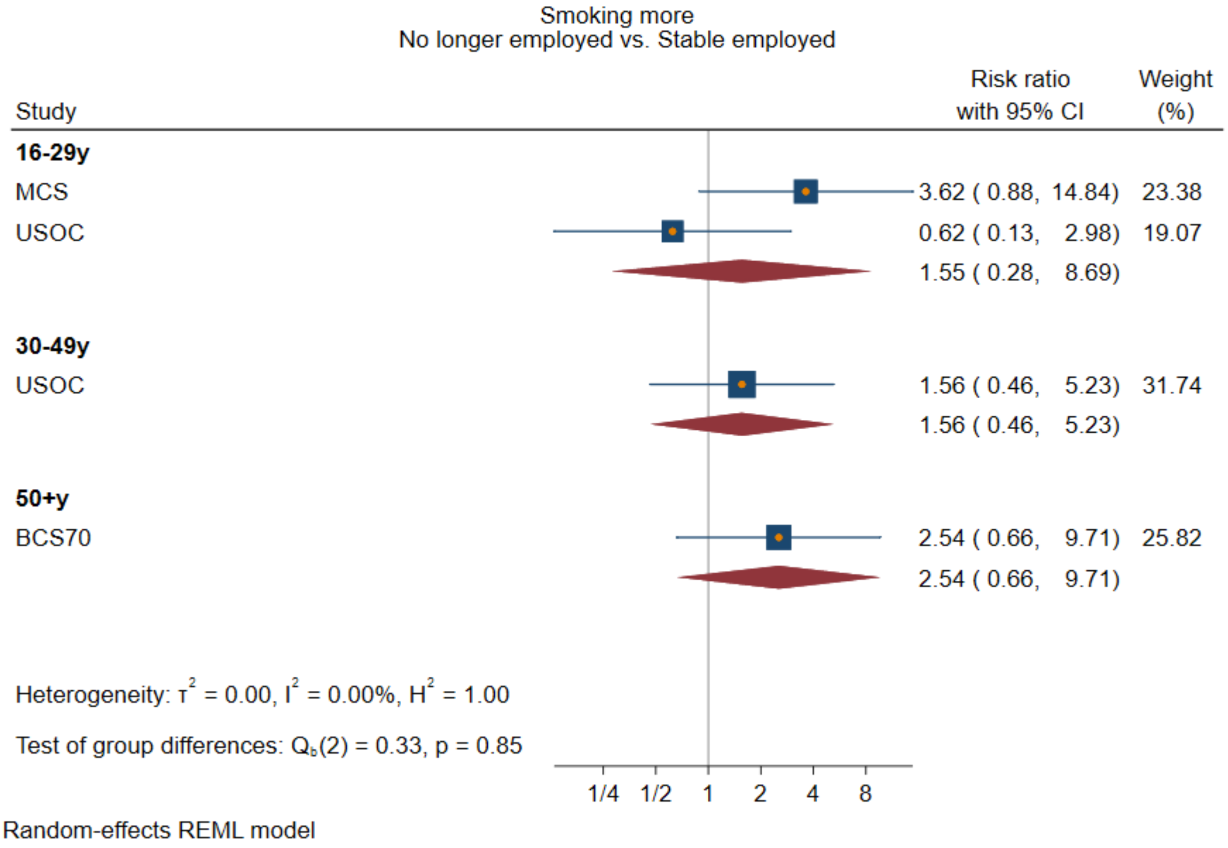

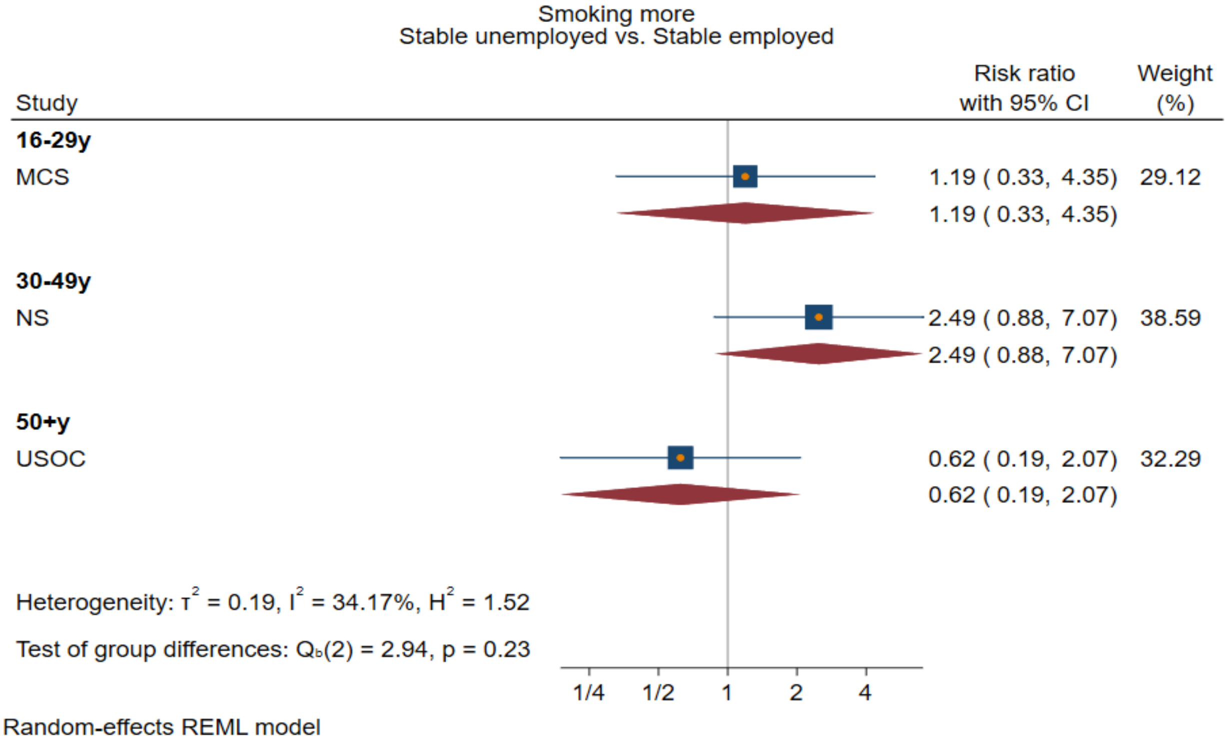
Smoking more
Stable unemployed vs. Stable employed
Study
Risk ratio with 95% CI
Weight (%)
16-29y
MCS
1.19 (0.33, 4.35) 29.12
1.19 (0.33, 4.35)
30-49y
NS
2.49 (0.88, 7.07) 38.59
2.49 (0.88, 7.07)
50+y
USOC
0.62 (0.19, 2.07) 32.29
0.62 (0.19, 2.07)
Heterogeneity: τ² = 0.19, I² = 34.17%, H² = 1.52
Test of group differences: Qb(2) = 2.94, p = 0.23
1/4
1/2
1
2
4
Random-effects REML model

Figure set 12: Smoking less

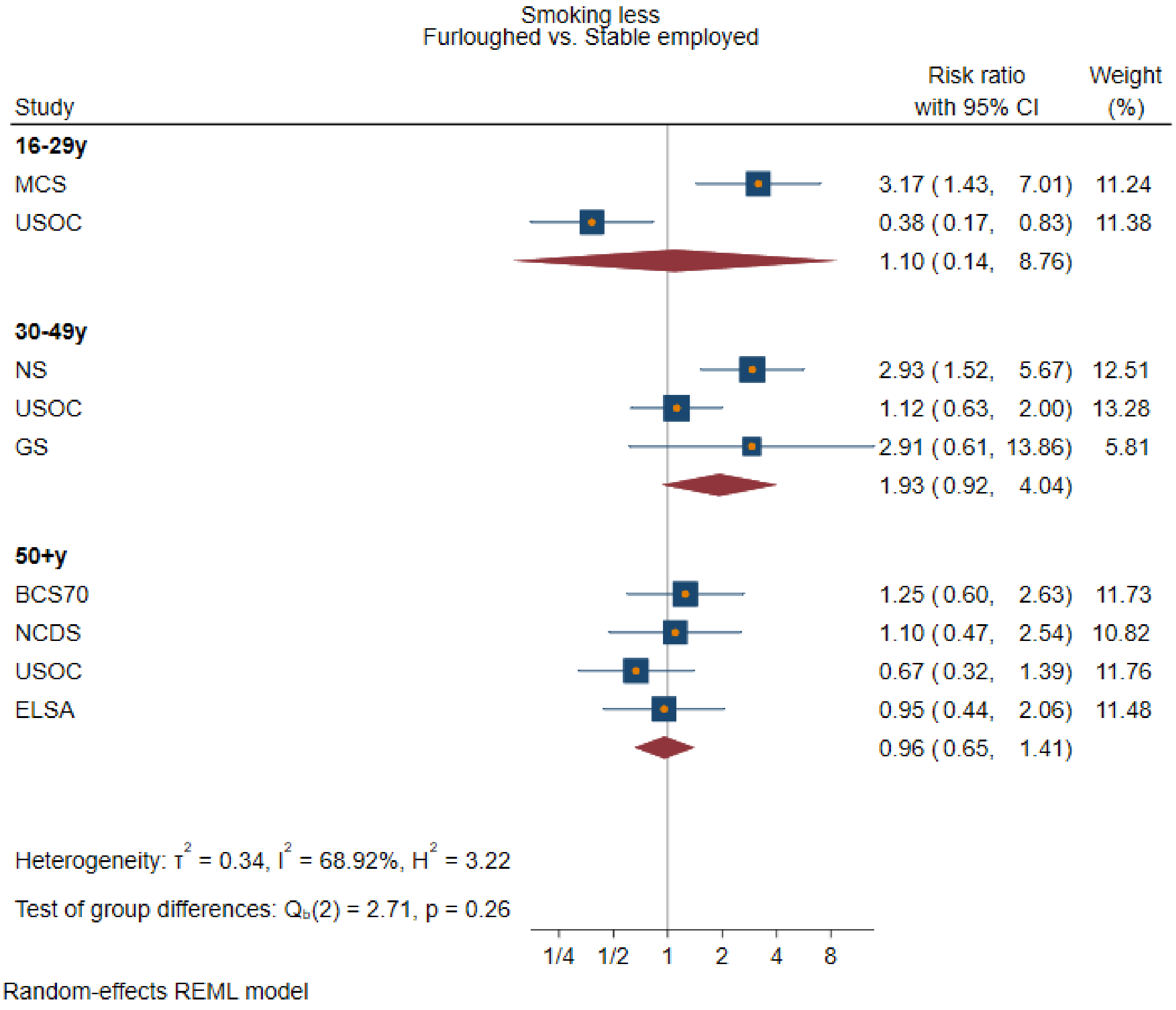

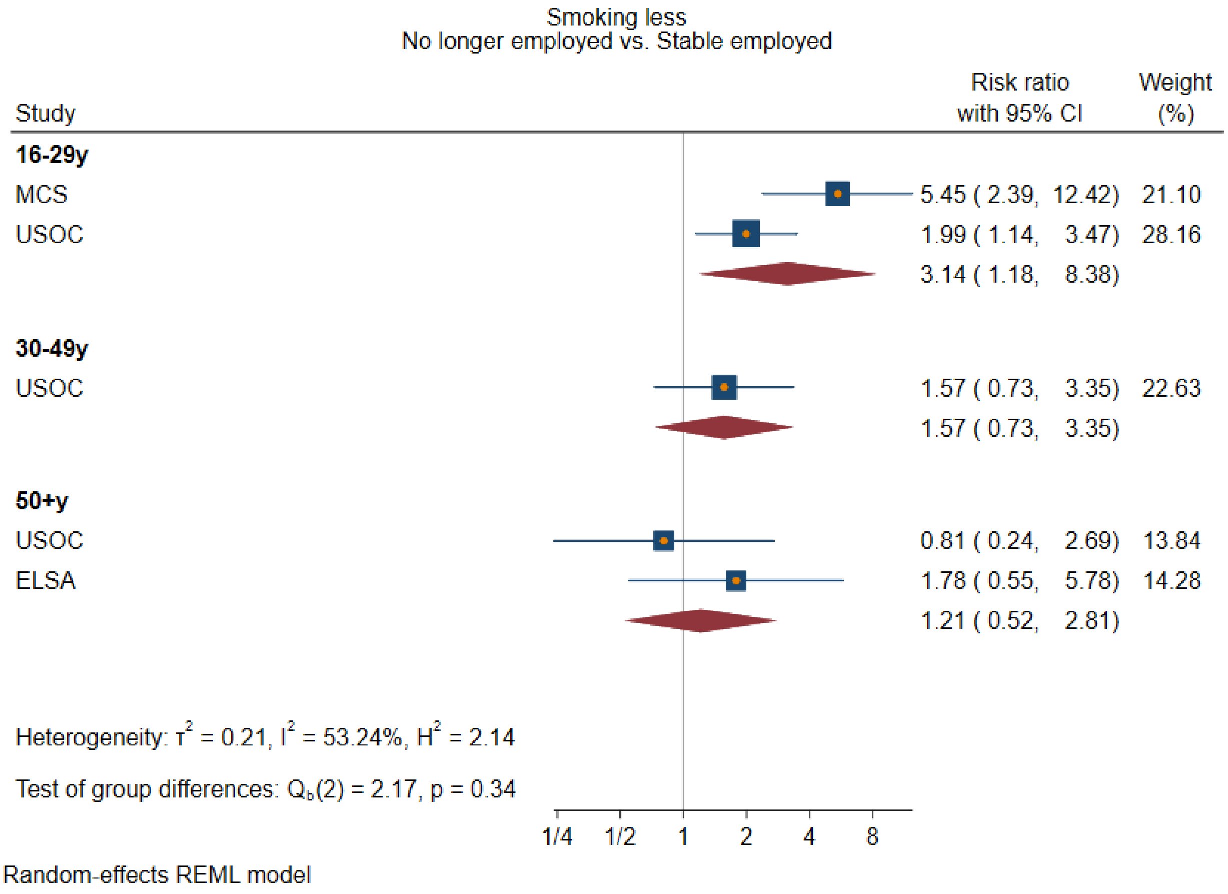

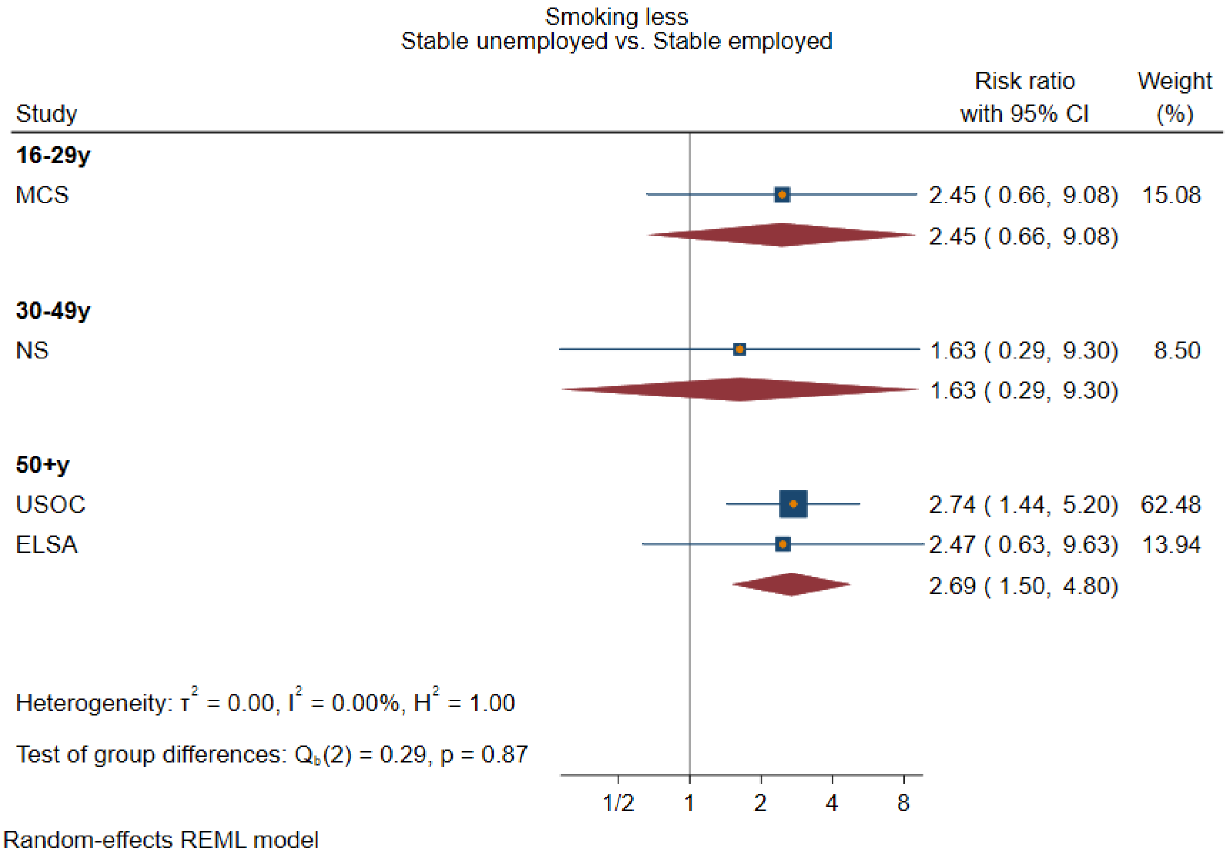
Smoking less
Stable unemployed vs. Stable employed
Study
Risk ratio with 95% CI
Weight (%)
16-29y
MCS
2.45 (0.66, 9.08) 15.08
2.45 (0.66, 9.08)
30-49y
NS
1.63 (0.29, 9.30) 8.50
1.63 (0.29, 9.30)
50+y
USOC
2.74 (1.44, 5.20) 62.48
ELSA
2.47 (0.63, 9.63) 13.94
2.69 (1.50, 4.80)
Heterogeneity: τ² = 0.00, I² = 0.00%, H² = 1.00
Test of group differences: Q_b(2) = 0.29, p = 0.87
1/2
1
2
4
8
Random-effects REML model

Figure set 13: Current vaper

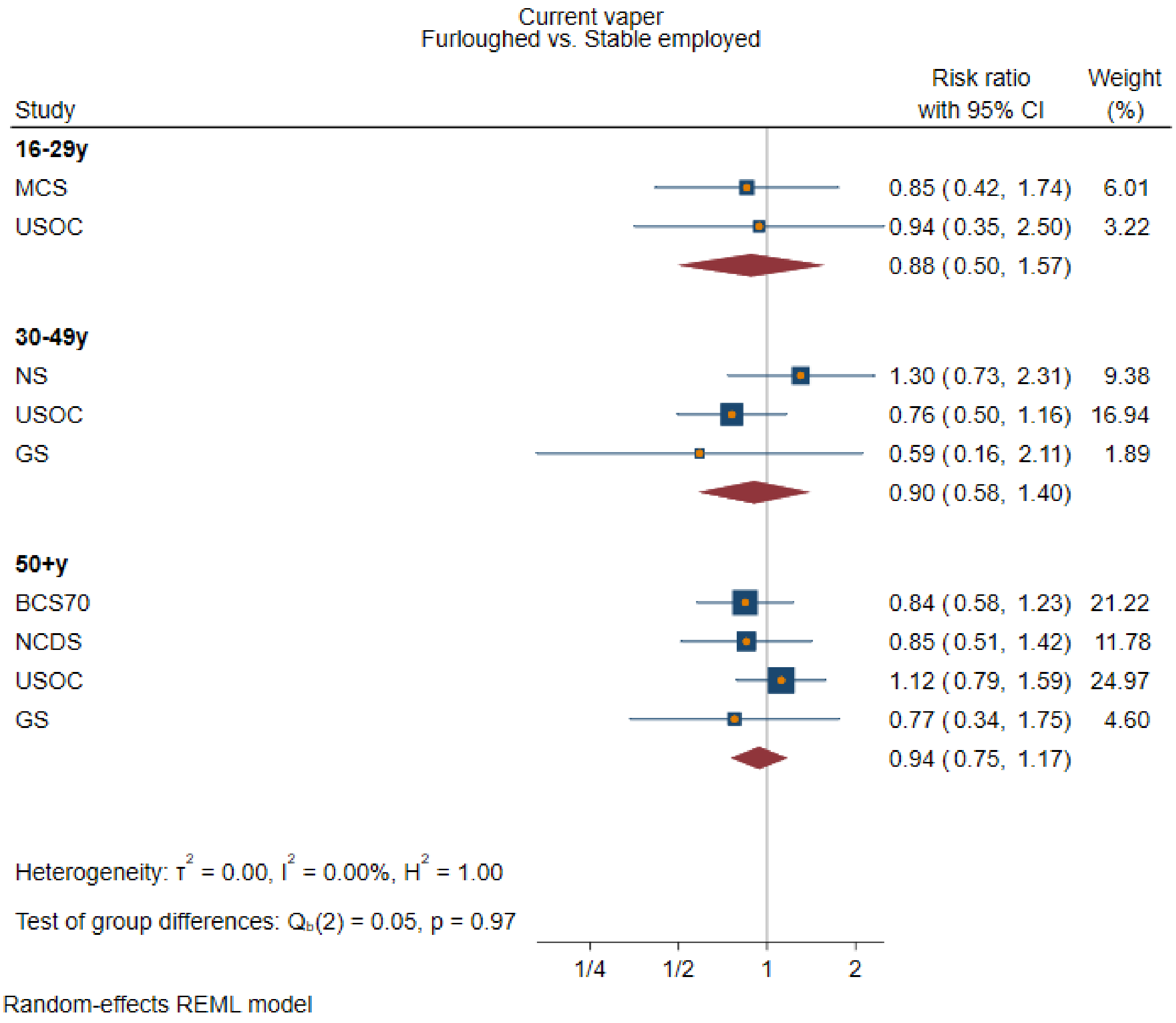

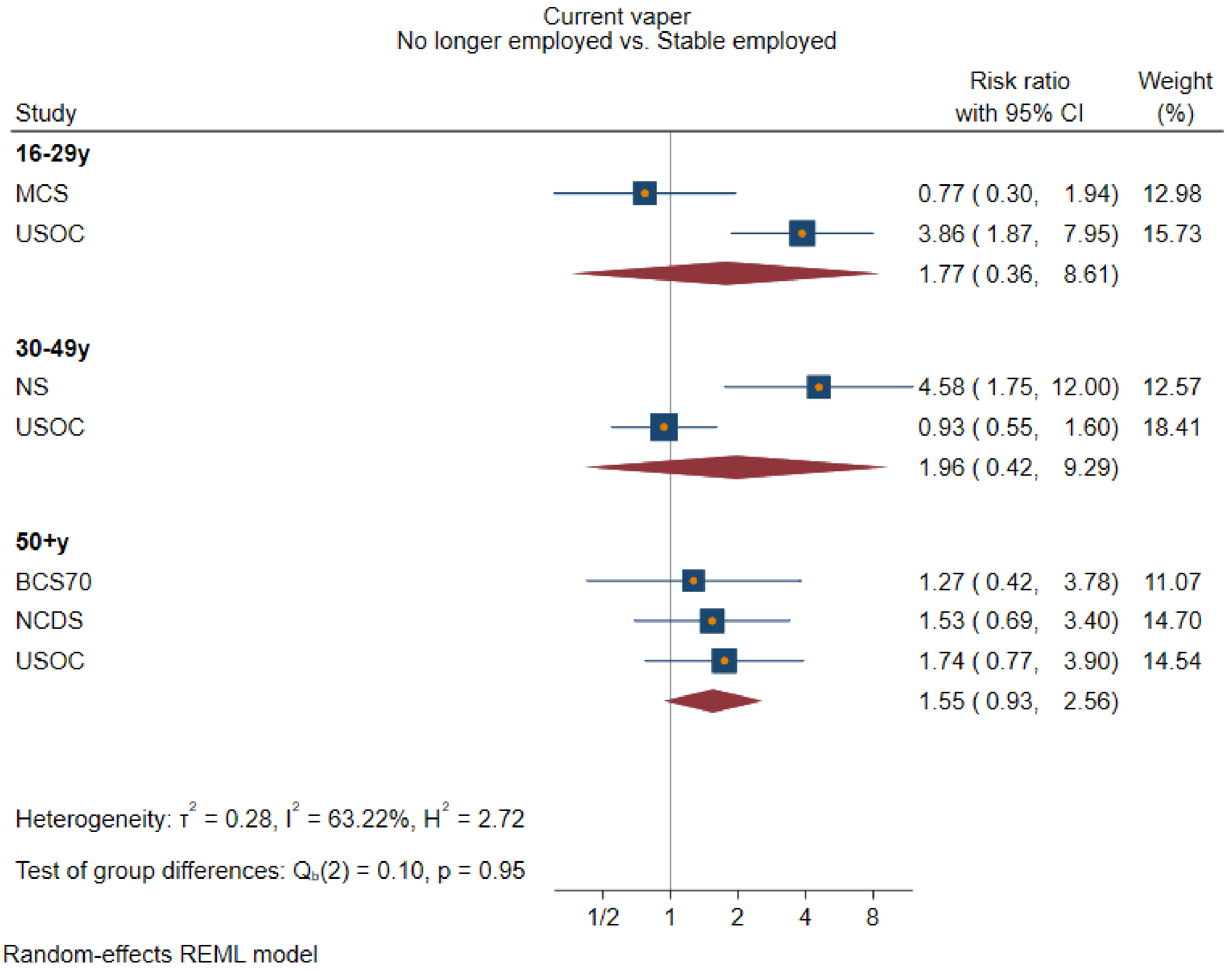

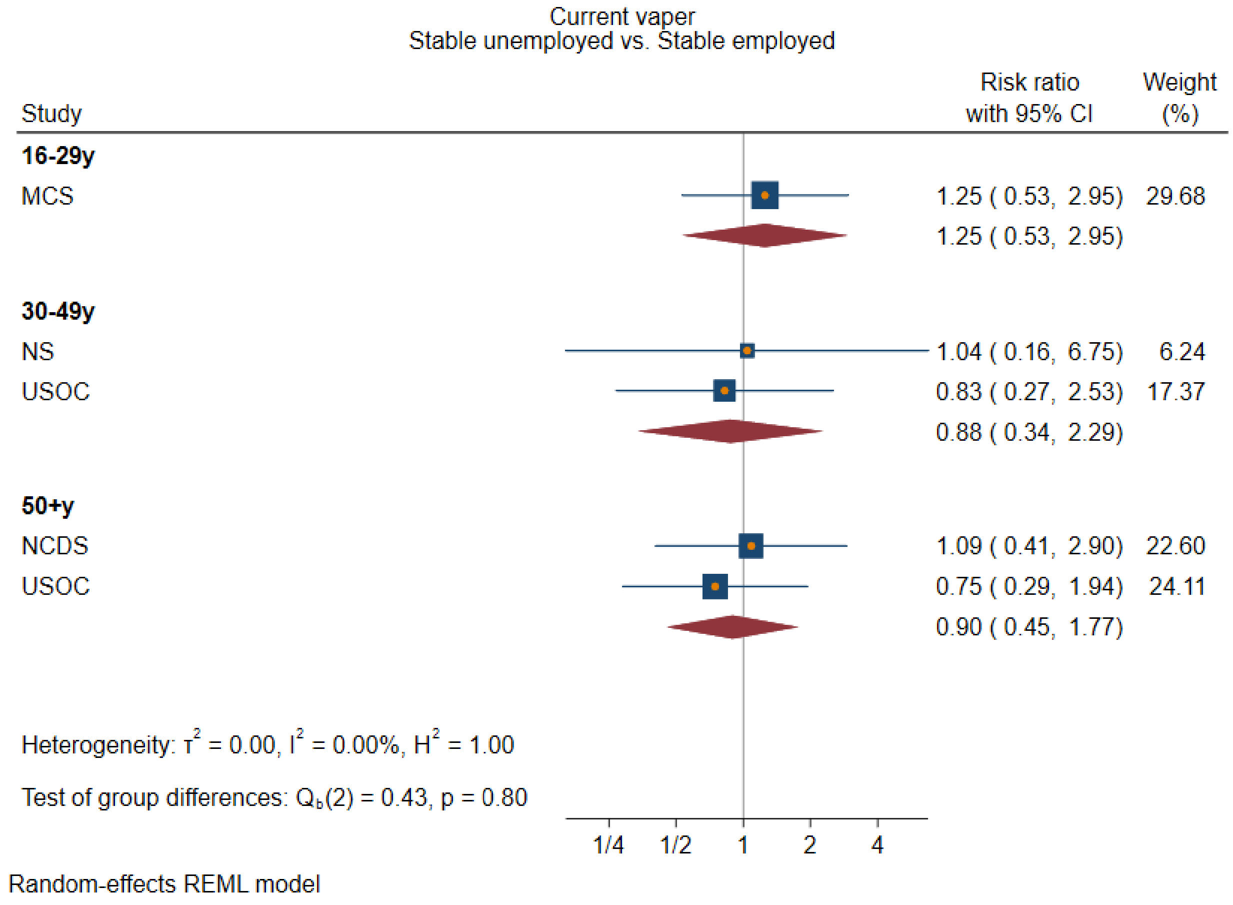
Current vaper
Stable unemployed vs. Stable employed
Study
Risk ratio with 95% CI
Weight (%)
16-29y
MCS 1.25 (0.53, 2.95) 29.68
1.25 (0.53, 2.95)
30-49y
NS 1.04 (0.16, 6.75) 6.24
USOC 0.83 (0.27, 2.53) 17.37
0.88 (0.34, 2.29)
50+y
NCDS 1.09 (0.41, 2.90) 22.60
USOC 0.75 (0.29, 1.94) 24.11
0.90 (0.45, 1.77)
Heterogeneity: τ^2 = 0.00, I^2 = 0.00%, H^2 = 1.00
Test of group differences: $Q_b(2)$ = 0.43, p = 0.80
1/4
1/2
1
2
4
Random-effects REML model

Figure set 14: Vaping more

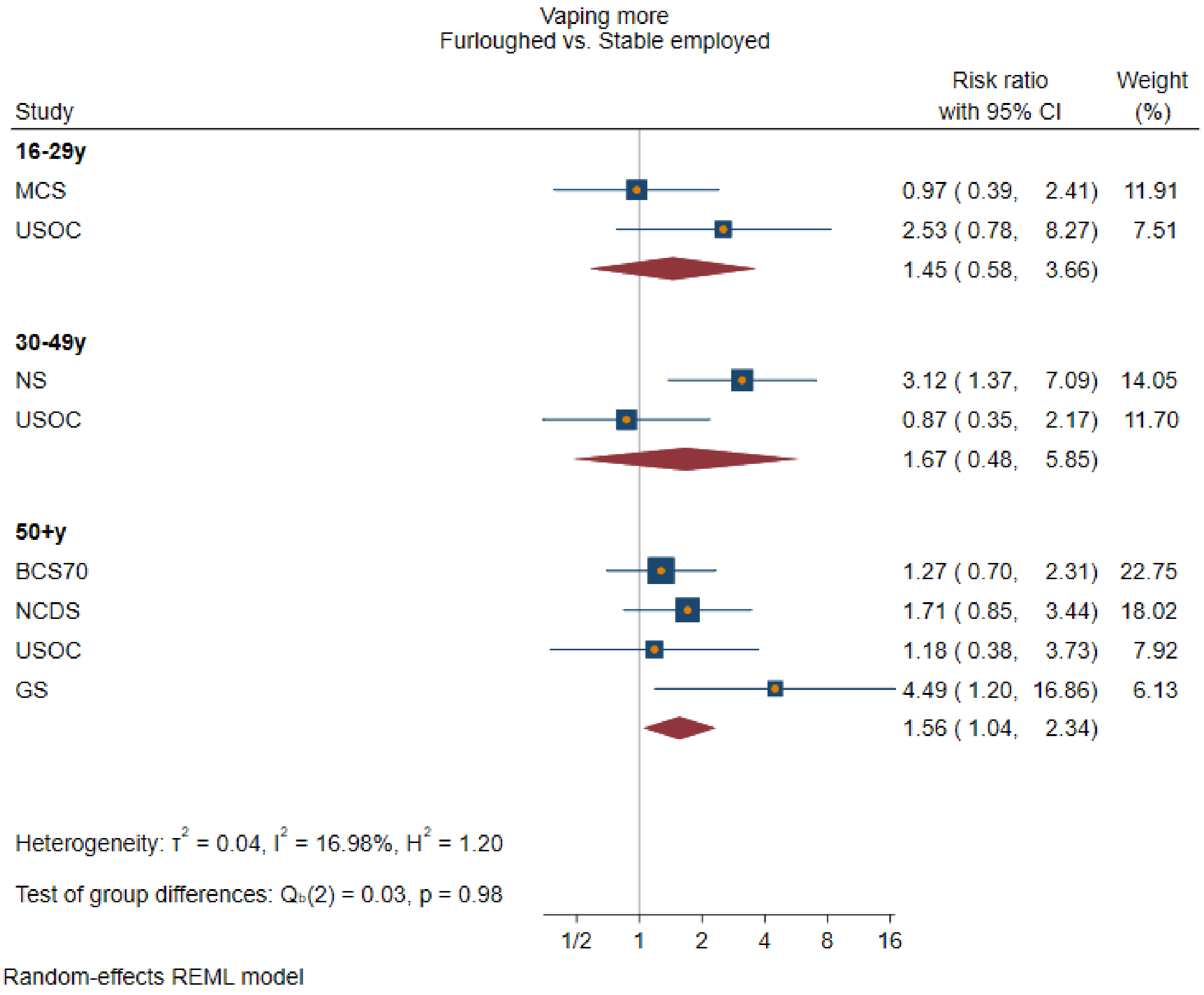

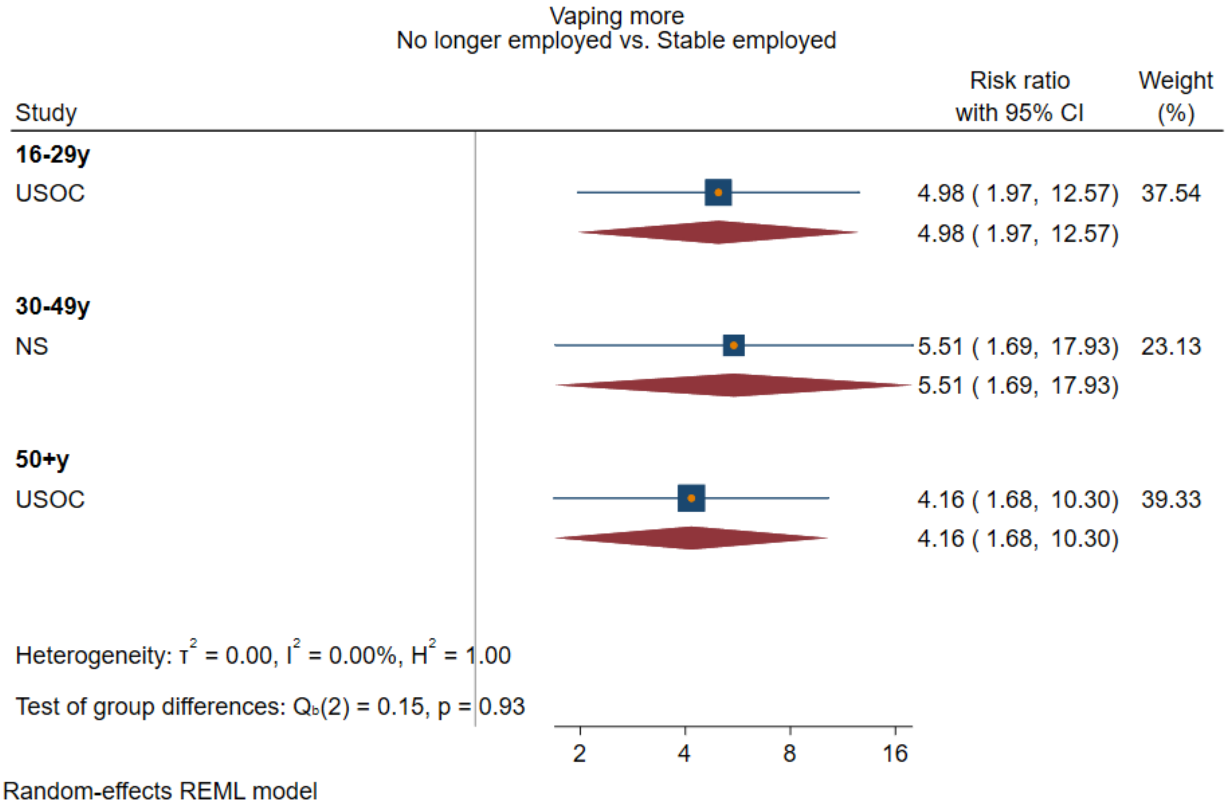

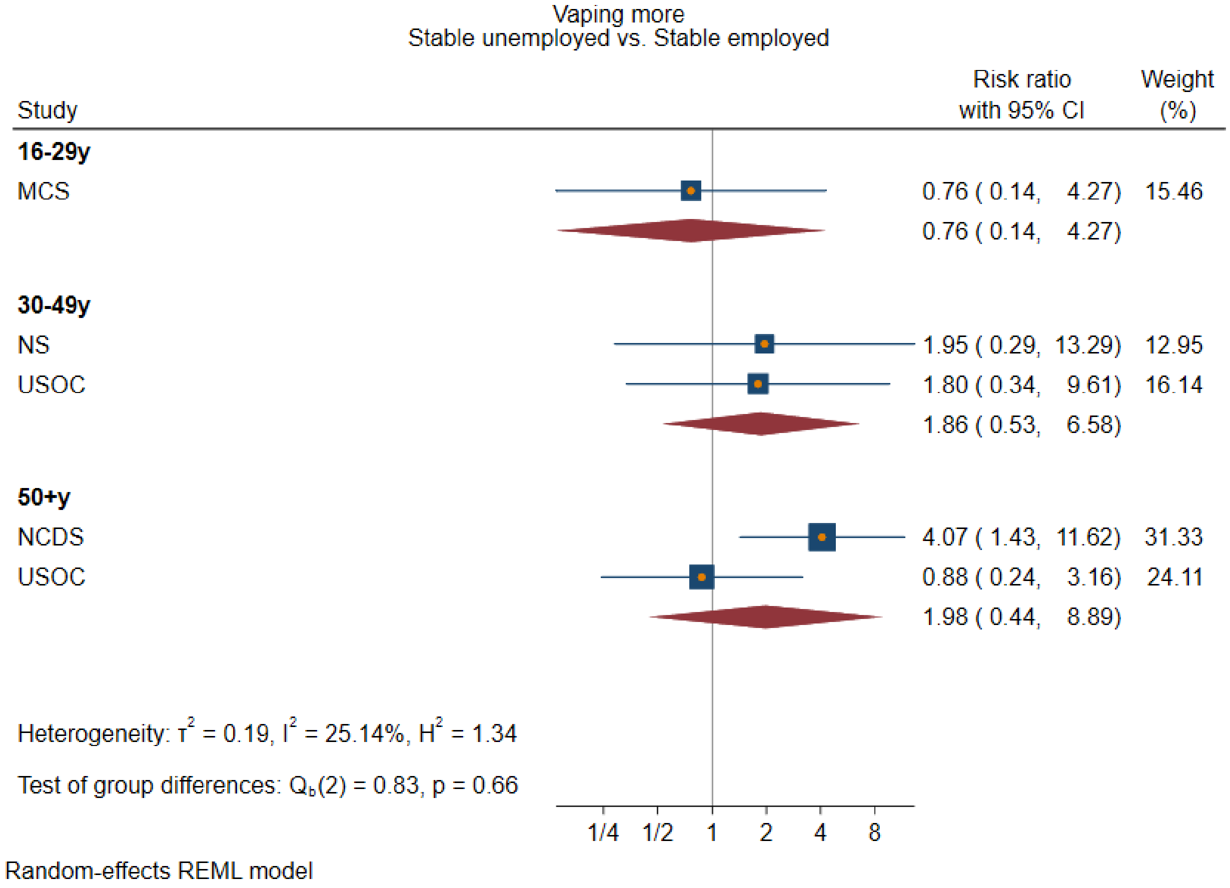
Vaping more
Stable unemployed vs. Stable employed
Study
Risk ratio with 95% CI
Weight (%)
16-29y
MCS 0.76 (0.14, 4.27) 15.46
0.76 (0.14, 4.27)
30-49y
NS 1.95 (0.29, 13.29) 12.95
USOC 1.80 (0.34, 9.61) 16.14
1.86 (0.53, 6.58)
50+y
NCDS 4.07 (1.43, 11.62) 31.33
USOC 0.88 (0.24, 3.16) 24.11
1.98 (0.44, 8.89)
Heterogeneity: τ^2 = 0.19, I^2 = 25.14%, H^2 = 1.34
Test of group differences: $Q_b(2)$ = 0.83, p = 0.66
1/4 1/2 1 2 4 8
Random-effects REML model

Figure set 15: Vaping less

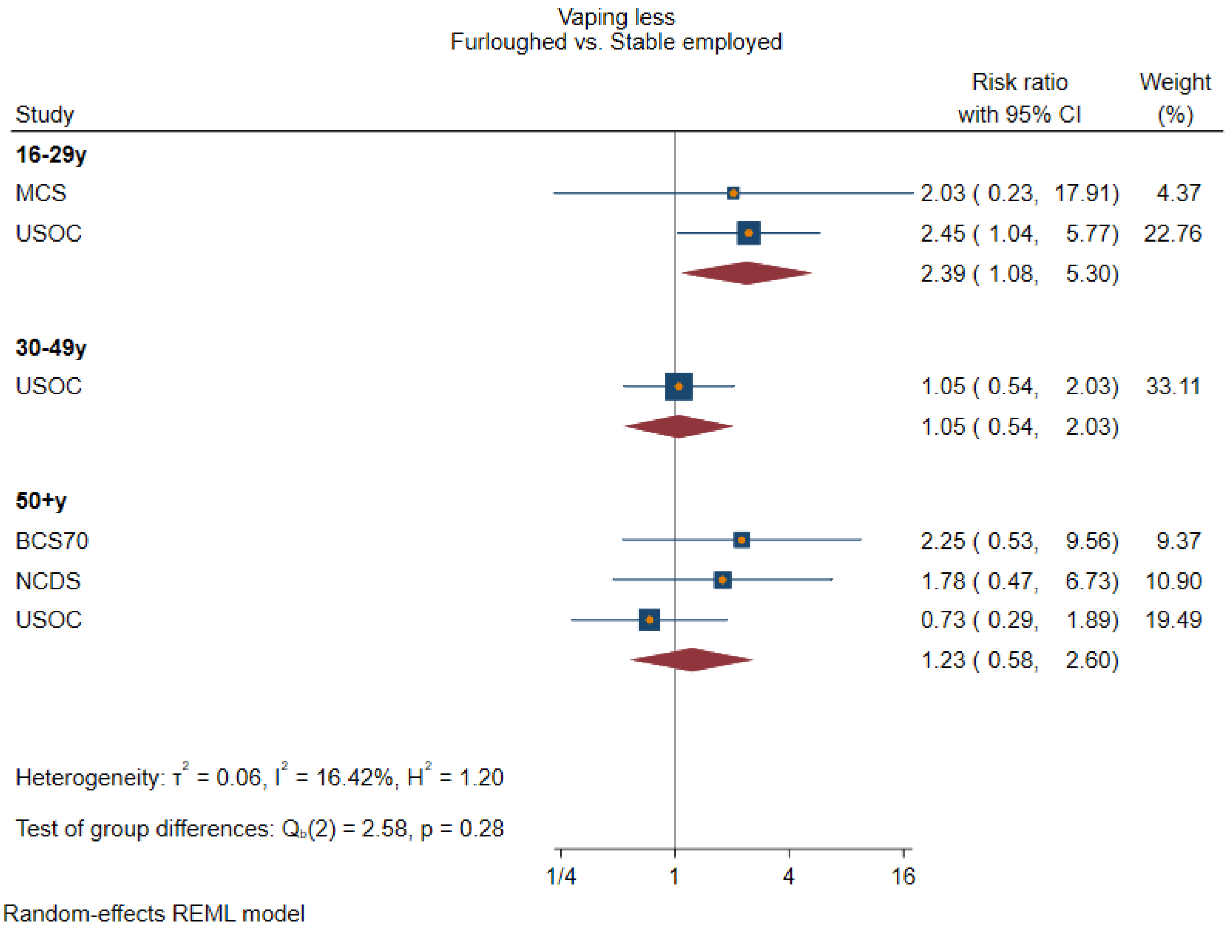

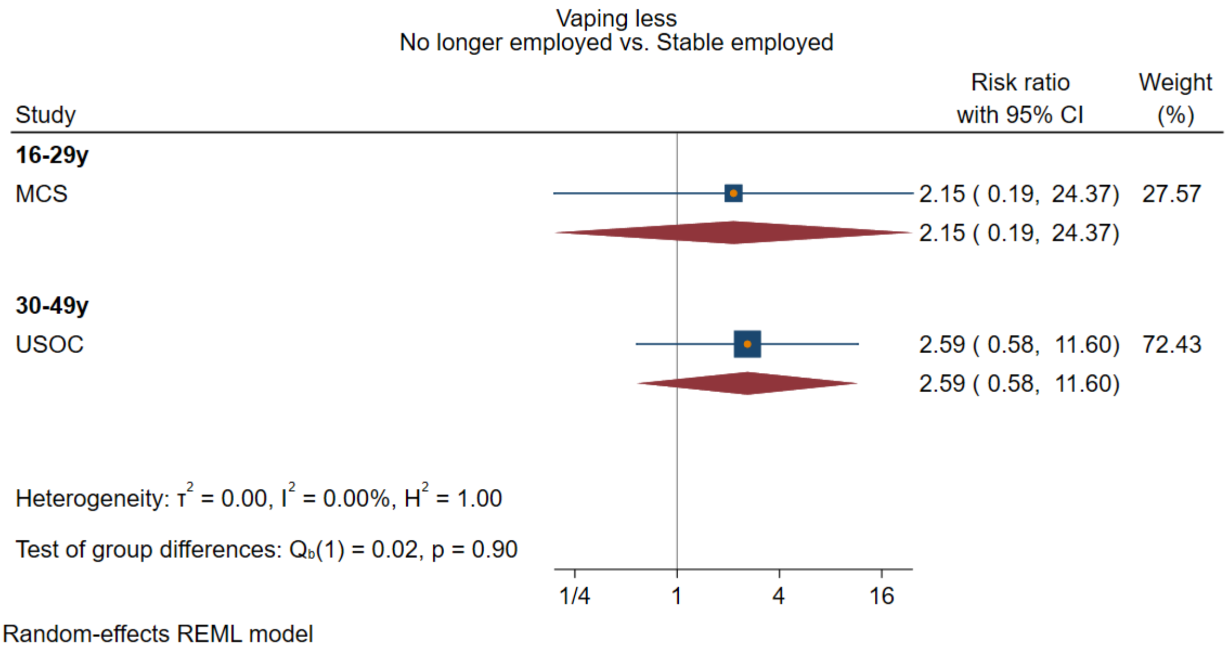

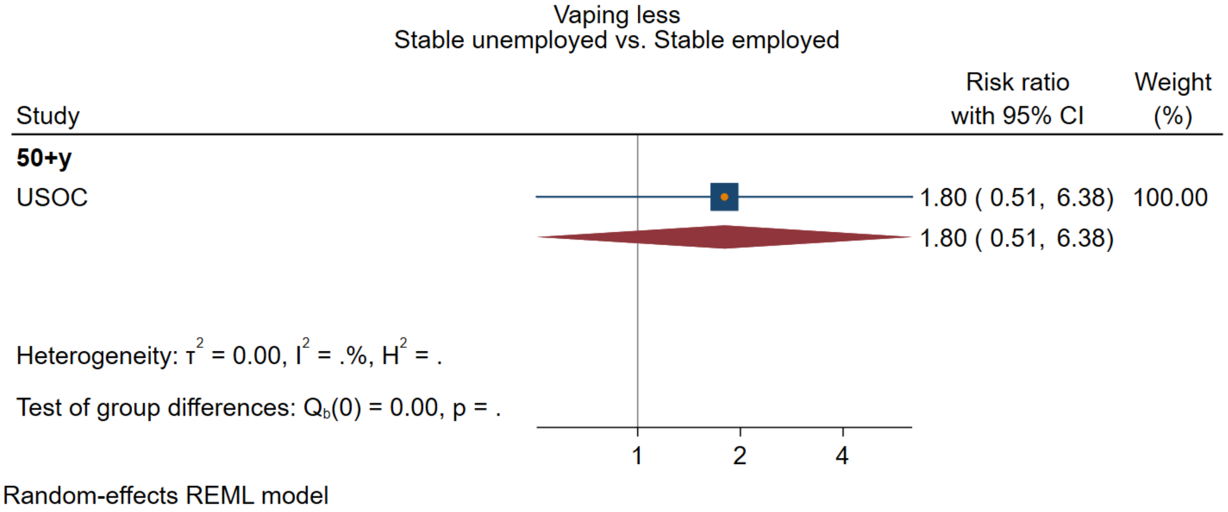

Figure set 16: Currently drinks 4+ days/week or 5+ drinks/occasion

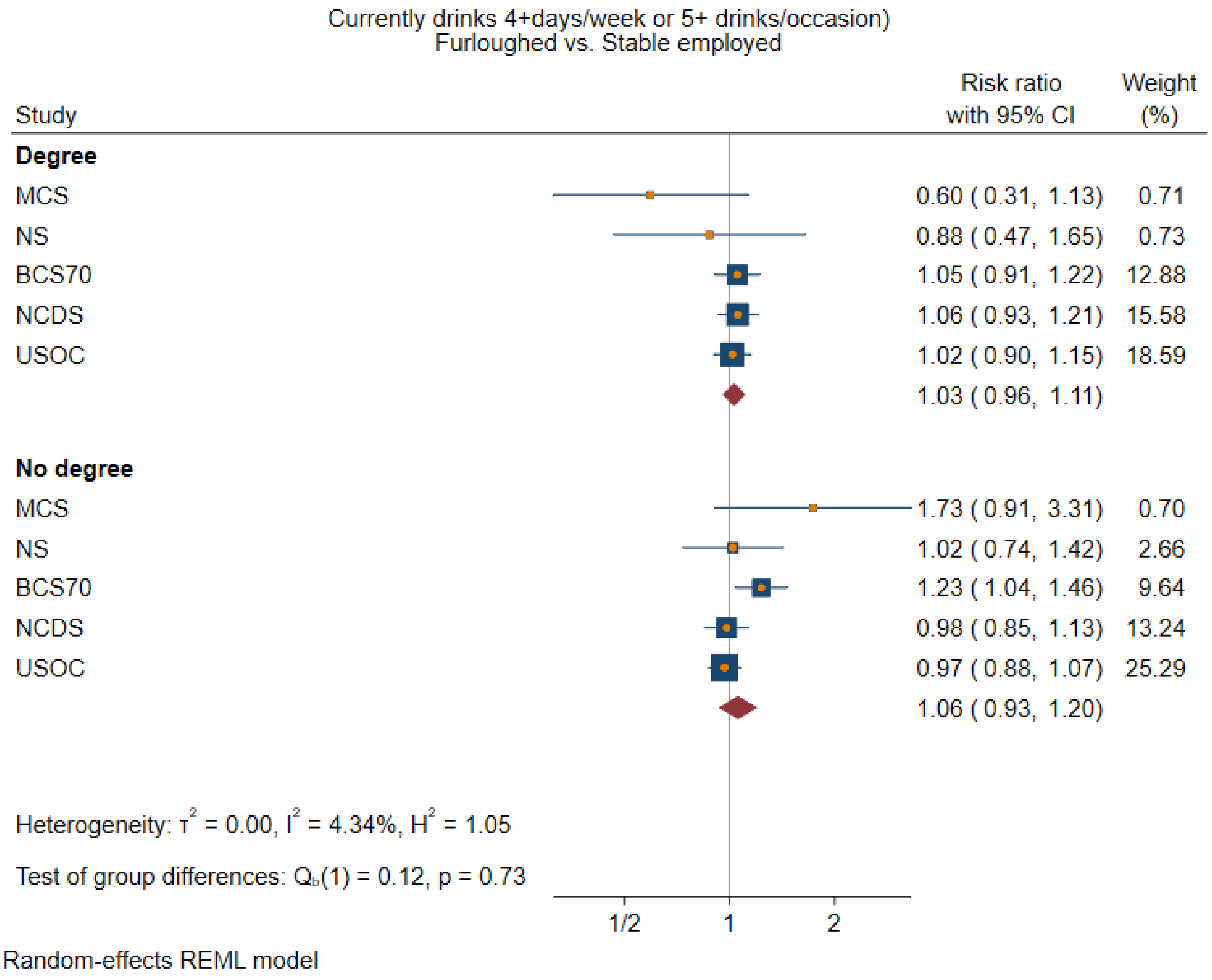

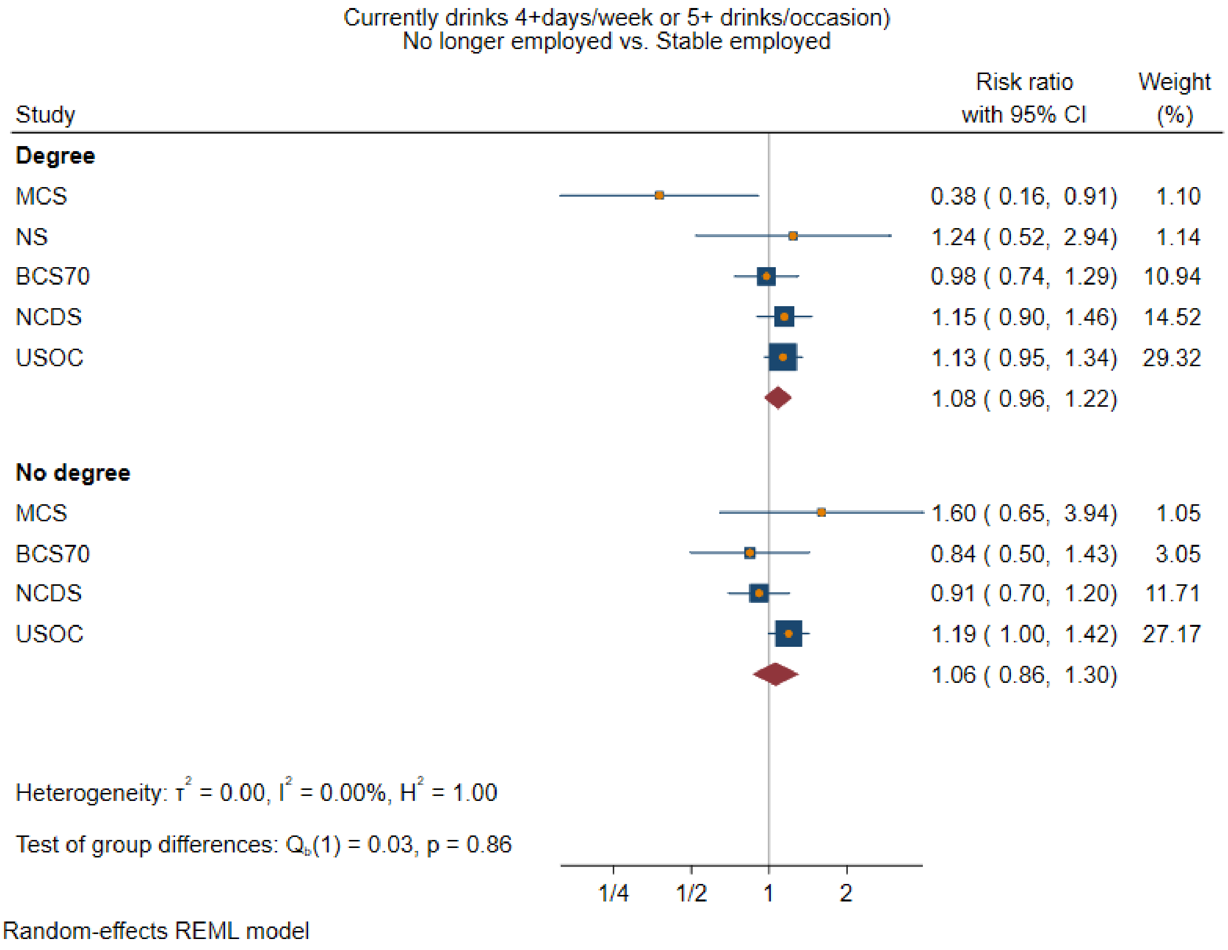

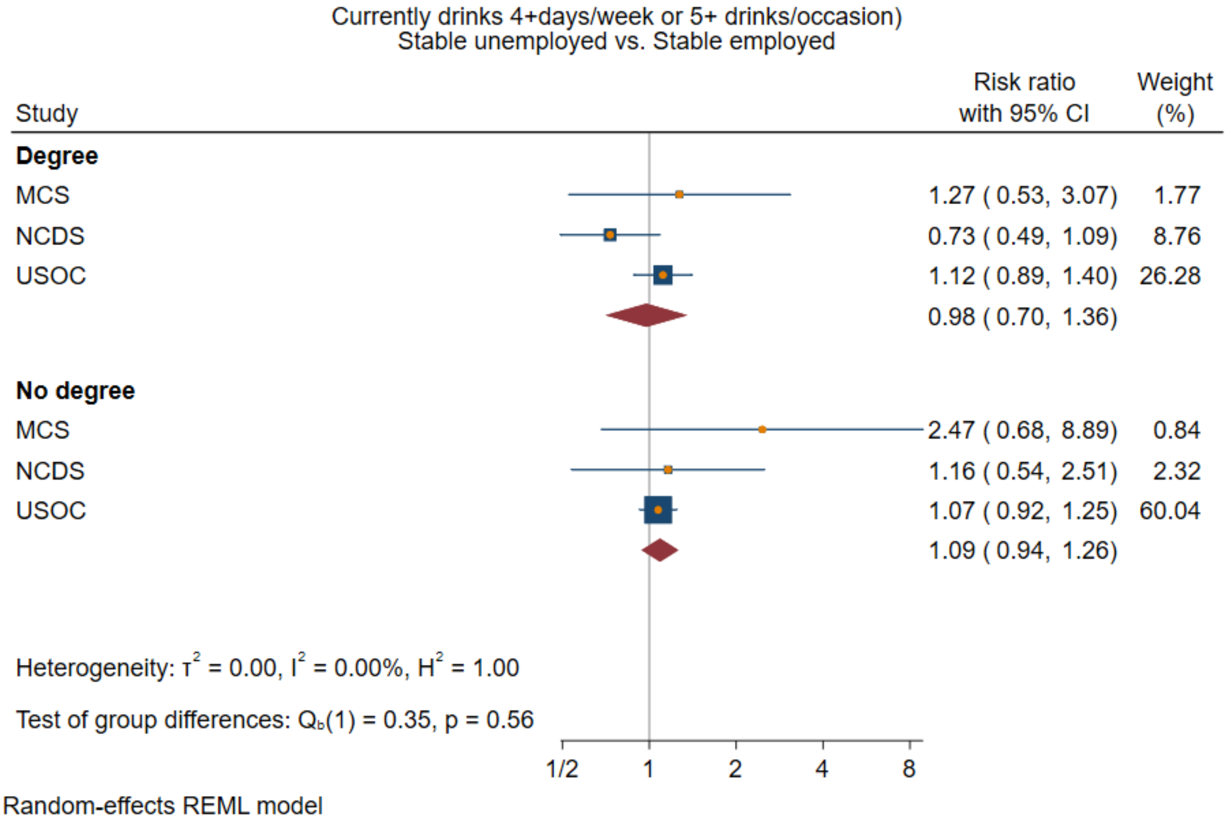
Currently drinks 4+days/week or 5+ drinks/occasion)
Stable unemployed vs. Stable employed
Study
Risk ratio with 95% CI
Weight (%)
Degree
MCS 1.27 (0.53, 3.07) 1.77
NCDS 0.73 (0.49, 1.09) 8.76
USOC 1.12 (0.89, 1.40) 26.28
0.98 (0.70, 1.36)
No degree
MCS 2.47 (0.68, 8.89) 0.84
NCDS 1.16 (0.54, 2.51) 2.32
USOC 1.07 (0.92, 1.25) 60.04
1.09 (0.94, 1.26)
Heterogeneity: τ² = 0.00, I² = 0.00%, H² = 1.00
Test of group differences: Qb(1) = 0.35, p = 0.56
1/2
1
2
4
8
Random-effects REML model

Figure set 17: Increased alcohol consumption

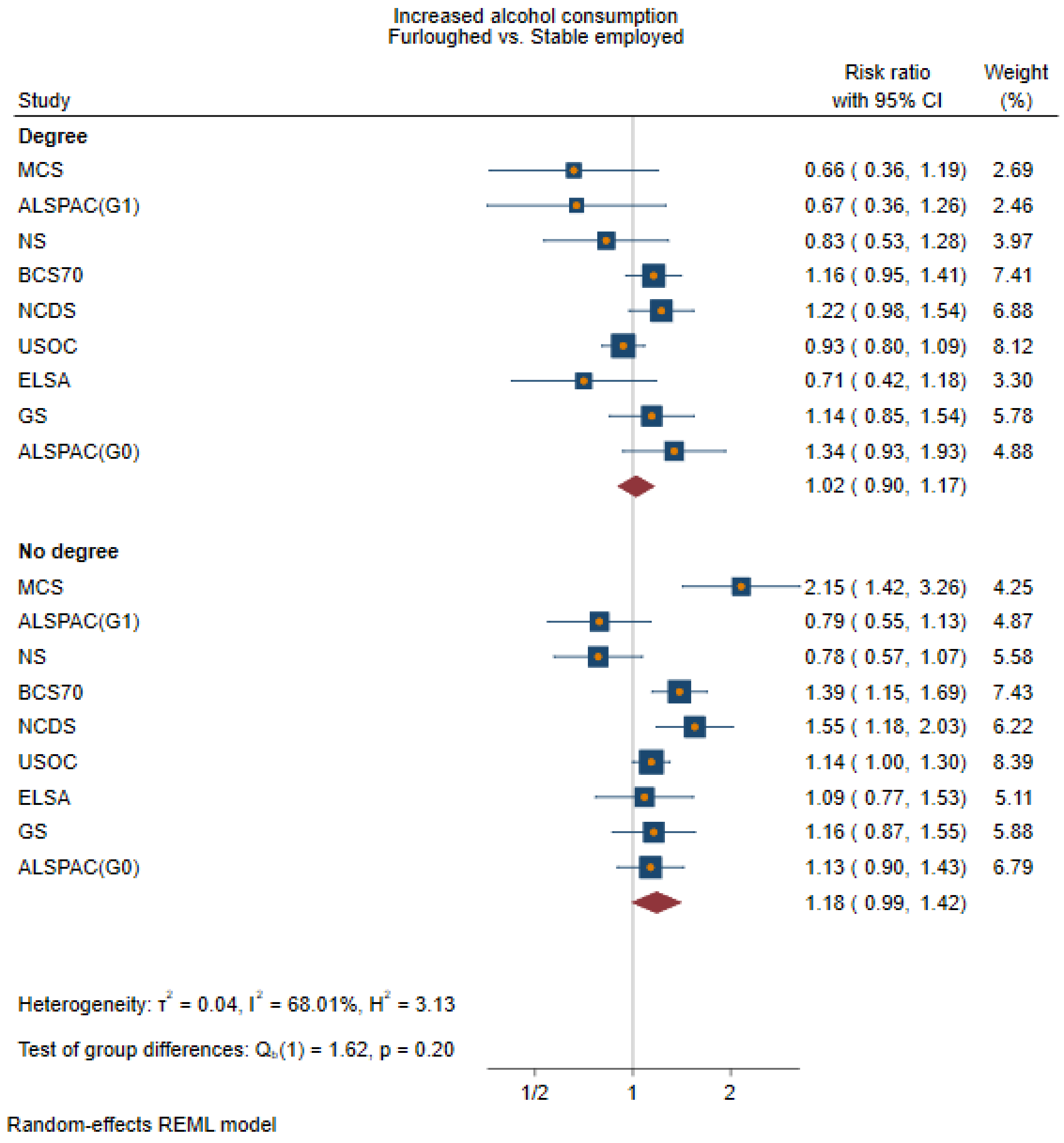

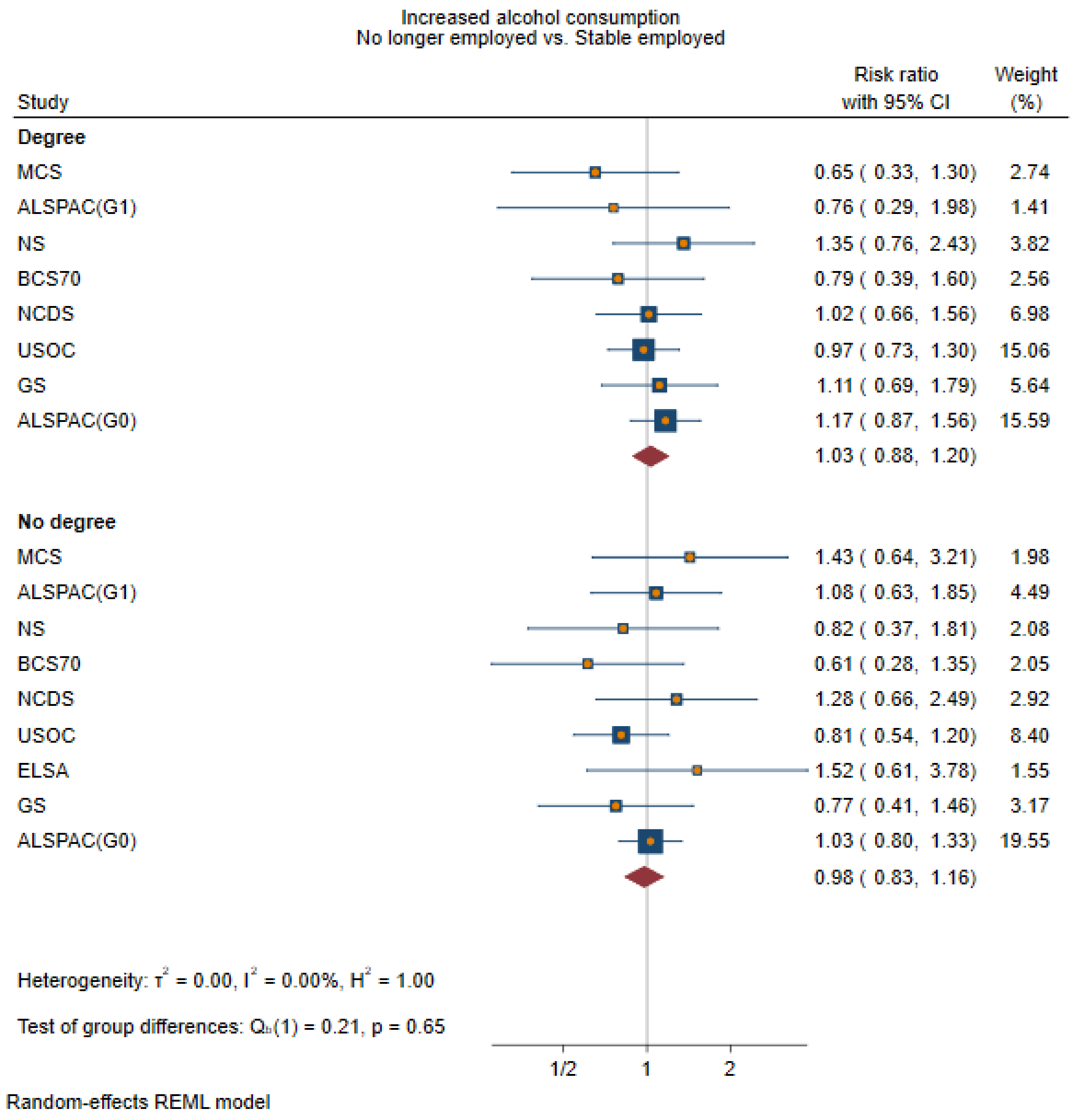
Increased alcohol consumption
No longer employed vs. Stable employed
Study
Risk ratio with 95% CI
Weight (%)
Degree
MCS 0.65 (0.33, 1.30) 2.74
ALSPAC(G1) 0.76 (0.29, 1.98) 1.41
NS 1.35 (0.76, 2.43) 3.82
BCS70 0.79 (0.39, 1.60) 2.56
NCDS 1.02 (0.66, 1.56) 6.98
USOC 0.97 (0.73, 1.30) 15.06
GS 1.11 (0.69, 1.79) 5.64
ALSPAC(G0) 1.17 (0.87, 1.56) 15.59
1.03 (0.88, 1.20)
No degree
MCS 1.43 (0.64, 3.21) 1.98
ALSPAC(G1) 1.08 (0.63, 1.85) 4.49
NS 0.82 (0.37, 1.81) 2.08
BCS70 0.61 (0.28, 1.35) 2.05
NCDS 1.28 (0.66, 2.49) 2.92
USOC 0.81 (0.54, 1.20) 8.40
ELSA 1.52 (0.61, 3.78) 1.55
GS 0.77 (0.41, 1.46) 3.17
ALSPAC(G0) 1.03 (0.80, 1.33) 19.55
0.98 (0.83, 1.16)
Heterogeneity: τ^2 = 0.00, I^2 = 0.00%, H^2 = 1.00
Test of group differences: $Q_b(1)$ = 0.21, p = 0.65
1/2
1
2
Random-effects REML model

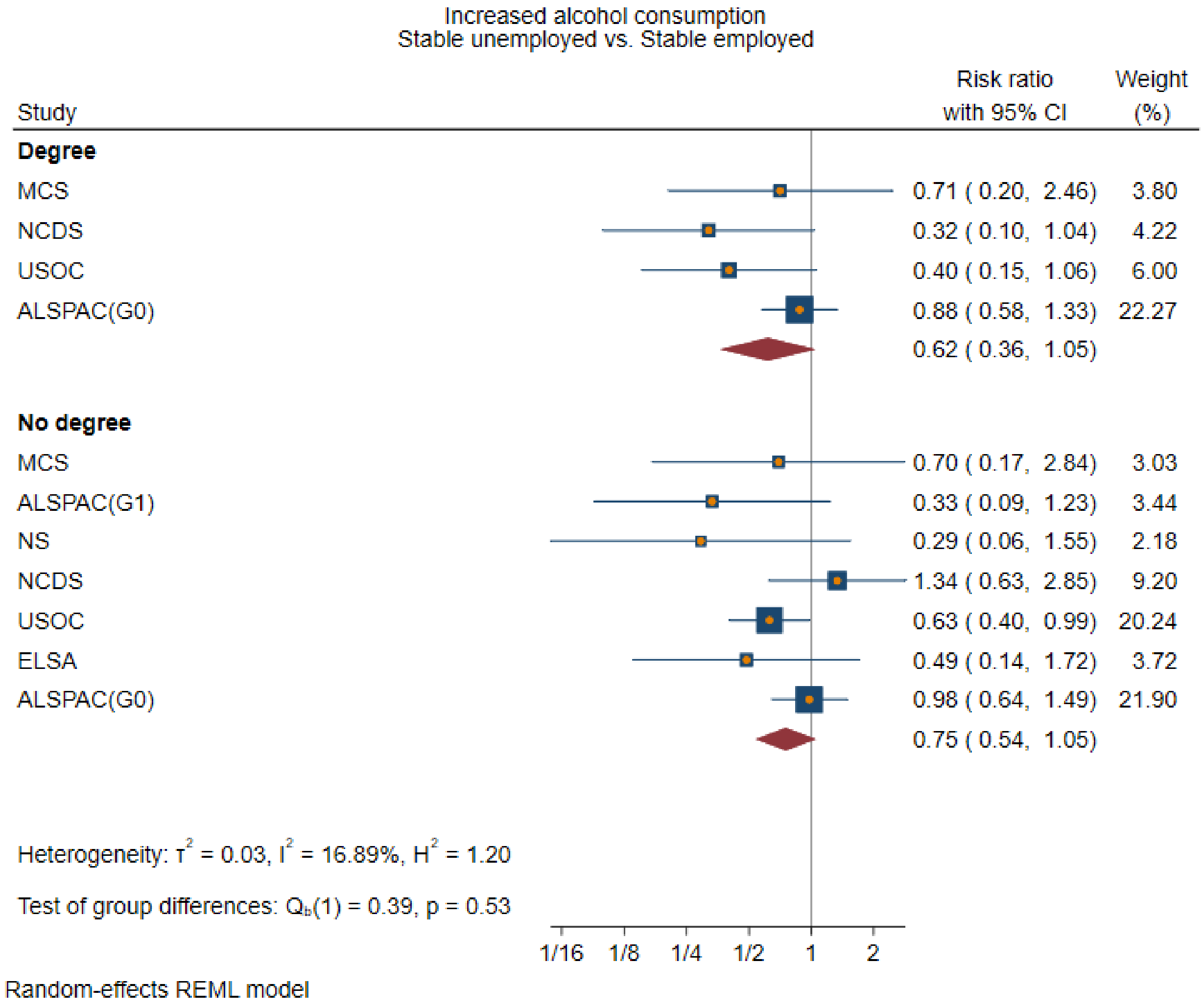
Increased alcohol consumption
Stable unemployed vs. Stable employed
Study
Risk ratio with 95% CI
Weight (%)
Degree
MCS 0.71 (0.20, 2.46) 3.80
NCDS 0.32 (0.10, 1.04) 4.22
USOC 0.40 (0.15, 1.06) 6.00
ALSPAC(G0) 0.88 (0.58, 1.33) 22.27
0.62 (0.36, 1.05)
No degree
MCS 0.70 (0.17, 2.84) 3.03
ALSPAC(G1) 0.33 (0.09, 1.23) 3.44
NS 0.29 (0.06, 1.55) 2.18
NCDS 1.34 (0.63, 2.85) 9.20
USOC 0.63 (0.40, 0.99) 20.24
ELSA 0.49 (0.14, 1.72) 3.72
ALSPAC(G0) 0.98 (0.64, 1.49) 21.90
0.75 (0.54, 1.05)
Heterogeneity: τ^2 = 0.03, I^2 = 16.89%, H^2 = 1.20
Test of group differences: $Q_b(1)$ = 0.39, p = 0.53
1/16
1/8
1/4
1/2
1
2
Random-effects REML model

Figure set 18: Reduced alcohol consumption

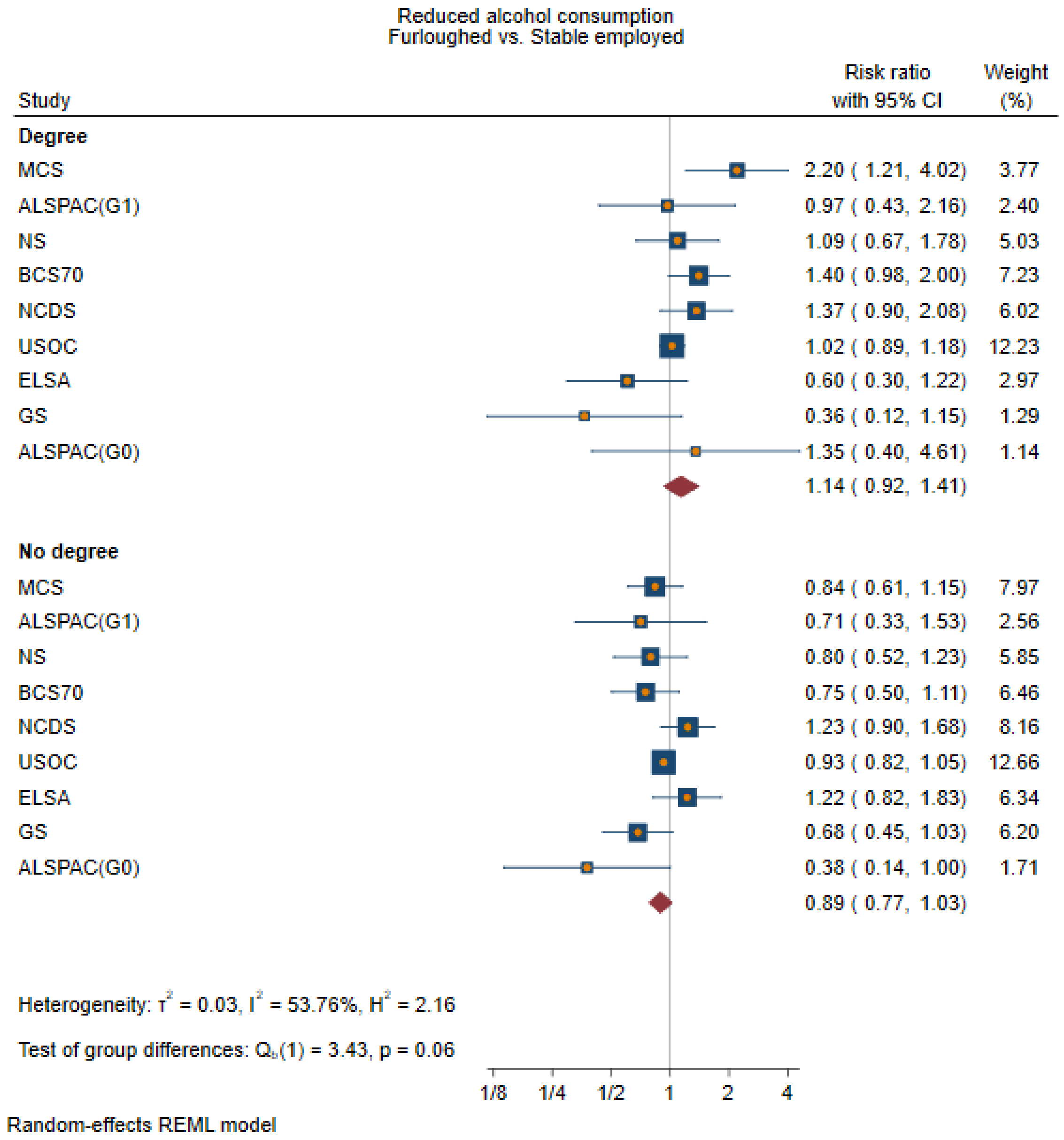

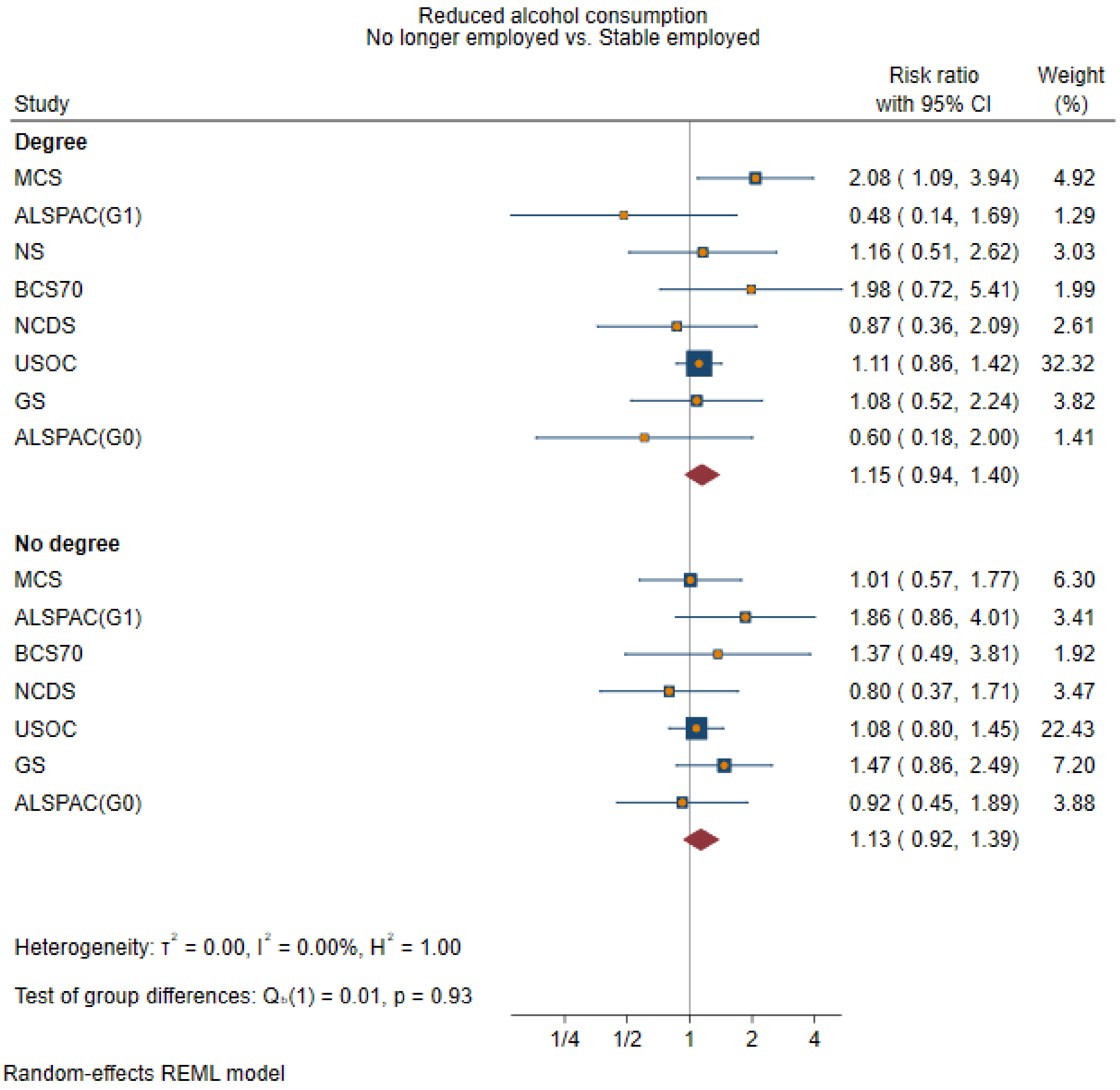

Reduced alcohol consumption
No longer employed vs. Stable employed
Study
Risk ratio with 95% CI
Weight (%)
Degree
MCS 2.08 (1.09, 3.94) 4.92
ALSPAC(G1) 0.48 (0.14, 1.69) 1.29
NS 1.16 (0.51, 2.62) 3.03
BCS70 1.98 (0.72, 5.41) 1.99
NCDS 0.87 (0.36, 2.09) 2.61
USOC 1.11 (0.86, 1.42) 32.32
GS 1.08 (0.52, 2.24) 3.82
ALSPAC(G0) 0.60 (0.18, 2.00) 1.41
1.15 (0.94, 1.40)
No degree
MCS 1.01 (0.57, 1.77) 6.30
ALSPAC(G1) 1.86 (0.86, 4.01) 3.41
BCS70 1.37 (0.49, 3.81) 1.92
NCDS 0.80 (0.37, 1.71) 3.47
USOC 1.08 (0.80, 1.45) 22.43
GS 1.47 (0.86, 2.49) 7.20
ALSPAC(G0) 0.92 (0.45, 1.89) 3.88
1.13 (0.92, 1.39)
Heterogeneity: τ² = 0.00, I² = 0.00%, H² = 1.00
Test of group differences: Qb(1) = 0.01, p = 0.93
1/4
1/2
1
2
4
Random-effects REML model

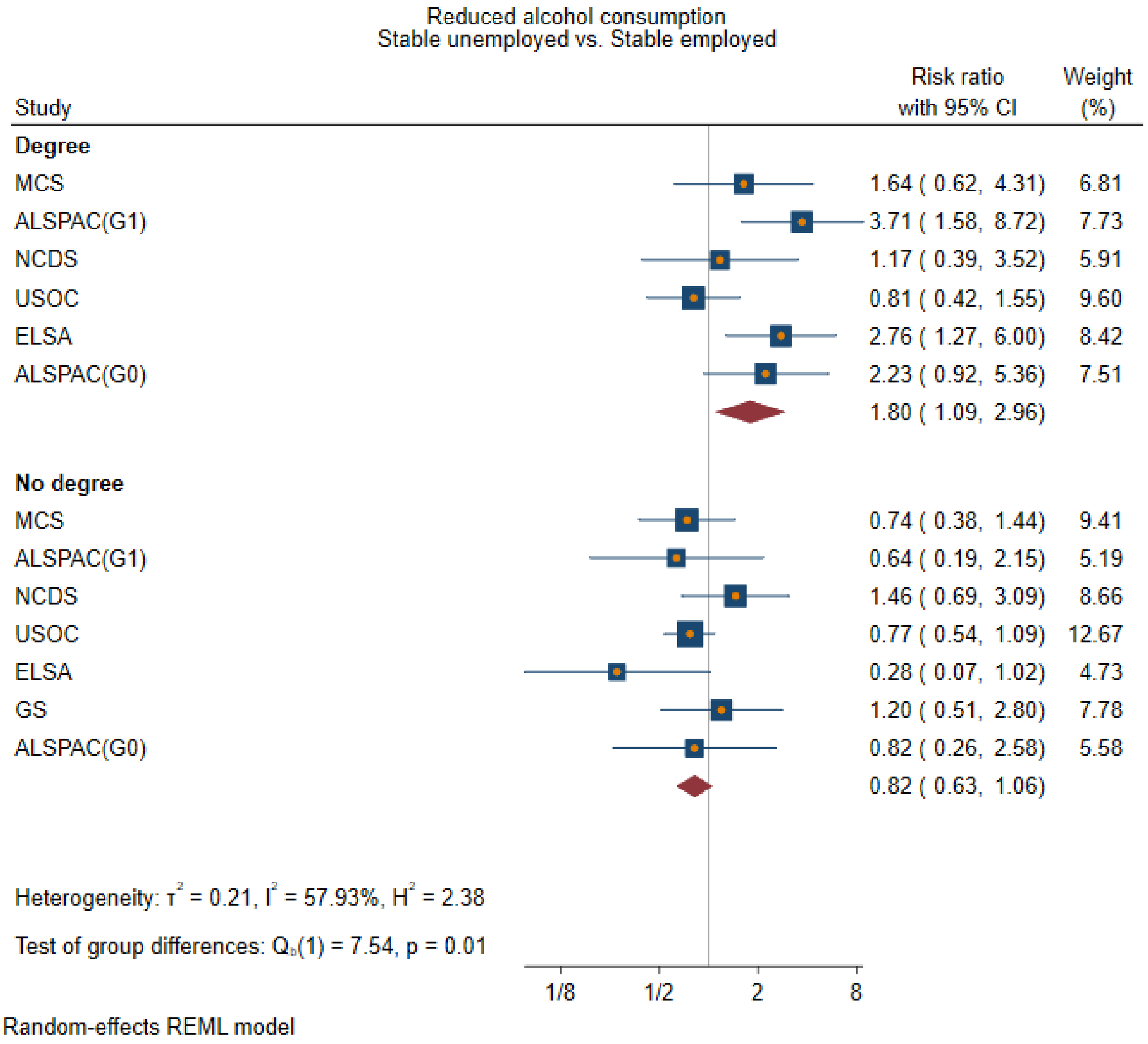

Reduced alcohol consumption
Stable unemployed vs. Stable employed
Study
Risk ratio with 95% CI
Weight (%)
Degree
MCS 1.64 (0.62, 4.31) 6.81
ALSPAC(G1) 3.71 (1.58, 8.72) 7.73
NCDS 1.17 (0.39, 3.52) 5.91
USOC 0.81 (0.42, 1.55) 9.60
ELSA 2.76 (1.27, 6.00) 8.42
ALSPAC(G0) 2.23 (0.92, 5.36) 7.51
1.80 (1.09, 2.96)
No degree
MCS 0.74 (0.38, 1.44) 9.41
ALSPAC(G1) 0.64 (0.19, 2.15) 5.19
NCDS 1.46 (0.69, 3.09) 8.66
USOC 0.77 (0.54, 1.09) 12.67
ELSA 0.28 (0.07, 1.02) 4.73
GS 1.20 (0.51, 2.80) 7.78
ALSPAC(G0) 0.82 (0.26, 2.58) 5.58
0.82 (0.63, 1.06)
Heterogeneity: τ² = 0.21, I² = 57.93%, H² = 2.38
Test of group differences: Qb(1) = 7.54, p = 0.01
1/8
1/2
2
8
Random-effects REML model

Figure set 19: Currently drinks 5+ drinks/occasions

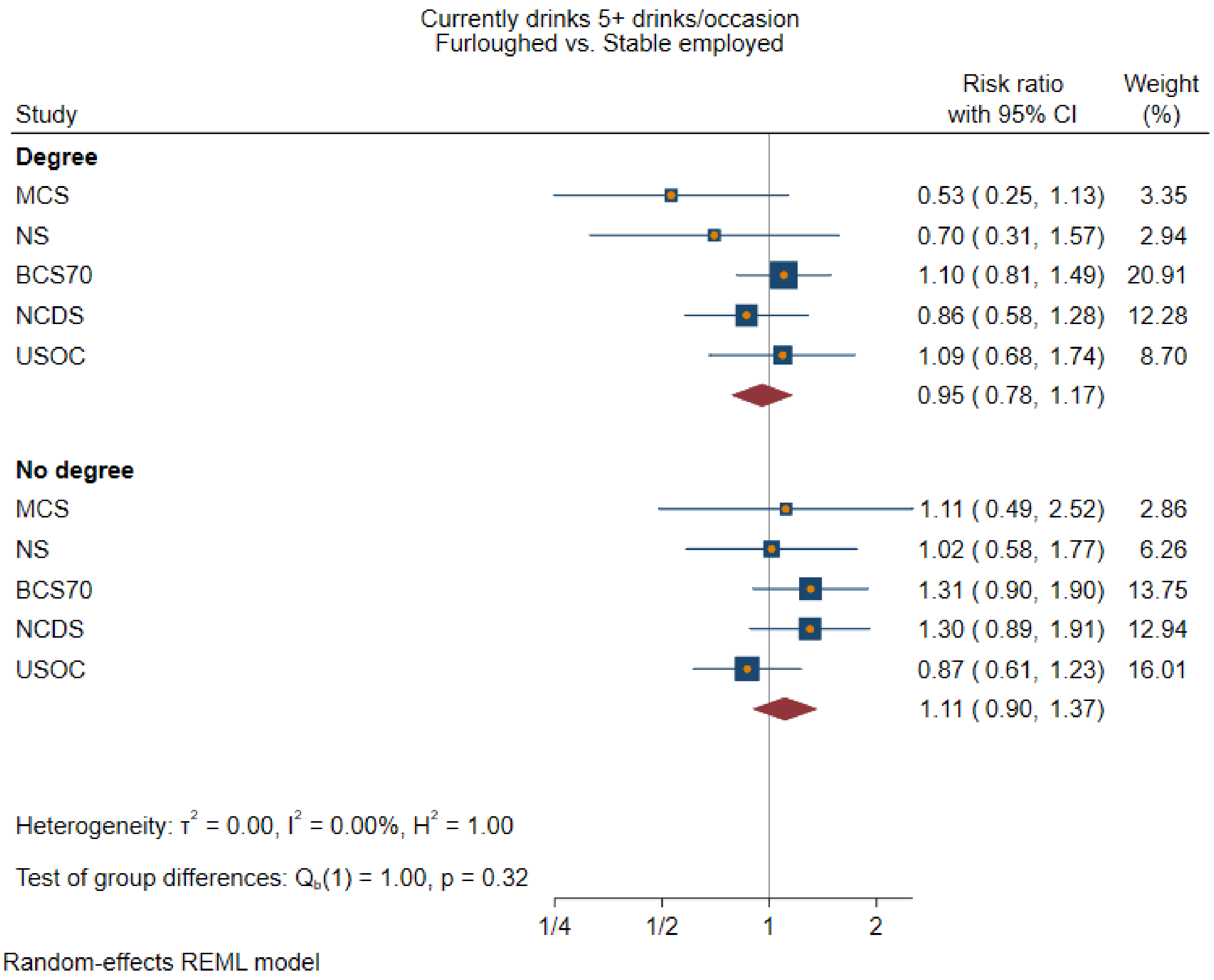

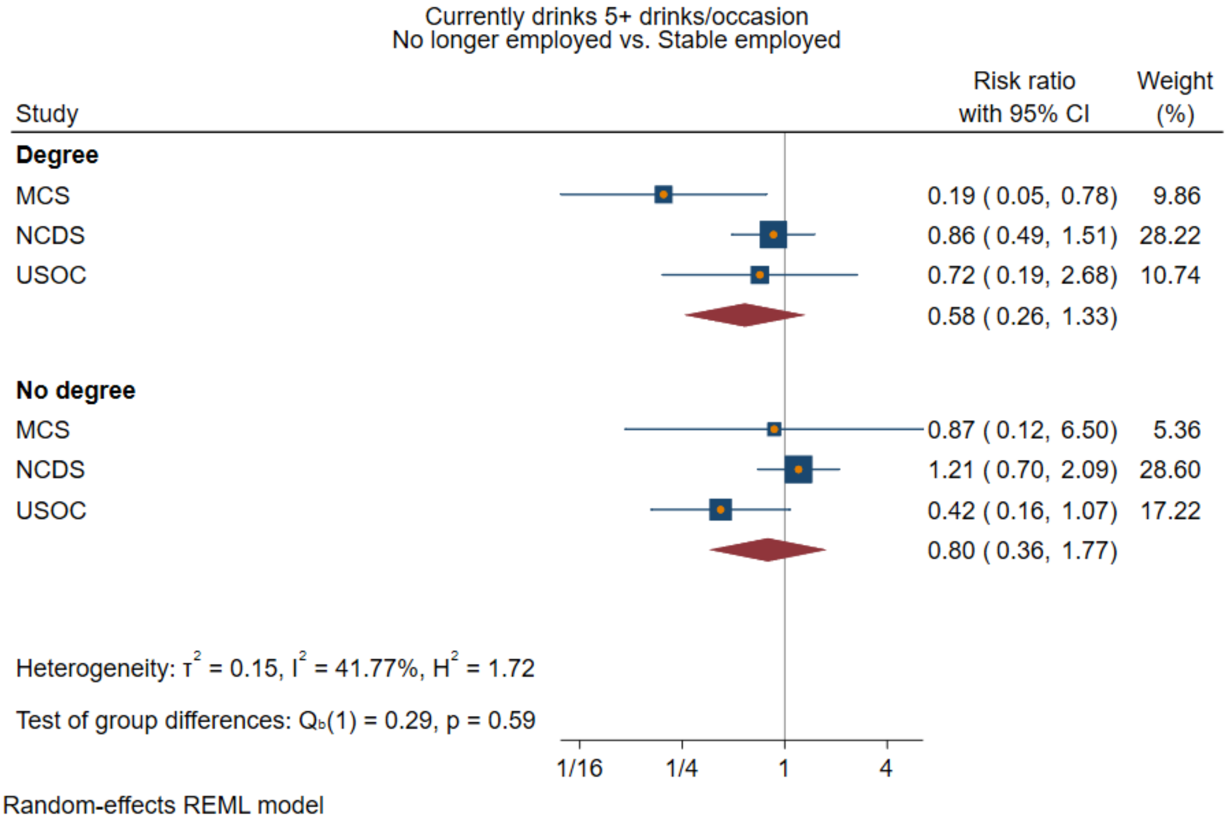

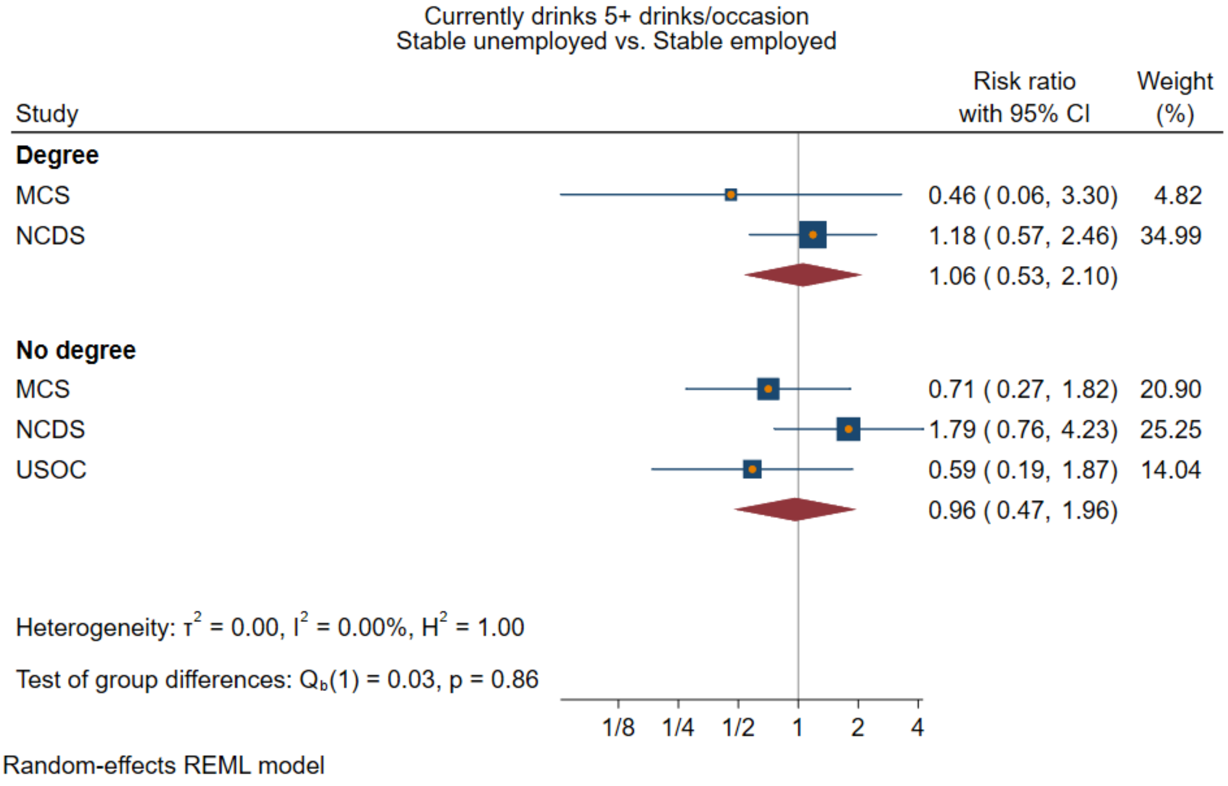
Currently drinks 5+ drinks/occasion
Stable unemployed vs. Stable employed
Study
Risk ratio with 95% CI
Weight (%)
Degree
MCS 0.46 (0.06, 3.30) 4.82
NCDS 1.18 (0.57, 2.46) 34.99
1.06 (0.53, 2.10)
No degree
MCS 0.71 (0.27, 1.82) 20.90
NCDS 1.79 (0.76, 4.23) 25.25
USOC 0.59 (0.19, 1.87) 14.04
0.96 (0.47, 1.96)
Heterogeneity: τ^2 = 0.00, I^2 = 0.00%, H^2 = 1.00
Test of group differences: $Q_b(1)$ = 0.03, p = 0.86
1/8 1/4 1/2 1 2 4
Random-effects REML model

Figure set 20: Drinks more alcohol units per occasions

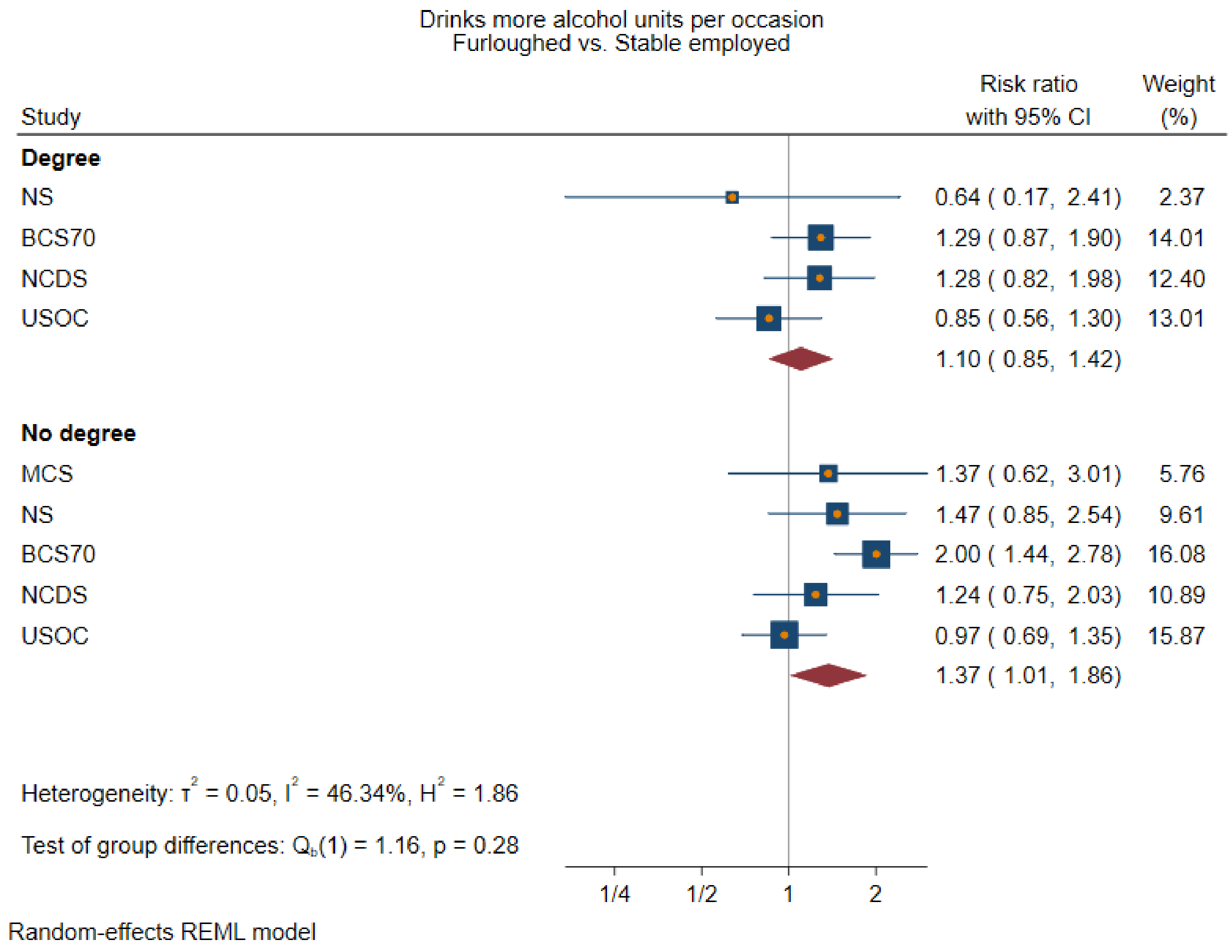

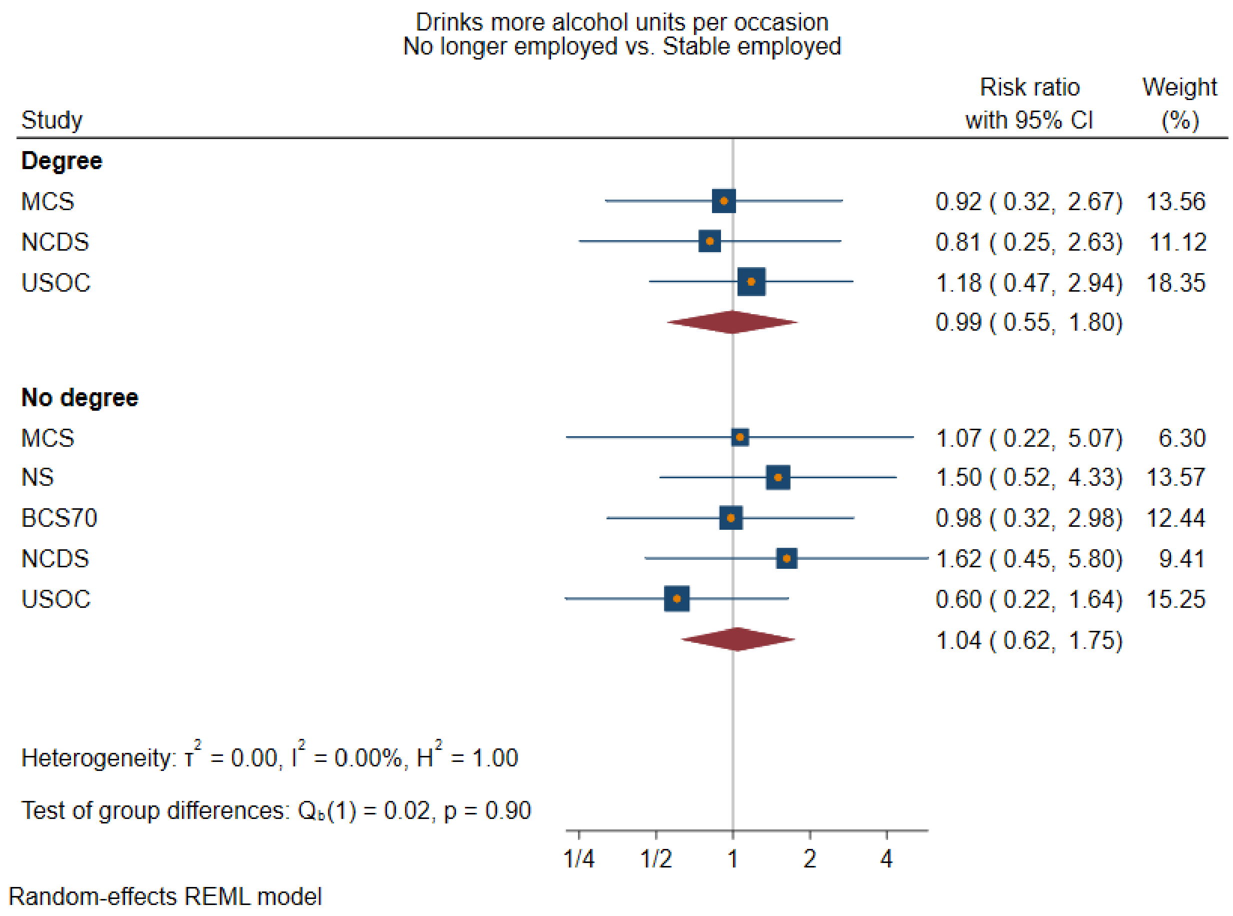

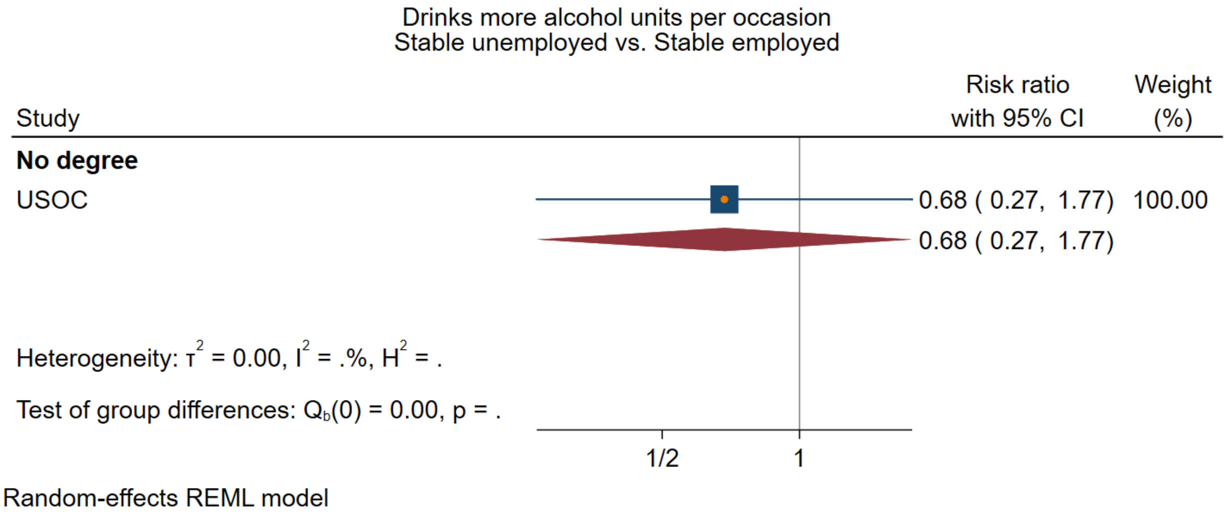
Drinks more alcohol units per occasion
Stable unemployed vs. Stable employed
Study
Risk ratio with 95% CI
Weight (%)
No degree
USOC
0.68 (0.27, 1.77)
100.00
0.68 (0.27, 1.77)
Heterogeneity: τ^2 = 0.00, I^2 = .%, H^2 = .
Test of group differences: $Q_b(0)$ = 0.00, p = .
1/2
1
Random-effects REML model

Figure set 21: Drinks fewer alcohol units per occasions

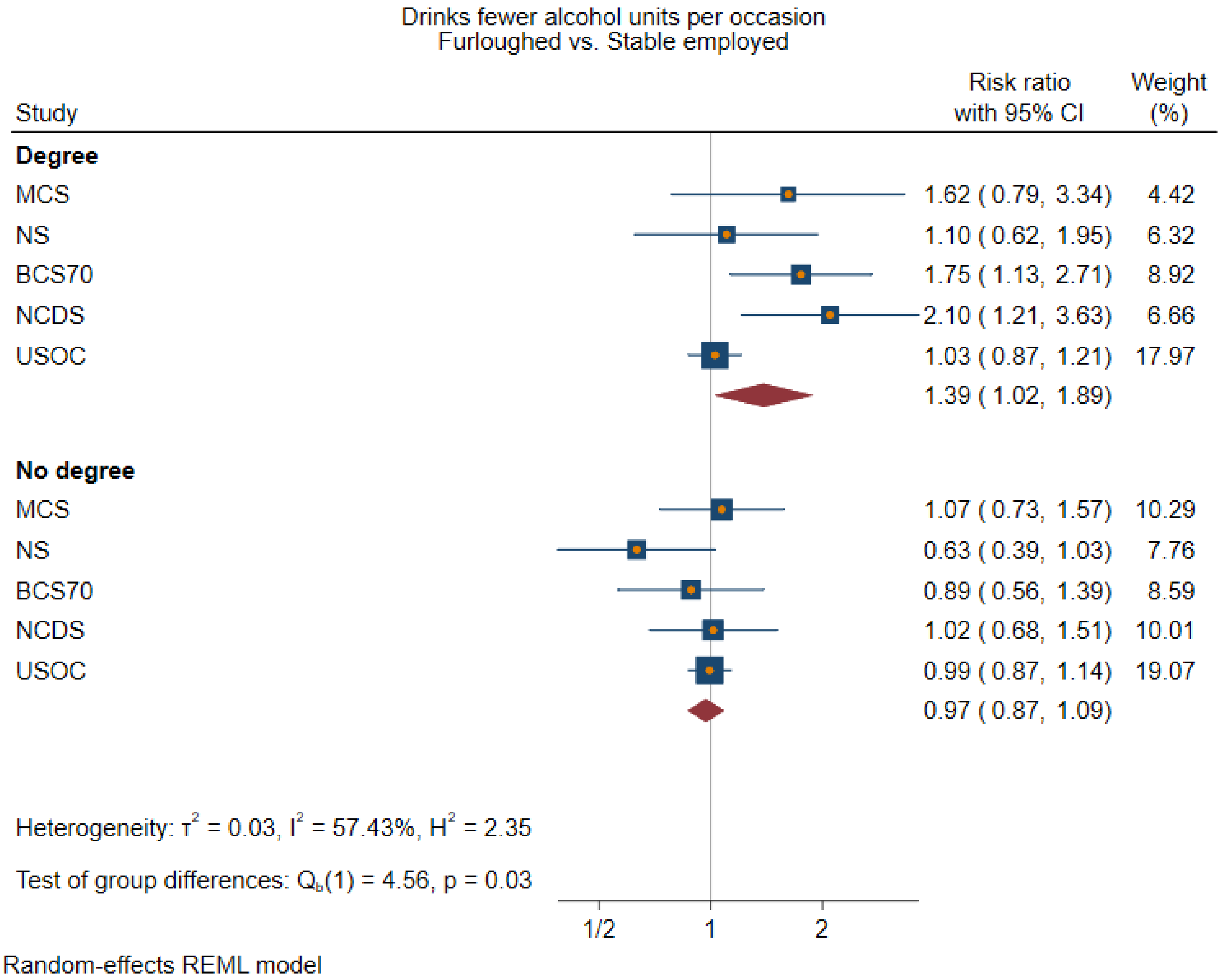

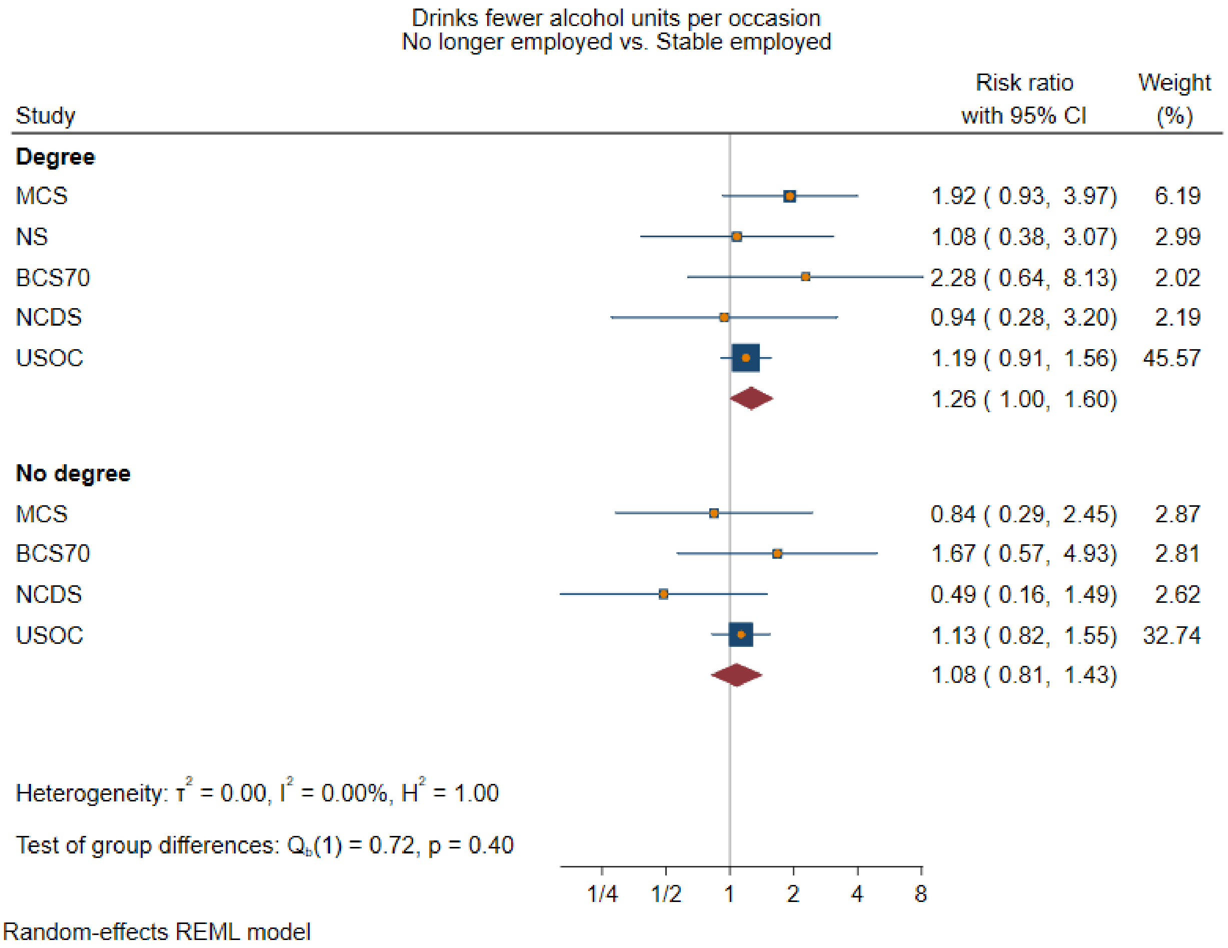

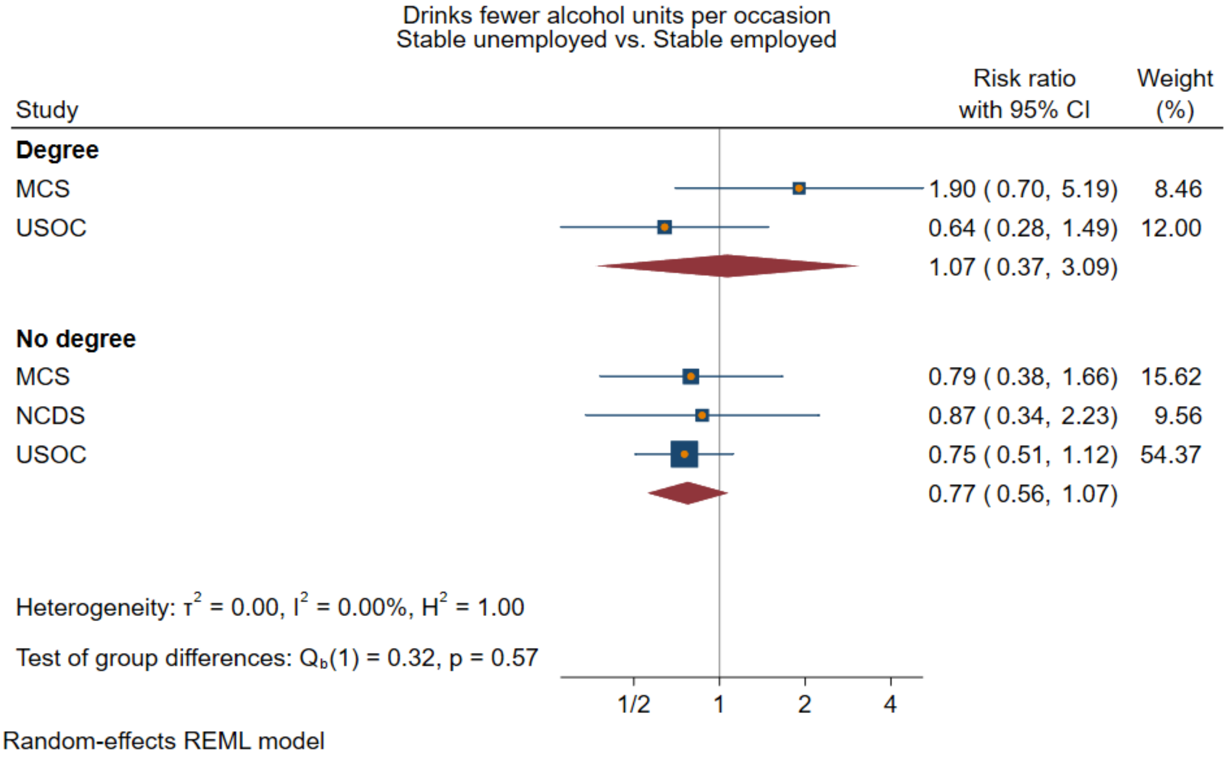
Drinks fewer alcohol units per occasion
Stable unemployed vs. Stable employed
Study
Risk ratio with 95% CI
Weight (%)
Degree
MCS 1.90 (0.70, 5.19) 8.46
USOC 0.64 (0.28, 1.49) 12.00
1.07 (0.37, 3.09)
No degree
MCS 0.79 (0.38, 1.66) 15.62
NCDS 0.87 (0.34, 2.23) 9.56
USOC 0.75 (0.51, 1.12) 54.37
0.77 (0.56, 1.07)
Heterogeneity: τ² = 0.00, I² = 0.00%, H² = 1.00
Test of group differences: Qb(1) = 0.32, p = 0.57
1/2
1
2
4
Random-effects REML model

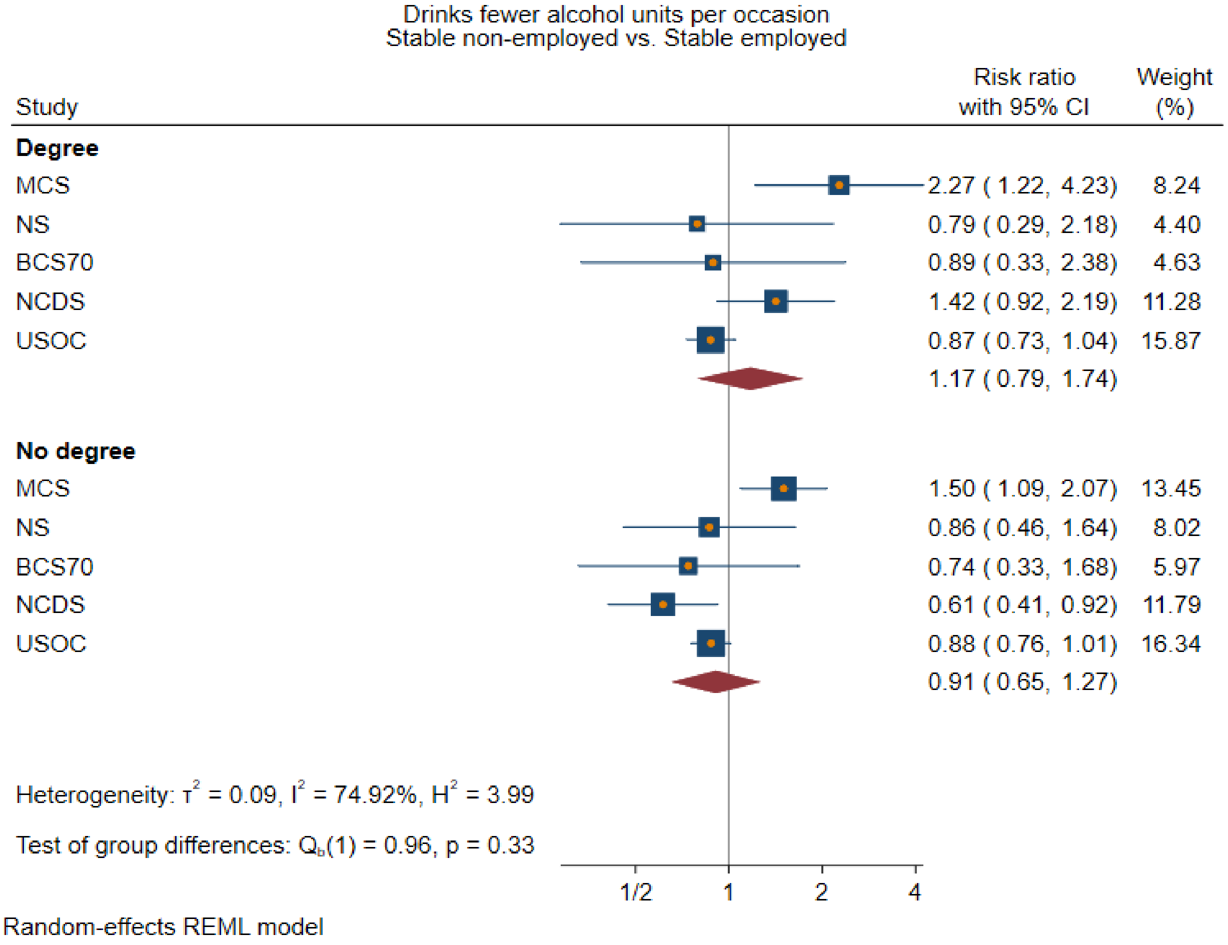
Drinks fewer alcohol units per occasion
Stable non-employed vs. Stable employed
Study
Risk ratio with 95% CI
Weight (%)
Degree
MCS 2.27 (1.22, 4.23) 8.24
NS 0.79 (0.29, 2.18) 4.40
BCS70 0.89 (0.33, 2.38) 4.63
NCDS 1.42 (0.92, 2.19) 11.28
USOC 0.87 (0.73, 1.04) 15.87
1.17 (0.79, 1.74)
No degree
MCS 1.50 (1.09, 2.07) 13.45
NS 0.86 (0.46, 1.64) 8.02
BCS70 0.74 (0.33, 1.68) 5.97
NCDS 0.61 (0.41, 0.92) 11.79
USOC 0.88 (0.76, 1.01) 16.34
0.91 (0.65, 1.27)
Heterogeneity: τ² = 0.09, I² = 74.92%, H² = 3.99
Test of group differences: Qb(1) = 0.96, p = 0.33
1/2
1
2
4
Random-effects REML model

Figure set 22: Currently drinks 4+ days/week

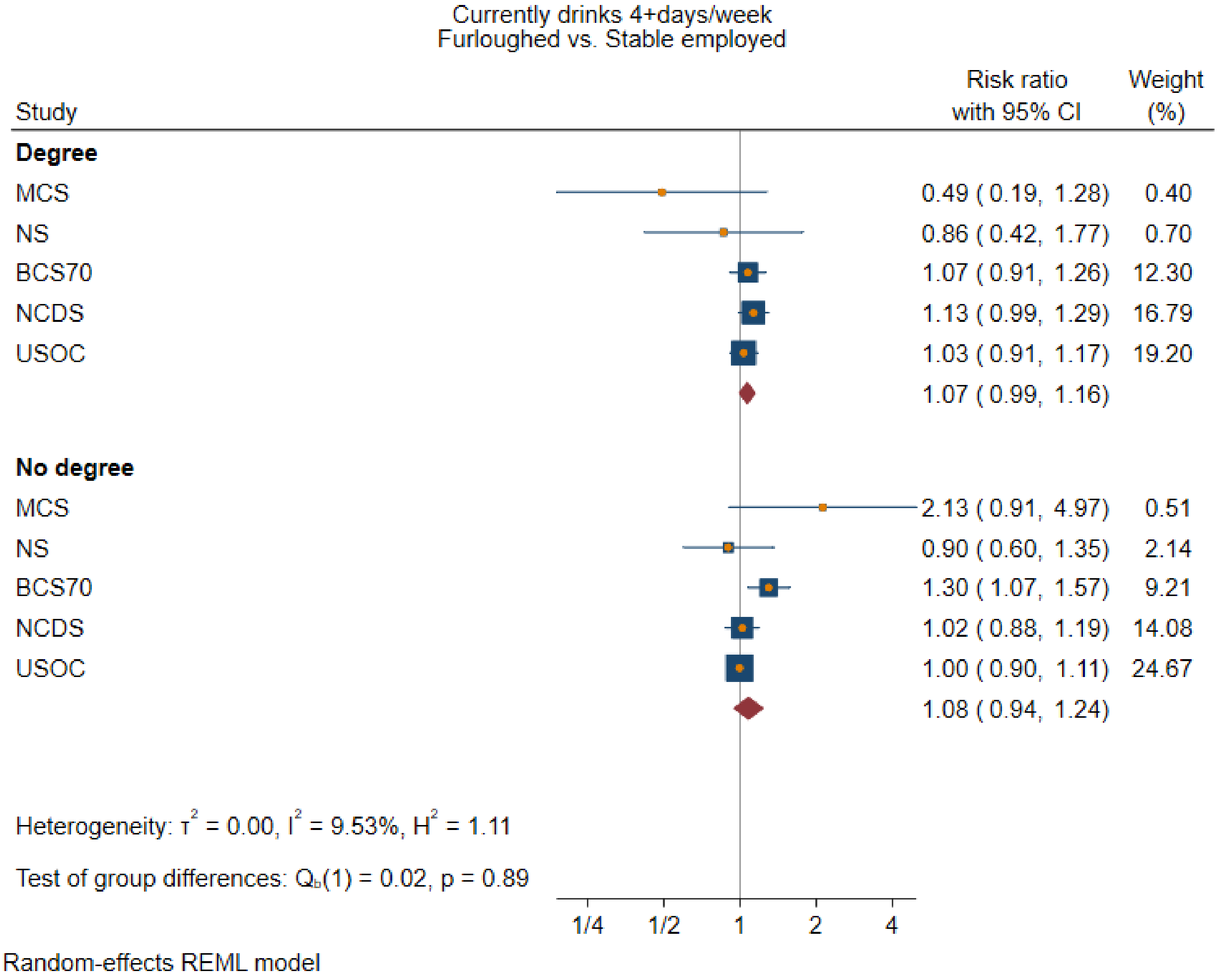

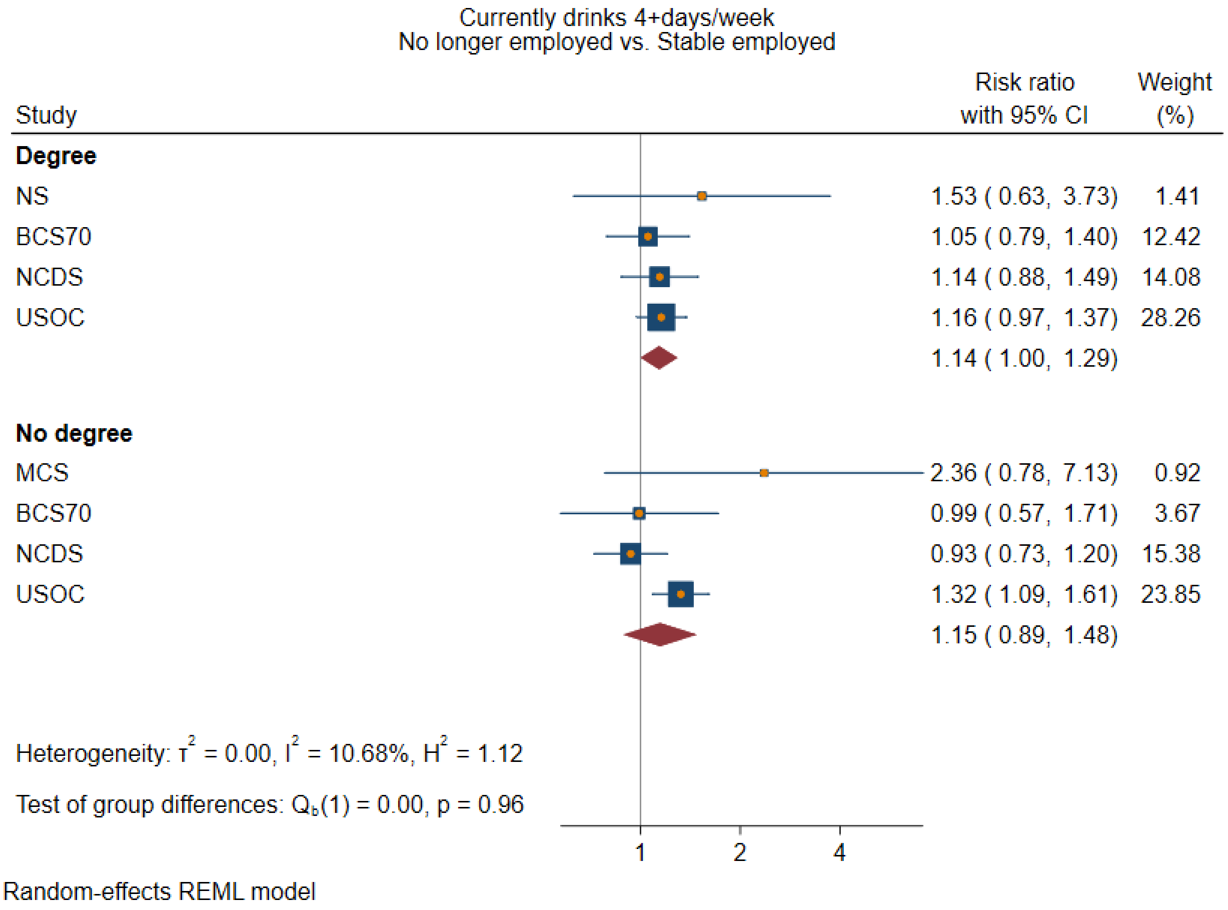

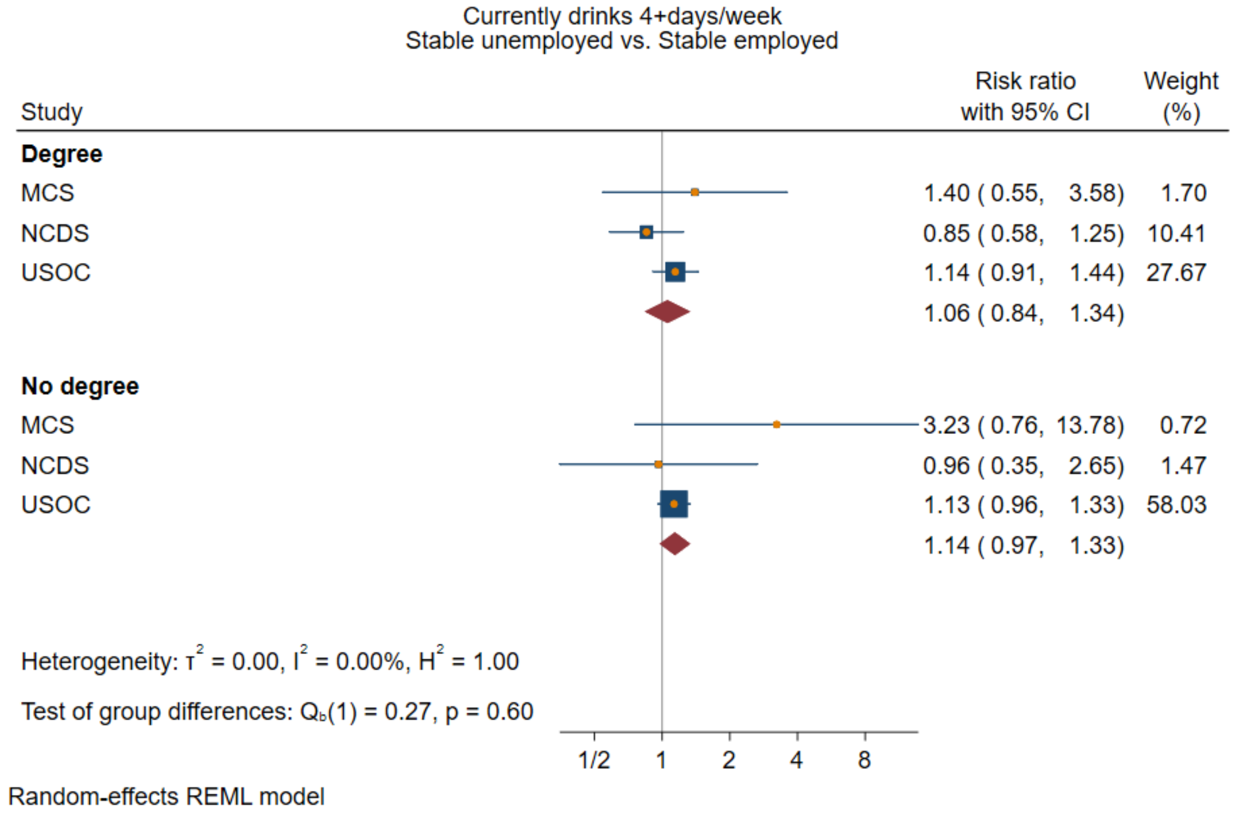
Currently drinks 4+days/week
Stable unemployed vs. Stable employed
Study
Risk ratio with 95% CI
Weight (%)
Degree
MCS 1.40 (0.55, 3.58) 1.70
NCDS 0.85 (0.58, 1.25) 10.41
USOC 1.14 (0.91, 1.44) 27.67
1.06 (0.84, 1.34)
No degree
MCS 3.23 (0.76, 13.78) 0.72
NCDS 0.96 (0.35, 2.65) 1.47
USOC 1.13 (0.96, 1.33) 58.03
1.14 (0.97, 1.33)
Heterogeneity: τ² = 0.00, I² = 0.00%, H² = 1.00
Test of group differences: Q_b(1) = 0.27, p = 0.60
1/2
1
2
4
8
Random-effects REML model

Figure set 23: Drinks more frequently

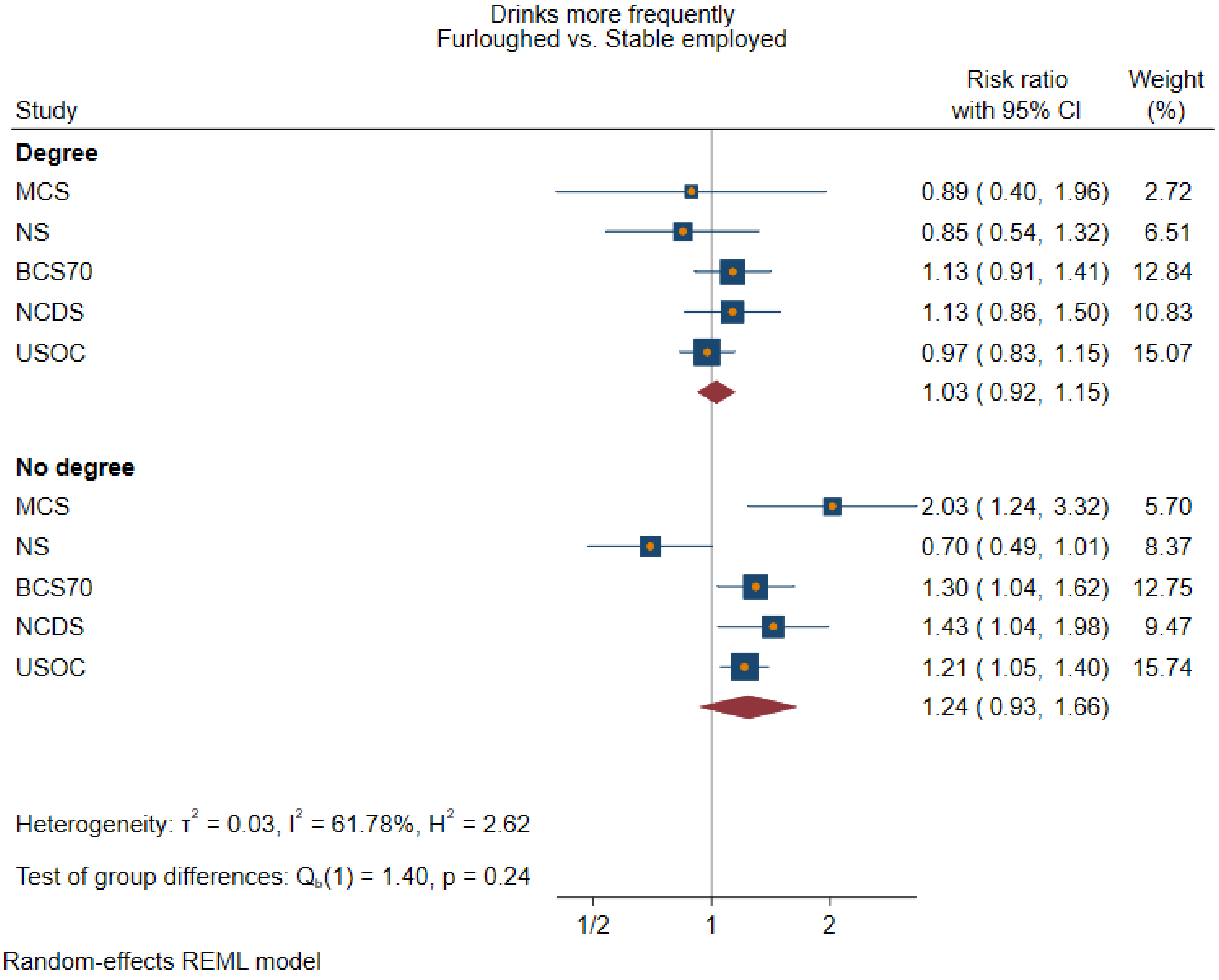

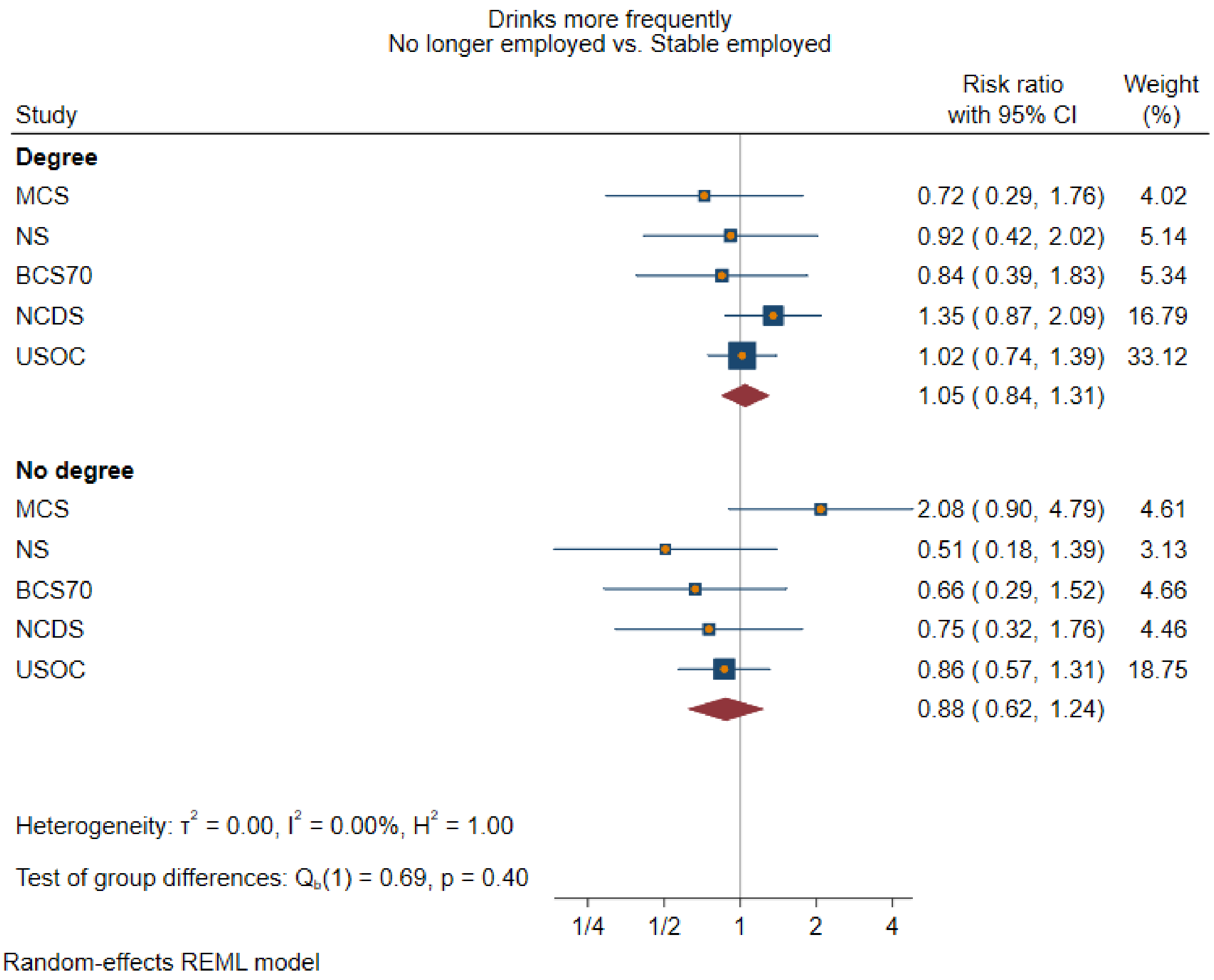

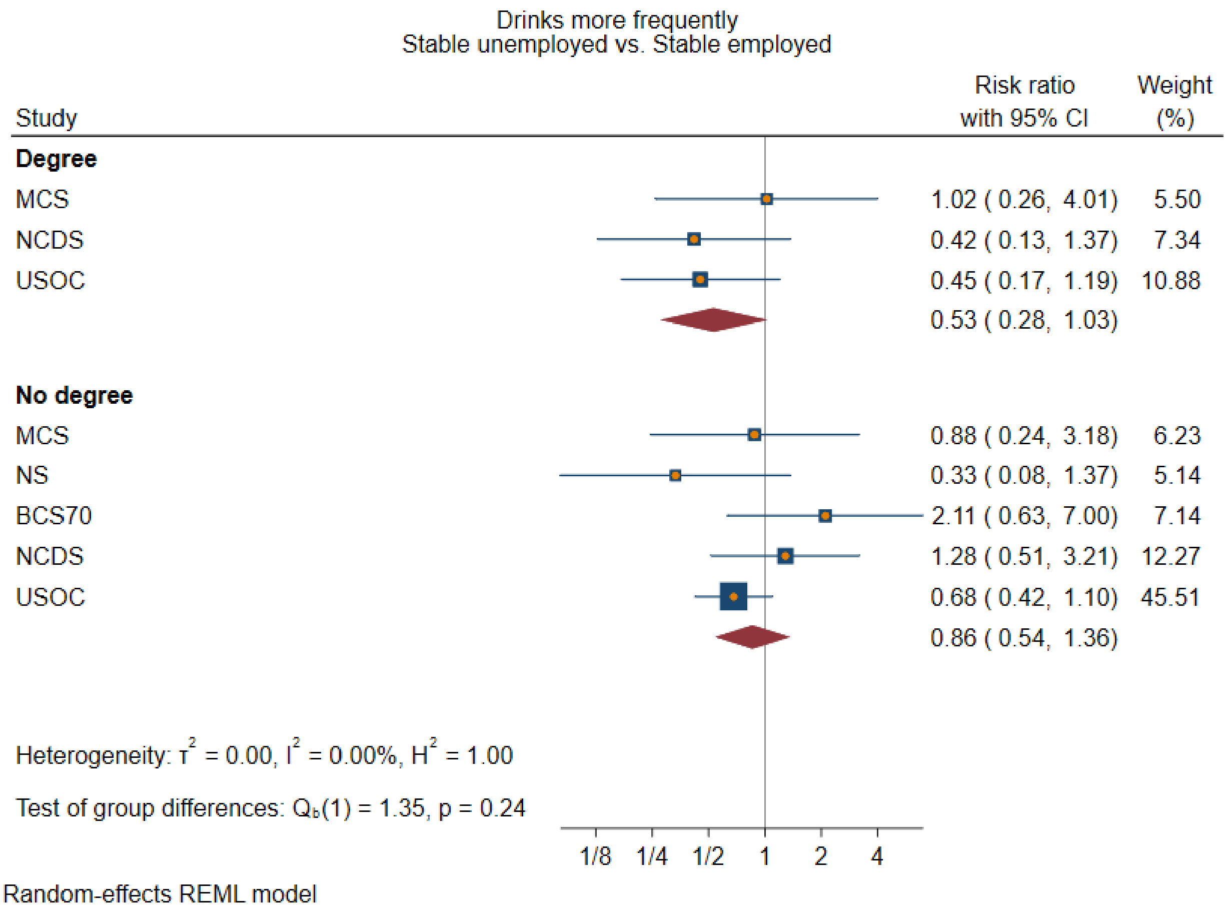
Drinks more frequently
Stable unemployed vs. Stable employed
Study
Risk ratio with 95% CI
Weight (%)
Degree
MCS 1.02 (0.26, 4.01) 5.50
NCDS 0.42 (0.13, 1.37) 7.34
USOC 0.45 (0.17, 1.19) 10.88
0.53 (0.28, 1.03)
No degree
MCS 0.88 (0.24, 3.18) 6.23
NS 0.33 (0.08, 1.37) 5.14
BCS70 2.11 (0.63, 7.00) 7.14
NCDS 1.28 (0.51, 3.21) 12.27
USOC 0.68 (0.42, 1.10) 45.51
0.86 (0.54, 1.36)
Heterogeneity: τ² = 0.00, I² = 0.00%, H² = 1.00
Test of group differences: Qb(1) = 1.35, p = 0.24
1/8 1/4 1/2 1 2 4
Random-effects REML model

Figure set 24: Drinks less frequently

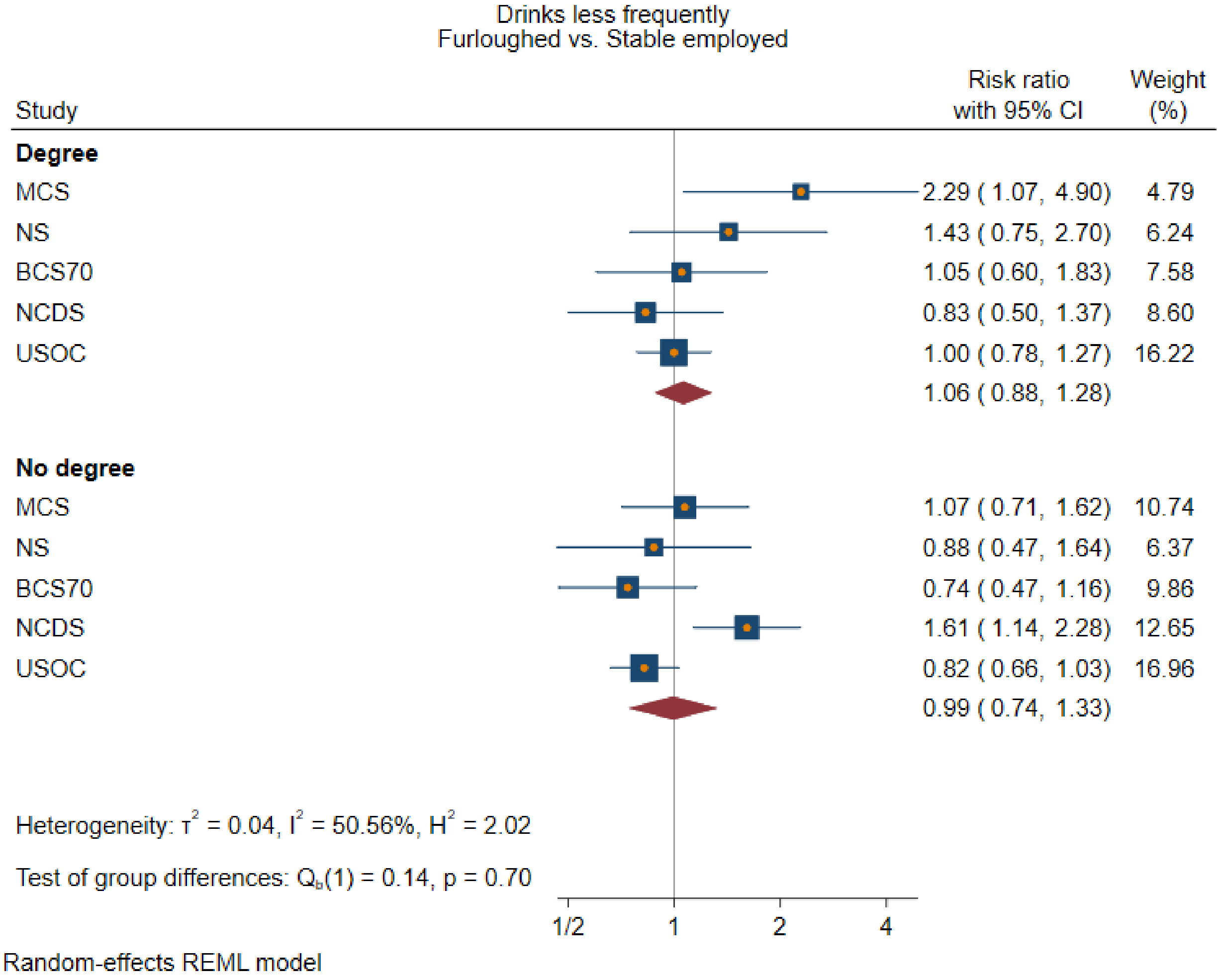

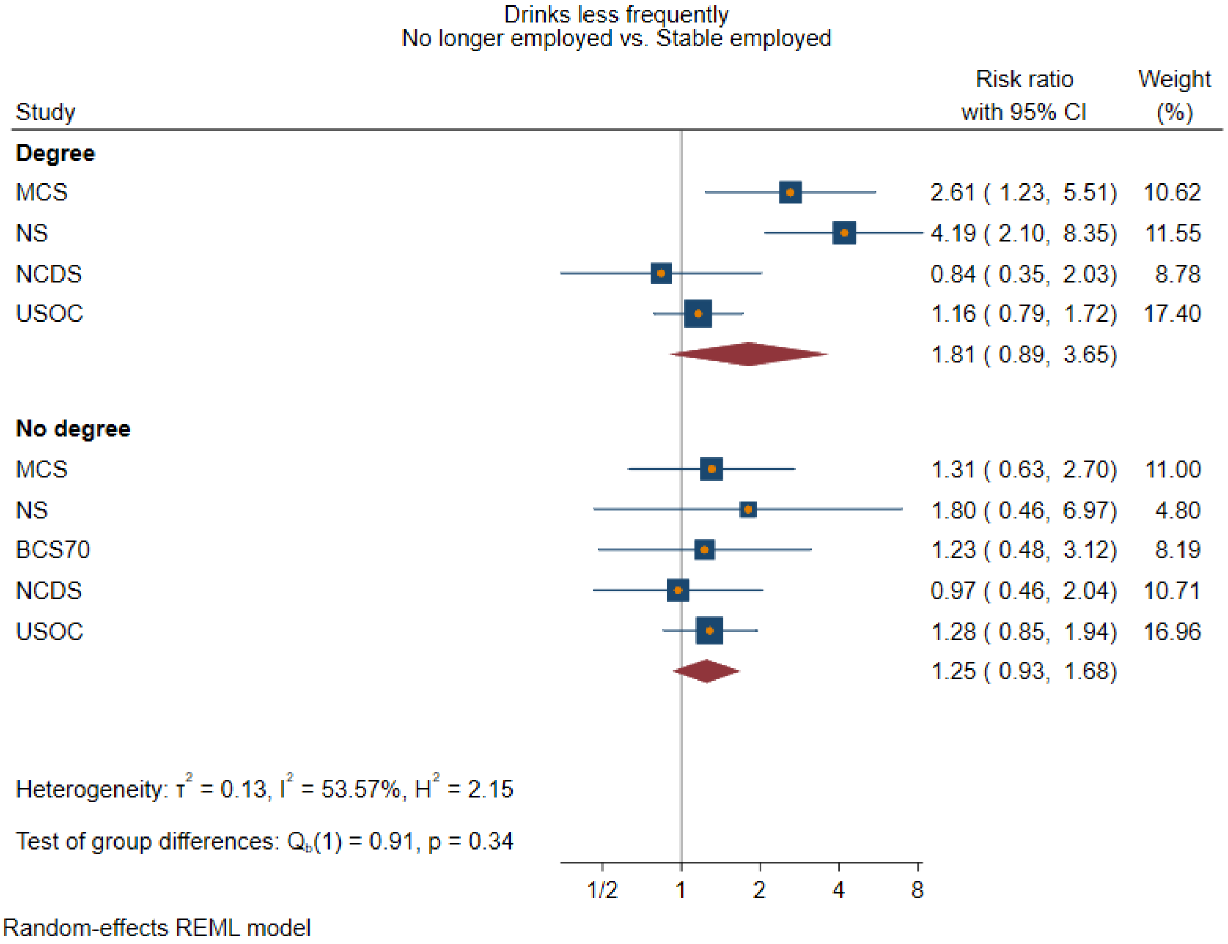

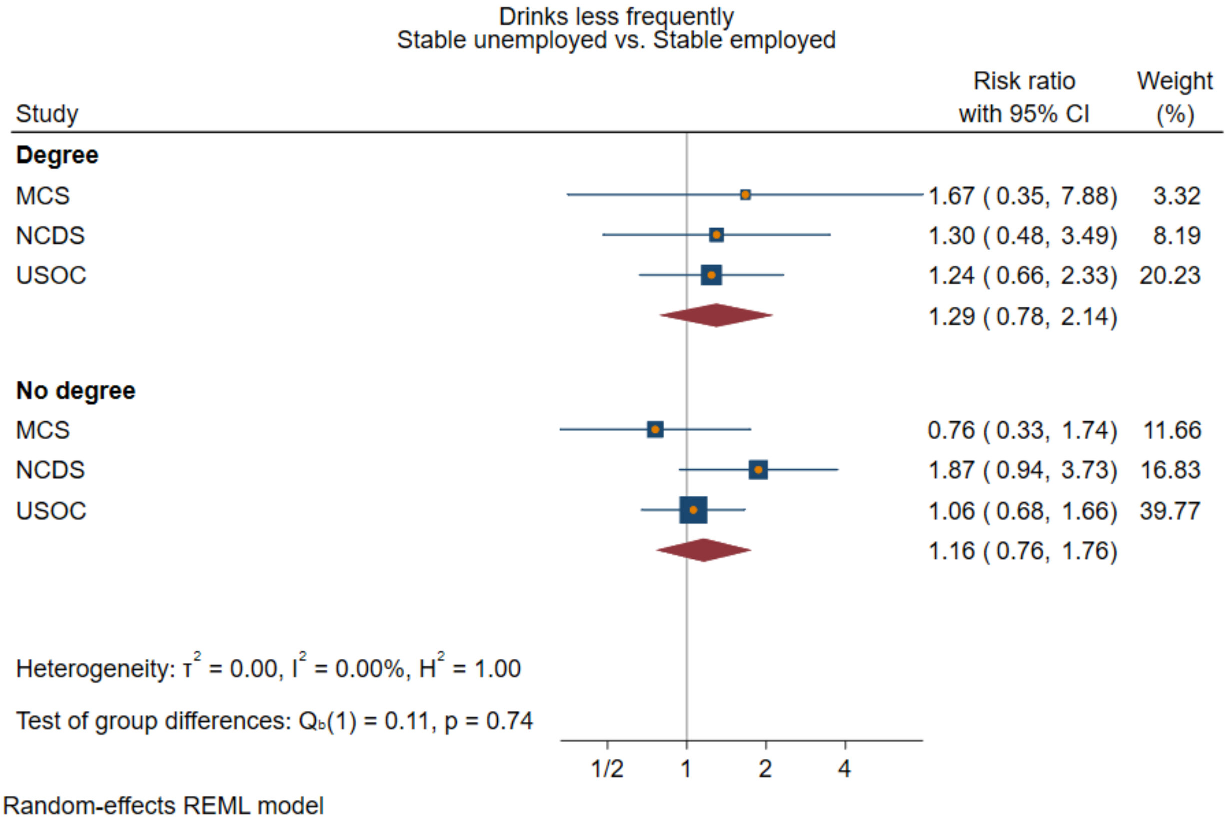
Drinks less frequently
Stable unemployed vs. Stable employed
Study
Risk ratio with 95% CI
Weight (%)
Degree
MCS 1.67 (0.35, 7.88) 3.32
NCDS 1.30 (0.48, 3.49) 8.19
USOC 1.24 (0.66, 2.33) 20.23
1.29 (0.78, 2.14)
No degree
MCS 0.76 (0.33, 1.74) 11.66
NCDS 1.87 (0.94, 3.73) 16.83
USOC 1.06 (0.68, 1.66) 39.77
1.16 (0.76, 1.76)
Heterogeneity: τ² = 0.00, I² = 0.00%, H² = 1.00
Test of group differences: Qb(1) = 0.11, p = 0.74
1/2
1
2
4
Random-effects REML model

Figure set 24: Current smoker

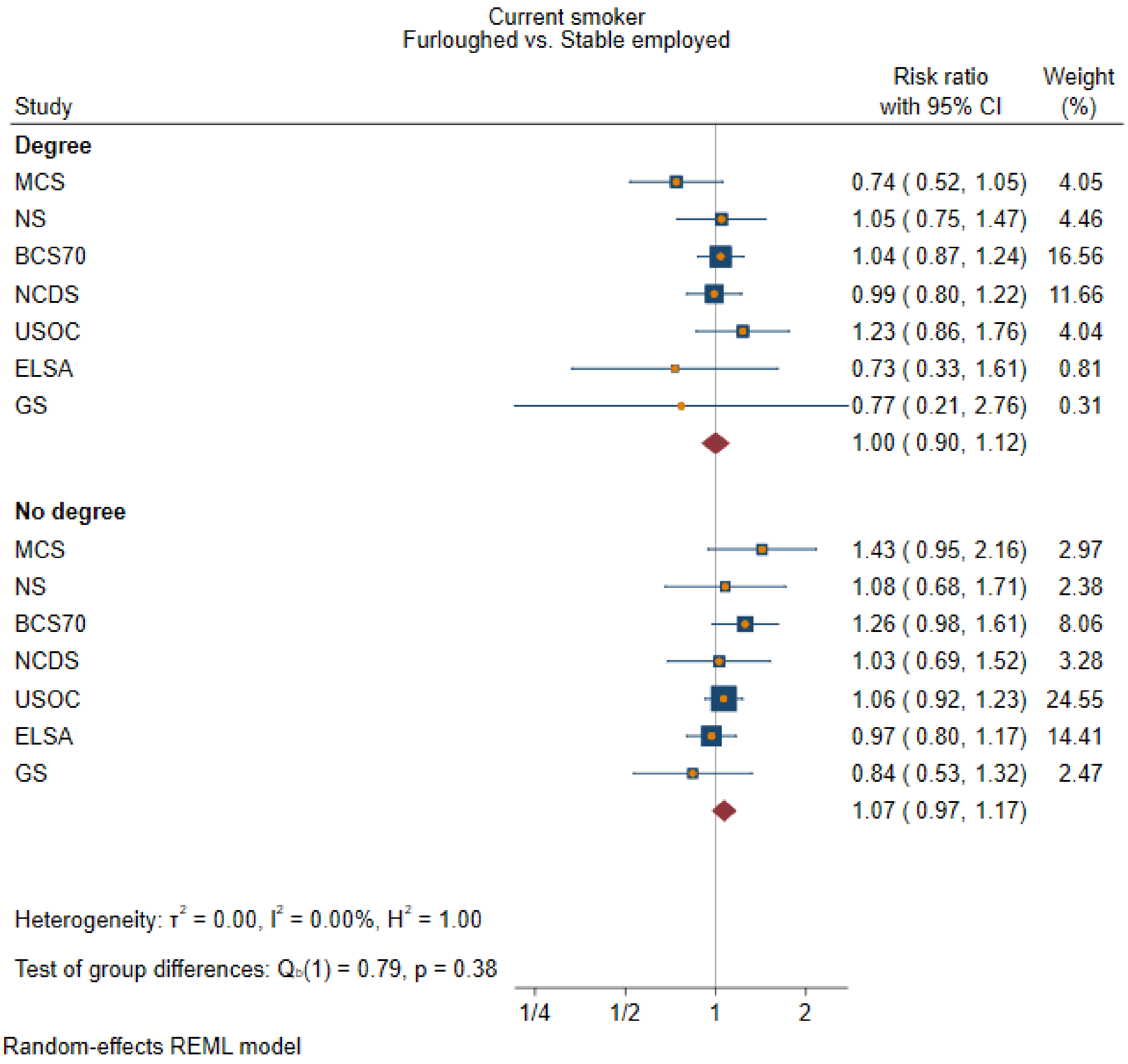

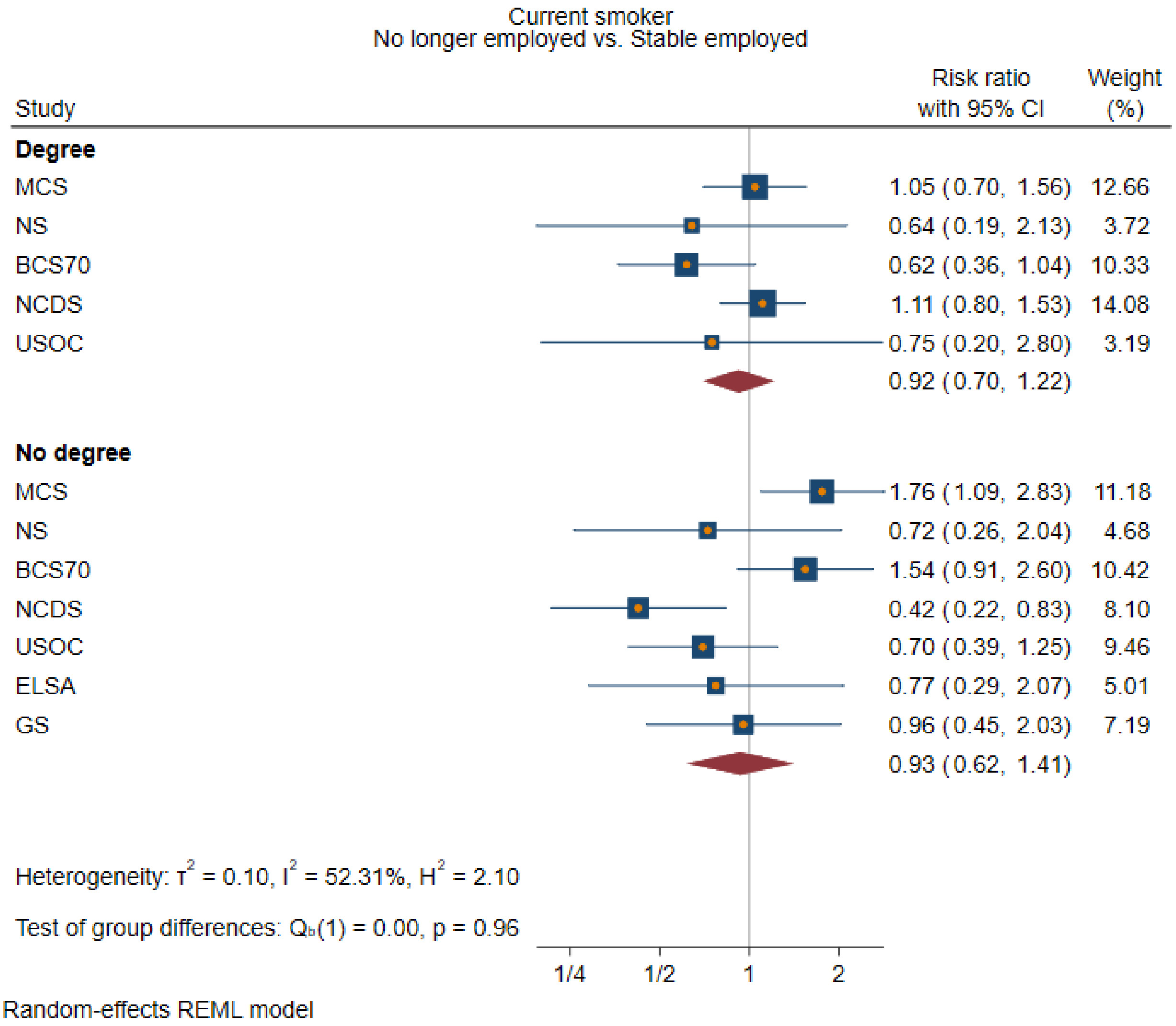

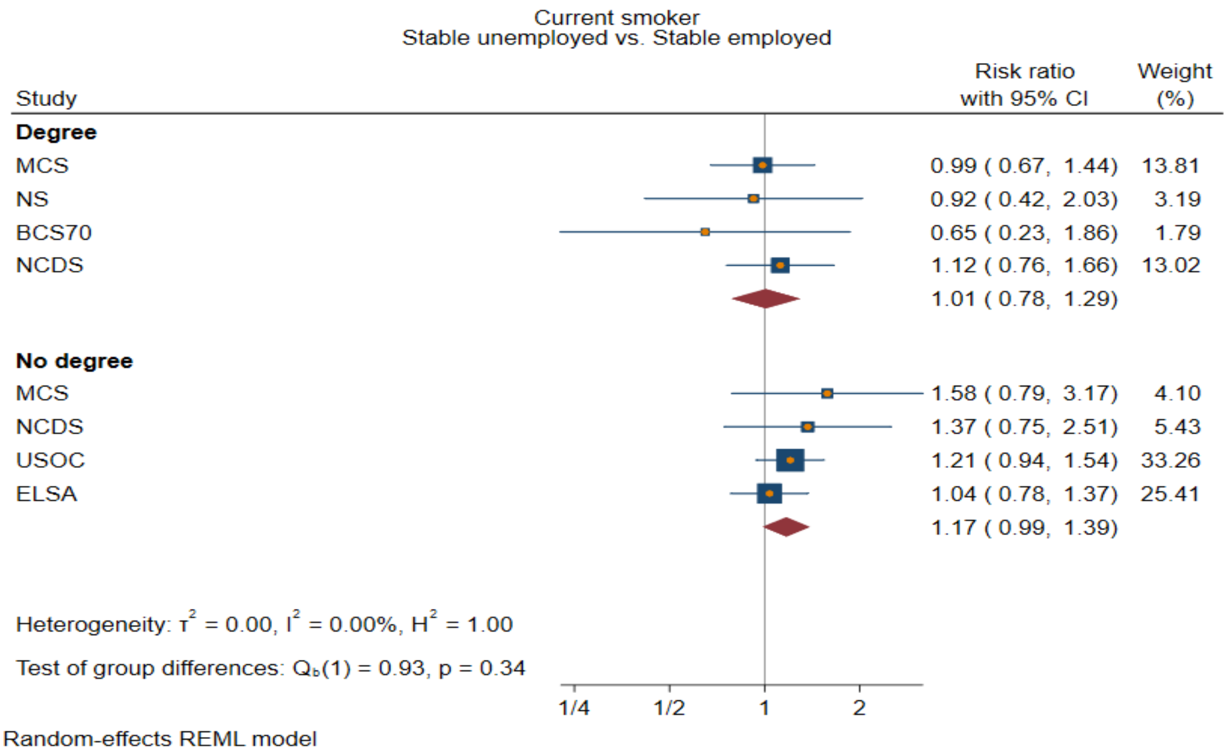
Current smoker
Stable unemployed vs. Stable employed
Study
Risk ratio with 95% CI
Weight (%)
Degree
MCS 0.99 (0.67, 1.44) 13.81
NS 0.92 (0.42, 2.03) 3.19
BCS70 0.65 (0.23, 1.86) 1.79
NCDS 1.12 (0.76, 1.66) 13.02
1.01 (0.78, 1.29)
No degree
MCS 1.58 (0.79, 3.17) 4.10
NCDS 1.37 (0.75, 2.51) 5.43
USOC 1.21 (0.94, 1.54) 33.26
ELSA 1.04 (0.78, 1.37) 25.41
1.17 (0.99, 1.39)
Heterogeneity: τ^2 = 0.00, I^2 = 0.00%, H^2 = 1.00
Test of group differences: $Q_b(1)$ = 0.93, p = 0.34
1/4
1/2
1
2
Random-effects REML model

Figure set 26: Smoking more

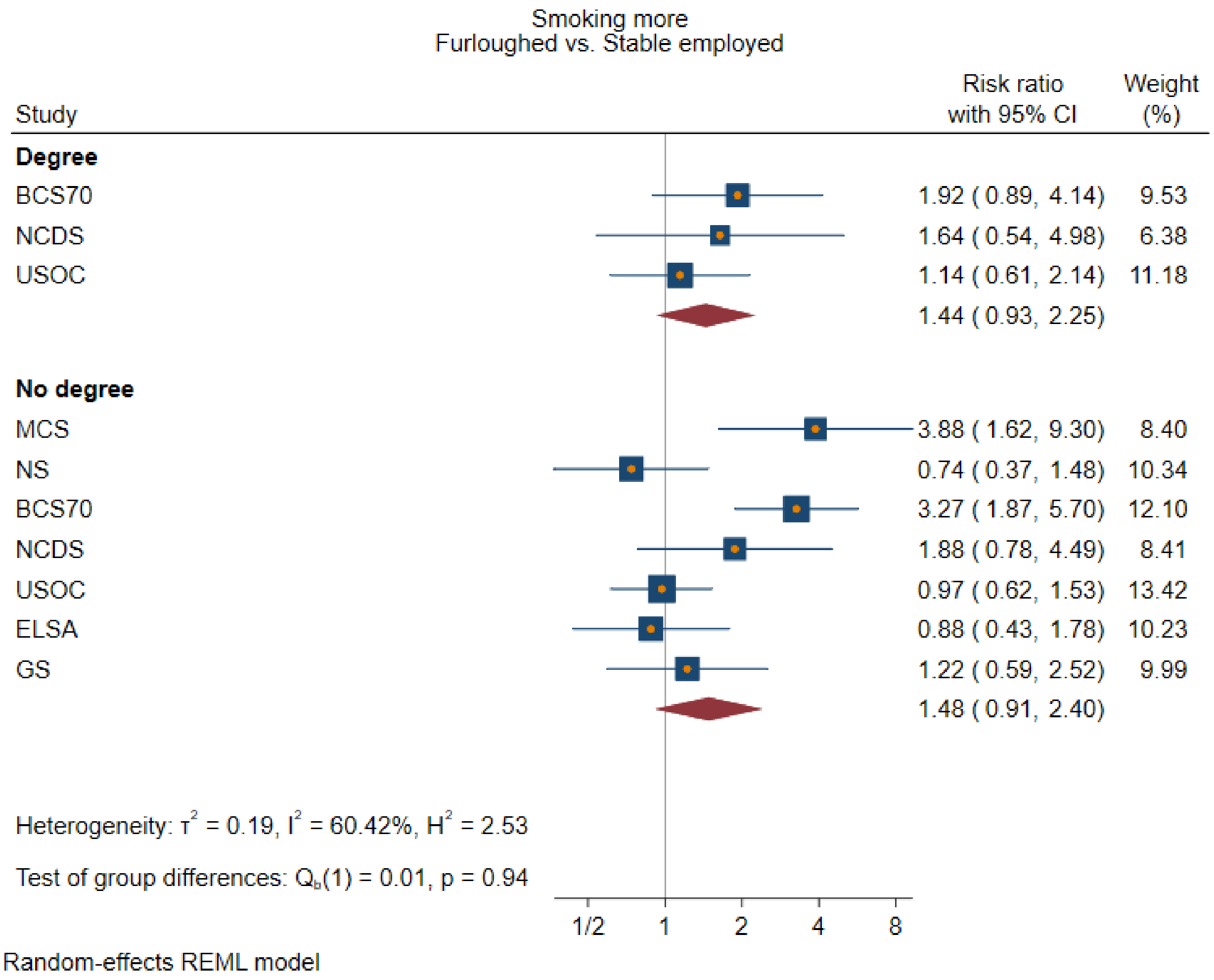

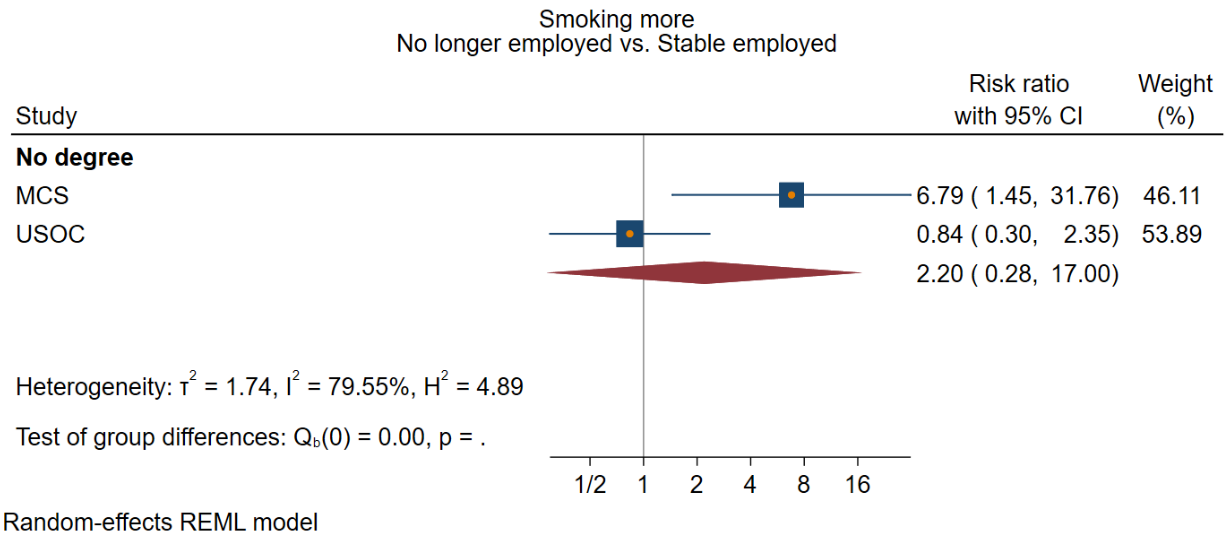

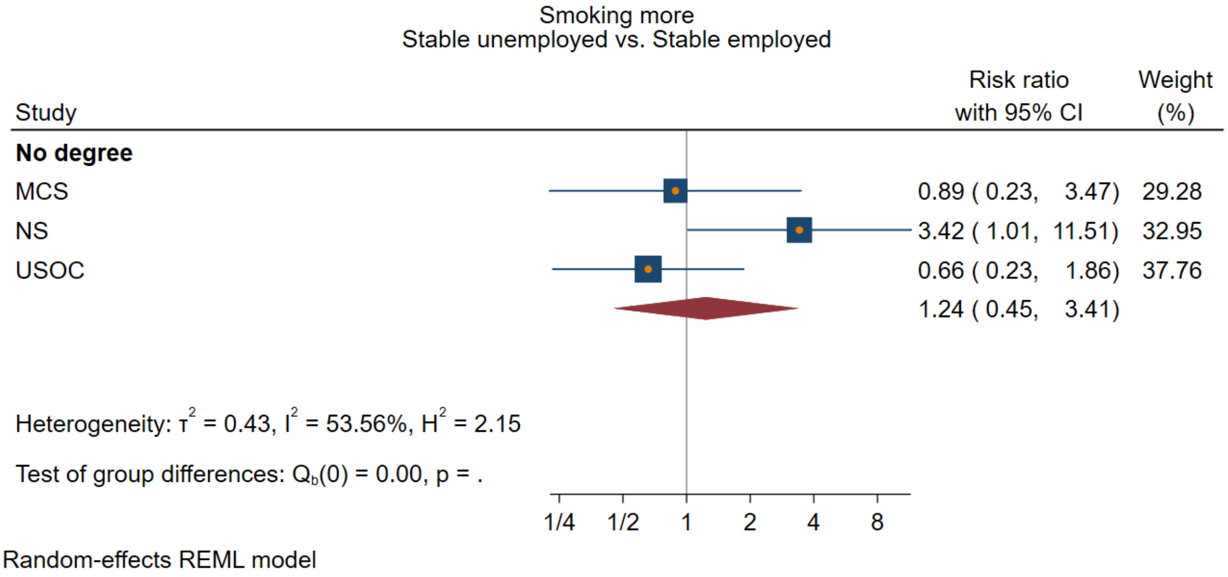

Figure set 27: Smoking less

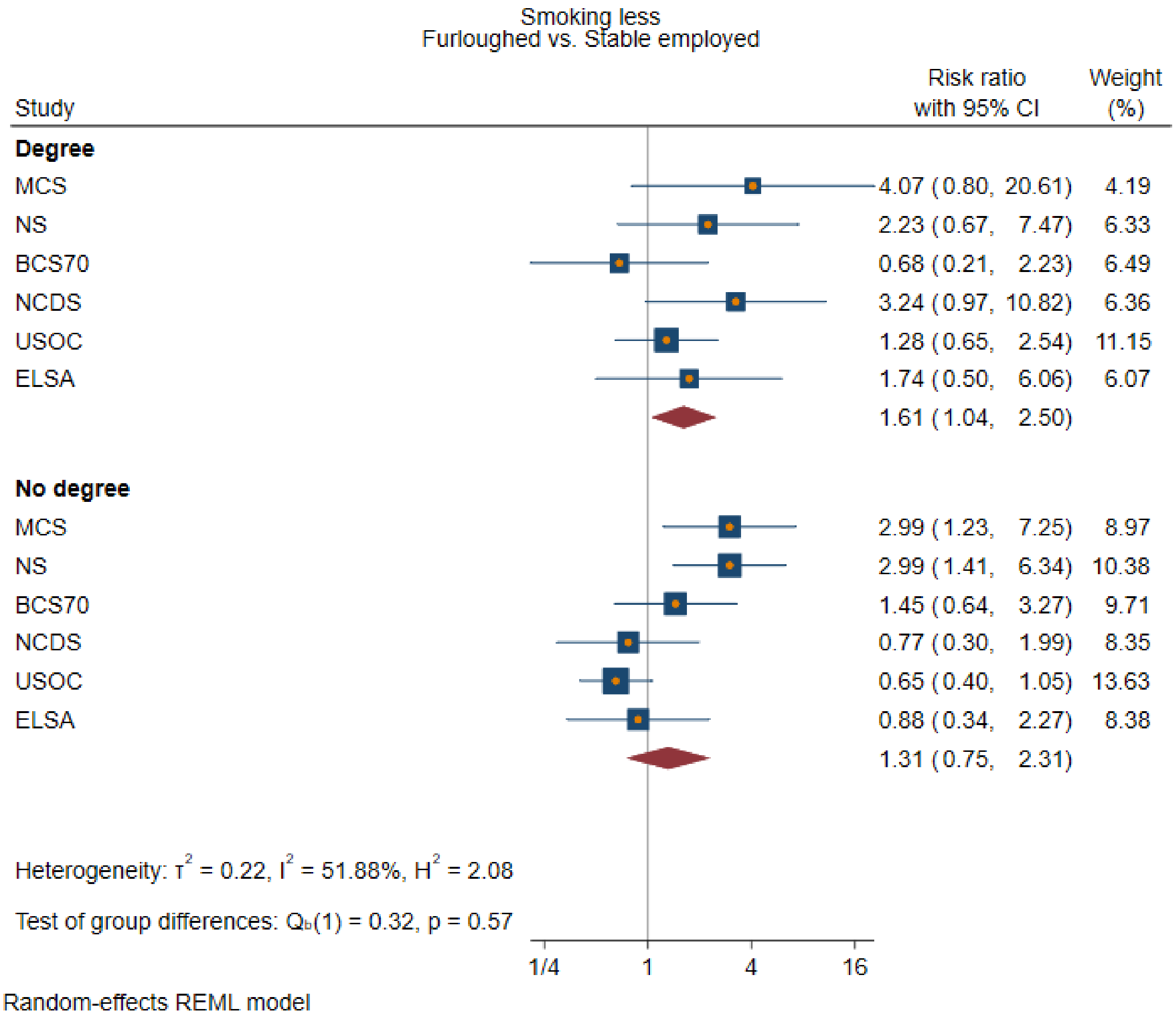

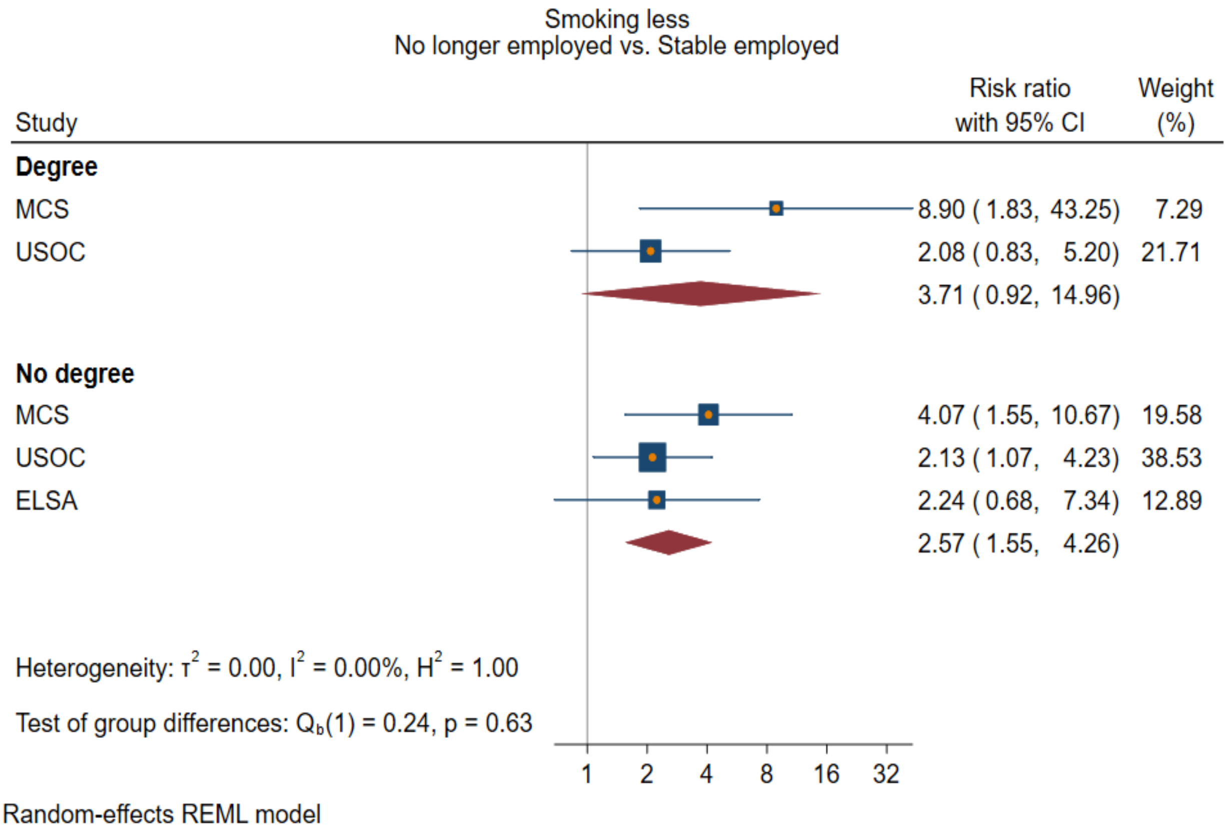

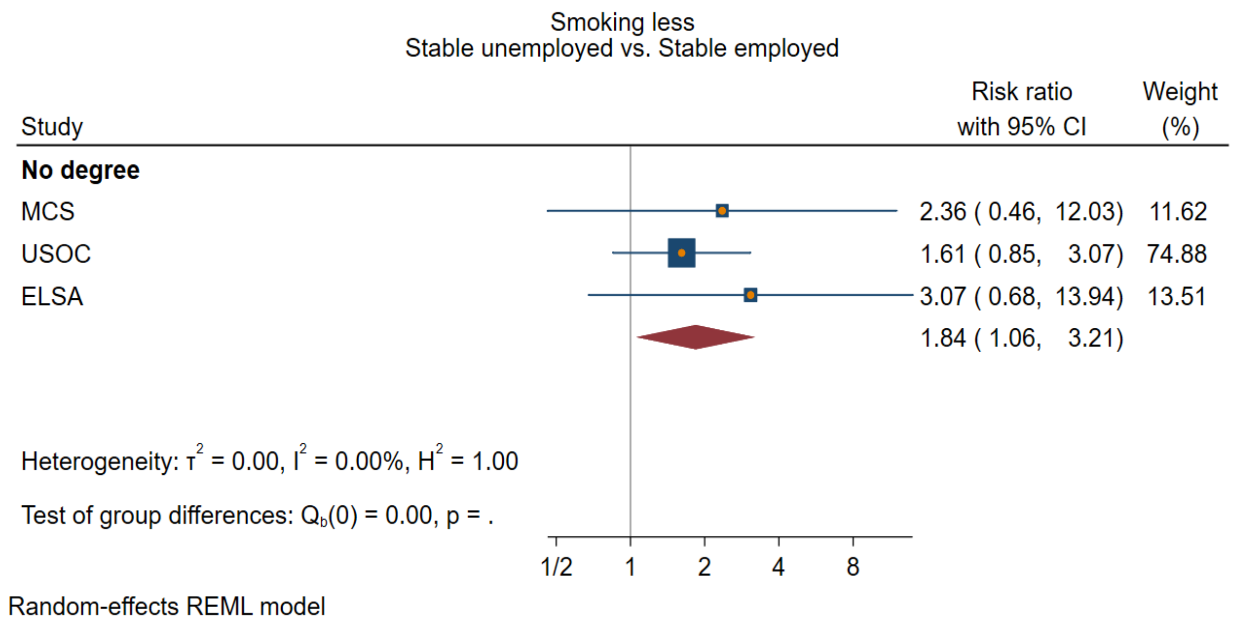
Smoking less
Stable unemployed vs. Stable employed
Study
Risk ratio with 95% CI
Weight (%)
No degree
MCS
2.36 (0.46, 12.03)
11.62
USOC
1.61 (0.85, 3.07)
74.88
ELSA
3.07 (0.68, 13.94)
13.51
1.84 (1.06, 3.21)
Heterogeneity: τ² = 0.00, I² = 0.00%, H² = 1.00
Test of group differences: Qb(0) = 0.00, p = .
1/2
1
2
4
8
Random-effects REML model

Figure set 28: Current vaper

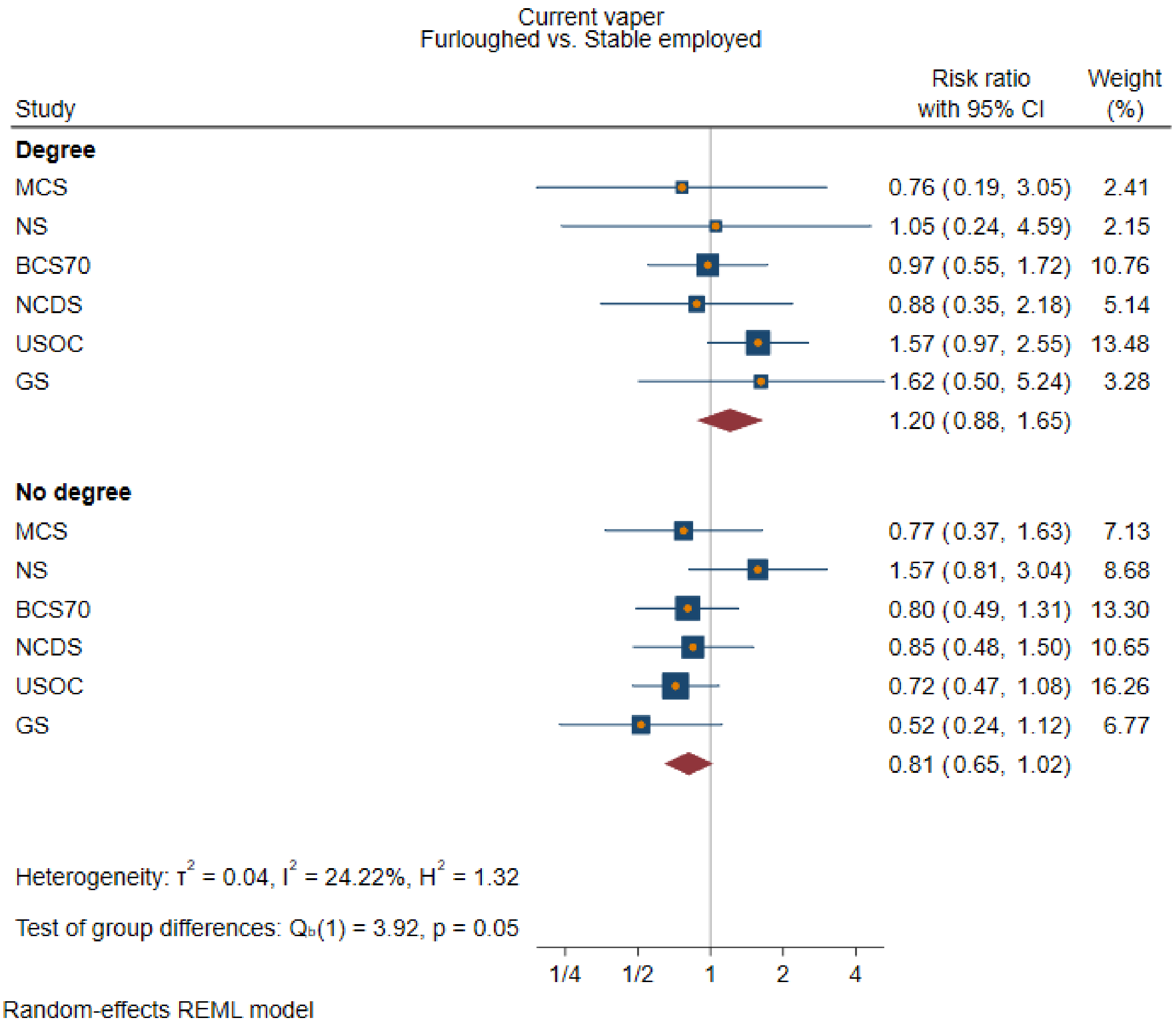

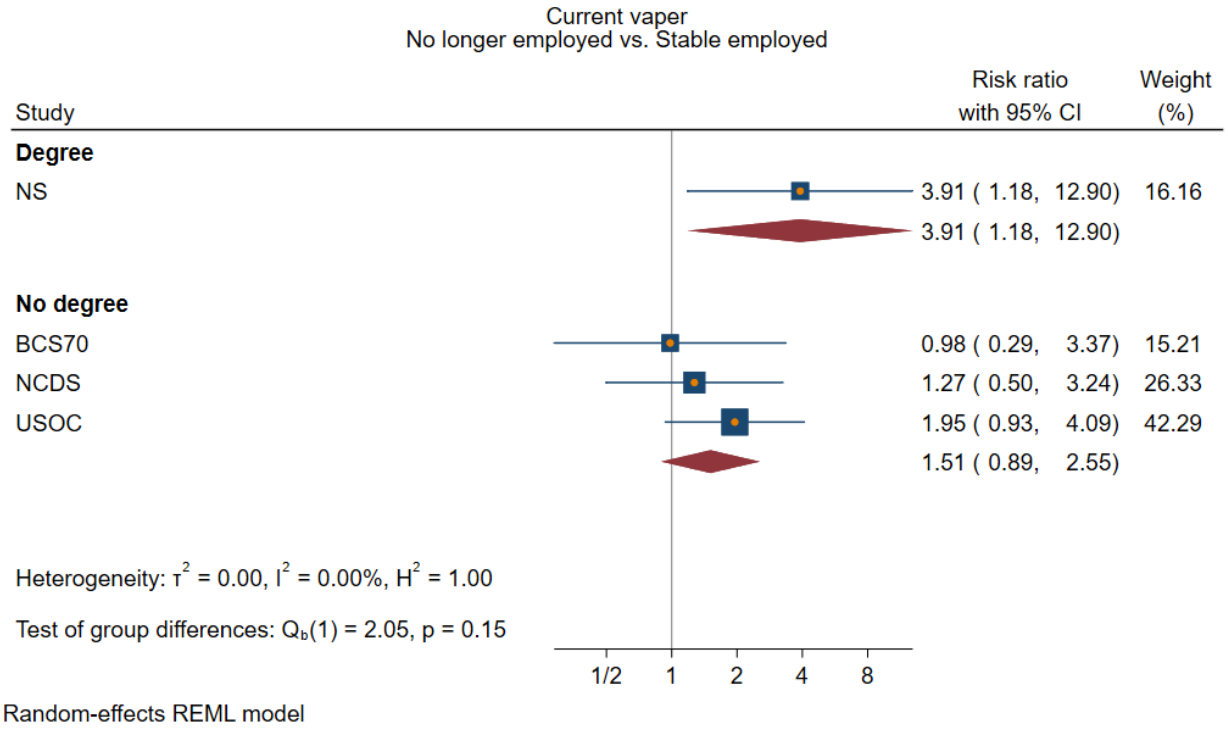

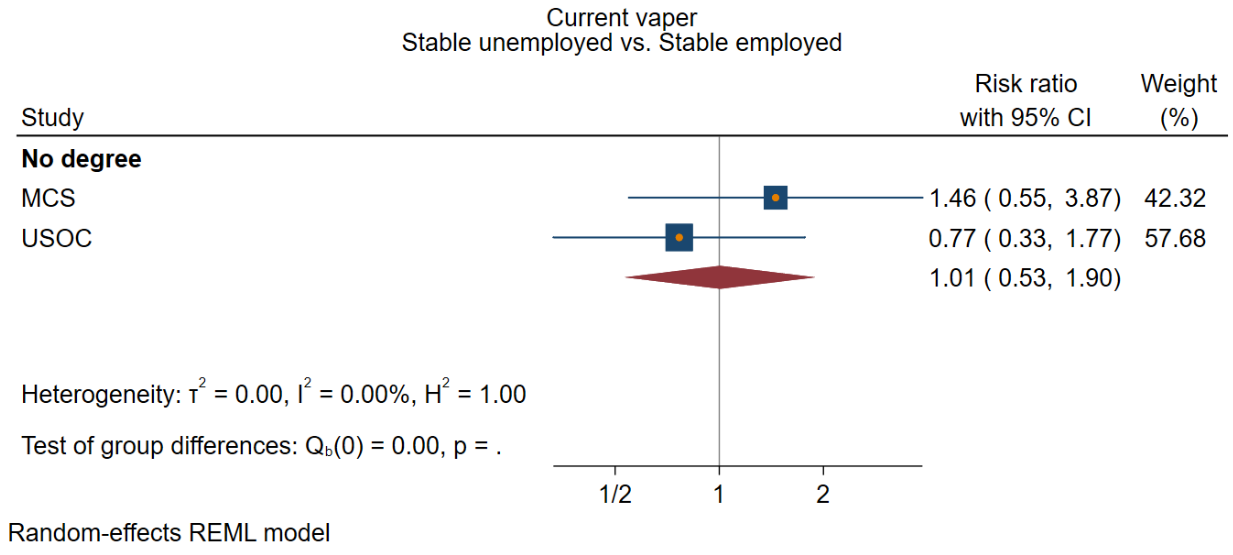

Current vaper
Stable unemployed vs. Stable employed
Study
Risk ratio with 95% CI
Weight (%)
No degree
MCS
1.46 (0.55, 3.87)
42.32
USOC
0.77 (0.33, 1.77)
57.68
1.01 (0.53, 1.90)
Heterogeneity: τ² = 0.00, I² = 0.00%, H² = 1.00
Test of group differences: Q_b(0) = 0.00, p = .
1/2
1
2
Random-effects REML model

Figure set 29: Vaping more

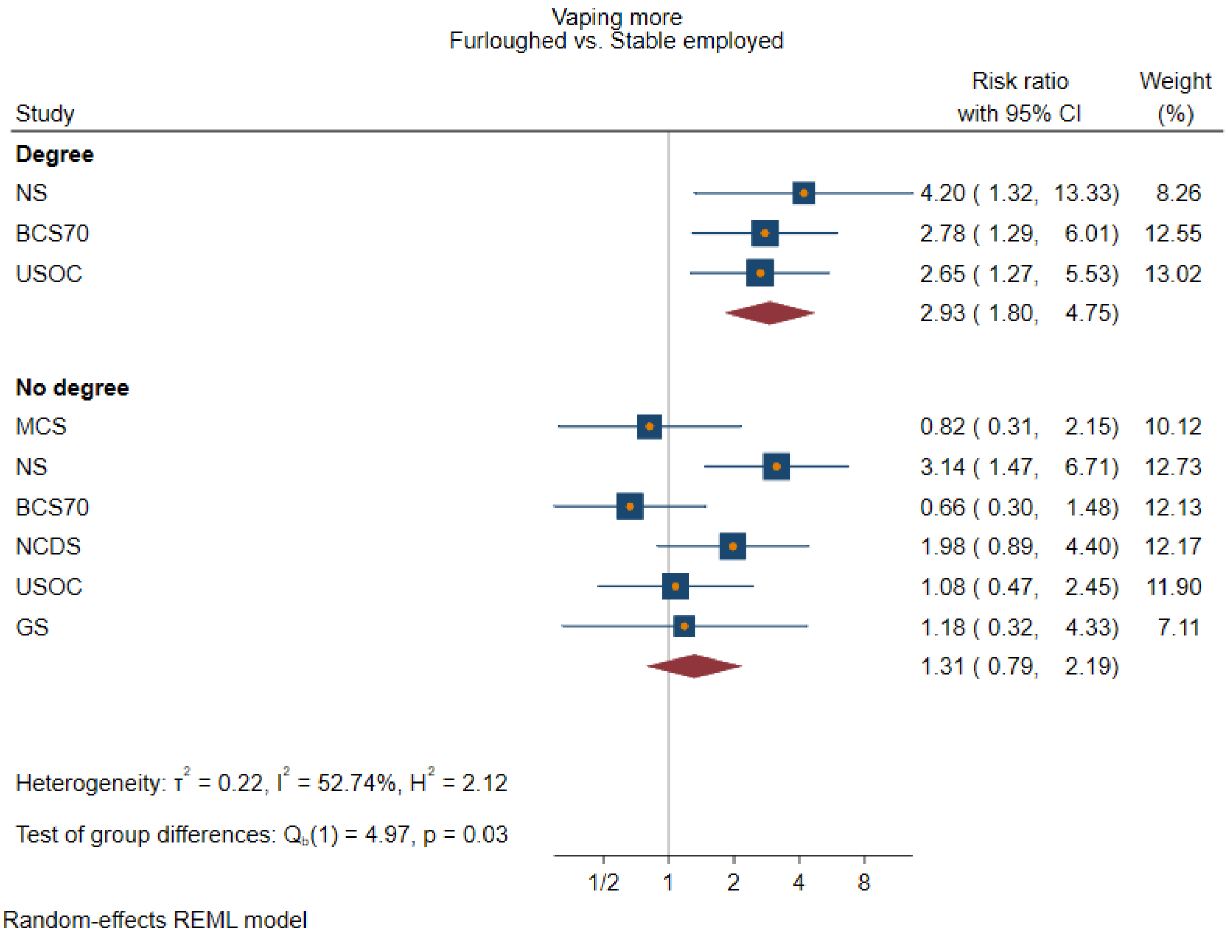

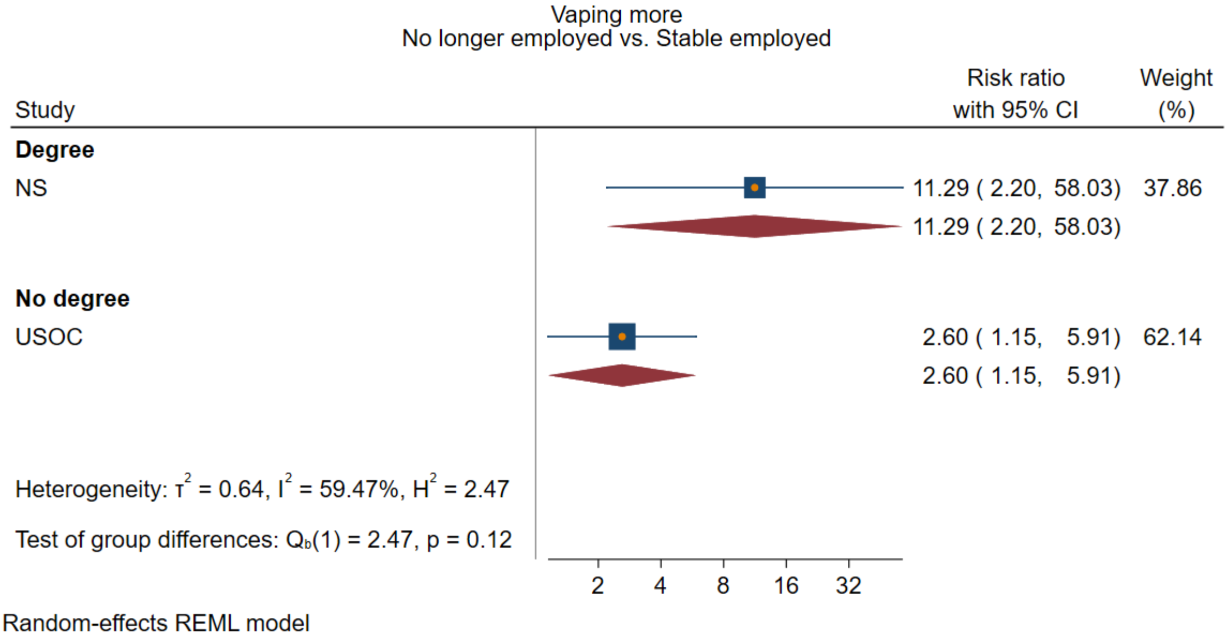

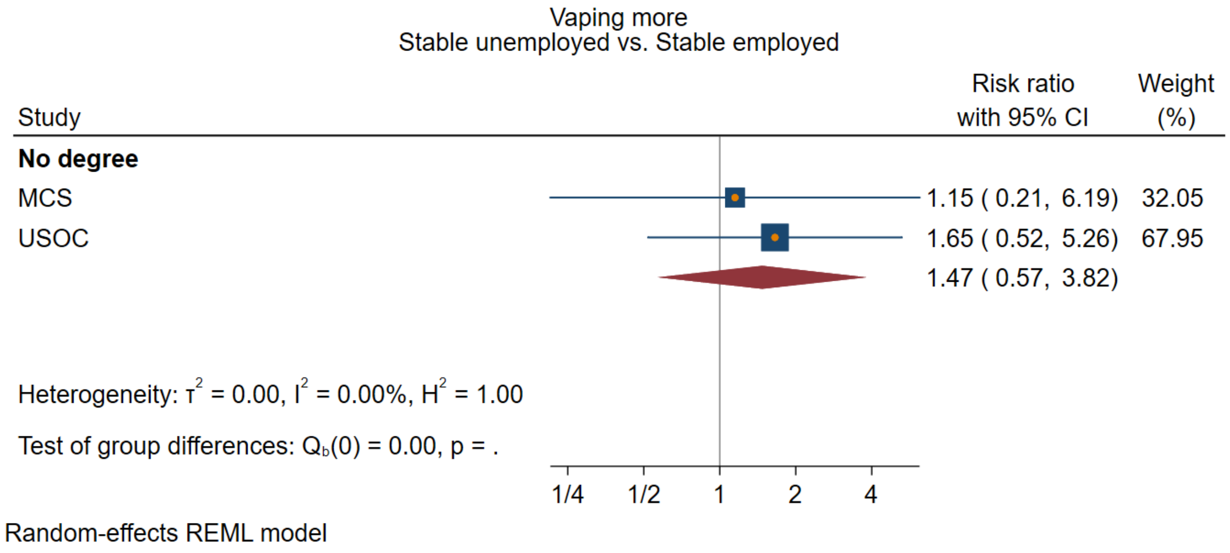
Vaping more
Stable unemployed vs. Stable employed
Study
Risk ratio with 95% CI
Weight (%)
No degree
MCS
1.15 (0.21, 6.19)
32.05
USOC
1.65 (0.52, 5.26)
67.95
1.47 (0.57, 3.82)
Heterogeneity: τ² = 0.00, I² = 0.00%, H² = 1.00
Test of group differences: Q_b(0) = 0.00, p = .
1/4
1/2
1
2
4
Random-effects REML model

Figure set 30: Vaping less

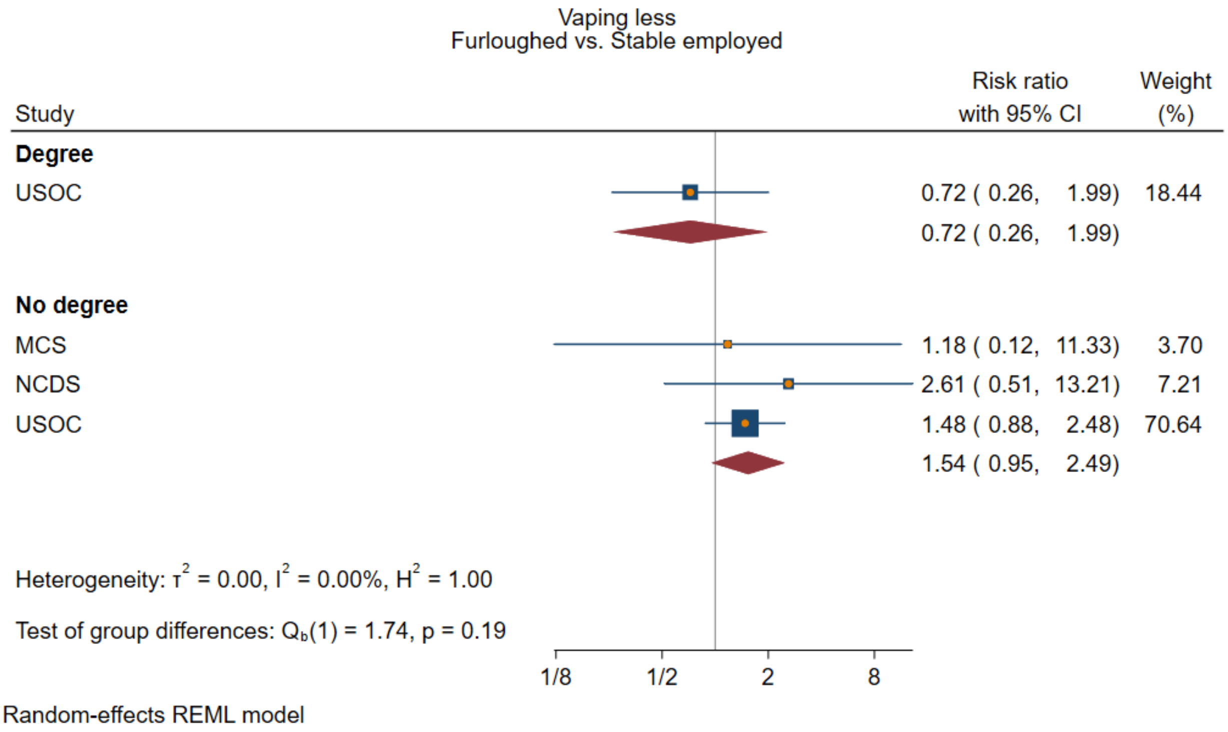

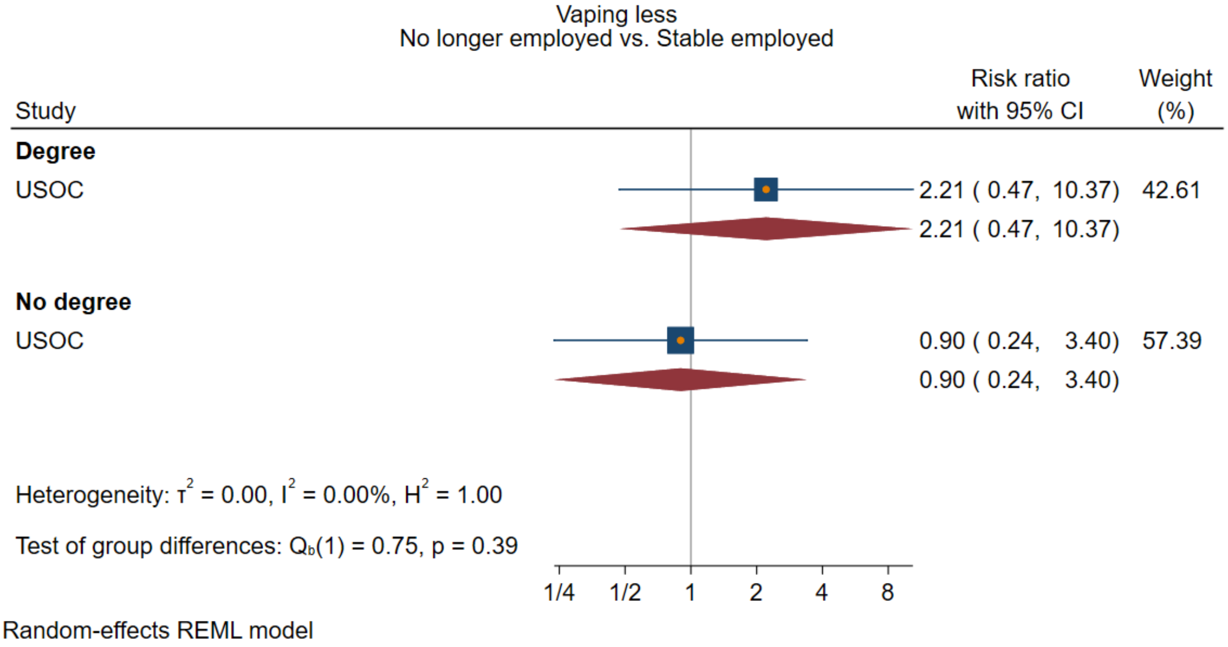

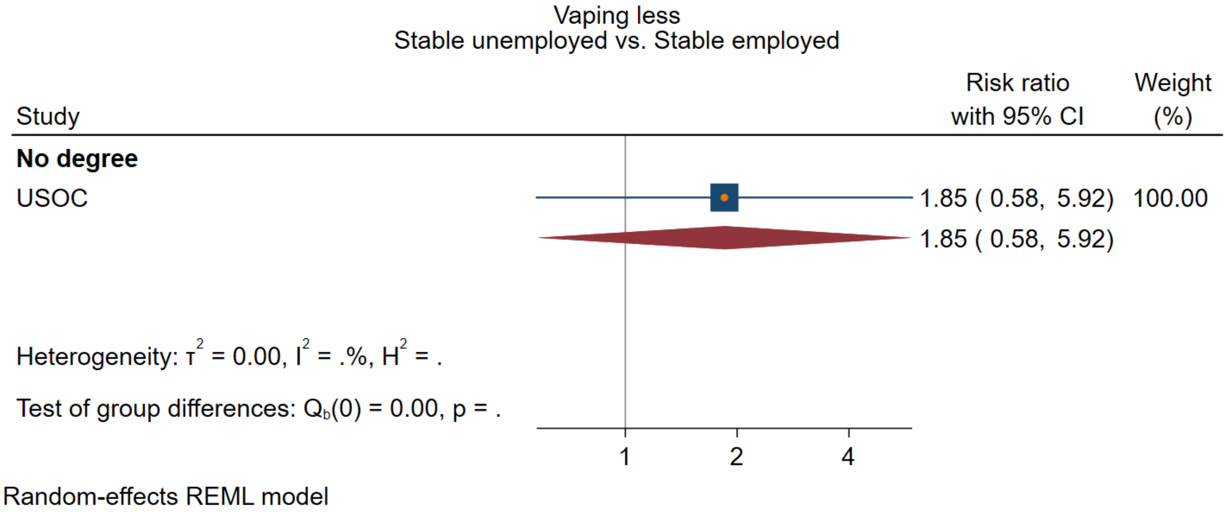

Figure set 31: Currently drinks 4+ days/week or 5+ drinks/occasion

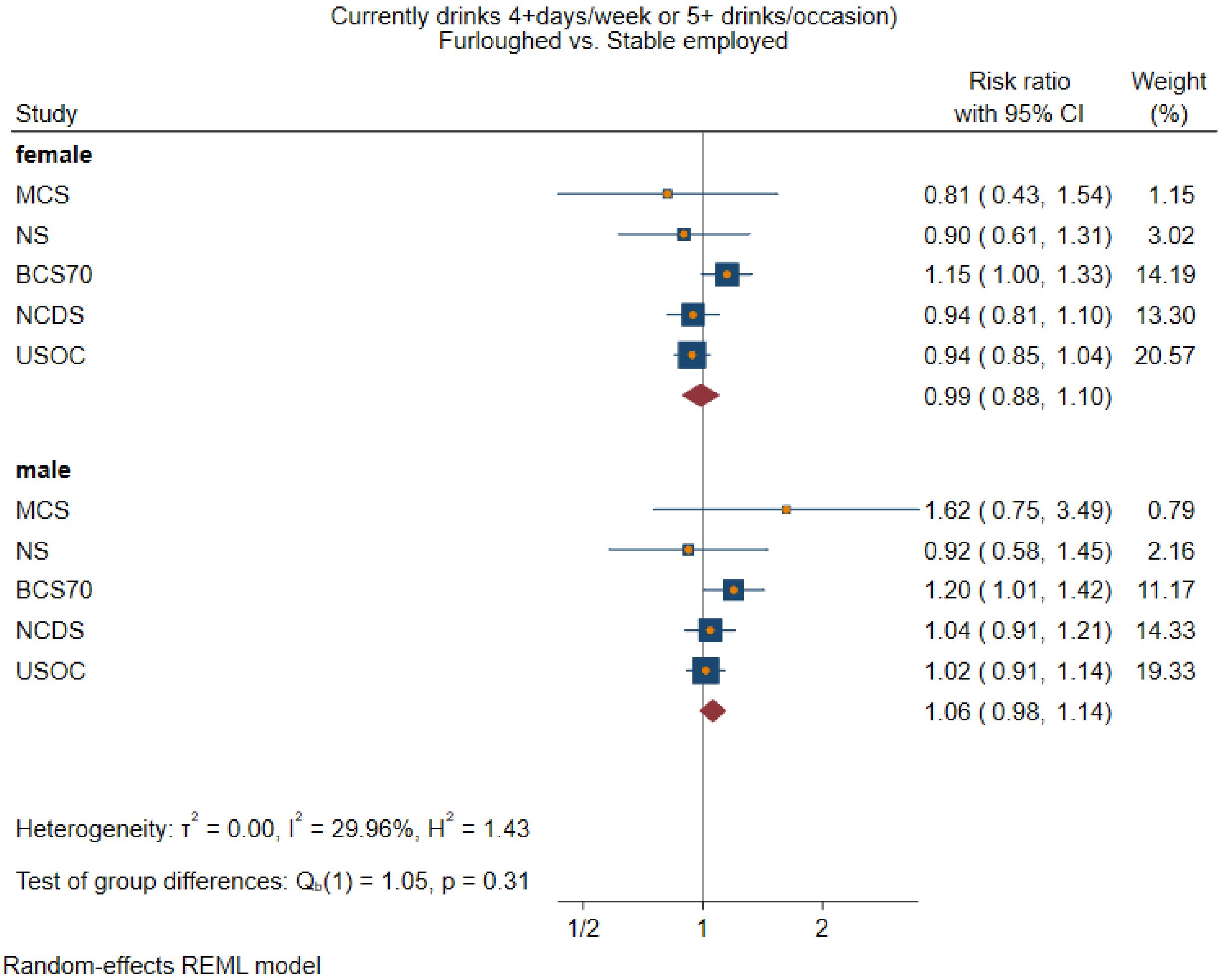
Currently drinks 4+days/week or 5+ drinks/occasion)
Furloughed vs. Stable employed
Study
Risk ratio with 95% CI
Weight (%)
female
MCS 0.81 (0.43, 1.54) 1.15
NS 0.90 (0.61, 1.31) 3.02
BCS70 1.15 (1.00, 1.33) 14.19
NCDS 0.94 (0.81, 1.10) 13.30
USOC 0.94 (0.85, 1.04) 20.57
0.99 (0.88, 1.10)
male
MCS 1.62 (0.75, 3.49) 0.79
NS 0.92 (0.58, 1.45) 2.16
BCS70 1.20 (1.01, 1.42) 11.17
NCDS 1.04 (0.91, 1.21) 14.33
USOC 1.02 (0.91, 1.14) 19.33
1.06 (0.98, 1.14)
Heterogeneity: τ^2 = 0.00, I^2 = 29.96%, H^2 = 1.43
Test of group differences: $Q_b(1)$ = 1.05, p = 0.31
1/2
1
2
Random-effects REML model

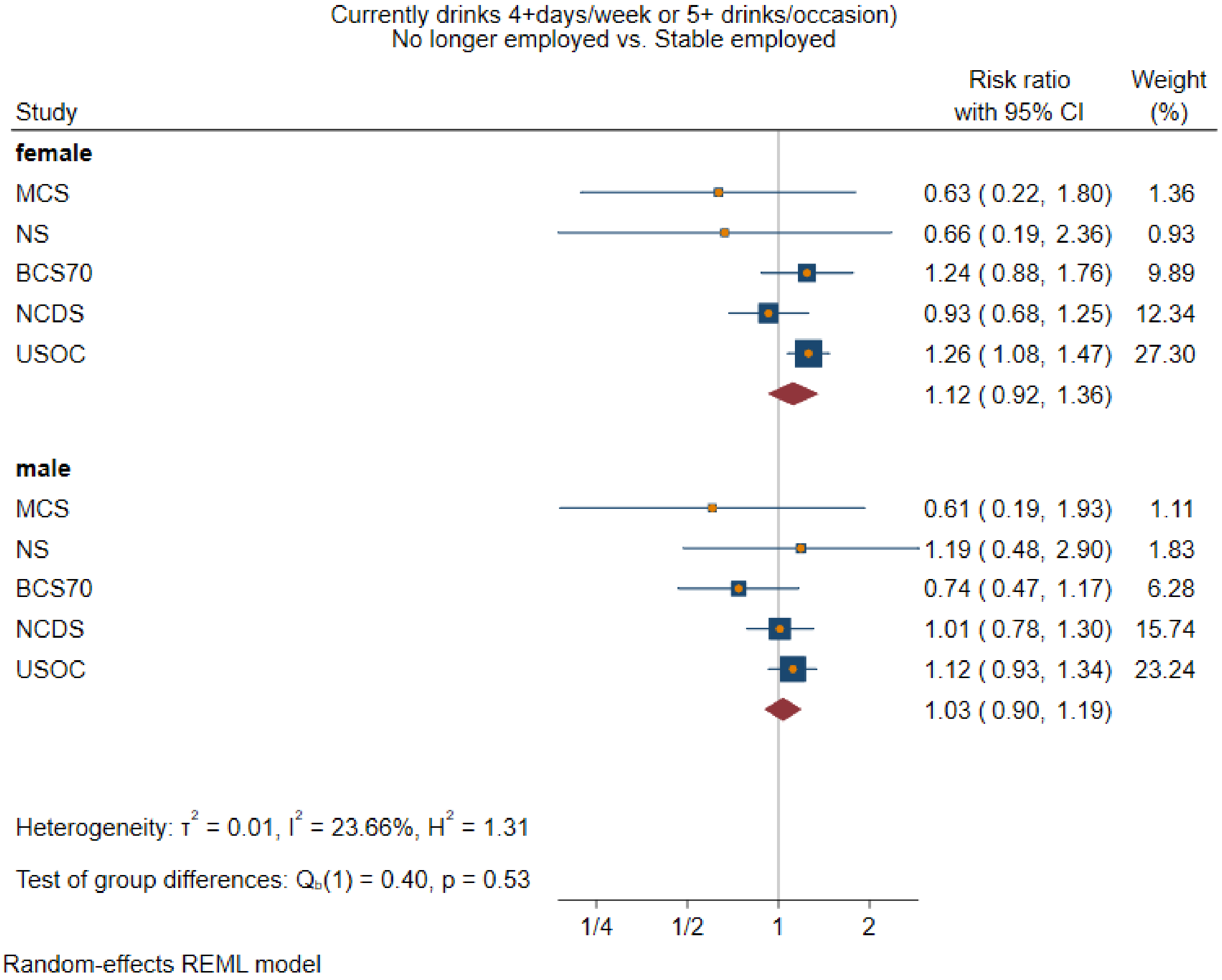
Currently drinks 4+days/week or 5+ drinks/occasion)
No longer employed vs. Stable employed
Study
Risk ratio with 95% CI
Weight (%)
female
MCS 0.63 (0.22, 1.80) 1.36
NS 0.66 (0.19, 2.36) 0.93
BCS70 1.24 (0.88, 1.76) 9.89
NCDS 0.93 (0.68, 1.25) 12.34
USOC 1.26 (1.08, 1.47) 27.30
1.12 (0.92, 1.36)
male
MCS 0.61 (0.19, 1.93) 1.11
NS 1.19 (0.48, 2.90) 1.83
BCS70 0.74 (0.47, 1.17) 6.28
NCDS 1.01 (0.78, 1.30) 15.74
USOC 1.12 (0.93, 1.34) 23.24
1.03 (0.90, 1.19)
Heterogeneity: τ^2 = 0.01, I^2 = 23.66%, H^2 = 1.31
Test of group differences: $Q_b(1)$ = 0.40, p = 0.53
1/4
1/2
1
2
Random-effects REML model

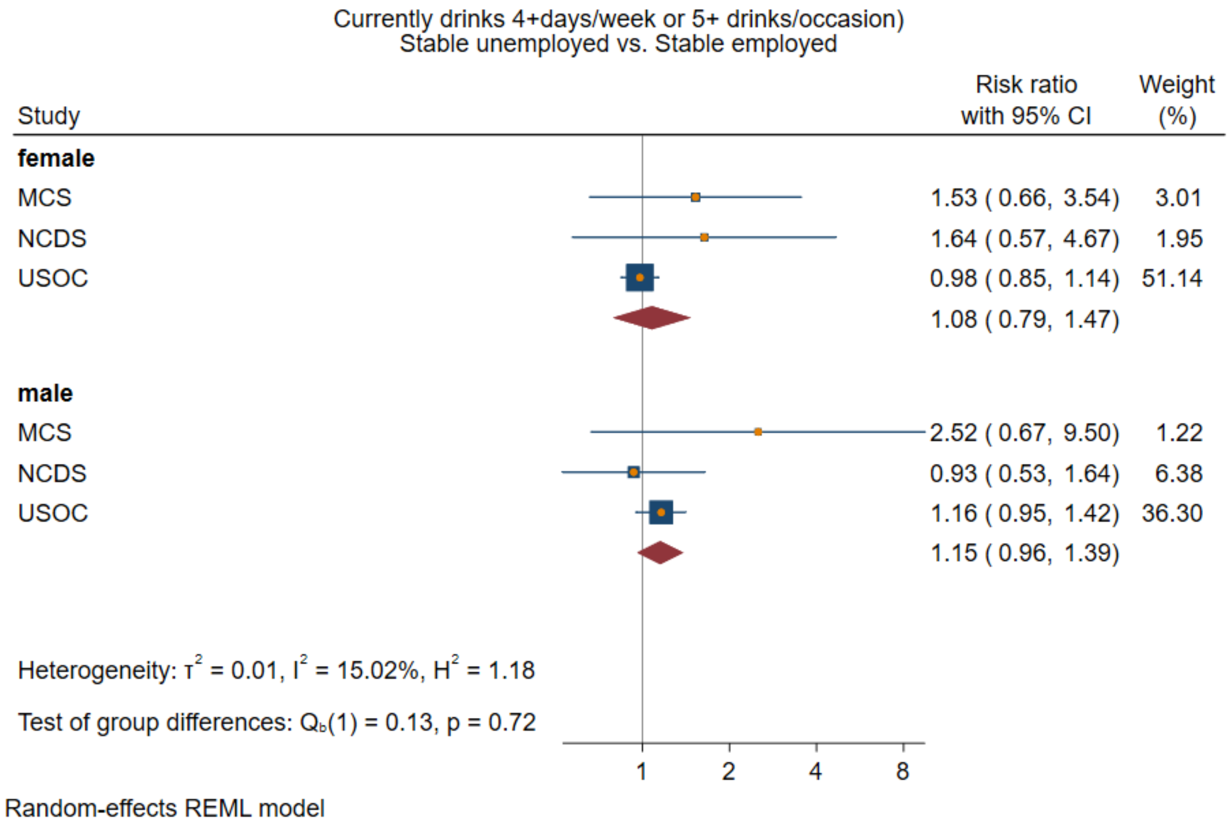
Currently drinks 4+days/week or 5+ drinks/occasion)
Stable unemployed vs. Stable employed
Study
Risk ratio with 95% CI
Weight (%)
female
MCS 1.53 (0.66, 3.54) 3.01
NCDS 1.64 (0.57, 4.67) 1.95
USOC 0.98 (0.85, 1.14) 51.14
1.08 (0.79, 1.47)
male
MCS 2.52 (0.67, 9.50) 1.22
NCDS 0.93 (0.53, 1.64) 6.38
USOC 1.16 (0.95, 1.42) 36.30
1.15 (0.96, 1.39)
Heterogeneity: τ² = 0.01, I² = 15.02%, H² = 1.18
Test of group differences: Qb(1) = 0.13, p = 0.72
1
2
4
8
Random-effects REML model

Figure set 32: Increased alcohol consumption

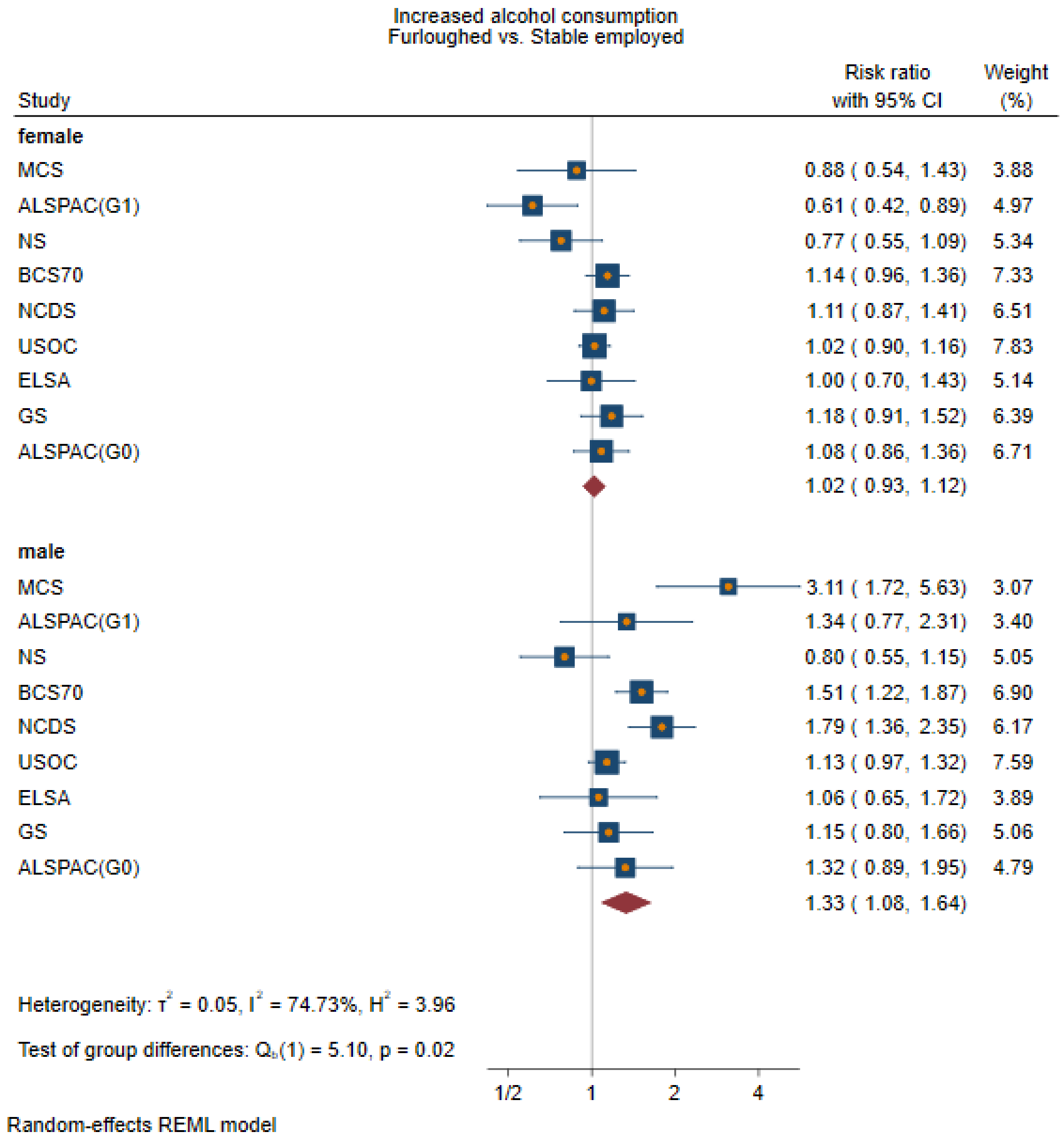

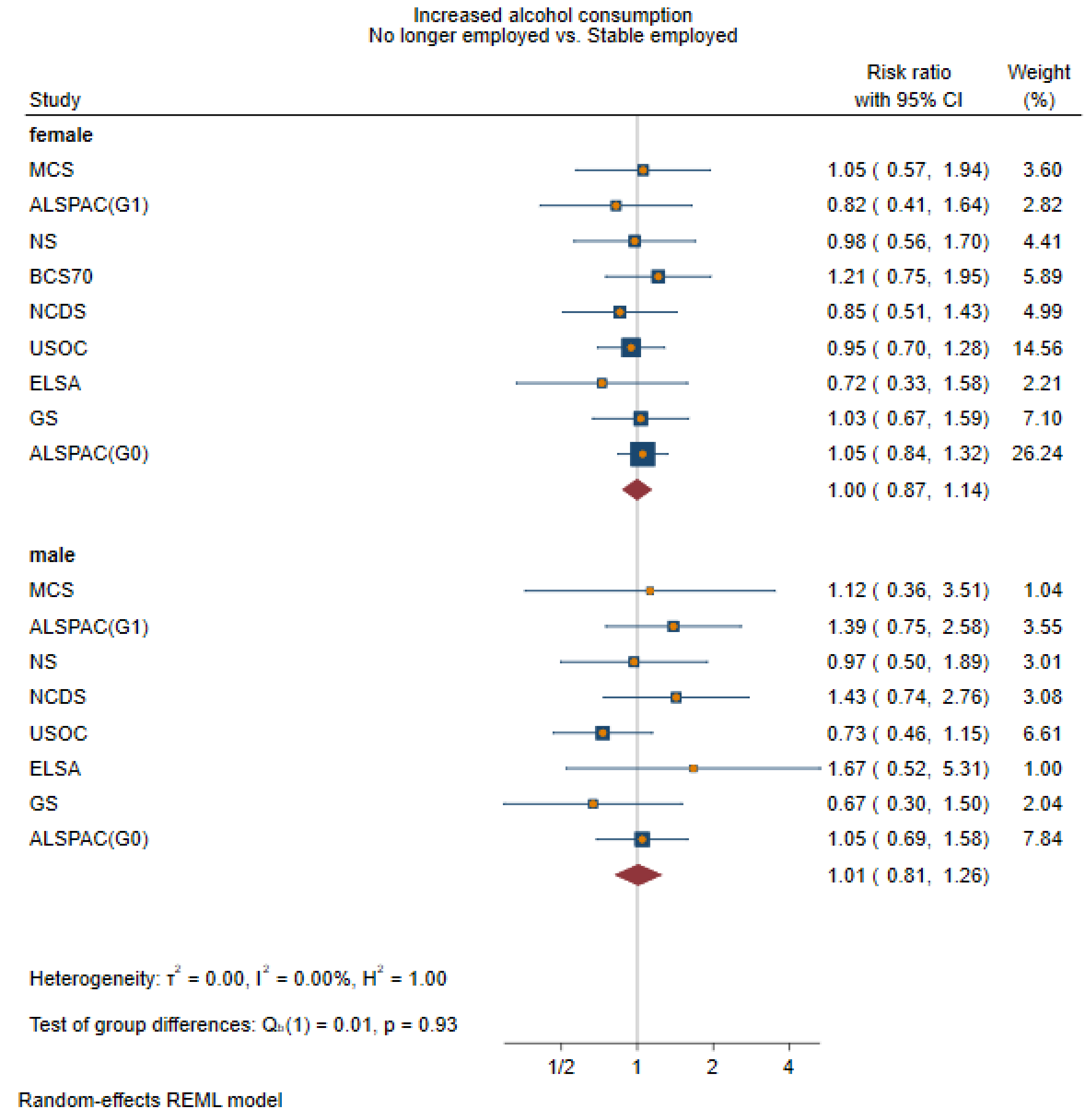
Increased alcohol consumption
No longer employed vs. Stable employed
Study
Risk ratio with 95% CI
Weight (%)
female
MCS 1.05 (0.57, 1.94) 3.60
ALSPAC(G1) 0.82 (0.41, 1.64) 2.82
NS 0.98 (0.56, 1.70) 4.41
BCS70 1.21 (0.75, 1.95) 5.89
NCDS 0.85 (0.51, 1.43) 4.99
USOC 0.95 (0.70, 1.28) 14.56
ELSA 0.72 (0.33, 1.58) 2.21
GS 1.03 (0.67, 1.59) 7.10
ALSPAC(G0) 1.05 (0.84, 1.32) 26.24
1.00 (0.87, 1.14)
male
MCS 1.12 (0.36, 3.51) 1.04
ALSPAC(G1) 1.39 (0.75, 2.58) 3.55
NS 0.97 (0.50, 1.89) 3.01
NCDS 1.43 (0.74, 2.76) 3.08
USOC 0.73 (0.46, 1.15) 6.61
ELSA 1.67 (0.52, 5.31) 1.00
GS 0.67 (0.30, 1.50) 2.04
ALSPAC(G0) 1.05 (0.69, 1.58) 7.84
1.01 (0.81, 1.26)
Heterogeneity: τ² = 0.00, I² = 0.00%, H² = 1.00
Test of group differences: Qb(1) = 0.01, p = 0.93
1/2 1 2 4
Random-effects REML model

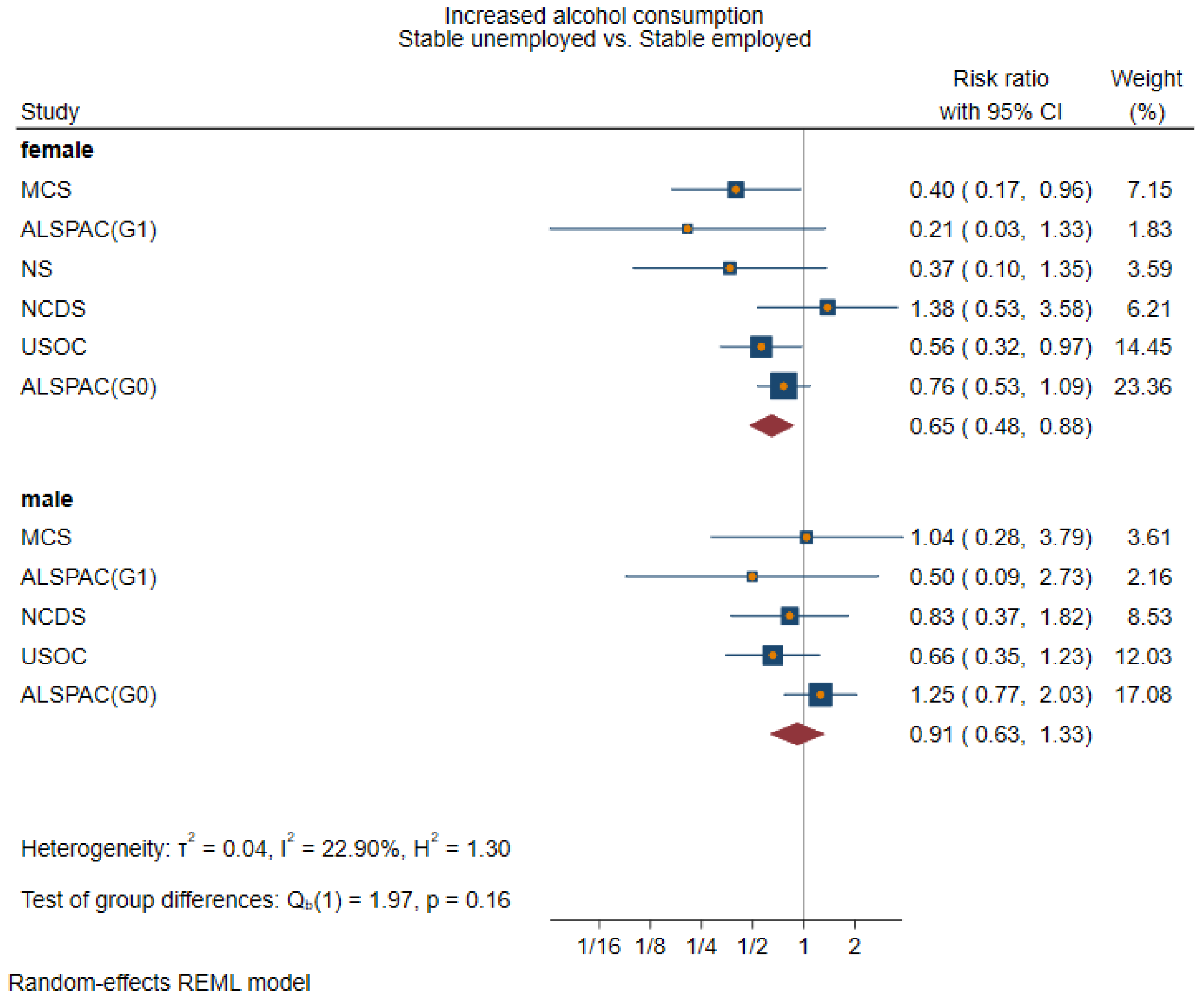
Increased alcohol consumption
Stable unemployed vs. Stable employed
Study
Risk ratio with 95% CI
Weight (%)
female
MCS 0.40 (0.17, 0.96) 7.15
ALSPAC(G1) 0.21 (0.03, 1.33) 1.83
NS 0.37 (0.10, 1.35) 3.59
NCDS 1.38 (0.53, 3.58) 6.21
USOC 0.56 (0.32, 0.97) 14.45
ALSPAC(G0) 0.76 (0.53, 1.09) 23.36
0.65 (0.48, 0.88)
male
MCS 1.04 (0.28, 3.79) 3.61
ALSPAC(G1) 0.50 (0.09, 2.73) 2.16
NCDS 0.83 (0.37, 1.82) 8.53
USOC 0.66 (0.35, 1.23) 12.03
ALSPAC(G0) 1.25 (0.77, 2.03) 17.08
0.91 (0.63, 1.33)
Heterogeneity: τ² = 0.04, I² = 22.90%, H² = 1.30
Test of group differences: Qb(1) = 1.97, p = 0.16
1/16 1/8 1/4 1/2 1 2
Random-effects REML model

Figure set 33: Reduced alcohol consumption

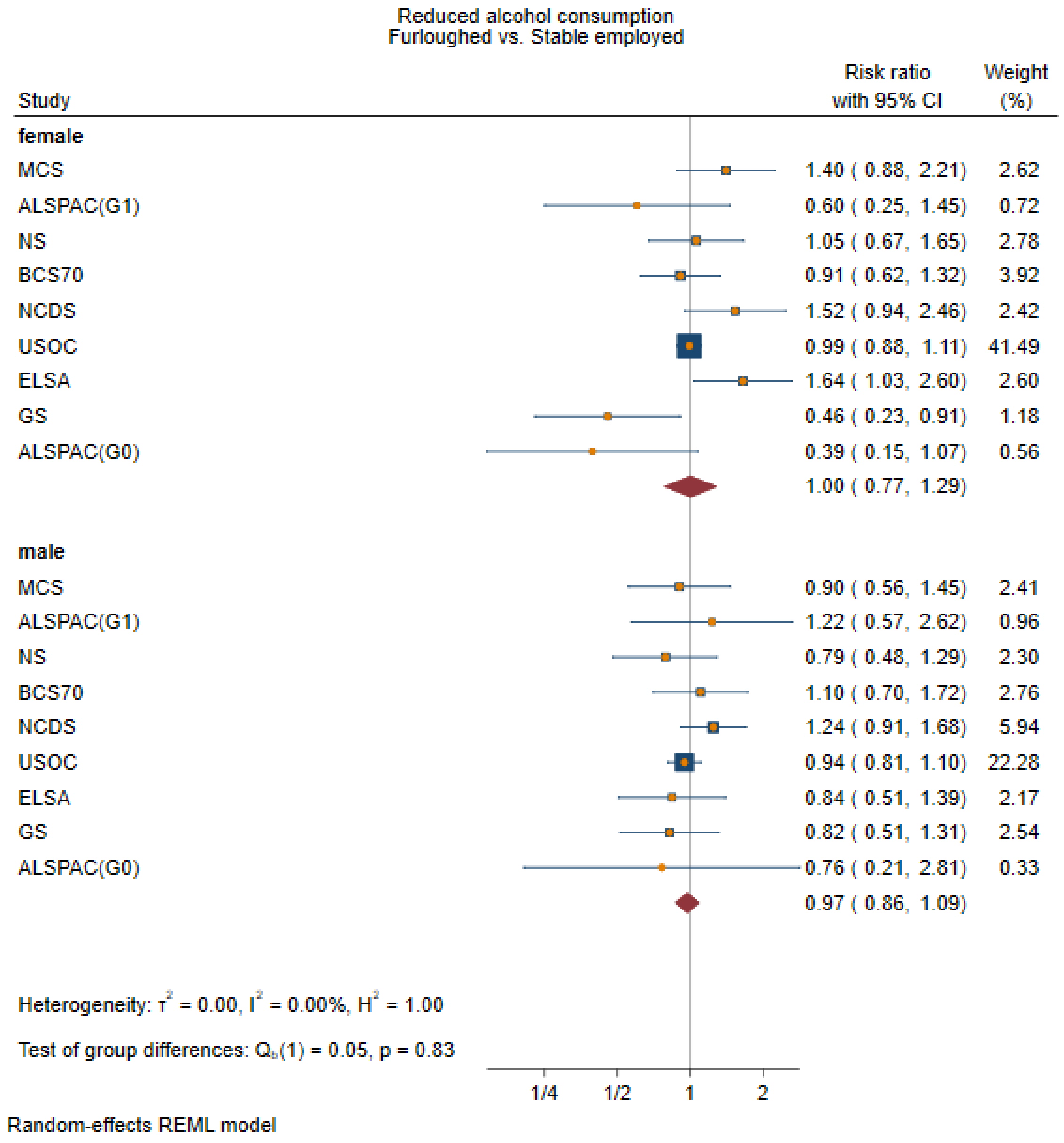

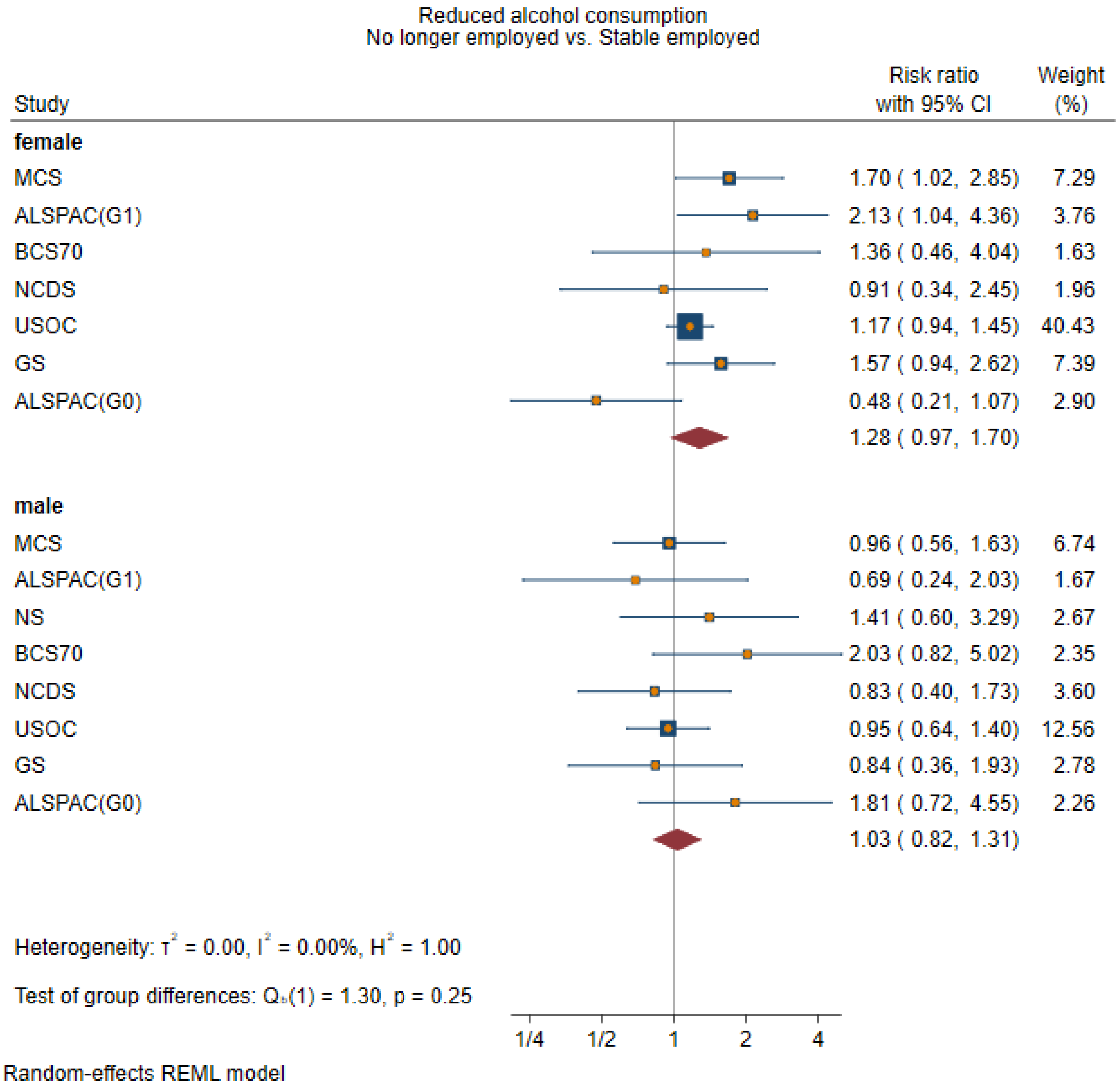
Reduced alcohol consumption
No longer employed vs. Stable employed
Study
Risk ratio with 95% CI
Weight (%)
female
MCS 1.70 (1.02, 2.85) 7.29
ALSPAC(G1) 2.13 (1.04, 4.36) 3.76
BCS70 1.36 (0.46, 4.04) 1.63
NCDS 0.91 (0.34, 2.45) 1.96
USOC 1.17 (0.94, 1.45) 40.43
GS 1.57 (0.94, 2.62) 7.39
ALSPAC(G0) 0.48 (0.21, 1.07) 2.90
1.28 (0.97, 1.70)
male
MCS 0.96 (0.56, 1.63) 6.74
ALSPAC(G1) 0.69 (0.24, 2.03) 1.67
NS 1.41 (0.60, 3.29) 2.67
BCS70 2.03 (0.82, 5.02) 2.35
NCDS 0.83 (0.40, 1.73) 3.60
USOC 0.95 (0.64, 1.40) 12.56
GS 0.84 (0.36, 1.93) 2.78
ALSPAC(G0) 1.81 (0.72, 4.55) 2.26
1.03 (0.82, 1.31)
Heterogeneity: τ^2 = 0.00, I^2 = 0.00%, H^2 = 1.00
Test of group differences: $Q_b(1)$ = 1.30, p = 0.25
1/4 1/2 1 2 4
Random-effects REML model

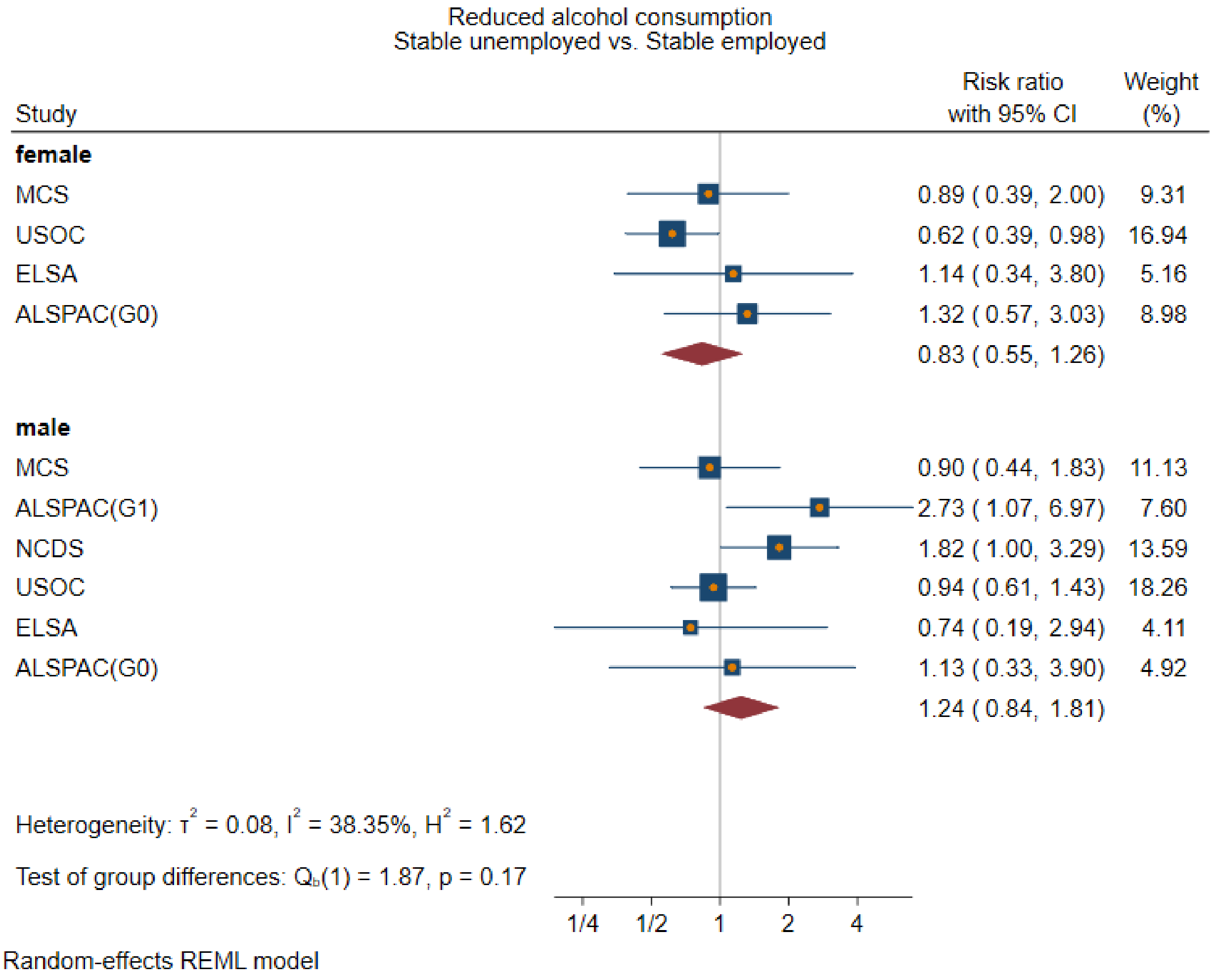
Reduced alcohol consumption
Stable unemployed vs. Stable employed
Study
Risk ratio with 95% CI
Weight (%)
female
MCS 0.89 (0.39, 2.00) 9.31
USOC 0.62 (0.39, 0.98) 16.94
ELSA 1.14 (0.34, 3.80) 5.16
ALSPAC(G0) 1.32 (0.57, 3.03) 8.98
0.83 (0.55, 1.26)
male
MCS 0.90 (0.44, 1.83) 11.13
ALSPAC(G1) 2.73 (1.07, 6.97) 7.60
NCDS 1.82 (1.00, 3.29) 13.59
USOC 0.94 (0.61, 1.43) 18.26
ELSA 0.74 (0.19, 2.94) 4.11
ALSPAC(G0) 1.13 (0.33, 3.90) 4.92
1.24 (0.84, 1.81)
Heterogeneity: τ^2 = 0.08, I^2 = 38.35%, H^2 = 1.62
Test of group differences: $Q_b(1)$ = 1.87, p = 0.17
1/4 1/2 1 2 4
Random-effects REML model

Figure set 34: Currently drinks 5+ drinks/occasion

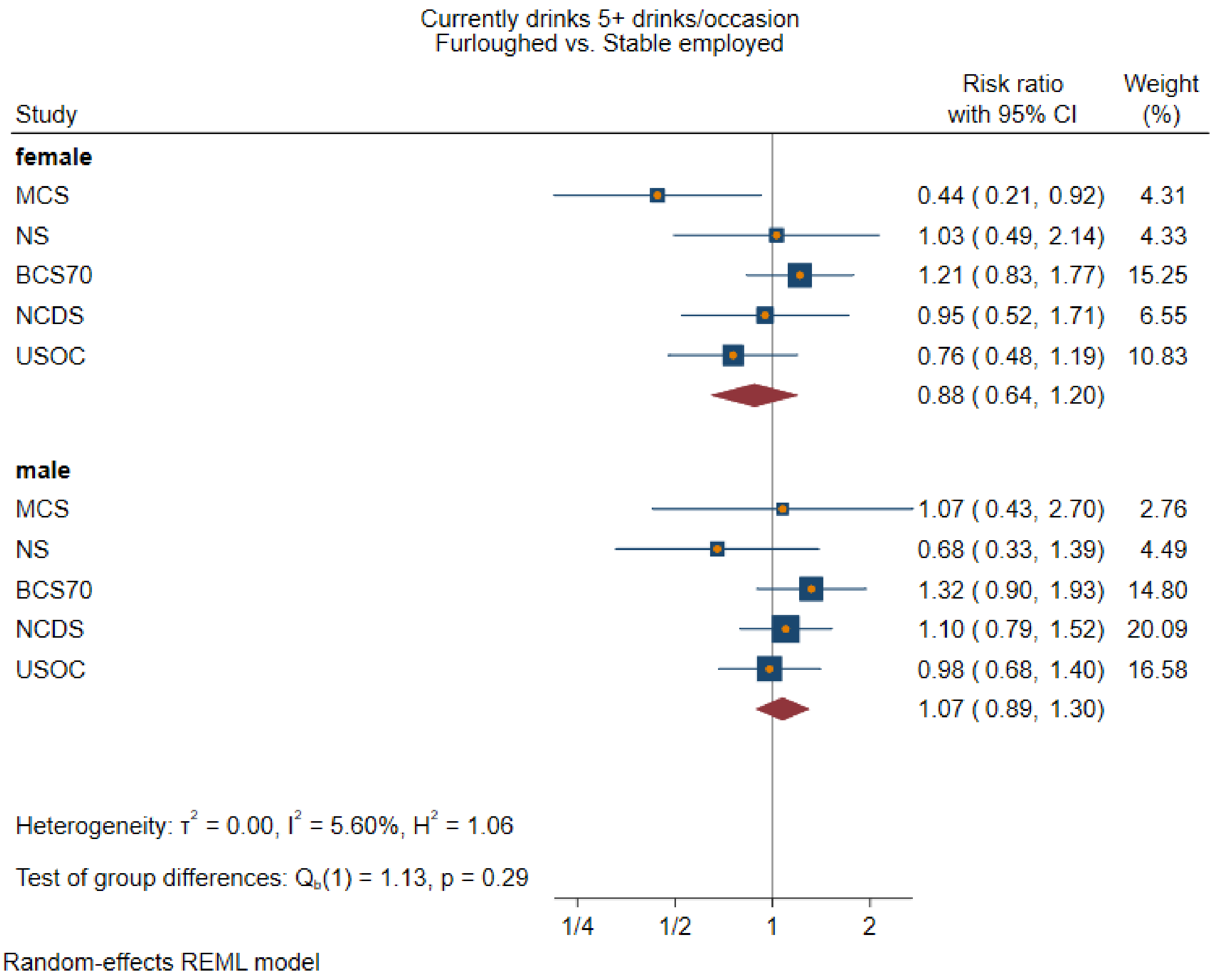

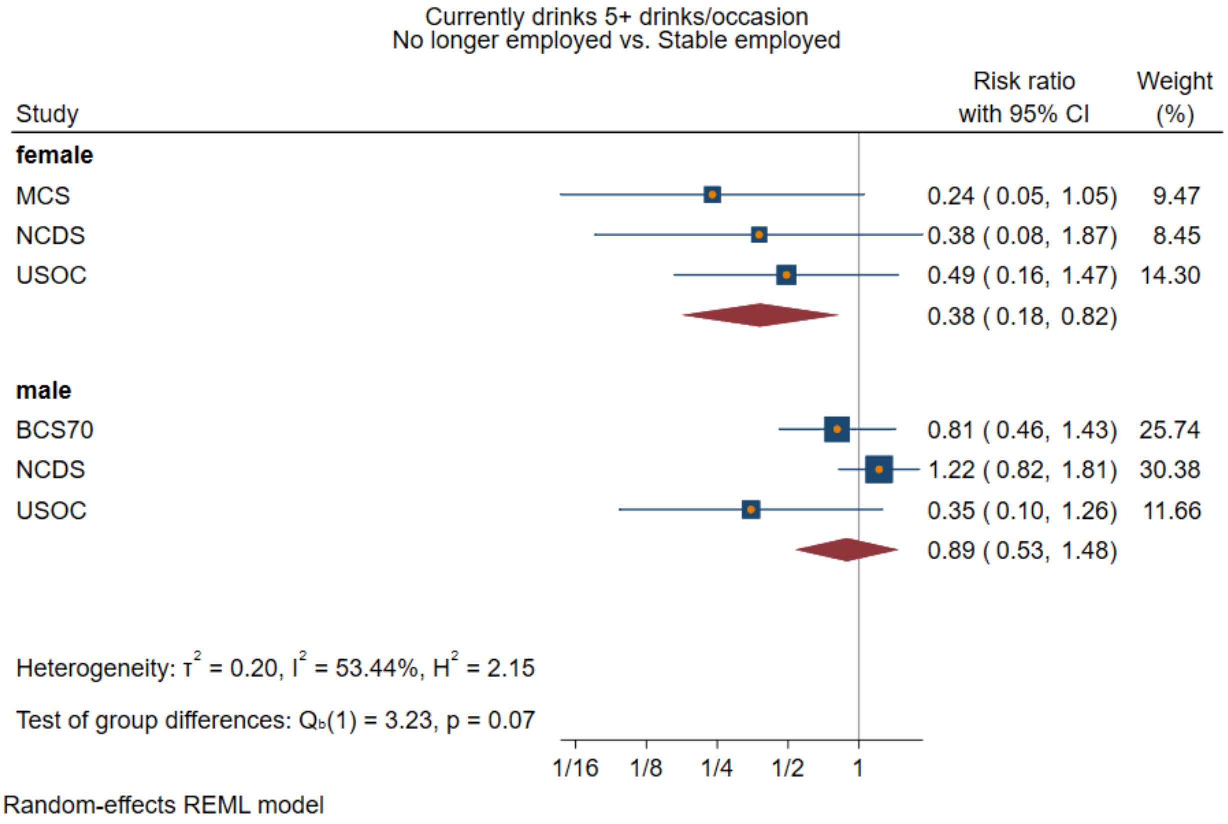

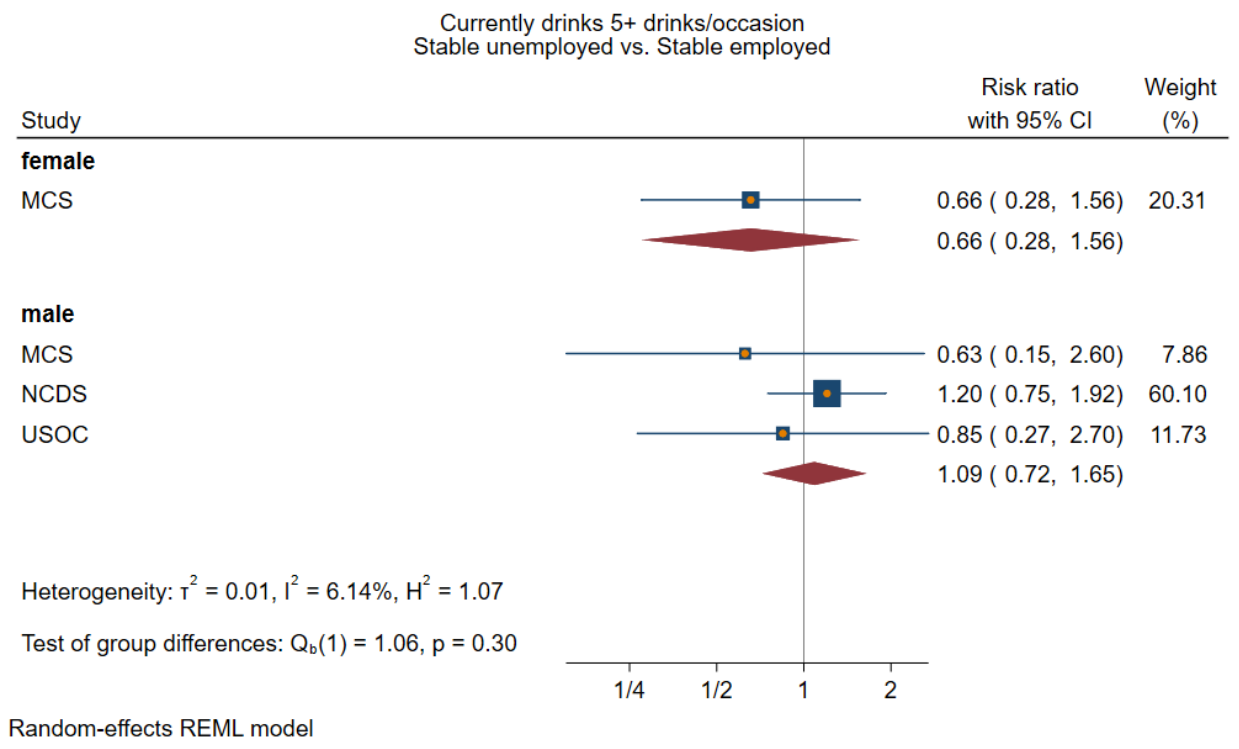
Currently drinks 5+ drinks/occasion
Stable unemployed vs. Stable employed
Study
Risk ratio with 95% CI
Weight (%)
female
MCS
0.66 (0.28, 1.56) 20.31
0.66 (0.28, 1.56)
male
MCS
0.63 (0.15, 2.60) 7.86
NCDS
1.20 (0.75, 1.92) 60.10
USOC
0.85 (0.27, 2.70) 11.73
1.09 (0.72, 1.65)
Heterogeneity: τ² = 0.01, I² = 6.14%, H² = 1.07
Test of group differences: Qb(1) = 1.06, p = 0.30
1/4
1/2
1
2
Random-effects REML model

Figure set 35: Drinks more alcohol units per occasion

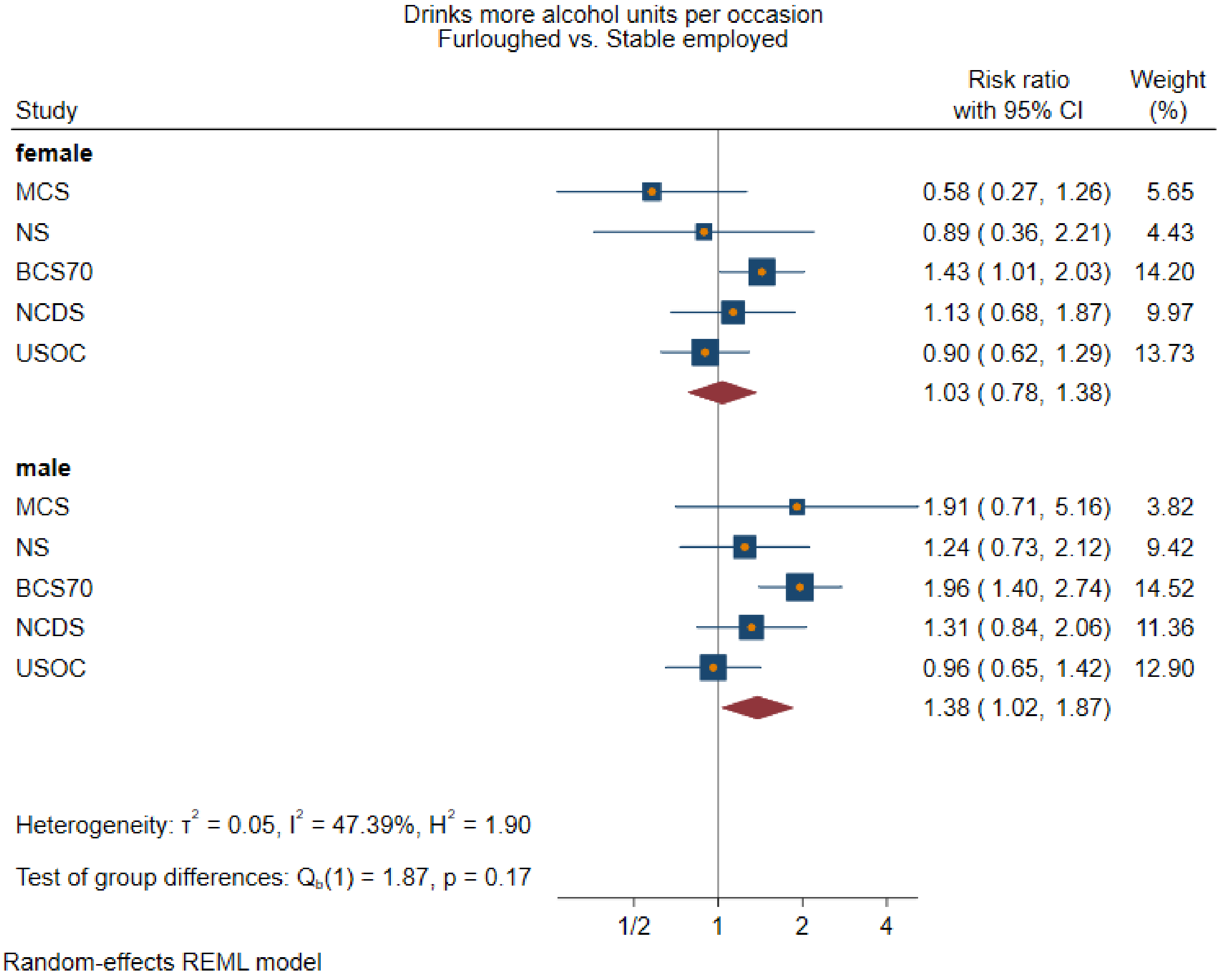

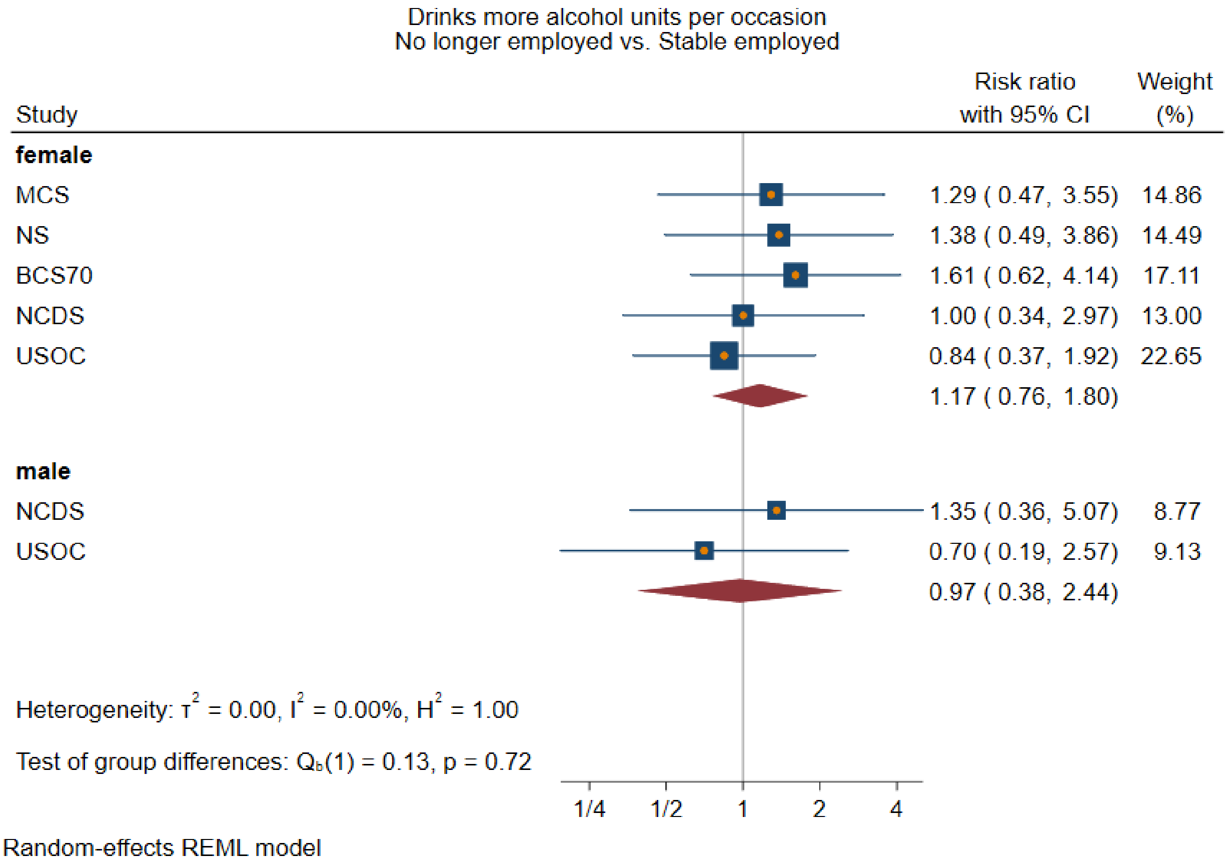

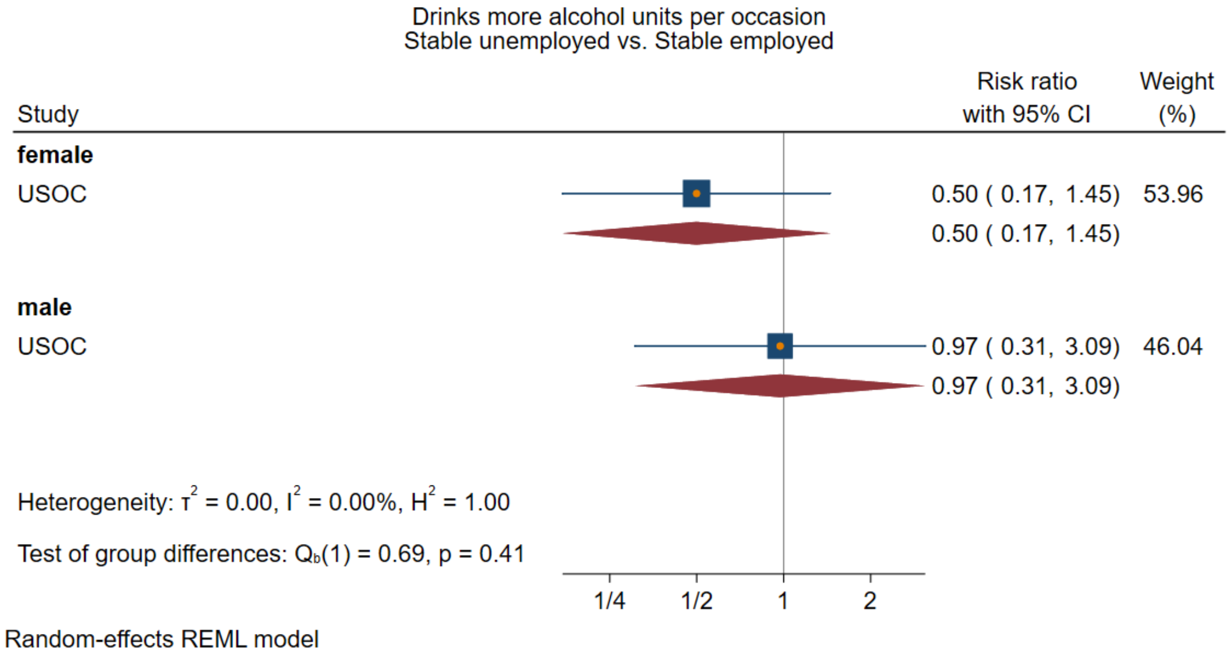
Drinks more alcohol units per occasion
Stable unemployed vs. Stable employed
Study
Risk ratio with 95% CI
Weight (%)
female
USOC
0.50 (0.17, 1.45)
53.96
0.50 (0.17, 1.45)
male
USOC
0.97 (0.31, 3.09)
46.04
0.97 (0.31, 3.09)
Heterogeneity: τ² = 0.00, I² = 0.00%, H² = 1.00
Test of group differences: Qb(1) = 0.69, p = 0.41
1/4
1/2
1
2
Random-effects REML model

Figure set 36: Drinks fewer alcohol units per occasion

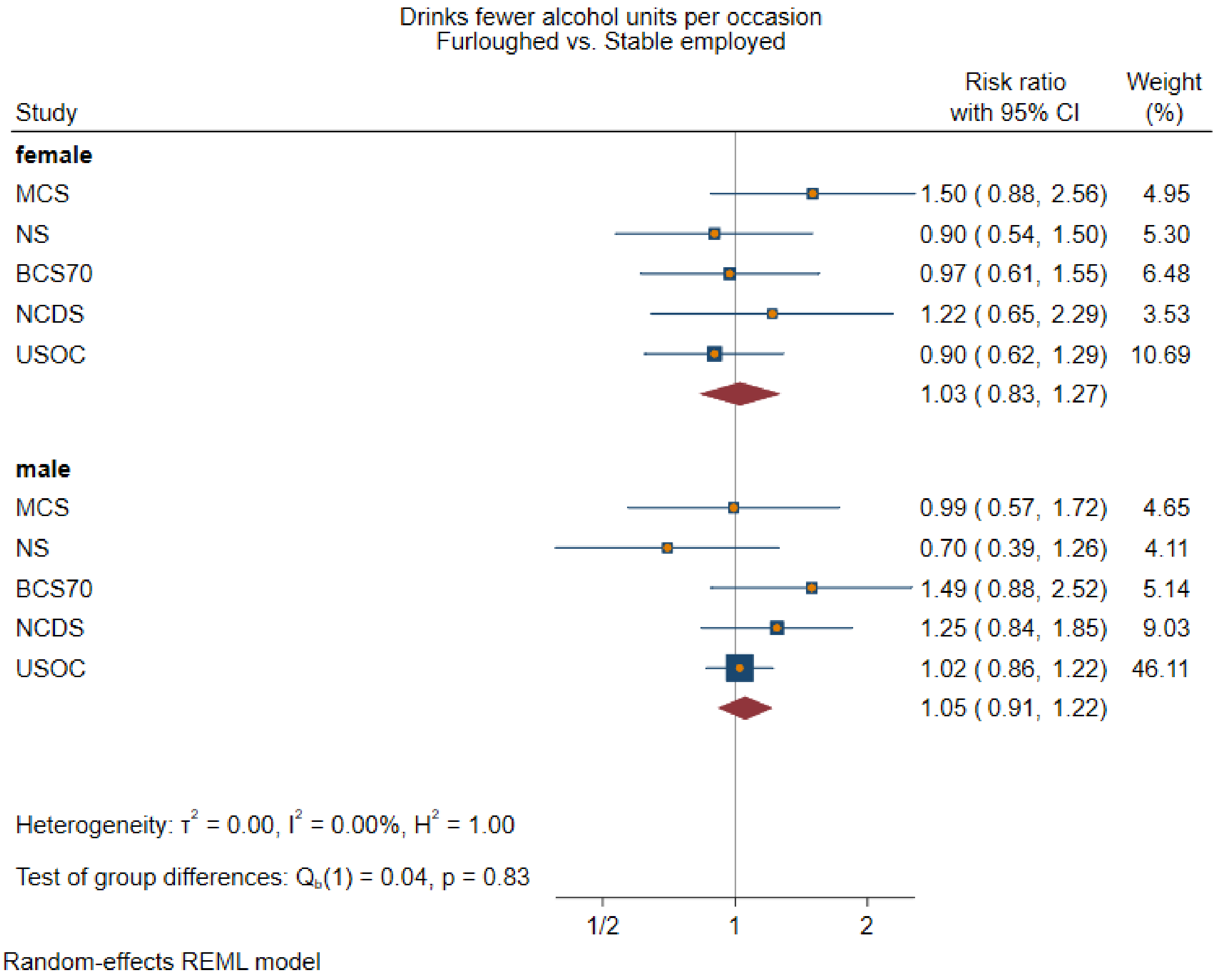

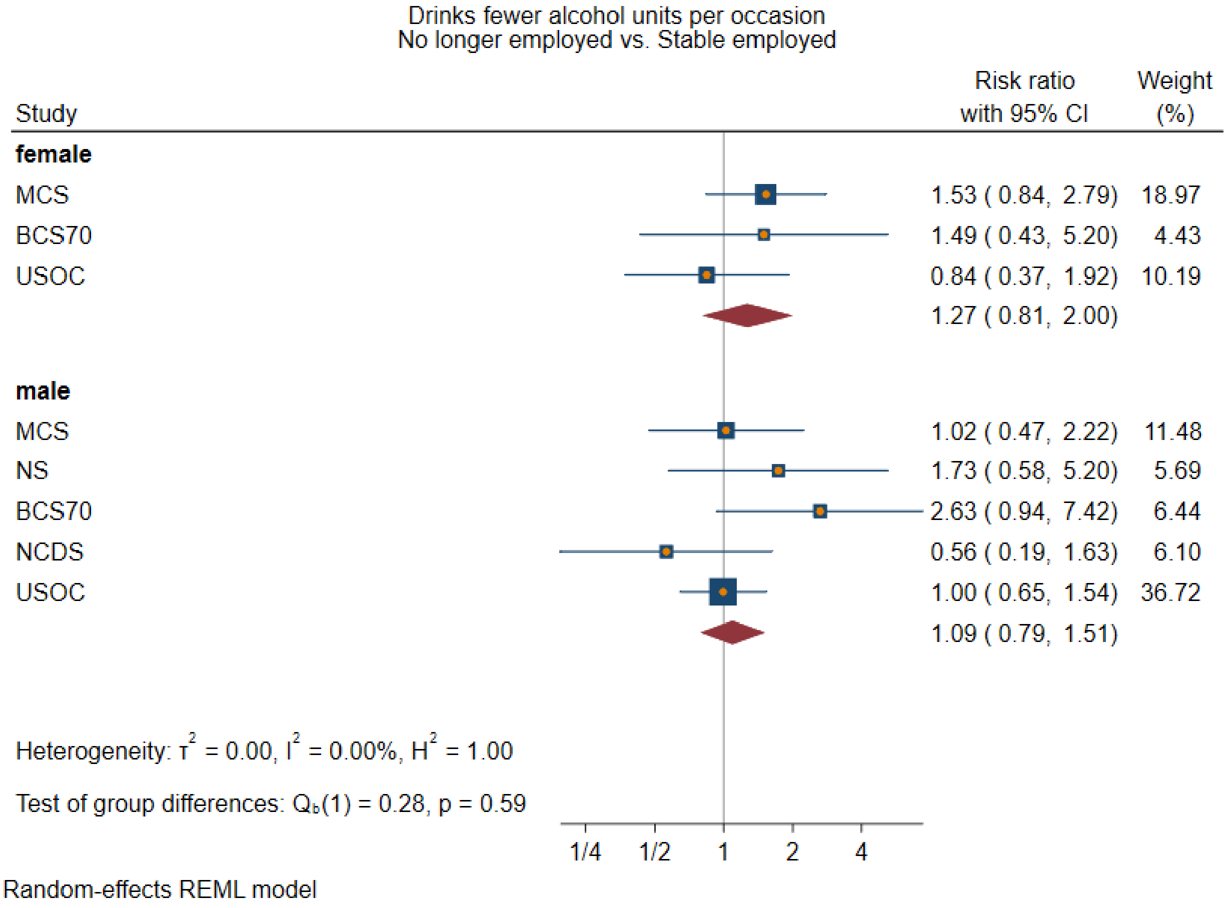

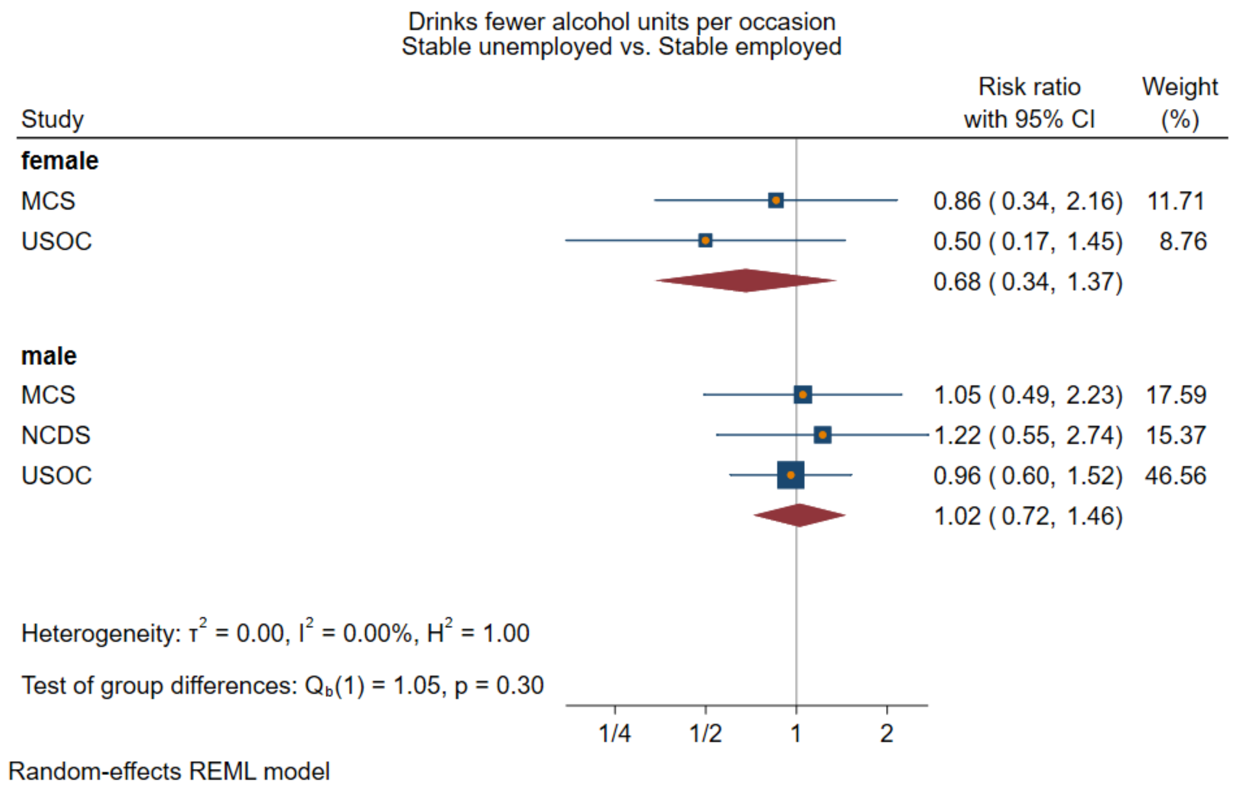

Drinks fewer alcohol units per occasion
Stable unemployed vs. Stable employed
Study
Risk ratio with 95% CI
Weight (%)
female
MCS
0.86 (0.34, 2.16) 11.71
USOC
0.50 (0.17, 1.45) 8.76
0.68 (0.34, 1.37)
male
MCS
1.05 (0.49, 2.23) 17.59
NCDS
1.22 (0.55, 2.74) 15.37
USOC
0.96 (0.60, 1.52) 46.56
1.02 (0.72, 1.46)
Heterogeneity: τ² = 0.00, I² = 0.00%, H² = 1.00
Test of group differences: Q_b(1) = 1.05, p = 0.30
1/4
1/2
1
2
Random-effects REML model

Figure set 37: Currently drinks 4+ days/week

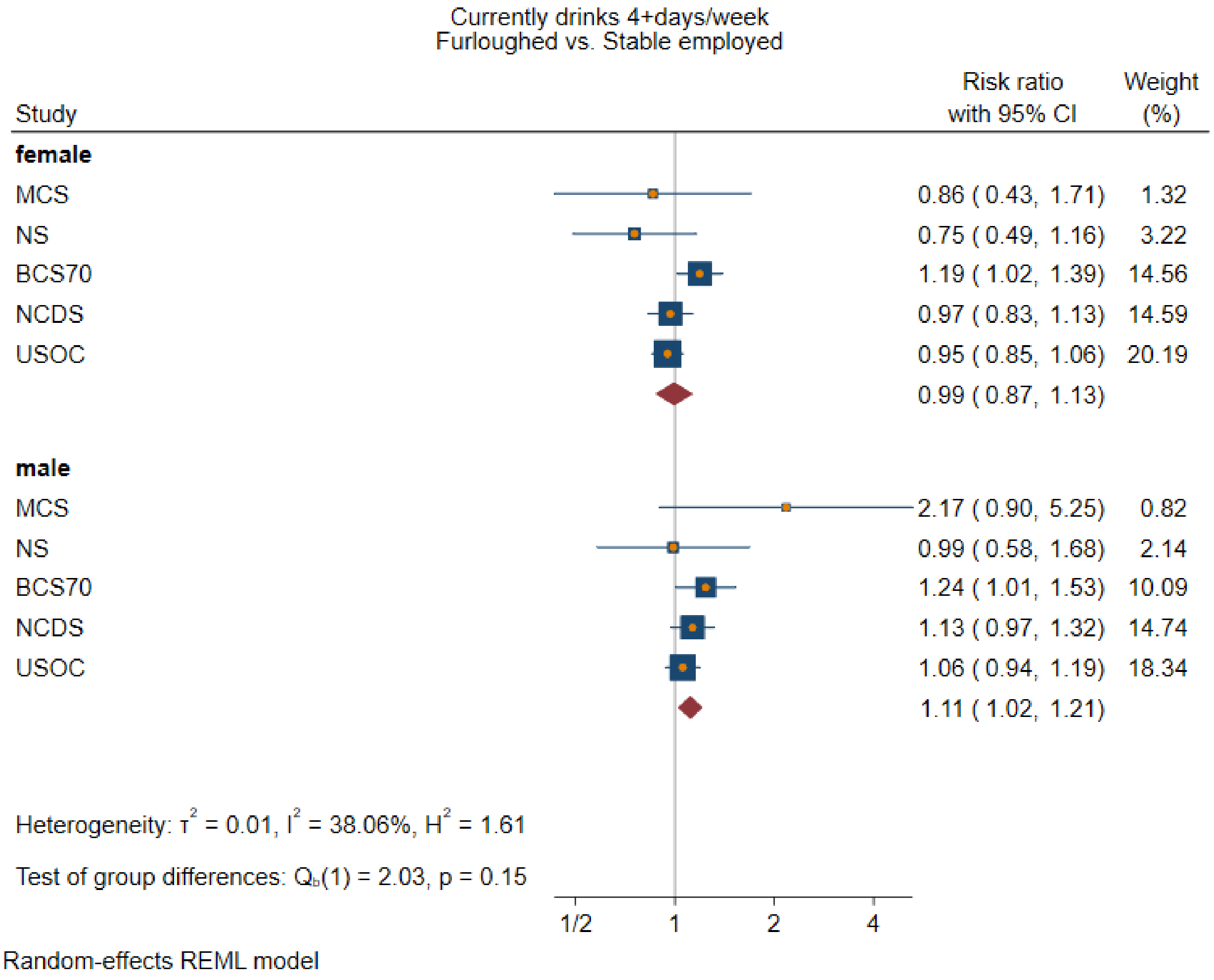

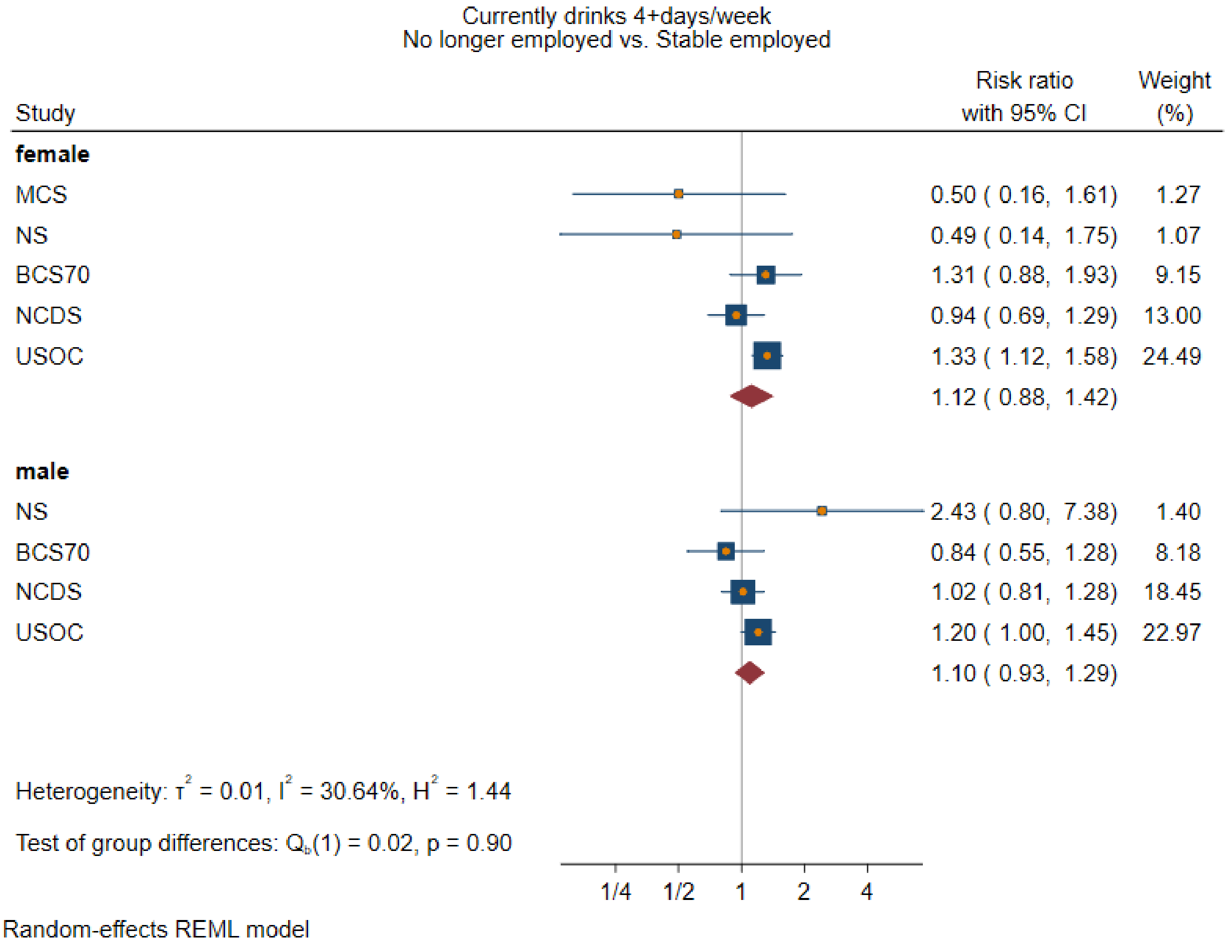

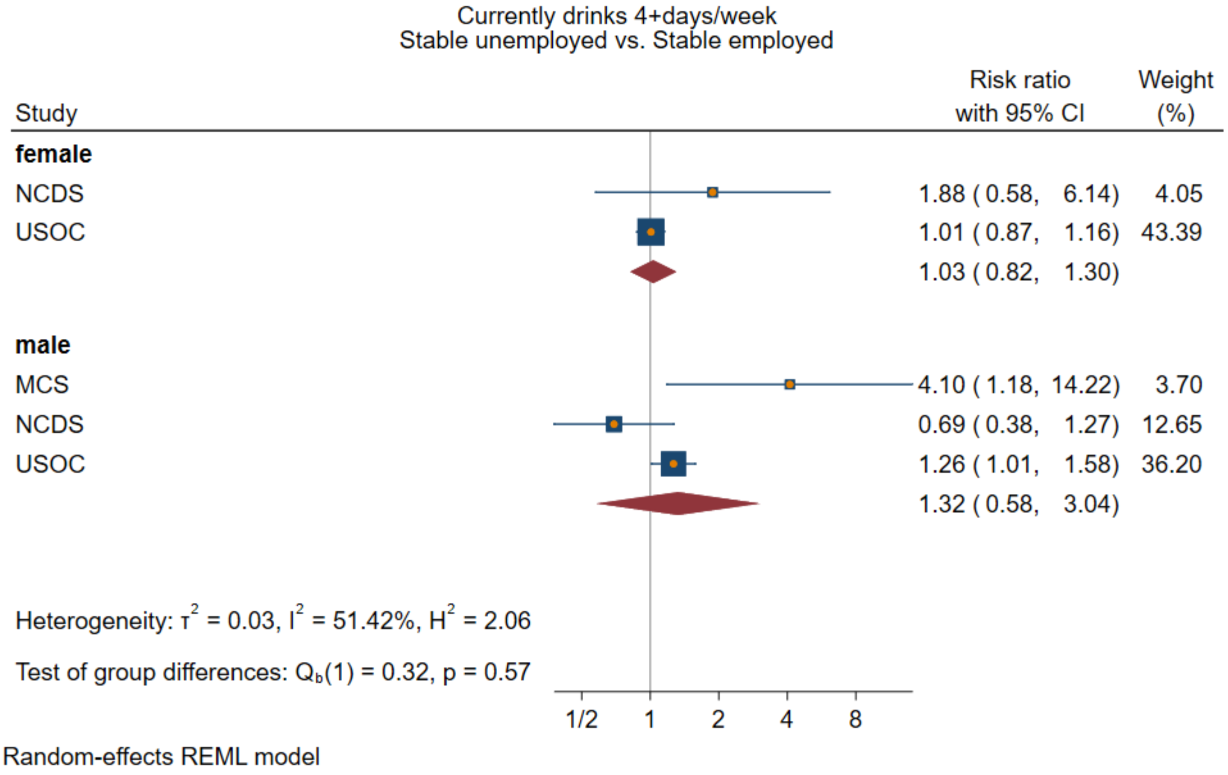

Currently drinks 4+days/week
Stable unemployed vs. Stable employed
Study
Risk ratio with 95% CI
Weight (%)
female
NCDS 1.88 (0.58, 6.14) 4.05
USOC 1.01 (0.87, 1.16) 43.39
1.03 (0.82, 1.30)
male
MCS 4.10 (1.18, 14.22) 3.70
NCDS 0.69 (0.38, 1.27) 12.65
USOC 1.26 (1.01, 1.58) 36.20
1.32 (0.58, 3.04)
Heterogeneity: τ2 = 0.03, I2 = 51.42%, H2 = 2.06
Test of group differences: Qb(1) = 0.32, p = 0.57
1/2
1
2
4
8
Random-effects REML model

Figure set 38: Drinks more frequently

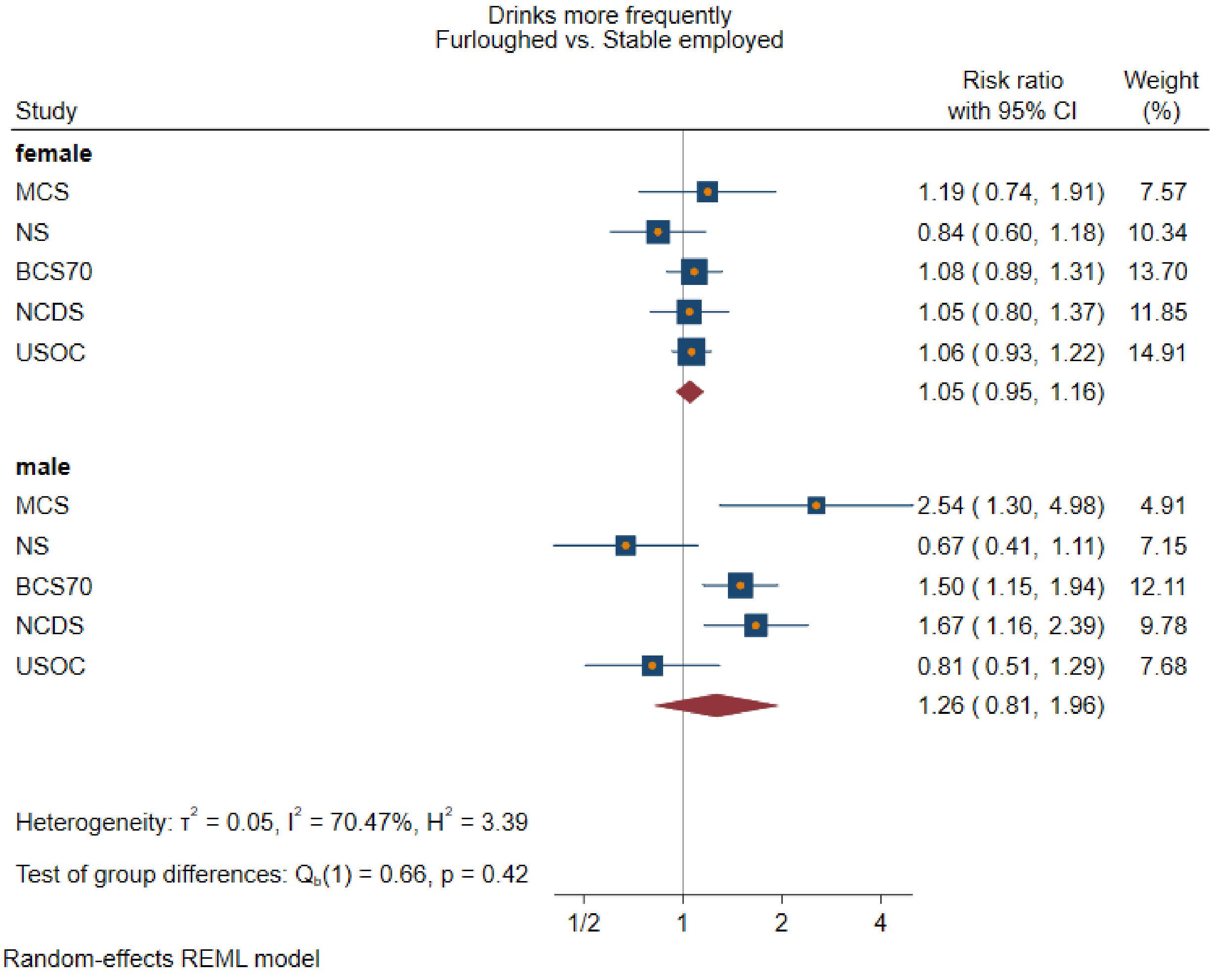

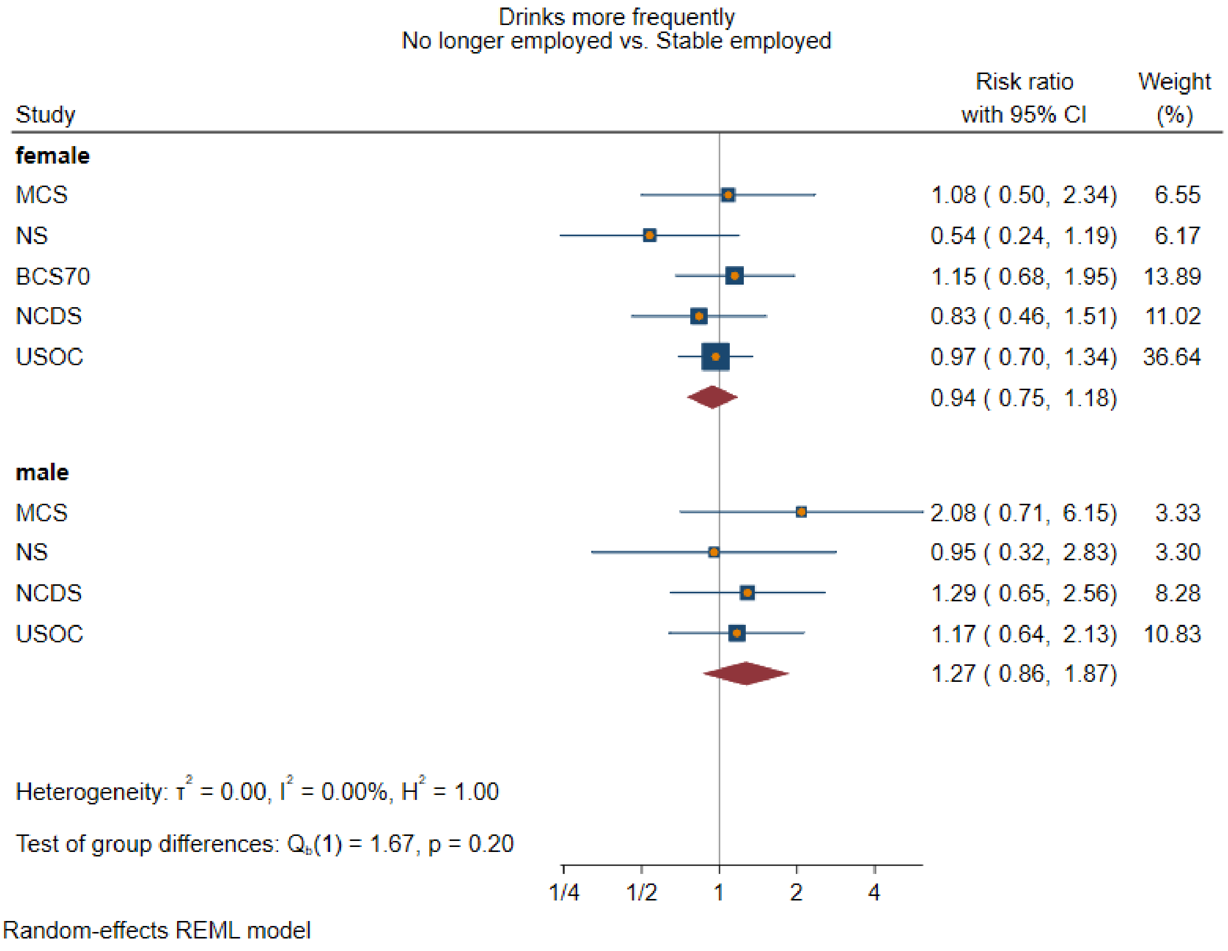

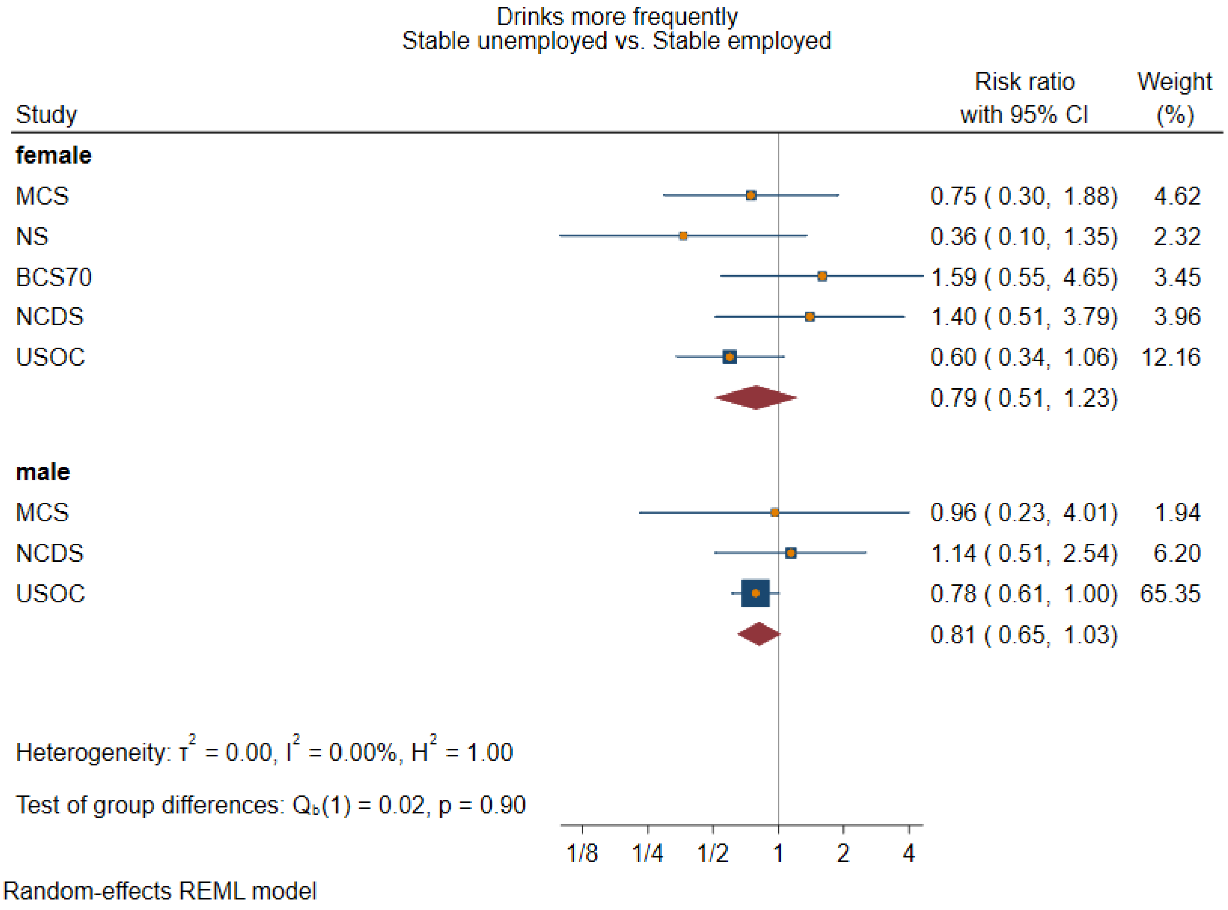
Drinks more frequently
Stable unemployed vs. Stable employed
Study
Risk ratio with 95% CI
Weight (%)
female
MCS 0.75 (0.30, 1.88) 4.62
NS 0.36 (0.10, 1.35) 2.32
BCS70 1.59 (0.55, 4.65) 3.45
NCDS 1.40 (0.51, 3.79) 3.96
USOC 0.60 (0.34, 1.06) 12.16
0.79 (0.51, 1.23)
male
MCS 0.96 (0.23, 4.01) 1.94
NCDS 1.14 (0.51, 2.54) 6.20
USOC 0.78 (0.61, 1.00) 65.35
0.81 (0.65, 1.03)
Heterogeneity: τ² = 0.00, I² = 0.00%, H² = 1.00
Test of group differences: Q_b(1) = 0.02, p = 0.90
1/8 1/4 1/2 1 2 4
Random-effects REML model

Figure set 39: Drinks less frequently

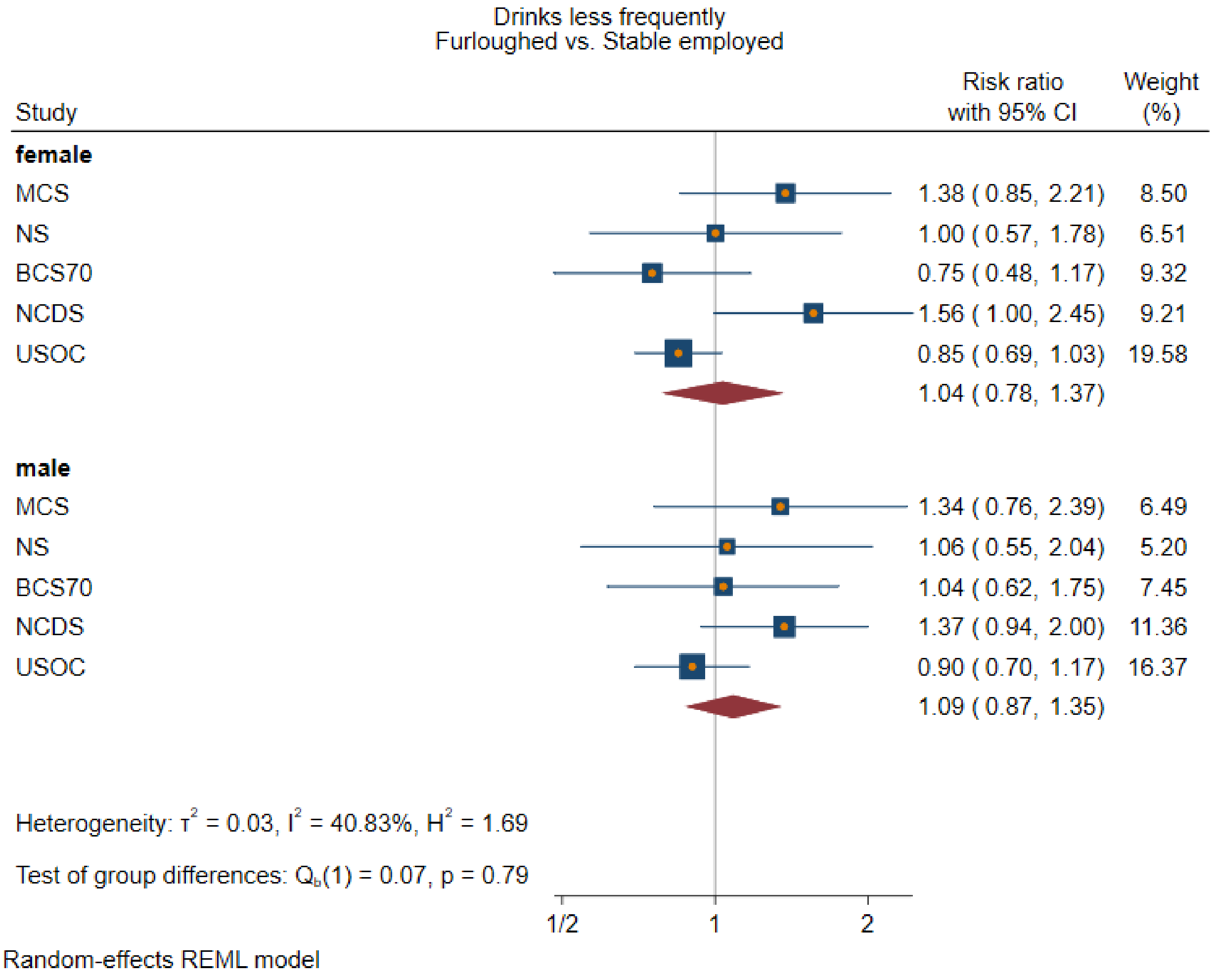

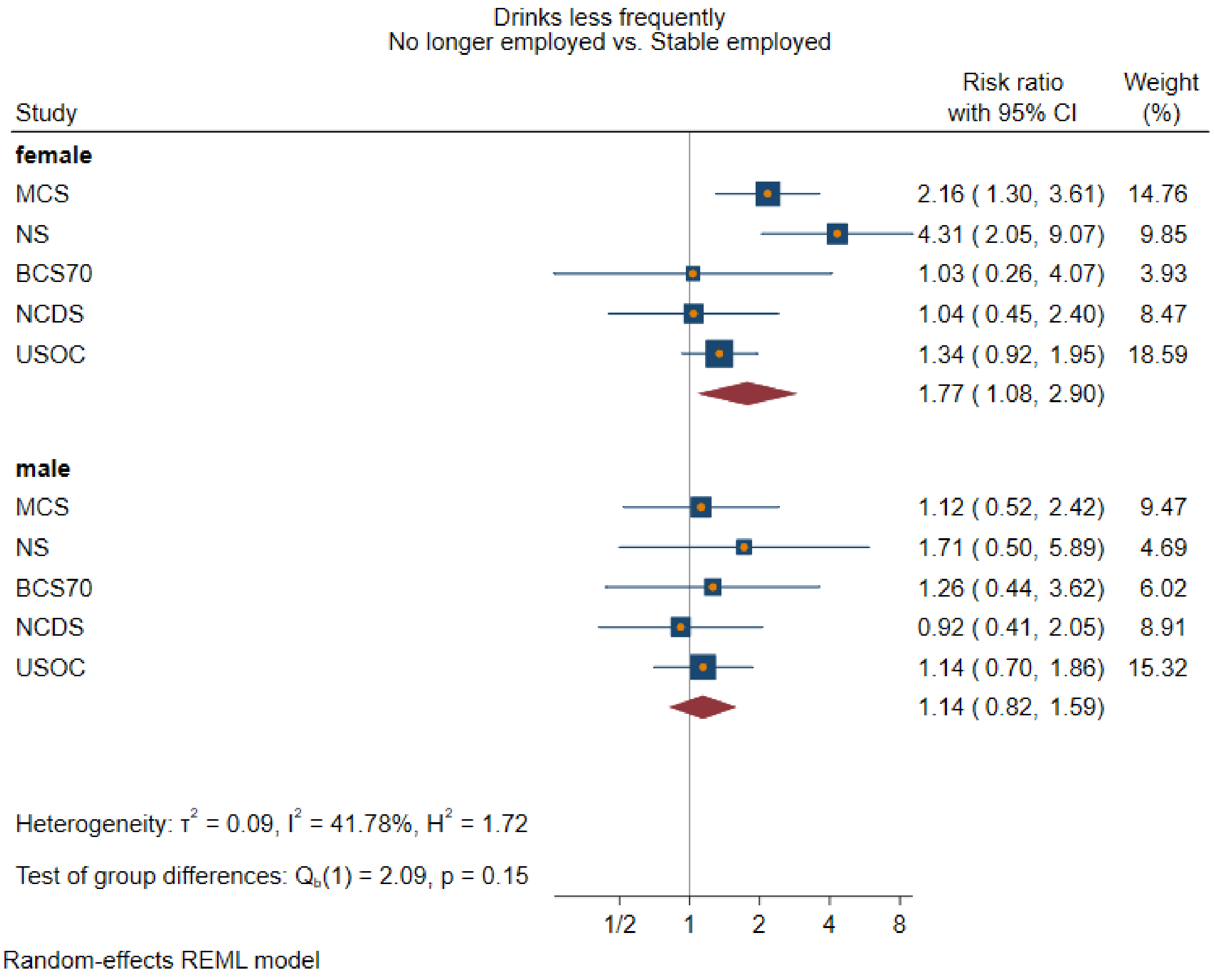

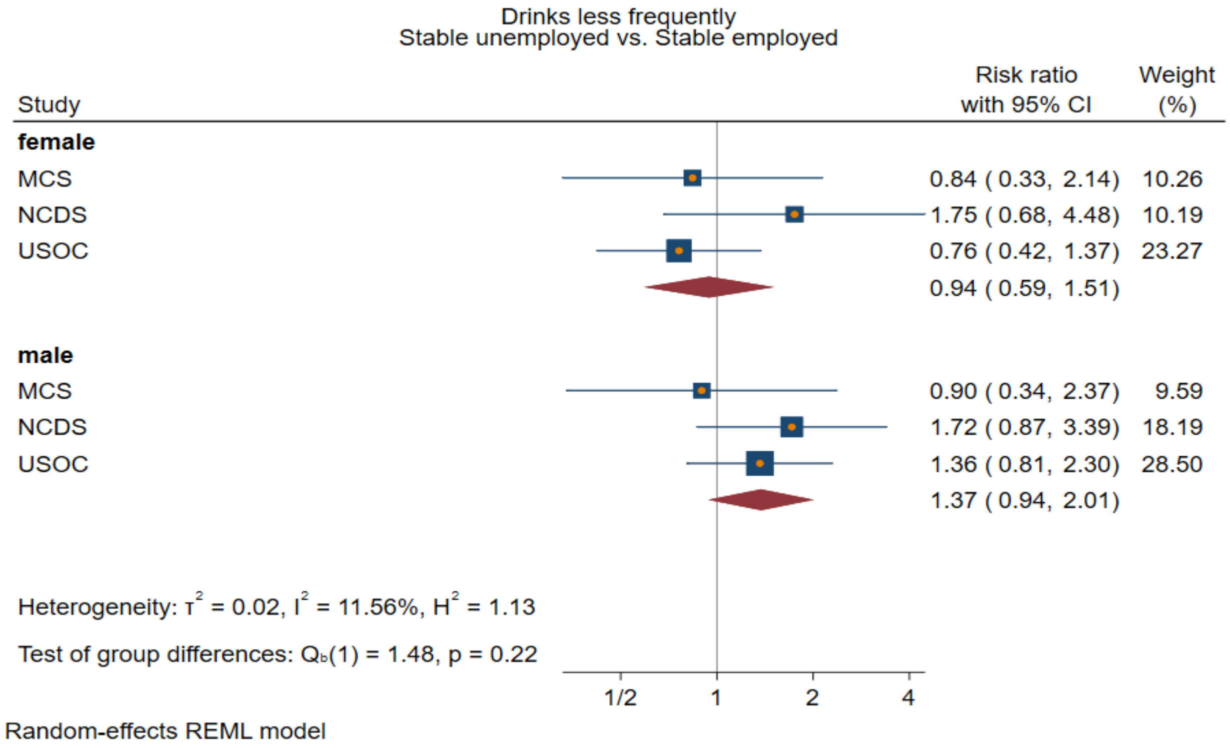
Drinks less frequently
Stable unemployed vs. Stable employed
Study
Risk ratio with 95% CI
Weight (%)
female
MCS 0.84 (0.33, 2.14) 10.26
NCDS 1.75 (0.68, 4.48) 10.19
USOC 0.76 (0.42, 1.37) 23.27
0.94 (0.59, 1.51)
male
MCS 0.90 (0.34, 2.37) 9.59
NCDS 1.72 (0.87, 3.39) 18.19
USOC 1.36 (0.81, 2.30) 28.50
1.37 (0.94, 2.01)
Heterogeneity: τ² = 0.02, I² = 11.56%, H² = 1.13
Test of group differences: Qb(1) = 1.48, p = 0.22
1/2
1
2
4
Random-effects REML model

Figure set 40: Current smoker

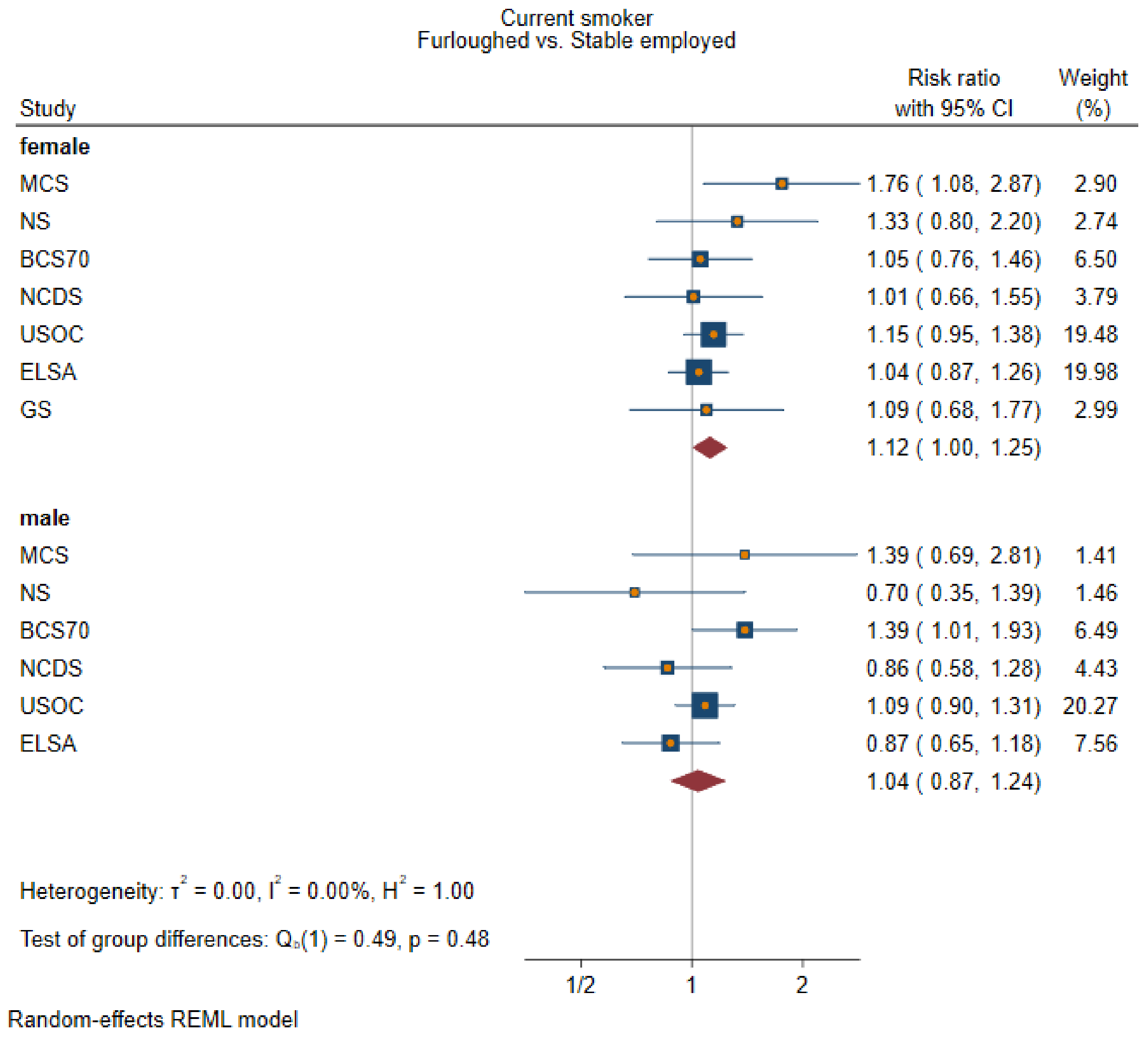

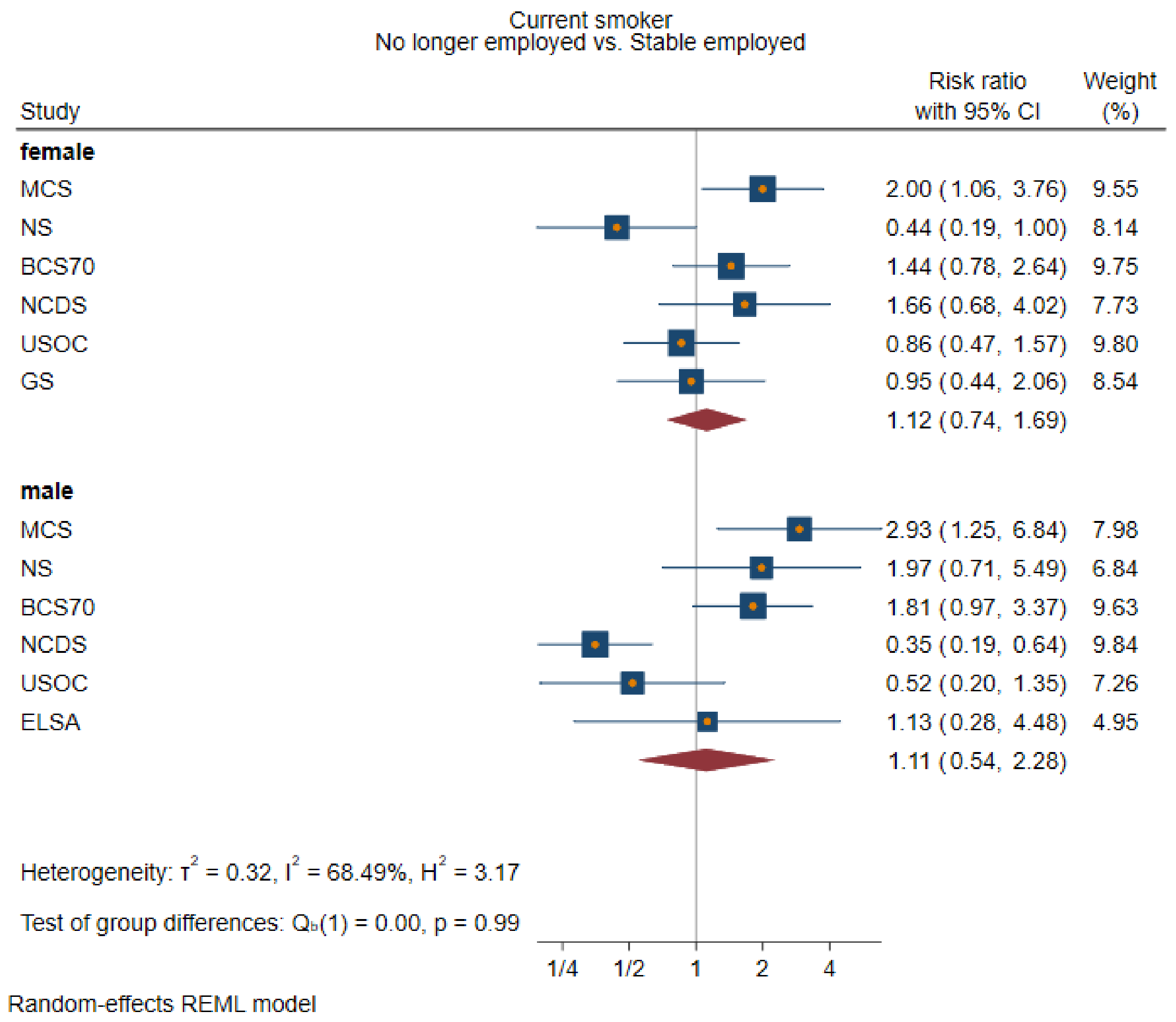

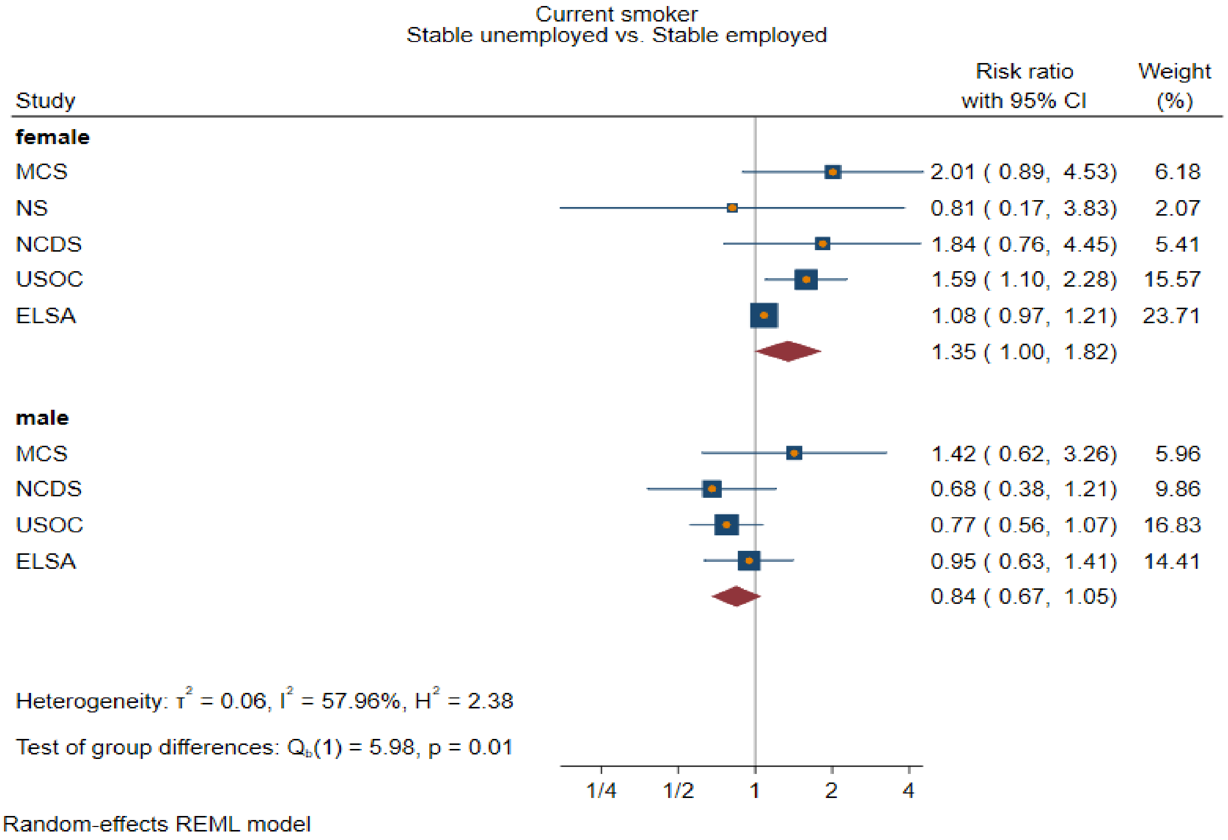
Current smoker
Stable unemployed vs. Stable employed
Study
Risk ratio with 95% CI
Weight (%)
female
MCS 2.01 (0.89, 4.53) 6.18
NS 0.81 (0.17, 3.83) 2.07
NCDS 1.84 (0.76, 4.45) 5.41
USOC 1.59 (1.10, 2.28) 15.57
ELSA 1.08 (0.97, 1.21) 23.71
1.35 (1.00, 1.82)
male
MCS 1.42 (0.62, 3.26) 5.96
NCDS 0.68 (0.38, 1.21) 9.86
USOC 0.77 (0.56, 1.07) 16.83
ELSA 0.95 (0.63, 1.41) 14.41
0.84 (0.67, 1.05)
Heterogeneity: τ^2 = 0.06, I^2 = 57.96%, H^2 = 2.38
Test of group differences: $Q_b(1)$ = 5.98, p = 0.01
1/4
1/2
1
2
4
Random-effects REML model

Figure set 41: Smoking more

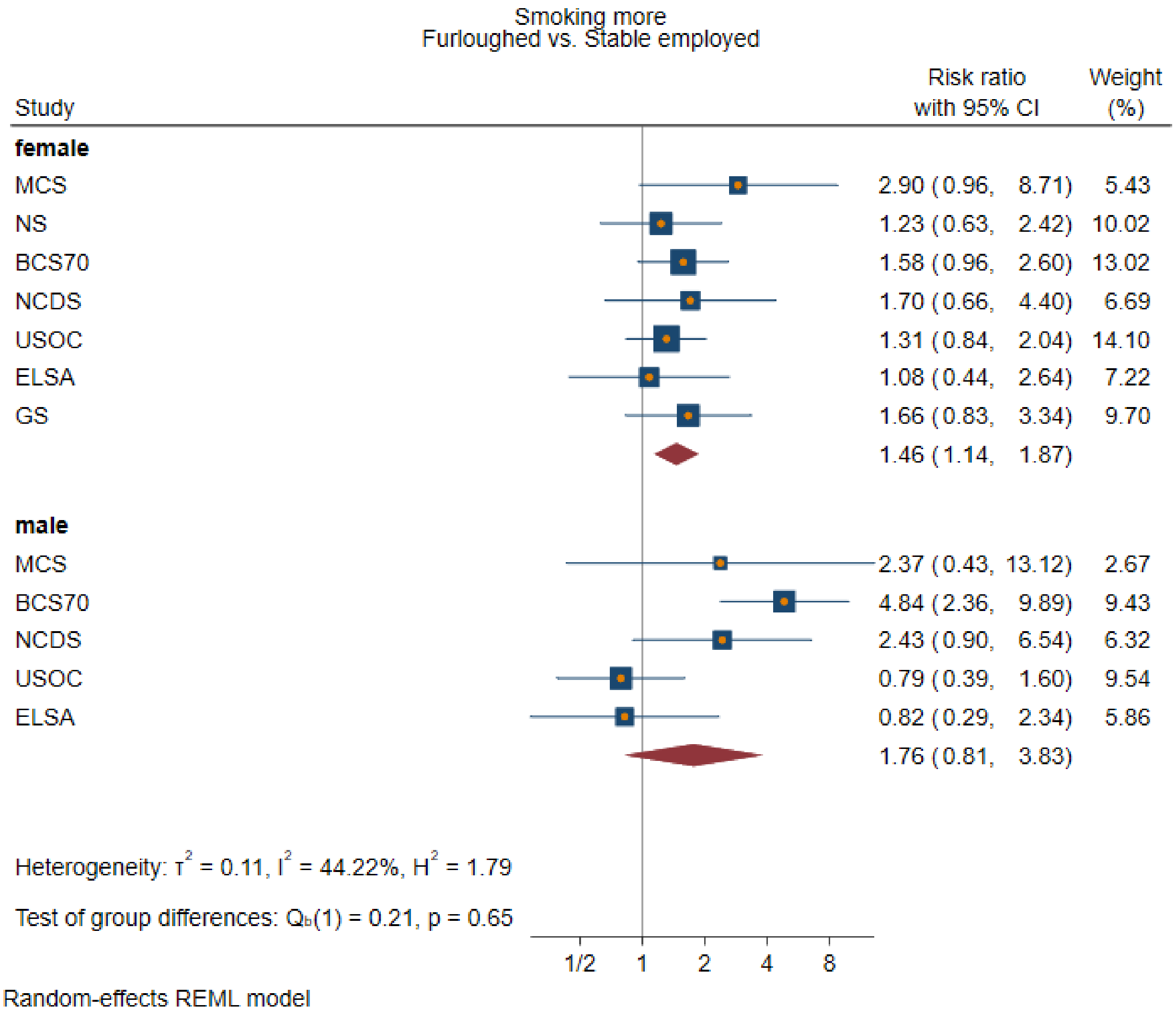

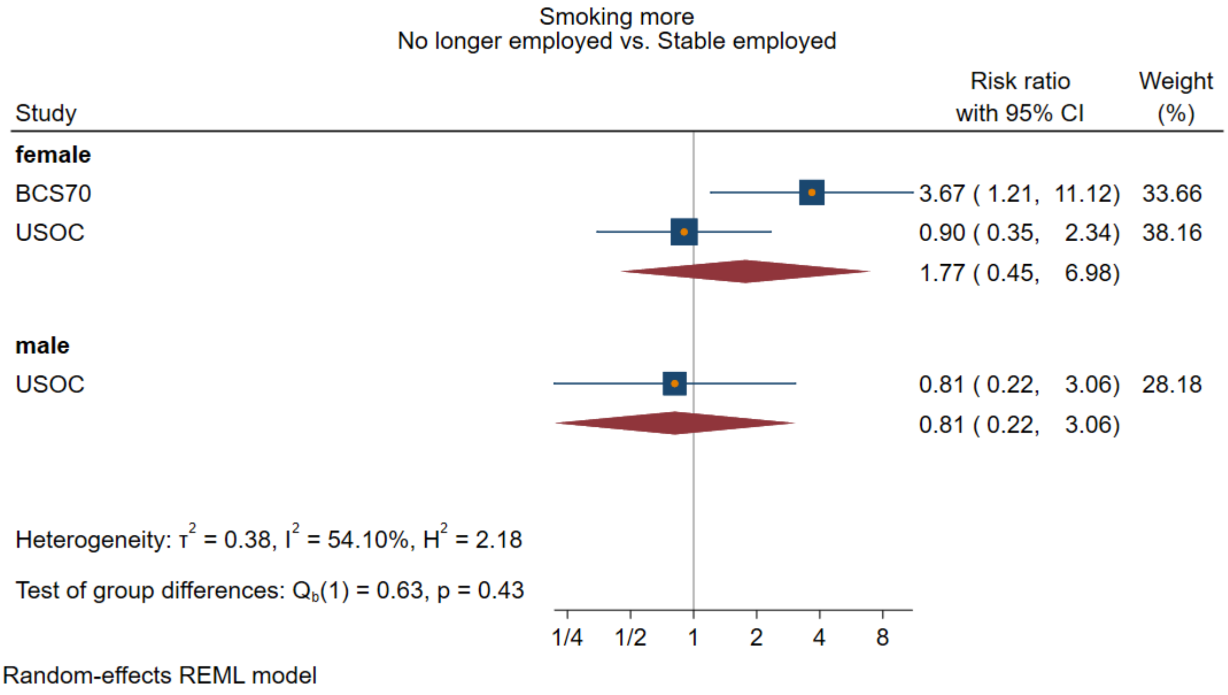

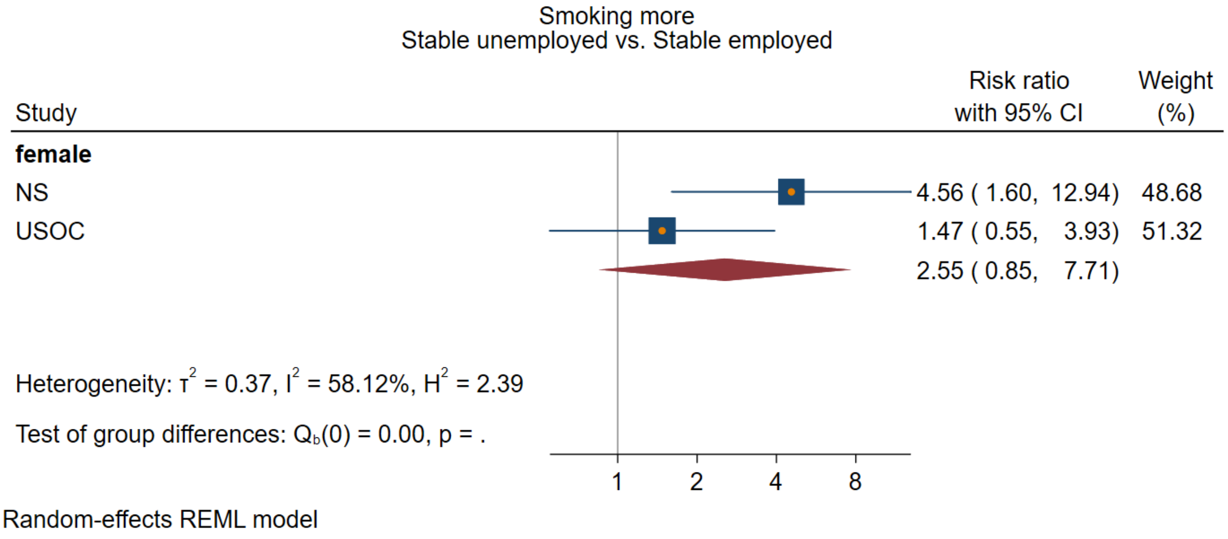
Smoking more
Stable unemployed vs. Stable employed
Study
Risk ratio with 95% CI
Weight (%)
female
NS
4.56 (1.60, 12.94)
48.68
USOC
1.47 (0.55, 3.93)
51.32
2.55 (0.85, 7.71)
Heterogeneity: τ² = 0.37, I² = 58.12%, H² = 2.39
Test of group differences: Qb(0) = 0.00, p = .
1
2
4
8
Random-effects REML model

Figure set 42: Smoking less

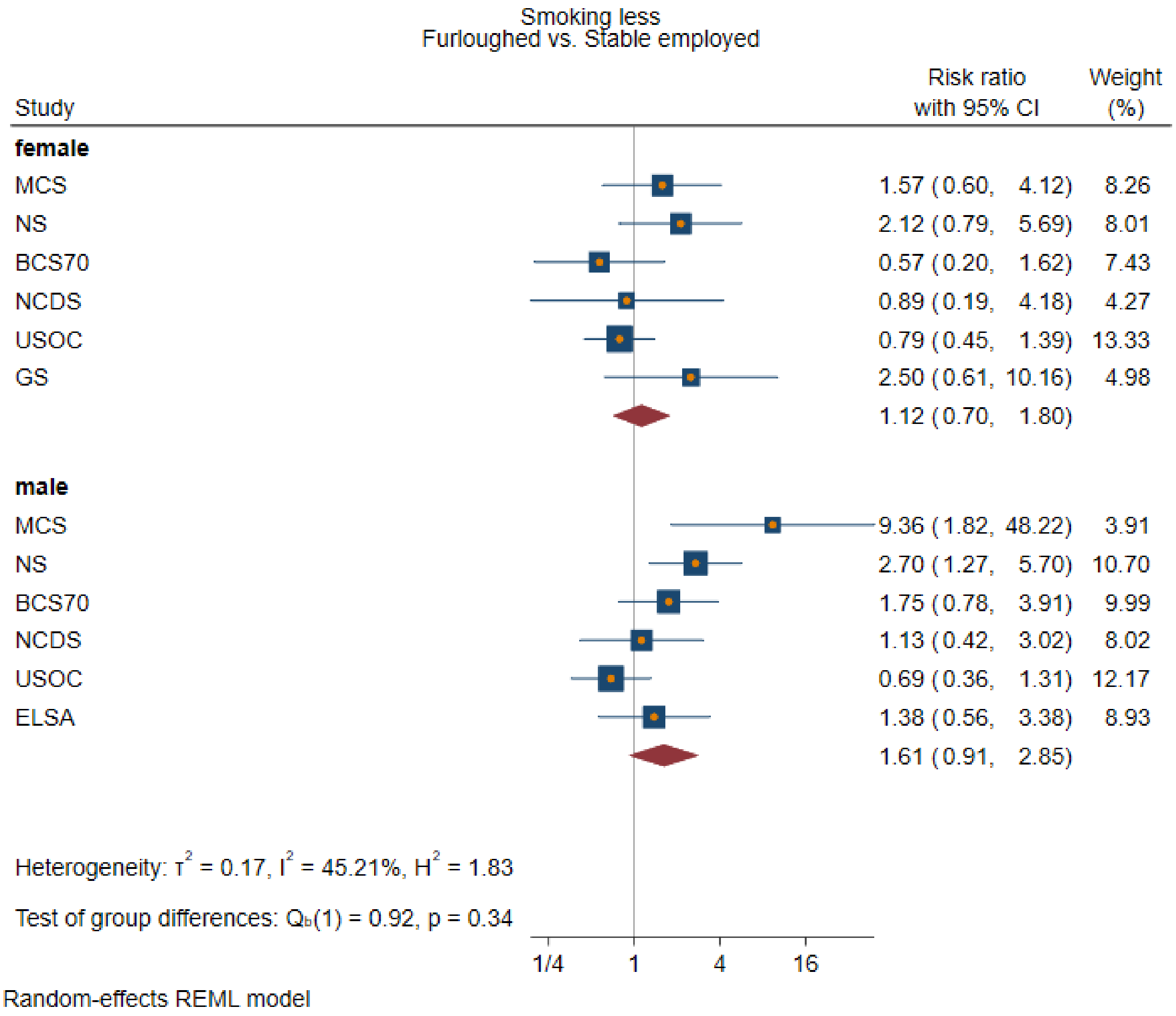

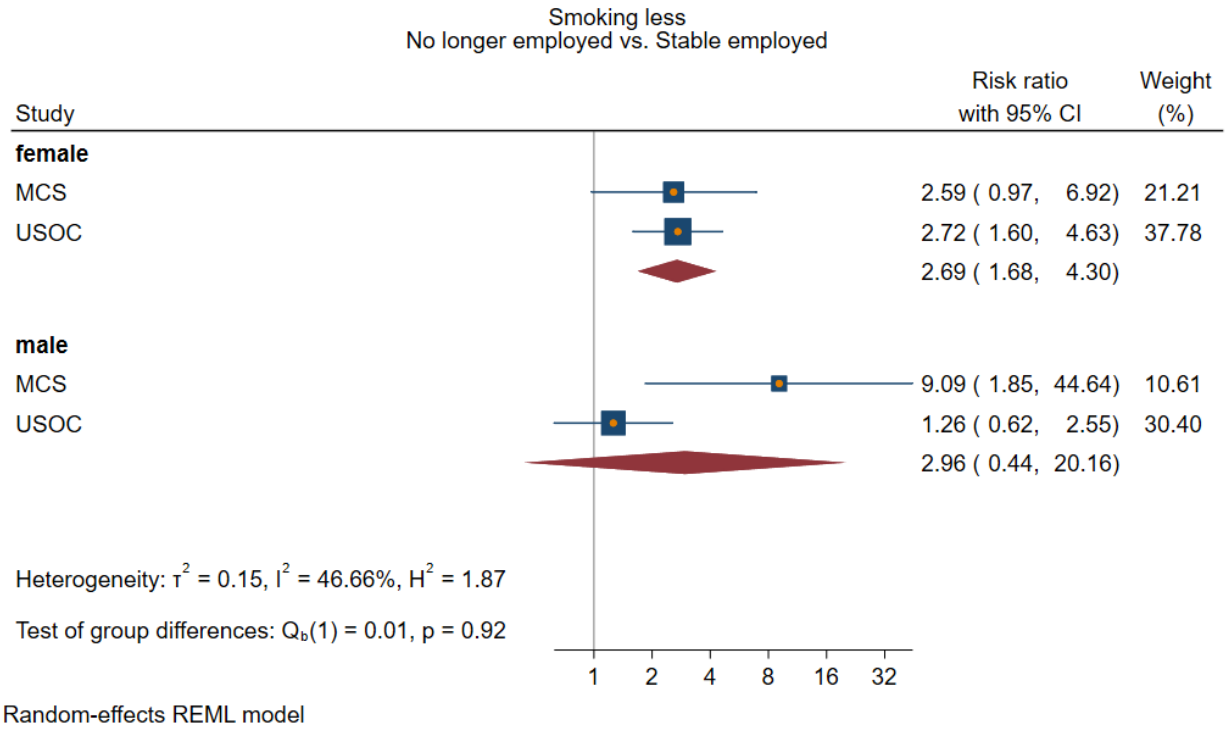

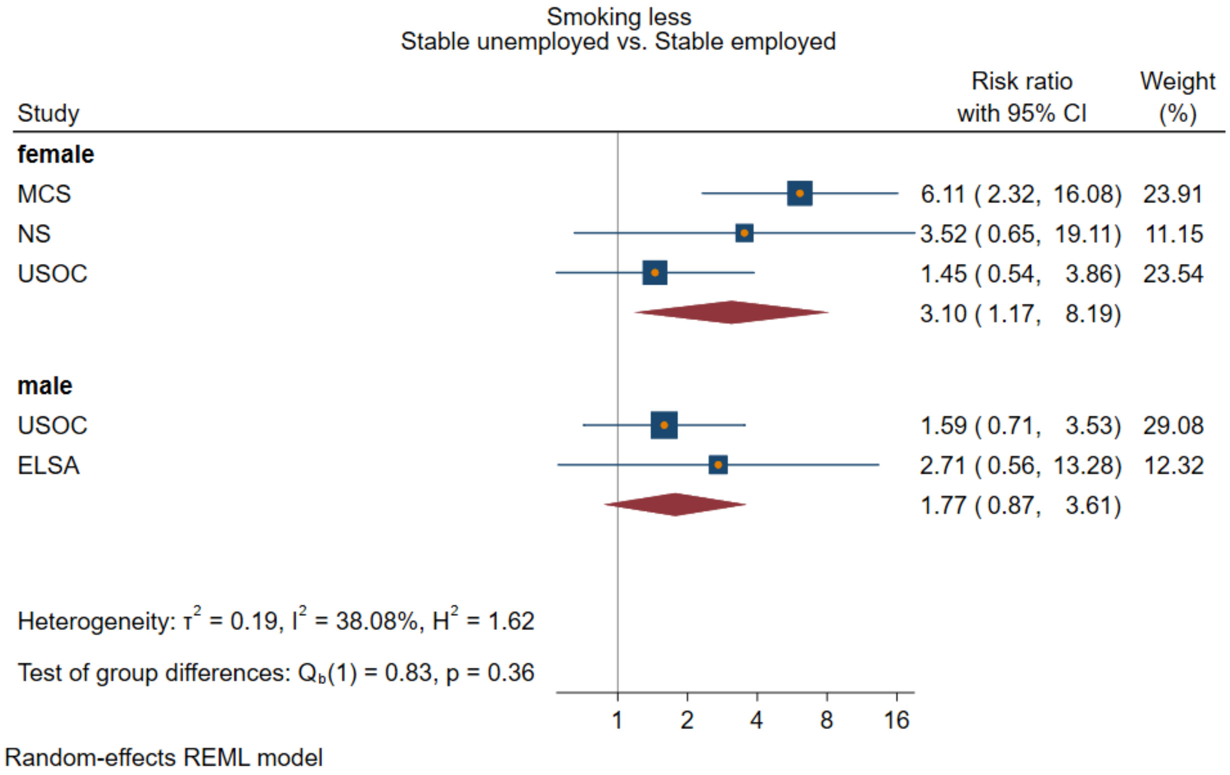
Smoking less
Stable unemployed vs. Stable employed
Study
Risk ratio with 95% CI
Weight (%)
female
MCS 6.11 (2.32, 16.08) 23.91
NS 3.52 (0.65, 19.11) 11.15
USOC 1.45 (0.54, 3.86) 23.54
3.10 (1.17, 8.19)
male
USOC 1.59 (0.71, 3.53) 29.08
ELSA 2.71 (0.56, 13.28) 12.32
1.77 (0.87, 3.61)
Heterogeneity: τ² = 0.19, I² = 38.08%, H² = 1.62
Test of group differences: Q_b(1) = 0.83, p = 0.36
1
2
4
8
16
Random-effects REML model

Figure set 43: Current vaper

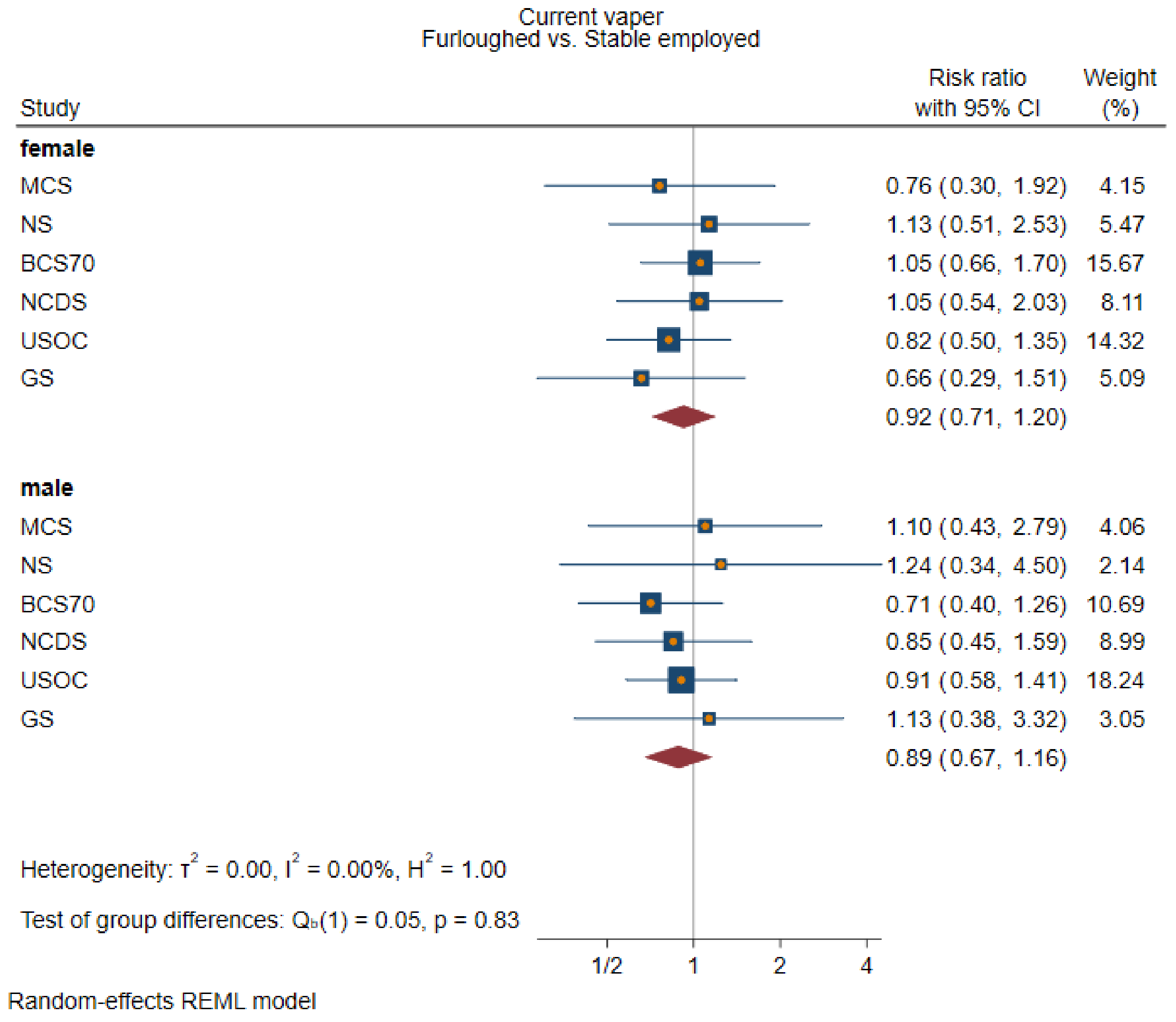

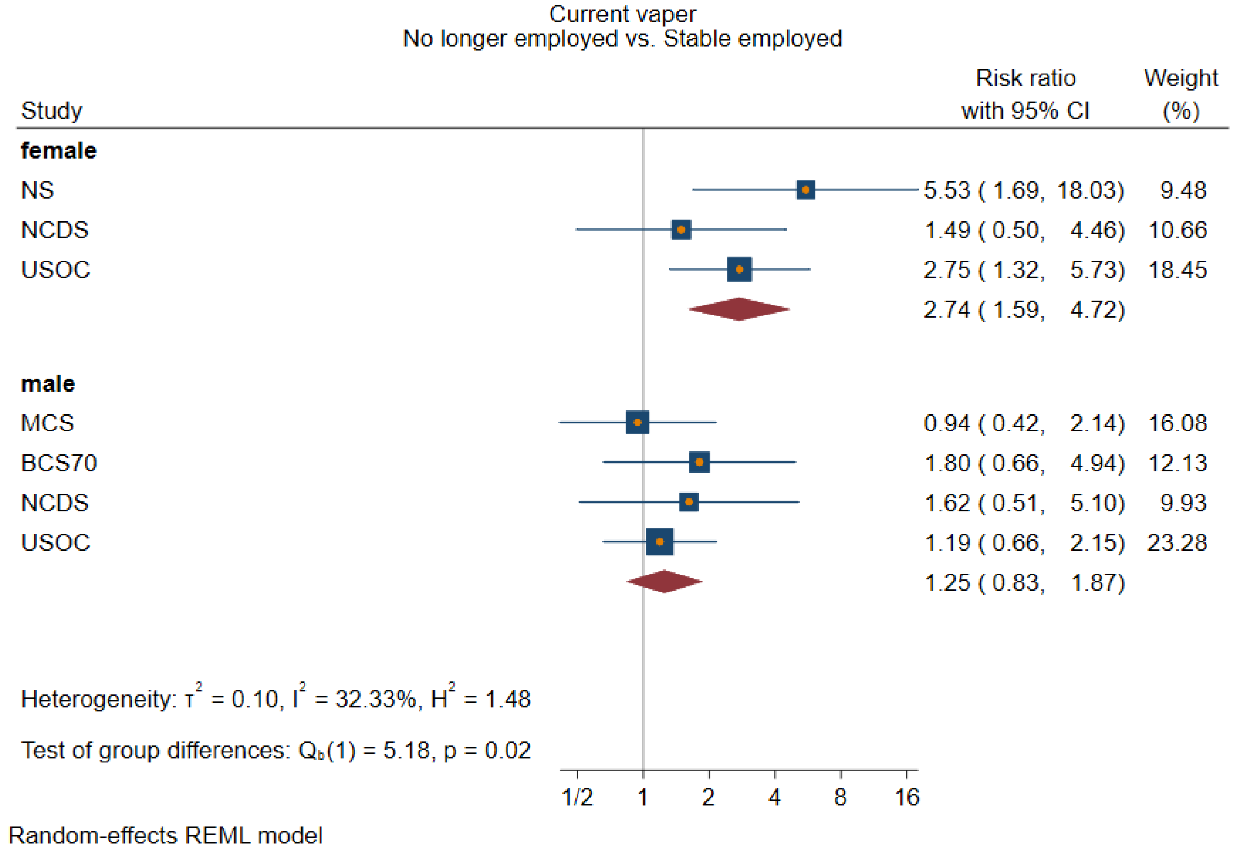

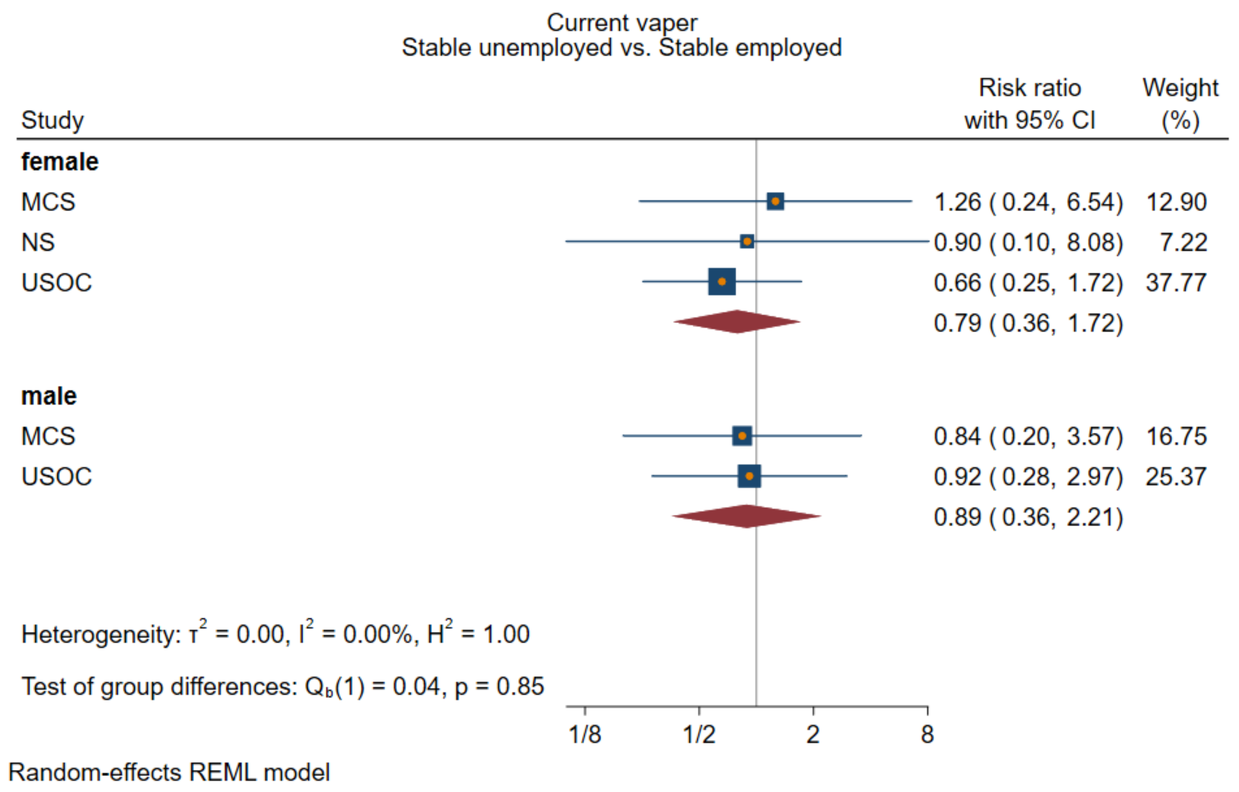
Current vaper
Stable unemployed vs. Stable employed
Study
Risk ratio with 95% CI
Weight (%)
female
MCS 1.26 (0.24, 6.54) 12.90
NS 0.90 (0.10, 8.08) 7.22
USOC 0.66 (0.25, 1.72) 37.77
0.79 (0.36, 1.72)
male
MCS 0.84 (0.20, 3.57) 16.75
USOC 0.92 (0.28, 2.97) 25.37
0.89 (0.36, 2.21)
Heterogeneity: τ² = 0.00, I² = 0.00%, H² = 1.00
Test of group differences: Q_b(1) = 0.04, p = 0.85
1/8
1/2
2
8
Random-effects REML model

Figure set 44: Vaping more

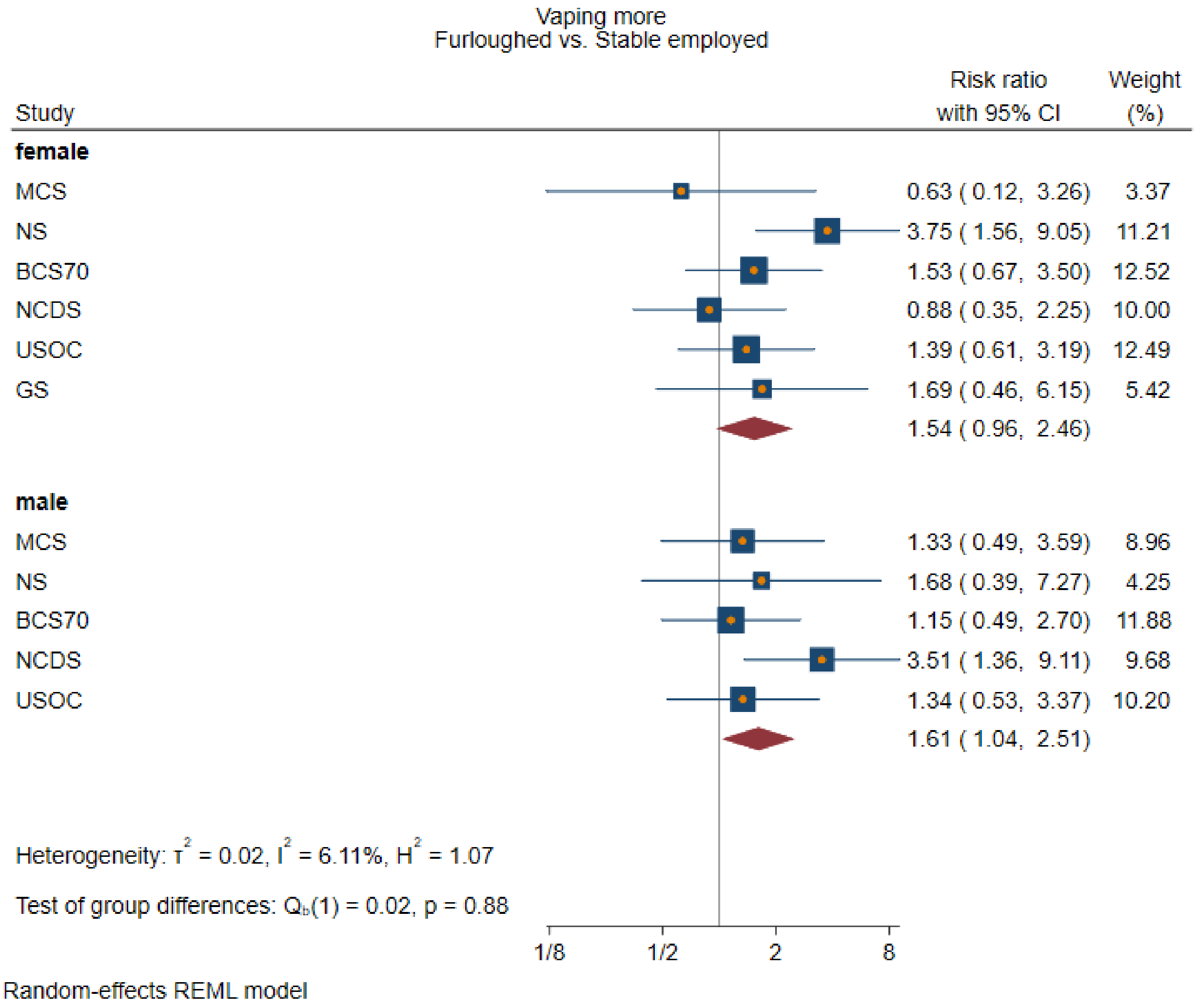

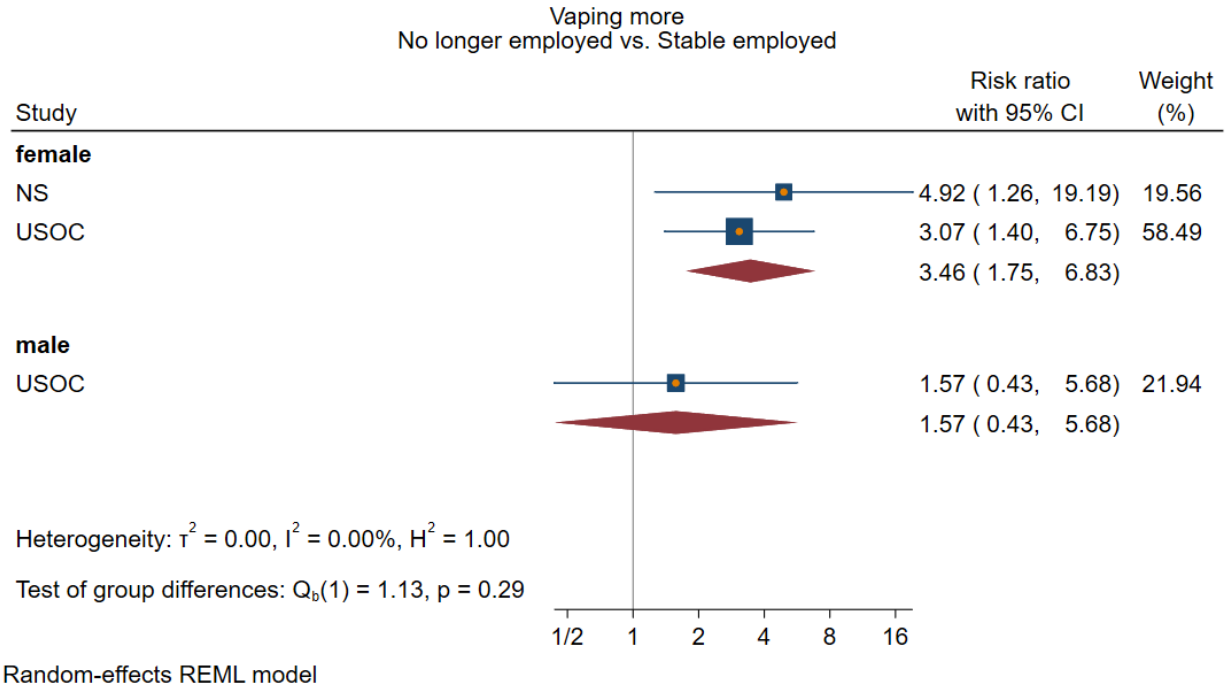

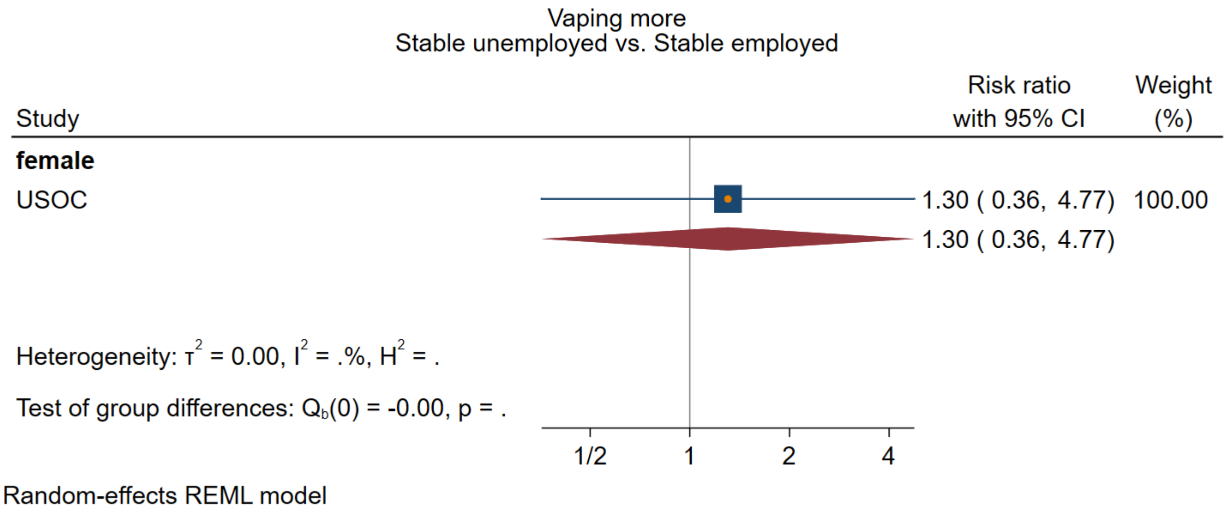
Vaping more
Stable unemployed vs. Stable employed
Study
Risk ratio with 95% CI
Weight (%)
female
USOC
1.30 (0.36, 4.77) 100.00
1.30 (0.36, 4.77)
Heterogeneity: τ² = 0.00, I² = .%, H² = .
Test of group differences: Q_b(0) = -0.00, p = .
1/2
1
2
4
Random-effects REML model

Figure set 45: Vaping less

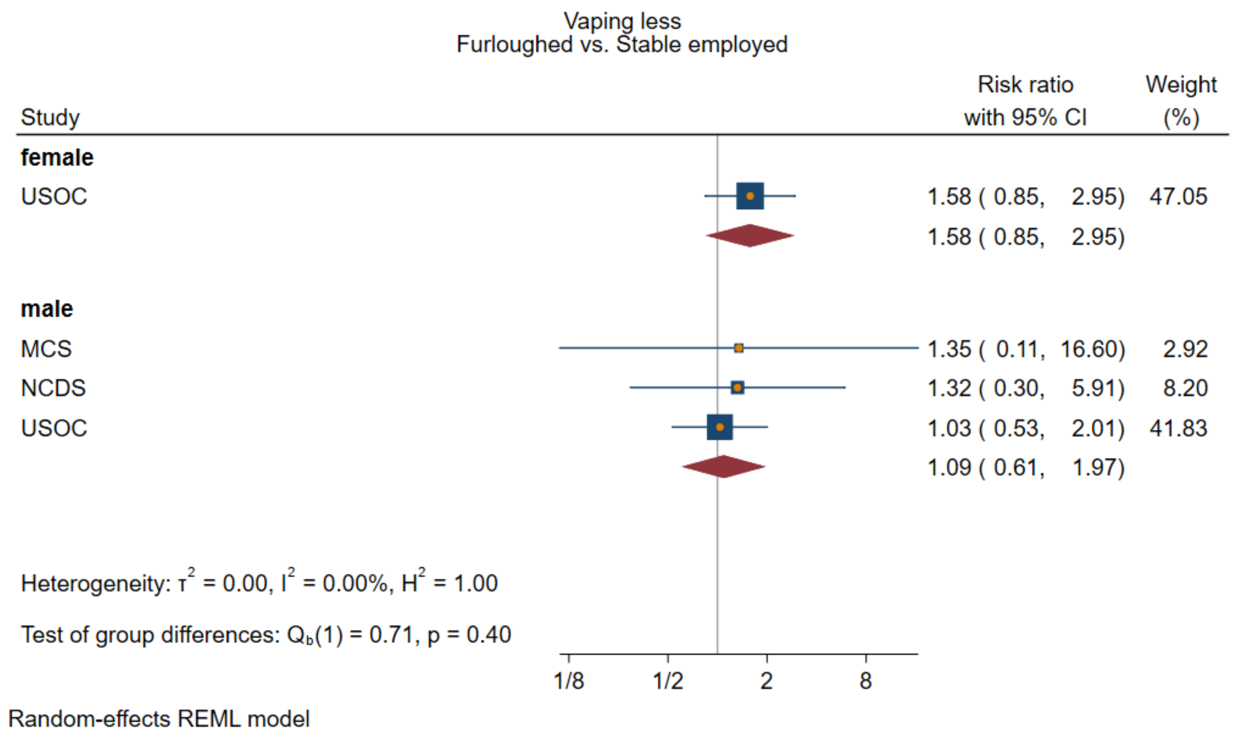

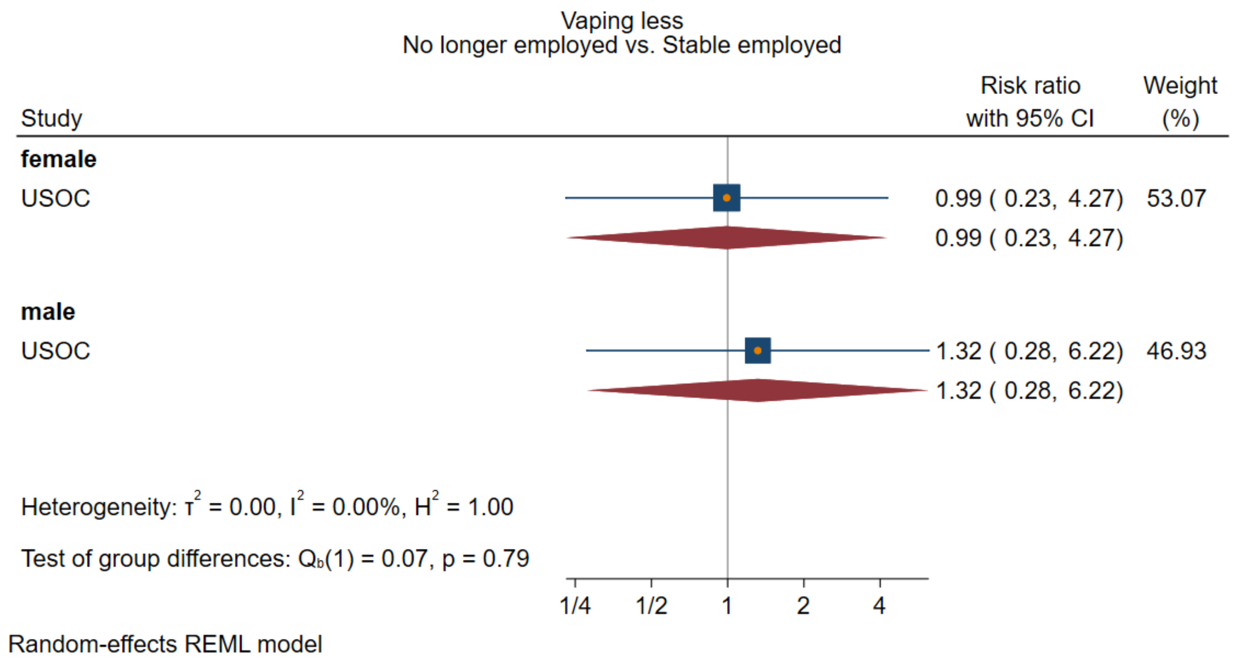

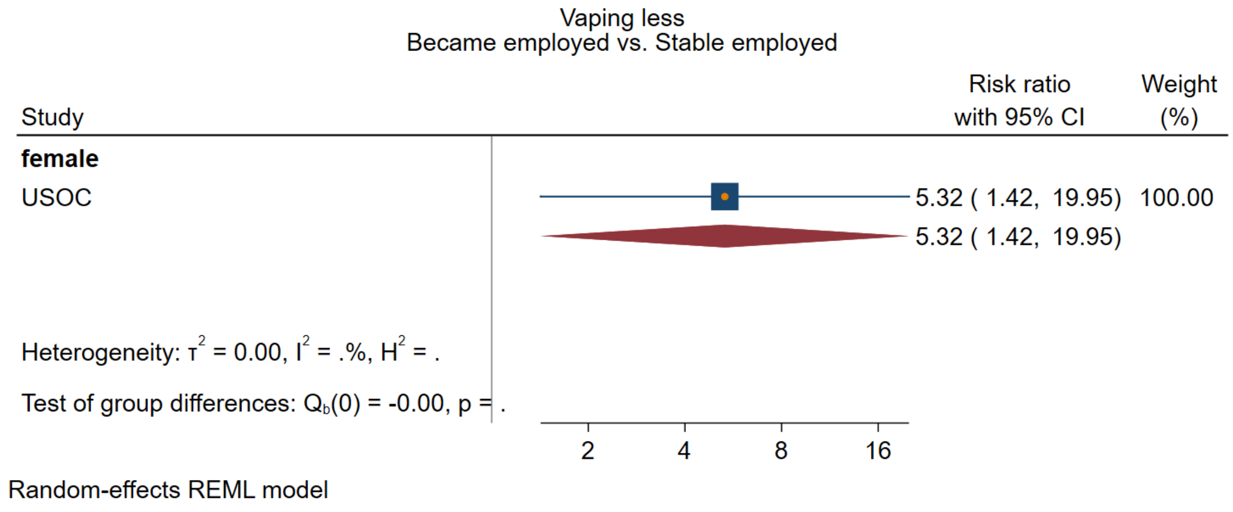

Vaping less
Became employed vs. Stable employed
Study
Risk ratio with 95% CI
Weight (%)
female
USOC
5.32 (1.42, 19.95)
100.00
5.32 (1.42, 19.95)
Heterogeneity: τ² = 0.00, I² = .%, H² = .
Test of group differences: Q_b(0) = -0.00, p = .
2
4
8
16
Random-effects REML model
